# Stn1 promotes zebrafish oocyte development via amplifying Wnt/β-catenin signaling

**DOI:** 10.1038/s44319-026-00775-8

**Published:** 2026-04-17

**Authors:** Xin Zhang, Yanpeng Zhai, Caixia Wang, Yuxue He, Yuxin He, Bo Wang, Chensong Huang, Aibo Sheng, Yan Bai, Xiaozhi Rong, Jianfeng Zhou

**Affiliations:** 1 Key Laboratory of Marine Drugs (Ocean University of China), Chinese Ministry of Education, and School of Medicine and Pharmacy, Ocean University of China, Qingdao, China; 2 Laboratory for Marine Drugs and Bioproducts, Qingdao Marine Science and Technology Center, Qingdao, China

**Keywords:** Development, Signal Transduction, Stem Cells & Regenerative Medicine

## Abstract

Wnt/β-catenin signaling regulates oocyte development in vertebrates. However, the dynamics of Wnt/β-catenin signaling in oocyte progression remain unclear. Here, we analyze oocytes across different developmental stages in zebrafish and find that the activity of Wnt/β-catenin signaling sharply increases when oocytes reach a size of 10 to 45 μm (stage IA to middle stage IB) and subsequently declines rapidly. In addition, we show that expression of the CST complex subunit Stn1 is enriched in germ cells. Stn1 interacts with the transcription factor Tcf/Lef, facilitates its association with promoters of germ cell-specific genes, thereby enhancing Wnt/β-catenin signaling activity in oocytes. Genetic deletion of *stn1* leads to massive loss of oocytes prior to or during their development to stage IB, resulting in a male-like phenotype associated with infertility. Temporal activation of the Wnt/β-catenin signaling pathway partially restores germ cell loss and facilitates oocyte development to stage IB. Our findings highlight the importance of Wnt/β-catenin signaling in promoting the expression of germ cell-specific genes and provide novel insights into the physiological function of Stn1 in maintaining oocyte development in zebrafish.

## Synopsis

Wnt/β-catenin signaling activation fluctuates in developing oocytes during the early primary growth stage in zebrafish. The CST complex subunit Stn1 is enriched in germ cells, interacts with the transcription factor Tcf/Lef, and facilitates its binding to the promoters of germ cell-specific genes, thereby enhancing pathway activity at the appropriate developmental time to ensure proper oocyte development.

- The activity of the Wnt/β-catenin signaling pathway sharply increases and subsequently declines in developing oocytes during the early primary growth stage in zebrafish.
- The expression of *stn1* is highly enriched in developing oocytes, and loss of Stn1 in zebrafish reduces Wnt/β-catenin signaling activity in germ cells and impairs germ cell development.
- Stn1 interacts with Tcf/Lef to facilitate the transcription of various germ cell-specific genes.
- Temporally induced activation of the Wnt/β-catenin signaling pathway partially restores oocyte development in *stn1* mutants.

## Abstract (web summary)

Wnt/β-catenin signaling activation fluctuates in developing oocytes during the early primary growth stage in zebrafish. The CST complex subunit Stn1 is enriched in germ cells, interacts with the transcription factor Tcf/Lef, and facilitates its binding to the promoters of germ cell-specific genes, thereby enhancing pathway activity at the appropriate developmental time to ensure proper oocyte development.

## Graphical abstract

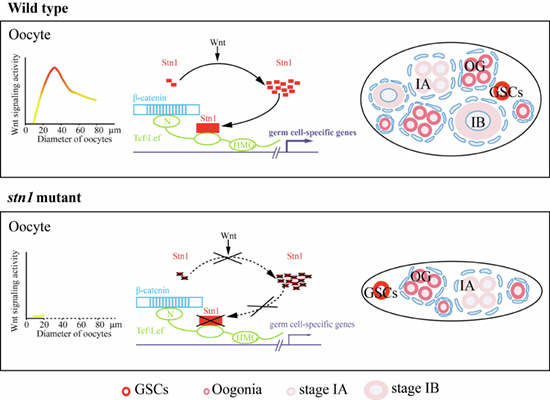

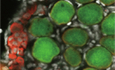

## Introduction

Germ cells have the unique capacity to generate gametes (sperm and oocytes), which are essential for all sexually reproducing organisms. In ovaries, the germ cell-intrinsic activity of the Wnt/β-catenin signaling pathway (also known as canonical Wnt signaling pathway), which regulates their development, is evolutionarily conserved across the animal kingdom (Le Rolle et al, 2021; Takase and Nusse, 2016). In the mouse embryonic ovary, germ cell-specific Wnt/β-catenin activity is crucial for pluripotent maintenance. Reduction in Wnt/β-catenin activity leads to differentiation and meiotic entry of germ cells(Le Rolle et al, 2021). In zebrafish, Wnt/β-catenin signaling plays important roles in the regulation of development and regeneration of ovaries (Cao et al, 2019; Kossack et al, 2019; Liu et al, 2022; Sreenivasan et al, 2014). Thus, the intrinsic activity of Wnt/β-catenin signaling is precisely and dynamically controlled in the progression of germ cells; however, the dynamic profile of Wnt/β-catenin activity, its action, and how it is regulated during the progression are not clearly understood.

The Wnt/β-catenin signaling pathway is an evolutionarily conserved transduction cascade and is critical in numerous biological processes, including cell proliferation and cell fate determination during embryonic and post-embryonic development (Holzem et al, 2024; Nusse and Clevers, 2017). In the absence of Wnt ligands, the destruction complex, which includes Axin1/2, APC, GSK3β, and CK1α, targets and phosphorylates cytoplasmic β-catenin, which allows it for proteasomal degradation. Upon Wnt activation, secreted Wnt ligands bind to and form a ternary complex with the frizzled transmembrane receptor family and LRP5/6 co-receptors, triggering signalosome formation that prevents β-catenin phosphorylation by inhibiting GSK3β activity, leading to β-catenin stabilization. Consequently, β-catenin accumulates in the cytoplasm and enters the nucleus. In the nucleus, β-catenin interacts with TCF/LEF transcription factors to activate the expression of Wnt target genes (Clevers and Nusse, 2012; Niehrs et al, 2024; Nusse and Clevers, 2017). However, relatively little is known about how the β-catenin/Tcf transactivation complex regulates germ cell development by controlling the transcription of specific target genes in germ cells.

CST complex subunit STN1, also known as OBFC1 or AAF44, is a component of the trimeric Ctc1–Stn1–Ten1 (CST) complex, which is essential for telomere maintenance and resolution of stalled replication forks (Bhattacharjee et al, 2017; Chastain et al, 2016; Chen et al, 2012; Miyake et al, 2009; Stewart et al, 2012; Surovtseva et al, 2009; Wang et al, 2012; Zhang et al, 2019). The CST complex functions as a negative regulator of telomerase activity, and its depletion results in excessive telomerase activity and dysregulation of telomere length (Chen et al, 2012; Zaug et al, 2021). Patients with point mutations in *STN1* exhibit intrauterine growth retardation and premature aging pathological features later in life (Simon et al, 2016). However, the in vivo functions of STN1 and the molecular mechanisms underlying its activity remain poorly understood.

Zebrafish is an excellent vertebrate model to study cellular and molecular mechanisms of germ cell specification, maintenance, and differentiation (Aharon and Marlow, 2021). To investigate the activity of Wnt/β-catenin signaling in oocytes in real-time, we utilized the oogenesis of zebrafish with a transgenic Wnt-response EGFP background as a model and monitored the intrinsic Wnt/β-catenin signaling in the progression of oocytes. Here, we showed that the activity of Wnt/β-catenin signaling sharply increases in the oocytes with size from 10 to 45 μm (corresponding to the early primary growth stage, or stage IA to the mid-stage IB) and then dives rapidly. In addition, we found that Stn1, which is highly enriched in developmental germ cells, enhances the activity of Wnt/β-catenin signaling in the early primary growth stage of oocytes. Genetic deletion of *stn1* impairs gonad development, resulting in a male-like phenotype associated with infertility. Mechanistically, activated Wnt/β-catenin signaling stabilizes Stn1, which functions as a binding partner of Tcf/Lef and facilitates the activity of the transcriptional complex, which in turn amplifies Wnt/β-catenin signaling activity and maintains oocytes at stage IB. In particular, we also showed that various germ cell-specific genes, including *ddx4*, *dnd1*, *piwil1*, *piwil2*, *tdrd1*, *tdrd7a*, and *tdrd9*, represent Wnt/β-catenin direct target genes. Collectively, our findings suggest that Wnt/β-catenin signaling promotes oocyte progression during the early primary growth stage, in which Stn1 enhances pathway activity at the appropriate time to ensure proper oocyte development.

## Results

### The activity of the Wnt/β-catenin signaling pathway sharply increases in developing oocytes with size from 10 to 45 μm and then rapidly declines in juvenile zebrafish

**Figure 1:**
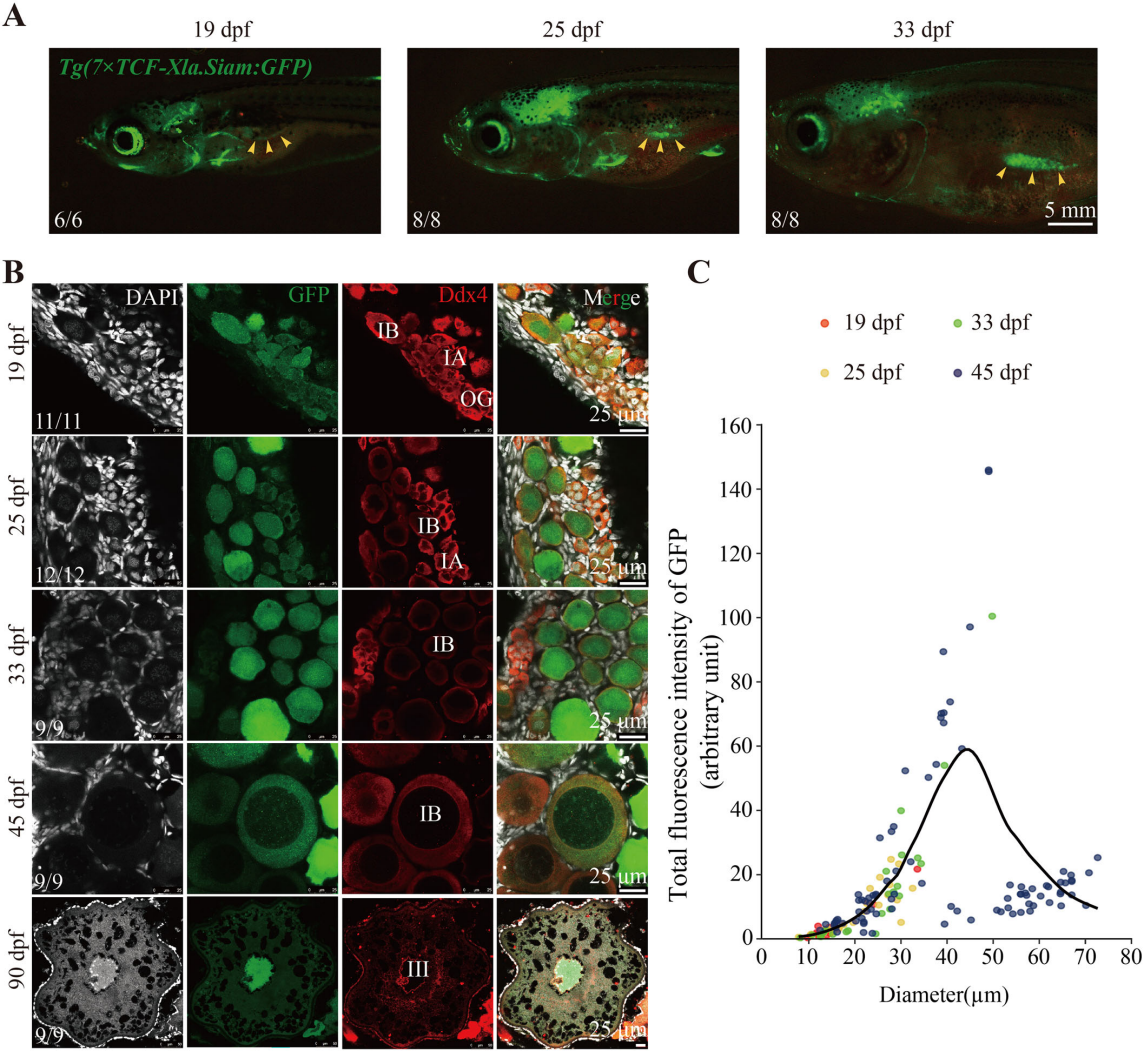
GFP expression in the gonads of juvenile zebrafish with a *Tg(7*×*TCF-Xla.Siam:GFP)* transgenic background during growth processes. (**A**) Representative images through the body wall showing GFP^+^ tissues of juvenile zebrafish with a *Tg(7*×*TCF-Xla.Siam:GFP)* transgenic background at the indicated time points. Yellow arrowheads indicate the gonads. Scale bar: 5 mm. The frequency of zebrafish with the indicated phenotypes is shown in the bottom left corner of each panel. (**B**) Representative confocal images of gonads of female fish with a *Tg(7*×*TCF-Xla.Siam:GFP)* transgenic background at the indicated time points were immunostained with anti-Ddx4 antibodies. OG oogonia, IA stage IA, IB stage IB, III stage III. Scale bar: 25 μm. The proportion of sections with the indicated phenotypes is shown in the bottom left corner of each panel. Each section was obtained from an individual zebrafish. The number of zebrafish analyzed at different time points was as follows: 11 at 19 dpf, 12 at 25 dpf, 9 at 33 dpf, 9 at 45 dpf, and 9 at 90 dpf. (**C**) Quantitative results from images shown in (**B**). Dot plot showing the total GFP intensity of germ cell. Each data point represents an individual oocyte. Source data are available online for this figure.

**Figure EV1:**
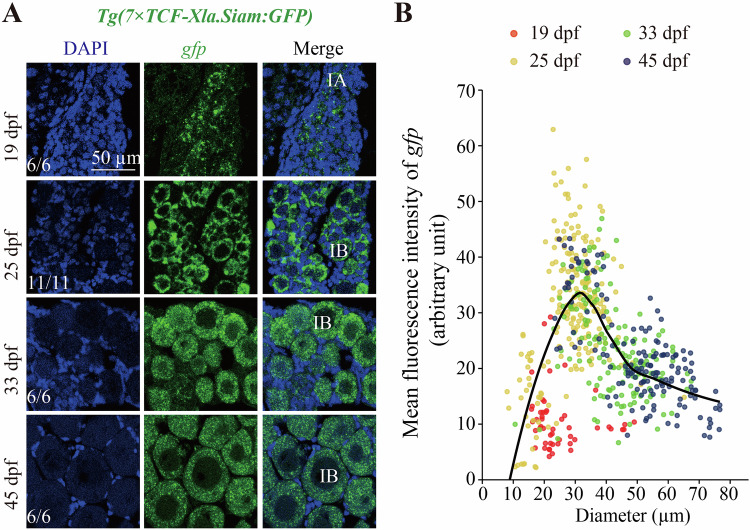
The *gfp* mRNA levels in the gonads of juvenile zebrafish with a *Tg(7*×*TCF-Xla.Siam:GFP)* transgenic background during growth processes. (**A**) Representative confocal images of gonads of female fish with a *Tg(7*×*TCF-Xla.Siam:GFP)* transgenic background at the indicated time points following staining for *gfp* mRNA. IA stage IA, IB stage IB. Scale bar: 50 μm. The proportion of sections with the indicated phenotypes is shown in the bottom left corner of each panel. Each section was obtained from an individual zebrafish. (**B**) Quantitative results from images shown in (**A**). Dot plot showing the mean fluorescence of *gfp* mRNA of germ cells. Each data point represents an individual oocyte. The number of zebrafish counted at different time points was 6 at 19 dpf, 11 at 25 dpf, 6 at 33 dpf, and 6 at 45 dpf. Source data are available online for this figure.

Wnt/β-catenin signaling is a key regulator of germ cell development, sex determination, and ovary regeneration in vertebrates (Cao et al, 2019; Chassot et al, 2011; Kossack et al, 2019; Le Rolle et al, 2021; Liu et al, 2022; Naillat et al, 2010; Sreenivasan et al, 2014; Vainio et al, 1999). However, the dynamic profile of Wnt/β-catenin signaling activity during oocyte development at different stages was not investigated. To address this question, we monitored the real-time activity of Wnt/β-catenin signaling in oocytes at different developmental stages using a transgenic zebrafish Wnt/β-catenin reporter line, *Tg*(*7*×*TCF-Xla.Siam:GFP)*. Associated GFP expression is driven by a promoter with seven Tcf/Lef-binding sites upstream of the minimal promoter of the *Xenopus* direct Wnt/β-catenin target gene *siamois*, facilitating the identification of signal-responsive cells and allowing for quantification of signaling activity through measurement of GFP protein or mRNA levels per cell (Moro et al, 2012). The GFP signal was enriched in the gonad of the zebrafish Wnt/β-catenin reporter line at 19, 25, and 33 days post-fertilization (dpf) (Fig. 1A). Next, the real-time activity of Wnt/β-catenin signaling in oocytes at different developmental stages was assessed. GFP signals with distinct intensity were observed in oocytes with different sizes at different stages, including oogonia, oocytes at stage IA and IB, while no GFP signals were observed in the somatic cells of the gonads (Fig. 1B). We next quantified GFP signal intensity in oocytes of different sizes and calculated the correlation between oocyte diameter and total GFP fluorescence intensity per oocyte. To our surprise, Wnt/β-catenin signaling activity sharply rises in oocytes with size from 10 to 45 μm (corresponding to stage IA, >7 μm to middle of stage IB, 20–140 μm, of primary growth stage) and then rapidly declines (Fig. 1C). To further validate these observations, we assessed *gfp* mRNA levels at corresponding developmental stages using fluorescence in situ hybridization (FISH), followed by quantification of *gfp* signal intensity in oocytes of different sizes. The *gfp* mRNA level sharply increases in oocytes with diameters ranging from 10 to 35 μm and then rapidly declines (Fig. EV1A,B). The slight discrepancy in the oocyte size range between GFP mRNA and protein expression is likely attributable to the greater stability of the GFP protein. Taken together, these results suggest that Wnt/β-catenin signaling activation undergoes rapid up-and-down fluctuations in developing oocytes during the early primary growth stage and likely plays important roles in oocytes ranging in size from 10 to 45 μm.

### The expression of *stn1* is highly enriched in developing oocytes; loss of Stn1 in zebrafish leads to a male-like phenotype associated with infertility

We noted that *wnt4*, a Wnt ligand, is specifically expressed in the somatic cells surrounding oocytes measuring 20 to 50 μm in zebrafish gonads at 20 to 25 dpf (Appendix Fig. S1A) (Kossack et al, 2019). In addition to *wnt4*, *wnt9b* is also expressed in follicle cells, and *wnt8a* in oocytes at 40 dpf, based on a single-cell RNA sequencing (scRNA-seq) dataset derived from zebrafish ovaries at this developmental stage (Liu et al, 2022, data ref: Liu et al, 2022). The cells in this scRNA-seq dataset encompass all known somatic cell types as well as germ cells across developmental stages, ranging from germline stem cells to early oocytes with diameters less than 40 μm (Liu et al, 2022, data ref: Liu et al, 2022). Thus, these Wnt ligands likely function as secreted signals to activate Wnt/β-catenin signaling in oocytes at this stage. Since the germ cell-intrinsic response of Wnt/β-catenin signaling sharply increases at this stage, we speculate that amplifier(s) of Wnt/β-catenin signaling may be present in oocytes during this phase. To identify the amplifier(s), we analyzed the published interacting partners in the β-catenin interactome to examine which one is also enriched in germ cells by using this scRNA-seq dataset obtained from zebrafish ovaries at 40 dpf (Liu et al, 2022, data ref: Liu et al, 2022). A recently identified potential β-catenin-associated protein, Stn1, has drawn our attention (Ehyai et al, 2018). We found that the expression of *stn1* was highly abundant in germ cells, including germline stem cells, germ cell progenitors, and early oocytes, while rare in various somatic cells (Appendix Fig. S1B–D). To further validate the germ cell-enriched expression pattern of *stn1*, we performed FISH on ovarian cryosections across different developmental stages (Appendix Fig. S1E). Consistent with the scRNA-seq results, *stn1* expression was predominantly observed in germ cells at different developmental stages and largely absent in somatic cells, supporting its preferential expression in the germline. Additionally, we also performed quantitative real-time RT-PCR (qRT–PCR) analysis to assess the transcriptional levels of *stn1* in different tissues of 6-month-old female and male zebrafish. The qRT–PCR results indicate that *stn1* was highly expressed in the ovaries and testes (Appendix Fig. S1F). Collectively, these results suggest that Stn1 may play a role in germ cell development.

To investigate the in vivo functions of Stn1, two *stn1*-null zebrafish lines with a target site on exon 2 were generated using CRISPR/Cas9 technology (Chang et al, 2013; Sun et al, 2019) (Appendix Fig. S2A,B). The *stn1* mutant fish did not exhibit overt phenotypes at 3 months post-fertilization (3 mpf). Interestingly, adult mutant fish at 3 mpf in all lines were phenotypically male-like, with a male-like pigmentation hue and abdomen shape and absent female-specific urogenital papillae (Appendix Fig. S3A,B). When mated to wild-type females, all mutant fish exhibited normal male mating behaviors and induced female egg-laying. However, the laid eggs were unfertilized (Appendix Fig. S3C,D). Indeed, *stn1* mutant fish at 3 mpf had no apparent gonads (Appendix Fig. S3E). To confirm this phenotype, a *Tg(β-actin:stn1-Flag)* transgenic line was generated, and rescue analysis was performed (Appendix Fig. S3F). The *stn1*^*+7/+7*^*; Tg(β-actin:stn1-Flag)* adult fish at 3 mpf included males and females (Appendix Fig. S3G). As expected, when mated with corresponding wild-type females or males, male and female *stn1*^*+7/+7*^*; Tg(β-actin:stn1-Flag)* adult fish produced fertilized eggs that were successfully raised to adulthood to generate offspring (Appendix Fig. S3H). Since *stn1* expression is enriched in germ cells, we further examined whether Stn1 functions in a germ cell-autonomous manner. To this end, a *Tg(piwil1:stn1-Flag)* transgenic line was generated using the germ cell-specific *piwil1* promoter for rescue analysis (Appendix Fig. S3I) (Leu and Draper, 2010). Adult *stn1*^*+7/+7*^*; Tg(piwil1:stn1-Flag)* zebrafish exhibit both female and male, and both are capable of producing fertilized eggs when crossed with wild-type males and females, respectively (Appendix Fig. S3J,K). Thus, we concluded that Stn1 functions in a germ cell-autonomous manner. Taken together, these data suggest that genetic loss of Stn1 leads to deficient gonads, a male-like phenotype associated with infertility.

### Genetic deletion of *stn1* impairs gonad development

**Figure 2:**
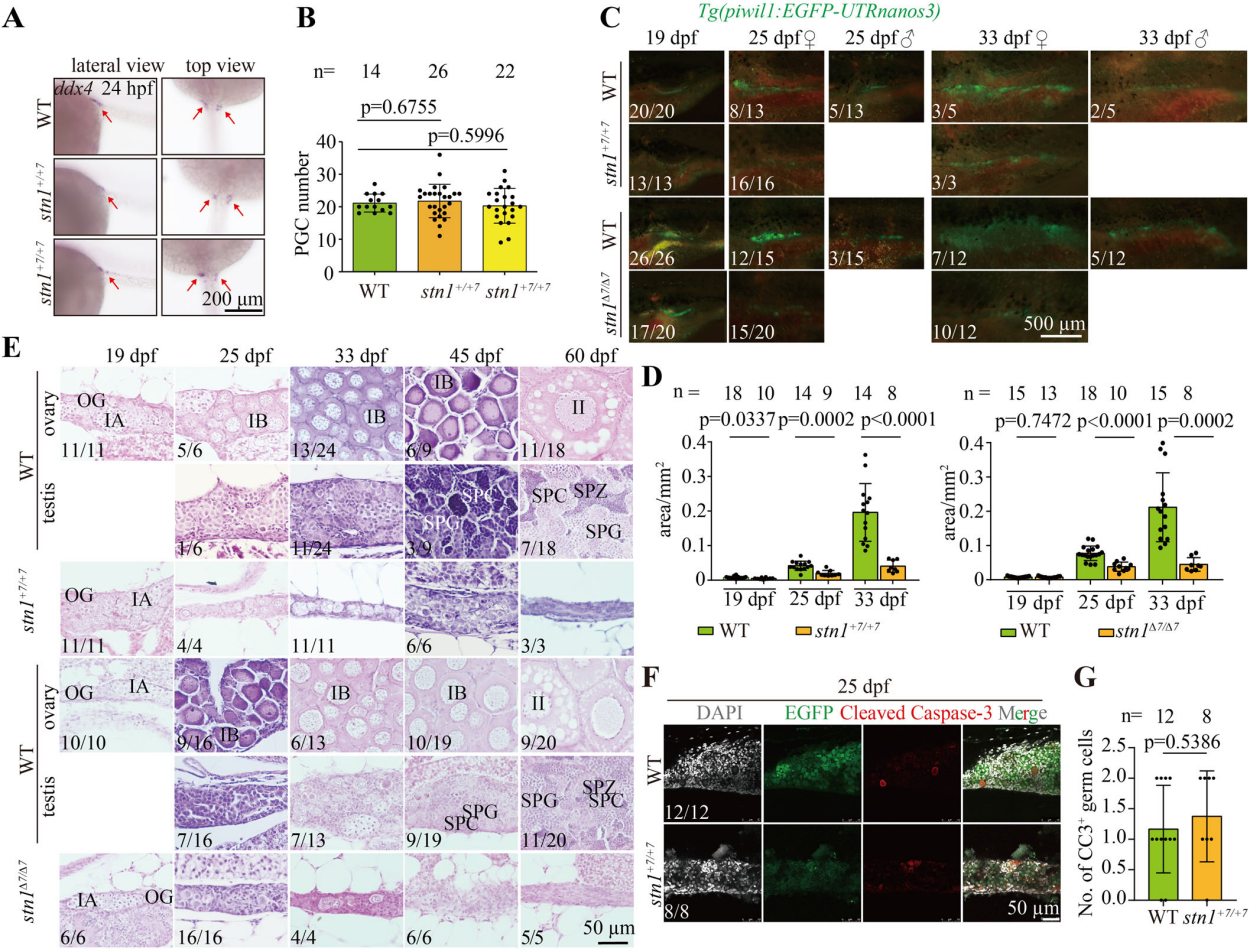
Loss of Stn1 impairs gonad development and leads to germ cell apoptosis. (**A**) Expression of a primordial germ cell marker *ddx4* in wild-type sibling, *stn1*^*+/+7*^, and *stn1*^*+7/+7*^ mutant embryos at 24 hpf. Scale bar: 200 μm. Red arrows indicate the location of *ddx4* expression. (**B**) Quantitative results from images shown in (**A**). Each data point represents an individual embryo, and the total numbers (*n*) are given at the top of columns. Values are represented as means ± SD; Unpaired *t* test, two-tailed. (**C**) Representative images through the body wall showing gonads of sibling and *stn1* mutant fish with a *Tg(piwil1:EGFP-UTRnanos3)* transgenic background at each indicated time point. Scale bar: 500 μm. The proportion of indicated phenotypes is shown in the bottom left corner of each panel. (**D**) Quantitative results from images shown in (**C**). Each dot represents a wild-type sibling or mutant fish, and the total numbers (*n*) are given at the top of columns. Values are represented as means ± SD; Unpaired *t* test, two-tailed. (**E**) Representative histological sections showing gonads of wild-type sibling and *stn1* mutant fish at the indicated time points. The proportion of sections with the indicated phenotypes is shown in the bottom left corner of each panel. OG oogonia, IA stage IA, IB stage IB, II stage II, SPG spermatogonia, SPC spermatocytes; SPZ: spermatozoa. Scale bar: 50 μm. (**F**) Representative gonads of apoptotic cells in the germ cells of wild-type siblings and *stn1* mutants at 25 dpf. Gonads with a *Tg(piwil1:EGFP-UTRnanos3)* transgenic background at 25 dpf immunostained with anti-cleaved caspase 3 antibodies. Scale bar: 50 μm. The proportion of sections with the indicated phenotypes is shown in the bottom left corner of each panel. (**G**) Quantitative results from images shown in (**F**). Each data point represents an individual fish, and the total numbers (*n*) are given at the top of columns. Values are represented as means ± SD; Unpaired *t* test, two-tailed. Source data are available online for this figure.

Given the enriched expression of *stn1* in germ cells and the autonomous requirement of Stn1 for germ cell development, we postulated that loss of Stn1 may impair germ cell development. In zebrafish, after maternally-driven specification, primordial germ cells (PGCs) proliferate and migrate to the genital ridge of embryos at 24 hpf (Weidinger et al, 1999; Yoon and Kawakam, 1999). Ablation of PGCs causes the gonads to adopt the testis fate and lack germ cells (Siegfried and Nusslein-Volhard, 2008; Slanchev, 2005). Therefore, we enumerated the PGCs in wild-type, sibling, and mutant embryos at 24 hpf with an RNA antisense probe of *ddx4* (*vasa*), a marker of PGCs (Braat et al, 1999; Yoon and Kawakam, 1999). Mis-migrated PGCs or reduced PGC abundance were not observed at the genital ridge in mutant embryos (Fig. 2A,B). Thus, genetic loss of Stn1 likely does not affect PGC fate.

**Figure EV2:**
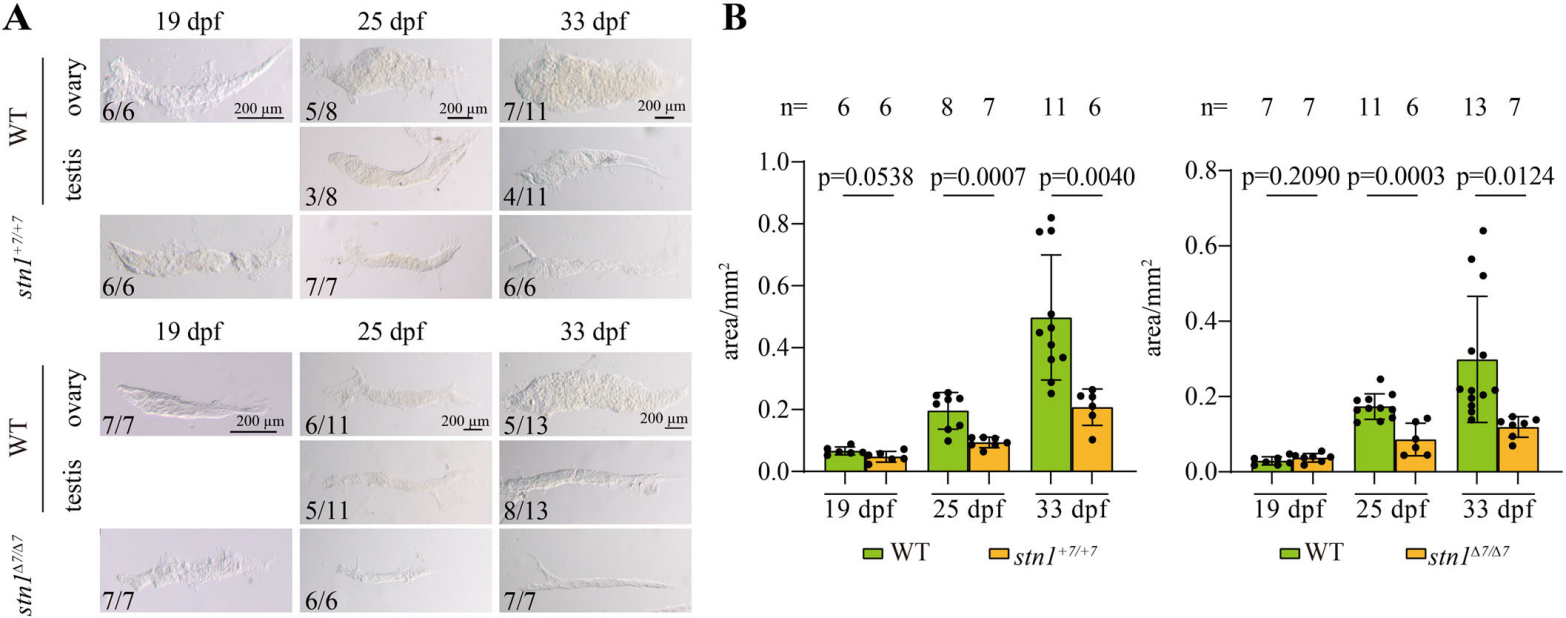
Loss of Stn1 impairs gonad development. (**A**) Representative gonads from sibling and *stn1* mutant zebrafish at the indicated time points. The proportion of gonads with the indicated phenotypes is shown in the bottom left corner of each panel. Scale bar: 200 μm. (**B**) Quantitative results from images shown in (**A**). Each data point represents an individual gonad, and the total numbers (*n*) are given at the top of columns. Values are represented as means ± SD; Unpaired *t* test, two-tailed. Source data are available online for this figure.

The germ cells begin proliferating at around 7–10 dpf in zebrafish (Leerberg et al, 2017). At 19–25 dpf, the undifferentiated gonad contains early-stage oocytes (Dranow et al, 2016; Rodriguez-Mari et al, 2010). Between 25 and 45 dpf, the gonad undergoes differentiation from a juvenile ovary to a mature ovary in females or testes in males due to apoptosis-driven oocyte degeneration (Uchida et al, 2002). To further determine whether and how Stn1 loss affects germ cell development, the developmental process of germ cells was monitored in *stn1* mutant lines with a *Tg(piwil1:EGFP-UTRnanos3)* genetic background, exclusively expressing GFP in the germ cells (Ye et al, 2019). No difference was observed in GFP intensity between sibling and mutant fish at 19 dpf. However, while the signal intensity of siblings markedly increased from 25 to 33 dpf, that of mutants exhibited a little to slight increase (Fig. 2C,D). To further confirm this result, we dissected the gonads of both sibling and mutant fish at 19, 25, and 33 hpf and then measured gonad size. A similar result was obtained (Fig. EV2A,B). Hence, genetic loss of Stn1 likely results in severely underdeveloped gonads starting around 25 dpf.

To further illustrate the function of Stn1 in gonad development, histological analysis was performed on the gonads of sibling and mutant fish from 19 to 60 dpf (Fig. 2E). At 19 dpf, sibling and *stn1* mutant fish exhibited characteristics of the juvenile ovary stage, comprising oogonia and stage IA oocytes. At 25 dpf, in the sibling group, oocyte development progressed to stage IB in some individuals, indicating female differentiation. In contrast, in the *stn1* mutants, oocyte development was arrested at stage IA with rare progression to stage IB or appearance of spermatogonia. From 33 to 60 dpf, the sibling group further differentiated: female oocytes developed from stage IB to stage II. Conversely, in the *stn1* mutants, the oocytes gradually diminished from the gonads. Taken together, these results suggest that genetic deletion of *stn1* leads to germ cell loss.

Considering that markedly fewer germ cells were present in the gonads of mutants than siblings at 25 dpf (Fig. 2E), we sought to determine whether the gradual reduction of oocytes resulted from apoptosis. Hence, an anti-active-caspase-3 antibody was employed to immunostain *stn1* mutant embryos with a *Tg(piwil1:EGFP-UTRnanos3)* genetic background at 25 dpf. Consistent with the phenotype observed in *ddx4* mutants (Hartung et al, 2014), the abundance of active-caspase-3-positive oocytes in the mutants was not increased compared to siblings, despite the apparent loss of oocytes in *stn1* mutants (Fig. 2F,G). This indicates that germ cell loss in *stn1* mutants does not primarily undergo through caspase-mediated apoptosis.

### Loss of Stn1 reduces the expression abundance of germ cell-specific genes

To further confirm the observed gonad deficiencies in *stn1* mutants, bulk RNA-sequencing (RNA-seq) was performed on the trunk region (including gonads) of sibling and mutant fish at 19 and 25 dpf to assess whether the transcript abundance of germ cell-specific genes was reduced (Appendix Fig. S4A). Through differential expression analysis, 27 reduced transcripts commonly shared among the mutants at 19 dpf were identified (Dataset EV1). In contrast, 376 reduced transcripts were specifically identified in the mutants at 25 dpf (Appendix Fig. S4B and Dataset EV1). All reduced transcripts in the mutants at 19 or 25 dpf were assessed for gene ontology (GO) “biological process” terms. The reduced transcripts in the mutants at 19 dpf were not sorted into any “biological process” category. In contrast, the enriched terms associated with the reduced transcripts in the mutants at 25 dpf were related to germ cell development and regulation of reproductive processes (Appendix Fig. S4C). Moreover, we noticed that several of the reduced transcripts at 25 dpf were germ cell-specific, including *ddx4*, *dnd1*, *piwil1/2*, *tdrd1/7a/9*, and *zp2/3* (Appendix Fig. S4D) (D’Orazio et al, 2021; Houston and King, 2000; Lehmann, 2012; Liu et al, 2006; Nguyen-Chi and Morello, 2011; Wolke et al, 2002). The reduction of most of these transcripts was further validated by qRT–PCR (Appendix Fig. S4E). However, it is difficult to determine whether the reduction in gene expression is attributable to decreased expression levels in germ cells or to mutants possessing significantly fewer germ cells. We next examined whether germ cell-specific gene expression was reduced. Since gonads in the mutants contain fewer germ cells, we performed FISH to compare the fluorescence intensity of oocytes between sibling and mutant zebrafish at 25 dpf. Indeed, loss of Stn1 led to markedly reduced expression of germ cell-specific genes in the residual germ cells (Appendix Fig. S4F). These results suggest that Stn1 loss leads to a significant reduction in germ cell numbers and a decrease in germ cell-specific gene expression in mutant gonads beginning at 25 dpf.

### Loss of Stn1 reduces Wnt/β-catenin activity in germ cells of juvenile zebrafish; Stn1 interacts with Tcf/Lef and augments Wnt reporter activity

Stn1 is enriched in developing oocytes, and germ cell-specific expression of Stn1 rescued the germ cell loss caused by Stn1 depletion. In addition, as mentioned earlier, Stn1 is a component of the CST complex and its depletion leads to excessive telomerase activity and a loss of telomere length control (Chen et al, 2012; Zaug et al, 2021). We therefore examined whether excessive telomerase activity contributed to germ cell loss in mutant fish. Previous studies have reported that telomerase-inactivated adult zebrafish exhibit both female and male sexes, and both are capable of producing fertilized eggs and sperm (Anchelin et al, 2013; Henriques et al, 2013). If Stn1 depletion-induced excessive telomerase activity is a major contributor to germ cell loss, inhibition of telomerase activity would be expected to restore germ cell development. Two structurally distinct telomerase inhibitors, BIBR1532 and RHPS4, have been reported to selectively inhibit telomerase activity and shorten telomeres (Chen et al, 2012; Cookson et al, 2005; Damm et al, 2001; Gowan et al, 2001). Hence, both compounds were used to treat mutant fish from 19 to 33 dpf (Appendix Fig. S5A). We observed that the addition of BIBR1532 or RHPS4 did not reverse germ cell loss caused by Stn1 depletion (Appendix Fig. S5B). Therefore, telomerase dysfunction is unlikely to be a major contributor to germ cell loss caused by Stn1 depletion.

**Figure 3:**
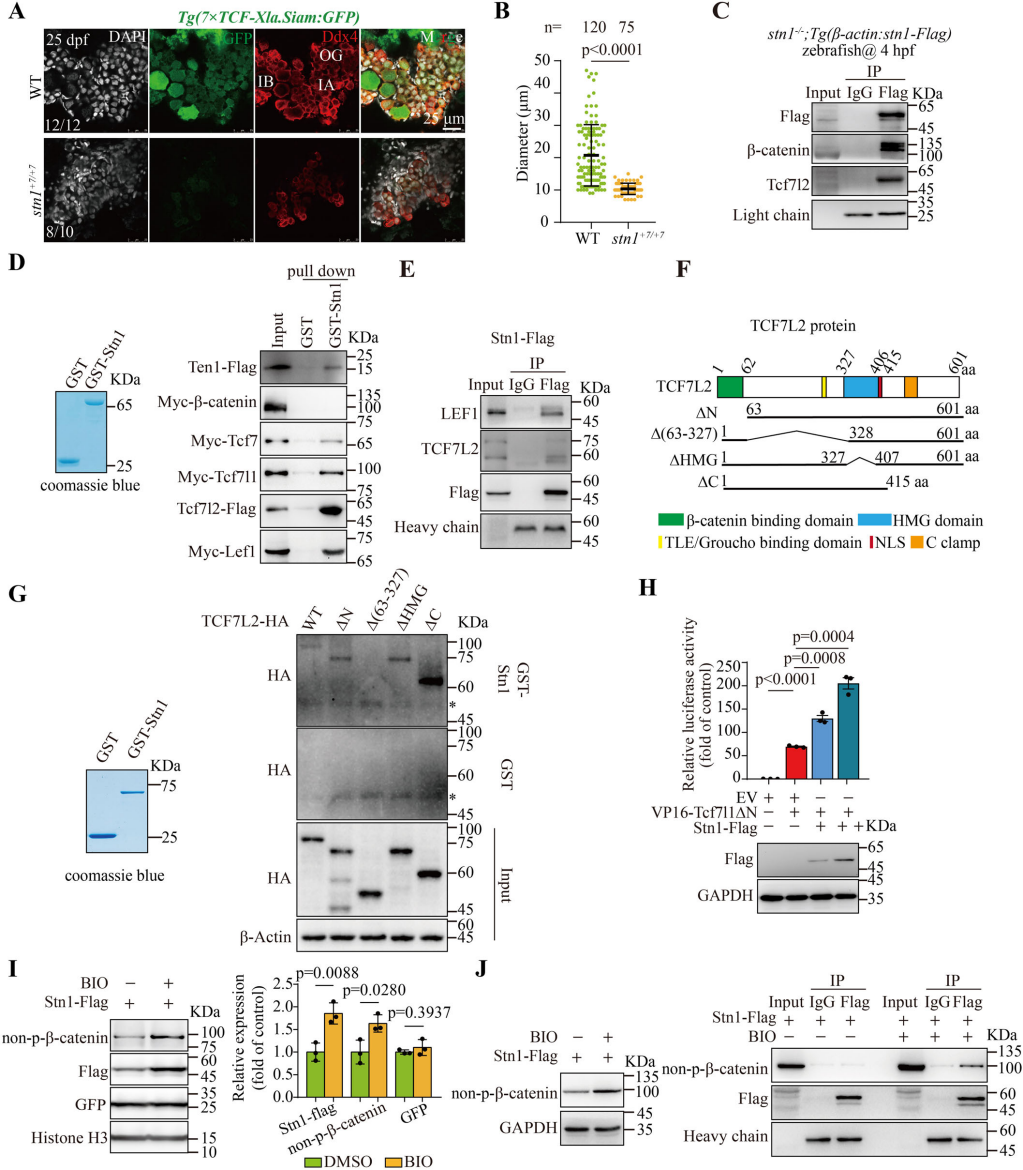
Loss of Stn1 reduces GFP expression in germ cells of juvenile zebrafish with a *Tg(7*×*TCF-Xla.Siam:GFP)* transgenic background; Stn1 interacts with Tcf/Lef and augments Wnt reporter activity. (**A**) Representative confocal images of gonads from siblings and *stn1* mutants at 25 dpf. Gonads of juvenile fish at 25 dpf with a *Tg(7*×*TCF-Xla.Siam:GFP)* transgenic background were immunostained with an anti-Ddx4 antibody. OG oogonia, IA stage IA, IB stage IB. Scale bar: 25 μm. The frequency of juvenile zebrafish with the indicated phenotypes is shown in the bottom left corner of each panel. (**B**) Quantitative results from images shown in (**A**). Each data point represents an individual oocyte, and the total numbers (*n*) are given at the top of columns. The number of wild-type siblings and mutant zebrafish counted was 12 and 10, respectively. Values are represented as means ± SD; Unpaired *t* test, two-tailed. (**C**) Stn1 interacts with β-catenin and Tcf7l2 as indicated by co-immunoprecipitation. Progenies of *stn1*^+7/+7^*;Tg (β-actin:stn1-Flag)* fish incrosses were raised to 4 hpf and subjected to co-immunoprecipitation. (**D**) Stn1 directly binds to Tcf/Lef. GST or GST-Stn1 proteins expressed in bacteria were incubated with cell lysates from HEK293T cells transfected with the indicated plasmids. Ten1-Flag is used as a positive control. (**E**) Stn1 interacts with endogenous LEF1 or TCF7L2 as indicated by co-immunoprecipitation. HEK293T cells with transfected Flag-Stn1 were harvested, and proteins were extracted from cell lysates and then subjected to immunoprecipitation. (**F**, **G**) Detection of the domain in Tcf7l2 responsible for the interaction with Stn1. Asterisk indicates a non-specific band. (**H**) Stn1 augments Wnt activity induced by VP16-Tcf7l1ΔN in vitro. HEK293T cells were transfected with TOPFlash reporter DNA with the plasmid of VP16-Tcf7l1ΔN or VP16-Tcf7l1ΔN plus different doses of the Stn1 plasmid. Values are represented as means ± SD from three independent biological experiments; Unpaired *t* test, two-tailed. (**I**) protein levels of Flag-Stn1 and endogenous non-p-β-catenin in HEK293T cells with or without BIO treatment. HEK293T cells were co-transfected with Flag-tagged Stn1 and GFP. The expression of GFP was used as a control to monitor transfection efficiency. After 24 h, cells were treated with or without BIO for 4 h. Quantification of the protein levels of the indicated proteins was normalized to Histone H3 (right panel). Values are represented as means ± SD from three independent biological experiments; Unpaired *t* test, two-tailed. (**J**) The association of endogenous non-p-β-catenin and Flag-Stn1 in HEK293T cells with or without BIO treatment. HEK293T cells with transfected Flag-Stn1 were treated with or without BIO for 4 h. The cells were harvested, and proteins were extracted from cell lysates and then subjected to immunoprecipitation. Source data are available online for this figure.

**Figure EV3:**
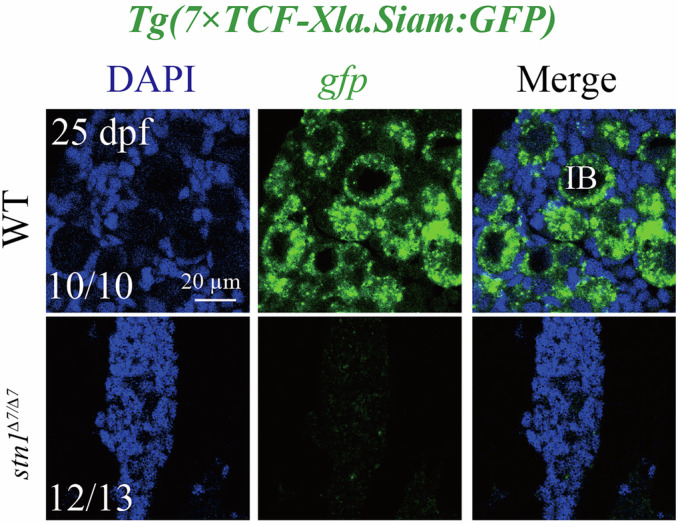
Loss of Stn1 reduces *gfp* mRNA levels in germ cells of juvenile zebrafish with a *Tg(7*×*TCF-Xla.Siam:GFP)* transgenic background. Representative confocal images of gonads from siblings and *stn1* mutants at 25 dpf. Gonads of juvenile fish at 25 dpf with a *Tg(7*×*TCF-Xla.Siam:GFP)* transgenic background were stained with *gfp* mRNA. IB stage IB. Scale bar: 20 μm. The frequency of juvenile zebrafish with the indicated phenotypes is shown in the bottom left corner of each panel. Source data are available online for this figure.

Given that Stn1 was identified as a binding partner of β-catenin, we postulate that it may function as a positive regulator of the Wnt/β-catenin signaling pathway to sustain the development of oocytes. Since the gonads of *stn1* mutant zebrafish exhibit germ cell loss between 19 and 25 dpf, the in vivo activity of Wnt/β-catenin signaling was evaluated in oocytes of *stn1* mutants with the Wnt/β-catenin reporter transgenic background at 25 dpf by measuring GFP protein and mRNA levels. Loss of Stn1 resulted in reduced in vivo Wnt/β-catenin signaling activity in the remaining germ cells, as indicated by markedly decreased GFP protein and mRNA expression (Figs. 3A and EV3). In addition, the size of most oocytes in the gonads of *stn1* mutant zebrafish did not reach 20 μm, i.e, stage IB (Fig. 3A,B).

To further elucidate the underlying molecular mechanism, potential interactions between Stn1 and β-catenin were evaluated through co-immunoprecipitation (Co-IP) assays. However, suitable antibodies against zebrafish Stn1 were not available. The *β-actin* promoter is ubiquitously active throughout development and into adulthood. To minimize the sacrifice of adult female fish while ensuring sufficient cell yield for Co-IP analysis, we used embryos from a *stn1*^*+7/+7*^*;Tg(β-actin:stn1-Flag)* line to examine the interaction between Flag-Stn1 and endogenous β-catenin and/or Tcf/Lef. A recent study showed that β-catenin interacts with Tcf7 or Tcf7l2 to induce expression of maternal β-catenin target genes at 4 hpf (He et al, 2020). Given that Flag-Stn1 is ubiquitously expressed in the *stn1*^*−/−*^*;Tg(β-actin:stn1-Flag)* line, embryos at 4 hpf were selected for Co-IP analysis to investigate whether Flag-Stn1 forms a complex with endogenous β-catenin and/or Tcf7l2. Flag-tagged Stn1 specifically retrieved endogenous β-catenin and Tcf7l2 (Fig. 3C). A subsequent pull-down assay revealed that purified GST-tagged Stn1 bound all family members of Tcf/Lef but not β-catenin, suggesting direct interactions between Stn1 and Tcf/Lef only (Fig. 3D). In addition, Flag-tagged Stn1 also retrieved endogenous LEF1 or TCF7L2 in HEK293T cells (Fig. 3E). Furthermore, a series of pull-down assays of full-length Stn1 was performed with truncated Tcf7l2. Stn1 bound with the Tcf7l2 fragment spanning residues 63–327 (Fig. 3F,G).

We subsequently determined whether Stn1 enhances Wnt/β-catenin transcriptional activity at the Tcf/Lef level. Human HEK293T cells exhibit a functional Wnt/β-catenin signaling system with basal Wnt/β-catenin activity and are commonly regarded as a Wnt-off cell line. This cell line is highly amenable to the induction of Wnt/β-catenin signaling activity through modulation of Wnt components at multiple levels. HEK293T cells were therefore transfected with a low dose of constitutively active Tcf7l1 (VP16-Tcf7l1ΔN), a β-catenin-independent VP16-Tcf7l1 fusion protein lacking the β-catenin-binding domain, to induce Wnt/β-catenin signaling activity. Slightly enhanced Wnt reporter (TOPFlash) activity was observed in the cultured cells (Rong et al, 2017). Overexpression of Stn1 facilitated VP16-Tcf7l1ΔN-induced Wnt activity in a dose-dependent manner (Fig. 3H). Collectively, these data suggest that Stn1 promotes Wnt/β-catenin activity at the Tcf/Lef level.

We next address the underlying mechanism by which Stn1 enhances Wnt/β-catenin transcriptional activity. HEK293T cells with transfected Flag-Stn1 were treated with 6-Bromoindirubin-3’-oxime (BIO), a small molecular compound that activates Wnt/β-catenin signaling by inhibiting GSK3β activity (Sato et al, 2004). Both the levels of non-phospho (active) β-catenin (non-p-β-catenin) and Flag-Stn1 were increased (Fig. 3I), suggesting that activated Wnt/β-catenin signaling promotes the stability of Stn1. Additionally, the amounts of endogenous non-p-β-catenin associated with Flag-Stn1 were increased under activated Wnt activity when assessed by Co-IP assays (Fig. 3J). Therefore, activated Wnt/β-catenin signaling promotes the stability of Stn1, which in turn facilitates the association of Stn1 and β-catenin/Tcf complex to enhance Wnt/β-catenin signaling activity.

### Various germ cell-specific genes are direct targets of Wnt/β-catenin signaling

**Figure 4:**
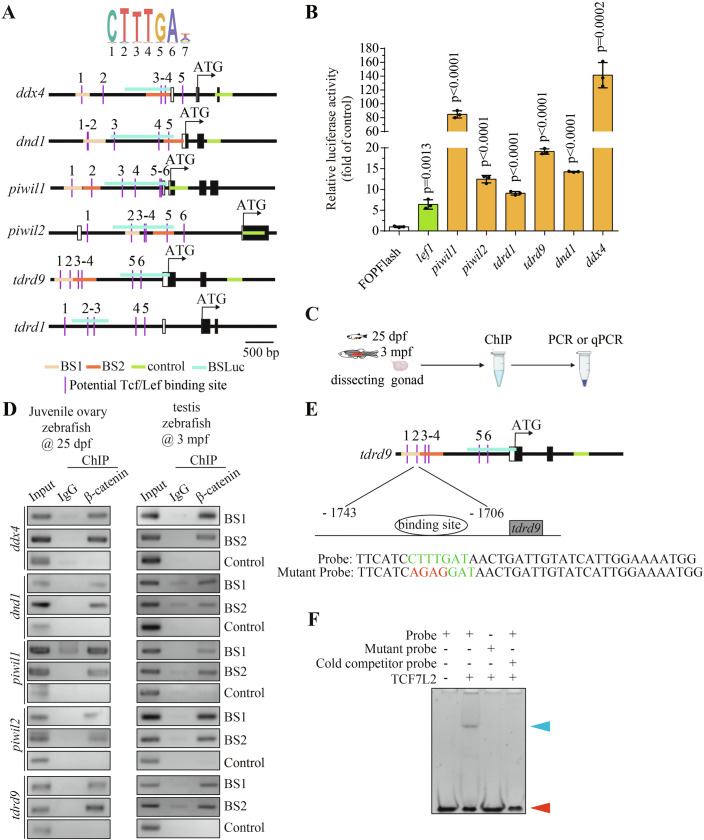
Various germ cell-specific genes are direct target genes of Wnt/β-catenin signaling. (**A**) Promoter regions of various germ cell-specific genes contain high-mobility group DNA-binding domain (HMG DBD) binding sites. Upper panel: HMG DBD binding sites of vertebrate TCF/LEFs. Lower panel: Position of putative HMG DBD DNA binding-sites in the promoter regions of indicated genes. Orange or red boxes: putative HMG DNA-binding sites for ChIP-PCR validation; green boxes: corresponding internal control regions for each indicated gene; cyan boxes: genomic regions used for construction of luciferase reporter vectors; purple vertical lines: potential Tcf/Lef binding sites; black boxes: protein-coding exons; white boxes: untranslated regions (UTRs); black lines: introns. (**B**) Relative Wnt reporter activity with indicated gene promoters induced by VP16-Tcf7l1ΔN in vitro. Each indicated plasmid was co-transfected with VP16-Tcf7l1ΔN plasmid into HEK293T cells, and the luciferase activity was measured at 24 h. Values are represented as means ± SD from three independent biological experiments; Unpaired *t* test, two-tailed. (**C**) Schematic representation of ChIP-PCR or ChIP-qPCR for ovaries of juvenile fish at 25 dpf or testes of adult fish at 3 mpf. (**D**) Endogenous β-catenin is associated with the promoters of the indicated genes in the ovaries or testes, as indicated by the ChIP assay. The binding sites (BS1 and BS2) and control region of the indicated genes are highlighted with orange or red boxes, and green boxes in (**A**), respectively. (**E**) The position and sequence of *tdrd9* with HMG DBD DNA-binding sites. (**F**) Direct binding between TCF7L2 protein and oligonucleotide probes corresponding to the wild-type and mutant binding sites of *tdrd9* examined by EMSA analysis. Red arrowhead: free probe; Blue arrowhead: shifted probe. Source data are available online for this figure.

Considering that knockout of Stn1 in juvenile zebrafish led to loss of oocytes and reduction of both the activity of Wnt/β-catenin signaling in oocytes and the expression abundance of myriad germ cell-specific genes, we speculated that these genes are Wnt-directed target genes. To test this, the promoter regions of some downregulated genes were analyzed using the JASPAR database (https://jaspar.elixir.no/) to determine if they harbored putative Tcf/Lef binding motifs. The promoter regions of *ddx4*, *dnd1*, *piwil1*, *piwil2*, *tdrd9*, and *tdrd1* contain multiple putative Tcf/Lef binding sites (Fig. 4A). Subsequently, a Tcf/Lef binding site-containing partial promoter region of each gene was isolated to generate promoter-driven luciferase reporters. The FopFlash plasmid, which contains mutated TCF/LEF-binding sites upstream of the luciferase reporter gene, was used as a negative control. The promoter of the well-established Wnt target gene *lef1* driven luciferase expression served as a positive control. The expression of these reporters was induced when co-transfected with VP16-Tcf7l1ΔN into HEK293T cells (Fig. 4B). Hence, the promoter regions of these germ cell-specific genes contain Wnt/β-catenin-responsive elements.

To further investigate whether these germ cell-specific genes were regulated by Wnt/β-catenin signaling directly, chromatin immunoprecipitation (ChIP)-PCR analysis was performed with dissected ovaries from juvenile zebrafish at 25 dpf or testes from adults at 3 mpf—tissues where *stn1* is highly expressed (Fig. 4C). ChIP analysis using an anti-β-catenin antibody indicated that the promoter regions contain Wnt-responsive elements. While no regions in the exons or introns of *ddx4*, *dnd1*, *piwil1*, *piwil2*, or *tdrd9* were enriched in the precipitated DNAs (Fig. 4D).

Since β-catenin requires Tcf/Lef to bind DNA, in vitro electrophoretic mobility shift assays (EMSAs) were performed with purified TCF7L2 protein to confirm the binding activity. One potential Tcf binding site within the −1743 to −1705 region of the *tdrd9* promoter was selected as a representative to incubate with recombinant TCF7L2. FAM-labeled wild-type but not mutant probe exhibited a band shift (Fig. 4E,F). Taken together, these data suggest that various germ cell-specific genes are direct target genes of Wnt/β-catenin signaling.

### Wnt/β-catenin signaling induces the transcription of various germ cell-specific genes

To determine whether the transcription of these germ cell-specific genes is regulated by Wnt/β-catenin signaling in vivo, Wnt/β-catenin activity was temporally manipulated through pharmacological inhibition or heat shock-induced activation of Wnt/β-catenin signaling. Wild-type larval zebrafish were treated from 19 to 33 dpf with XAV939 or PNU74654, which are Wnt/β-catenin signaling-specific inhibitors that activate Axin or inhibit the β-catenin/TCF complex, respectively (Appendix Fig. S6A) (Huang et al, 2009; Trosset et al, 2006). Juvenile zebrafish were collected and subjected to body length measurement. Treatment with XAV939 or PNU74654 did not reduce body length in zebrafish (Appendix Fig. S6B,C). Ovarian cryosections were used to assess germ cell loss and to examine mRNA levels of Wnt-induced *gfp* or potential Wnt target genes, as well as to quantify gene expression in oocytes of different sizes for comparison between inhibitor-treated and DMSO control groups. Slight or minimal germ cell loss was observed after XAV939 or PNU74654 treatment. This observation may be attributed to the suboptimal concentration of the inhibitors administered, which was maintained at a lower level to minimize potential toxicity in juvenile zebrafish (Appendix Fig. S6D). Given that the *gfp* mRNA level in the *Tg(7*×*TCF-Xla.Siam:GFP)* transgenic line sharply increases in oocytes with diameters ranging from 10 to 35 μm and then rapidly declines, we performed FISH to assess the transcript levels of Wnt-induced *gfp*, *ddx4*, *dnd1*, *piwil1*, *piwil2*, *tdrd9*, *tdrd1*, and *tdrd7a* in oocytes measuring 10 to 30 μm in diameter. All transcripts were decreased after XAV939 or PNU74654 treatment (Appendix Fig. S6D,E). In addition, the larval offspring of heterozygous *Tg(hsp70l:wnt8a-GFP)* transgenic fish outcrossed with wild-type fish were subjected to heat shock treatments daily from 19 to 33 dpf (Appendix Fig. S7A). Treatment with heat shock did not result in an increase in body length in zebrafish (Appendix Fig. S7B). The transcript levels of *ddx4*, *dnd1*, *piwil1*, *piwil2*, *tdrd9*, *tdrd1*, and *tdrd7a* in oocytes measuring 10–30 μm in diameter from juvenile zebrafish with a heterozygous *Tg(hsp70l:wnt8a-GFP)* transgenic background were upregulated, as revealed by FISH analysis (Appendix Fig. S7C,D). Since the body length of juvenile zebrafish following inhibitor or heat shock treatment remained unchanged, the observed changes in mRNA levels of the examined genes cannot be attributed to alterations in developmental rate. Taken together, these results indicate that the transcription of these germ cell-specific genes is regulated by Wnt/β-catenin activity in vivo.

### Stn1 enhances Wnt/β-catenin signaling by facilitating the binding between the β-catenin/Tcf transcription activation complex and target gene promoters

**Figure 5:**
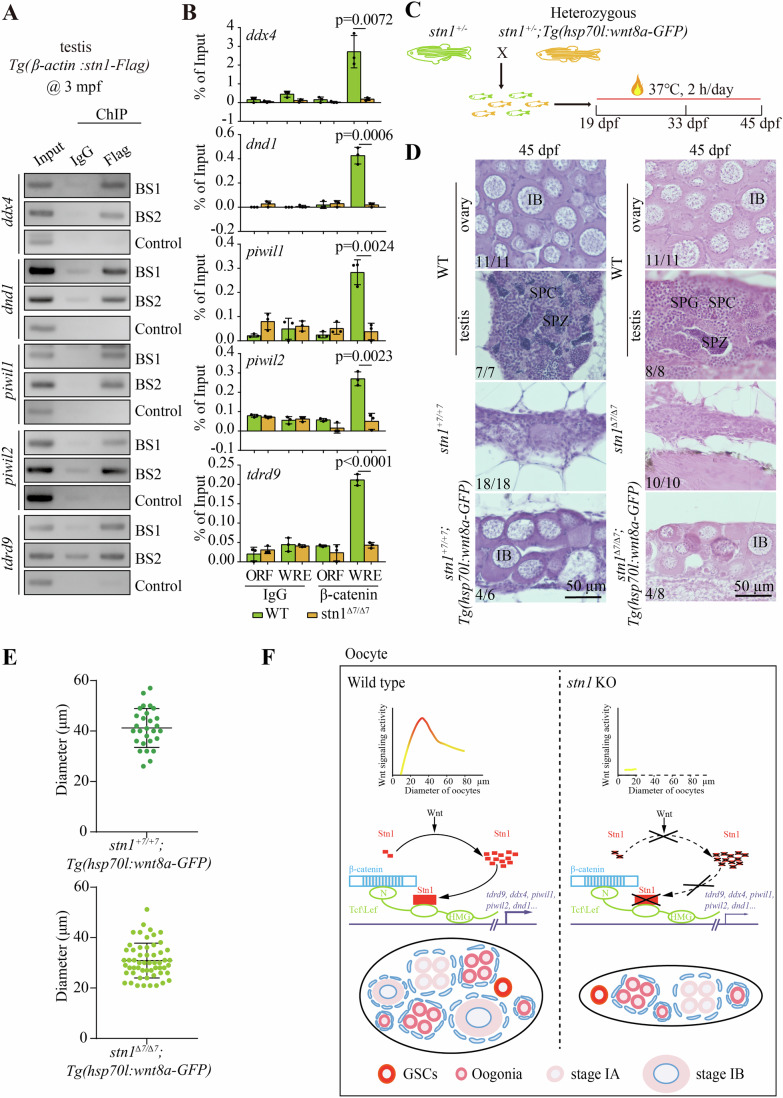
Temporally inducible expression of Wnt8a partially restores the progression of oocytes in *stn1* mutants. (**A**) ChIP assay showing that Stn1 associates with the same promoter region of the indicated genes as β-catenin in the testes of adult *stn1*^*+/−*^*;Tg* (*β-actin:stn1-Flag*) fish at 3 mpf. (**B**) Depletion of Stn1 decreases the association between β-catenin and the promoters of indicated genes, as indicated by ChIP-qPCR analysis. Progeny of *stn1*^*+/−*^ incrosses were raised to 25 dpf, manually dissected gonads, and genotyped. The dissected ovaries were collected. Equal numbers of germ cells with a diameter below 20 µm from siblings and mutants were subjected to ChIP-qPCR analysis. Values are represented as means ± SD from three independent biological experiments; Unpaired *t* test, two-tailed. (**C**–**E**) Heat shock-inducible expression of Wnt8a alleviates the effect caused by Stn1 depletion in gonads. Progeny of *stn1*^*+/−*^ and *stn1*^*+/−*^*; Tg(hsp70l:wnt8a-GFP)* crosses raised to 19 dpf and exposed to a 2-h heat shock (37 °C) daily to 33 or 45 dpf (**C**). Juvenile zebrafish at 45 dpf harvested, genotyped, and gonads dissected for histological analysis. Representative histological sections showing gonads of heat shock-induced sibling and *stn1* mutant fish with or without *Tg(hsp70l:wnt8a-GFP)* genetic background at 45 dpf (**D**). The frequency of the indicated phenotypes is shown in the bottom left corner of each panel. (**E**) Quantitative results from images shown in (**D**). Dot plot showing the size of germ cells at stage IB of homozygous female zebrafish with a *Tg(hsp70l:wnt8a-GFP)* genetic background. Each data point represents an individual oocyte. IB stage IB, SPG spermatogonia, SPC spermatocytes, SPZ spermatozoa. Scale bar: 50 μm. The number of *stn1*^*+7/+7*^*; Tg(hsp70l:wnt8a-GFP)* and *stn1*^*Δ7/Δ7*^*; Tg(hsp70l:wnt8a-GFP)* zebrafish analyzed was 4 and 7, respectively. (**F**) Profile of Wnt/β-catenin activity in the early primary growth stage of oocytes in zebrafish, and proposed model for Stn1 role in amplifying Wnt/β-catenin activity and in gonad development. Source data are available online for this figure.

The molecular mechanism by which Stn1 positively regulates the activity of Wnt/β-catenin signaling in zebrafish oocytes was evaluated. Since Stn1 directly binds to Tcf/Lef and enhances VP16-Tcf7l1ΔN-induced Wnt activity, Stn1 likely occupies the β-catenin/Tcf complex binding promoter regions of these germ cell-specific genes. To test this, the ChIP-PCR assay was performed on the testes from adult fish at 3 mpf with a *stn1*^+7/+7^*;Tg (β-actin:stn1-Flag)* genetic background. The promoter regions of *ddx4*, *dnd1*, *piwil1*, *piwil2*, and *tdrd9* that associate with β-catenin were also enriched in the precipitated DNAs using an anti-Flag antibody (Fig. 5A). Thus, Stn1 likely functions by binding with Tcf/Lef at target promoters and promoting the association of β-catenin/Tcf complex with the target gene promoters. Furthermore, ChIP-qPCR experiments were performed using germ cells isolated from sibling and *stn1* mutant zebrafish at 25 dpf to assess the binding of β-catenin to the promoter regions of germ cell-specific genes. At this stage, ovaries from siblings and mutants contain germ cells of differing numbers and sizes. To address this variability, ovaries from sibling zebrafish were dissected, and germ cells were dissociated from the somatic gonad tissue. Given that the oocyte diameter of most germ cells in the mutants was less than 20 µm, the dissociated germ cells were filtered through a sieve to enrich for those with a diameter below 20 µm. Equal numbers of germ cells from siblings and mutants were then subjected to ChIP-qPCR analysis. Stn1 depletion resulted in a reduced association between β-catenin and the promoter regions of germ cell-specific genes at 25 dpf (Fig. 5B). Collectively, these data indicate that Stn1 associates with β-catenin/Tcf at promoters of germ cell-specific genes and facilitates β-catenin/Tcf binding to the target gene promoters.

### Temporally induced expression of Wnt8a partially restores oocyte development in *stn1* mutants

**Figure EV4:**
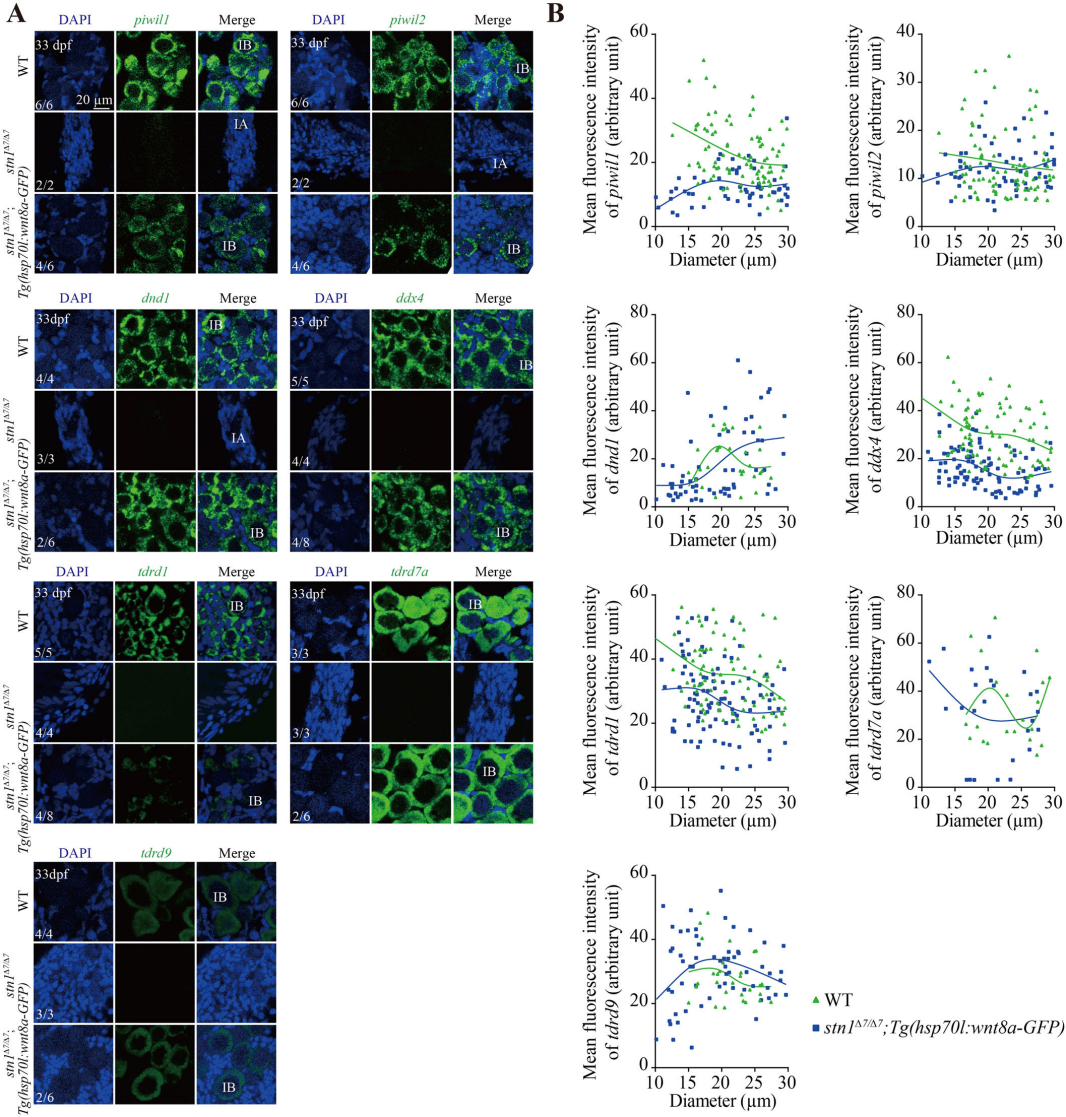
Temporally inducible expression of Wnt8a partially restores the expression of germ cell-specific Wnt target genes in *stn1* mutants. (**A**) Representative confocal images of gonads from wild-type siblings and *stn1* mutants with or without *Tg(hsp70l:wnt8a-GFP)* genetic background at 33 dpf. Gonads from juvenile fish of the indicated genotypes at 33 dpf were stained for the mRNA of each specified gene. IA stage IA, IB stage IB. Scale bar: 20 μm. The frequency of the indicated phenotypes is shown in the bottom left corner of each panel. (**B**) Quantitative results from images shown in (**A**). Each data point represents an individual oocyte. The counts of wild-type siblings, and *stn1*^*Δ7/Δ7*^*; Tg(hsp70l:wnt8a-GFP)* zebrafish were as follows: 4, and 6 for *dnd1* and *tdrd9*; 5, and 8 for *ddx4* and *tdrd1*; 6, and 6 for *piwil1* and *piwil2*; and 3, and 6 for *tdrd7a*, respectively. Source data are available online for this figure.

Given that the transcript levels of these germ cell-specific genes are regulated by Wnt/β-catenin signaling and that Stn1 amplifies the activity of this pathway, temporally inducible Wnt8a was expected to promote the development of oocytes with developmental deficits in *stn1* mutants. To test this hypothesis, *stn1* mutants at 19 dpf with or without *Tg(hsp70l:wnt8a-GFP)* transgenic background were subjected to heat shock treatments daily from 19 to 33 or 45 dpf (Fig. 5C). The gonadal sections of *stn1* mutants at 45 dpf with or without a *Tg(hsp70l:wnt8a-GFP)* genetic background were evaluated. Temporal induction of Wnt/β-catenin signaling significantly promoted the development of oocytes in *stn1* mutants to stage IB, with sizes ranging from 20 to 60 μm (Fig. 5D,E). In addition, the FISH analysis revealed that temporal induction of Wnt/β-catenin signaling activity alleviated the markedly reduced mRNA levels of germ cell-specific genes caused by Stn1 loss (Fig. EV4A,B). Taken together, these data indicate that Stn1 depletion leads to reduced germ cell-intrinsic Wnt/β-catenin signaling activity, resulting in decreased expression of germ cell-specific genes and aberrant progression of germ cell development.

## Discussion

In the present study, we utilized zebrafish oogenesis as a model and showed that Wnt/β-catenin signaling activity sharply rises in the oocytes with size from 10 to 45 μm and rapidly declines later. Various germ cell-specific genes, including *ddx4*, *dnd1*, *piwil1/2*, *tdrd1*, *tdrd7a*, and *tdrd9* were identified as Wnt/β-catenin direct target genes. In addition, we found that *stn1* is highly enriched in germ cells, and its genetic deletion in zebrafish leads to germ cell loss and results in a male-like phenotype associated with infertility. This function of Stn1 is exerted in a germ cell-autonomous manner. Furthermore, we found that activated Wnt/β-catenin signaling enhances the stability of Stn1, which functions as a novel binding partner of Tcf/Lef and facilitates the activity of β-catenin/Tcf complex, thereby amplifying Wnt/β-catenin signaling. Loss of Stn1 reduces Wnt/β-catenin signaling activity in oocytes and impairs oocyte development. Furthermore, temporal induction of Wnt signaling activation partially restores oocyte development in Stn1-depleted conditions to a level consistent with the developmental stage normally characterized by Wnt activation. Thus, Stn1, regulated by Wnt activity, plays a critical role in promoting oocyte development at the early primary growth stage, and interacts with the β-catenin/Tcf complex to modulate its binding to promoters of germ cell-specific genes, thereby influencing Wnt/β-catenin activity in oocytes.

The Wnt/β-catenin signaling pathway participates in germ cell development regulation in vertebrates, such as Wnt/β-catenin signaling promotes spermatogonial stem cell proliferation and differentiation in the adult mouse testes (Chassot et al, 2017; Takase and Nusse, 2016; Tokue et al, 2017). In addition, Wnt/β-catenin signaling controls oocyte differentiation and their entry into meiosis in mice (Chassot et al, 2022; Le Rolle et al, 2021; Vainio et al, 1999). In zebrafish, Wnt/β-catenin signaling regulates sex determination and facilitates the proliferation of germline stem cells to promote ovary regeneration (Cao et al, 2019; Sreenivasan et al, 2014). However, little is known about the real-time dynamic profile of activity of Wnt/β-catenin signaling and its action in oogenesis. In this study, we provided in vivo evidence that the activity of Wnt/β-catenin signaling sharply rises in the oocytes with size from 10 to 45 μm and rapidly declines later. As mentioned earlier, *wnt4* and *wnt9b* are expressed in follicle cells, and *wnt8a* in oocytes at this developmental stage (Liu et al, 2022). Hence, these Wnt ligands likely function as secreted signals to regulate Wnt/β-catenin signaling activity in oocytes during the early primary growth stage. We showed that the Wnt/β-catenin signaling directly controls the expression of various germ cell-specific genes, including *ddx4*, *dnd1*, *piwil1*, *piwil2*, *tdrd1*, *tdrd7a*, and *tdrd9*, at the transcriptional level. Ddx4 is a universal marker of animal germ cells as it is exclusively expressed in the germ cell lineages (Hay, 1988; Lasko and Ashburner, 1988). In zebrafish, Ddx4 is required for germ cell differentiation and maintenance, while its disruption results in sterile adult fish with a male-like phenotype (Hartung et al, 2014). Dnd1 is implicated in germ cell fate maintenance; its depletion leads to complete loss of germ cells in zebrafish (Chu et al, 2023; Weidinger et al, 2003). Piwi proteins are specifically expressed in germ cells and function in germline maintenance (Houwing et al, 2007). The zebrafish genome harbors *piwil1* and *piwil2*, two Piwi genes. Genetic inactivation of Piwil1 leads to progressive germ cell loss (Houwing et al, 2007). Loss of Piwil2 impairs germ cell differentiation and meiosis (Houwing et al, 2008). Tdrd1, Tdrd7a, and Tdrd9 act as molecular scaffolds in the Piwi pathway, which is implicated in germ cell maintenance and transposon silencing (D’Orazio et al, 2021; Huang et al, 2011; Juliano et al, 2011; Rojas-Rios and Simonelig, 2018; Strasser et al, 2008). Thus, activation of Wnt/β-catenin signaling facilitates the transcription of these genes, which impact germ cell development. In zebrafish, oocytes with sizes from 10 to 45 μm spans the early stage of oocyte development, in which the oocyte dramatically increases in size. In addition, intense transcriptional activity was observed during oocyte growth in mice (Clarke, 2012). Activity of Wnt/β-catenin signaling likely acts as a key to maintain and promote the proper progression of oocyte development through the early phase of the primary growth stage. To deeply understand the action of Wnt/β-catenin signaling in intrinsic germ cells, a more thorough investigation into the germ cell-specific target gene profile and the action of Wnt/β-catenin signaling is needed in the future.

Another key discovery of this study is that Stn1 maintains the development of oocytes by amplifying Wnt/β-catenin signaling at the early phase of the primary growth stage. CTC1, STN1, and TEN1 form a trimeric complex termed CST, which is implicated in multiple steps of telomere replication (Chen et al, 2012; Gu et al, 2012; Huang et al, 2012; Miyake et al, 2009; Stewart et al, 2012; Surovtseva et al, 2009; Wang et al, 2012; Wu et al, 2012). CST acts as a DNA polymerase α-primase (Pol-α) cofactor to enhance its enzymatic activity (Casteel et al, 2009; Nakaoka et al, 2012). In addition, CST functions genome-wide to recover stalled replication forks (Bhattacharjee et al, 2017; Chastain et al, 2016; Miyake et al, 2009; Stewart et al, 2012; Zhang et al, 2019). It also facilitates DNA damage repair to maintain genome stability (Greetham et al, 2015; Kratz and Lange, 2018; Mirman et al, 2018; Wang et al, 2014). The CST complex suppresses telomerase activity, and its depletion results in aberrantly elevated telomerase activity and loss of proper telomere length (Chen et al, 2012; Zaug et al, 2021). In humans, dysfunctional mutations in *CTC1* and *STN1* can cause Coats plus syndrome and Dyskeratosis congenita with genomic and telomere defects (Anderson et al, 2012; Han et al, 2020; Keller et al, 2012; Simon et al, 2016). Patients with Dyskeratosis Congenita also carry mutations in genes associated with the telomerase complex, such as *TERT* (telomerase reverse transcriptase) (Kirwan and Dokal, 2009). Genetic loss of *tert* in zebrafish leads to premature aging and degenerative phenotypes. Female and male *tert* mutants produce sperm and eggs, albeit some fertilized embryos exhibit abnormal phenotypes with a higher mortality rate (Anchelin et al, 2013; Henriques et al, 2013). To our knowledge, no complete *Stn1-*knockout animal model has previously been reported. Therefore, the physiological role of Stn1 remained unclear. In this study, we found that Stn1 is required for the maintenance of germ cells in zebrafish. Germ cell loss in *stn1* mutants does not primarily occur through caspase-mediated apoptosis. Stn1 depletion leads to a reduction in the expression of various germ cell-specific genes. Mutants of *ddx4* and *piwil2* exhibit germ cell loss through non-caspase-mediated mechanisms (Hartung et al, 2014; Houwing et al, 2008). In contrast, *piwil1* mutants exhibit germ cell loss through caspase-mediated mechanisms (Houwing et al, 2007). However, the mechanisms underlying germ cell loss through non-caspase-mediated pathways remain poorly understood. Given the complexity of the process, a comprehensive understanding of the molecular mechanisms driving germ cell loss in *stn1* mutants is required to fully elucidate in the future.

Pharmacological inhibition of telomerase activity using small-molecule compounds did not alleviate germ cell loss resulting from Stn1 depletion. Interestingly, temporal induction of Wnt/β-catenin activity partially restored oocyte loss in *stn1* mutants, resulting in a substantial number of oocytes reaching stage IB with diameters of 20–60 μm, yet failing to support further developmental progression. There are several possible explanations for this observation. First, Wnt4 and Wnt9b have been demonstrated to participate in both the canonical and noncanonical Wnt signaling pathways (Carroll et al, 2005; Karner et al, 2009; Park et al, 2007; Tanigawa et al, 2011). Canonical Wnt signaling may be required only at early stages, whereas noncanonical Wnt signaling might be necessary at later stages. Second, Stn1 is a component of the CST complex, which plays an essential role in telomere maintenance and the resolution of stalled replication forks. The function of the CST complex may be important during later stages of development. Stn1 likely has additional roles in promoting later stages of oocyte development. Further studies are required to elucidate these underlying mechanisms. Mechanistically, activated Wnt/β-catenin signaling enhances the stability of Stn1, which in turn associates with and facilitates the activity of the β-catenin/Tcf complex, thereby amplifying Wnt/β-catenin signaling. Our data suggest that Stn1 plays a pivotal role in amplifying Wnt/β-catenin signaling activity in oocytes at the early primary growth stage and maintains the development of oocytes.

In summary, our findings link Wnt/β-catenin signaling, Stn1, and multiple germ cell-specific genes to the maintenance of oocytes in zebrafish. We showed that the activity of Wnt/β-catenin signaling sharply rises in the oocytes with a size from 10 to 45 μm and rapidly declines later. Wnt activity promotes the stability of Stn1, which in turn binds to Tcf/Lef and facilitates the association of the β-catenin/Tcf transcriptional complex with the promoter regions of germ cell-specific Wnt target genes, thereby amplifying Wnt/β-catenin signaling activity. Depletion of Stn1 does not lead to amplification of Wnt signaling in zebrafish germ cells, resulting in reduced Wnt activity and impaired oocyte development, with the majority of oocytes failing to reach a diameter of 20 μm. In particular, our results indicate that Wnt/β-catenin signaling directly regulates various germ cell-specifically expressed essential genes (Fig. 5F). Therefore, our findings not only highlight the importance of Wnt/β-catenin signaling in the regulation of oocyte development but also provide novel insights into the physiological functions of Stn1 in the maintenance of oocytes.

## Methods

**Table:** Reagents and tools table

Reagent/resource	Reference or source	Identifier or catalog number
**Experimental models**		
HEK 293T cells (*H. sapiens*)	ATCC	CRL 3216
*stn1* ^*+7/+7*^	This study	N/A
*stn1* ^*∆7/∆7*^	This study	N/A
*Tg(piwil1:EGFP-UTRnanos3)* ^*ihb327*^	Ye et al (2019)	N/A
*Tg(7×TCF-Xla.Siam:GFP)* ^*ia4*^	Moro et al (2012)	N/A
*Tg(hsp70l:wnt8a-GFP)* ^*w34*^	Stoick-Cooper et al (2007)	N/A
*Tg(β-actin:stn1-Flag)*	This study	N/A
*Tg(piwil1:stn1-Flag)*	This study	N/A
**Recombinant DNA**		
Super 8 ×TOPFlash	Addgene	12456
Super 8 ×FOPFlash	Addgene	12457
Renilla	Addgene	79496
pCS2-VP16-Tcf7l1ΔN	Rong et al (2017)	N/A
pGEX-2T	GE Healthcare	28–9546-53
pGEX-2T- Stn1	This study	N/A
pCS2-Stn1-Flag	This study	N/A
pCS2-Ten1-Flag	This study	N/A
pCS2-Tcf7l2-Flag	This study	N/A
pCS2- Myc-β-catenin	This study	N/A
pCS2- Myc-Tcf7	Wei Wu lab, School of Life Sciences, Tsinghua University	N/A
pCS2- Myc-Tcf7l1	Wei Wu lab, School of Life Sciences, Tsinghua University	N/A
pCS2-Myc-Lef1	Wei Wu lab, School of Life Sciences, Tsinghua University	N/A
pCDNA3.1-TCF7L2-HA	Wang et al, 2025	N/A
pCDNA3.1-TCF7L2ΔN-HA	Wang et al, 2025	N/A
pCDNA3.1-TCF7L2Δ(63-327)-HA	Wang et al, 2025	N/A
pCDNA3.1-TCF7L2ΔHMG-HA	Wang et al, 2025	N/A
pCDNA3.1-TCF7L2ΔC-HA	Wang et al, 2025	N/A
**Antibodies**		
Rabbit anti-TCF7L2	Cell Signaling Technology	2569
Rabbit anti-LEF1	Cell Signaling Technology	2230
Rabbit anti-HA	Cell Signaling Technology	3724
Rabbit anti-non-P-β-catenin (Ser33/37/Thr41)	Cell Signaling Technology	8814
Rabbit anti-cleaved caspase-3(Asp175)	Cell Signaling Technology	9664
Mouse anti-β-catenin (44C6)	Abmart	M24002
Rabbit anti-Ddx4	GeneTex	GTX128306
Mouse anti-GFP	Developmental Studies Hybridoma Bank	DSHB-GFP-1D2
Rabbit anti-GAPDH	BBI Life Sciences	D110016
Rabbit anti-Histone H3.1	Abmart	P30266
Rabbit anti-β-Actin	Absin	ABS132001
Mouse anti-Myc	Santa Cruz Biotechnology	sc-40
Mouse anti-Flag	Sigma	F1804
HRP-labeled Goat Anti-Mouse IgG(H + L)	Beyotime	A0216
HRP-labeled Goat Anti-Rabbit IgG(H + L)	Beyotime	A0208
AF488-labeled Goat Anti- Mouse IgG (H + L)	Beyotime	A0428
Cy3-labeled Goat Anti- Rabbit IgG (H + L)	Beyotime	A0516
Anti-DIG-POD	Roche	11207733910
Anti-fluorescein-POD	Roche	11426346910
**Oligonucleotides and other sequence-based reagents**		
*β-actin*-qF	ACAGGGAAAAGATGACACAG	N/A
*β-actin*-qR	AGAGTCCATCACGATACCAG	N/A
*stn1*-zf-qF	CCGTGTTTTCTTCCTATT	N/A
*stn1*-zf-qR	GCCATTTCTCATCTTTCC	N/A
*piwil1*-qF	TGACATAACAGATGGCAACCA	N/A
*piwil1*-qR	GCCCTCTCTCTGTTCAGGACT	N/A
*piwil2*-qF	TGACAAACAGAGACTGGGTG	N/A
*piwil2*-qR	TCATACCTCTGCAGGTGGTC	N/A
*tdrd1*-qF	AAGGTTCTGAGATAAAGGG	N/A
*tdrd1*-qR	GCGAATGAAGAGCAATAGG	N/A
*tdrd7a*-qF	TCAGAGCAGTAAGCACGGT	N/A
*tdrd7a*-qR	ACACACGCCAGCAAAACAC	N/A
*tdrd9*-qF	CGGTGTGTTACATGAGGTGT	N/A
*tdrd9*-qR	GTTGGAAGTTTCTCGGGATT	N/A
*dnd1*-qF	TGATTCCTCAACCCACCATAA	N/A
*dnd1*-qR	TGGACTTCATATTGCGGAGA	N/A
*ddx4*-qF	GGGCTGCAATGTTCTGTGTG	N/A
*ddx4*-qR	CAGTTTGCGCATTTCTGGCT	N/A
CHIP-*ddx4*-F1 / CHIP-qPCR-*ddx4*-WRE-F	GGAGAAGTTTTATTTCGGCAAGAATTG	N/A
CHIP-*ddx4*-R1 / CHIP-qPCR-*ddx4*-WRE-R	TTAAAGTGACATTTAAAGGCTTAGGGT	N/A
CHIP-*ddx4*-F2	GGAGATCCTAACCATCCATACACGT	N/A
CHIP-*ddx4*-R2	CGTGGGCGGGAAAATCGATGTAACG	N/A
CHIP-*ddx4*-NC-F	ATTTACCCTTTTTACAGAGTCCC	N/A
CHIP-*ddx4*-NC-R	TAAGTCACAGGTTTTCAGCTTTC	N/A
CHIP-*dnd1*-F1	ATACATTTTGTCACCTTGGAATAAG	N/A
CHIP-*dnd1*-R1	AATTCCAAAAGTGTTGCAGCTGTCC	N/A
CHIP-*dnd1*-F2	AGTATCCAGAATGGCGTTCACTCAC	N/A
CHIP-*dnd1*-R2	AGAAACCTCATGACTCCTAGAGACG	N/A
CHIP-*dnd1*-NC-F	TAAAGACGAGCACATGGCATCATTT	N/A
CHIP-*dnd1*-NC-R	ACCTCCAGGTGTCCACGTTTTAATG	N/A
CHIP-*piwil1*-F1	TAACGTTTAGCATGCACGCACCAGC	N/A
CHIP-*piwil1*-R1	CATGCAAAAGCATAATGTTACCTTC	N/A
CHIP-*piwil1*-F2 / CHIP-qPCR-*piwil1*-WRE-F	ATTCAGACAGCACAATCTGCTCAAT	N/A
CHIP-*piwil1*-R2 / CHIP-qPCR-*piwil1*-WRE-R	TTTAGGAGATTTACCGACAGAACAG	N/A
CHIP-*piwil1*-NC-F	GACAGGACGAGCAAGAGCAAGAT	N/A
CHIP-*piwil1*-NC-R	ATGGACTTGTGGGGGAAACCAGAGC	N/A
CHIP-*piwil2*-F1 / CHIP-qPCR-*piwil2*-WRE-F	ACATGTTACTCTGCACATCACTGT	N/A
CHIP-*piwil2*-R1 / CHIP-qPCR-*piwil2*-WRE-R	AATGTCATCTGTAGCTTCCCCAGAT	N/A
CHIP-*piwil2*-F2	CAGAATGATGAGGCACTTAAATTCT	N/A
CHIP-*piwil2*-R2	AAATGGGGTGTACTCAATTATGCTG	N/A
CHIP-*piwil2*-NC-F	ACCAACCTTTCCCAGTCCCCCAG	N/A
CHIP-*piwil2*-NC-R	AGCCCCTCTCCCTATCCCAACAGTG	N/A
CHIP-*tdrd9*-F1	GGAGGATCTTGCTTTGCATTCTTTA	N/A
CHIP-*tdrd9*-R1	CATGGAATGCCTTTTGGGCAGGGAT	N/A
CHIP-*tdrd9*-F2	ACCAGAGGTGAAGAAAAGTTTTTTC	N/A
CHIP-*tdrd9*-R2	TTGTGTGAGCAGTTGCGTACAACAT	N/A
CHIP-*tdrd9*-NC-F	CAGTTTTTGGACTTGCTGTATCACA	N/A
CHIP-*tdrd9*-NC-R	TATTTTACCATTTTTCAGACCCCGC	N/A
CHIP-qPCR-*dnd1*-WRE-F	ATCAGTCTAAAACGCCTTGT	N/A
CHIP-qPCR-*dnd1*-WRE-R	AGAAACCTCATGACTCCTAGAGACG	N/A
CHIP-qPCR-*dnd1*-ORF-F	CTGAGCAGAAACAGAAAGTC	N/A
CHIP-qPCR-*dnd1*-ORF-R	CAAAGCACAAAAGAGGCATG	N/A
CHIP-qPCR-*tdrd9*-WRE-F	GATTTAGGCTACTCATAAACACCCT	N/A
CHIP-qPCR-*tdrd9*-WRE-R	GTCGTCATTCCTCACCCCTCTTAAC	N/A
CHIP-qPCR-*tdrd9*-ORF-F	CACAGTTTGCGAACATCAGG	N/A
CHIP-qPCR-*tdrd9*-ORF-R	CACATTTCTTTGCGGCTTTAT	N/A
CHIP-qPCR-*piwil1-*ORF-F	CAGTTGGCATTTCTGTGTGG	N/A
CHIP-qPCR-*piwil1*-ORF-R	GTCTATGGACTTGTGGGGGA	N/A
CHIP-qPCR-*piwil2-*ORF-F	GCTAGAGGTGTGAGGTTACC	N/A
CHIP-qPCR-*piwil2*-ORF-R	GCTTTTTGAATGTCCAGTGT	N/A
CHIP-qPCR-*ddx4*-ORF-F	ATTTACCCTTTTTACAGAGT	N/A
CHIP-qPCR-*ddx4*-ORF-R	CGAAAACTTGTATAATTAGT	N/A
TCF7L2 binding probe labeled with FAM-F	TTCATCCTTTGATAACTGATTGTATCATTGGAAAATGG	N/A
TCF7L2 binding probe labeled with FAM-R	CCATTTTCCAATGATACAATCAGTTATCAAAGGATGAA	N/A
TCF7L2 binding mutant probe labeled with FAM-F	TTCATCAGAGGATAACTGATTGTATCATTGGAAAATGG	N/A
TCF7L2 binding mutant probe labeled with FAM-R	CCATTTTCCAATGATACAATCAGTTATCCTCTGATGAA	N/A
*stn1*-probe-F	ACTCCGTGGGCTTTTCAAACTCAT	N/A
*stn1*-probe-R(T7)	TGTAATACGACTCACTATAGGGACTTGTGTGGCAAATGATGTCCCG	N/A
*ddx4*-probe-F	GATCTCAACCACAAGCATCCAT	N/A
*ddx4*-probe-R(T7)	TGTAATACGACTCACTATAGGGCGTCACCAGTATCCGTCTTTATT	N/A
*tdrd9*-probe-F	AGCAGCAGTCTGAGAGAGCTCCCAT	N/A
*tdrd9*-probe-R(T7)	TGTAATACGACTCACTATAGGGCAGACAACCACTGCATCAAC	N/A
*dnd1*-probe-F	ACAGATGGTCGGAGACATGGAT	N/A
*dnd1*-probe-R(T7)	TGTAATACGACTCACTATAGGGTTGAGACTCGGCACAAGGTT	N/A
*piwil1*-probe-F	GTCAAGGTGGTTCTCCAAGTG	N/A
*piwil1*-probe-R(T7)	TGTAATACGACTCACTATAGGGCATTACAACGATTTATCTTTC	N/A
*piwil2*-probe-F	GATGGTGATTGGAGTGGATGT	N/A
*piwil2*-probe-R(T7)	TGTAATACGACTCACTATAGGGAGGGTTTTGTTGAGATGCTGAAT	N/A
*tdrd1*-probe-F	GCTGCCTGCCAATGTCAATGAAGAG	N/A
*tdrd1*-probe-R(T7)	TGTAATACGACTCACTATAGGGCATAAAAGCACAAAGGGTATAC	N/A
*tdrd7a*-probe-F	GATGAGTGACGTGGAGTTGGTTA	N/A
*tdrd7a*-probe-R(T7)	TGTAATACGACTCACTATAGGGTGGCTGTATTTATTGAGCAGCTG	N/A
**Chemicals, enzymes, and other reagents**		
TCF7L2(1-456) protein	CUSABIO	CSB-EP889079HU
CHIP assay kit	Millipore	17-295
EZ-PCR Mycoplasma Detection Kit	Biological Industries	20-700-20
TSA plus Fluorescein detection kit	PerkinElmer	NEL741001KT
TSA plus Cyanine 3 (TSA Cy3) detection kit	PerkinElmer	NEL744001KT
Fluorescein RNA Labeling Mix	Roche	11685619910
DIG RNA Labeling Mix	Roche	11277073910
Proteinase K	Roche	03115879001
5×All-In-One RT MasterMix Kit	Applied Biological Materials Inc	G592
NovoStart Universal Fast SYBR qPCR SuperMix	novoprotein	E401
Premix Taq	Takara	RR901A
RNAiso Plus	Takara	9109
Dulbecco’s modified Eagle’s medium	Hyclone	SH30243.01
penicillin and streptomycin	Hyclone	SV30010
Polyethylenimine	Polysciences Inc.	23966-2
Fetal calf serum	ExCell	FSP500
Dual-Luciferase Reporter Assay System	Promega	E1960
Glutathione Sepharose 4B beads	GE Healthcare	71024800-GE
XAV939	MedChemExpress	HY-15147
PNU-74654	MedChemExpress	HY-101130
BIO (GSK 3 Inhibitor IX)	MedChemExpress	HY-10580
BIBR1532	TargetMol	T2380
RHPS4	TargetMol	T6967
Protein A/G Plus-agarose	Santa Cruz Biotechnology	sc-2003
DAPI (4’,6-Diamidino-2-Phenylindole, Dihydrochloride)	Beyotime	C1002
BeyoBlue™ Plus Coomassie Blue SuperFast Staining Solution	Beyotime	P0003S
**Software**		
GraphPad Prism	GraphPad Software	Version 9
ImageJ	NIH	Version 1.54p
**Other**		
ChemiDoc MP Imaging System	Bio-Rad	
Trans-Blot SD Cell	Bio-Rad	
QuantStudio 3 Real-Time PCR System	Applied Biosystems	
Leica M205 microscope	Leica	
Leica RM2016 microtome	Leica	
Leica SP8 confocal microscope	Leica	
VCX 130 Sonicator	Sonics	

### Experimental animals

Wild-type zebrafish (Tübingen strain) were used. The *stn1* mutant zebrafish was constructed using the CRISPR/Cas9 system, with the target site (5′-GACTCACCCCATCTTCCAGG-3′) in the second exon. The *Tg(β-actin:stn1-Flag)* and *Tg(piwil1:stn1-Flag)* transgenic lines were established using the Multisite Gateway system (Kwan et al, 2007). The *stn1* mutants with *Tg(β-actin:stn1-Flag)*, *Tg(piwil1:stn1-Flag)*, *Tg(piwil1:EGFP-UTRnanos3)* ^*ihb327*^, *Tg(7×TCF-Xla.Siam:GFP)*^*ia4*^, or *Tg(hsp70l:wnt8a-GFP)*^*w34*^ genetic backgrounds were acquired through natural breeding. Zebrafish were kept at 28.5 °C on a 14-h light/10-h dark cycle. All experimental protocols were approved by and carried out in accordance with the Ethical Committee of Experimental Animal Care at Ocean University of China.

### Fertility assessment

When assessing the fertility of *stn1* mutant fish against wild-type female fish, to exclude individual differences among female fish, the same wild-type female was sequentially mated with a wild-type male, followed by a mutant male-like fish within 30 min of the initial spawning event. At the next mating time, the mutant male-like and wild-type male fish were switched to mate with the same wild-type female fish.

To assess the fertility of wild-type, *stn1* mutant, and *stn1* mutant with a *Tg(actin:stn1-Flag)* or a *Tg(piwil1:stn1-Flag)* transgenic background fish, females and males of different genotypes were crossed with a batch of wild-type counterparts. The fertilization rate of each pair of mated fish was counted when the offspring developed to the shield stage.

### RNA extraction and RT-PCR analysis

Total RNA was isolated from three manually dissected trunk regions or gonads at the indicated stages using the RNAiso Plus Kit; 2 µg of RNA was reverse transcribed into cDNA using the 5×All-In-One RT MasterMix Kit. RT-PCR was performed using Taq DNA polymerase. Quantitative real-time RT-PCR (qRT–PCR) was carried out using a QuantStudio 3 Real-Time PCR System (Applied Biosystems, Wakefield, RI, USA). The mRNA levels of the genes of interest were calculated using the 2^-ΔΔCt^ method and normalized to *β-actin* levels (Livak and Schmittgen, 2001).

### RNA sequencing (RNA-seq) and single-cell RNA sequencing (scRNA-seq)

For RNA-seq, trunk regions were dissected from wild-type or *stn1* mutant zebrafish at 19 or 25 dpf. Each sample comprised tissue from two to three individuals and two biological replicates for each group. Total RNA was isolated using TRIzol according to standard protocols. The 2 × 100 bp paired-end sequencing was performed on a BGISEQ-500 platform by BGI following the vendor’s recommended protocol (BGI, Qingdao, China). For data processing, raw reads were filtered using SOAPnuke (v1.5.2) to obtain clean reads, which were aligned to the reference genome using HISAT2 (v2.0.4). The gene and transcript expression levels were calculated using RSEM (v1.2.12). The heatmap was generated using TBtools (Chen et al, 2023) based on FPKM values. Differentially expressed genes (DEGs) were identified using the PoissonDis algorithm and visualized on a volcano map plotted by https://www.bioinformatics.com.cn (Tang et al, 2023), applying a filter of adjusted *P* value (*P*adj) <0.05 and |log_2_foldchange | >1. Subsequently, pathway enrichment analysis of these DEGs was conducted using the Metascape platform (Zhou et al, 2019).

For scRNA-seq, processed scRNA-seq datasets from ovaries of juvenile zebrafish at 40 dpf, which encompassed various ovarian cell types (Liu et al, 2022, data ref: Liu et al, 2022), were used and accessed through the Single Cell Portal (https://singlecell.broadinstitute.org/single_cell/study/SCP928/40dpf-ovary-all-cells).

### Cell culture and luciferase assays

The HEK293T cells were cultured in DMEM with 10% FBS and 1% PS (penicillin and streptomycin) in a humid incubator with 5% CO_2_ at 37 °C. To confirm their identity and ensure the absence of mycoplasma contamination, the cell lines were subjected to short tandem repeat profiling by ShCellBank and periodic testing every 3 months using the EZ-PCR Mycoplasma Detection Kit. HEK293T cells were seeded in 24-well plates to reach 70–80% confluence. Transfections were performed using Polyethylenimine.

To assess the effect of Stn1 on Wnt/β-catenin signaling, the pCS2-VP16-Tcf7l1ΔN plasmid and pcDNA3.1 vector or different doses of the pcDNA3.1-Stn1-Flag plasmid were co-transfected into cells, along with TOPFlash and *Renilla* reporter plasmids in each group.

In assays validating promoter interaction with Tcf/Lef, cells were transfected with a *Renilla* plasmid, accompanied by the FOPFlash plasmid or gene promoter-linked luciferase constructs, in the presence of pCS2-VP16-Tcf7l1ΔN. Uniform DNA quantities across wells were maintained using an empty vector supplement. Luciferase activities were measured using a Dual-Luciferase Reporter Assay System, with the Firefly luciferase output normalized against *Renilla* luciferase activity for precise assessment of transcriptional responses and the values were further normalized to FOPFlash control.

### Co-immunoprecipitation (Co-IP), GST-pull down, and western blotting

Progenies of *stn1*^*−/−*^*;Tg(β-actin:stn1-Flag)* intercrosses were reared to 4 hpf for Co-IP analysis. After removal of chorions and yolks, embryos were lysed in IP lysis buffer (Tris-HCl [pH 7.5], 50 mM; NaCl, 150 mM; EDTA, 1 mM; 10% glycerol and 1% Triton X-100) containing protease inhibitors. The HEK293T cells were transfected with Stn1-Flag. After 24 h, the cells were treated with DMSO or BIO (2 μM) for 4 h, followed by harvesting with IP lysis buffer containing protease inhibitors. Immunoprecipitation was then performed using an anti-Flag antibody, and enrichment was carried out with Protein A/G PLUS-Agarose beads. Immuno-complexes were washed 3 times and then eluted from beads by boiling in a loading buffer and analyzed through western blotting.

For the GST-pull-down, pGEX-2T and pGEST-2T-Stn1 plasmids were individually transformed into *E. coli* BL21 and induced with Isopropyl β-D-thiogalactoside (IPTG) to express GST and GST-Stn1, respectively (Wang et al, 2025). GST-tagged proteins were enriched using GSH Sepharose 4B Beads and analyzed by Coomassie Brilliant Blue staining. Cell lysates were incubated with beads coupled with GST or GST-Stn1. Proteins bound to the beads were washed two times and then eluted by boiling in loading buffer and analyzed through western blotting.

For western blot analysis, proteins were separated by SDS-PAGE and transferred onto a PVDF membrane, and incubated with primary and secondary antibodies, followed by chemiluminescence detection.

### Pharmacological and heat shock treatment

The small-molecule compounds XAV939 and PNU-74654 were applied to inhibit Wnt/β-catenin signaling in vivo. Wild-type zebrafish were randomly assigned to treatment with 5 μM of each inhibitor from 19 to 33 dpf. The small-molecule compounds BIBR1532 and RHPS4 were applied to inhibit telomerase activity and shorten telomeres. The *stn1*^*+/−*^ intercrosses were raised to 19 dpf and randomly assigned to treatment with DMSO, 1 μM BIBR1532, or 15 μM RHPS4 from 19 to 33 dpf. The inhibitor was refreshed every 2 days. Juvenile zebrafish were harvested at 33 dpf and genotyped for further analysis.

Blind experiments were conducted to evaluate the effects of Wnt/β-catenin signaling pathway overactivation on germ cell-specific genes and to determine whether it could restore the oocyte development defect caused by *stn1* knockout. To this end, the progenies of wild-type crossed with heterozygous *Tg(hsp70l:wnt8a-GFP)* or *stn1*^*+/−*^; *Tg(hsp70l:wnt8a-GFP)* intercrosses were raised to 19 dpf and exposed to a 2 h heat shock treatment at 37 °C daily until indicated stages. The heat shock-treated zebrafish were harvested and subjected to genotyping analysis before further analyses.

### Whole-mount in situ hybridization (WISH)

Whole-mount in situ hybridization using a DIG-labeled RNA riboprobe was carried out as previously described (Bai et al, 2014). A blinded assay was performed to assess the effect of *stn1* knockout on *ddx4* expression at 24 hpf. Mixed embryos at 24 hpf were fixed overnight at 4 °C in 4% paraformaldehyde (PFA). Following permeabilization, rehydration, proteinase K treatment, re-fixation, and pre-hybridization, embryos were hybridized with a digoxigenin (DIG)-UTP-labeled *ddx4* antisense RNA probe (Yoon and Kawakam, 1999). Images were captured using a Leica M205 microscope. Following image acquisition, genomic DNA was extracted from individual embryos for genotyping.

### Fluorescence in situ hybridization (FISH)

Following anesthesia, zebrafish were fixed in 4% PFA and stored at 4 °C for 48 h. The head and tail regions were subsequently removed, and the tail was preserved for genotyping analysis. The trunk segment containing the gonads was processed for paraffin embedding. Serial sections of 5 μm thickness were obtained using a Leica RM2016 microtome and collected for subsequent histological analysis.

FISH on tissue sections was performed according to established protocols (Chen and Ge, 2012). Sections were deparaffinized and rehydrated, followed by treatment with proteinase K (4 µg/mL) at 37 °C for 15 min. Hybridization was carried out overnight at 65 °C using DIG- or fluorescein-labeled RNA riboprobes. After hybridization, sections were incubated with anti-DIG-POD or anti-fluorescein-POD antibodies, and signal amplification was performed using a TSA Plus Fluorescein or Cyanine 3 detection kit. Nuclei were counterstained with DAPI, and images were acquired using a Leica SP8 confocal microscope. Cell diameter and mean fluorescence intensity were quantified using ImageJ software.

### Immunofluorescence staining

Immunofluorescence analysis was performed according to a previously reported protocol with minor modifications (Yang and Xu, 2012). Briefly, zebrafish gonads were dissected and fixed in 4% PFA overnight at 4 °C. After washing with PBST, samples were blocked in 10% FBS for 2 h, incubated with primary antibodies overnight at 4 °C, washed with PBST, and incubated with an appropriate secondary antibody for 2 h at room temperature. DAPI staining was performed, and images were captured using a Leica SP8 confocal microscope. Cell diameter and total fluorescence intensity were quantified using ImageJ software.

### Histological examination

Histological analysis was largely performed following a previously reported protocol (Copper et al, 2018). Briefly, zebrafish were fixed with 4% PFA, adjusting the fixation temperature and duration based on the sample sizes. When zebrafish were more than 21 days old, they were fixed and decalcified with EDTA. The specimens were then dehydrated in an alcohol series, clarified with xylene, embedded in paraffin, and sectioned to a thickness of 8 μm. The sections were subjected to hematoxylin and eosin (HE) staining, mounted, and imaged using a Nikon E80i microscope.

### Chromatin immunoprecipitation (ChIP)

The ChIP assay was carried out using the CHIP assay kit according to the manufacturer’s protocol (Rong et al, 2017). The ovaries of sibling and mutant zebrafish at 25 dpf were dissected, and germ cells were dissociated from the gonad using a modified protocol (Blokhina et al, 2019). Dissociated cells from sibling zebrafish were filtered through a sieve to enrich for germ cells with a diameter less than 20 µm, according to the previously described method (Elkouby and Mullins, 2017). Equal numbers of rough size-matched germ cells from siblings and mutants were collected and fixed with formaldehyde. In addition, ovaries from wild-type zebrafish at 25 dpf and testes at 3 mpf were similarly fixed with formaldehyde. After washing with PBST, the tissues were lysed in lysis buffer Ⅰ [Tris-HCl (pH 8.0), 10 mM; NaCl, 10 mM; NP-40, 0.5%] and lysis buffer ⅠⅠ [Tris-HCl (pH 8.0), 50 mM; EDTA, 10 mM; SDS, 1%]. Subsequently, the IP buffer [Tris-HCl (pH 8.0), 20 mM; EDTA, 2 mM; NaCl, 150 mM; SDS, 0.01%] was added before subjecting the tissues to ultrasound (Vibra-Cell^TM^ Sonicator; 10 s on, 20 s pulses, total time is 10 min; 40% amplitude). ChIP samples were kept in ice water. The obtained supernatant was incubated with a β-catenin antibody, a Flag antibody, or IgG protein; Protein A/G PLUS agarose beads were collected in wash buffer I/II/III [wash buffer I: Tris-HCl (pH 8.0), 20 mM; EDTA, 2 mM; NaCl, 150 mM; Triton X-100, 1%; SDS, 0.1%. wash buffer II: Tris-HCl (pH 8.0), 20 mM; EDTA, 2 mM; NaCl, 500 mM; Triton X-100, 1%; SDS, 0.1%. wash buffer III: Tris-HCl (pH 8.0), 10 mM; EDTA, 1 mM; LiCl, 250 mM; NP-40, 1%; SDS, 1%] and TE buffer [Tris-HCl (pH 8.0), 10 mM; EDTA, 1 mM]. Finally, the supernatant was obtained following elution buffer [Tris-HCl (pH 8.0), 25 mM; EDTA, 10 mM; SDS, 0.5%] treatment at 65 °C. DNA fragments were obtained by decross-linking for 6 h at 65 °C. After removing RNA and protein, DNA was extracted and purified by phenol-chloroform. Finally, the binding of promoter regions for which the DNA was purified was detected by semi-quantitative PCR or qPCR. qPCR data were expressed as a percentage of the input DNA.

### Electrophoretic mobility shift assay (EMSA)

TCF7L2(1-456) protein was purchased from CUSABIO. The probe and mutant probe for the TCF7L2 binding site, labeled with FAM at the 3-terminal end, were synthesized by Sangon Biotech for the EMSA assay (Sangon Biotech Co., Ltd., Shanghai, China). After annealing, the probes were incubated with 1.25 μg TCF7L2(1-456) protein in binding buffer [Tris-HCl (pH 8.0), 10 mM, NaCl 50 mM, MgCl_2_ 1 mM, EDTA 0.5 mM, DTT 0.5 mM and glycerol 4%] for 40 min at room temperature. The reaction concentration of the probe and mutant probe was 0.1 μM, while the cold competitive probe was used at 1 μM. The incubated products were subjected to electrophoresis using 4% non-denatured polyacrylamide gel and detected with the ChemiDoc MP Imaging System.

### Statistical analysis

To investigate the relationship between fluorescence intensity of GFP protein or mRNA and changes in cell diameter in zebrafish oocytes, a locally estimated scatterplot smoothing (LOESS) curve was fitted using a standard tricubic weight function with a span parameter of 0.75. Statistical analyses were performed using R statistical software. The code required to reproduce the figures presented in this study is publicly available on GitHub (https://github.com/songsongh977-cmd/Fluorescence-intensity-statistics).

All experiments were repeated at least three times. All experimental data were analyzed using GraphPad Prism version 9. Values are presented as means ± standard deviation (SD). Statistical significance among experimental groups was determined using two-tailed, unpaired Student’s *t* test.

## Supplementary information

Appendix

Peer Review File

Dataset EV1

Source data Fig. 1

Source data Fig. 2A-D F G

Source data Fig. 2E

Source data Fig. 3

Source data Fig. 4

Source data Fig. 5

EV Figures Source Data

Figure Source Data for Appendix Figures

Expanded View Figures

## Data Availability

The RNA-seq data have been deposited to the Sequence Read Archive database under accession number PRJNA1156060. The source data of this paper are collected in the following database record: biostudies:S-SCDT-10_1038-S44319-026-00775-8.
