# Supplementary material for: Stn1 promotes zebrafish oocyte development via amplifying Wnt/β-catenin signaling
Source: EMBO Rep. 2026 Apr 17;27(12):3252–76. doi: 10.1038/s44319-026-00775-8 (PMC13304171; doi:10.1038/s44319-026-00775-8)
Supplement: Supplementary file 1 — Appendix [file 44319_2026_775_MOESM1_ESM.pdf]

Appendix

Table of Contents

Appendix Figure S1…………………….………….. 2

Appendix Figure S2……………………….…….. 3

Appendix Figure S3…………………….……….. 3

Appendix Figure S4……………………….…….. 5

Appendix Figure S5……………………….…….. 6

Appendix Figure S6…………………….……….. 7

Appendix Figure S7……………………….…….. 8

References………...………………………….……. 8

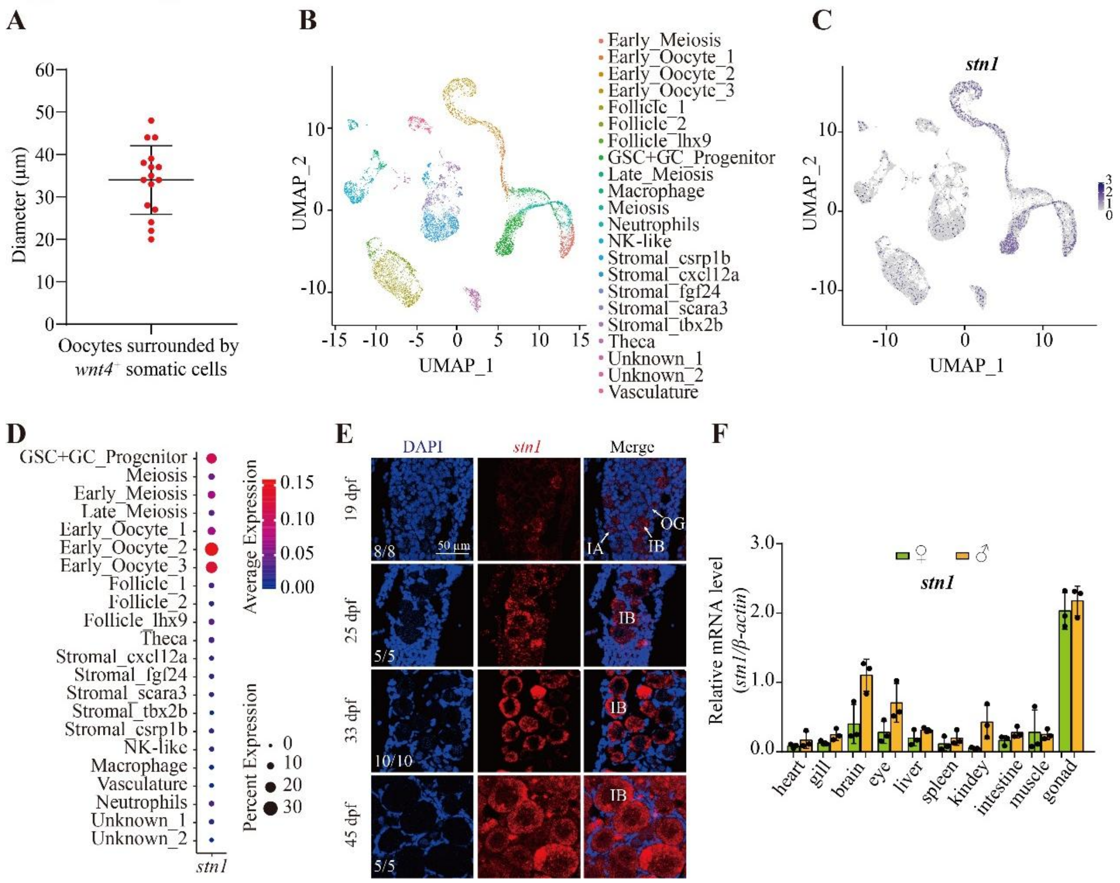

Appendix Figure S1. Characterization of *wnt4* and *stn1* expression. (A) Size distribution of oocytes surrounded by *wnt4*$^+$ somatic cells in gonads from 20 to 25 dpf, which was quantitated from Figure 2E–G in Kossack et al., 2019 (Kossack et al., 2019). (B, C) Gene expression plots of *stn1* mRNA in cell clusters from the ovaries of 40-day-old zebrafish. Uniform manifold approximation and project (UMAP) visualization of cell clusters from the ovaries of 40-day-old zebrafish. Cells expressing the indicated gene are purple; relative intensity indicates relative expression levels (intensity scale for each plot is on the right). (D) Dot plot showing the expression of *stn1* in cell clusters in (B). (E) Expression of *stn1* mRNA in ovaries at indicated developmental stages. Scale bar: 50 µm. The proportion of sections with the indicated phenotypes is shown in the bottom left corner of each panel. Each section was obtained from an individual zebrafish. OG: oogonia; IA: stage IA; IB: stage IB. (F) Expression of *stn1* mRNA in different tissues of adult female and male zebrafish (6-month-old) measured by real-time qPCR. Data were obtained from three independent biological experiments. Values are represented as means ± SD.

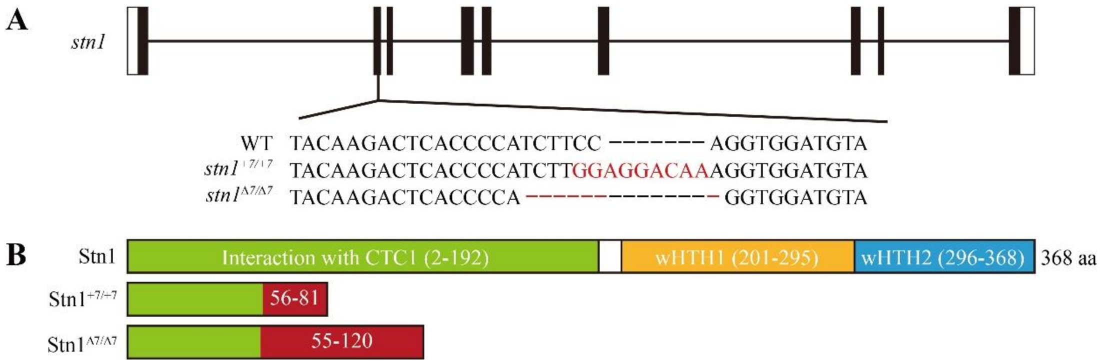

Appendix Figure S2. Generation of zebrafish *stn1* mutant lines. (A) Schematic diagram of the *stn1* gene structure. Black boxes: exons, white boxes: UTR, black lines: introns. Variations in different mutant strains compared to the wild-type gene sequence are highlighted in red. (B) Schematic diagram of the Stn1 protein structure. Variations in different mutant strains compared to the wild-type protein sequence are highlighted in red. wHTH, winged helix-turn-helix.

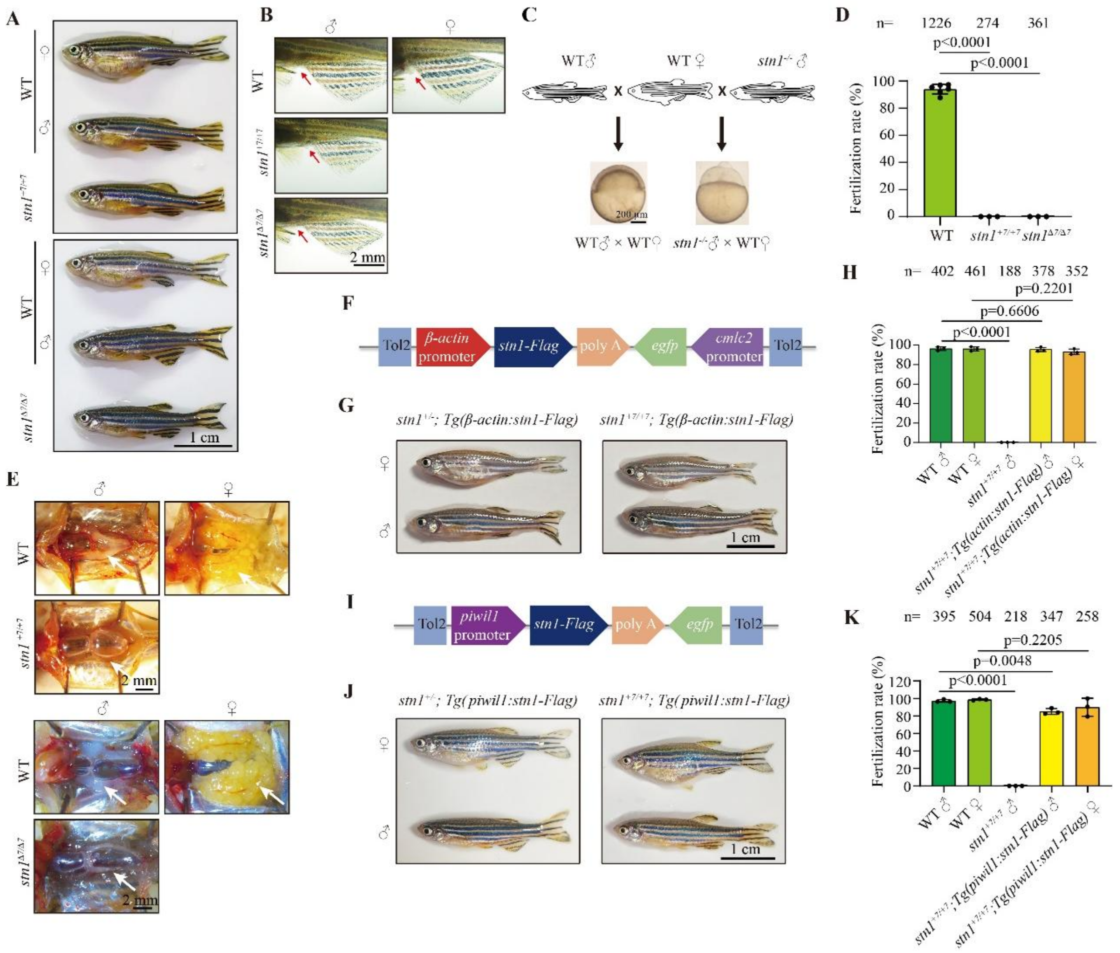

Appendix Figure S3. Genetic inactivation of Stn1 results in a male-like phenotype associated with infertility. (A) Representative images of wild-type sibling and *stn1* mutant fish (3-month-old). Scale bar: 1 cm. (B) Secondary sex characteristics of adult wild-type and *stn1* mutant fish (3-month-old). Representative genital papilla (red arrow) of wild-type and *stn1*

mutants. Scale bar: 2 mm. (C) Schematic representation of the experimental design for mating a wild-type female fish with wild-type male and mutant male-like fish. A wild-type female fish was first mated with a wild-type male and then with a mutant male-like fish within 30 min after spawning; the mating order of wild-type male and mutant male-like fish was reversed in the next week. Representative images of embryos at 6 hpf from different mating combination are shown. Whether fertilization has occurred is determined by whether the embryo reaches the shield stage by 6 hpf. Scale bar: 200 μm. (D) Fertilization rate of the wild-type female fish mated with wild-type male or mutant male-like fish. The fertilization rate of each pair of mated fish was counted when offspring developed to shield stage. Each dot represents a wild-type male or mutant male-like fish. The total embryo numbers (n) are given at the top of columns. Values are represented as means ± SD; Unpaired *t*-test, two-tailed. (E) Gonads from adult wild-type sibling and *stn1* mutant fish (3-month-old). Representative gonads (white arrow) of siblings and *stn1* mutants. Scale bar: 2 mm. (F) Diagram of the construct for generating *Tg*(*actin:stn1-Flag*) transgenic fish. (G) Representative images of sibling and *stn1* mutant fish with a *Tg*(*actin:stn1-Flag*) transgenic background (3-month-old). Scale bar: 1 cm. (H) Fertilization rate of indicated genotypic fish crossed with wild-type adult fish. Each dot represents an adult fish and the total embryo numbers (n) are given at the top of columns. Values are represented as means ± SD; Unpaired *t*-test, two-tailed. (I) Diagram of the construct for generating *Tg*(*piwil1:stn1-Flag*) transgenic fish. (J) Representative images of sibling and *stn1* mutant fish with a *Tg*(*piwil1:stn1-Flag*) transgenic background (3-month-old). Scale bar: 1 cm. (K) Fertilization rate of indicated genotypic fish crossed with wild-type adult fish. Each dot represents an adult fish and the total embryo numbers (n) are given at the top of columns. Values are represented as means ± SD; Unpaired *t*-test, two-tailed.

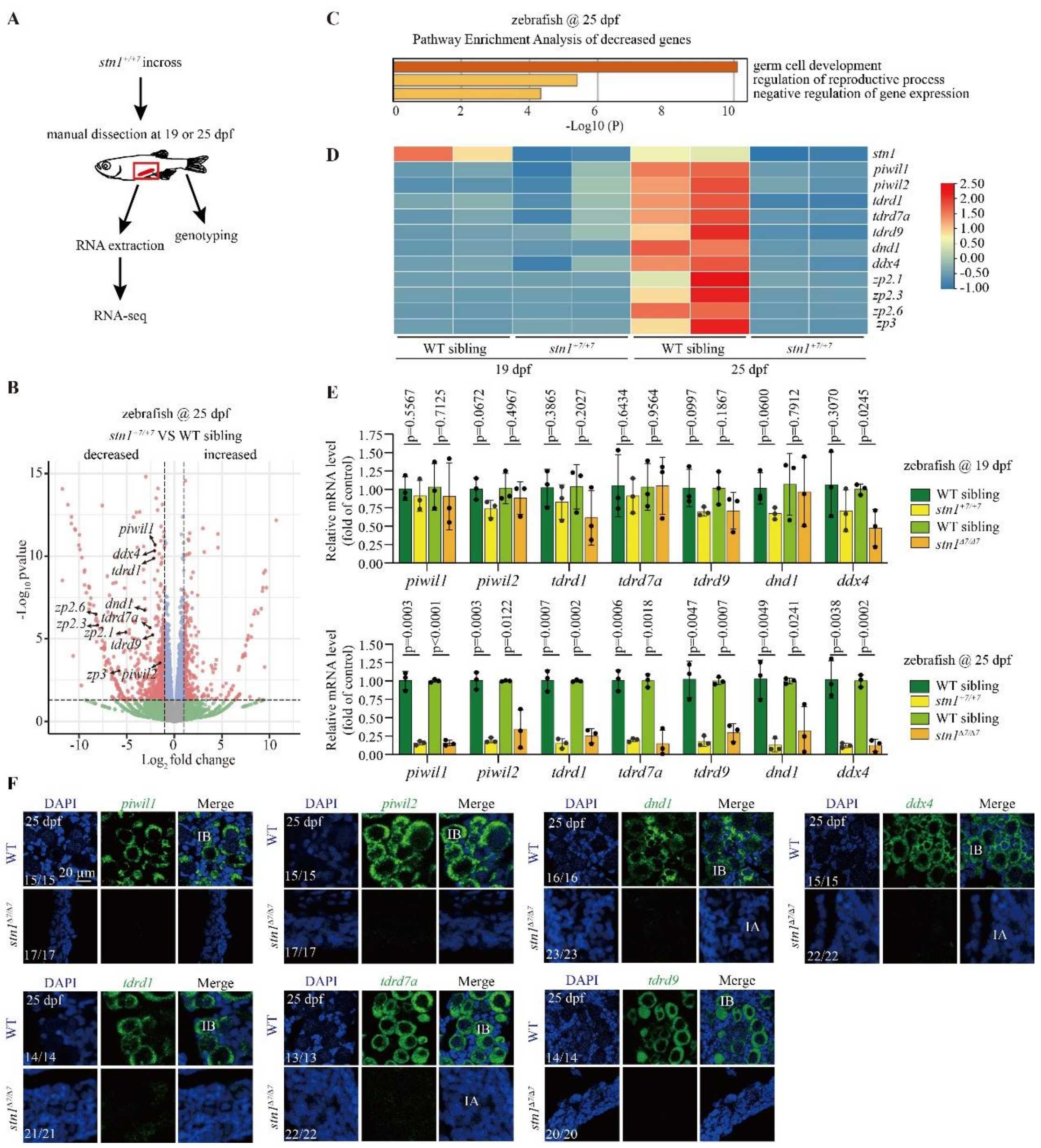

Appendix Figure S4. Loss of Stn1 reduces the expression abundance of germ cell-specific genes. (A) Schematic representation of the experimental design for the RNA sequencing (RNA-seq) approach. Progenies of *stn1*$^{+/-}$ fish incrosses were raised to 19 or 25 dpf and subjected to genotyping. RNA extraction was performed on manually dissected trunk region of fish (indicated by the red box). (B) Volcano plot showing a set of decreased germ cell development-related genes in *stn1* mutants at 25 dpf with a fold change < 0.5 and $P < 0.05$. (C) Gene ontology (GO) enrichment analysis showing the three most relevant biological processes associated with the decreased genes in *stn1* mutants at 25 dpf; each process has a -log10(P) value > 4. (D) Heatmaps of transcripts showing the reduced germ cell development-related genes in *stn1* mutants at 19 and 25 dpf. Heatmaps show results from two replicates. (E) qRT-PCR validation of the indicated genes in (D). Values are represented as means ± SD from three independent biological experiments; Unpaired *t*-test, two-tailed. (F) Expression of indicated germ cell-specific genes in wild-type sibling, and *stn1* mutant embryos at 25 dpf. Scale bar: 20 μm. The

proportion of sections with the indicated phenotypes is shown in the bottom left corner of each panel. Each section was obtained from an individual zebrafish. IA: stage IA; IB: stage IB.

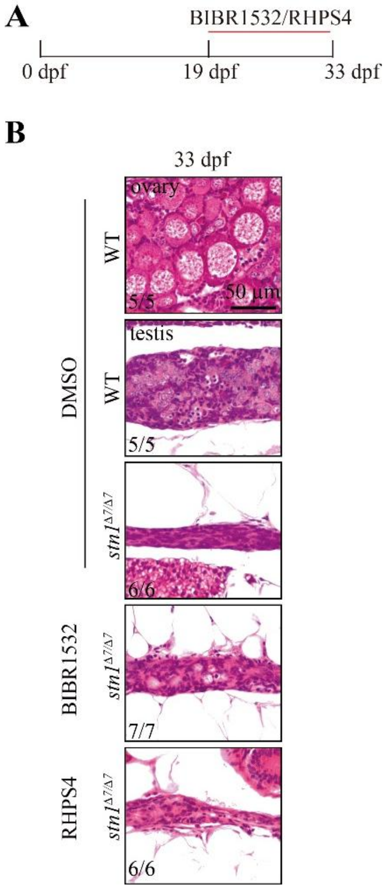

Appendix Figure S5. Pharmacological inhibition of telomerase activity does not restore germ cell loss caused by *stn1* deletion. (A) Outline of the experimental design on juvenile zebrafish treated with small-molecule inhibitors of telomerase. Progeny from *stn1*$^{+/-}$ crosses were raised to 19 dpf and subsequently exposed to BIBR1532 or RHPS4 daily until 33 dpf. At 33 dpf, juvenile zebrafish were harvested, genotyped, and their gonads dissected for histological analysis. (B) Representative histological sections showing gonads from wild-type sibling and *stn1* mutant fish, with or without treatment with BIBR1532 or RHPS4, at 33 dpf. Scale bar: 50 μm. The proportion of sections with the indicated phenotypes is shown in the bottom left corner of each panel. Each section was obtained from an individual zebrafish.

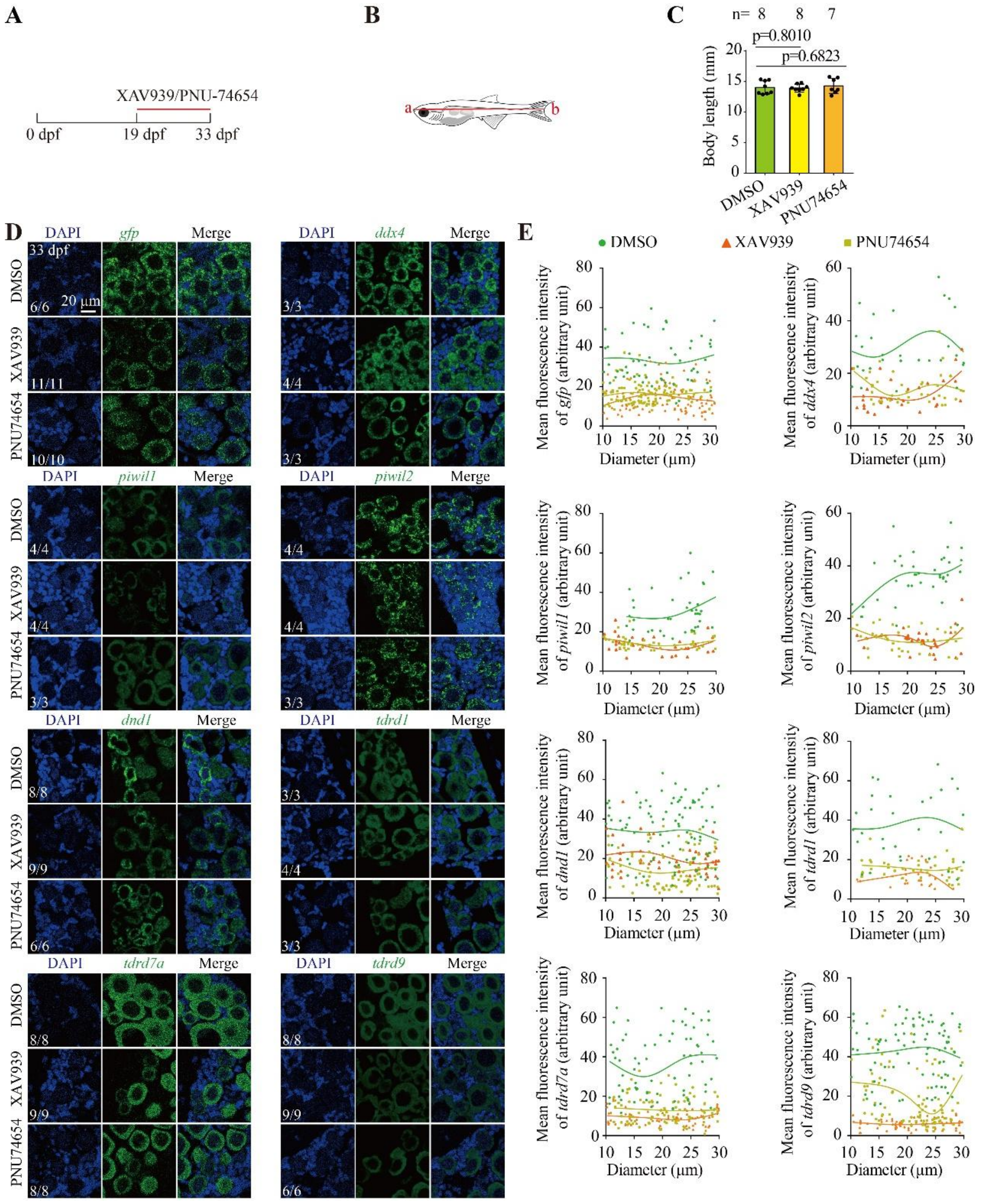

Appendix Figure S6. Transcriptional response of various germ cell-specific genes to inhibition of Wnt/β-catenin signaling. (A) Outline of the experimental design on juvenile zebrafish treated with small-molecule inhibitors of Wnt/β-catenin signaling. Wild-type embryos were raised to 19 dpf and subsequently exposed to XAV939 or PNU-74654 daily until 33 dpf. At 33 dpf, juvenile zebrafish were harvested and their gonads dissected for FISH analysis. (B) Quantification of the body length of a-b (mouth to end of tail) in juvenile fish at 33 dpf. (C) Quantitative results of the body length. The total number (n) of juvenile zebrafish of each group are given at the top. Values are represented as means ± SD; Unpaired *t*-test, two-tailed. (D) Transcriptional levels of indicated genes in juvenile zebrafish at 33 dpf treated with or without XAV939 or PNU-74654, as determined by FISH analysis. Representative confocal

images of gonads from female zebrafish at 33 dpf following staining for each indicated mRNA. Scale bar: 20 μm. The proportion of sections with the indicated phenotypes is shown in the bottom left corner of each panel. Each section was obtained from an individual zebrafish. (E) Quantitative results from images shown in (D). Each data point represents an individual oocyte. Counts of zebrafish treated with DMSO, XAV939, or PNU-74654 were as follows: 6, 11, and 10 for *gfp*; 8, 9, and 6 for *dnd1* and *tdrd9*; 3, 4, and 3 for *ddx4* and *tdrd*1; 4, 4, and 3 for *piwil1* and *piwil2*; and 8, 9, and 8 for *tdrd7a*, respectively.

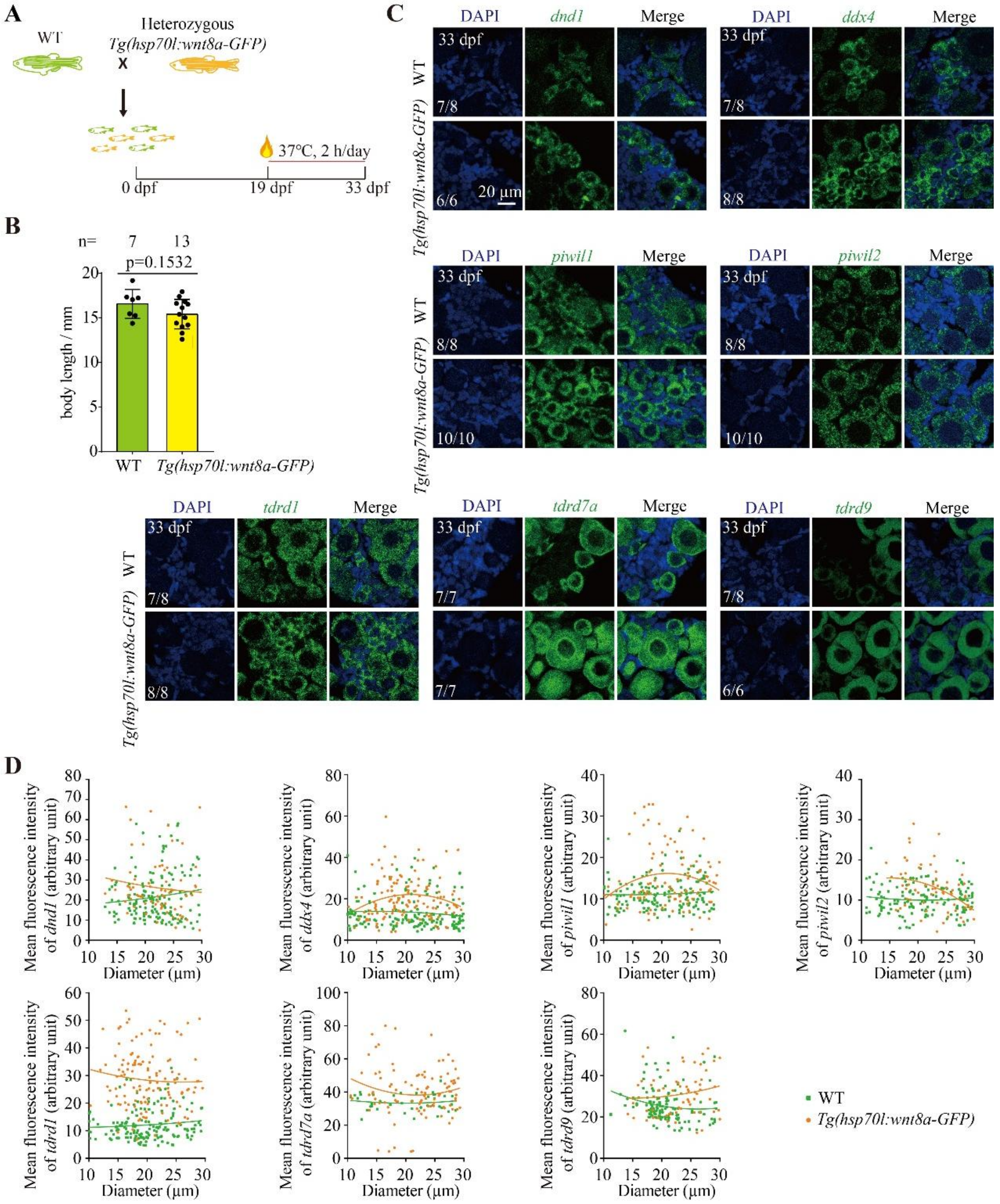

Appendix Figure S7. Transcriptional response of various germ cell-specific genes to activation of Wnt/β-catenin signaling. (A) Progenies of heterozygous *Tg(hsp70l:wnt8a-GFP)* transgenic fish outcrossed with wild-type fish were raised to 19 dpf and exposed to a 2-h heat

shock (37 °C) daily to 33 dpf. (B) Quantification of the body length of a-b (mouth to end of tail) in juvenile fish at 33 dpf. The total number (n) of juvenile zebrafish of each group are given at the top. Values are represented as means ± SD; Unpaired *t*-test, two-tailed. (C) Transcriptional levels of indicated genes in juvenile zebrafish with or without *Tg(hsp70l:wnt8a-GFP)* transgenic background at 33 dpf after inducible expression of Wnt8a, as indicated by FISH analysis. The progeny were genotyped and subjected to FISH analysis. Representative confocal images of gonads from female zebrafish at 33 dpf following staining for each indicated mRNA. Scale bar: 20 μm. The proportion of sections with the indicated phenotypes is shown in the bottom left corner of each panel. Each section was obtained from an individual zebrafish. (D) Quantitative results from images shown in (C). Each data point represents an individual oocyte. Counts of wild-type siblings and *stn1*$^{\Delta7/\Delta7}$*; Tg(hsp70l:wnt8a-GFP)* zebrafish were as follows: 8 and 6 for *dnd1* and *tdrd9*; 8 and 8 for *ddx4* and *tdrd1*; 8 and 10 for *piwil1* and *piwil2*; and 7 and 7 for *tdrd7a*, respectively.

References

Kossack ME, High SK, Hopton RE, Yen YL, Postlethwait JH, Draper BW (2019) Female Sex Development and Reproductive Duct Formation Depend on Wnt4a in Zebrafish. *Genetics* 211: 219-233
