# Supplementary figures and images for: Stn1 promotes zebrafish oocyte development via amplifying Wnt/β-catenin signaling (part 1 of 5)
Source: EMBO Rep. 2026 Apr 17;27(12):3252–76. doi: 10.1038/s44319-026-00775-8 (PMC13304171; doi:10.1038/s44319-026-00775-8)

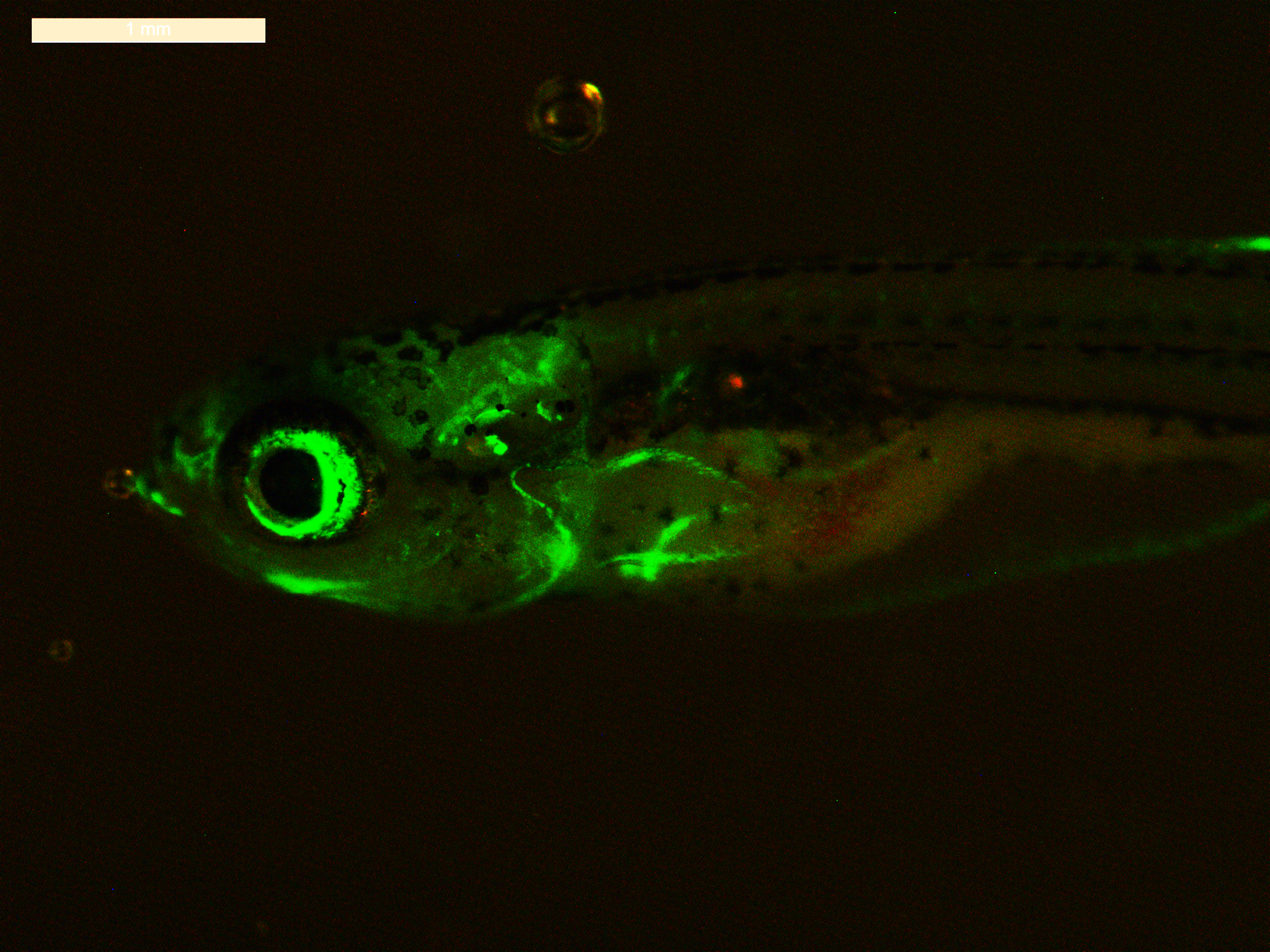

Supplement: Supplementary file 4 — Source data Fig. 1 [file 44319_2026_775_MOESM4_ESM.zip › Figure 1/Figure 1A/GFP 19 dpf.tif]

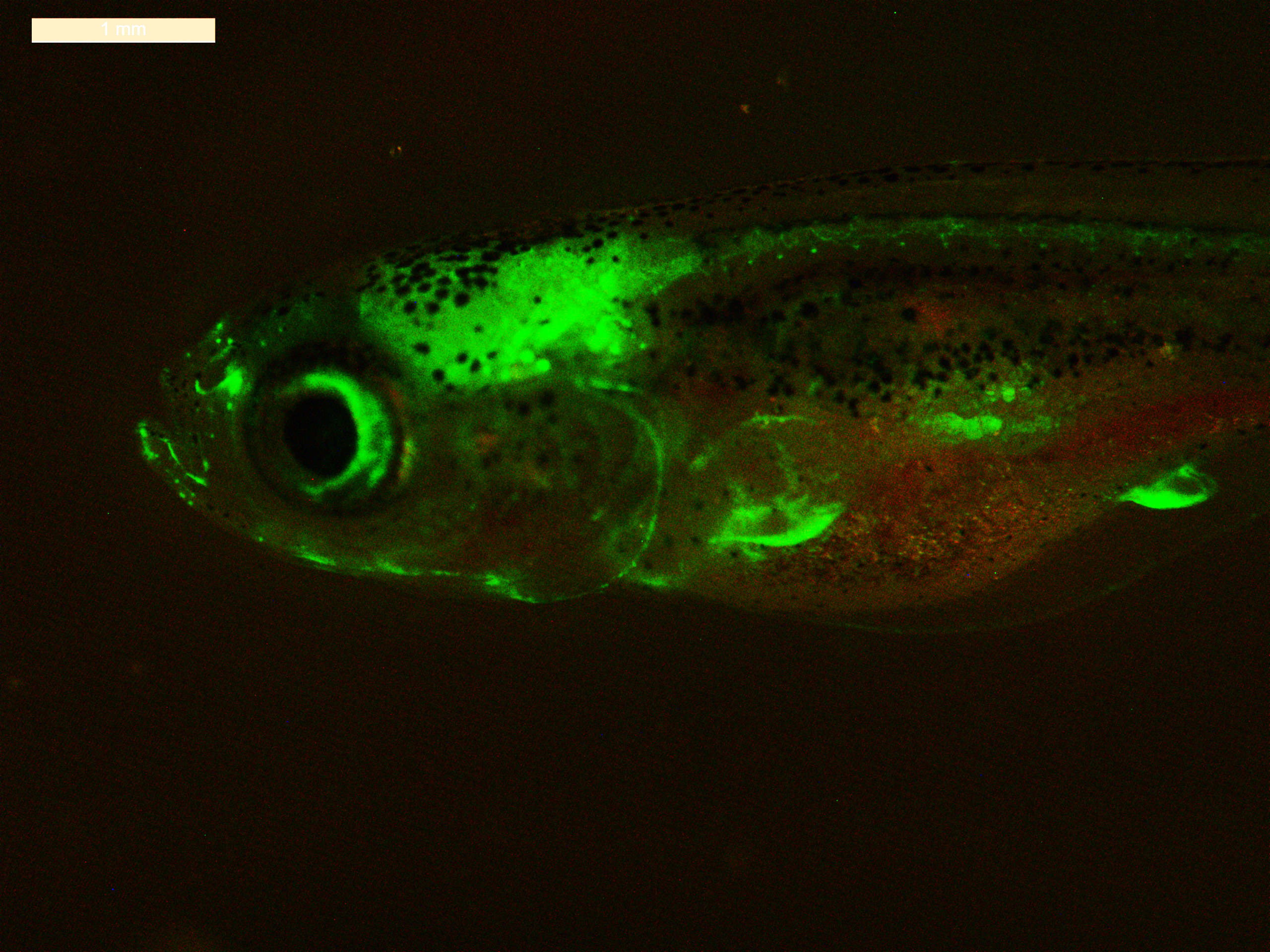

Supplement: Supplementary file 4 — Source data Fig. 1 [file 44319_2026_775_MOESM4_ESM.zip › Figure 1/Figure 1A/GFP 25 dpf.tif]

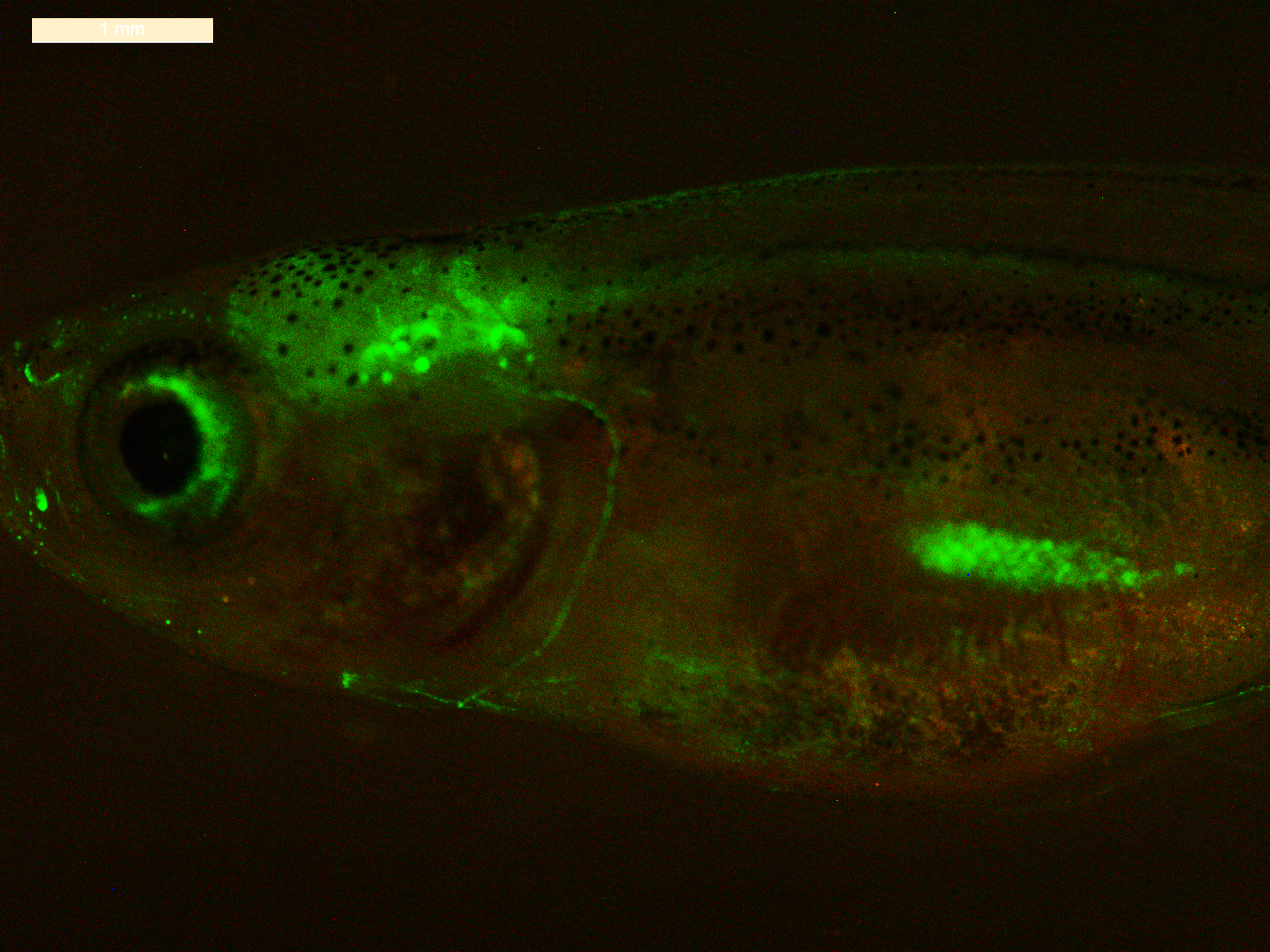

Supplement: Supplementary file 4 — Source data Fig. 1 [file 44319_2026_775_MOESM4_ESM.zip › Figure 1/Figure 1A/GFP 33 dpf.tif]

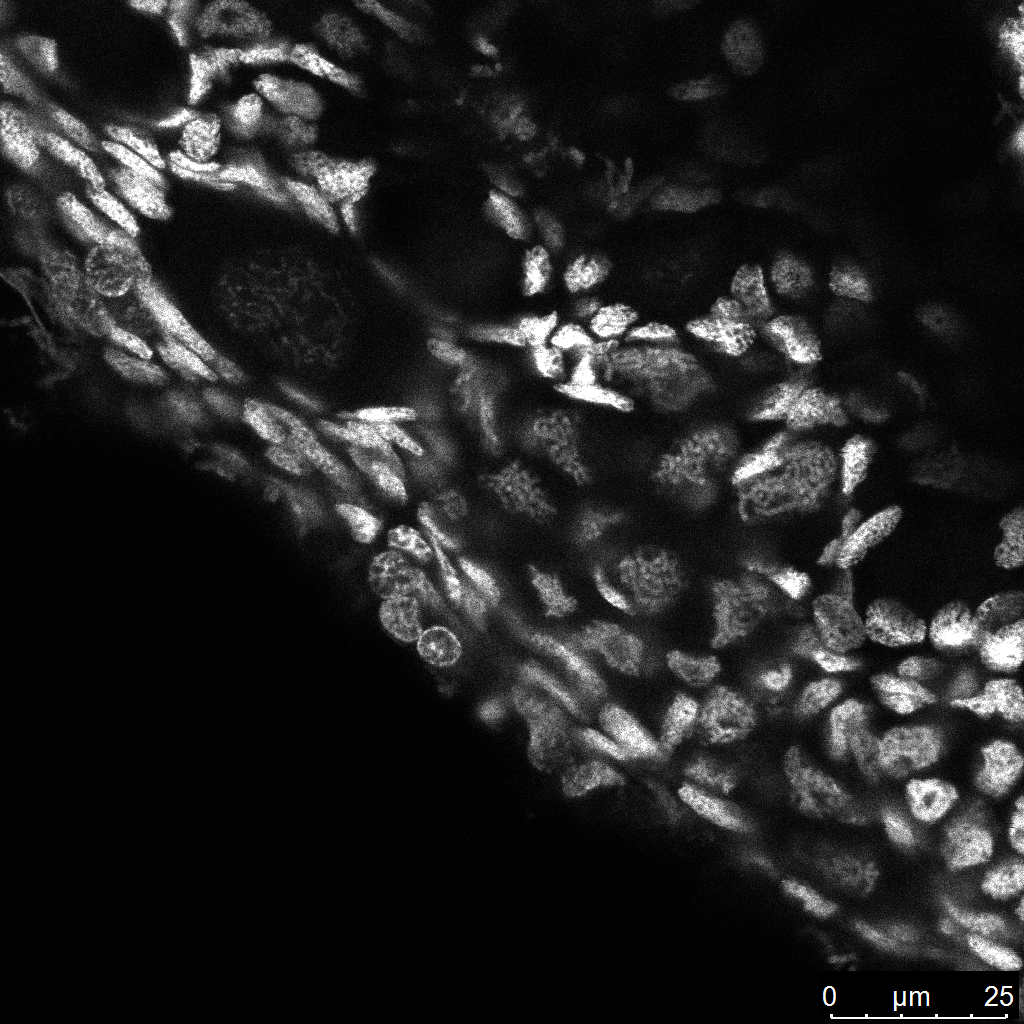

Supplement: Supplementary file 4 — Source data Fig. 1 [file 44319_2026_775_MOESM4_ESM.zip › Figure 1/Figure 1B/19 dpf DAPI.tif]

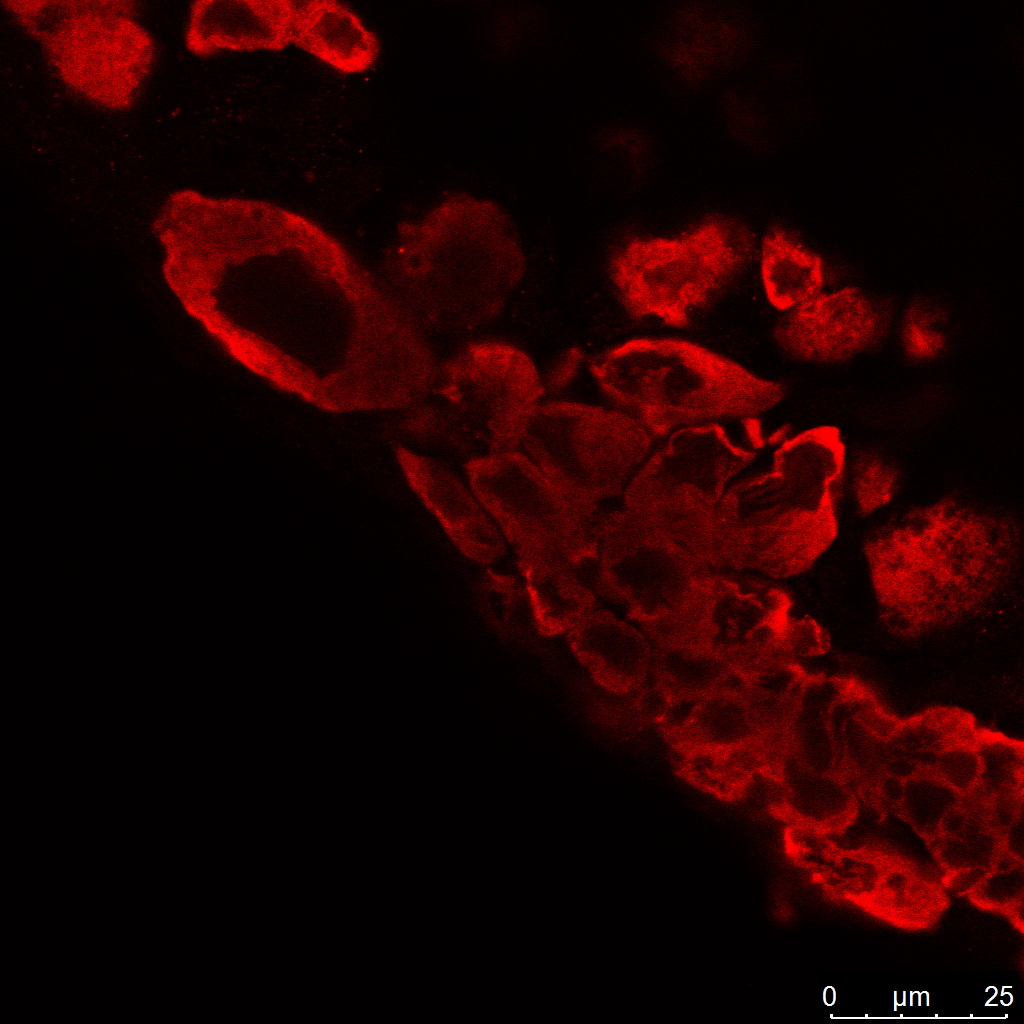

Supplement: Supplementary file 4 — Source data Fig. 1 [file 44319_2026_775_MOESM4_ESM.zip › Figure 1/Figure 1B/19 dpf Ddx4.tif]

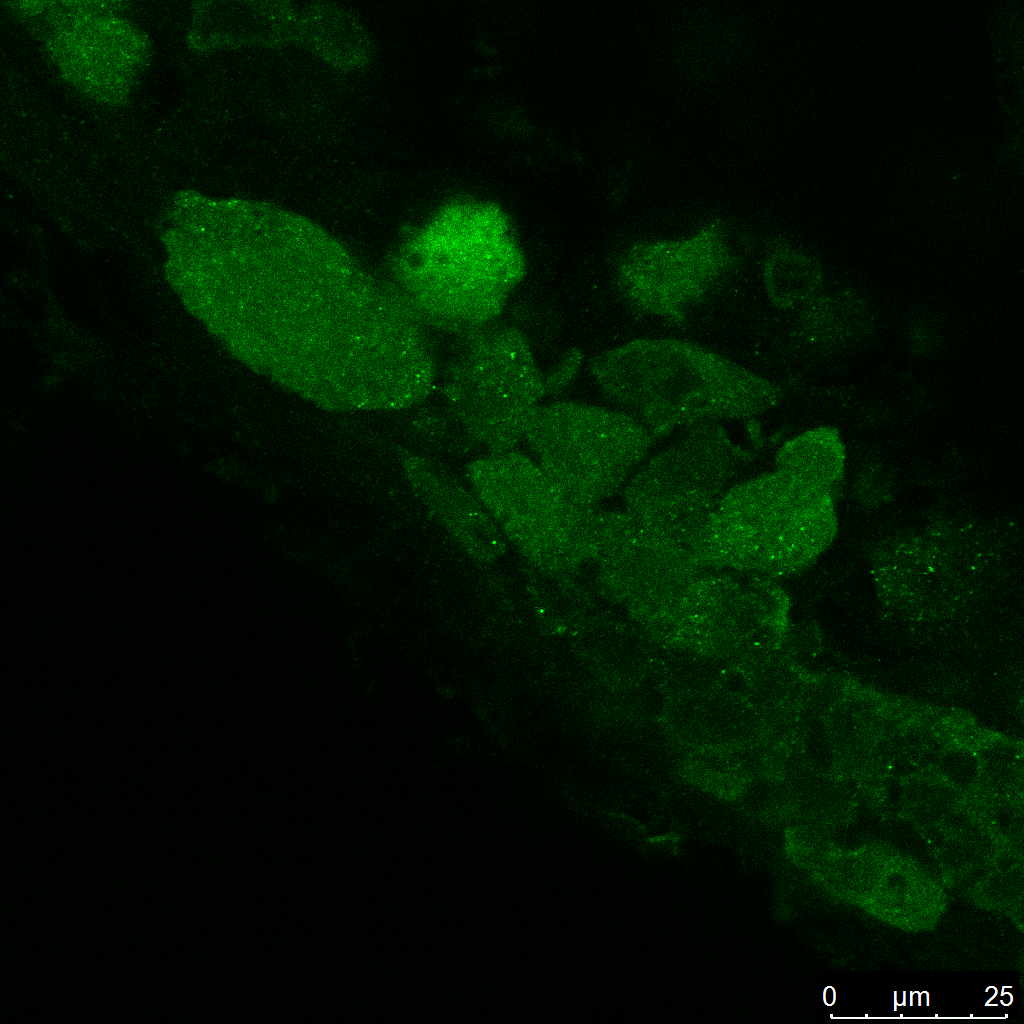

Supplement: Supplementary file 4 — Source data Fig. 1 [file 44319_2026_775_MOESM4_ESM.zip › Figure 1/Figure 1B/19 dpf GFP.tif]

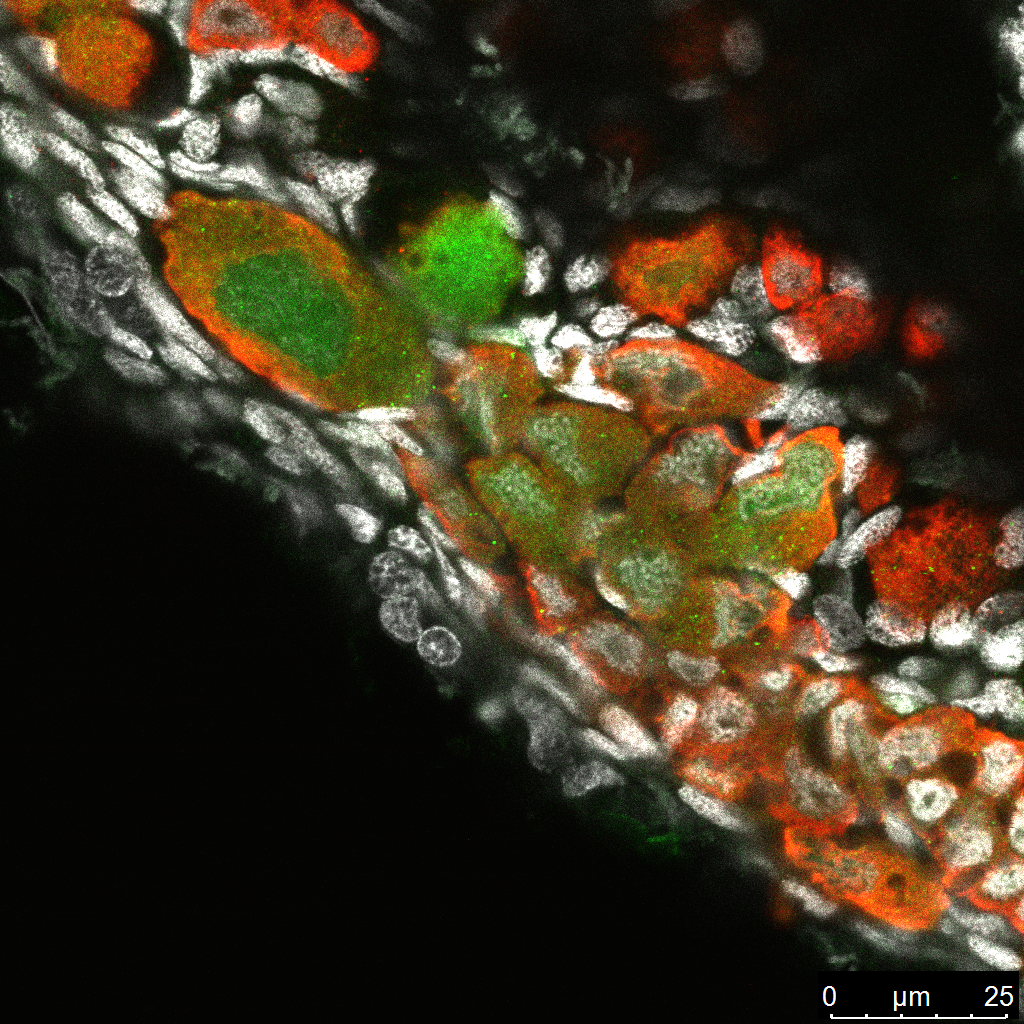

Supplement: Supplementary file 4 — Source data Fig. 1 [file 44319_2026_775_MOESM4_ESM.zip › Figure 1/Figure 1B/19 dpf Merge.tif]

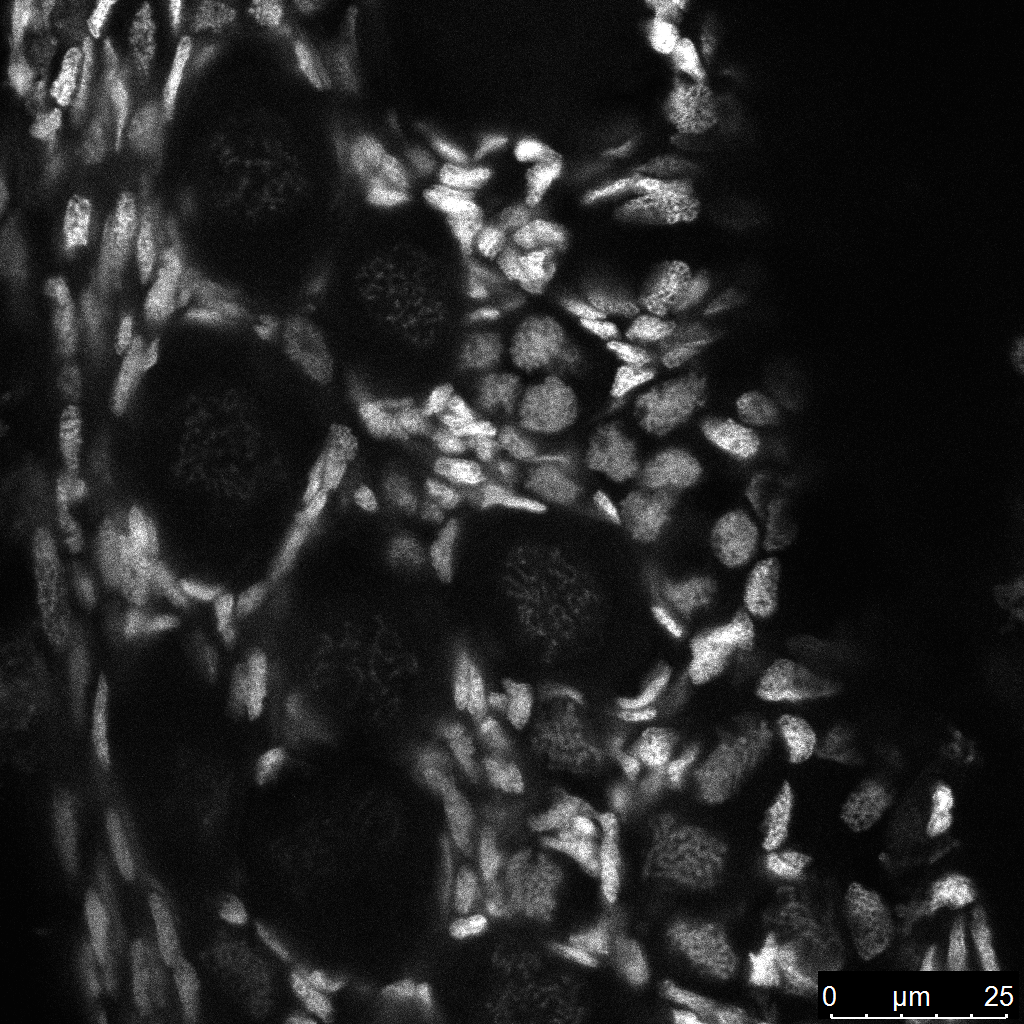

Supplement: Supplementary file 4 — Source data Fig. 1 [file 44319_2026_775_MOESM4_ESM.zip › Figure 1/Figure 1B/25 dpf DAPI.tif]

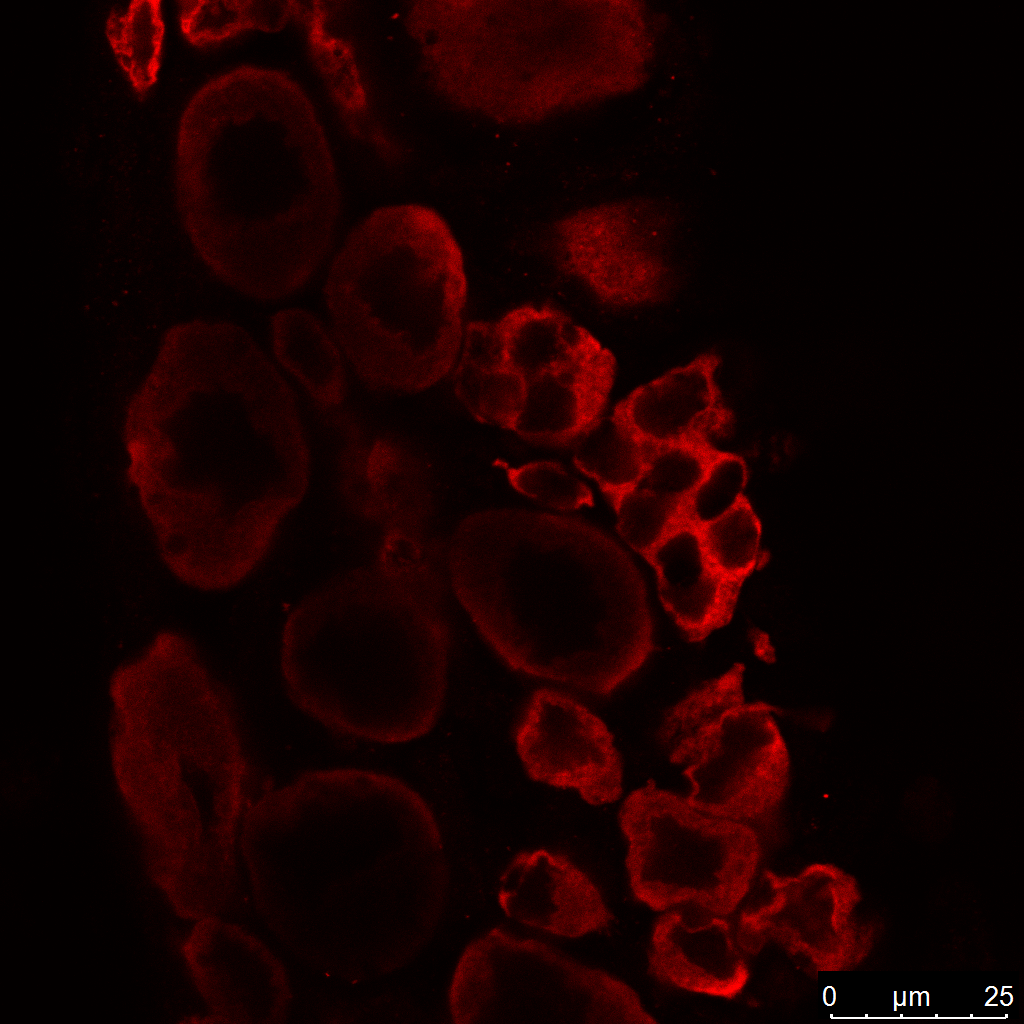

Supplement: Supplementary file 4 — Source data Fig. 1 [file 44319_2026_775_MOESM4_ESM.zip › Figure 1/Figure 1B/25 dpf Ddx4.tif]

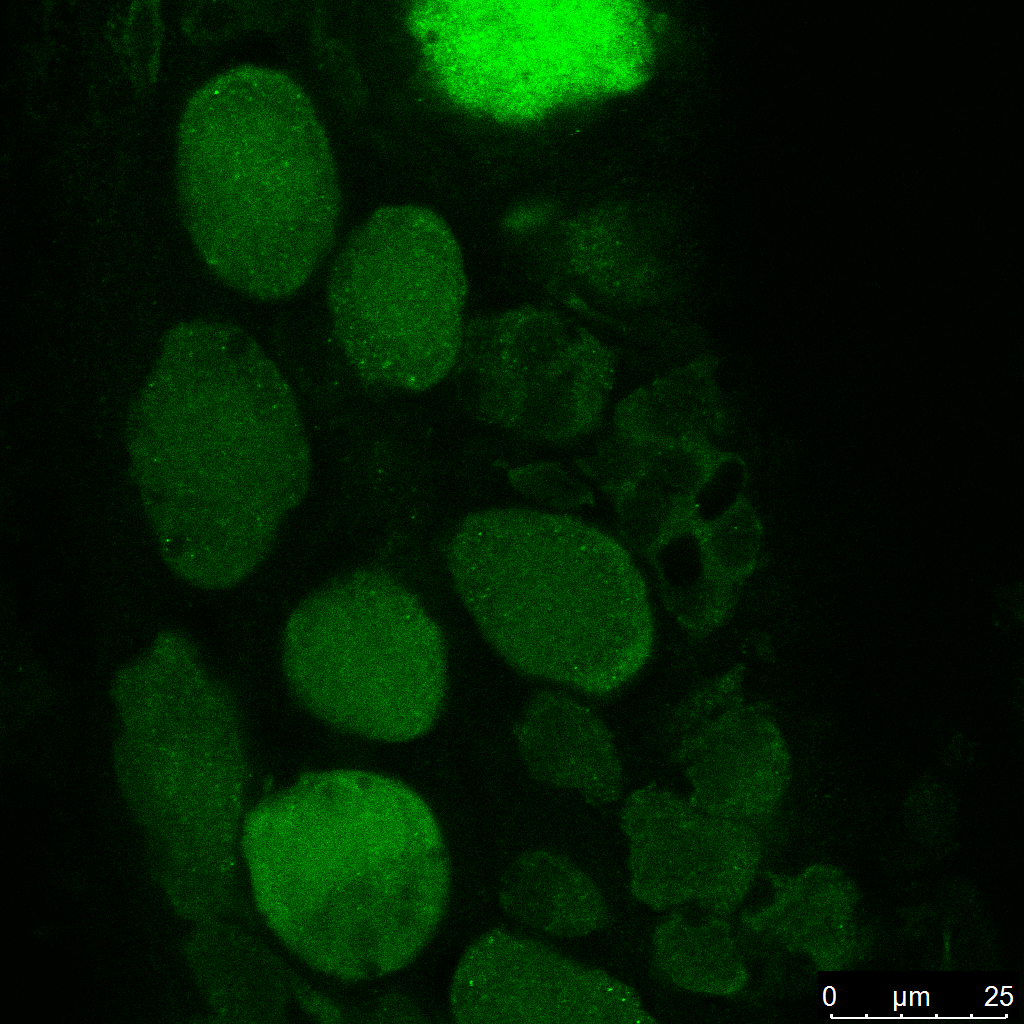

Supplement: Supplementary file 4 — Source data Fig. 1 [file 44319_2026_775_MOESM4_ESM.zip › Figure 1/Figure 1B/25 dpf GFP.tif]

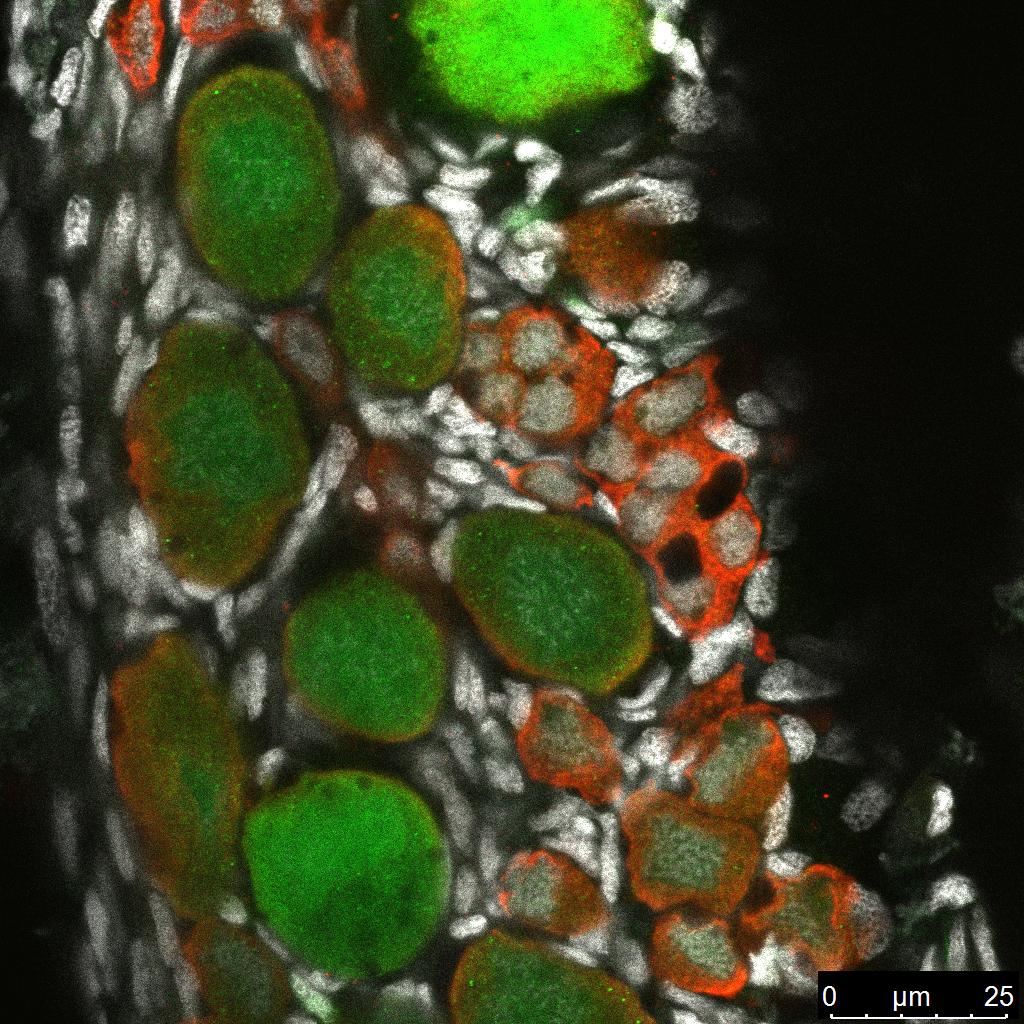

Supplement: Supplementary file 4 — Source data Fig. 1 [file 44319_2026_775_MOESM4_ESM.zip › Figure 1/Figure 1B/25 dpf Merge.tif]

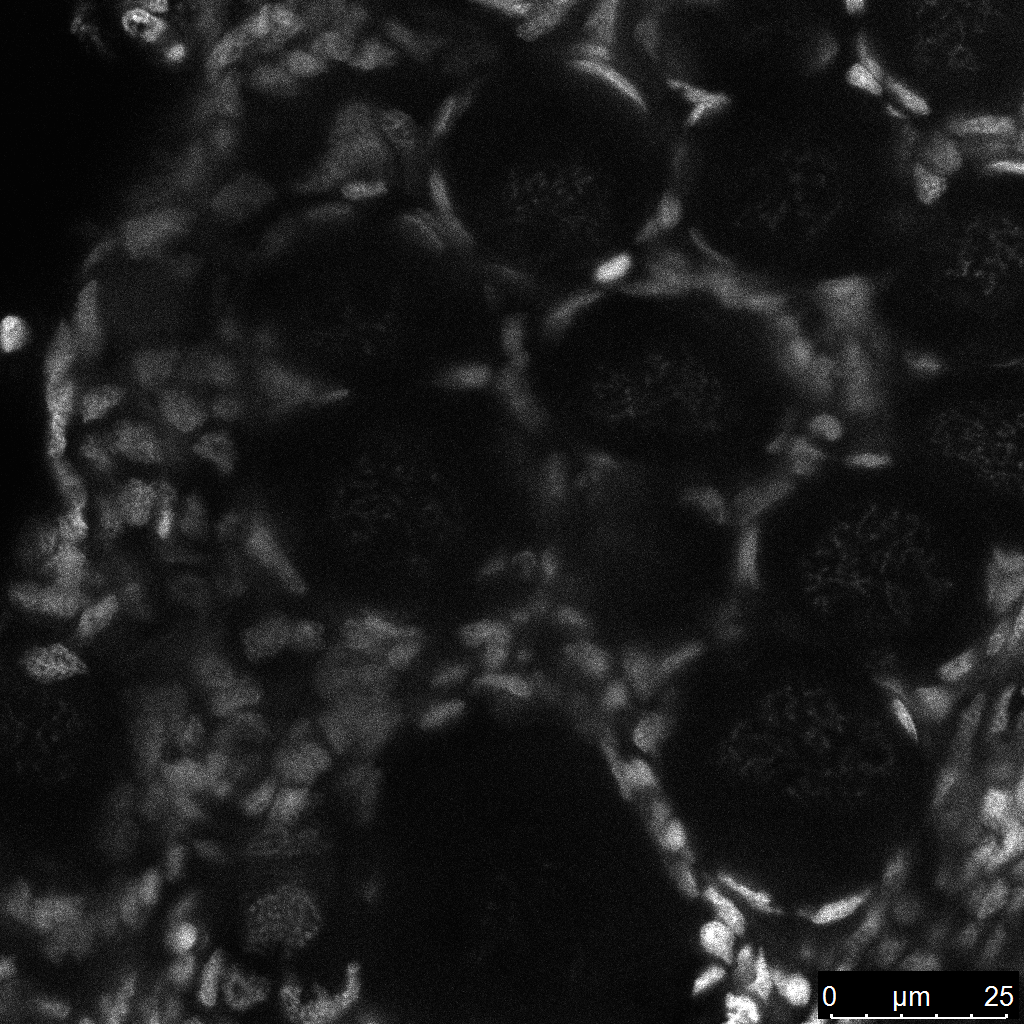

Supplement: Supplementary file 4 — Source data Fig. 1 [file 44319_2026_775_MOESM4_ESM.zip › Figure 1/Figure 1B/33 dpf DAPI.tif]

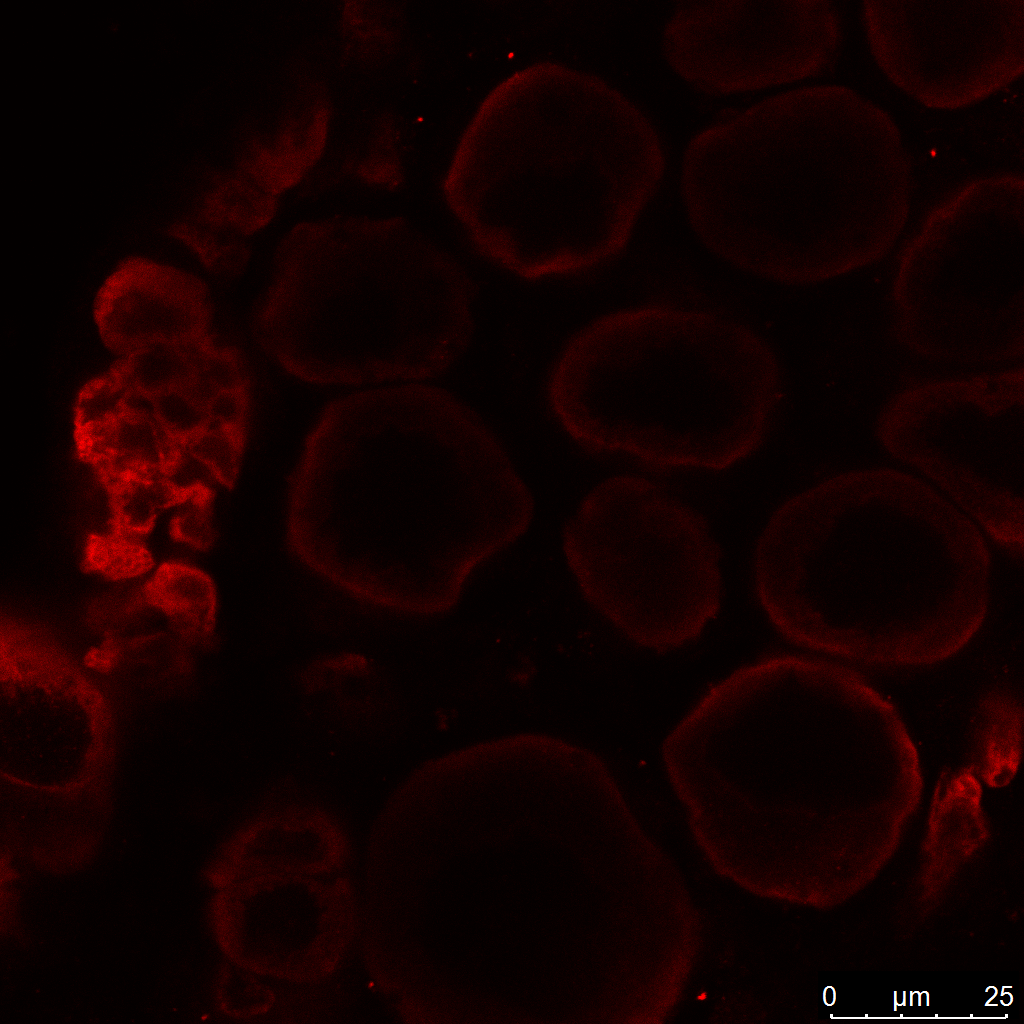

Supplement: Supplementary file 4 — Source data Fig. 1 [file 44319_2026_775_MOESM4_ESM.zip › Figure 1/Figure 1B/33 dpf Ddx4.tif]

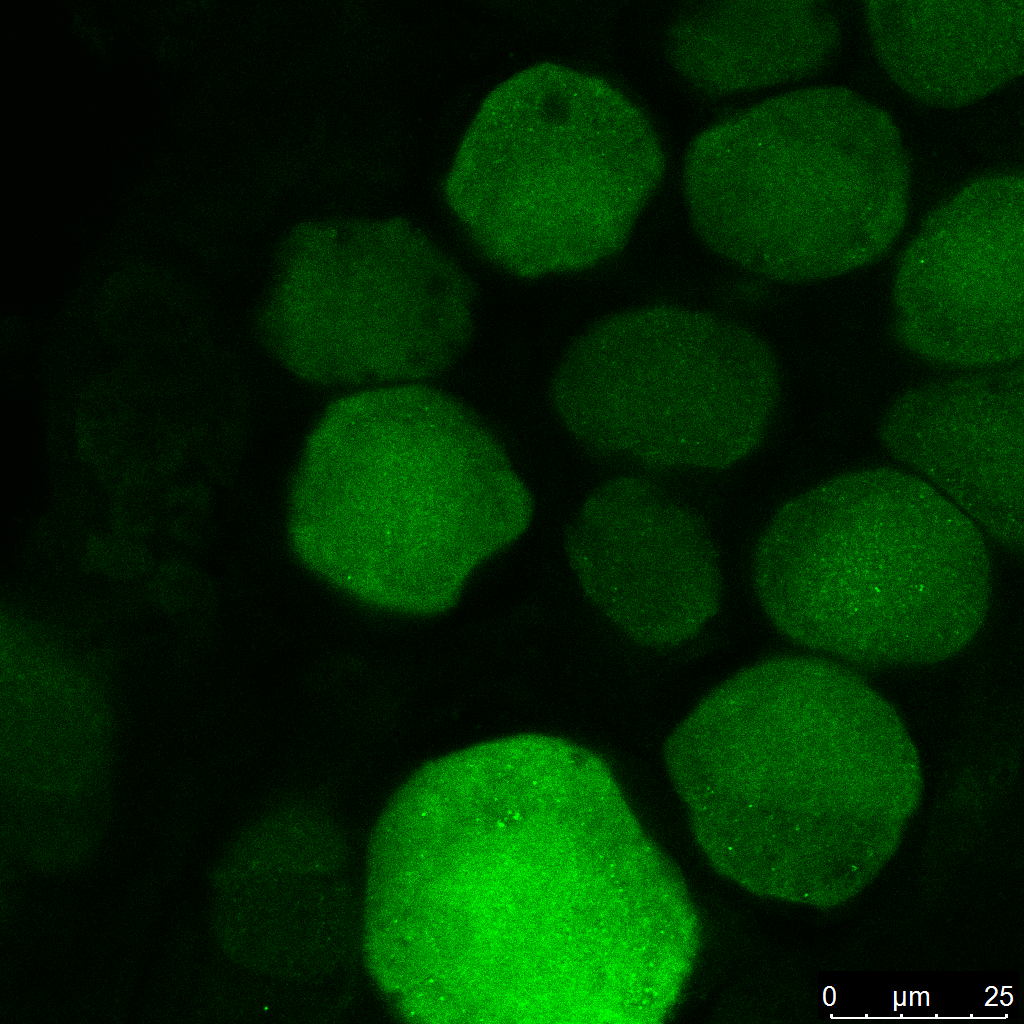

Supplement: Supplementary file 4 — Source data Fig. 1 [file 44319_2026_775_MOESM4_ESM.zip › Figure 1/Figure 1B/33 dpf GFP.tif]

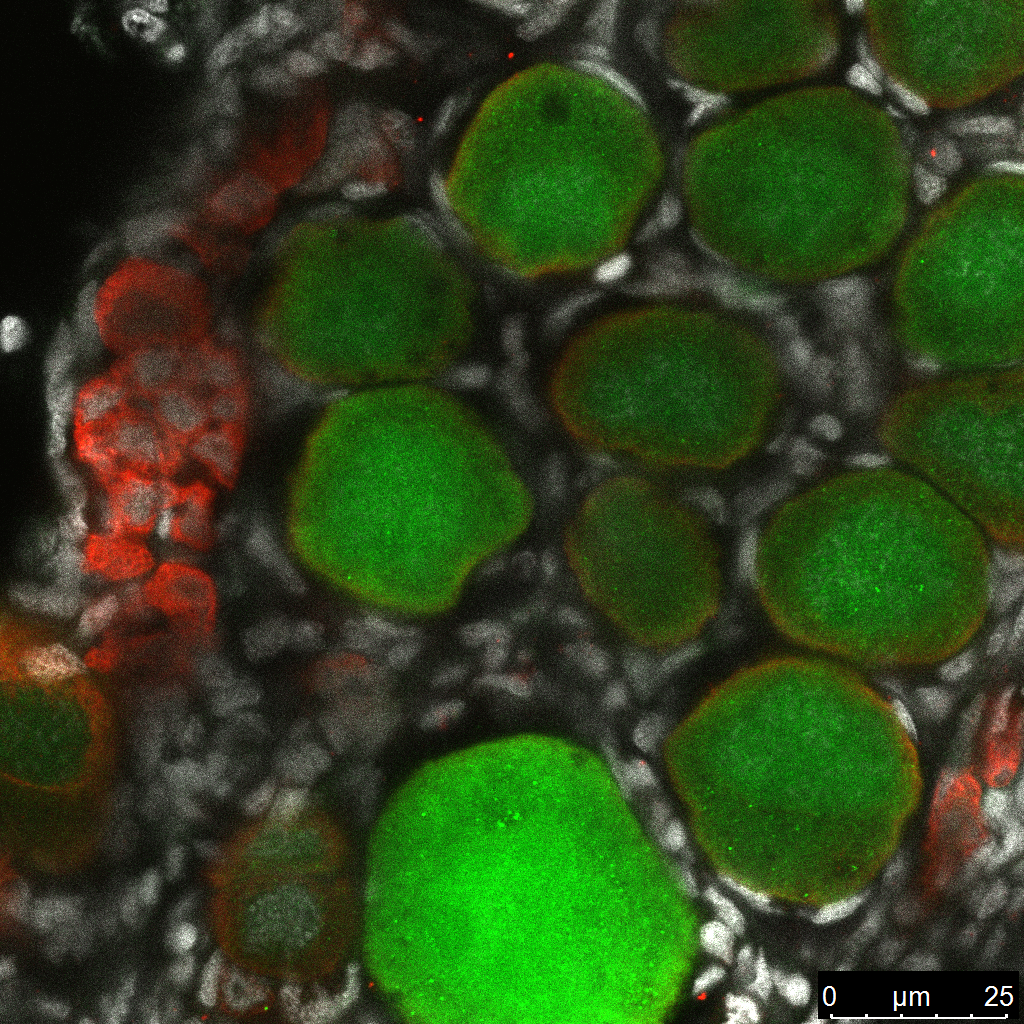

Supplement: Supplementary file 4 — Source data Fig. 1 [file 44319_2026_775_MOESM4_ESM.zip › Figure 1/Figure 1B/33 dpf Merge.tif]

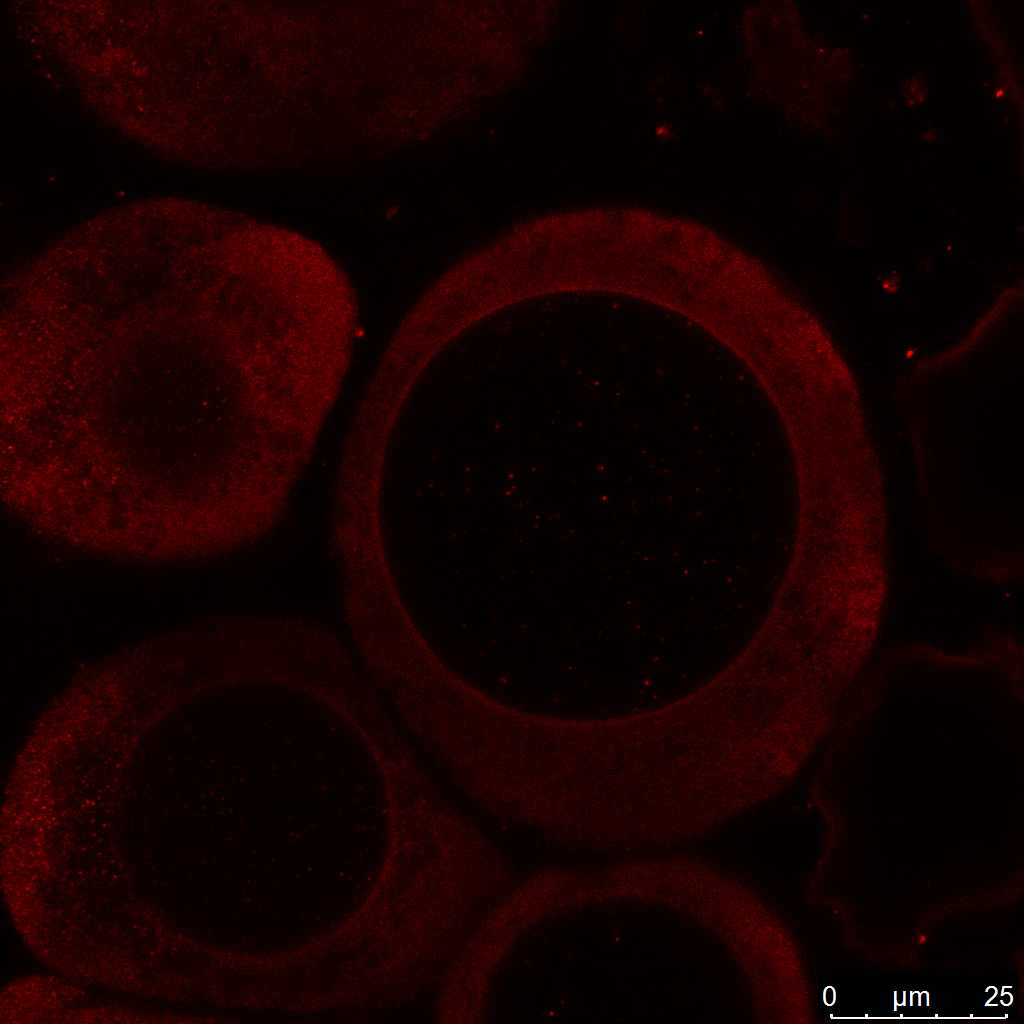

Supplement: Supplementary file 4 — Source data Fig. 1 [file 44319_2026_775_MOESM4_ESM.zip › Figure 1/Figure 1B/45 dpf Ddx4.tif]

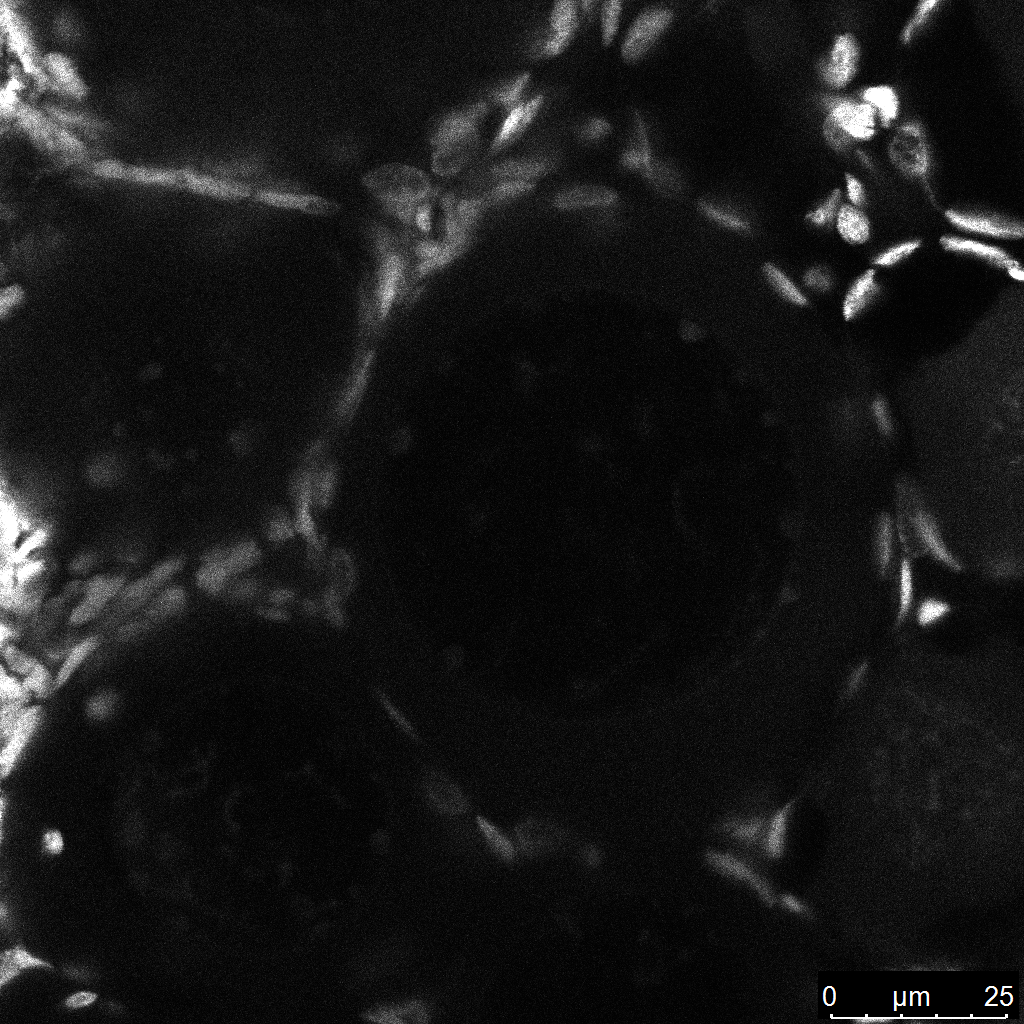

Supplement: Supplementary file 4 — Source data Fig. 1 [file 44319_2026_775_MOESM4_ESM.zip › Figure 1/Figure 1B/45 dpf DAPI.tif]

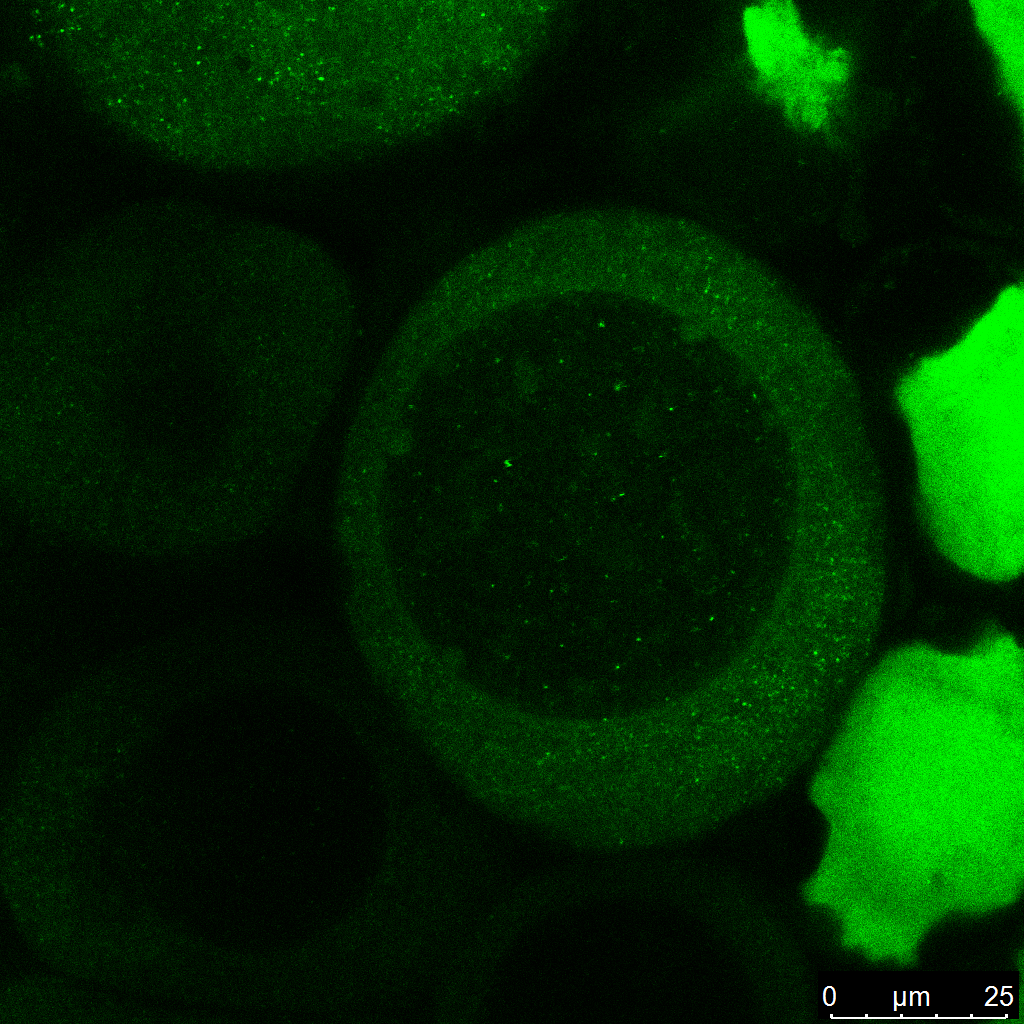

Supplement: Supplementary file 4 — Source data Fig. 1 [file 44319_2026_775_MOESM4_ESM.zip › Figure 1/Figure 1B/45 dpf GFP.tif]

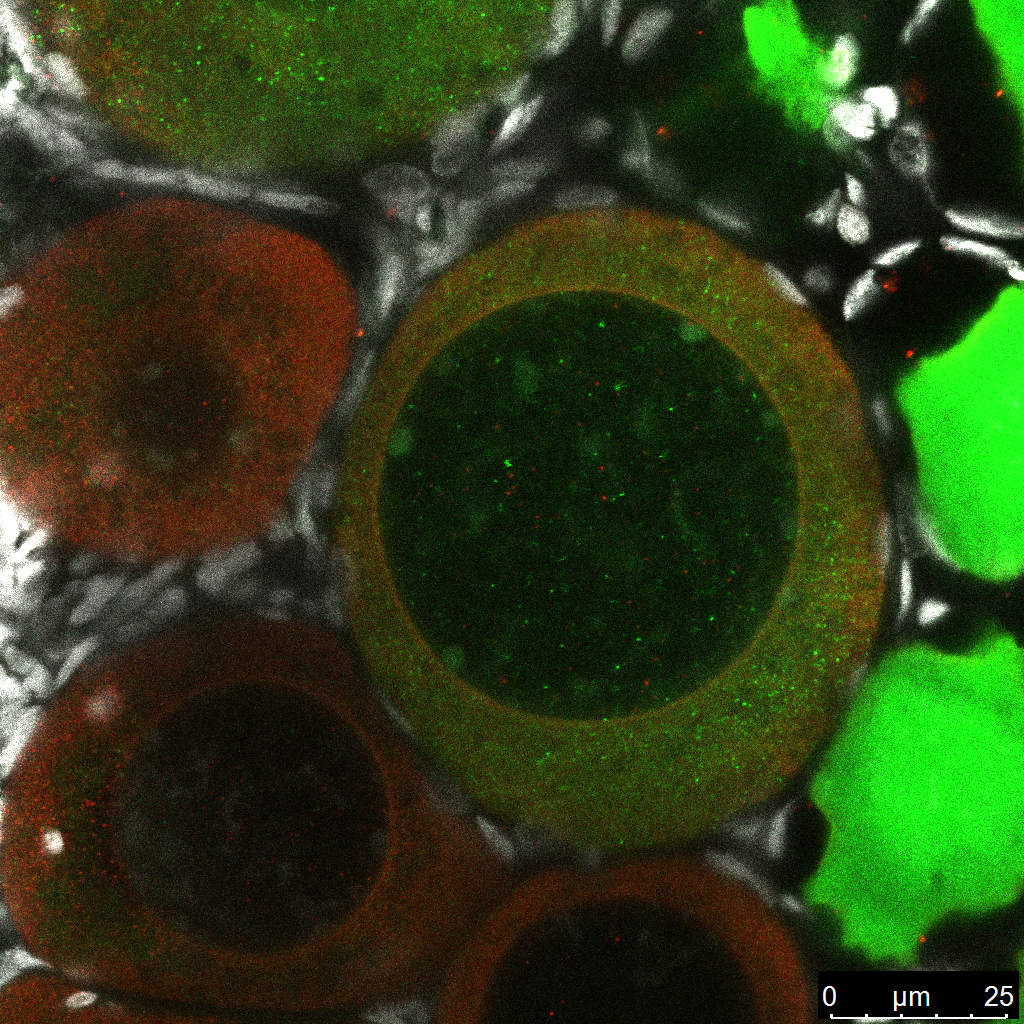

Supplement: Supplementary file 4 — Source data Fig. 1 [file 44319_2026_775_MOESM4_ESM.zip › Figure 1/Figure 1B/45 dpf Merge.tif]

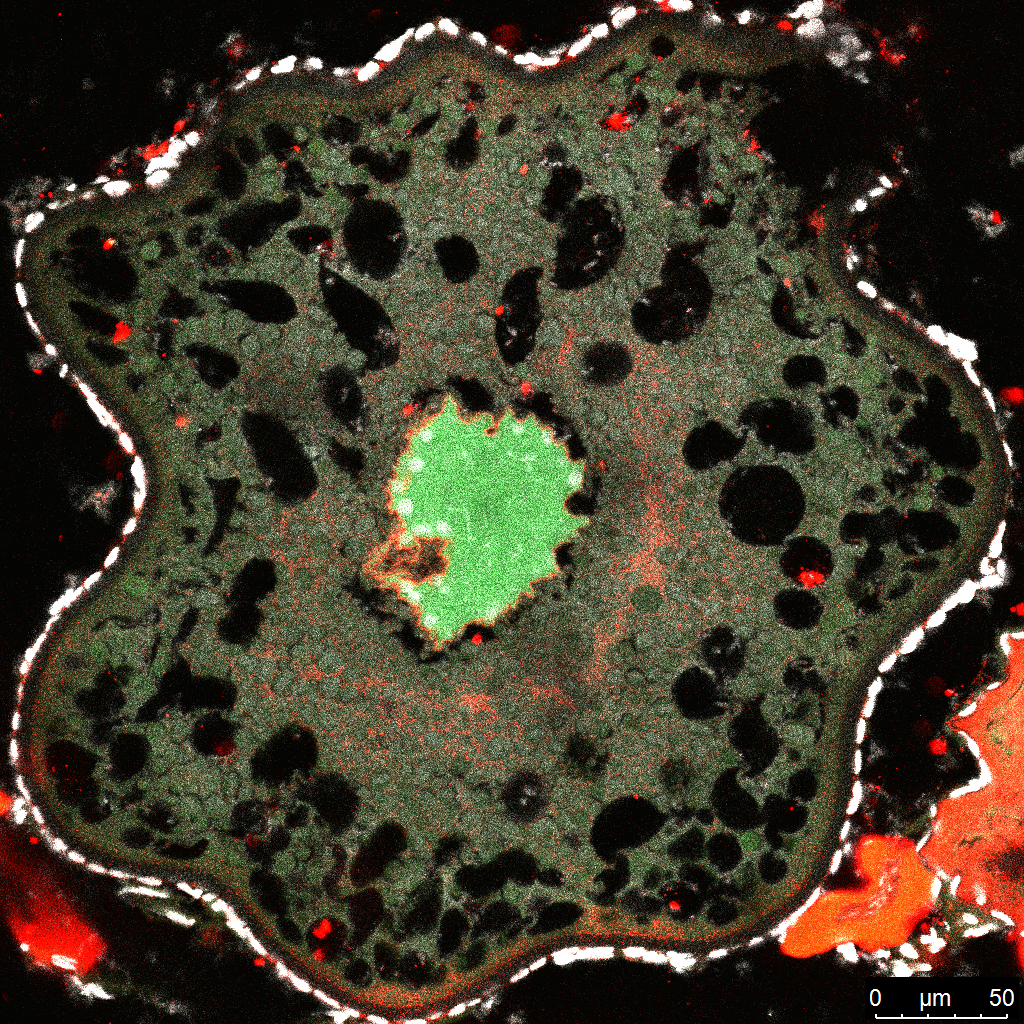

Supplement: Supplementary file 4 — Source data Fig. 1 [file 44319_2026_775_MOESM4_ESM.zip › Figure 1/Figure 1B/90 dpf Merge.tif]

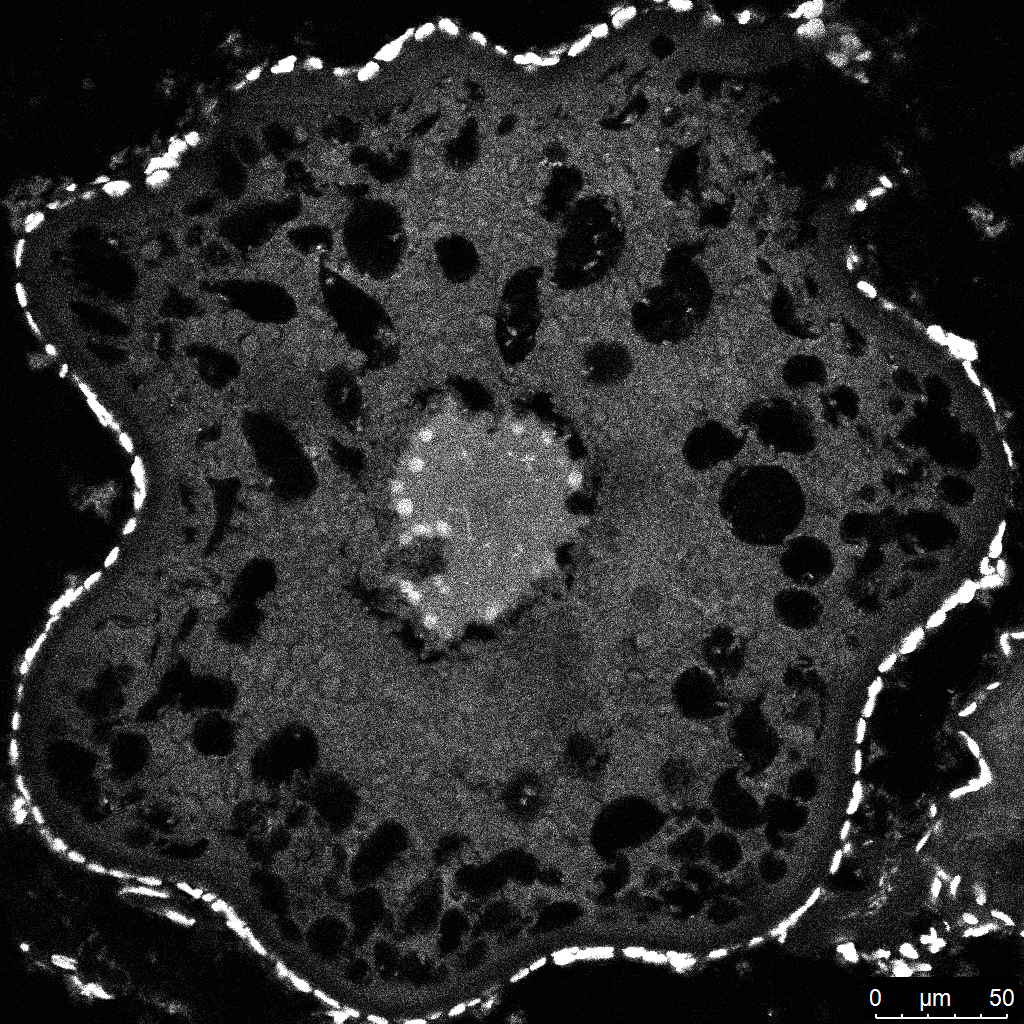

Supplement: Supplementary file 4 — Source data Fig. 1 [file 44319_2026_775_MOESM4_ESM.zip › Figure 1/Figure 1B/90 dpf DAPI.tif]

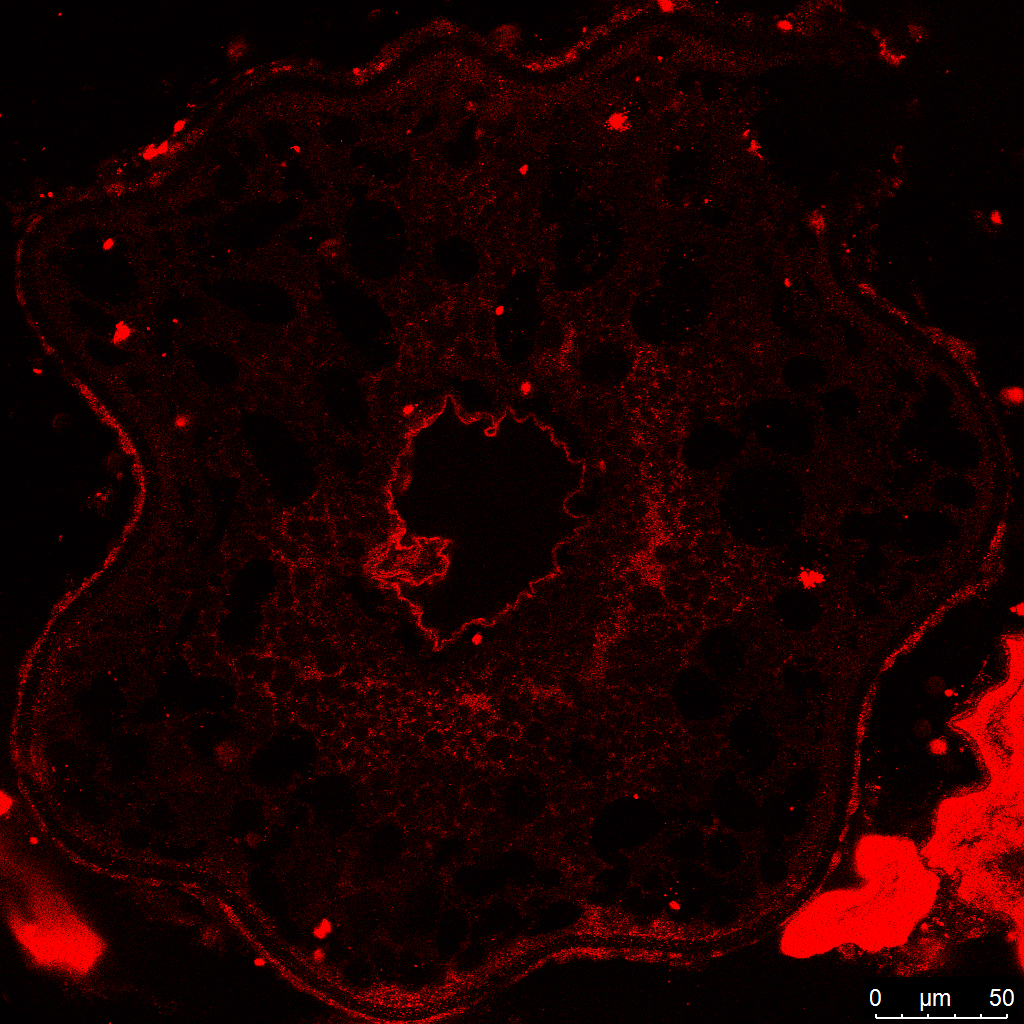

Supplement: Supplementary file 4 — Source data Fig. 1 [file 44319_2026_775_MOESM4_ESM.zip › Figure 1/Figure 1B/90 dpf Ddx4.tif]

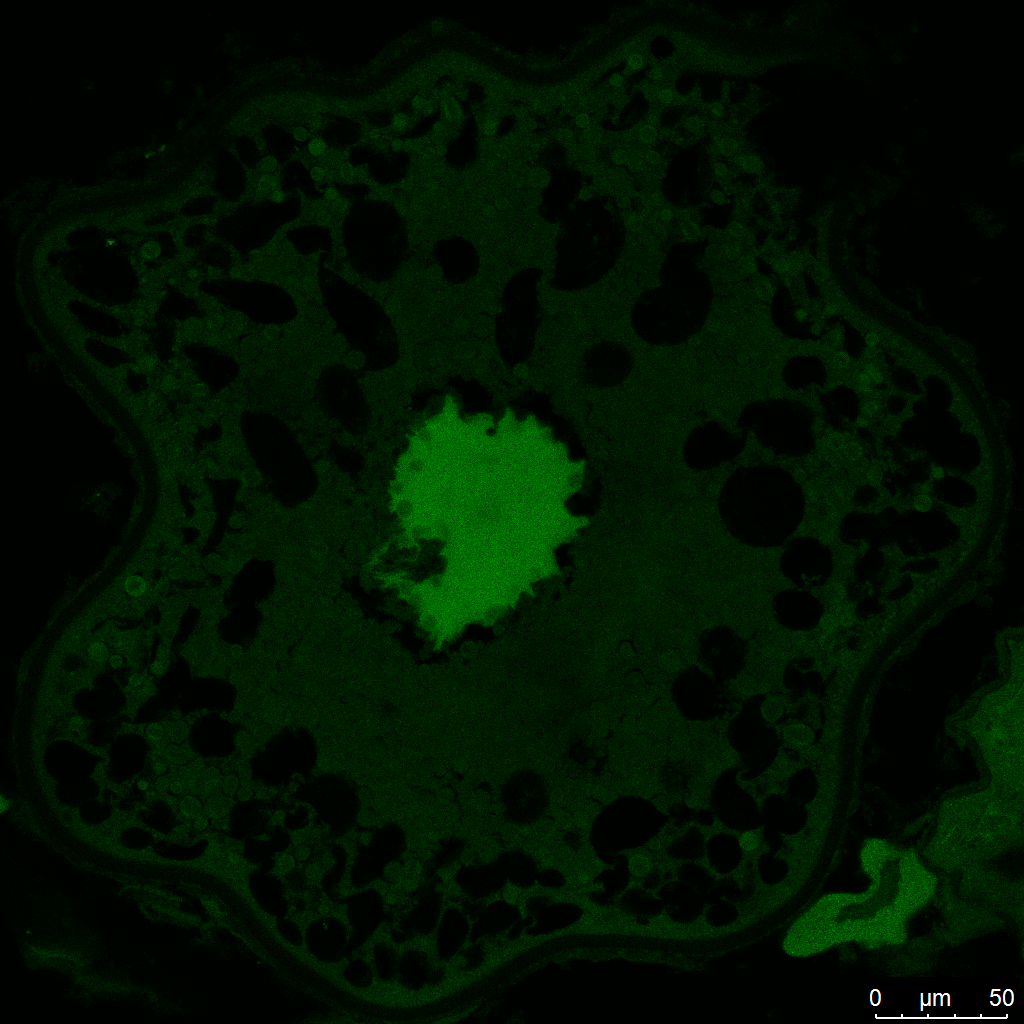

Supplement: Supplementary file 4 — Source data Fig. 1 [file 44319_2026_775_MOESM4_ESM.zip › Figure 1/Figure 1B/90 dpf GFP.tif]

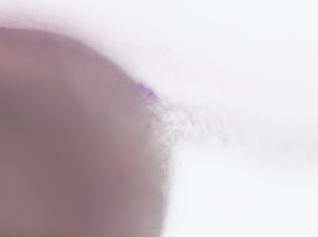

Supplement: Supplementary file 5 — Source data Fig. 2A-D F G [file 44319_2026_775_MOESM5_ESM.zip › Figure 2A-D F G/Figure 2A/ddx4 24 hpf lateral view het.tif]

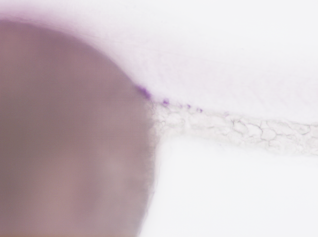

Supplement: Supplementary file 5 — Source data Fig. 2A-D F G [file 44319_2026_775_MOESM5_ESM.zip › Figure 2A-D F G/Figure 2A/ddx4 24 hpf lateral view hom.tif]

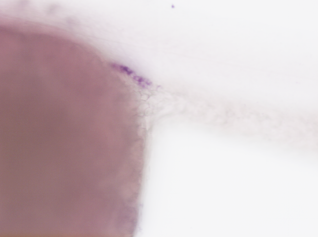

Supplement: Supplementary file 5 — Source data Fig. 2A-D F G [file 44319_2026_775_MOESM5_ESM.zip › Figure 2A-D F G/Figure 2A/ddx4 24 hpf lateral view WT.tif]

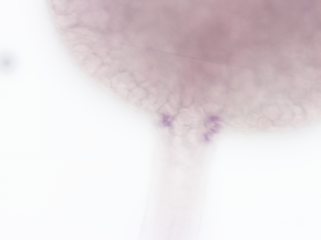

Supplement: Supplementary file 5 — Source data Fig. 2A-D F G [file 44319_2026_775_MOESM5_ESM.zip › Figure 2A-D F G/Figure 2A/ddx4 24 hpf top view het.tif]

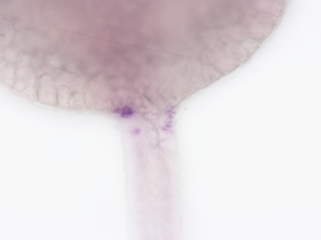

Supplement: Supplementary file 5 — Source data Fig. 2A-D F G [file 44319_2026_775_MOESM5_ESM.zip › Figure 2A-D F G/Figure 2A/ddx4 24 hpf top view hom.tif]

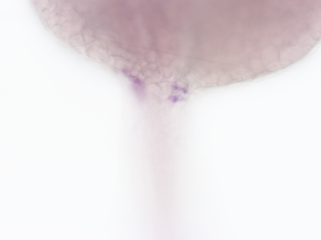

Supplement: Supplementary file 5 — Source data Fig. 2A-D F G [file 44319_2026_775_MOESM5_ESM.zip › Figure 2A-D F G/Figure 2A/ddx4 24 hpf top view WT.tif]

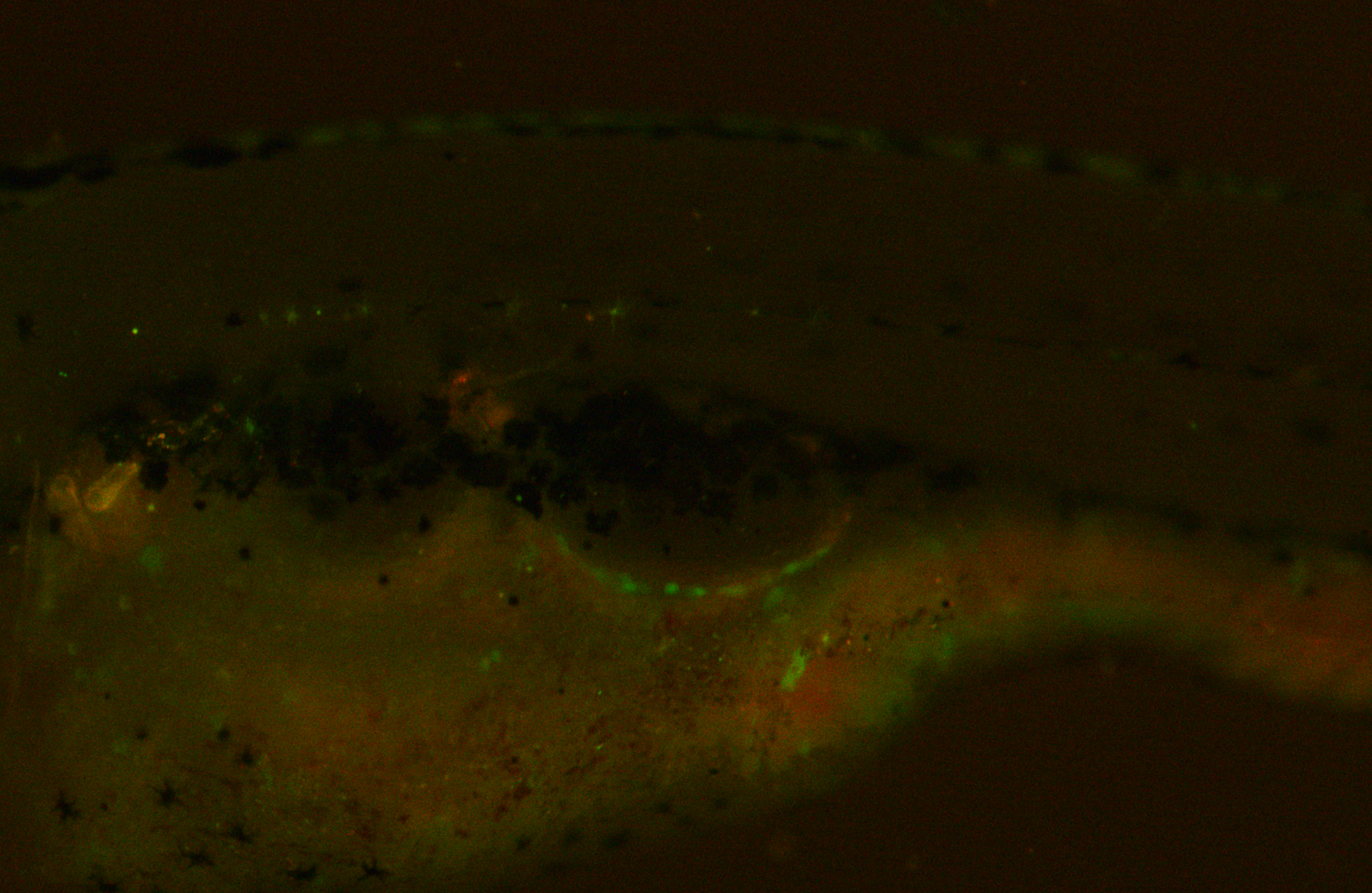

Supplement: Supplementary file 5 — Source data Fig. 2A-D F G [file 44319_2026_775_MOESM5_ESM.zip › Figure 2A-D F G/Figure 2C/+7 line 19 dpf-hom.tif]

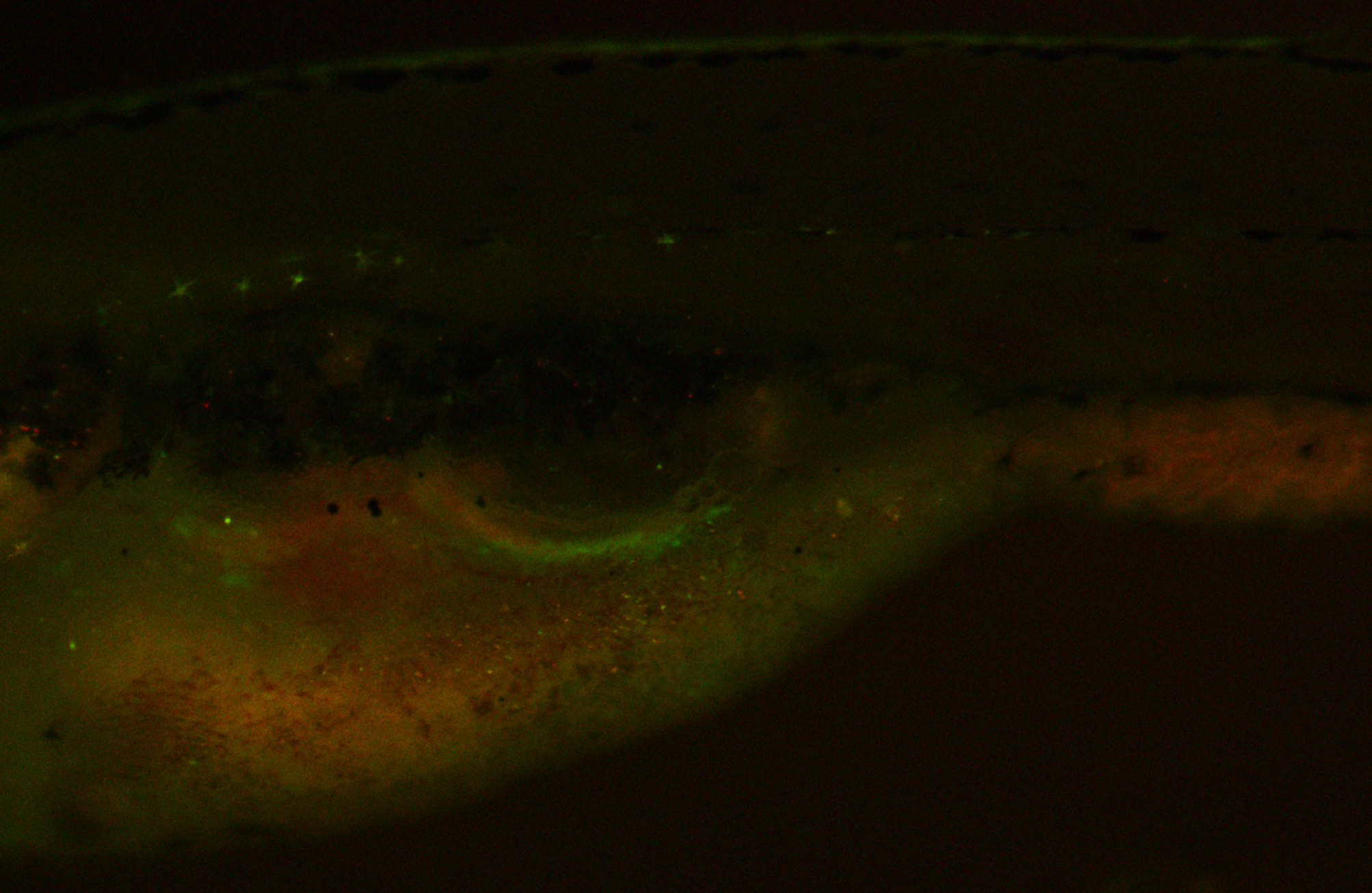

Supplement: Supplementary file 5 — Source data Fig. 2A-D F G [file 44319_2026_775_MOESM5_ESM.zip › Figure 2A-D F G/Figure 2C/+7 line 19 dpf-WT.tif]

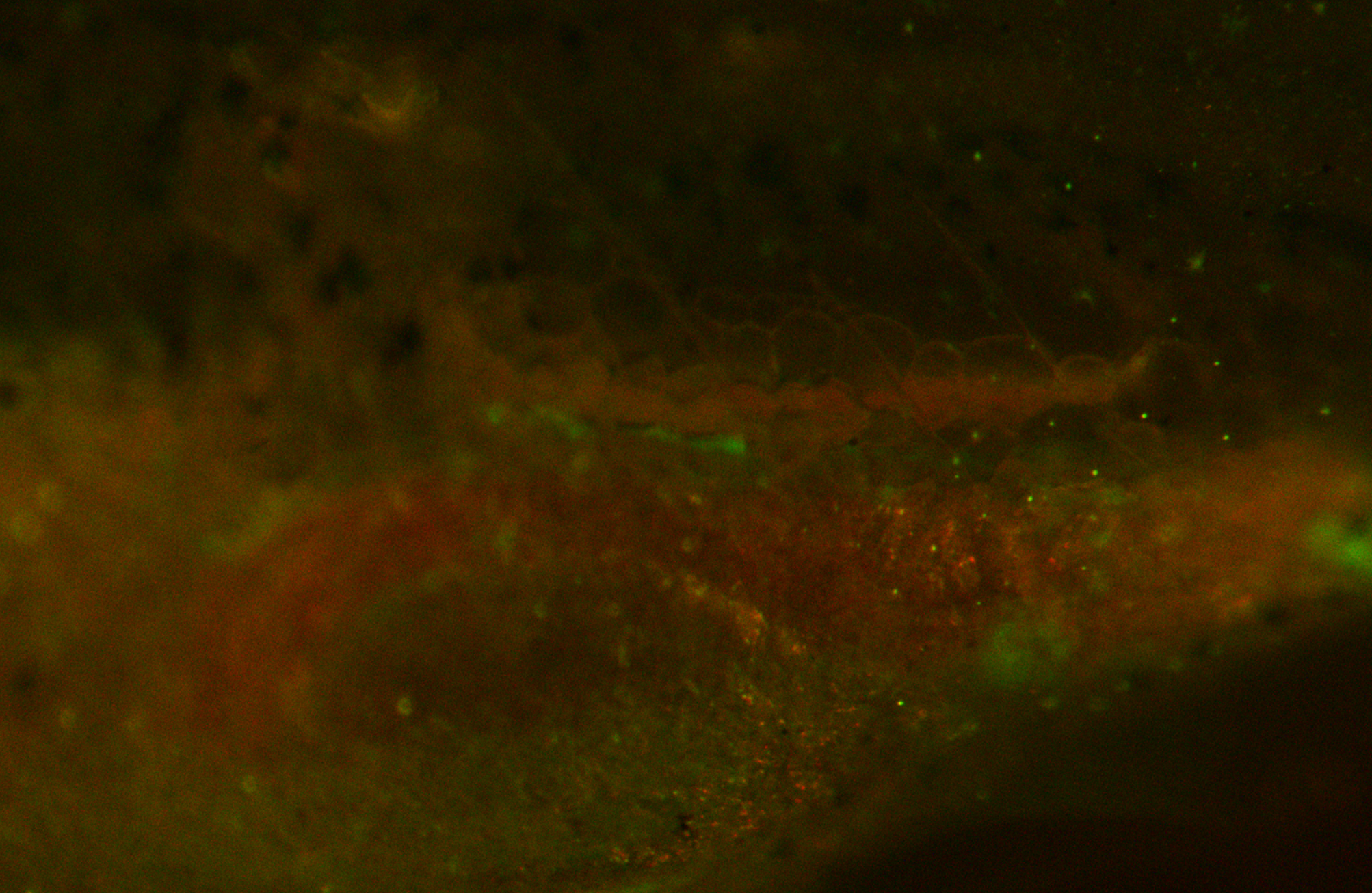

Supplement: Supplementary file 5 — Source data Fig. 2A-D F G [file 44319_2026_775_MOESM5_ESM.zip › Figure 2A-D F G/Figure 2C/+7 line 25 dpf-hom.tif]

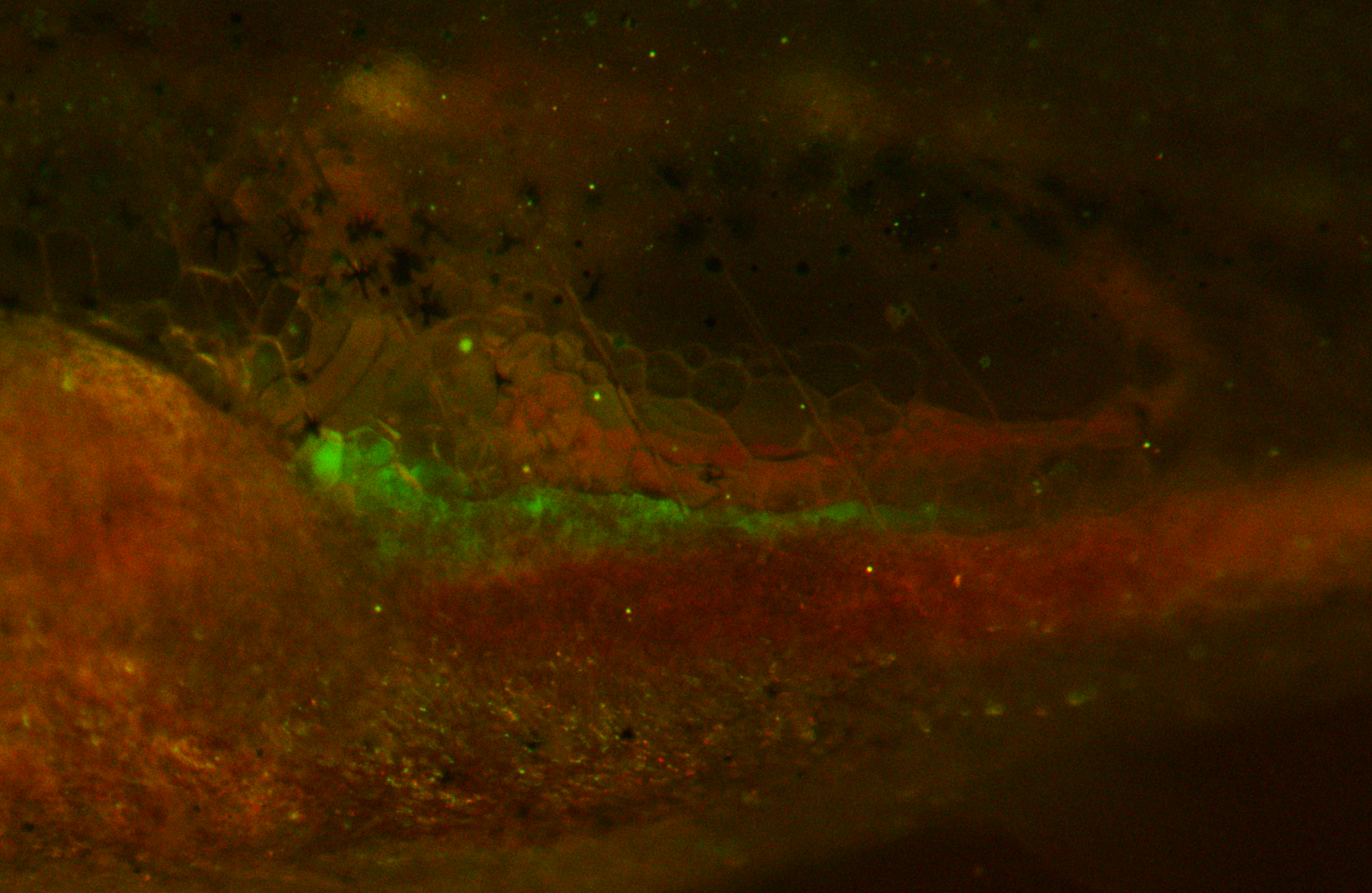

Supplement: Supplementary file 5 — Source data Fig. 2A-D F G [file 44319_2026_775_MOESM5_ESM.zip › Figure 2A-D F G/Figure 2C/+7 line 25 dpf-WT female.tif]

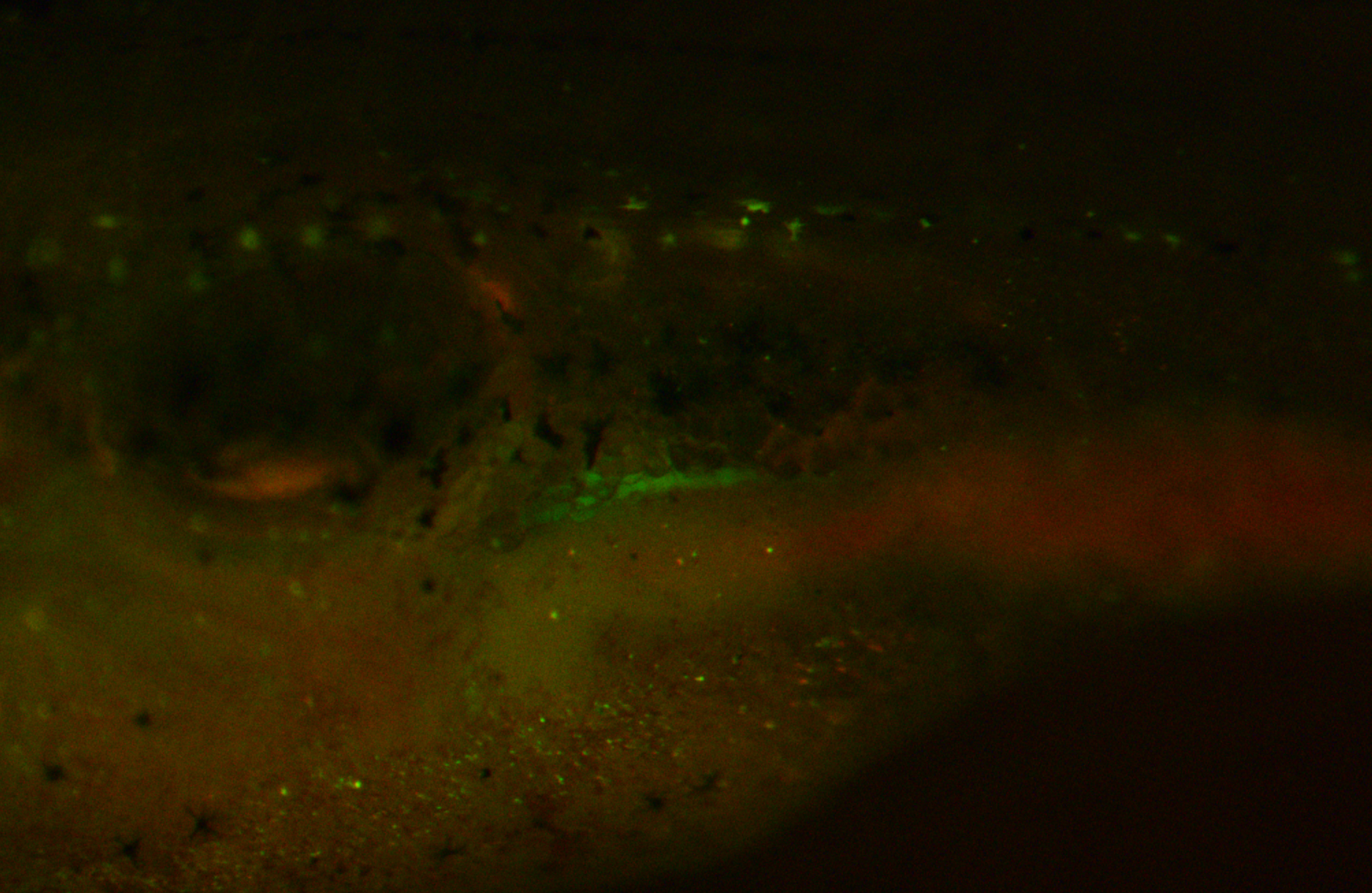

Supplement: Supplementary file 5 — Source data Fig. 2A-D F G [file 44319_2026_775_MOESM5_ESM.zip › Figure 2A-D F G/Figure 2C/+7 line 25 dpf-WT male.tif]

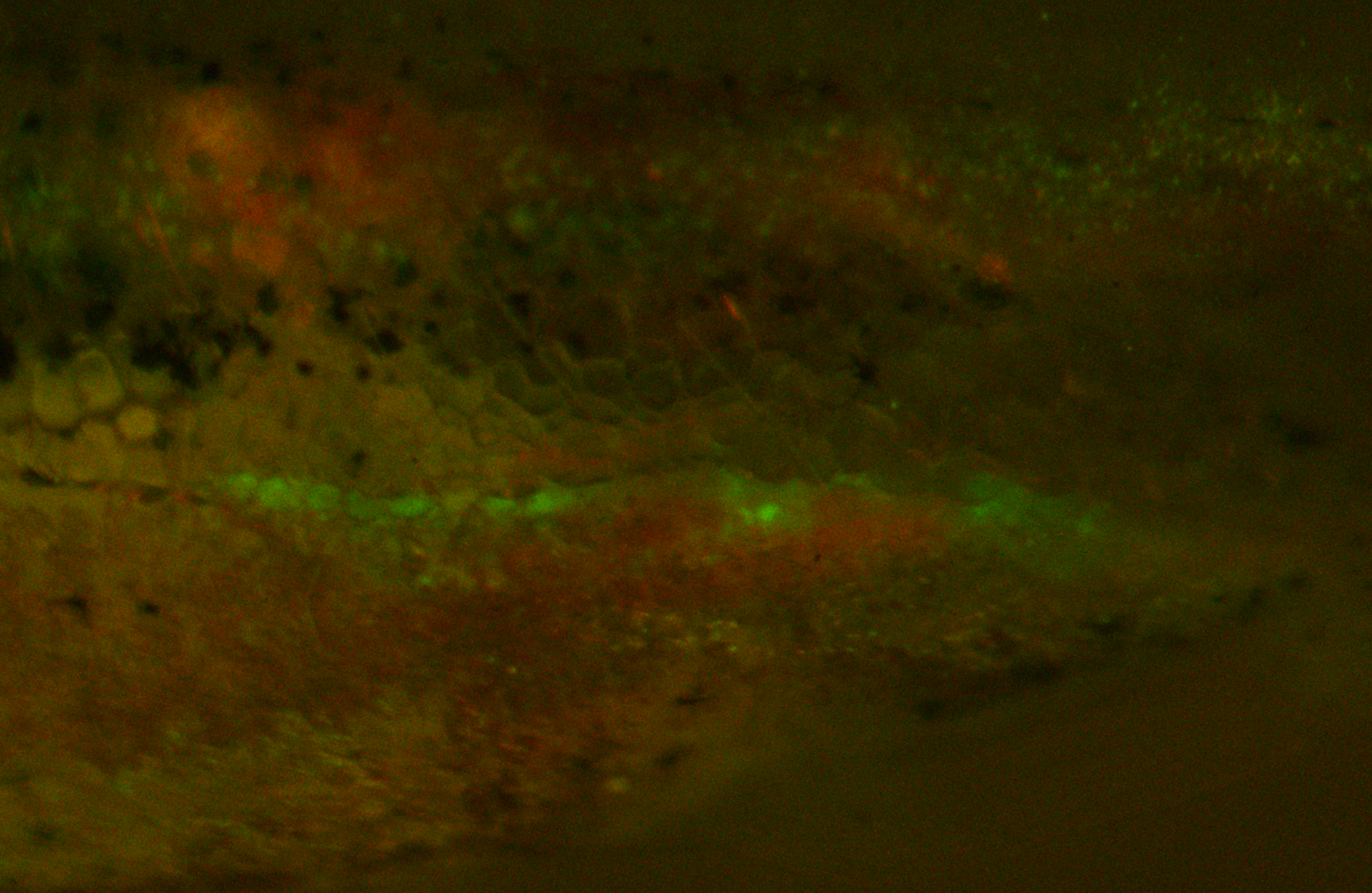

Supplement: Supplementary file 5 — Source data Fig. 2A-D F G [file 44319_2026_775_MOESM5_ESM.zip › Figure 2A-D F G/Figure 2C/+7 line 33 dpf-hom.tif]

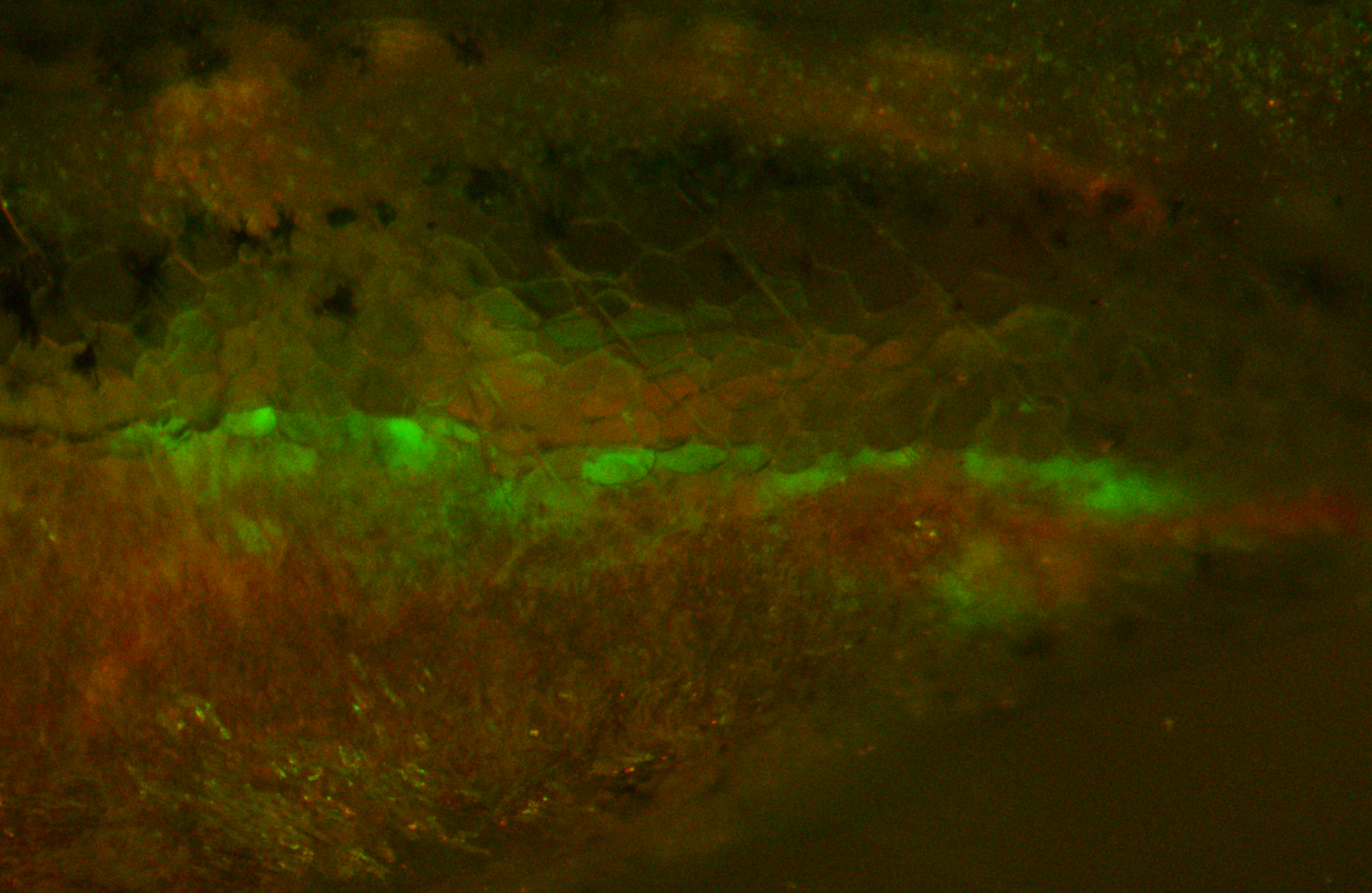

Supplement: Supplementary file 5 — Source data Fig. 2A-D F G [file 44319_2026_775_MOESM5_ESM.zip › Figure 2A-D F G/Figure 2C/+7 line 33 dpf-WT female.tif]

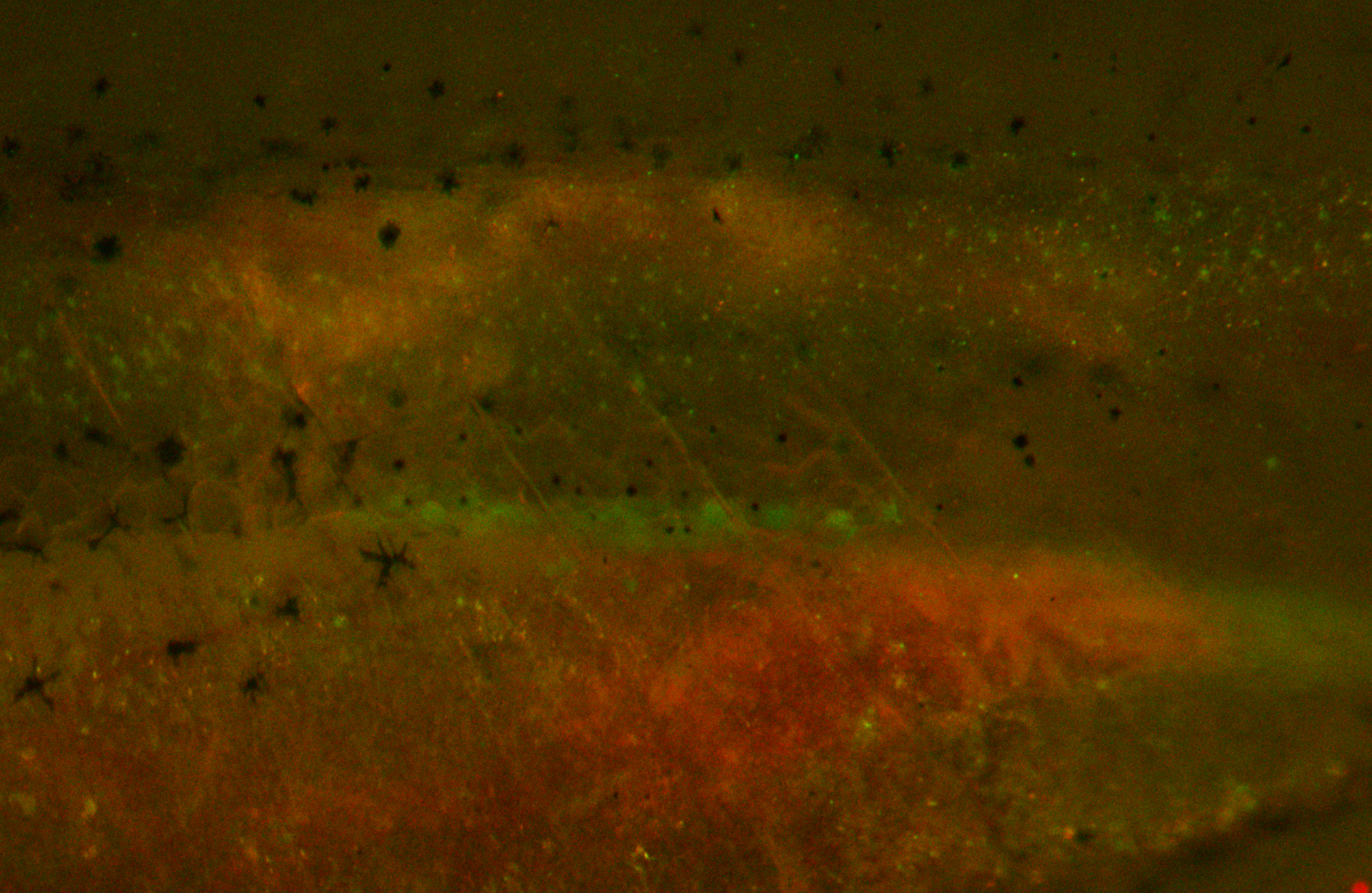

Supplement: Supplementary file 5 — Source data Fig. 2A-D F G [file 44319_2026_775_MOESM5_ESM.zip › Figure 2A-D F G/Figure 2C/+7 line 33 dpf-WT male.tif]

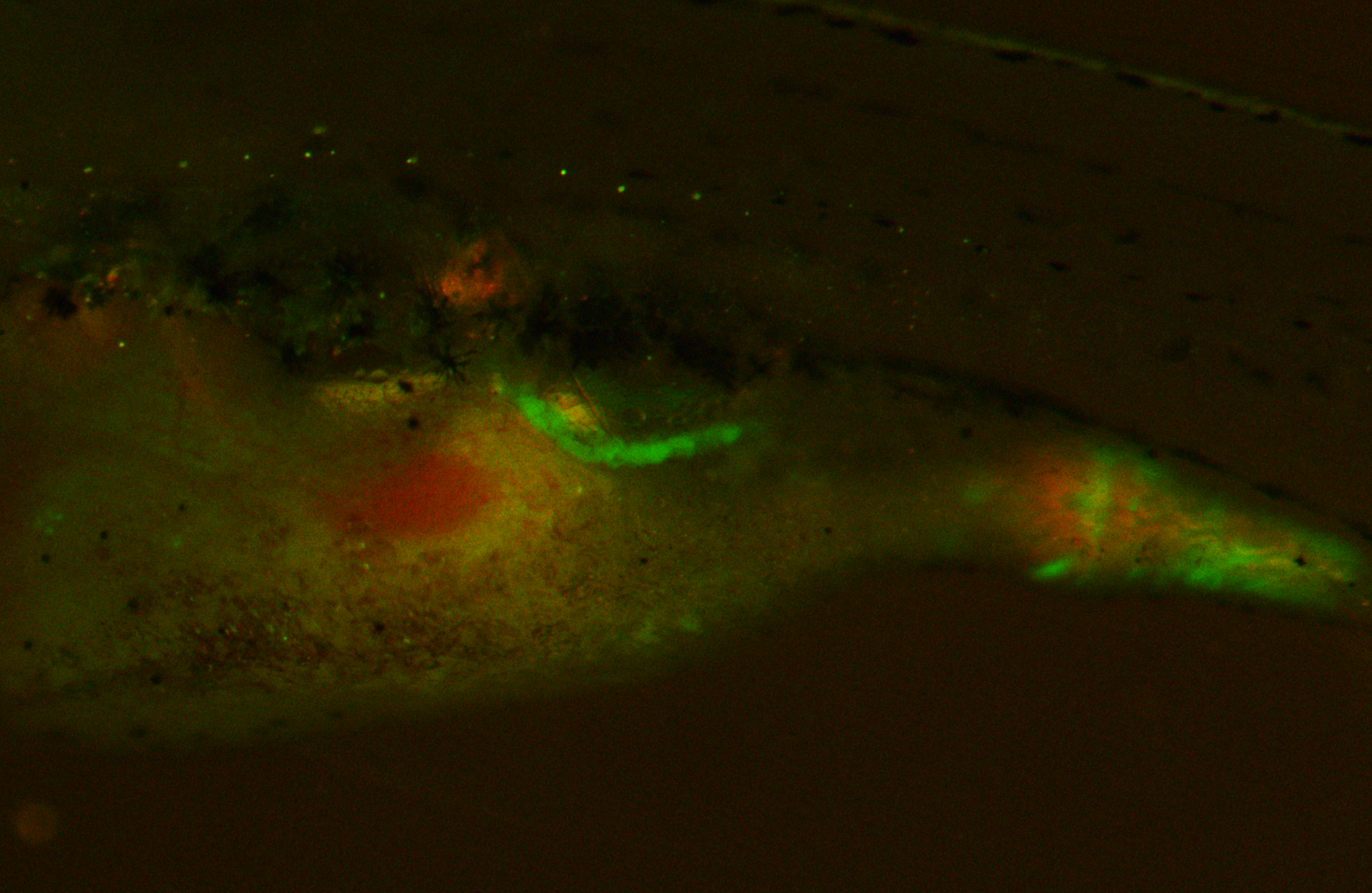

Supplement: Supplementary file 5 — Source data Fig. 2A-D F G [file 44319_2026_775_MOESM5_ESM.zip › Figure 2A-D F G/Figure 2C/Δ7 line 19 dpf-hom.tif]

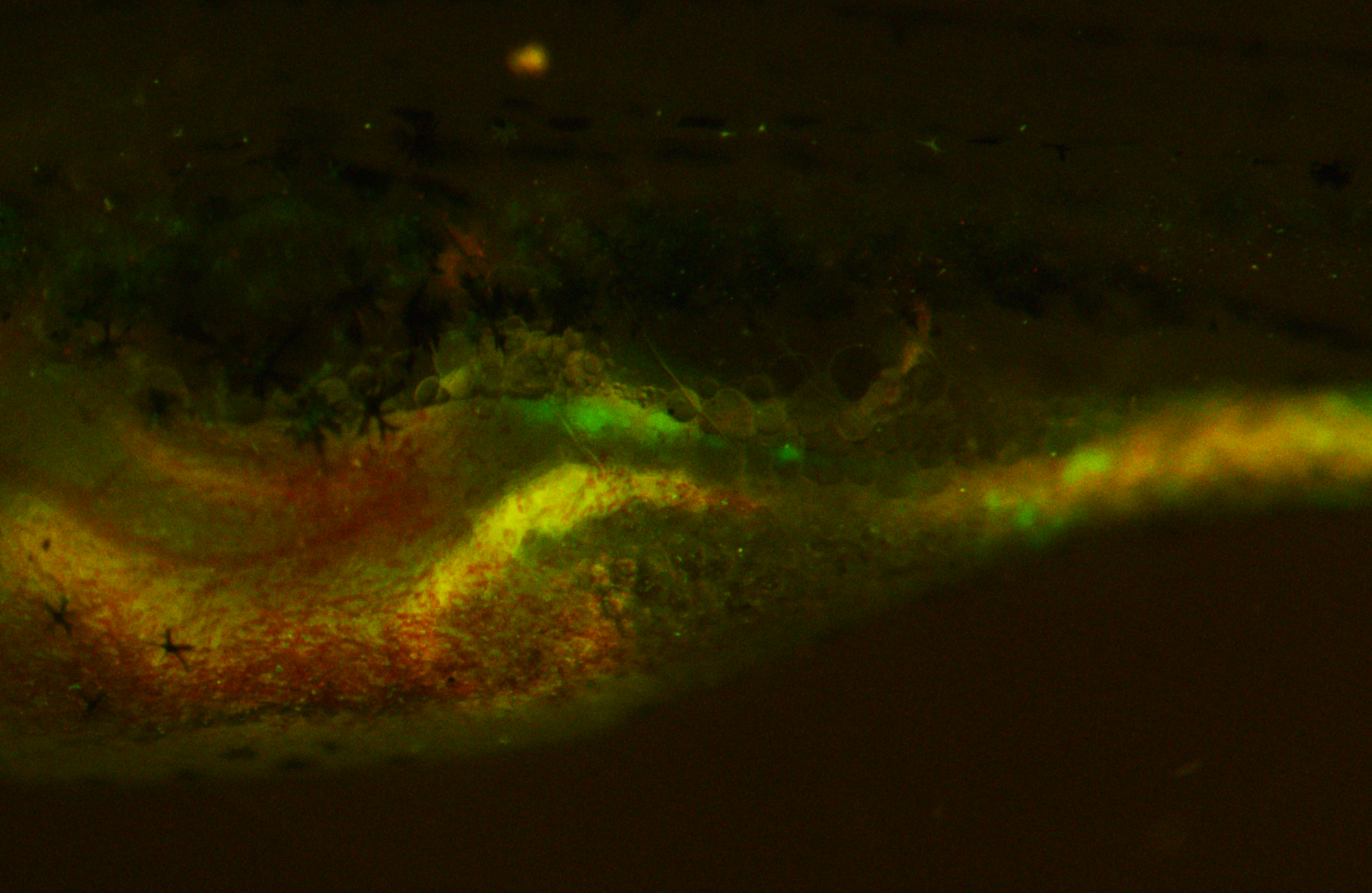

Supplement: Supplementary file 5 — Source data Fig. 2A-D F G [file 44319_2026_775_MOESM5_ESM.zip › Figure 2A-D F G/Figure 2C/Δ7 line 19 dpf-WT.tif]

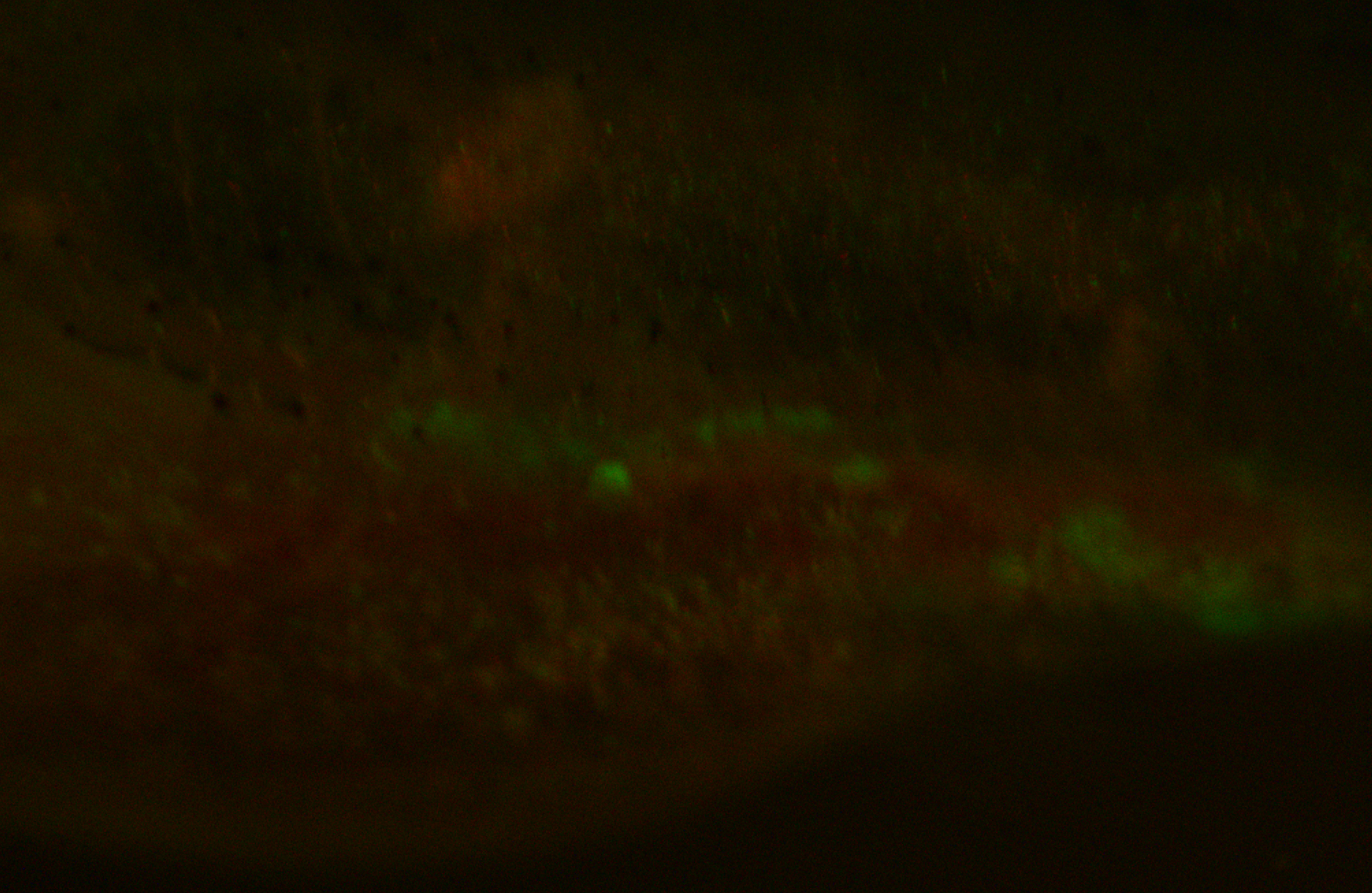

Supplement: Supplementary file 5 — Source data Fig. 2A-D F G [file 44319_2026_775_MOESM5_ESM.zip › Figure 2A-D F G/Figure 2C/Δ7 line 25 dpf-hom.tif]

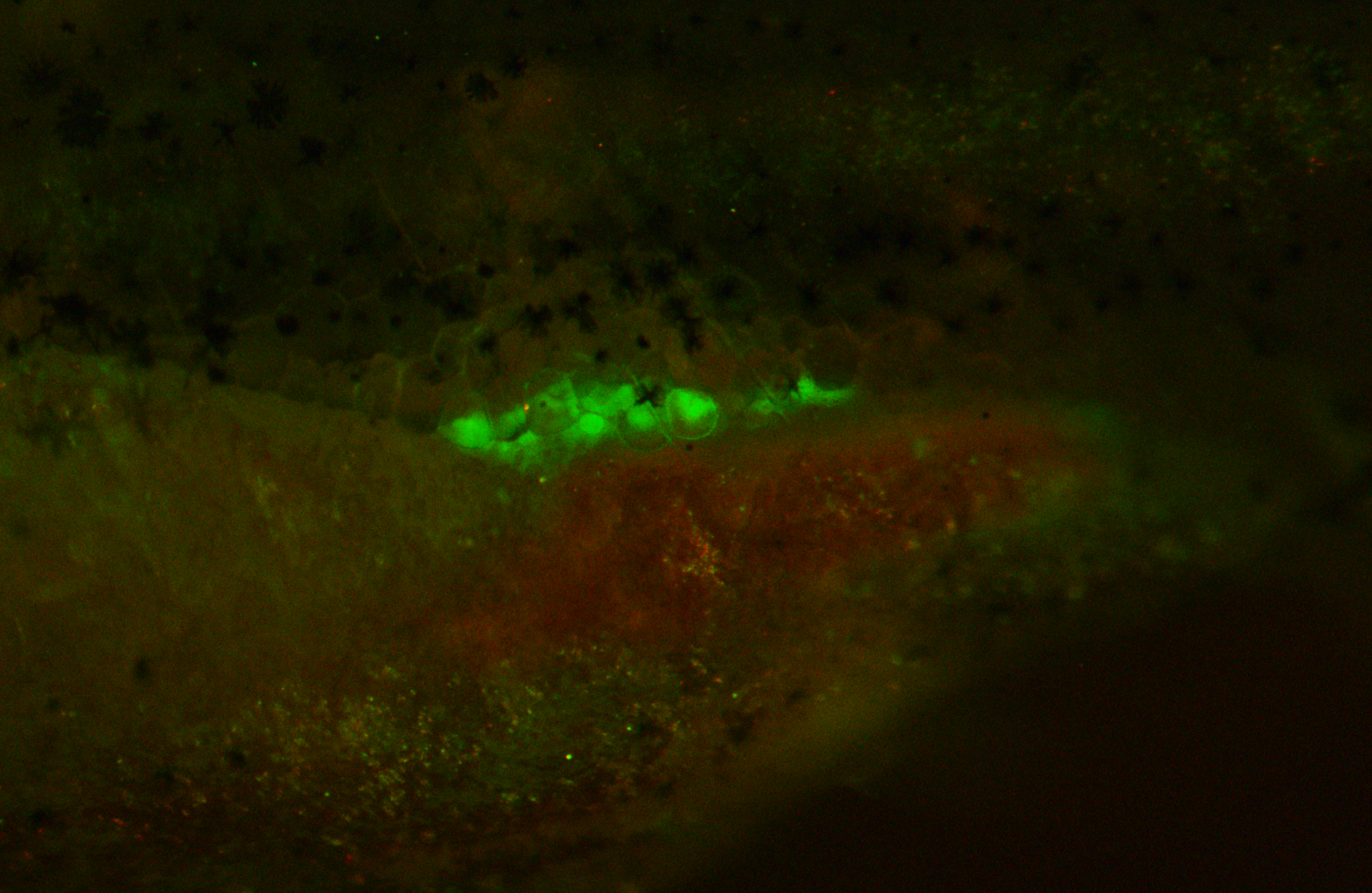

Supplement: Supplementary file 5 — Source data Fig. 2A-D F G [file 44319_2026_775_MOESM5_ESM.zip › Figure 2A-D F G/Figure 2C/Δ7 line 25 dpf-WT female.tif]

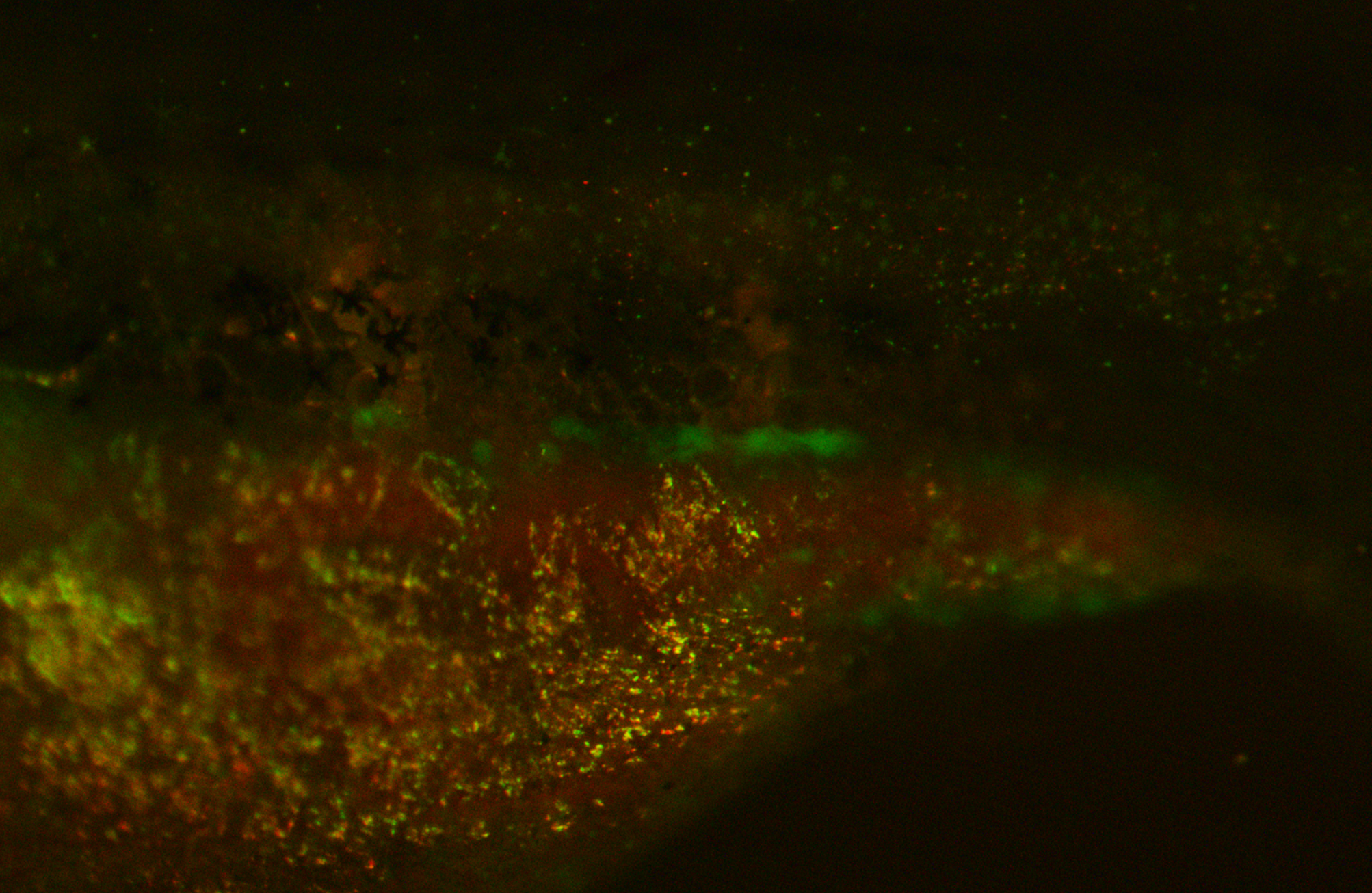

Supplement: Supplementary file 5 — Source data Fig. 2A-D F G [file 44319_2026_775_MOESM5_ESM.zip › Figure 2A-D F G/Figure 2C/Δ7 line 25 dpf-WT male.tif]

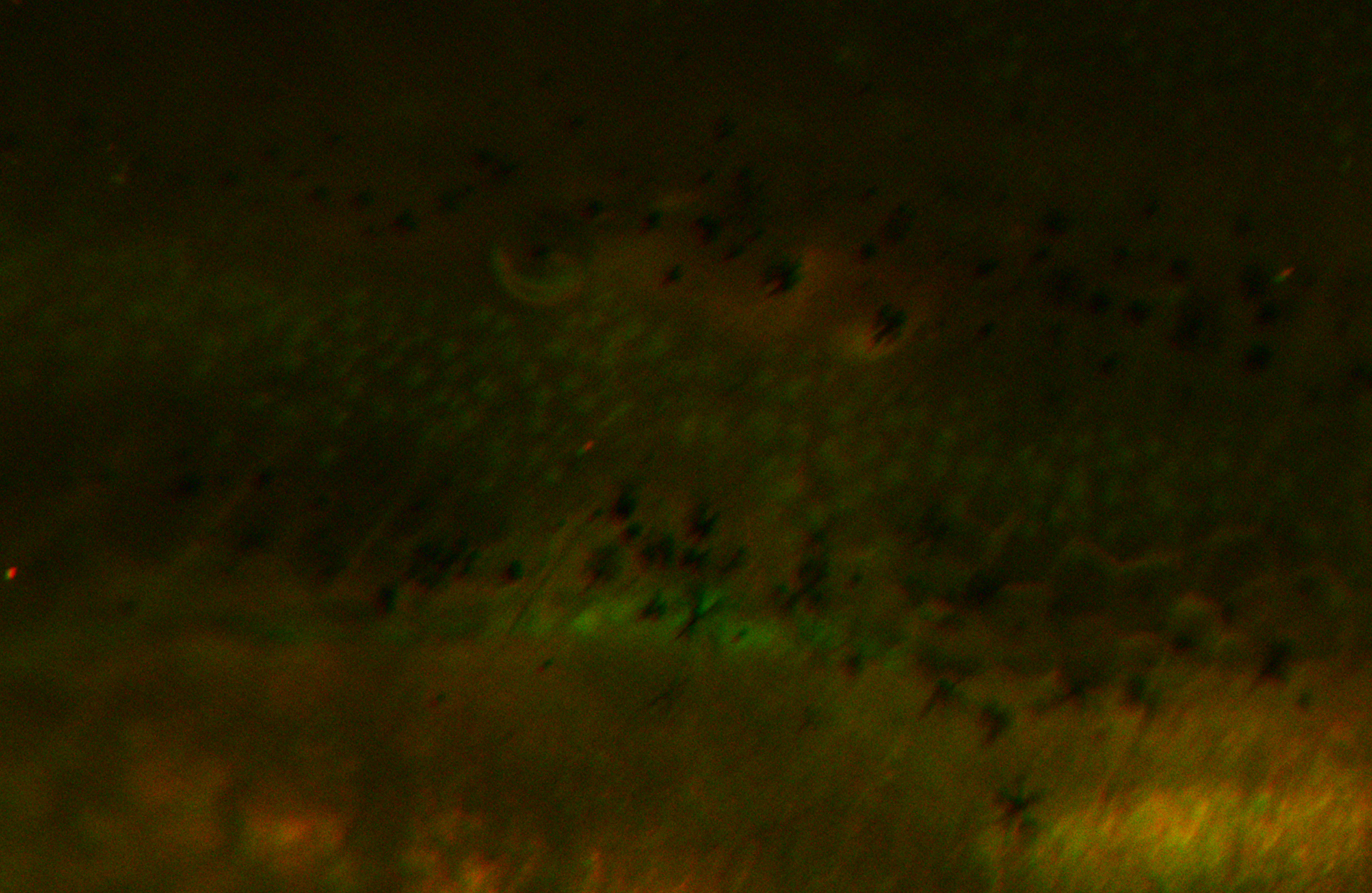

Supplement: Supplementary file 5 — Source data Fig. 2A-D F G [file 44319_2026_775_MOESM5_ESM.zip › Figure 2A-D F G/Figure 2C/Δ7 line 33 dpf-hom.tif]

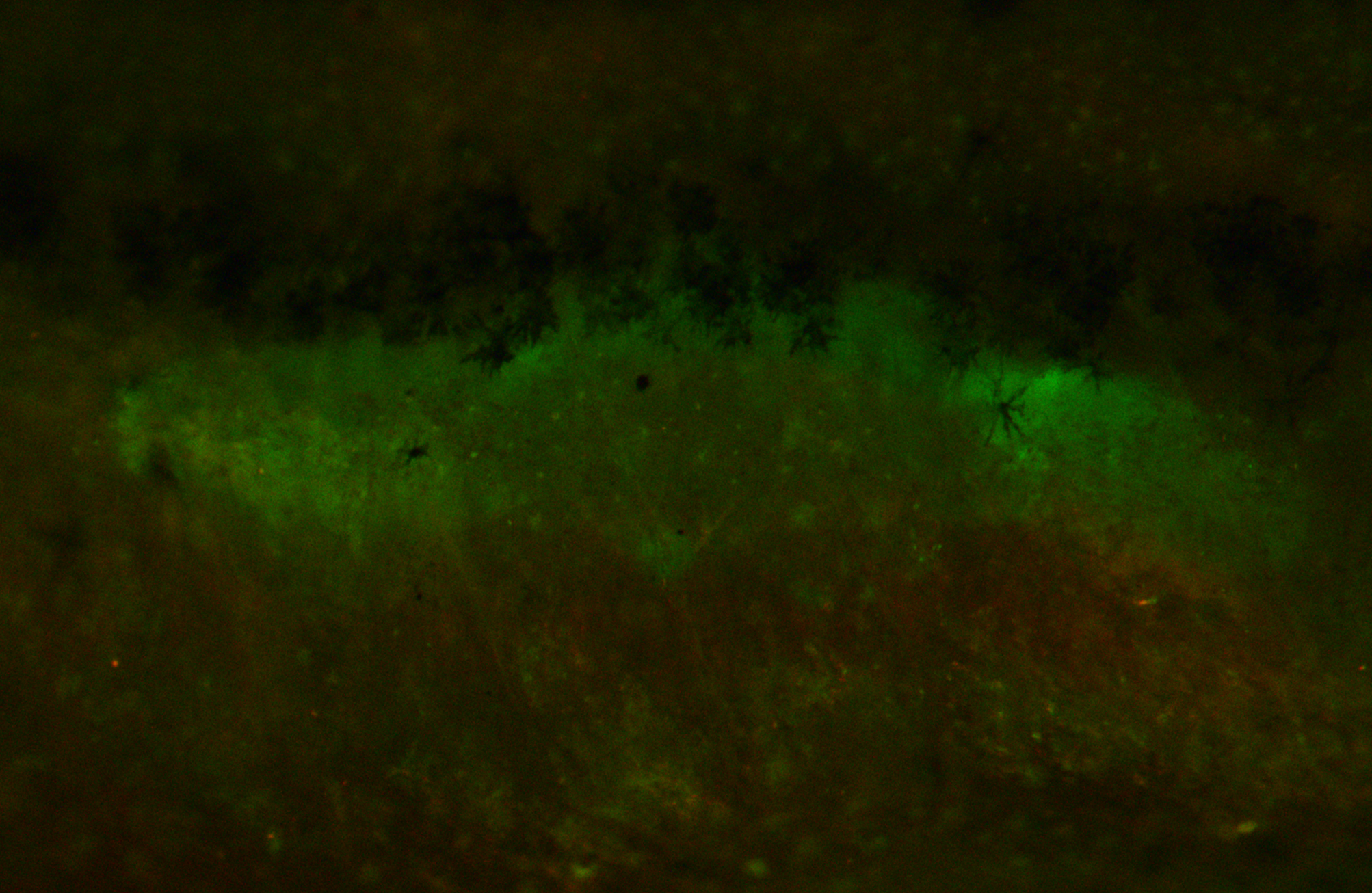

Supplement: Supplementary file 5 — Source data Fig. 2A-D F G [file 44319_2026_775_MOESM5_ESM.zip › Figure 2A-D F G/Figure 2C/Δ7 line 33 dpf-WT female.tif]

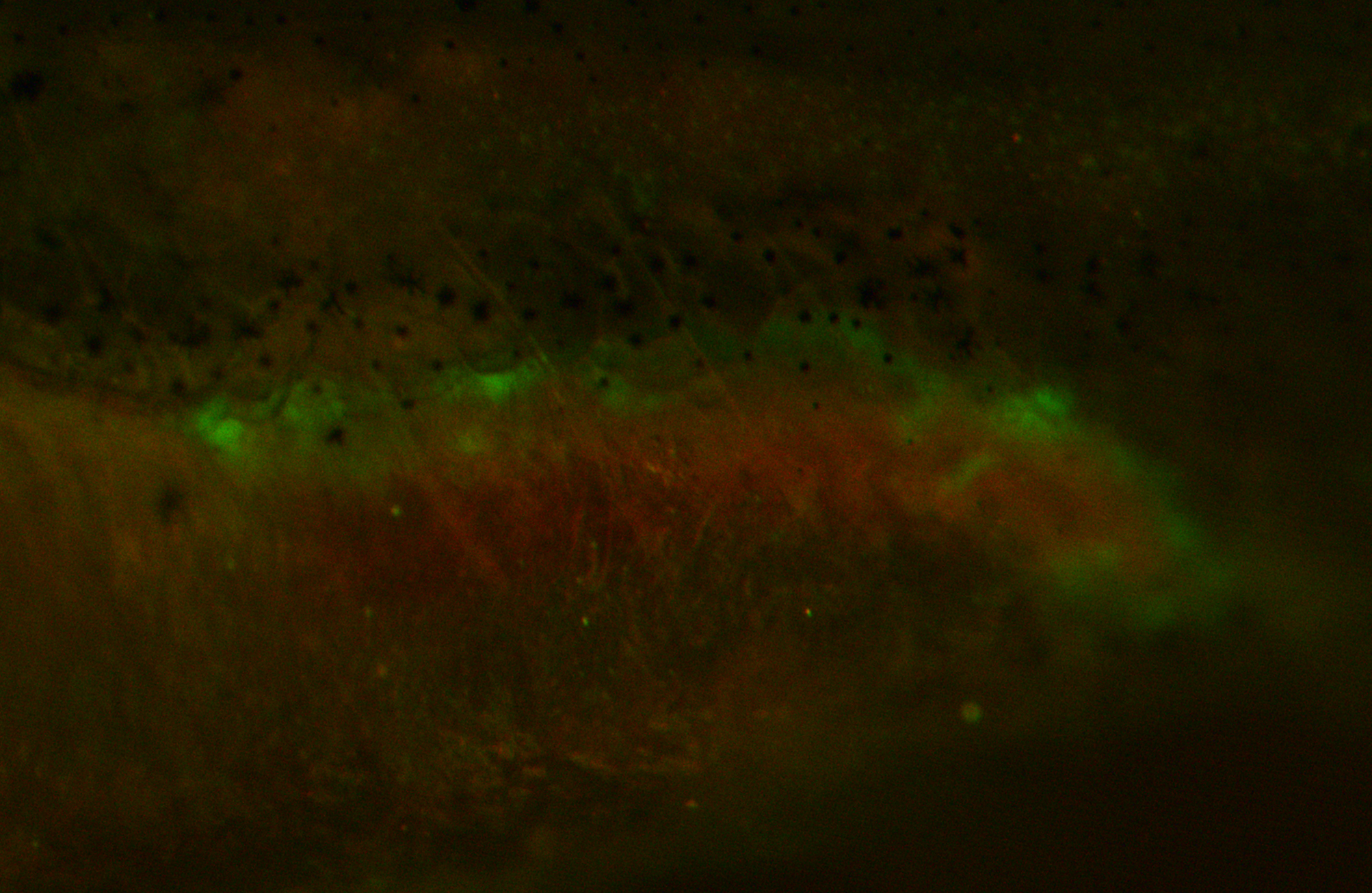

Supplement: Supplementary file 5 — Source data Fig. 2A-D F G [file 44319_2026_775_MOESM5_ESM.zip › Figure 2A-D F G/Figure 2C/Δ7 line 33 dpf-WT male.tif]

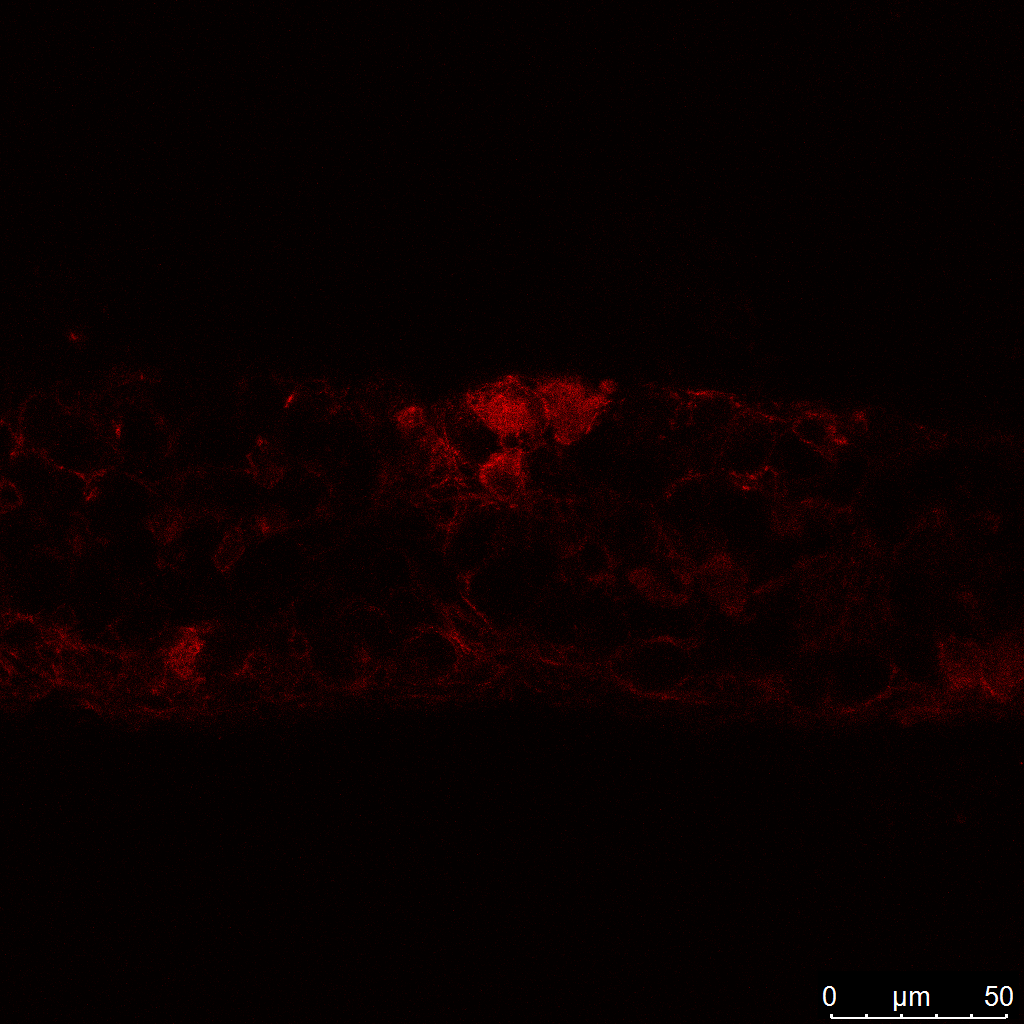

Supplement: Supplementary file 5 — Source data Fig. 2A-D F G [file 44319_2026_775_MOESM5_ESM.zip › Figure 2A-D F G/Figure 2F/Cleaved Caspase-3 hom.tif]

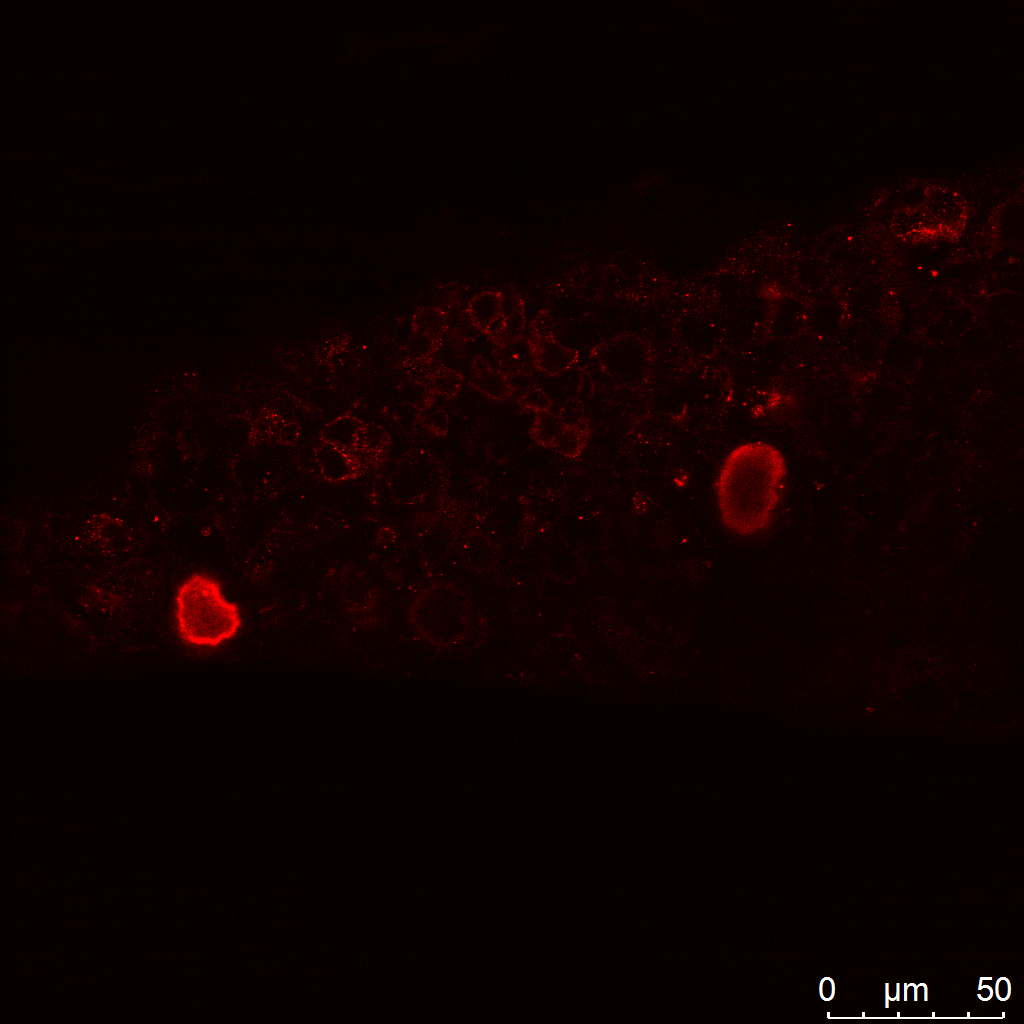

Supplement: Supplementary file 5 — Source data Fig. 2A-D F G [file 44319_2026_775_MOESM5_ESM.zip › Figure 2A-D F G/Figure 2F/Cleaved Caspase-3 WT.tif]

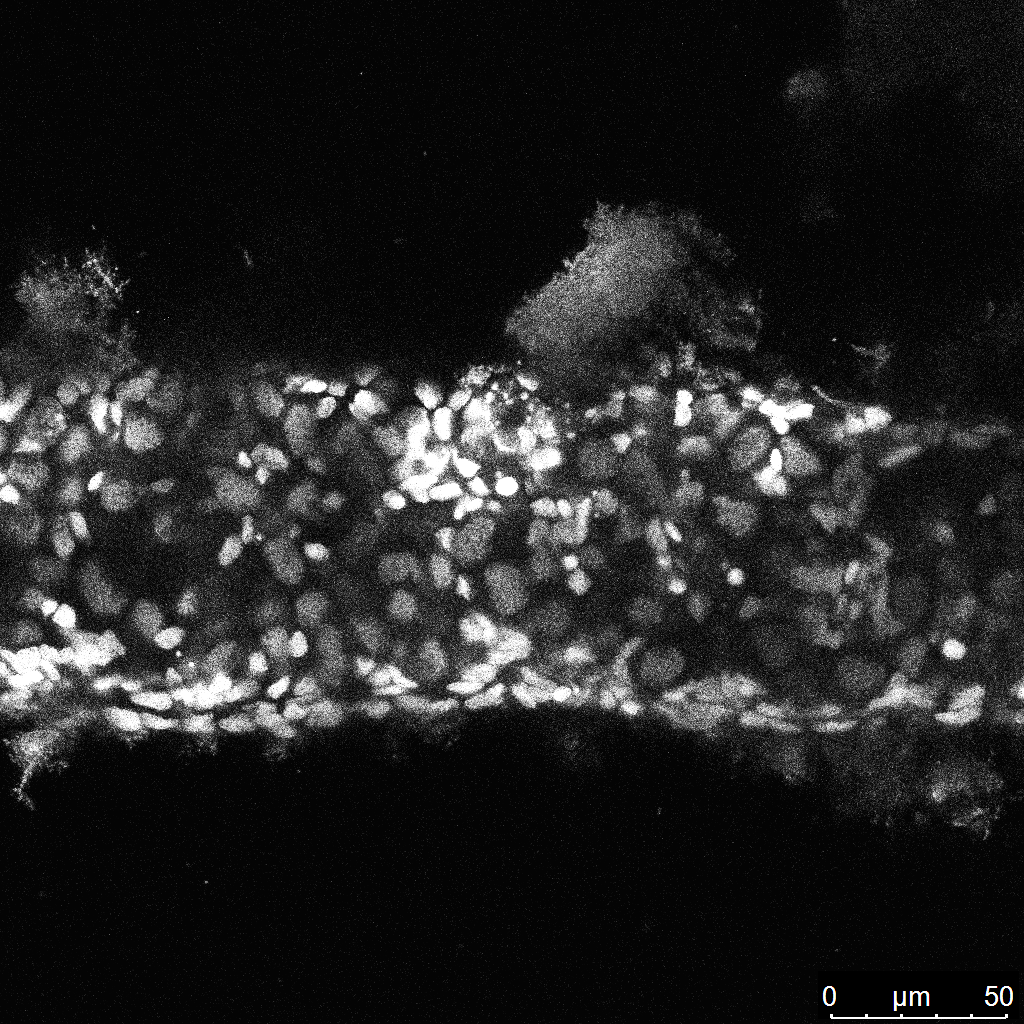

Supplement: Supplementary file 5 — Source data Fig. 2A-D F G [file 44319_2026_775_MOESM5_ESM.zip › Figure 2A-D F G/Figure 2F/DAPI hom.tif]

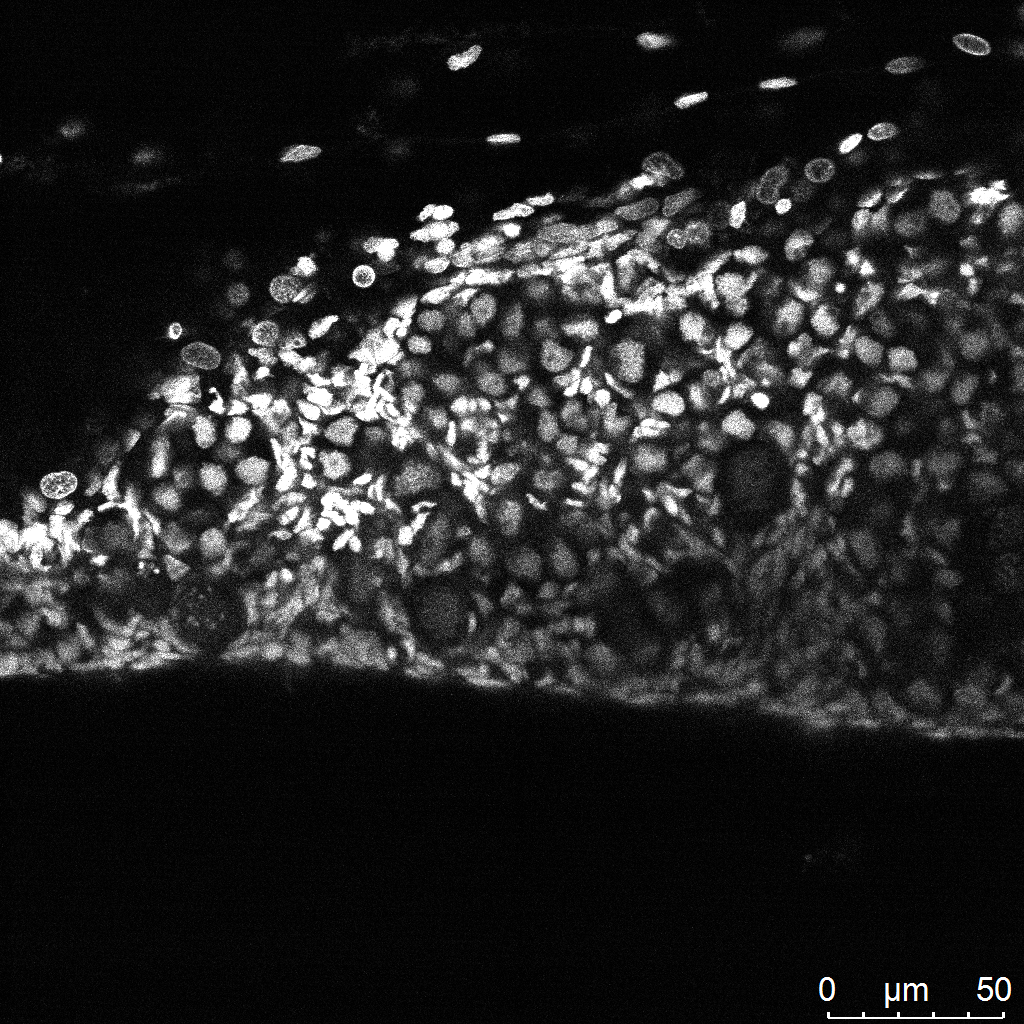

Supplement: Supplementary file 5 — Source data Fig. 2A-D F G [file 44319_2026_775_MOESM5_ESM.zip › Figure 2A-D F G/Figure 2F/DAPI WT.tif]

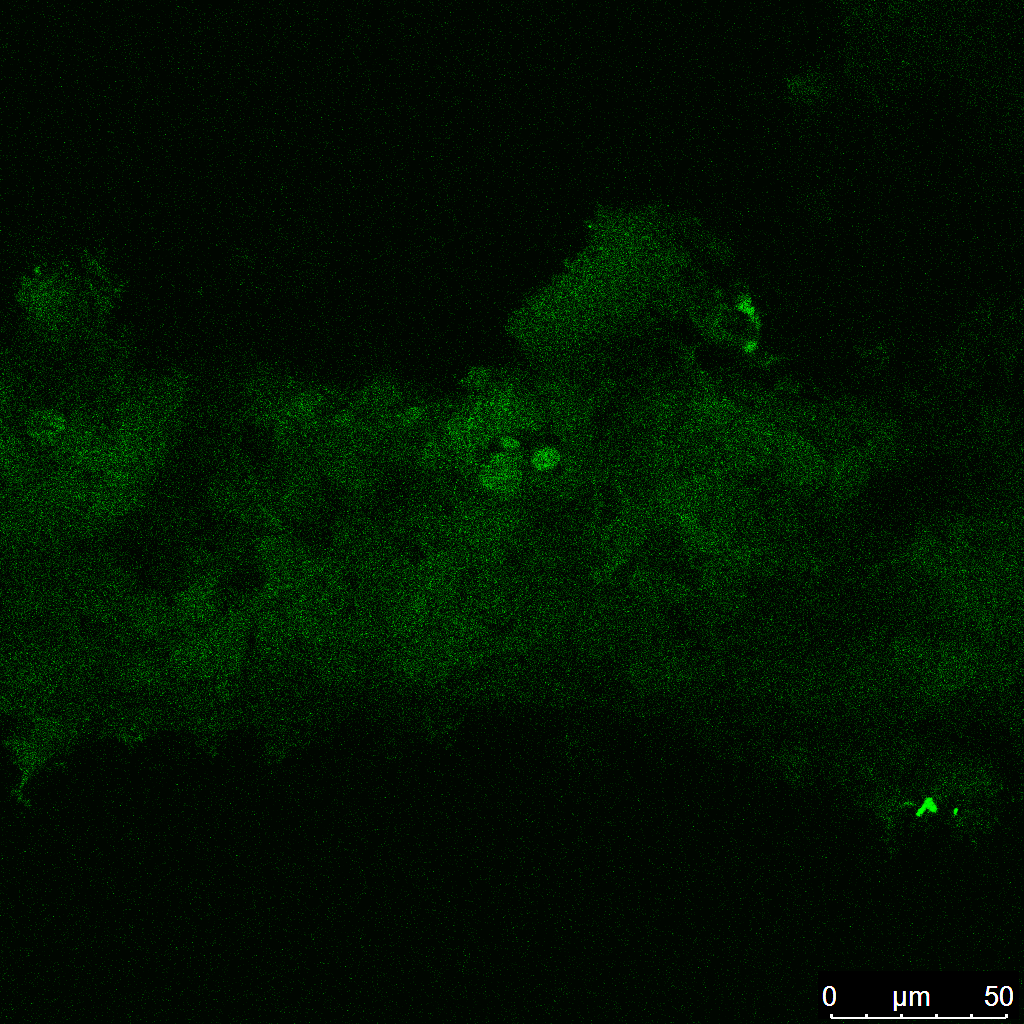

Supplement: Supplementary file 5 — Source data Fig. 2A-D F G [file 44319_2026_775_MOESM5_ESM.zip › Figure 2A-D F G/Figure 2F/EGFP hom.tif]

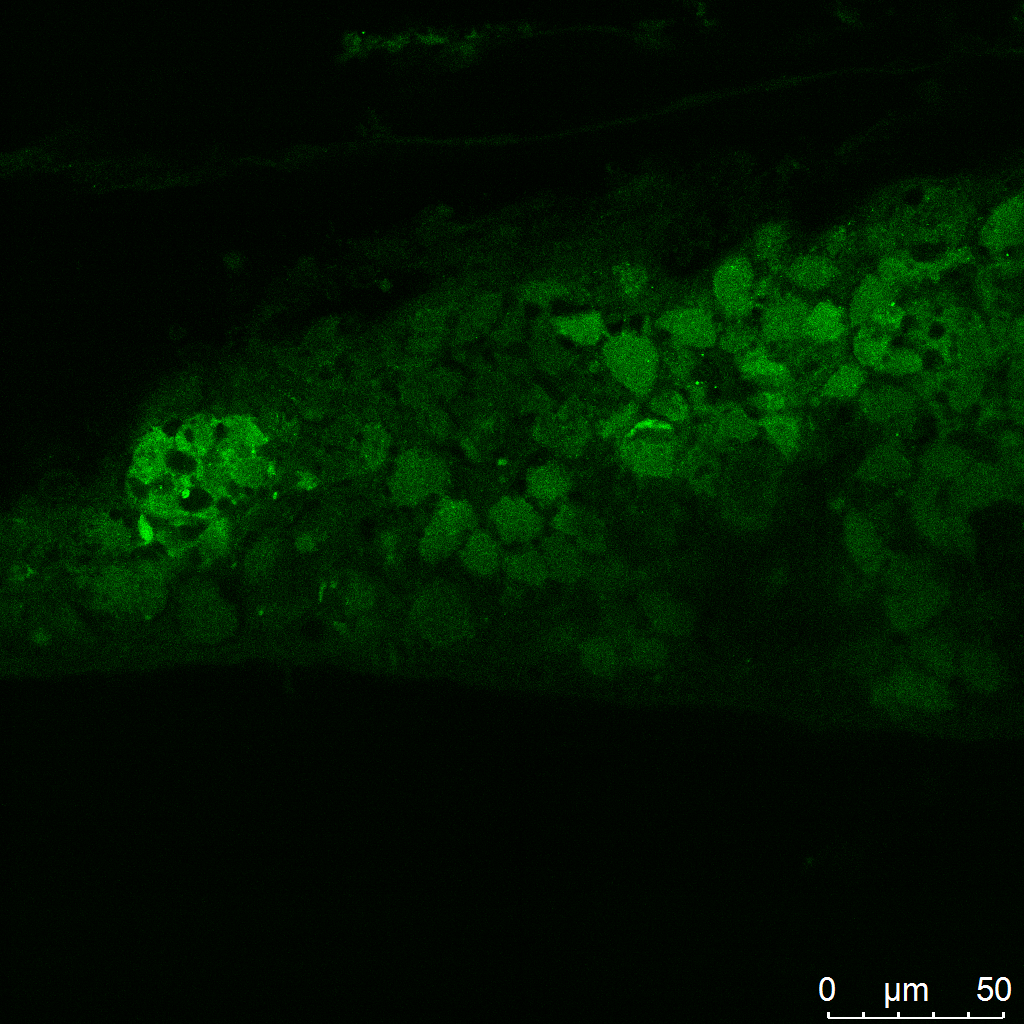

Supplement: Supplementary file 5 — Source data Fig. 2A-D F G [file 44319_2026_775_MOESM5_ESM.zip › Figure 2A-D F G/Figure 2F/EGFP WT.tif]

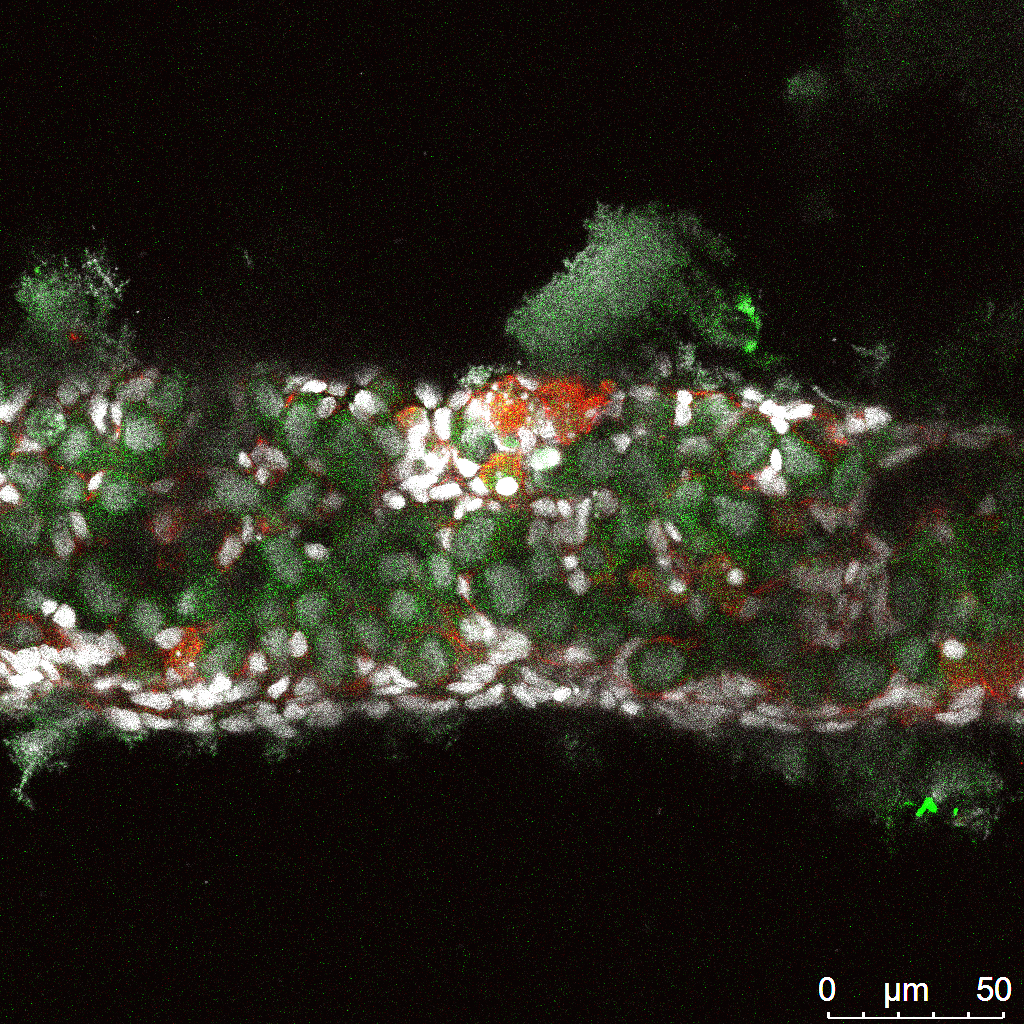

Supplement: Supplementary file 5 — Source data Fig. 2A-D F G [file 44319_2026_775_MOESM5_ESM.zip › Figure 2A-D F G/Figure 2F/Merge hom.tif]

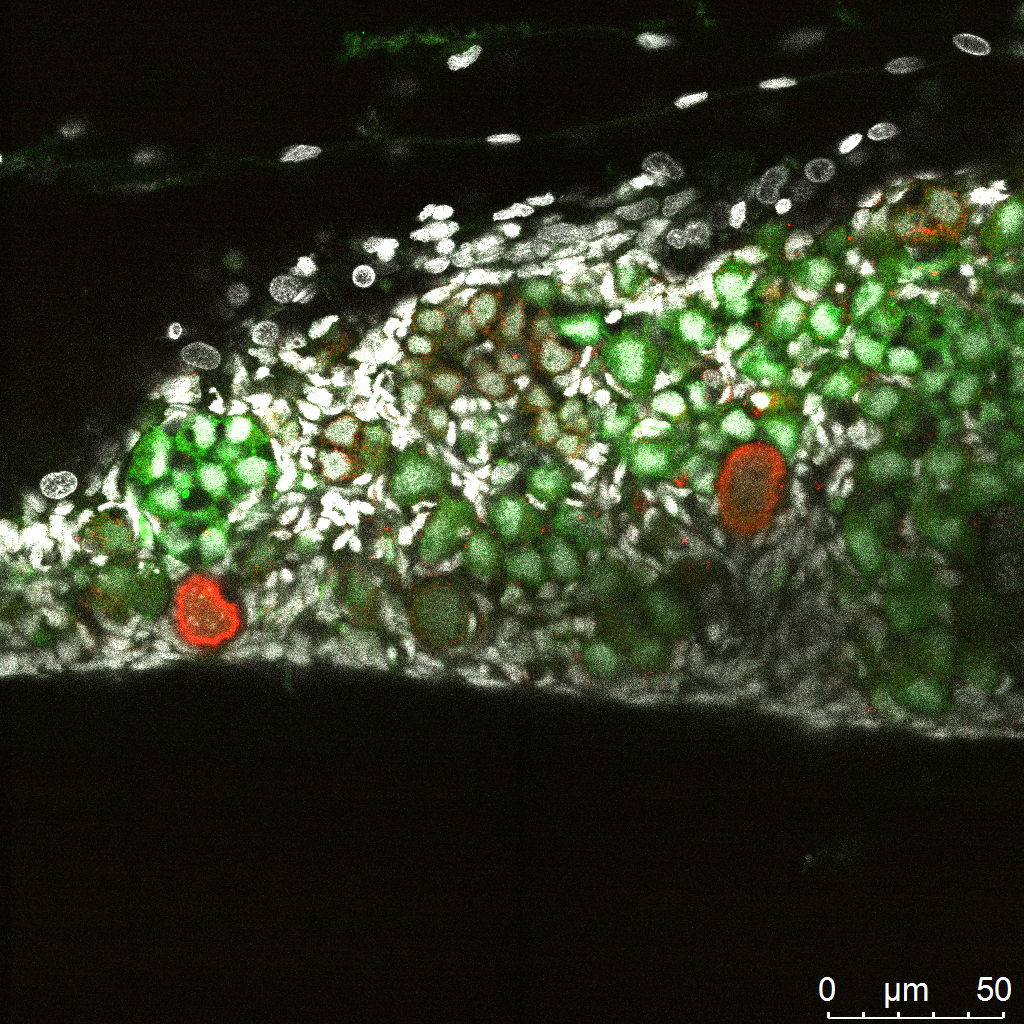

Supplement: Supplementary file 5 — Source data Fig. 2A-D F G [file 44319_2026_775_MOESM5_ESM.zip › Figure 2A-D F G/Figure 2F/Merge WT.tif]

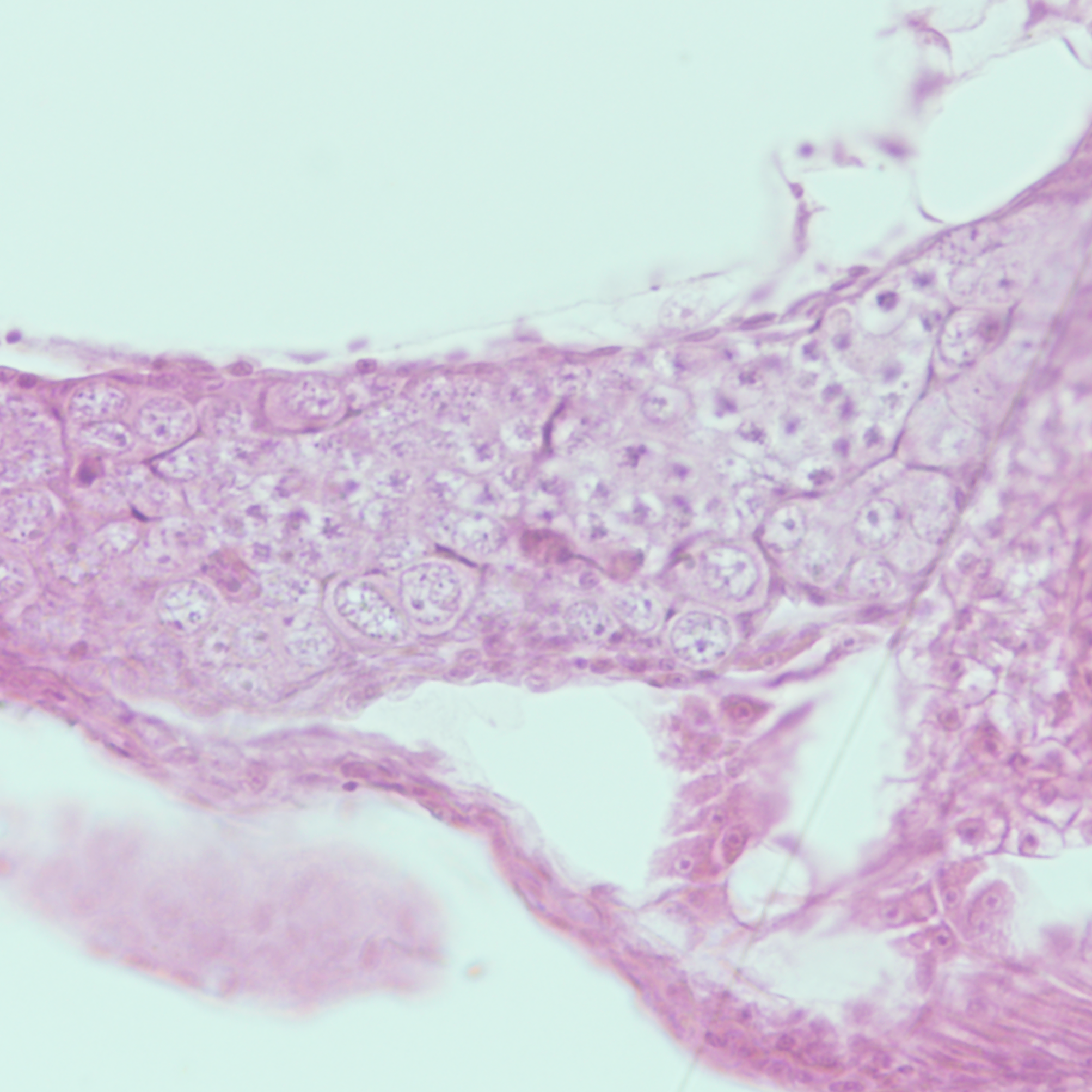

Supplement: Supplementary file 6 — Source data Fig. 2E [file 44319_2026_775_MOESM6_ESM.zip › Figure 2E/+7 line 19 dpf-hom.tif]

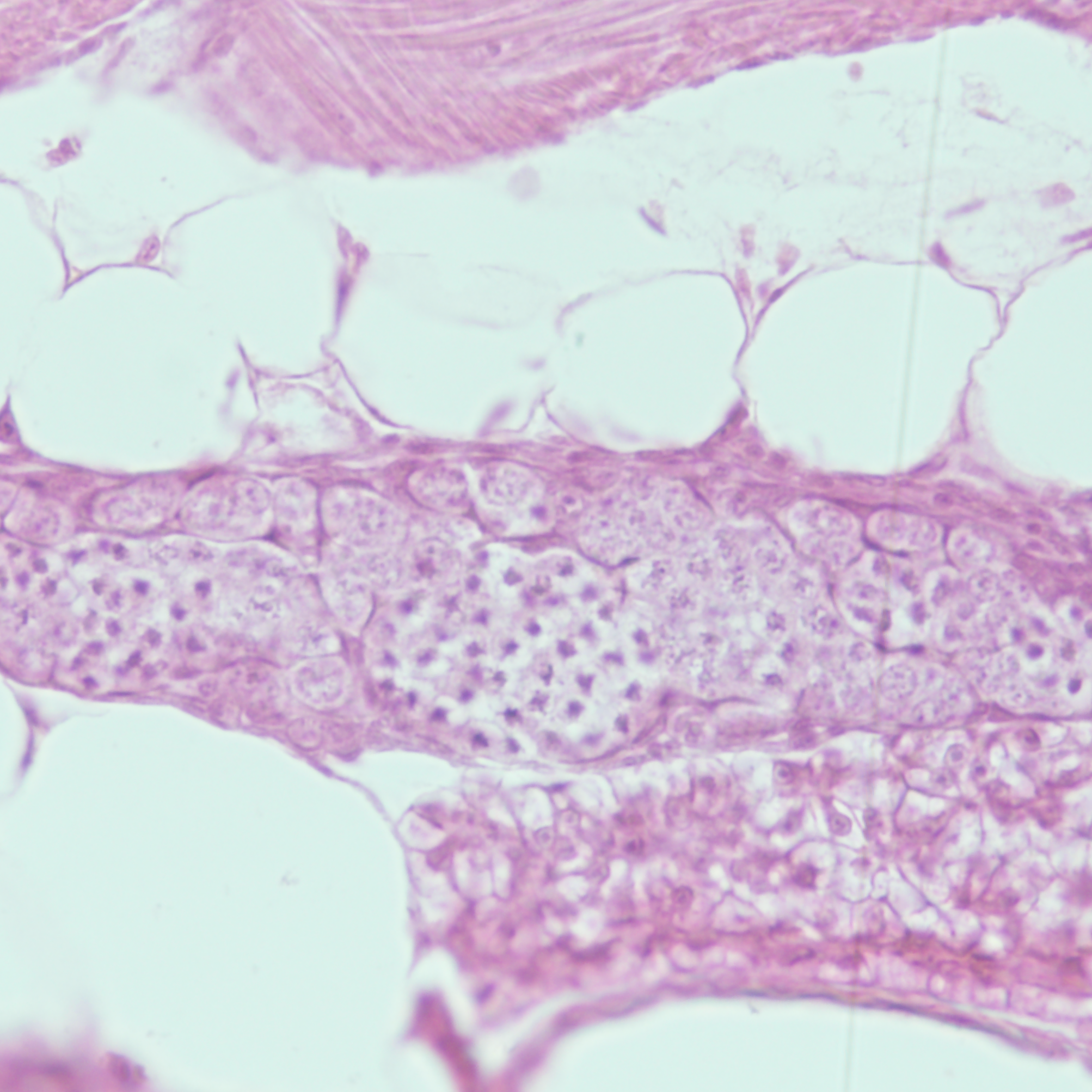

Supplement: Supplementary file 6 — Source data Fig. 2E [file 44319_2026_775_MOESM6_ESM.zip › Figure 2E/+7 line 19 dpf-WT.tif]

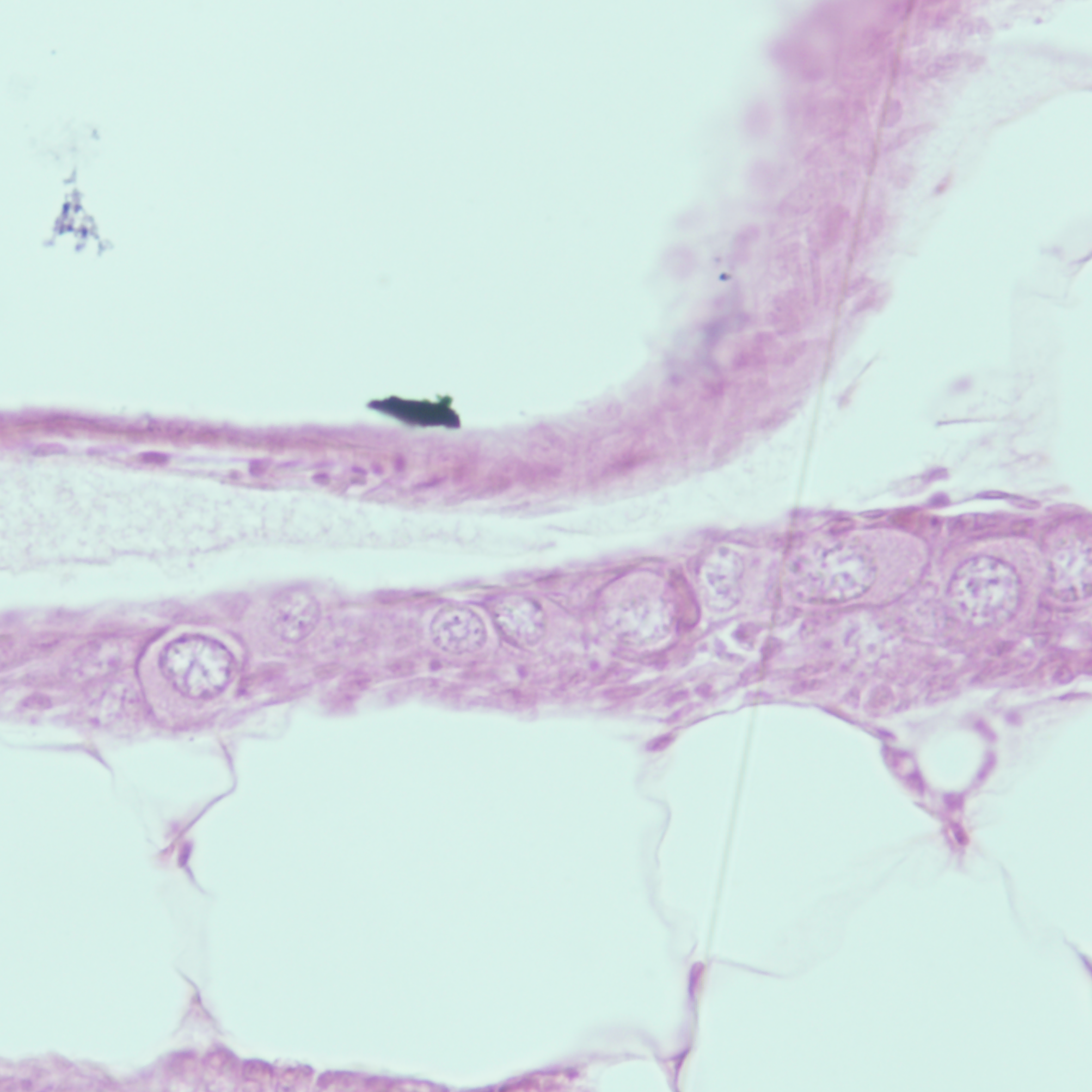

Supplement: Supplementary file 6 — Source data Fig. 2E [file 44319_2026_775_MOESM6_ESM.zip › Figure 2E/+7 line 25 dpf-hom.tif]

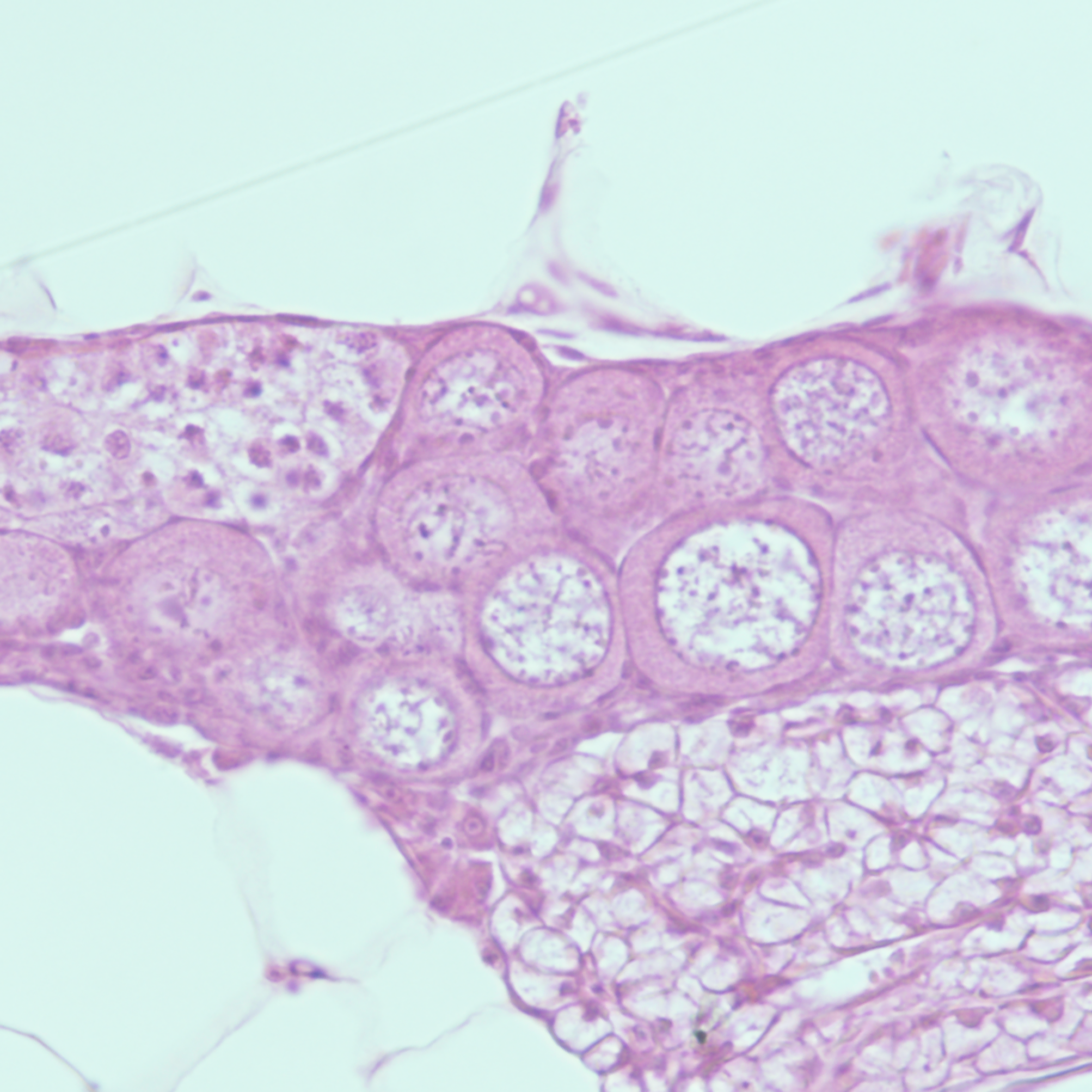

Supplement: Supplementary file 6 — Source data Fig. 2E [file 44319_2026_775_MOESM6_ESM.zip › Figure 2E/+7 line 25 dpf-WT ovary.tif]

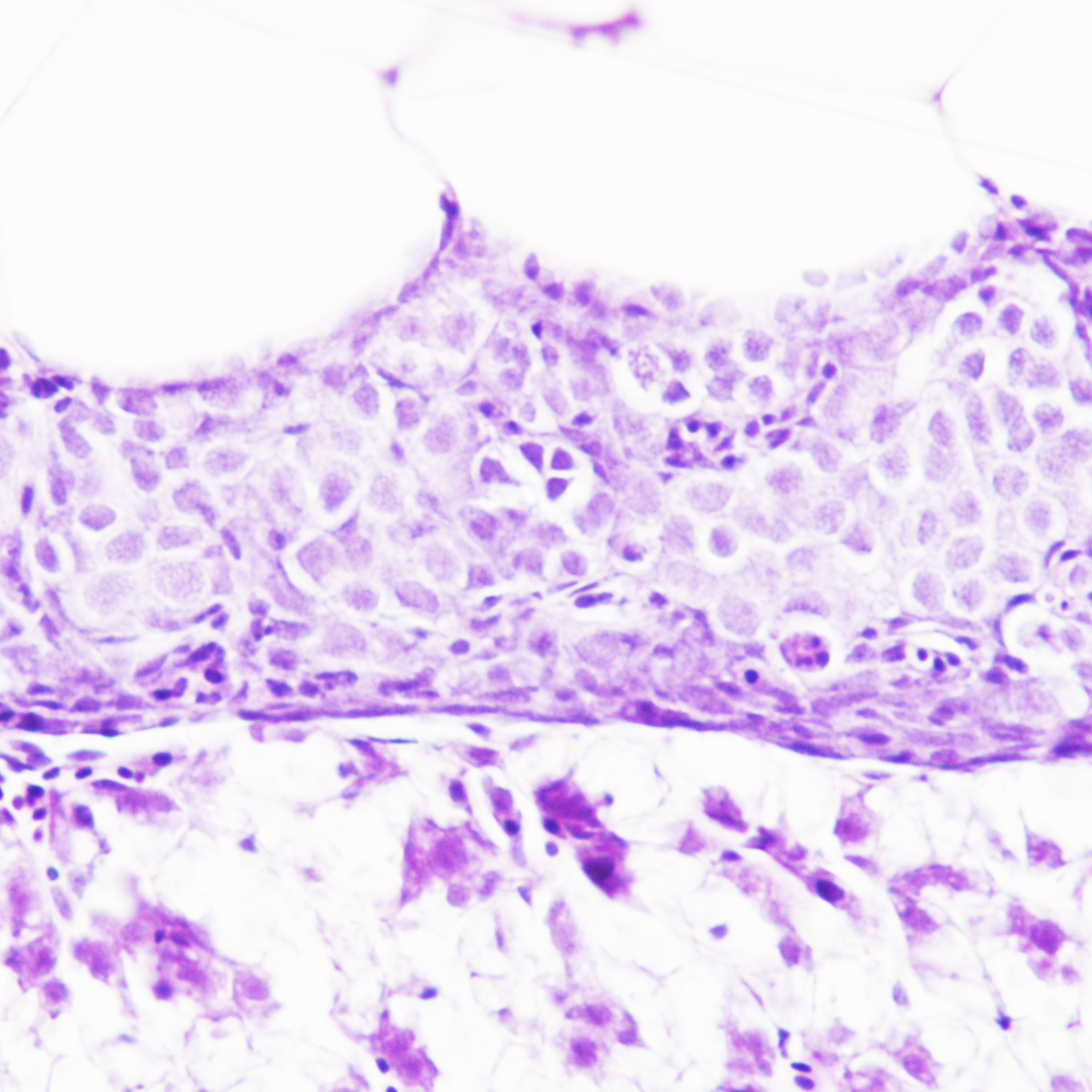

Supplement: Supplementary file 6 — Source data Fig. 2E [file 44319_2026_775_MOESM6_ESM.zip › Figure 2E/+7 line 25 dpf-WT testis.tif]

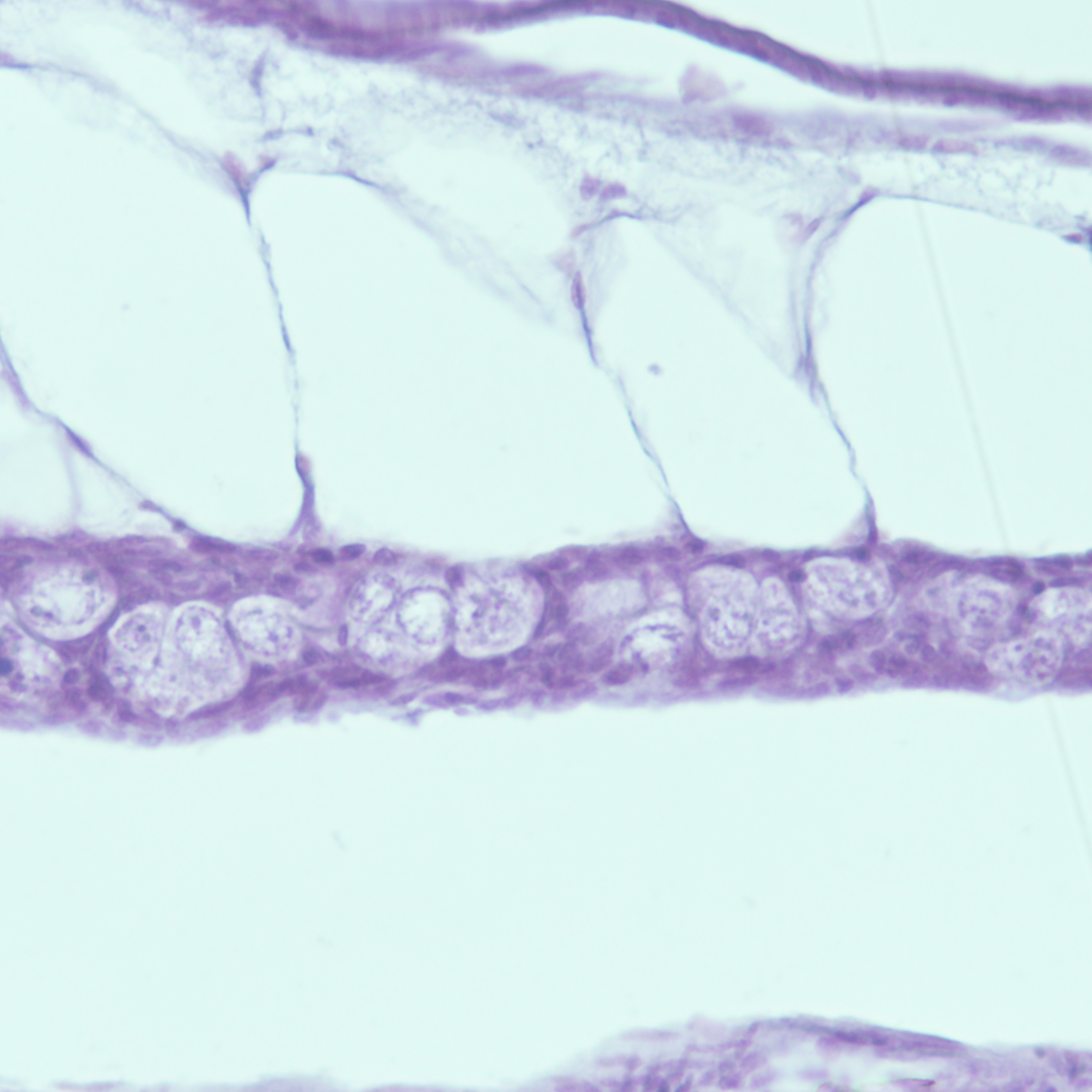

Supplement: Supplementary file 6 — Source data Fig. 2E [file 44319_2026_775_MOESM6_ESM.zip › Figure 2E/+7 line 33 dpf-hom.tif]

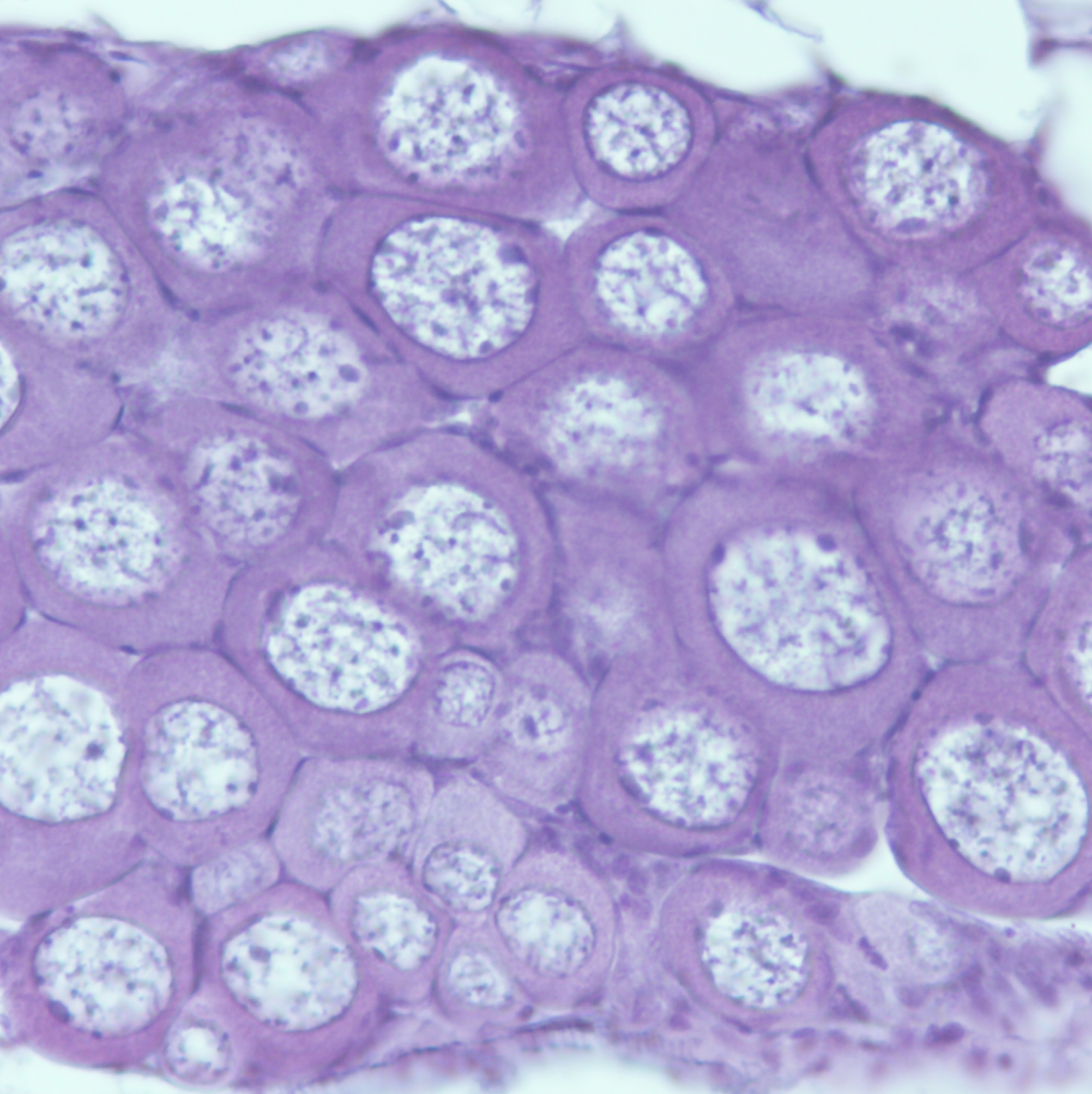

Supplement: Supplementary file 6 — Source data Fig. 2E [file 44319_2026_775_MOESM6_ESM.zip › Figure 2E/+7 line 33 dpf-WT ovary.tif]

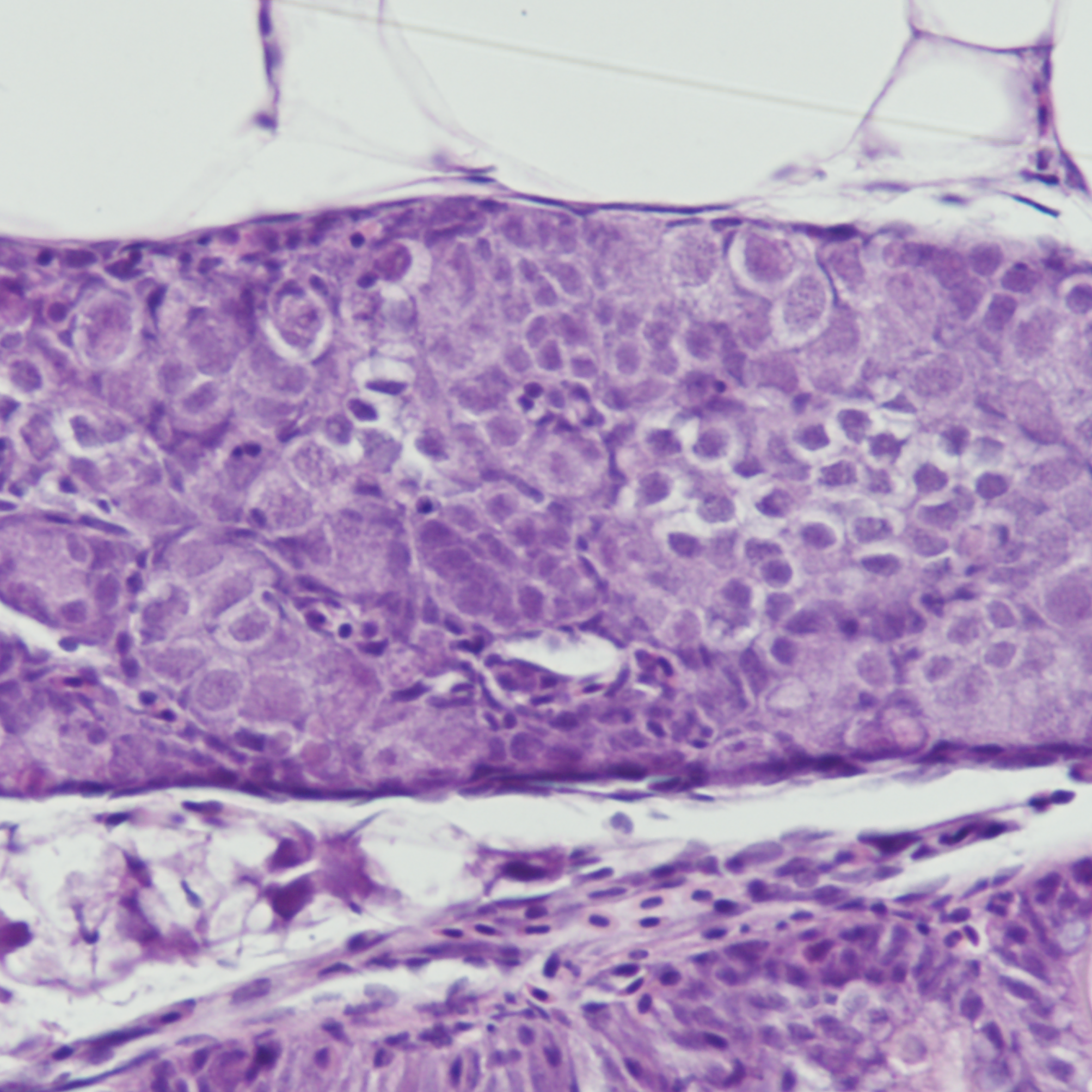

Supplement: Supplementary file 6 — Source data Fig. 2E [file 44319_2026_775_MOESM6_ESM.zip › Figure 2E/+7 line 33 dpf-WT testis.tif]

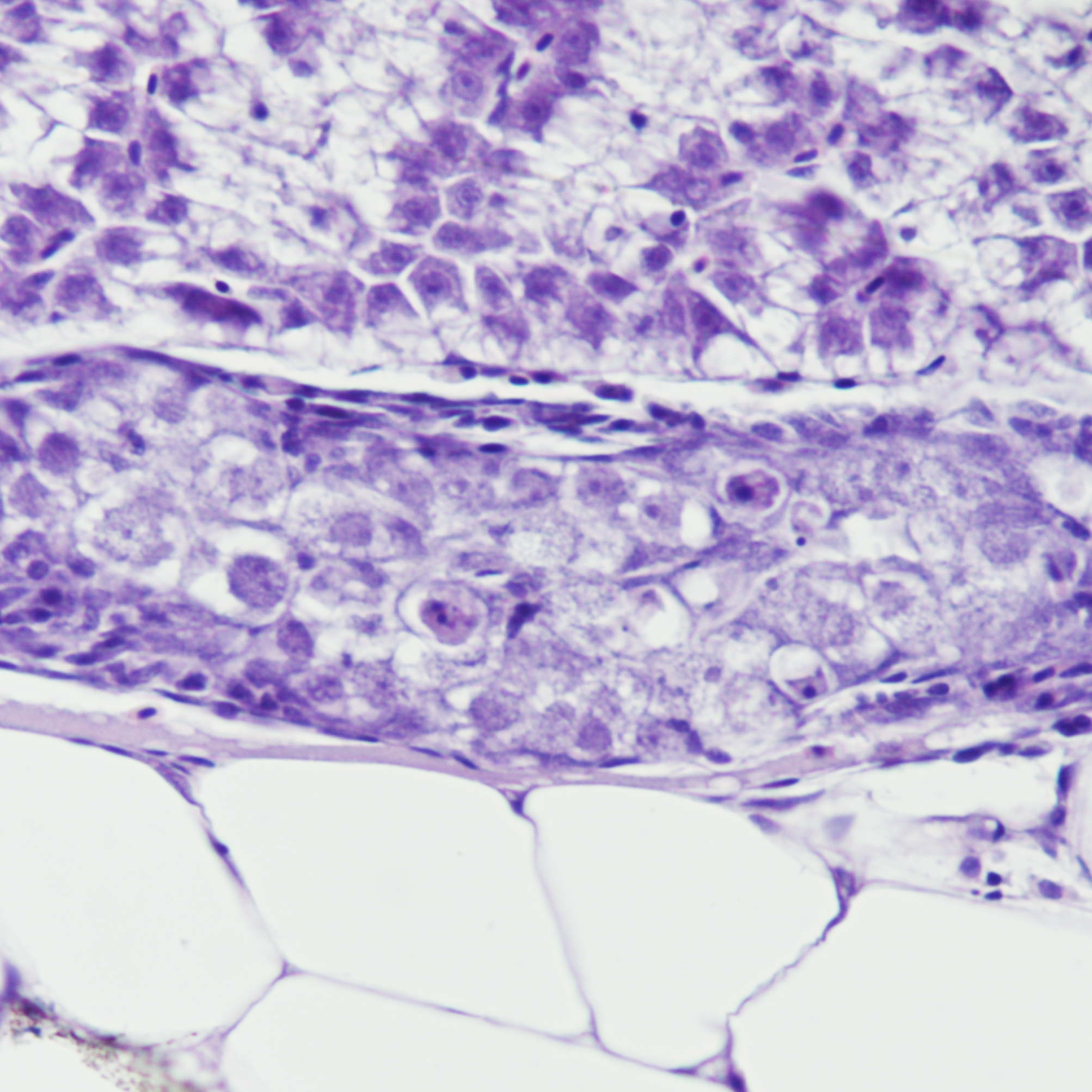

Supplement: Supplementary file 6 — Source data Fig. 2E [file 44319_2026_775_MOESM6_ESM.zip › Figure 2E/+7 line 45 dpf-hom.tif]

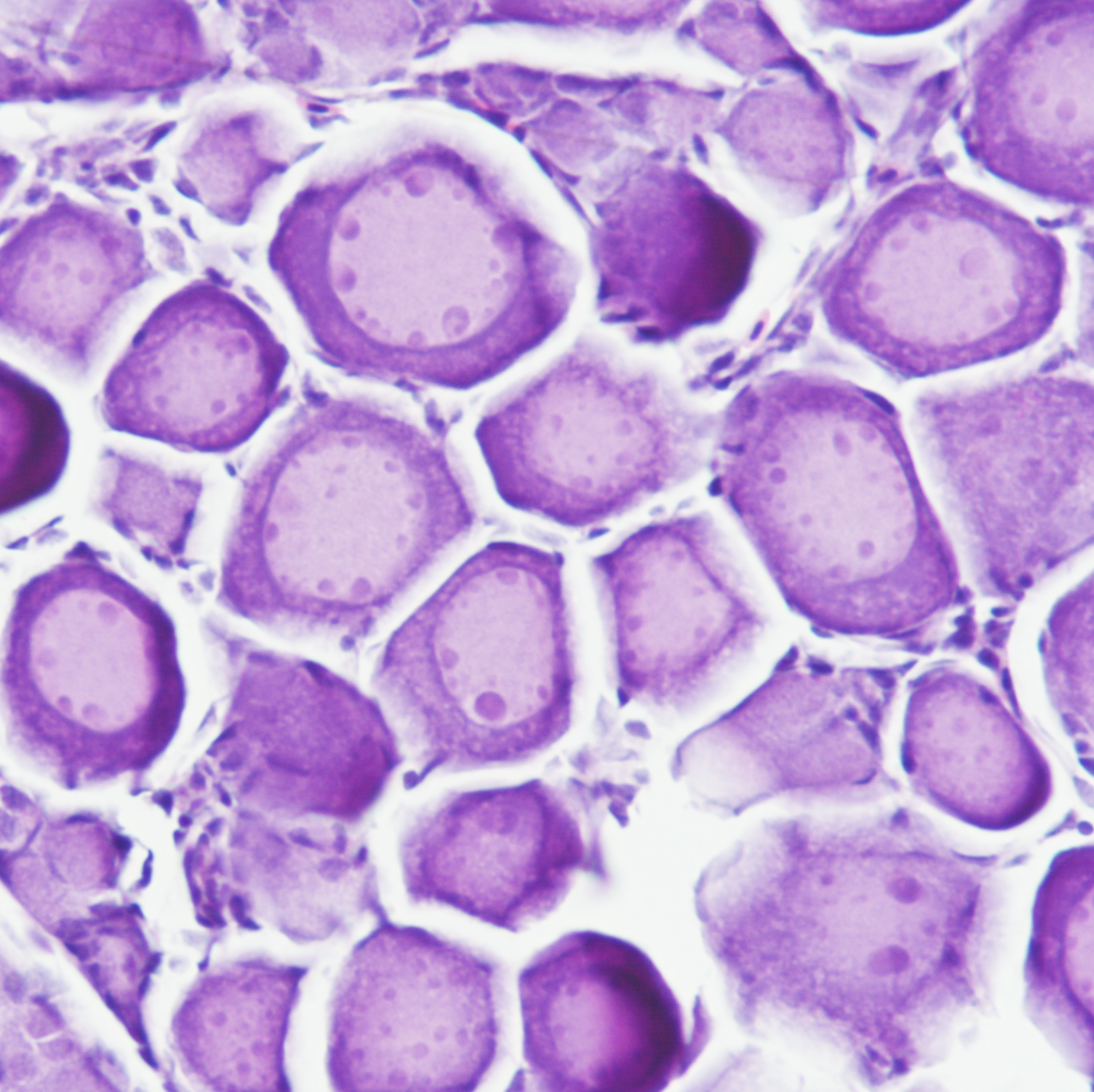

Supplement: Supplementary file 6 — Source data Fig. 2E [file 44319_2026_775_MOESM6_ESM.zip › Figure 2E/+7 line 45 dpf-WT ovary.tif]

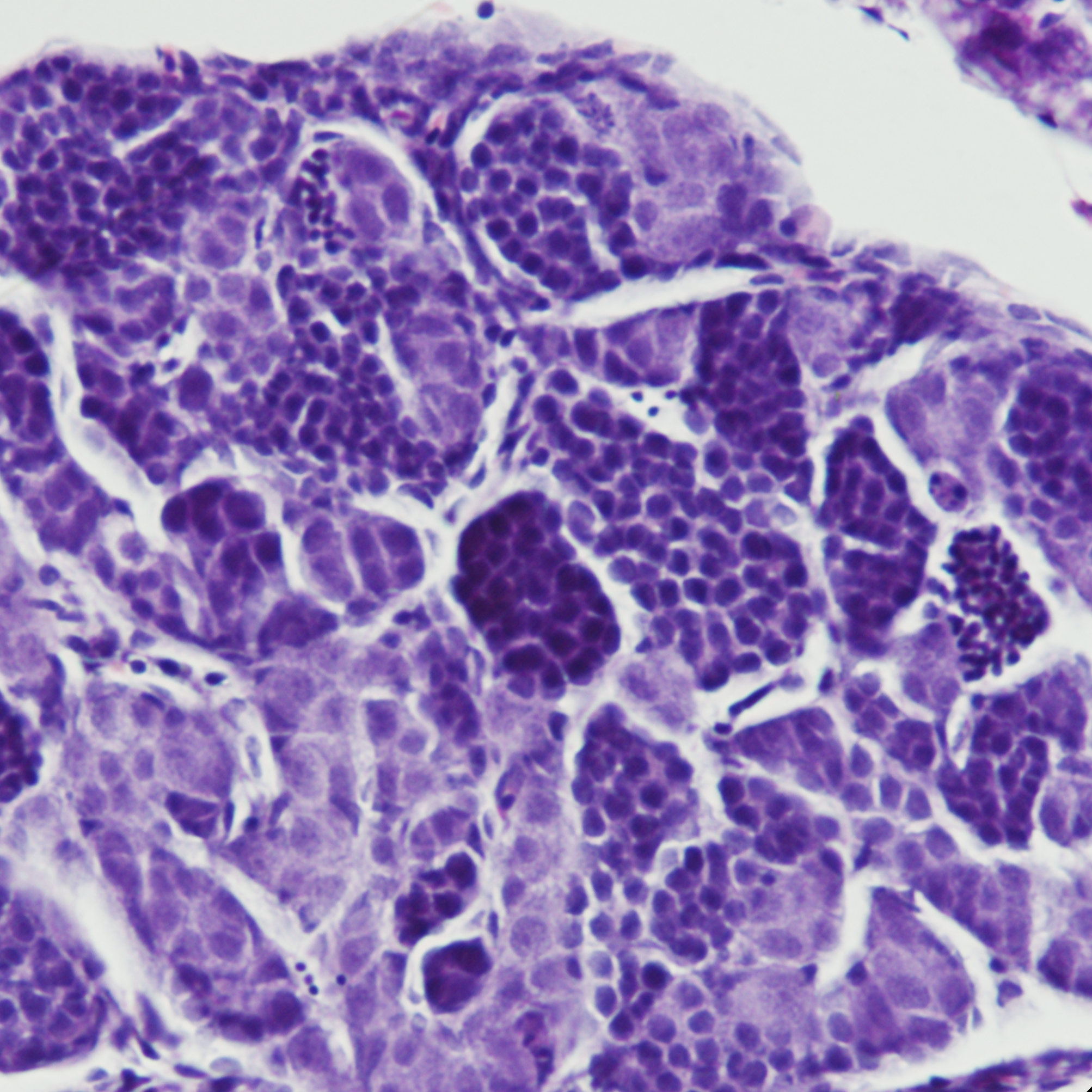

Supplement: Supplementary file 6 — Source data Fig. 2E [file 44319_2026_775_MOESM6_ESM.zip › Figure 2E/+7 line 45 dpf-WT testis.tif]

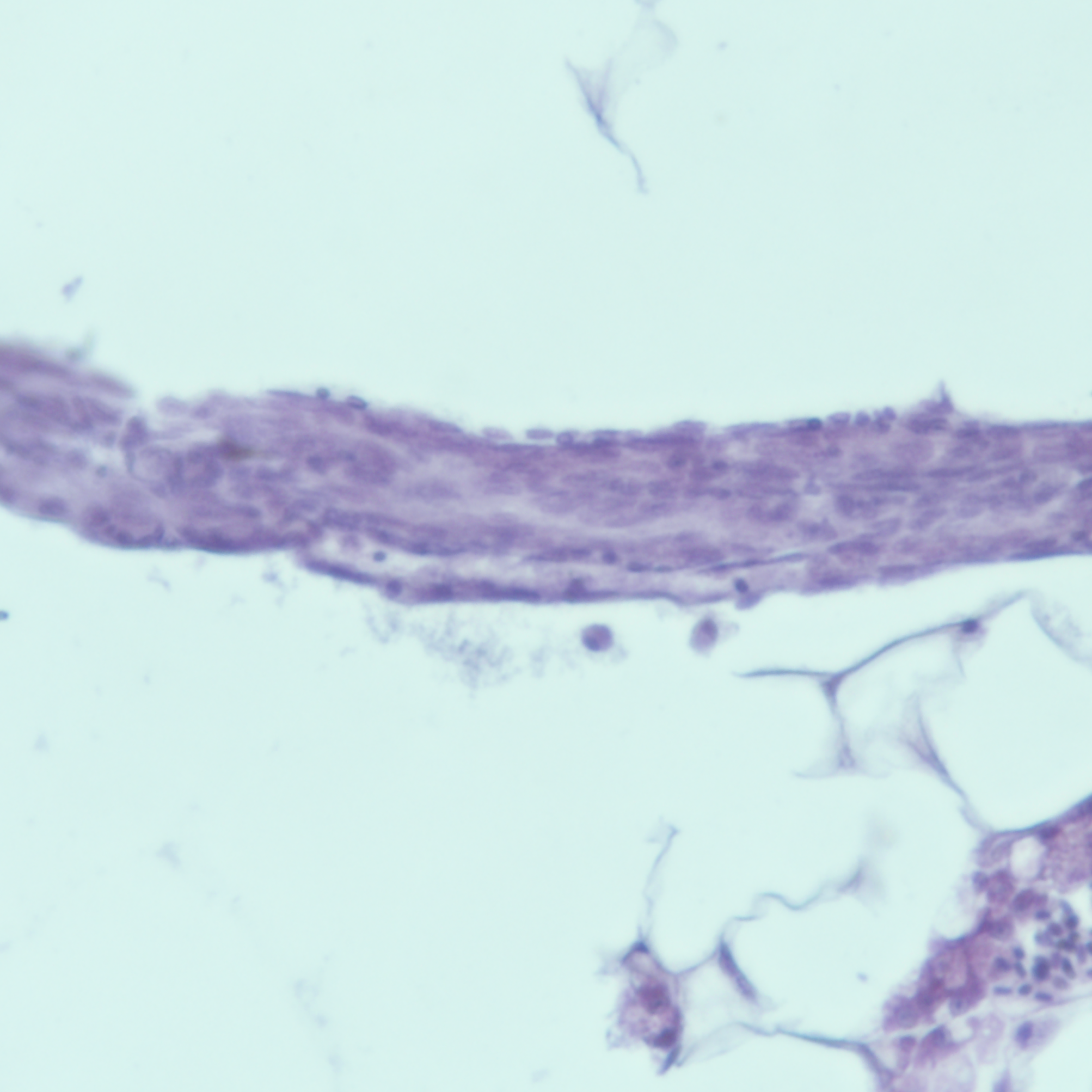

Supplement: Supplementary file 6 — Source data Fig. 2E [file 44319_2026_775_MOESM6_ESM.zip › Figure 2E/+7 line 60 dpf-hom.tif]

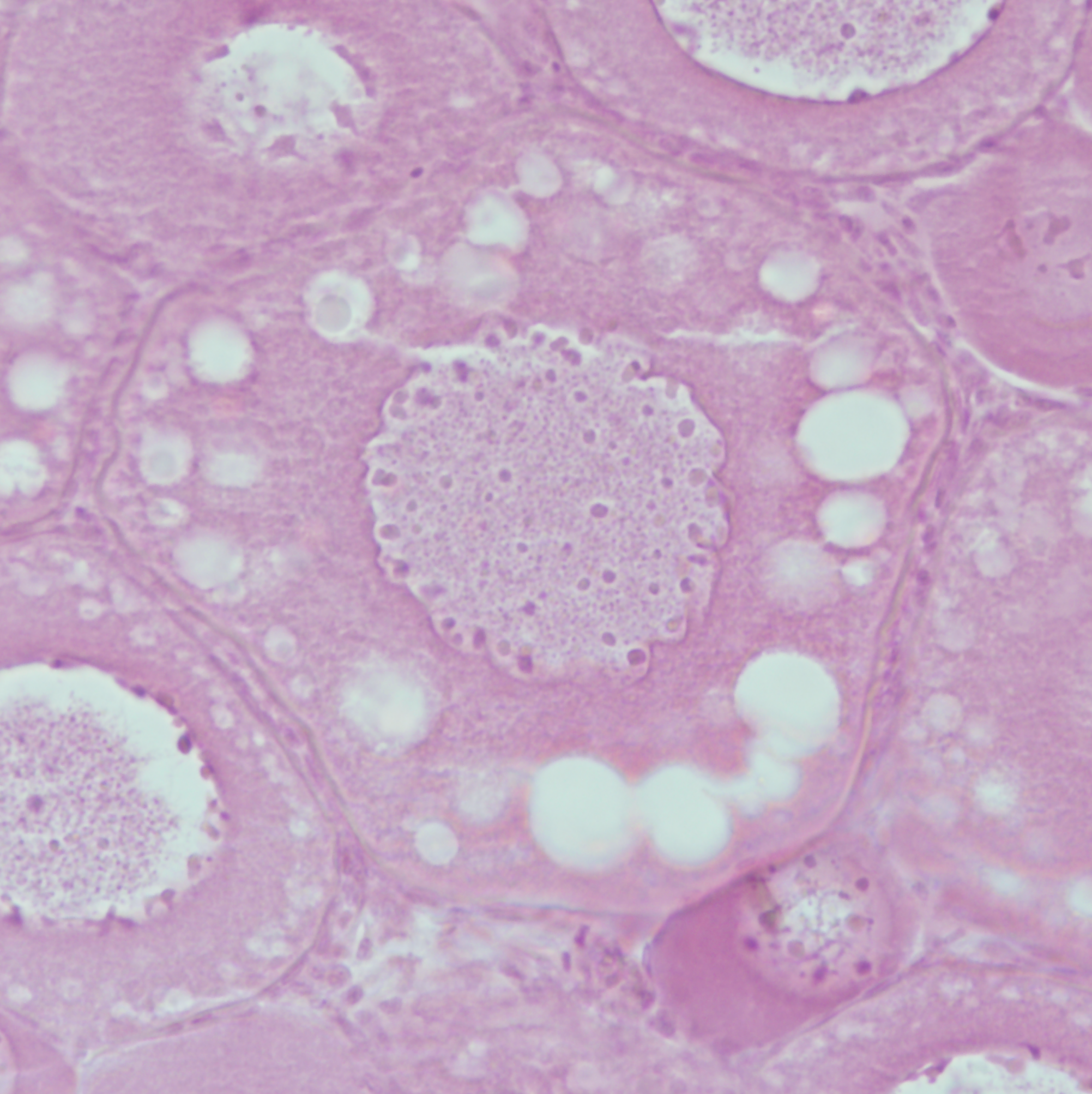

Supplement: Supplementary file 6 — Source data Fig. 2E [file 44319_2026_775_MOESM6_ESM.zip › Figure 2E/+7 line 60 dpf-WT ovary.tif]

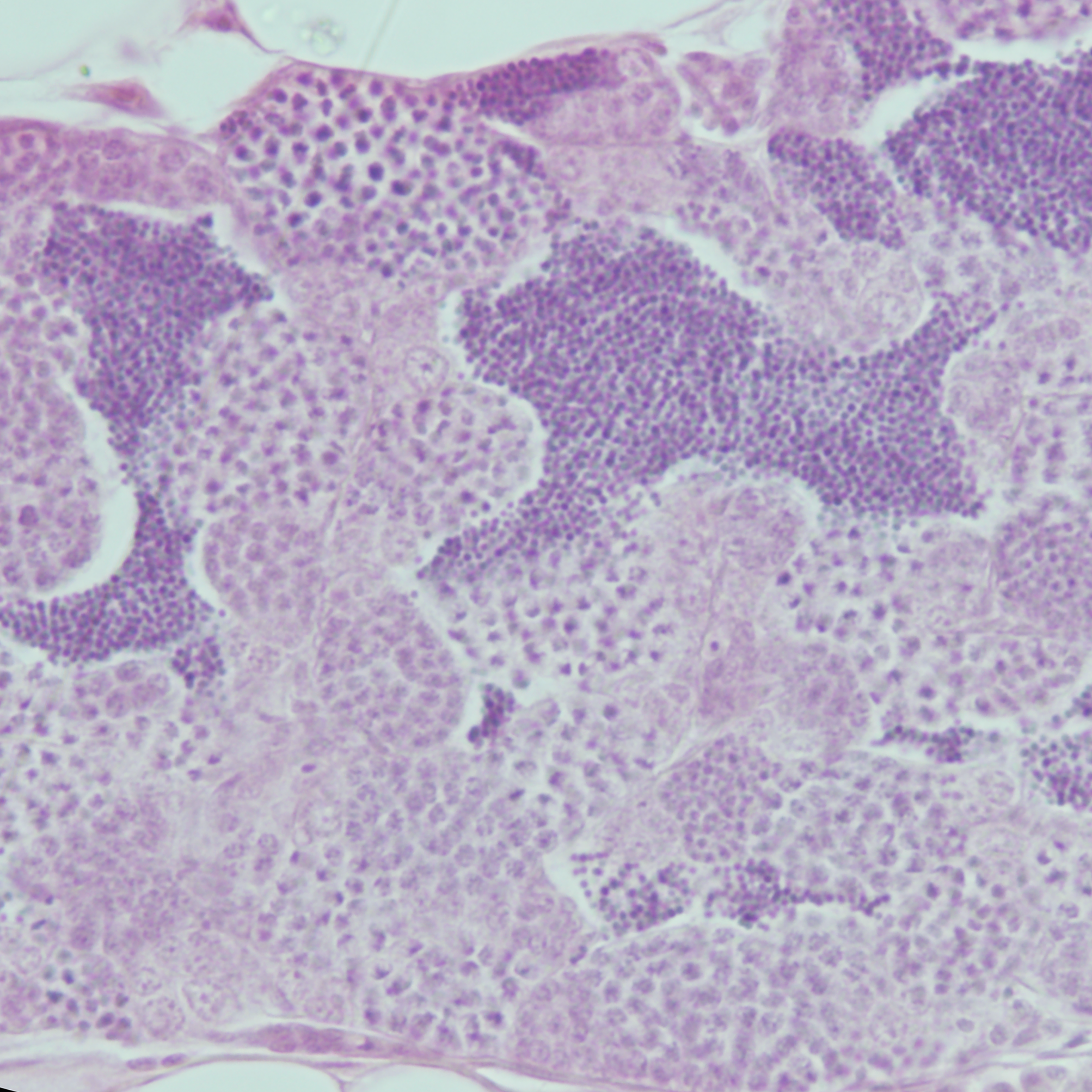

Supplement: Supplementary file 6 — Source data Fig. 2E [file 44319_2026_775_MOESM6_ESM.zip › Figure 2E/+7 line 60 dpf-WT testis.tif]

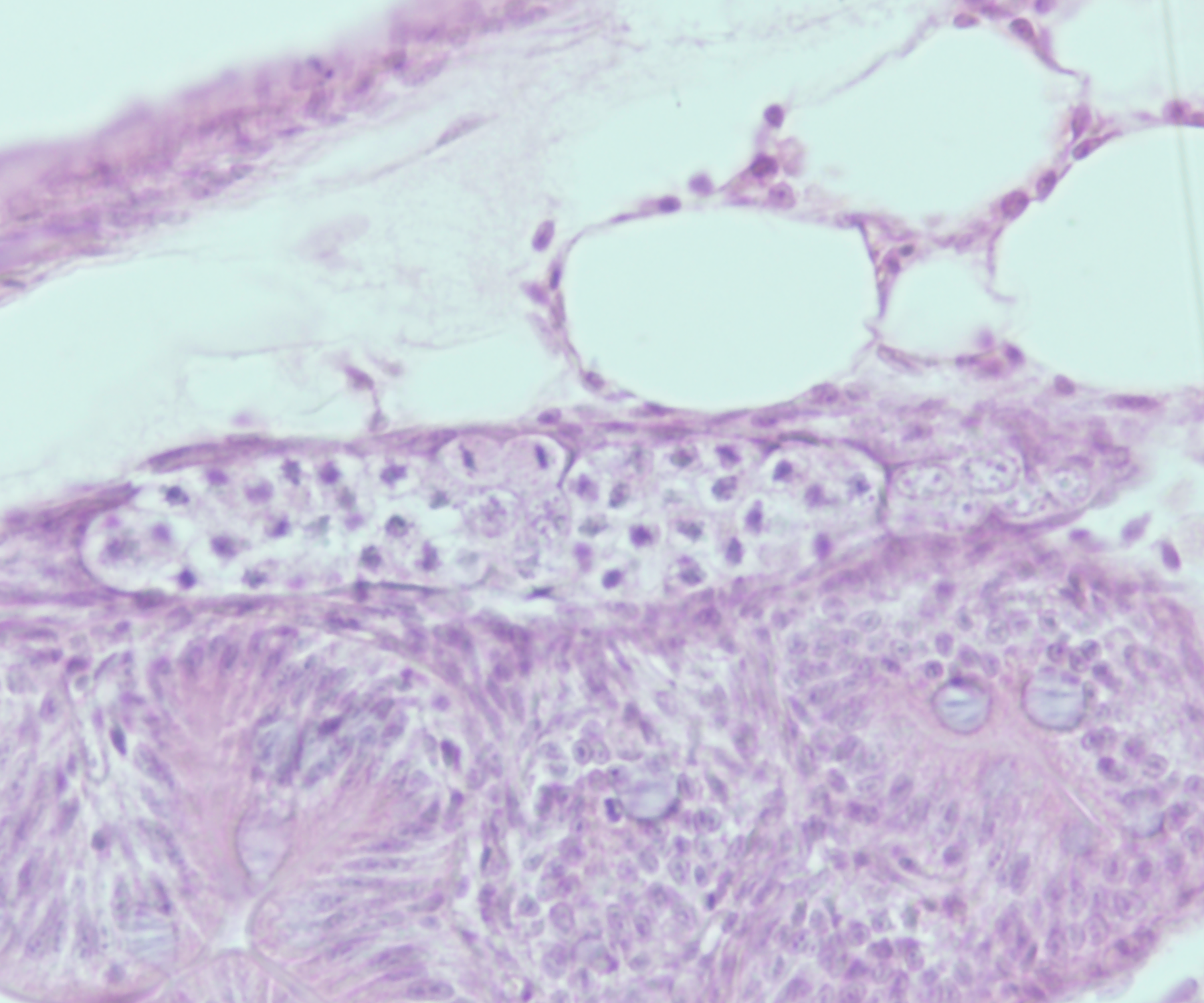

Supplement: Supplementary file 6 — Source data Fig. 2E [file 44319_2026_775_MOESM6_ESM.zip › Figure 2E/Δ7 line 19 dpf-hom.tif]

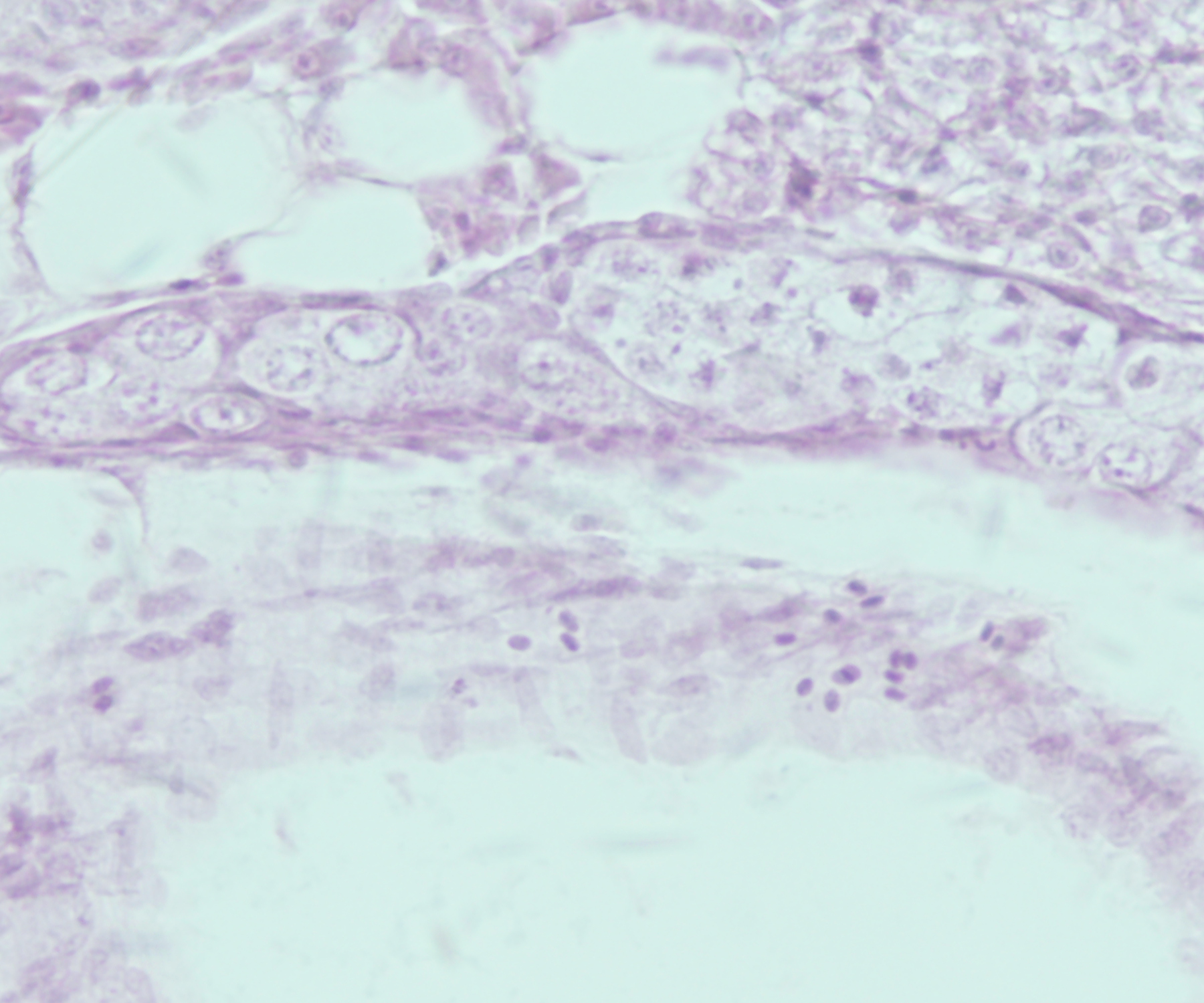

Supplement: Supplementary file 6 — Source data Fig. 2E [file 44319_2026_775_MOESM6_ESM.zip › Figure 2E/Δ7 line 19 dpf-WT.tif]

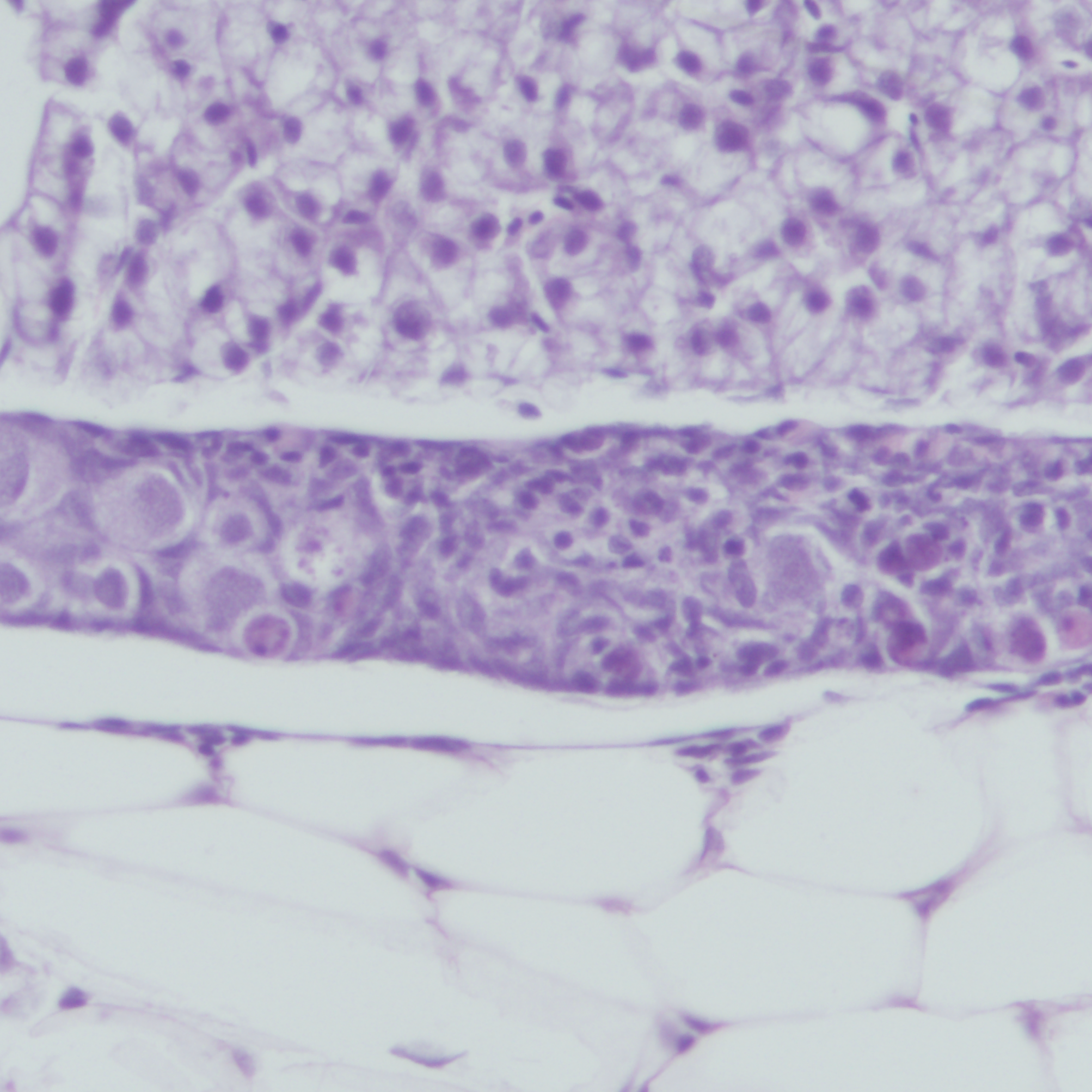

Supplement: Supplementary file 6 — Source data Fig. 2E [file 44319_2026_775_MOESM6_ESM.zip › Figure 2E/Δ7 line 25 dpf-hom.tif]

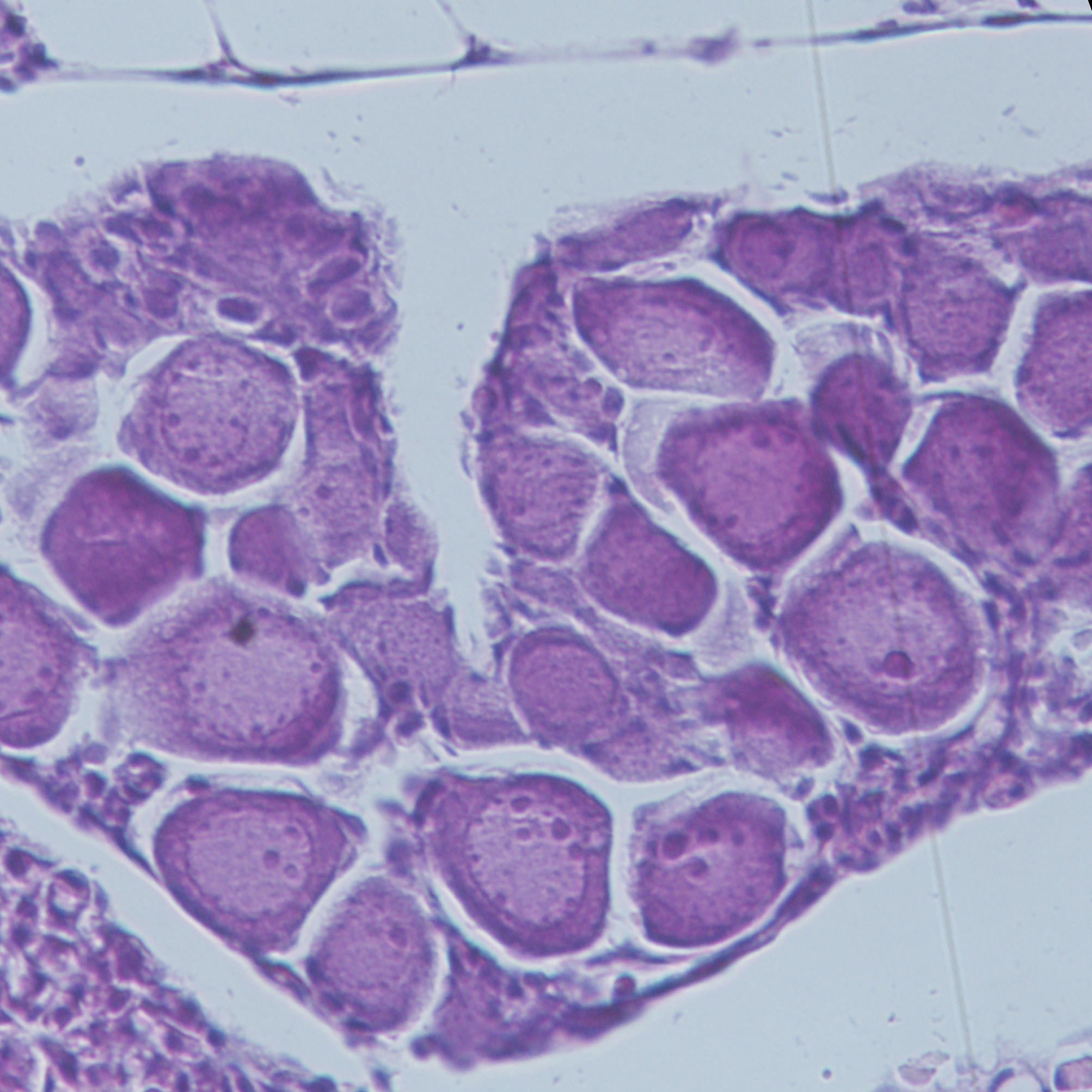

Supplement: Supplementary file 6 — Source data Fig. 2E [file 44319_2026_775_MOESM6_ESM.zip › Figure 2E/Δ7 line 25 dpf-WT ovary.tif]

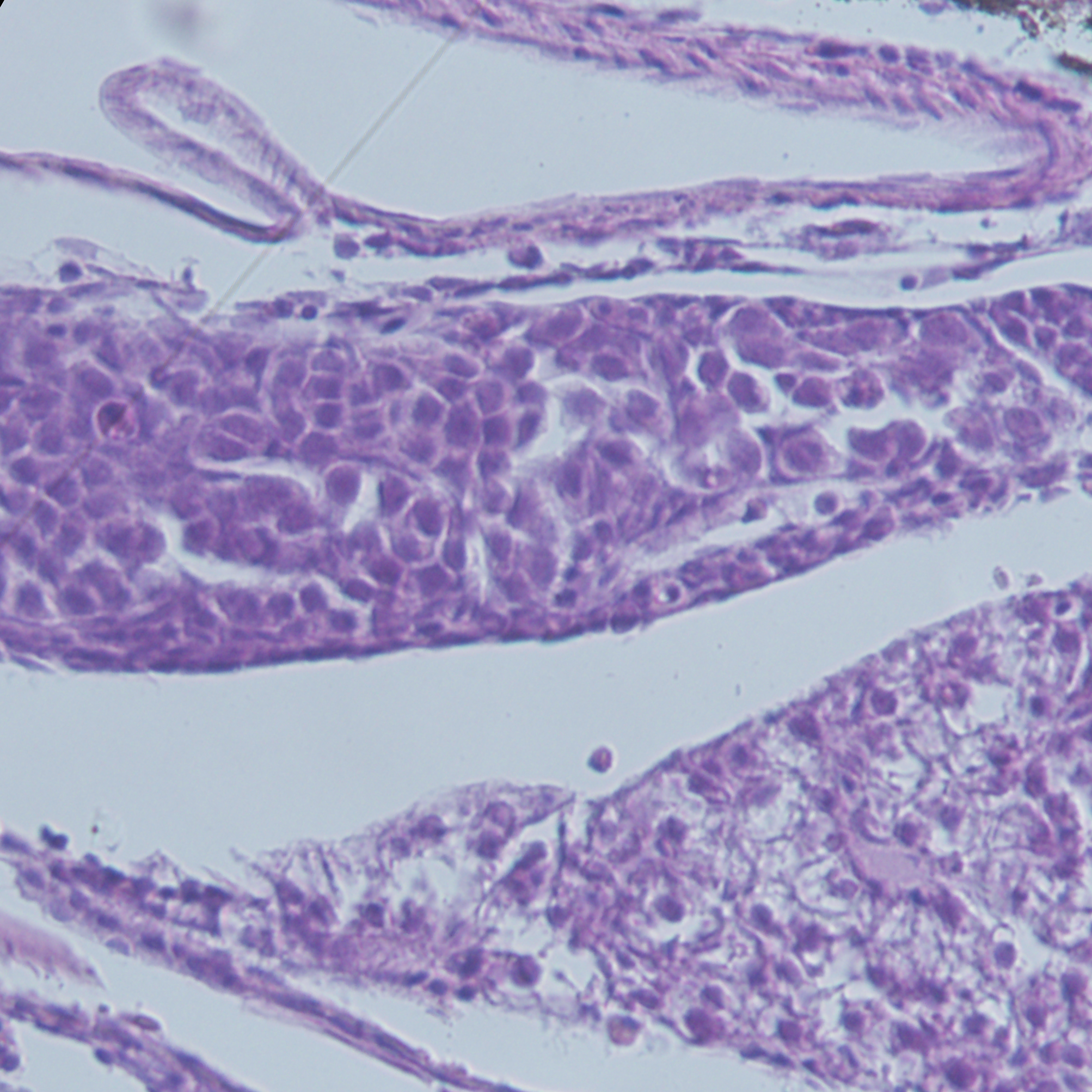

Supplement: Supplementary file 6 — Source data Fig. 2E [file 44319_2026_775_MOESM6_ESM.zip › Figure 2E/Δ7 line 25 dpf-WT testis.tif]

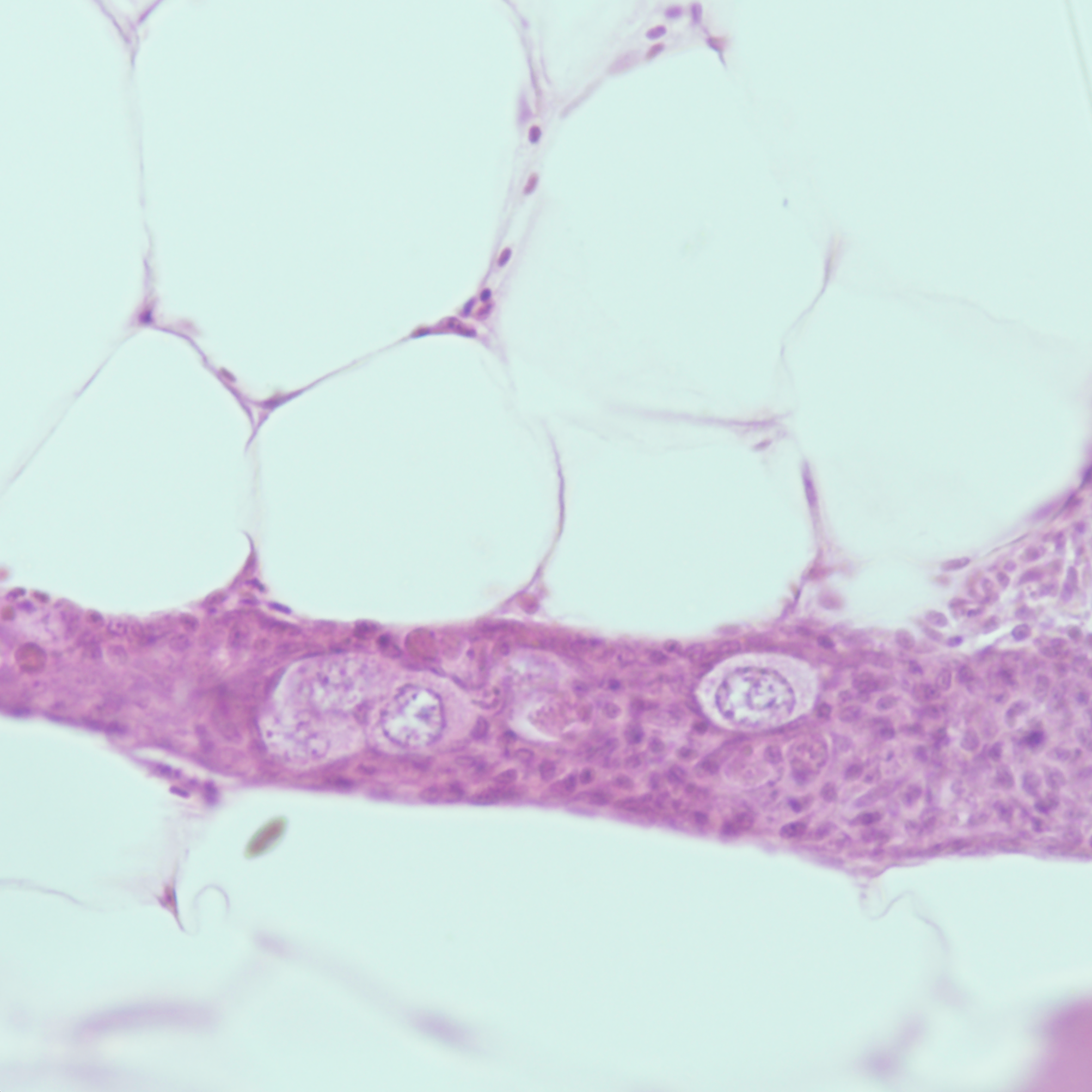

Supplement: Supplementary file 6 — Source data Fig. 2E [file 44319_2026_775_MOESM6_ESM.zip › Figure 2E/Δ7 line 33 dpf-hom.tif]

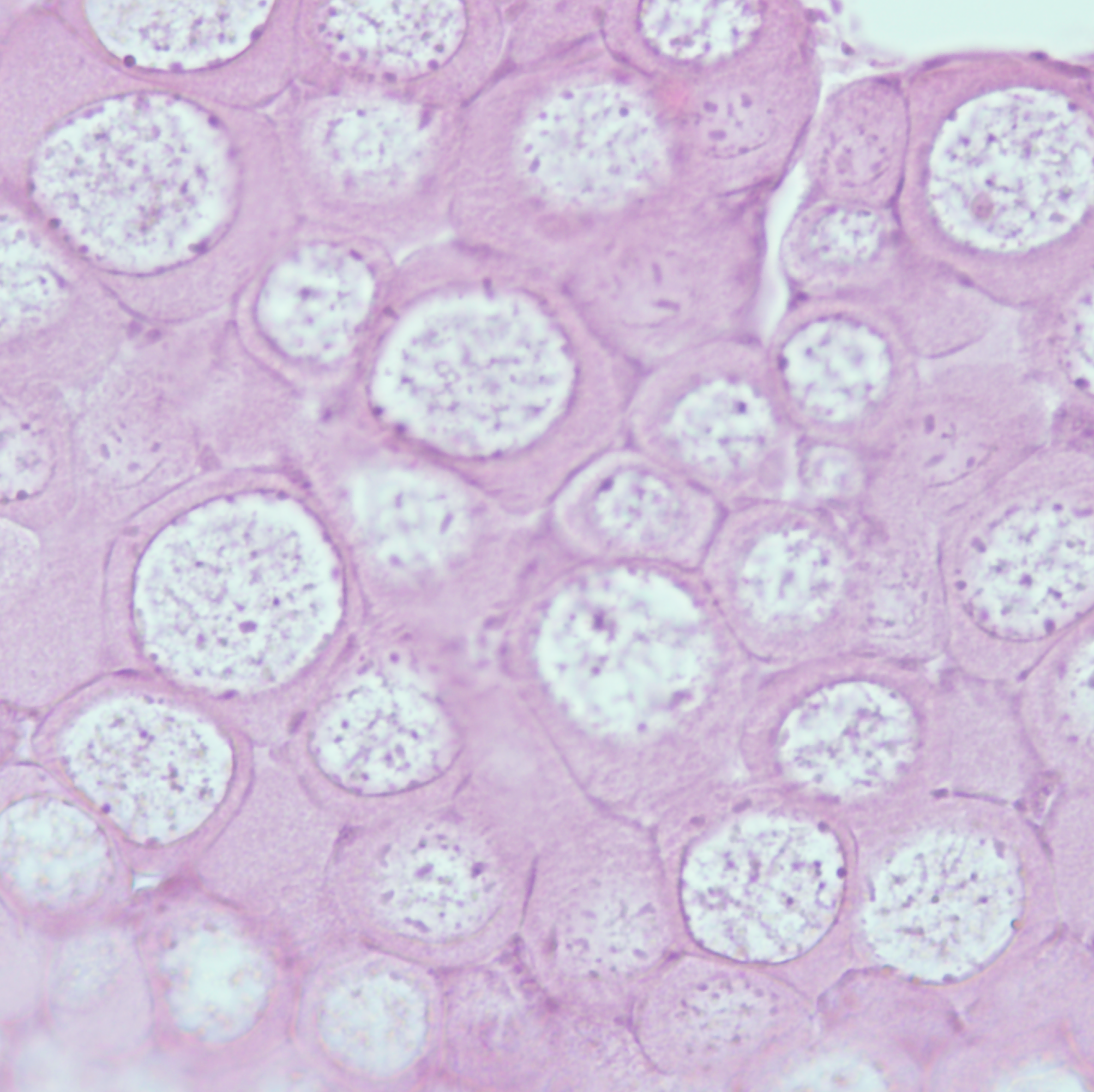

Supplement: Supplementary file 6 — Source data Fig. 2E [file 44319_2026_775_MOESM6_ESM.zip › Figure 2E/Δ7 line 33 dpf-WT ovary.tif]

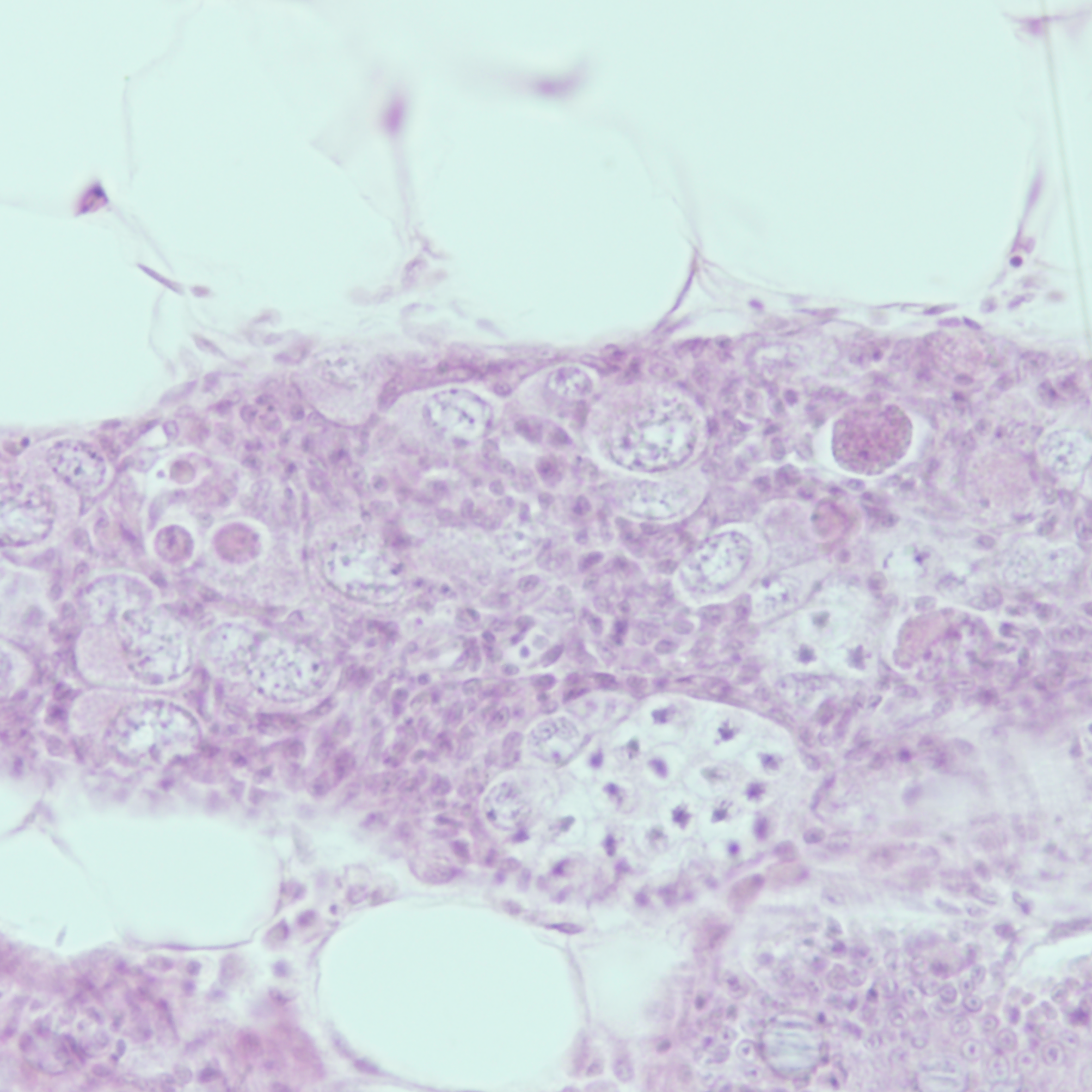

Supplement: Supplementary file 6 — Source data Fig. 2E [file 44319_2026_775_MOESM6_ESM.zip › Figure 2E/Δ7 line 33 dpf-WT testis.tif]

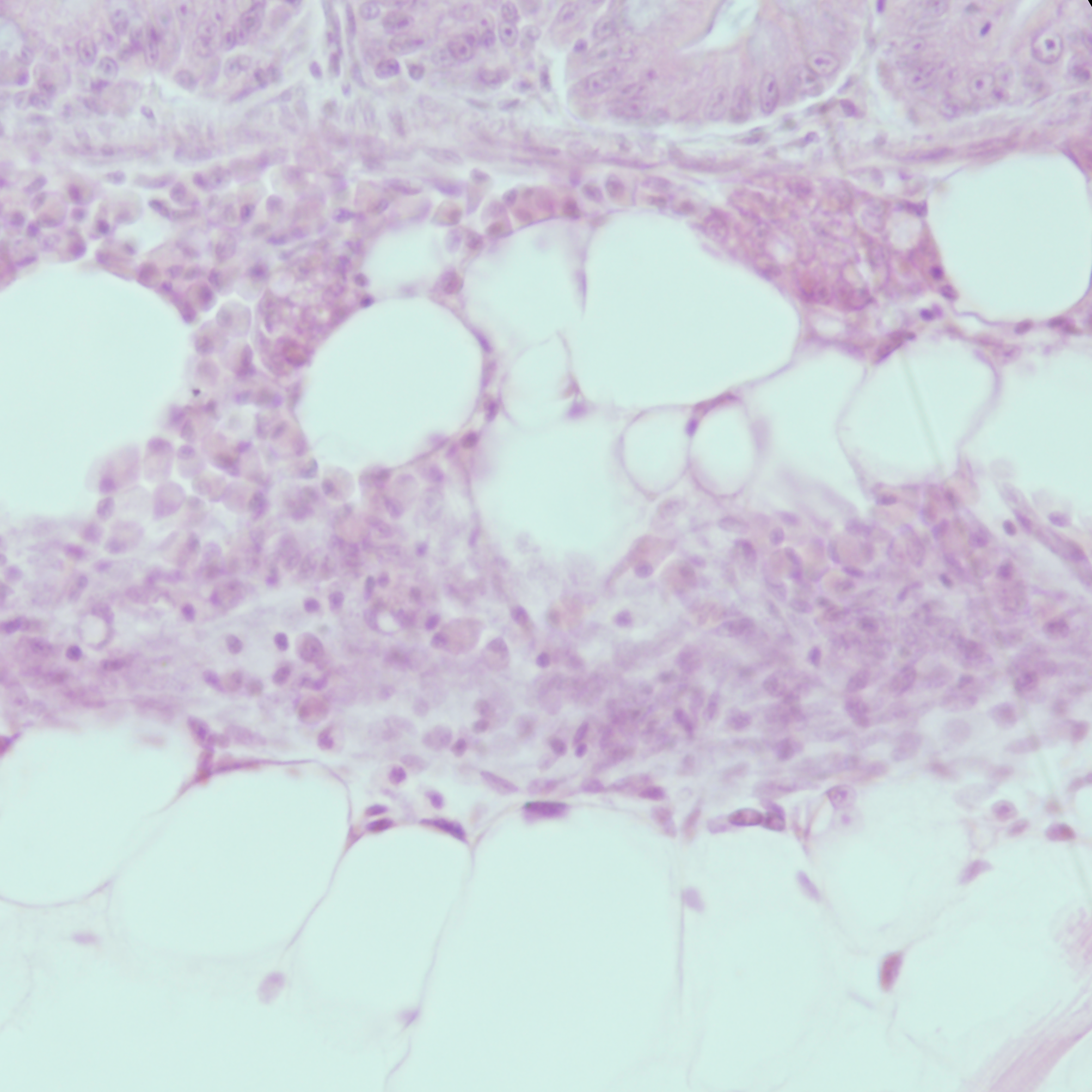

Supplement: Supplementary file 6 — Source data Fig. 2E [file 44319_2026_775_MOESM6_ESM.zip › Figure 2E/Δ7 line 45 dpf-hom.tif]

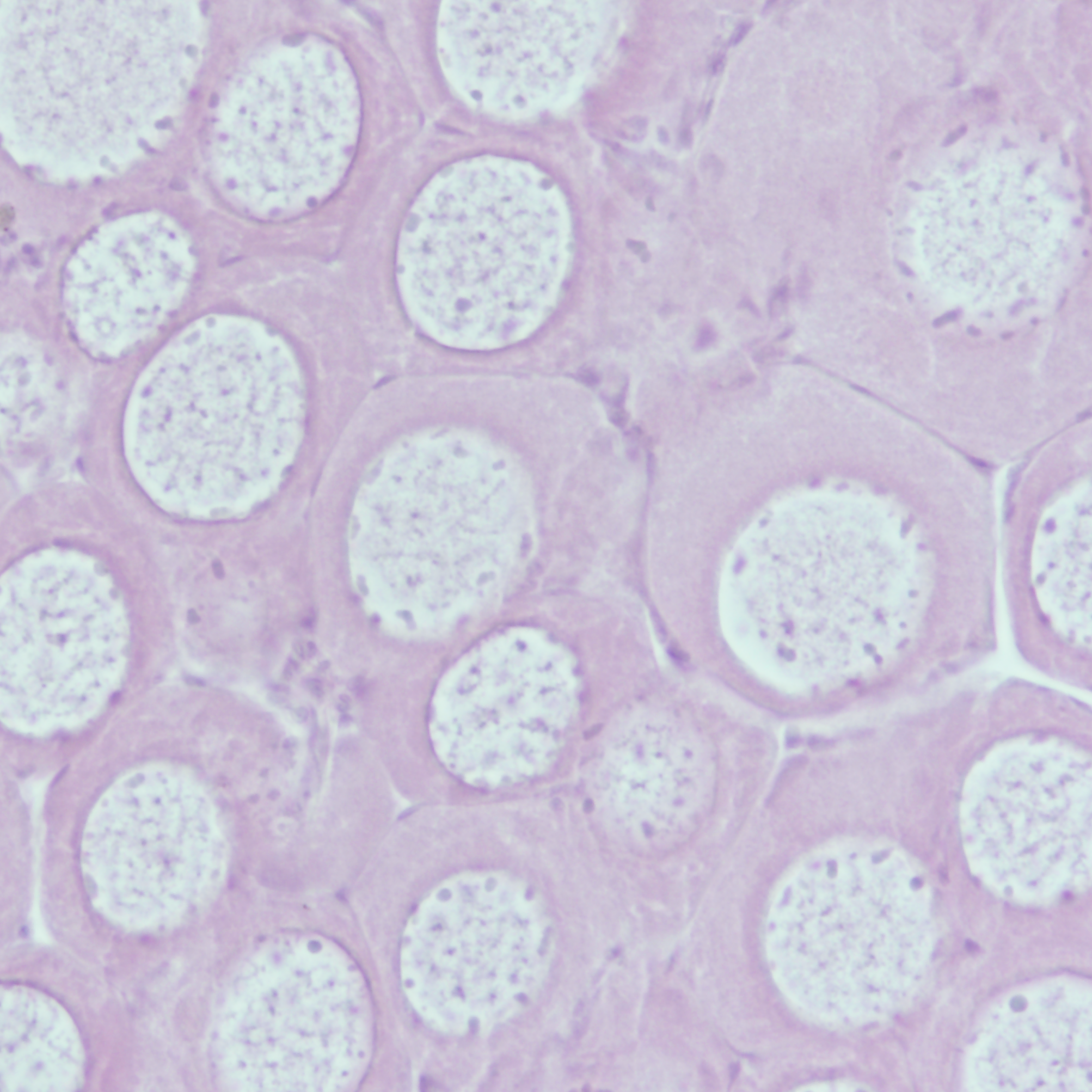

Supplement: Supplementary file 6 — Source data Fig. 2E [file 44319_2026_775_MOESM6_ESM.zip › Figure 2E/Δ7 line 45 dpf-WT ovary.tif]

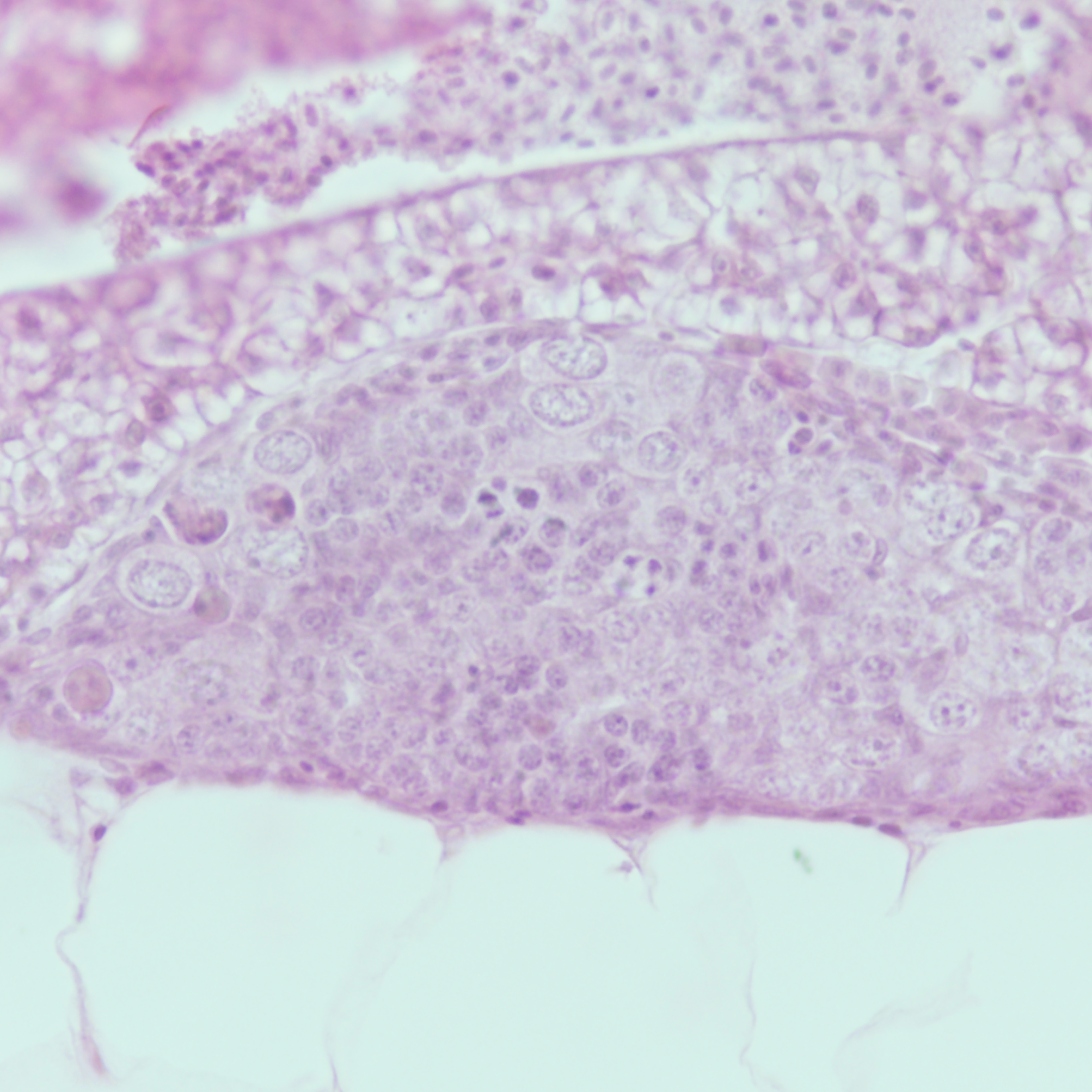

Supplement: Supplementary file 6 — Source data Fig. 2E [file 44319_2026_775_MOESM6_ESM.zip › Figure 2E/Δ7 line 45 dpf-WT testis.tif]

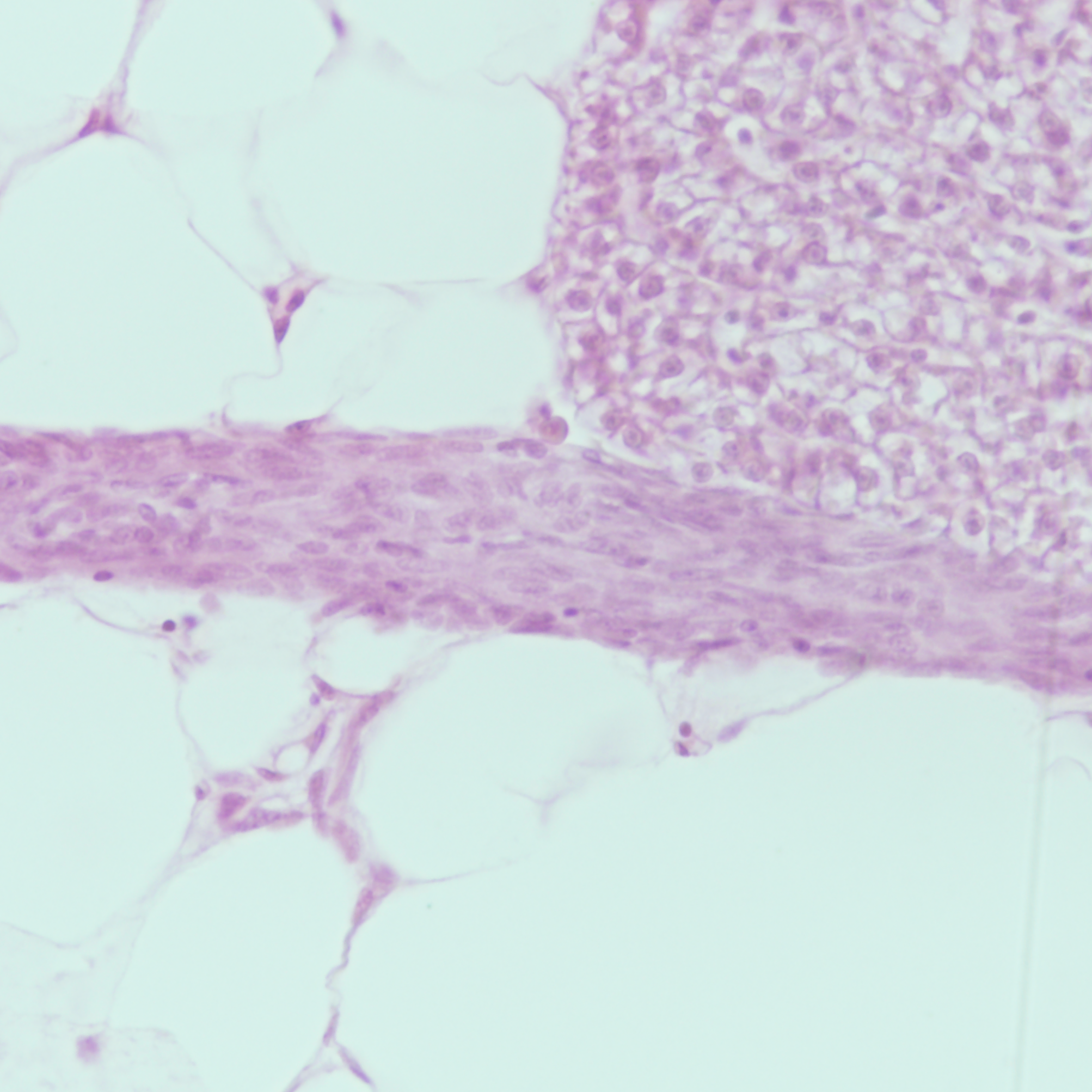

Supplement: Supplementary file 6 — Source data Fig. 2E [file 44319_2026_775_MOESM6_ESM.zip › Figure 2E/Δ7 line 60 dpf-hom.tif]

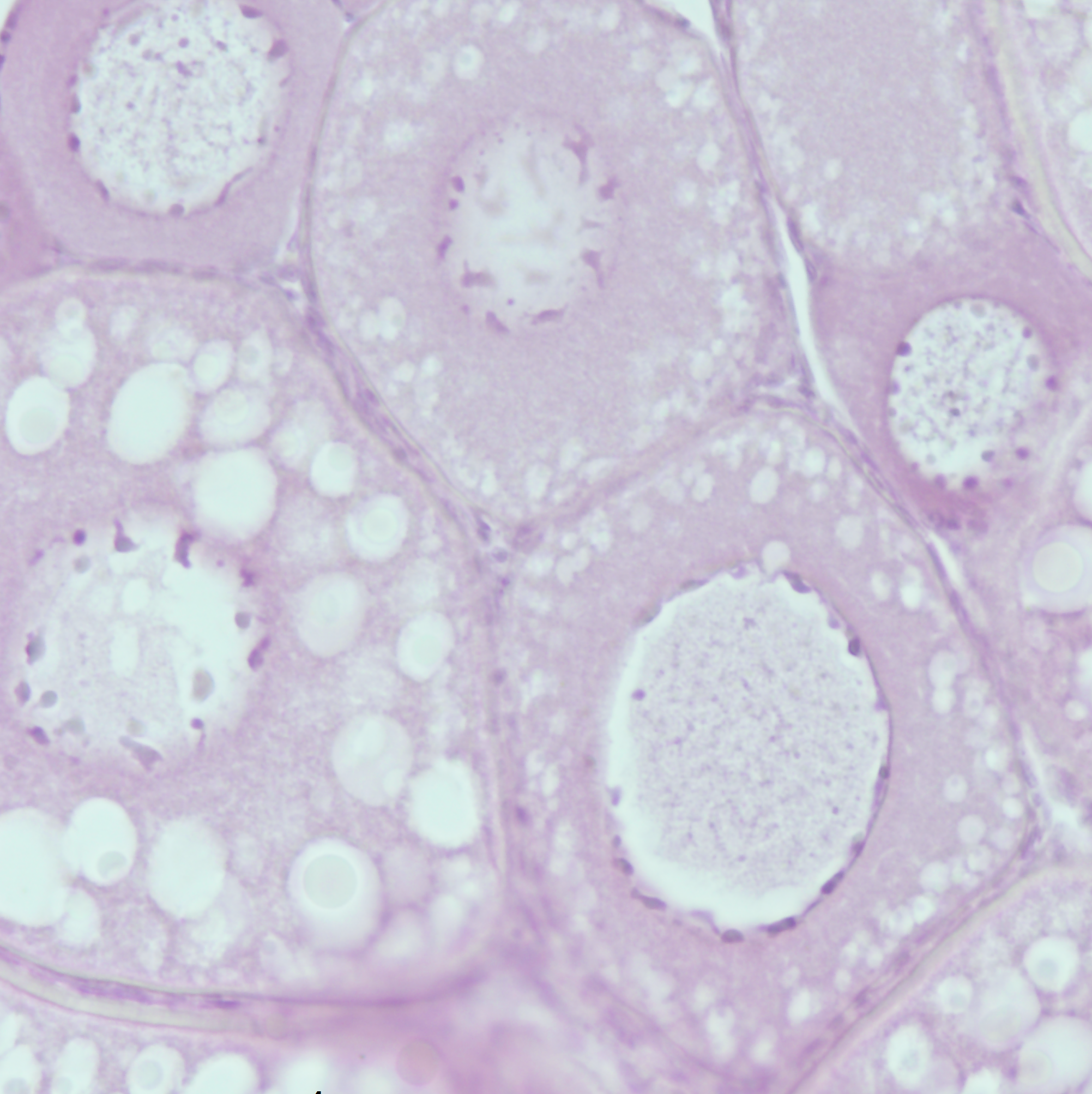

Supplement: Supplementary file 6 — Source data Fig. 2E [file 44319_2026_775_MOESM6_ESM.zip › Figure 2E/Δ7 line 60 dpf-WT ovary.tif]

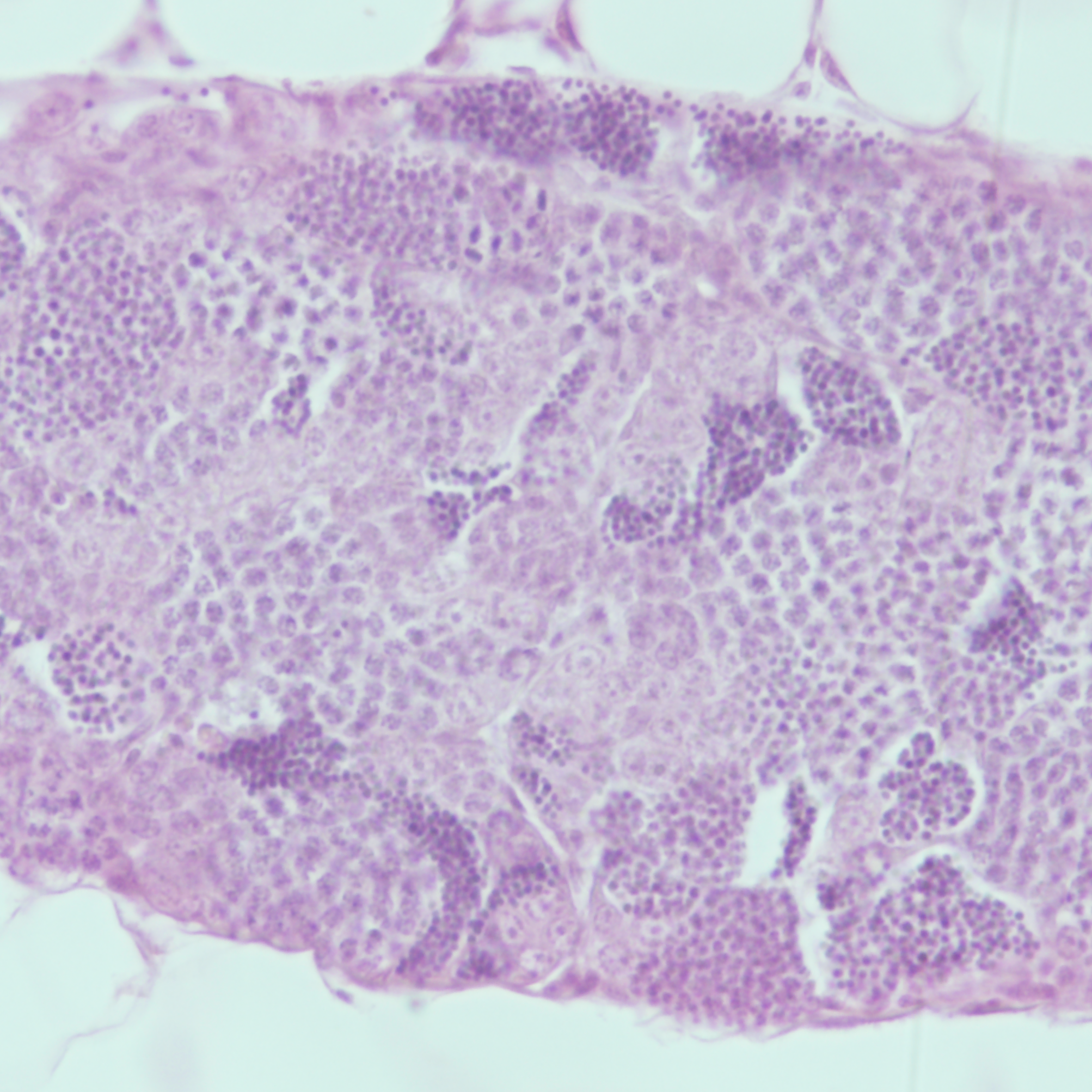

Supplement: Supplementary file 6 — Source data Fig. 2E [file 44319_2026_775_MOESM6_ESM.zip › Figure 2E/Δ7 line 60 dpf-WT testis.tif]

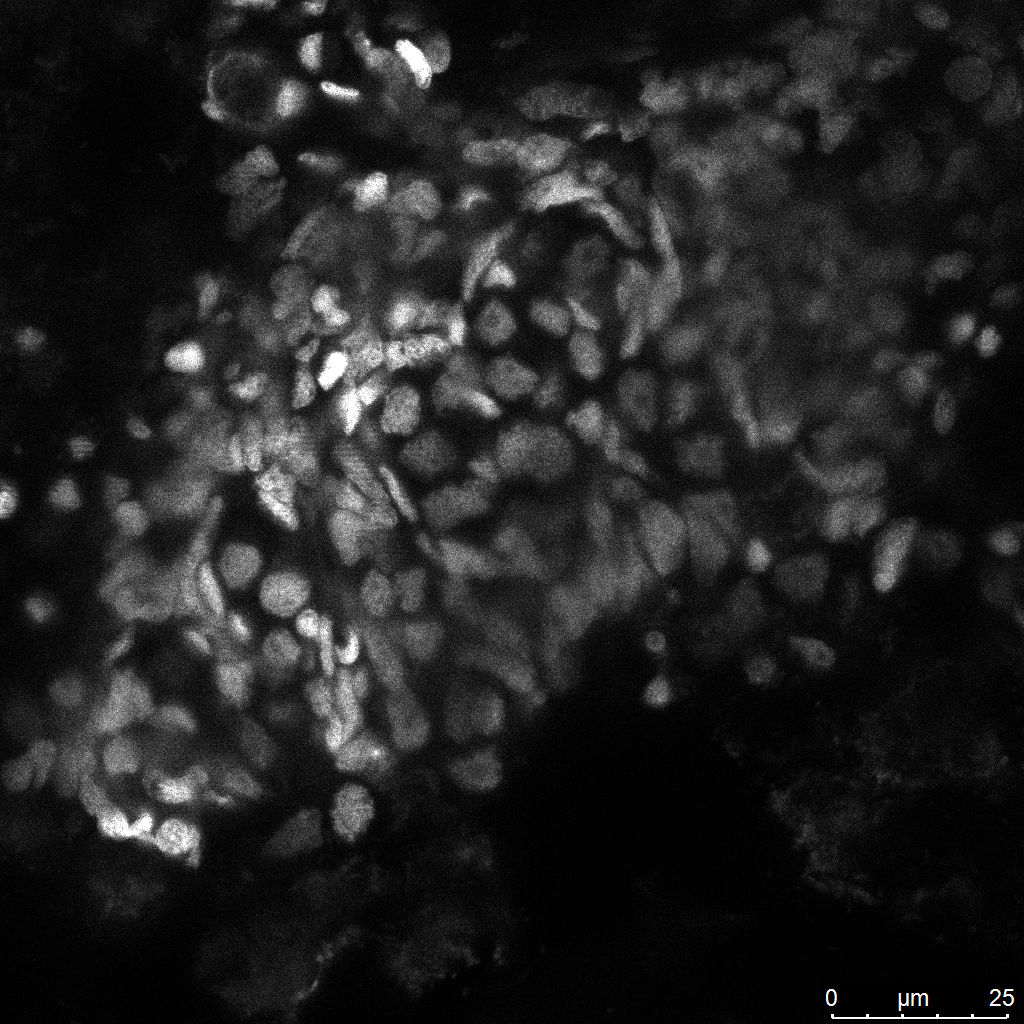

Supplement: Supplementary file 7 — Source data Fig. 3 [file 44319_2026_775_MOESM7_ESM.zip › Figure 3/Figure 3A/DAPI hom.tif]

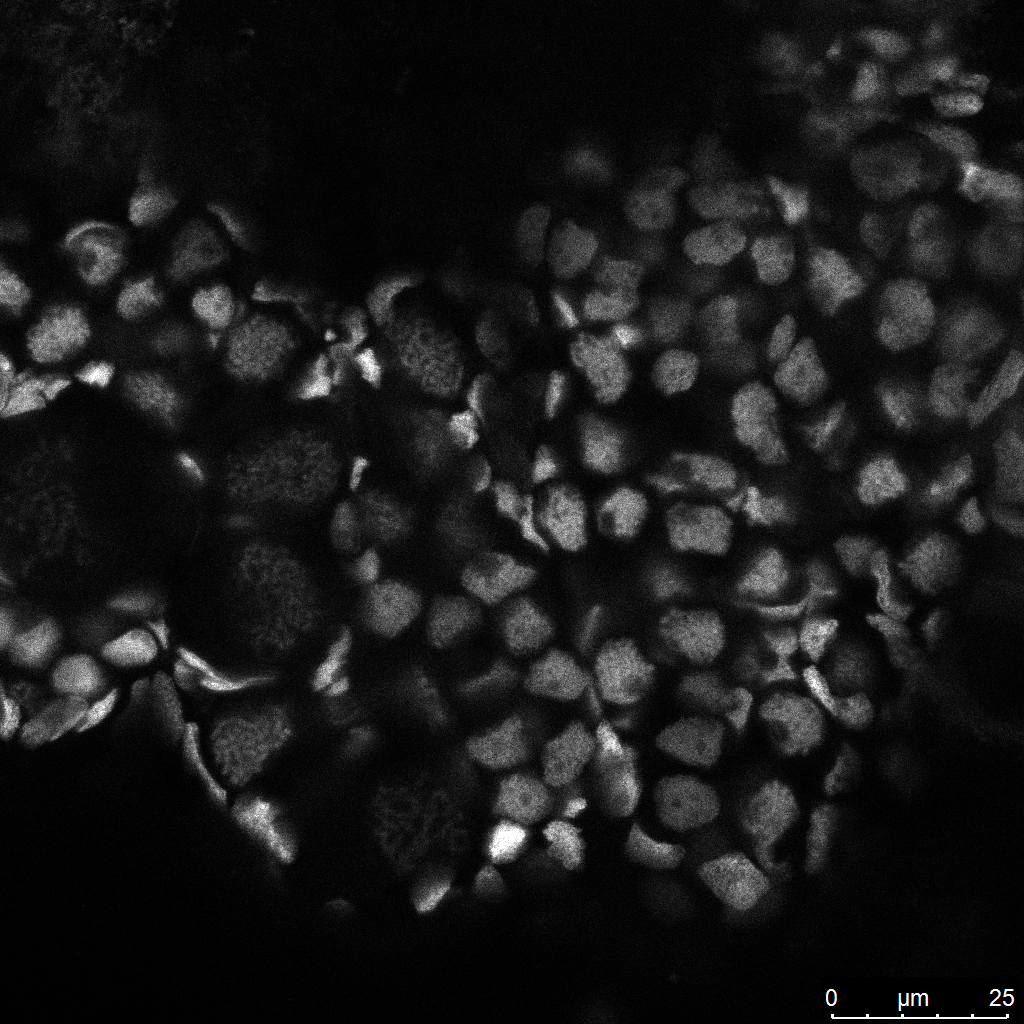

Supplement: Supplementary file 7 — Source data Fig. 3 [file 44319_2026_775_MOESM7_ESM.zip › Figure 3/Figure 3A/DAPI WT.tif]

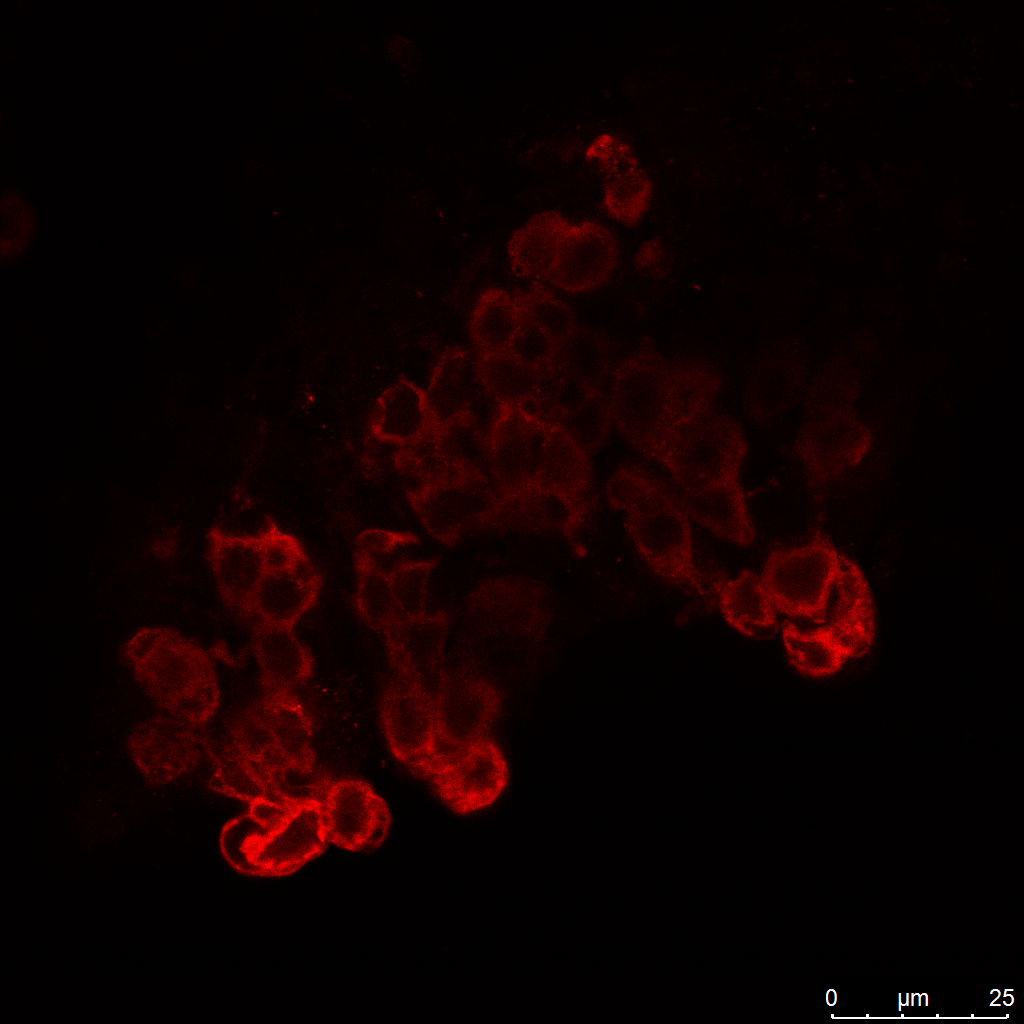

Supplement: Supplementary file 7 — Source data Fig. 3 [file 44319_2026_775_MOESM7_ESM.zip › Figure 3/Figure 3A/Ddx4 hom.tif]

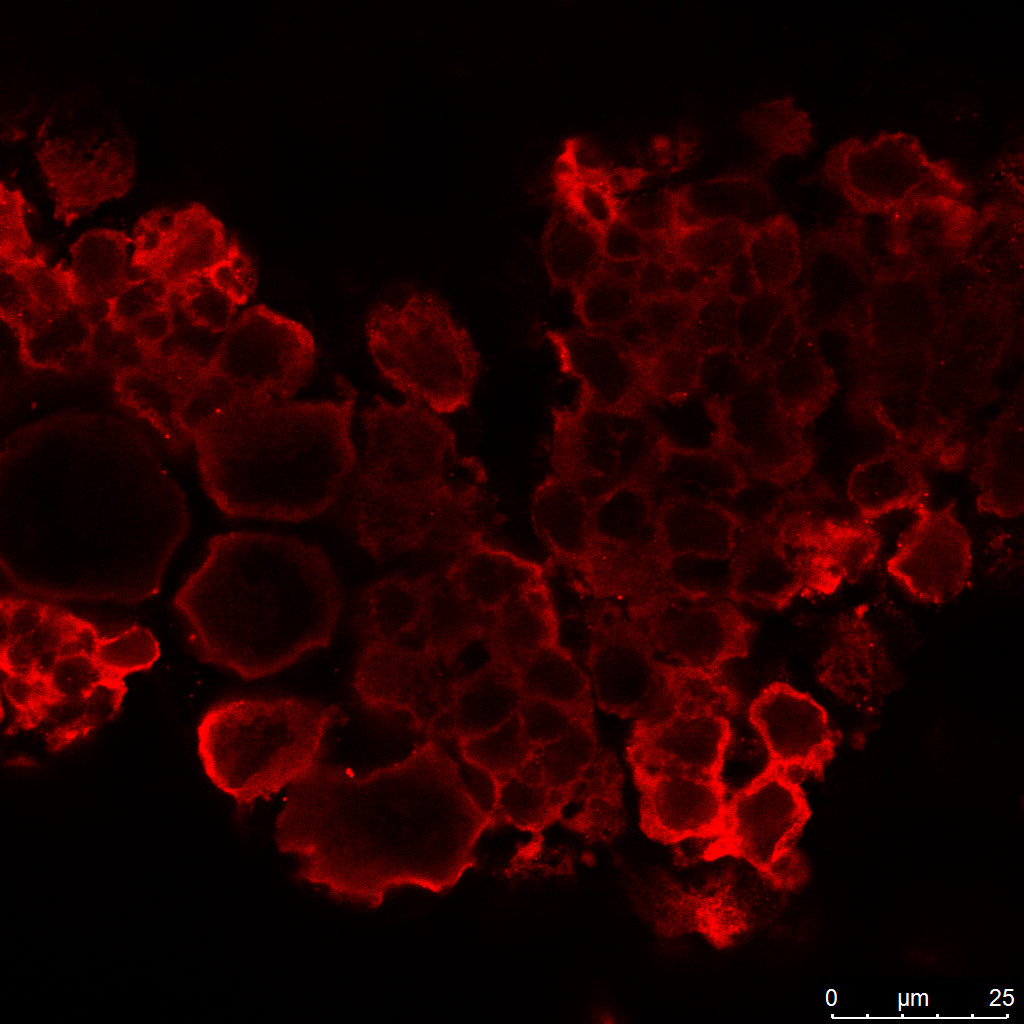

Supplement: Supplementary file 7 — Source data Fig. 3 [file 44319_2026_775_MOESM7_ESM.zip › Figure 3/Figure 3A/Ddx4 WT.tif]

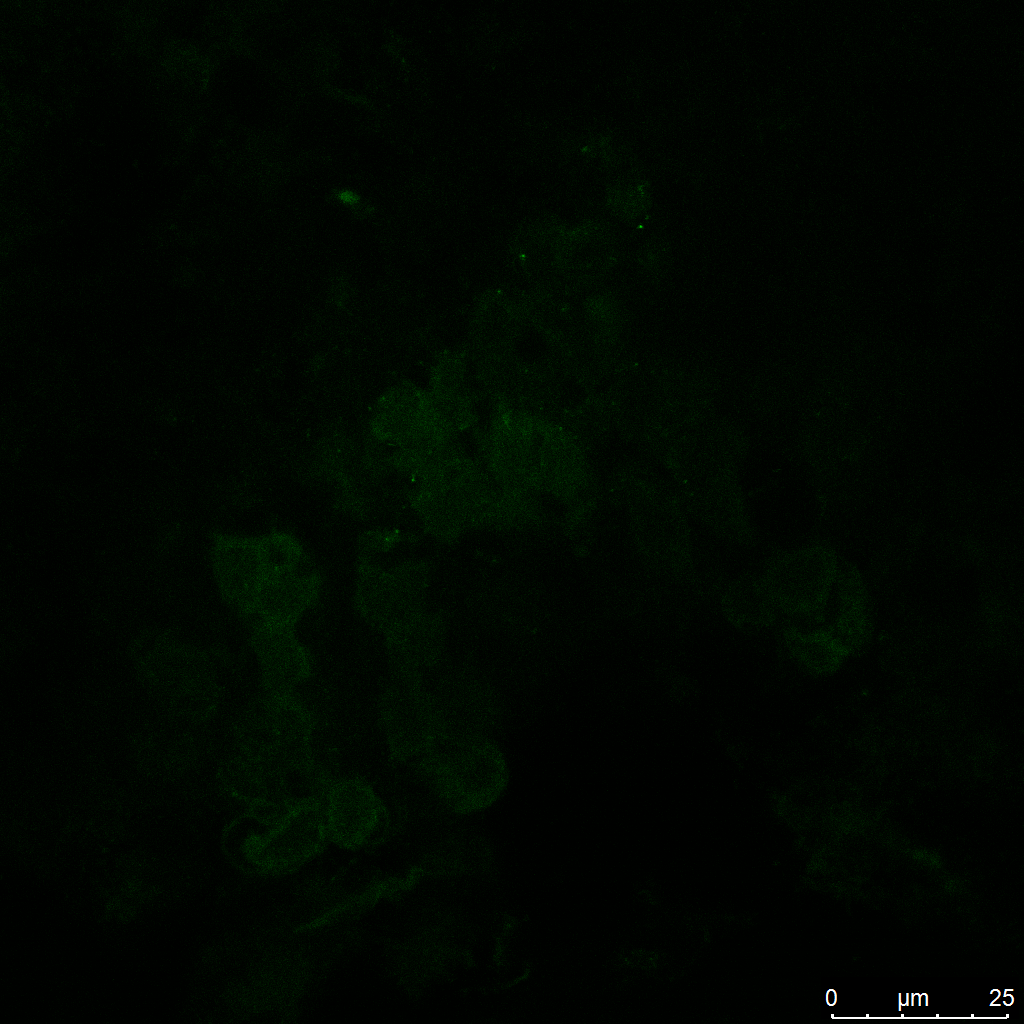

Supplement: Supplementary file 7 — Source data Fig. 3 [file 44319_2026_775_MOESM7_ESM.zip › Figure 3/Figure 3A/GFP hom.tif]

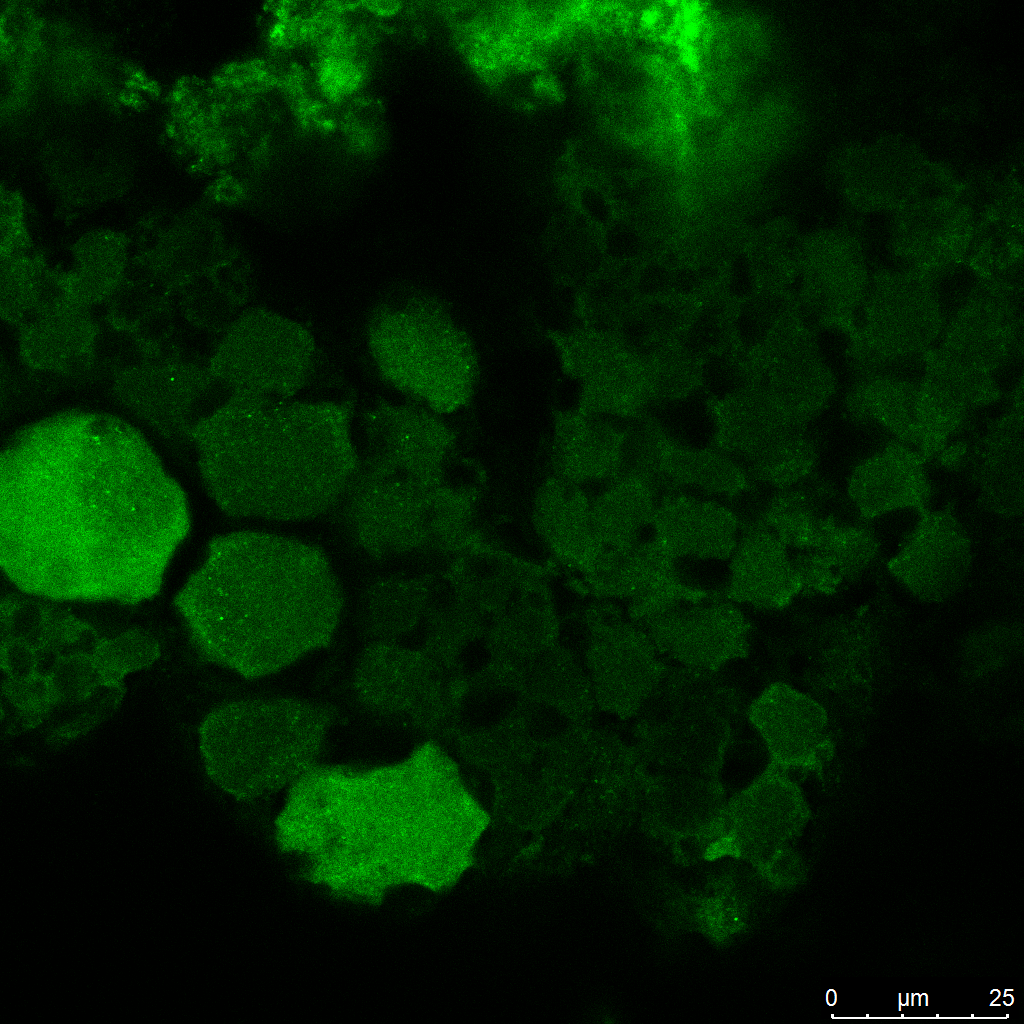

Supplement: Supplementary file 7 — Source data Fig. 3 [file 44319_2026_775_MOESM7_ESM.zip › Figure 3/Figure 3A/GFP WT.tif]

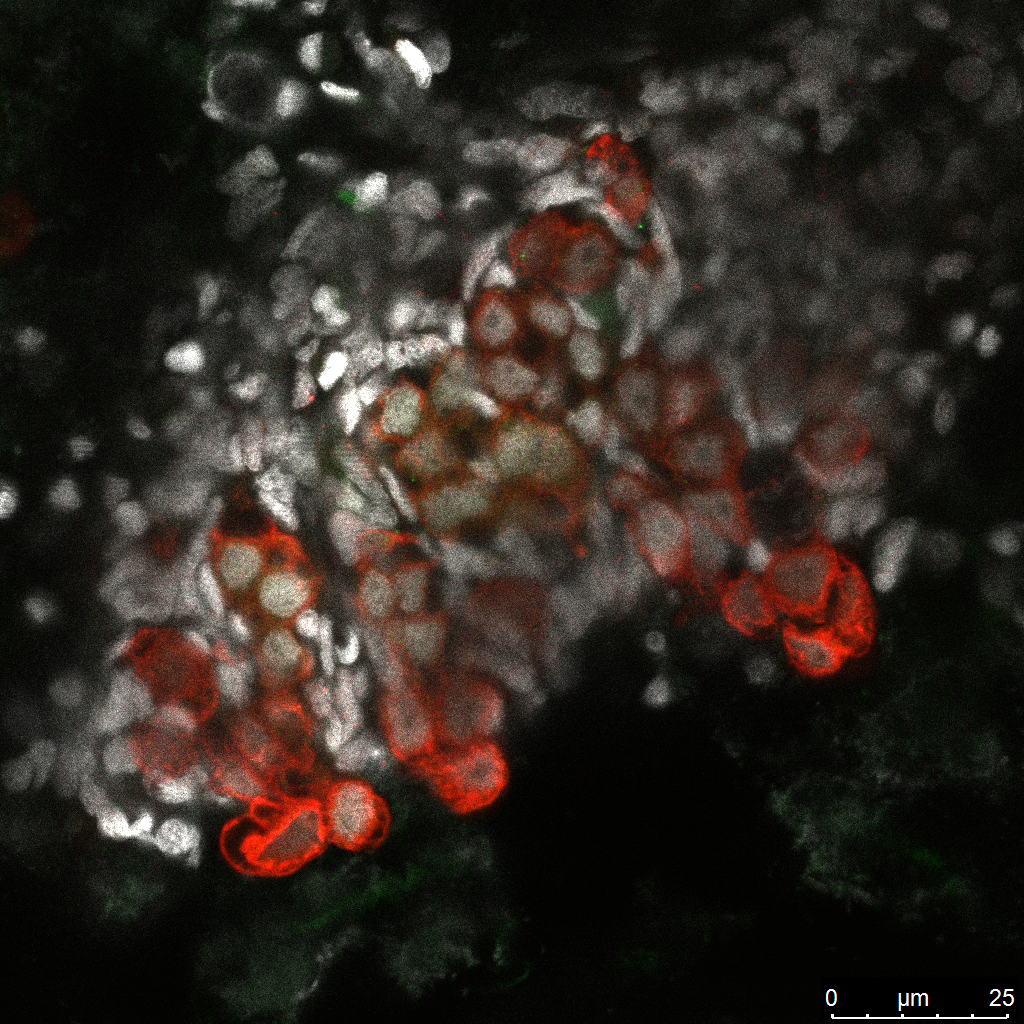

Supplement: Supplementary file 7 — Source data Fig. 3 [file 44319_2026_775_MOESM7_ESM.zip › Figure 3/Figure 3A/Merge hom.tif]

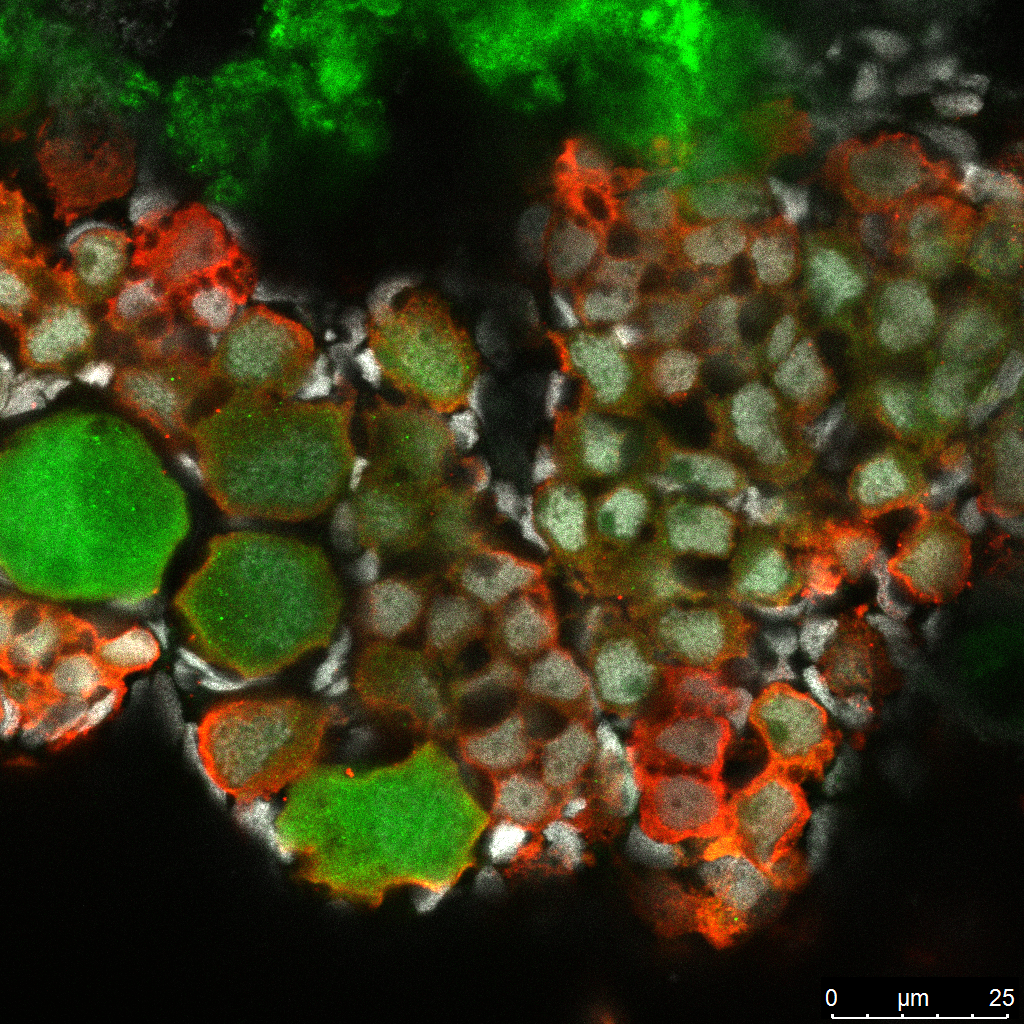

Supplement: Supplementary file 7 — Source data Fig. 3 [file 44319_2026_775_MOESM7_ESM.zip › Figure 3/Figure 3A/Merge WT.tif]

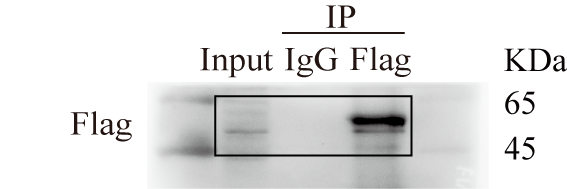

Supplement: Supplementary file 7 — Source data Fig. 3 [file 44319_2026_775_MOESM7_ESM.zip › Figure 3/Figure 3C/Flag.tif]

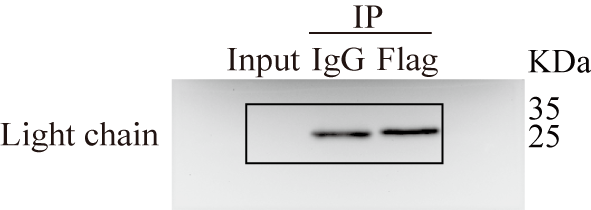

Supplement: Supplementary file 7 — Source data Fig. 3 [file 44319_2026_775_MOESM7_ESM.zip › Figure 3/Figure 3C/Light chain.tif]

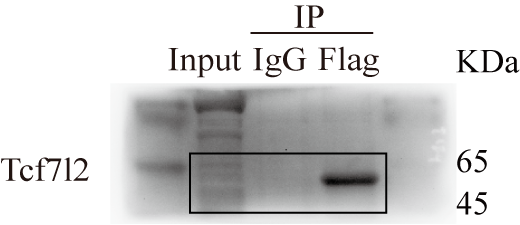

Supplement: Supplementary file 7 — Source data Fig. 3 [file 44319_2026_775_MOESM7_ESM.zip › Figure 3/Figure 3C/Tcf7l2.tif]

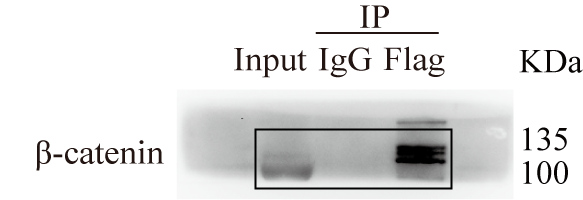

Supplement: Supplementary file 7 — Source data Fig. 3 [file 44319_2026_775_MOESM7_ESM.zip › Figure 3/Figure 3C/β-catenin.tif]

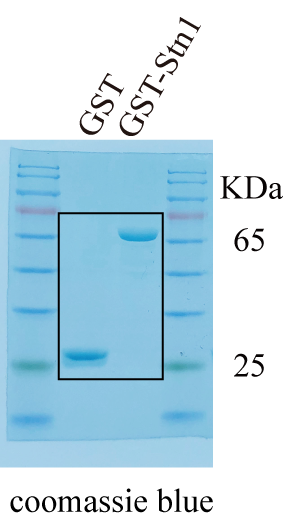

Supplement: Supplementary file 7 — Source data Fig. 3 [file 44319_2026_775_MOESM7_ESM.zip › Figure 3/Figure 3D/coomassie blue.tif]

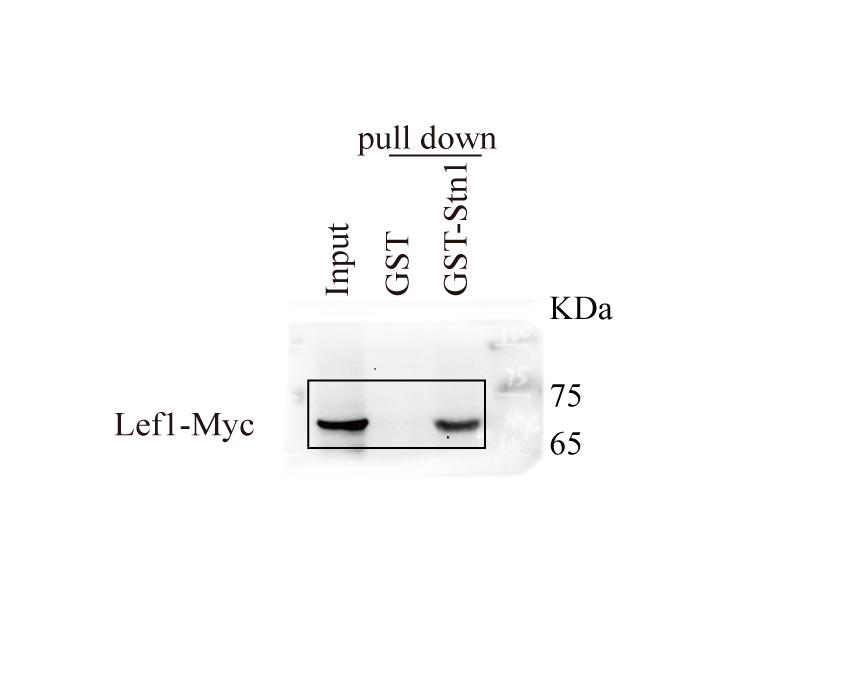

Supplement: Supplementary file 7 — Source data Fig. 3 [file 44319_2026_775_MOESM7_ESM.zip › Figure 3/Figure 3D/Lef1-Myc.tif]

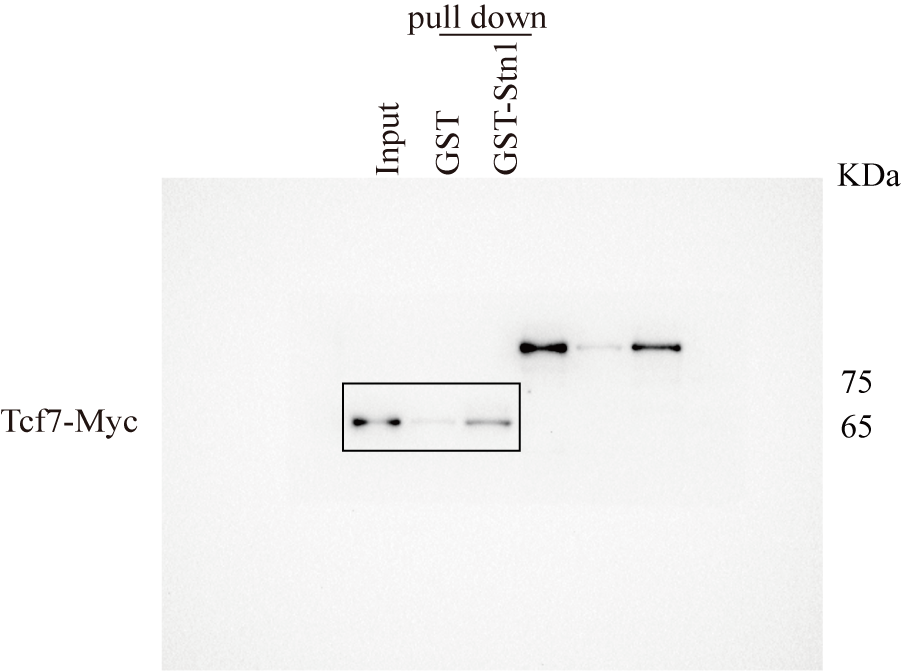

Supplement: Supplementary file 7 — Source data Fig. 3 [file 44319_2026_775_MOESM7_ESM.zip › Figure 3/Figure 3D/Tcf7-Myc.tif]

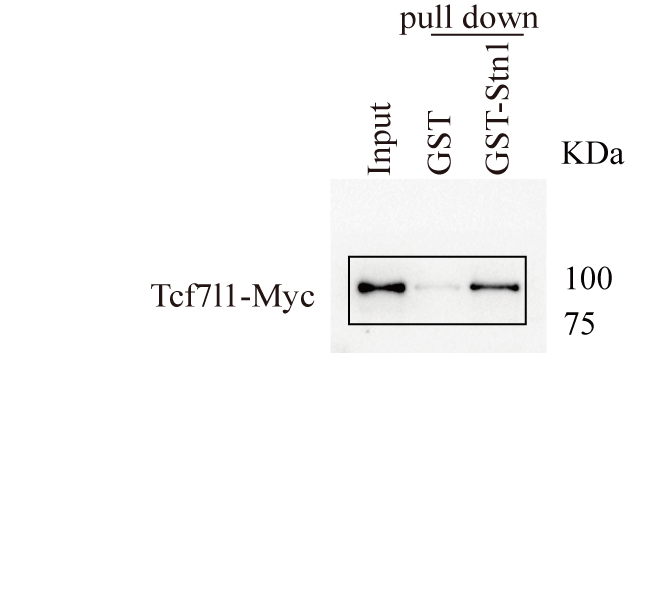

Supplement: Supplementary file 7 — Source data Fig. 3 [file 44319_2026_775_MOESM7_ESM.zip › Figure 3/Figure 3D/Tcf7l1-Myc.tif]

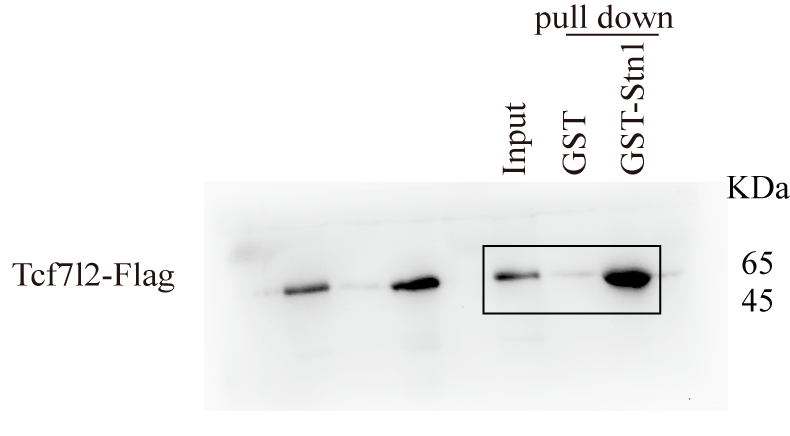

Supplement: Supplementary file 7 — Source data Fig. 3 [file 44319_2026_775_MOESM7_ESM.zip › Figure 3/Figure 3D/Tcf7l2-Flag.tif]

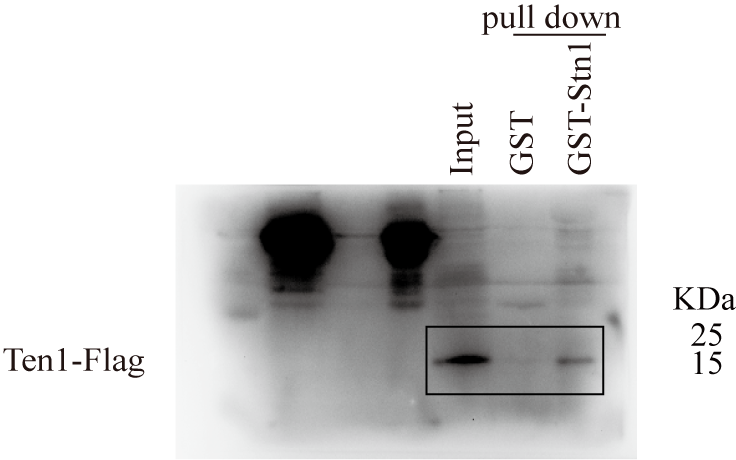

Supplement: Supplementary file 7 — Source data Fig. 3 [file 44319_2026_775_MOESM7_ESM.zip › Figure 3/Figure 3D/Ten1-Flag.tif]

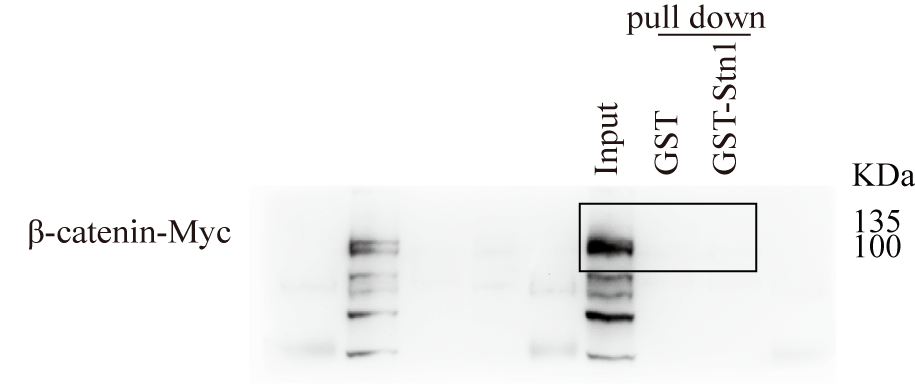

Supplement: Supplementary file 7 — Source data Fig. 3 [file 44319_2026_775_MOESM7_ESM.zip › Figure 3/Figure 3D/β-catenin-Myc.tif]
