# Supplementary figures and images for: Stn1 promotes zebrafish oocyte development via amplifying Wnt/β-catenin signaling (part 2 of 5)
Source: EMBO Rep. 2026 Apr 17;27(12):3252–76. doi: 10.1038/s44319-026-00775-8 (PMC13304171; doi:10.1038/s44319-026-00775-8)

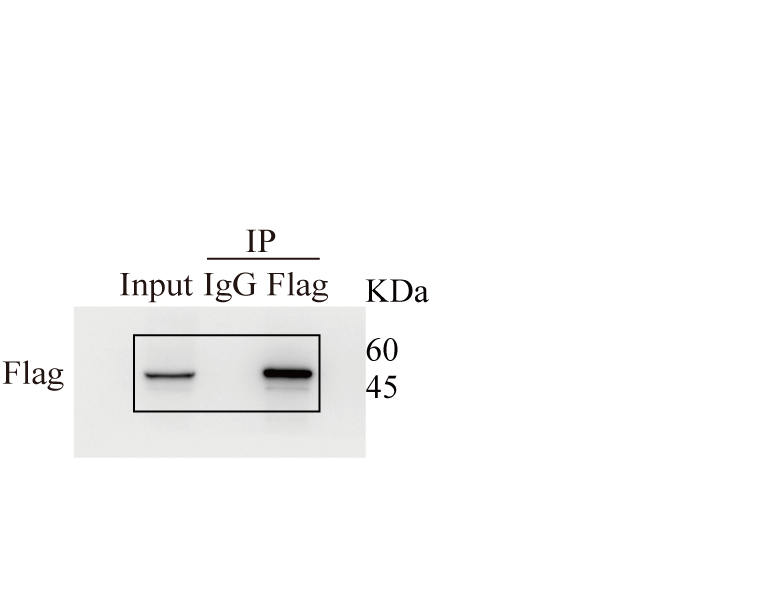

Supplement: Supplementary file 7 — Source data Fig. 3 [file 44319_2026_775_MOESM7_ESM.zip › Figure 3/Figure 3E/Flag.tif]

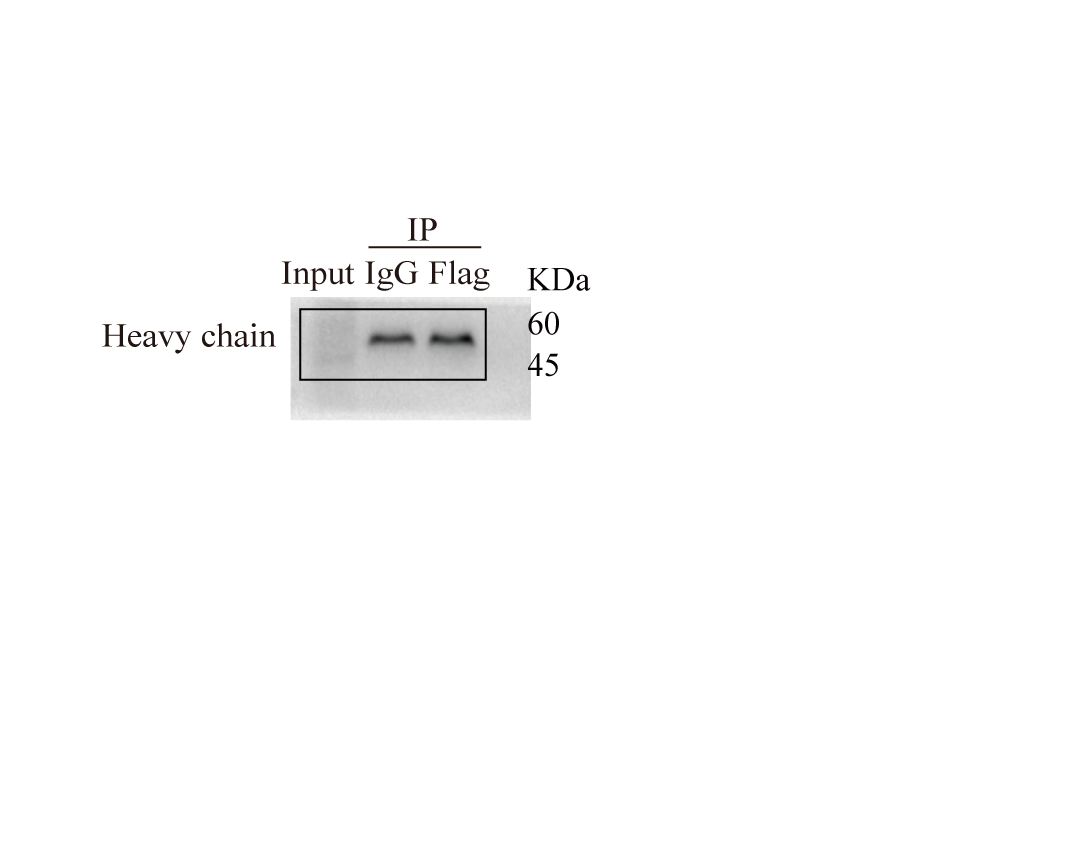

Supplement: Supplementary file 7 — Source data Fig. 3 [file 44319_2026_775_MOESM7_ESM.zip › Figure 3/Figure 3E/Heavy chain.tif]

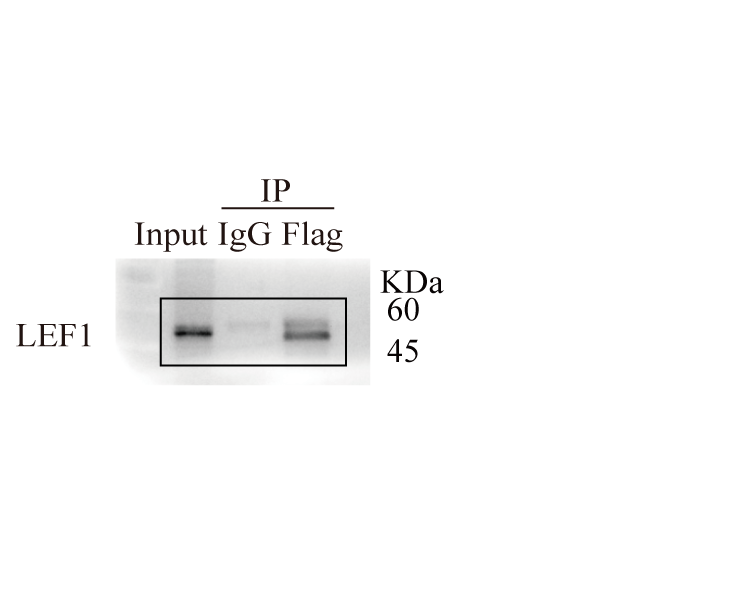

Supplement: Supplementary file 7 — Source data Fig. 3 [file 44319_2026_775_MOESM7_ESM.zip › Figure 3/Figure 3E/LEF1.tif]

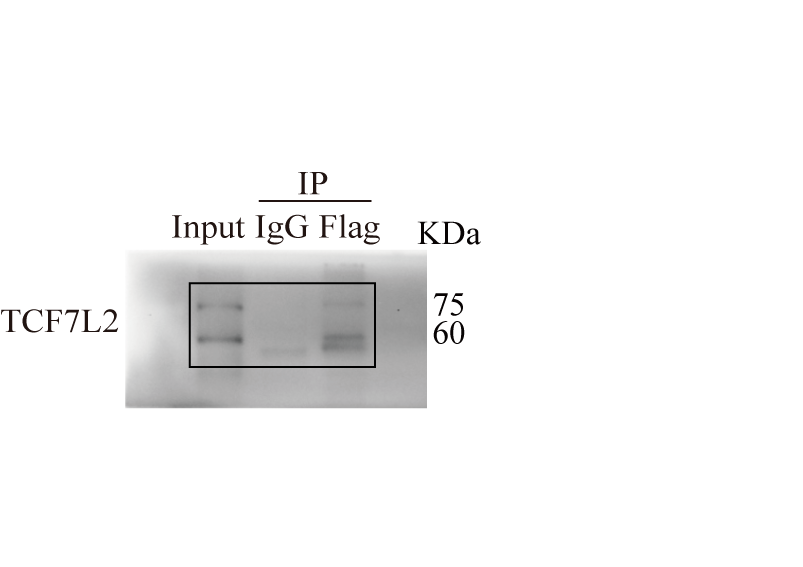

Supplement: Supplementary file 7 — Source data Fig. 3 [file 44319_2026_775_MOESM7_ESM.zip › Figure 3/Figure 3E/TCF7L2.tif]

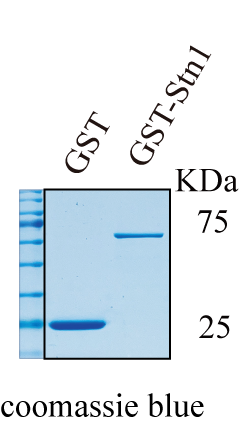

Supplement: Supplementary file 7 — Source data Fig. 3 [file 44319_2026_775_MOESM7_ESM.zip › Figure 3/Figure 3G/coomassie blue.tif]

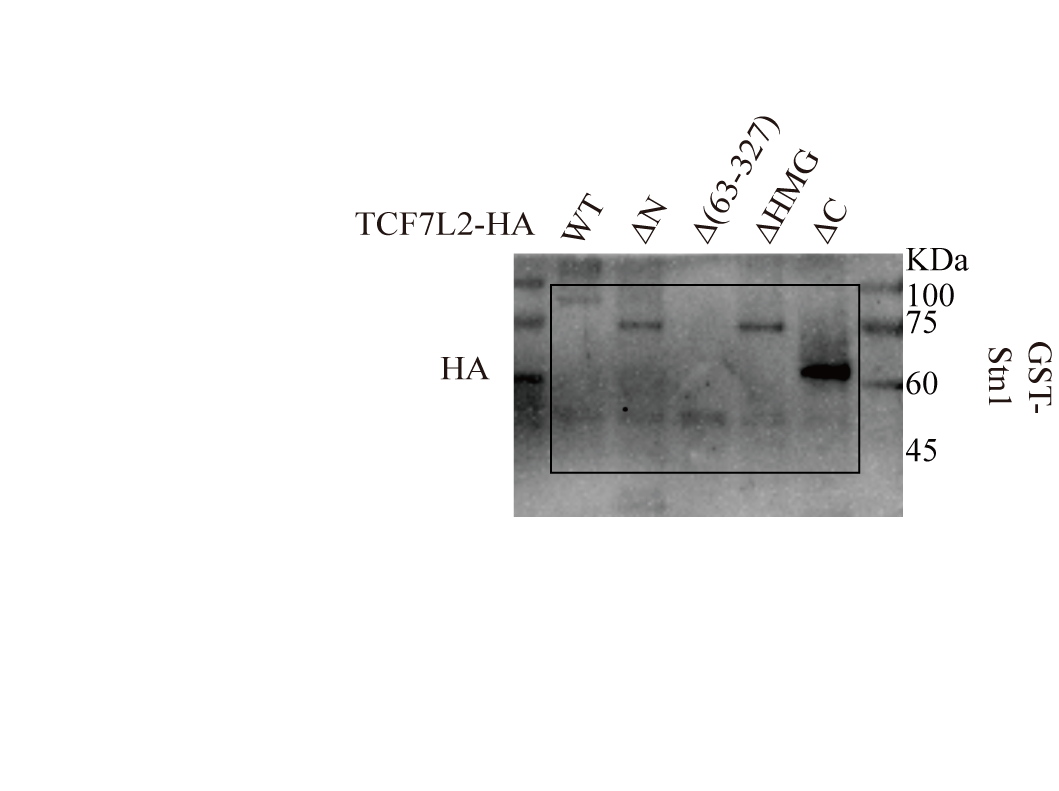

Supplement: Supplementary file 7 — Source data Fig. 3 [file 44319_2026_775_MOESM7_ESM.zip › Figure 3/Figure 3G/HA GST-Stn1.tif]

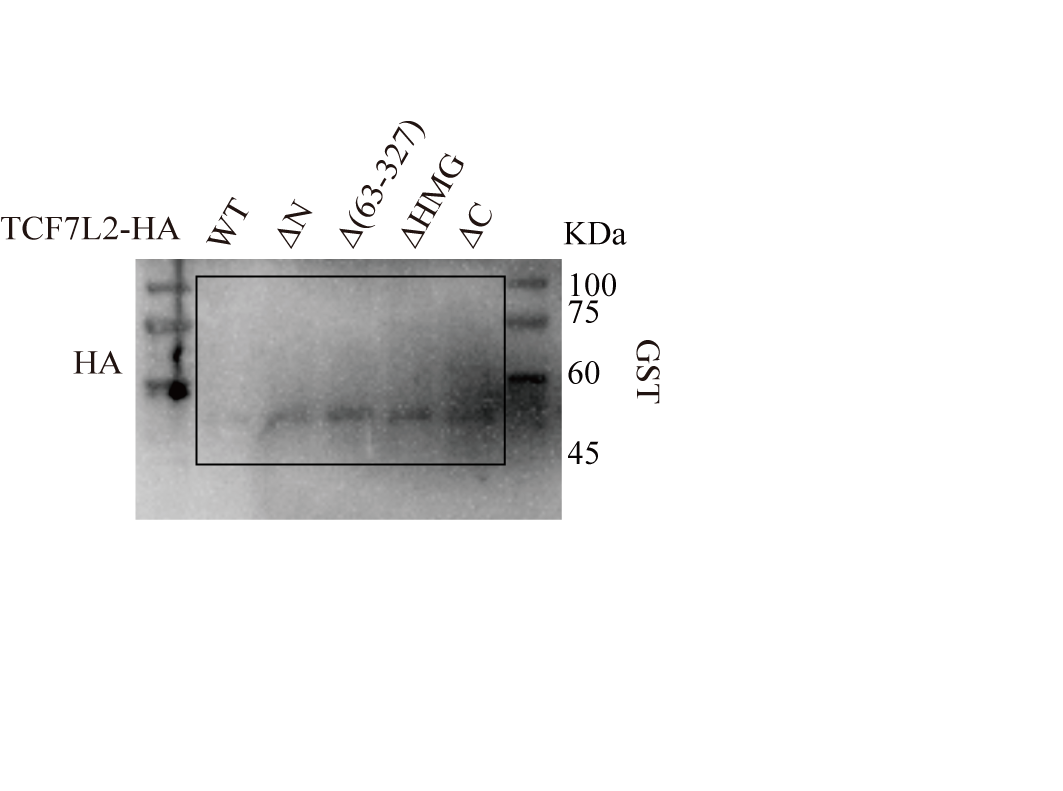

Supplement: Supplementary file 7 — Source data Fig. 3 [file 44319_2026_775_MOESM7_ESM.zip › Figure 3/Figure 3G/HA GST.tif]

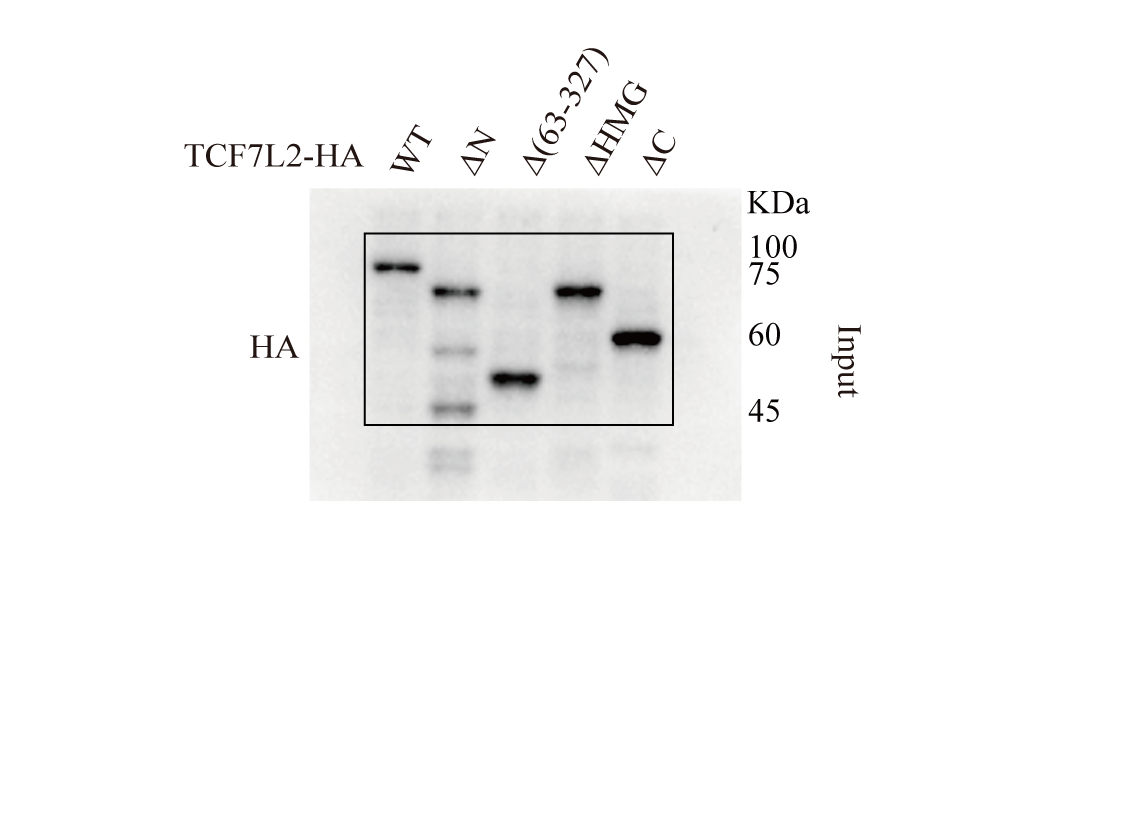

Supplement: Supplementary file 7 — Source data Fig. 3 [file 44319_2026_775_MOESM7_ESM.zip › Figure 3/Figure 3G/HA Input.tif]

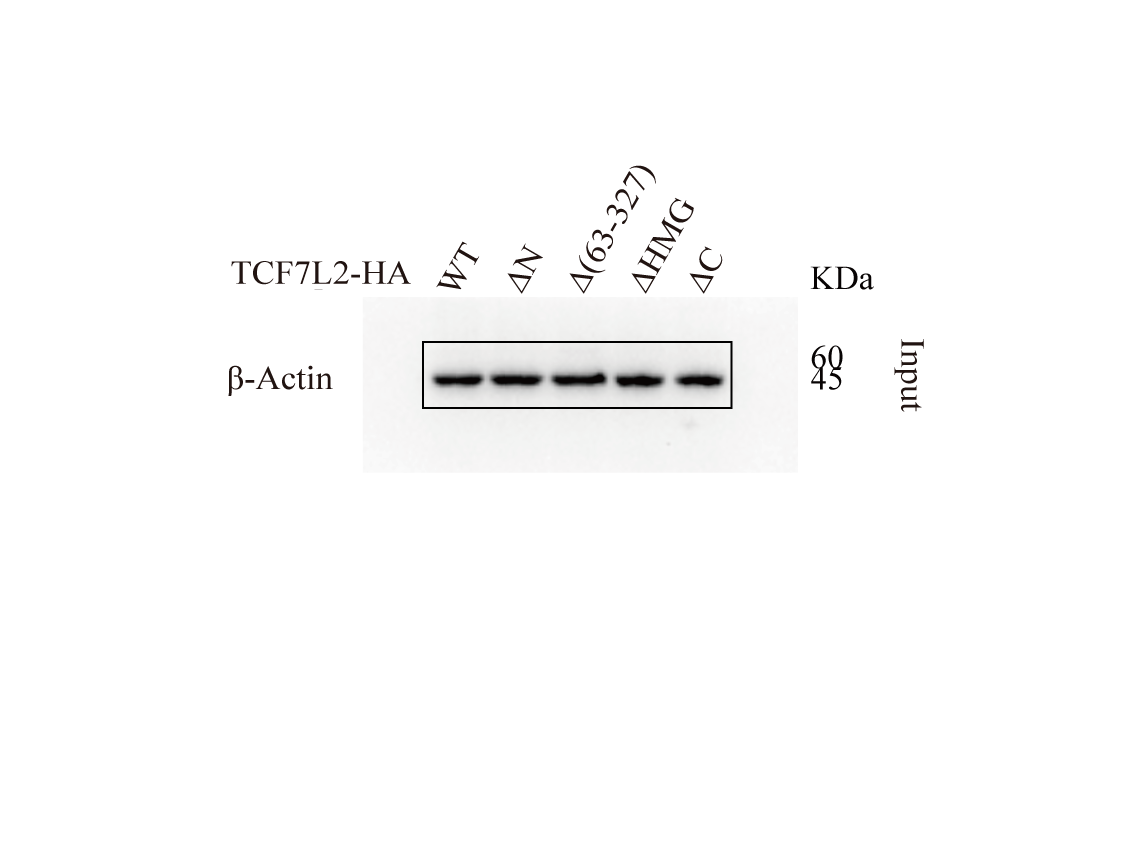

Supplement: Supplementary file 7 — Source data Fig. 3 [file 44319_2026_775_MOESM7_ESM.zip › Figure 3/Figure 3G/β-Actin Input.tif]

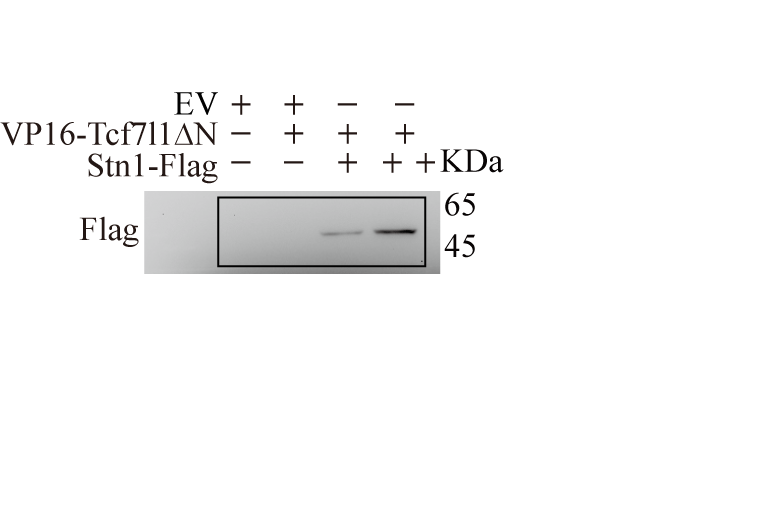

Supplement: Supplementary file 7 — Source data Fig. 3 [file 44319_2026_775_MOESM7_ESM.zip › Figure 3/Figure 3H/Figure 3H western blot/Flag.tif]

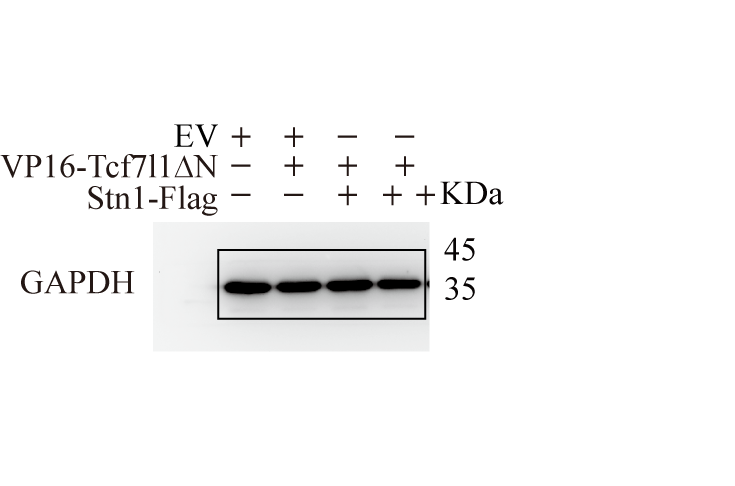

Supplement: Supplementary file 7 — Source data Fig. 3 [file 44319_2026_775_MOESM7_ESM.zip › Figure 3/Figure 3H/Figure 3H western blot/GAPDH.tif]

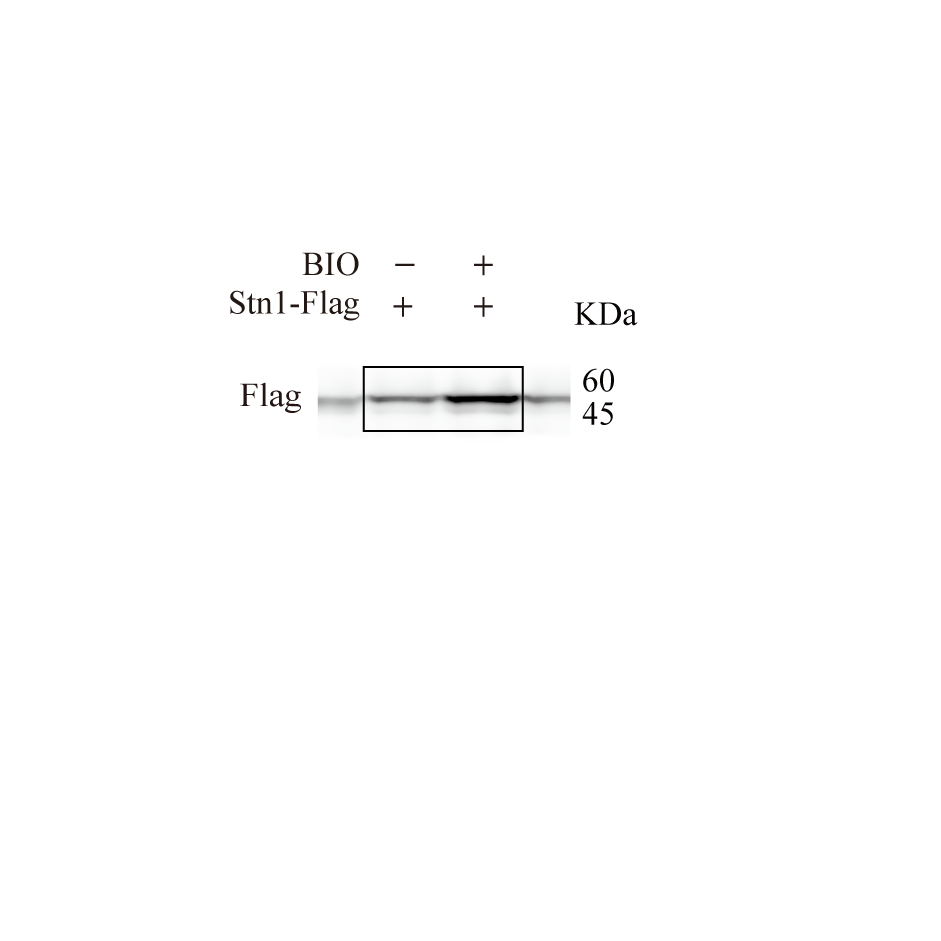

Supplement: Supplementary file 7 — Source data Fig. 3 [file 44319_2026_775_MOESM7_ESM.zip › Figure 3/Figure 3I/Figure 3I western blot/Flag.tif]

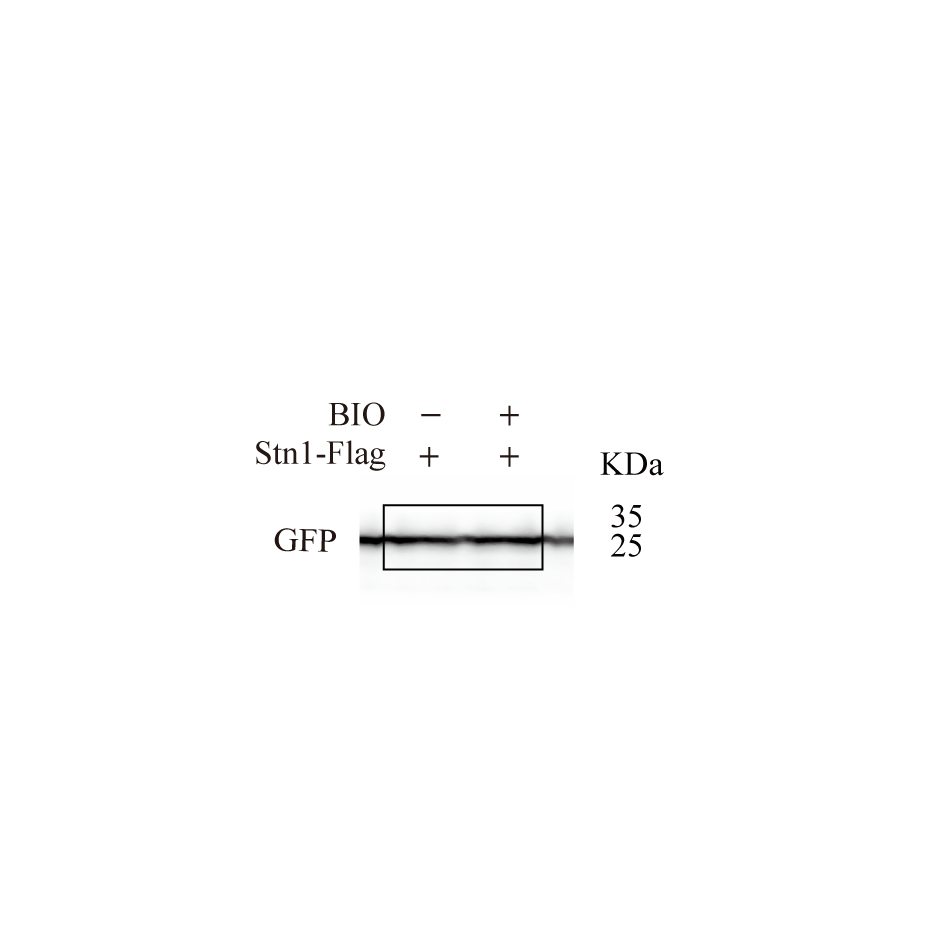

Supplement: Supplementary file 7 — Source data Fig. 3 [file 44319_2026_775_MOESM7_ESM.zip › Figure 3/Figure 3I/Figure 3I western blot/GFP.tif]

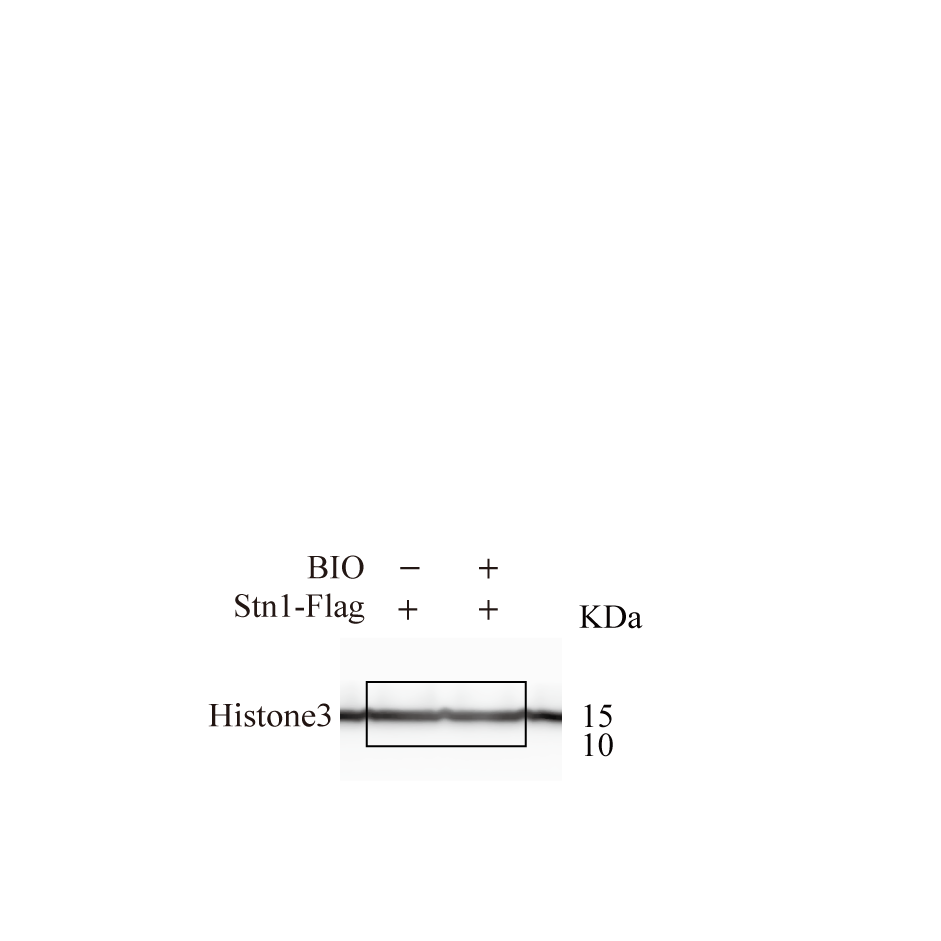

Supplement: Supplementary file 7 — Source data Fig. 3 [file 44319_2026_775_MOESM7_ESM.zip › Figure 3/Figure 3I/Figure 3I western blot/Histone 3.tif]

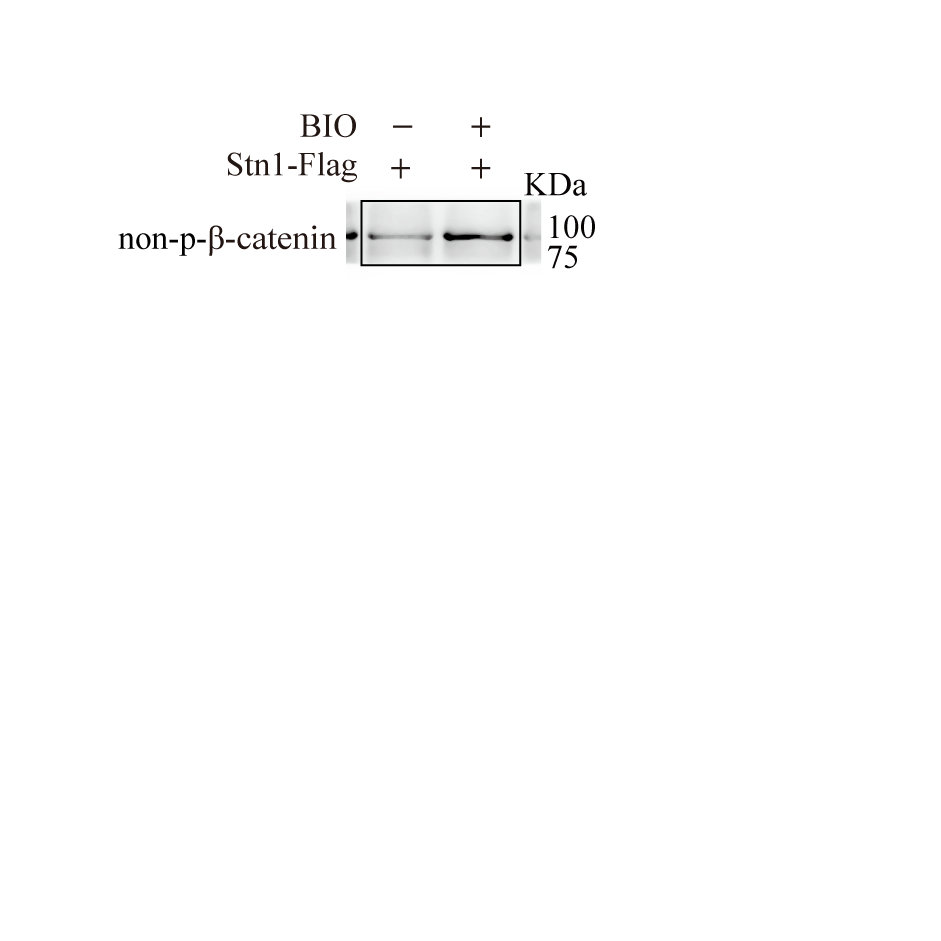

Supplement: Supplementary file 7 — Source data Fig. 3 [file 44319_2026_775_MOESM7_ESM.zip › Figure 3/Figure 3I/Figure 3I western blot/non-p-β-catenin.tif]

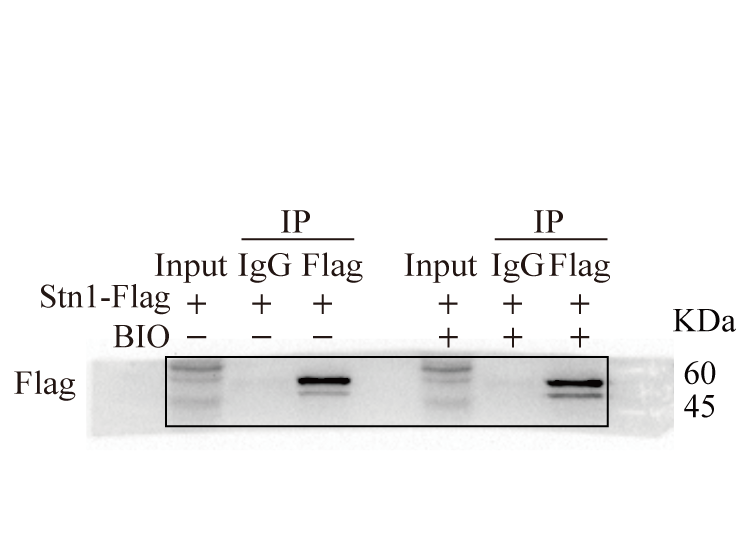

Supplement: Supplementary file 7 — Source data Fig. 3 [file 44319_2026_775_MOESM7_ESM.zip › Figure 3/Figure 3J/Flag right.tif]

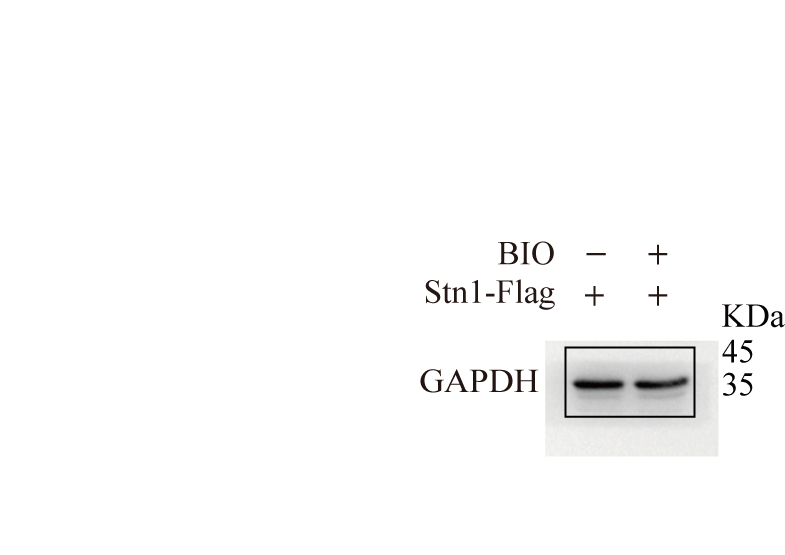

Supplement: Supplementary file 7 — Source data Fig. 3 [file 44319_2026_775_MOESM7_ESM.zip › Figure 3/Figure 3J/GAPDH left.tif]

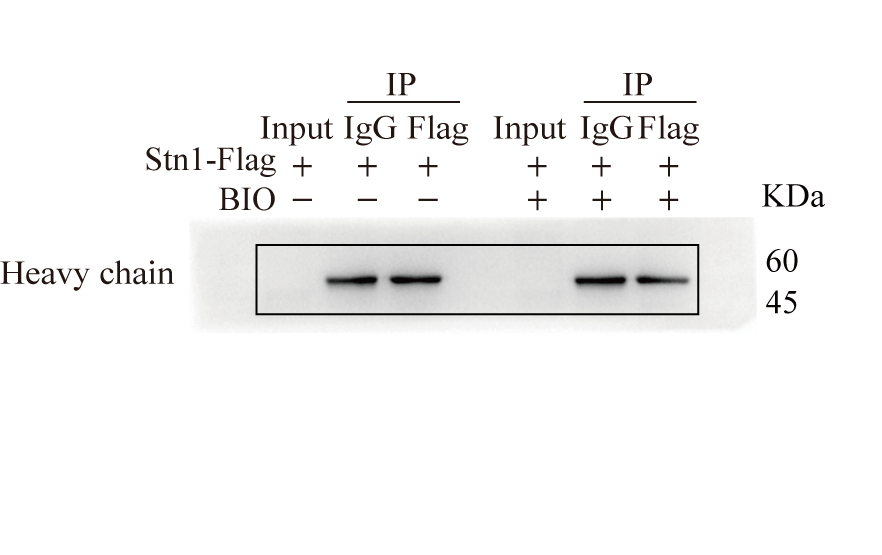

Supplement: Supplementary file 7 — Source data Fig. 3 [file 44319_2026_775_MOESM7_ESM.zip › Figure 3/Figure 3J/Heavy chain right.tif]

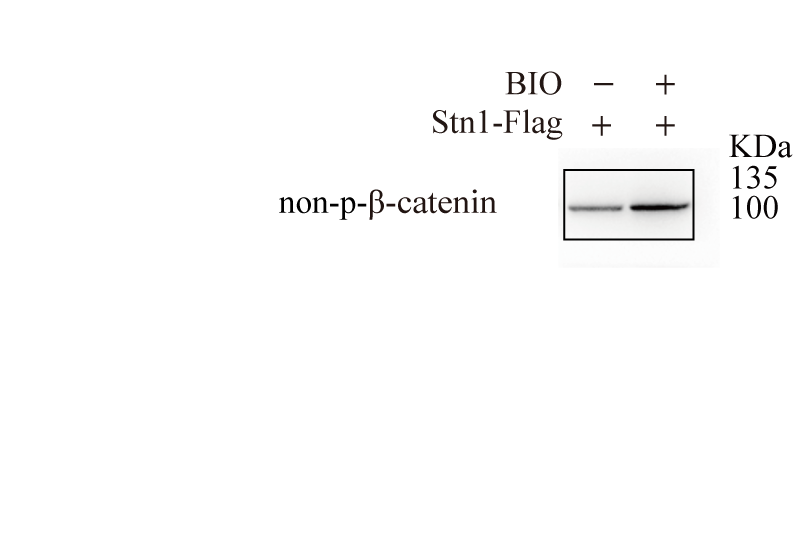

Supplement: Supplementary file 7 — Source data Fig. 3 [file 44319_2026_775_MOESM7_ESM.zip › Figure 3/Figure 3J/non-p-β-catenin left.tif]

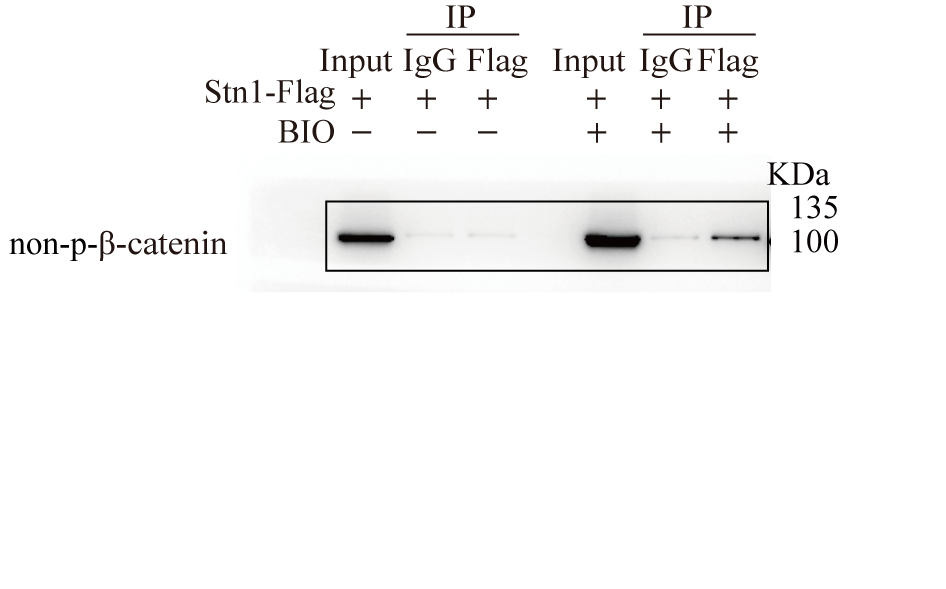

Supplement: Supplementary file 7 — Source data Fig. 3 [file 44319_2026_775_MOESM7_ESM.zip › Figure 3/Figure 3J/non-p-β-catenin right.tif]

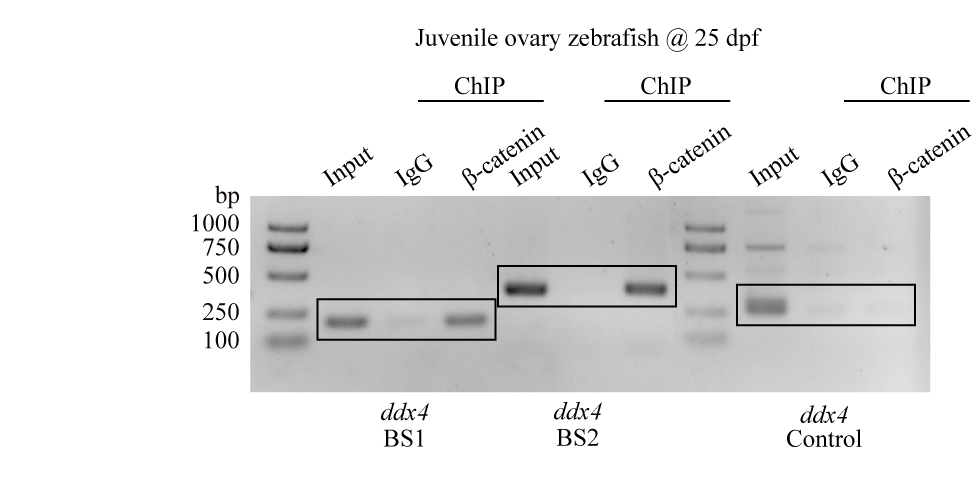

Supplement: Supplementary file 8 — Source data Fig. 4 [file 44319_2026_775_MOESM8_ESM.zip › Figure 4/Fig 4D/ddx4 25 dpf.tif]

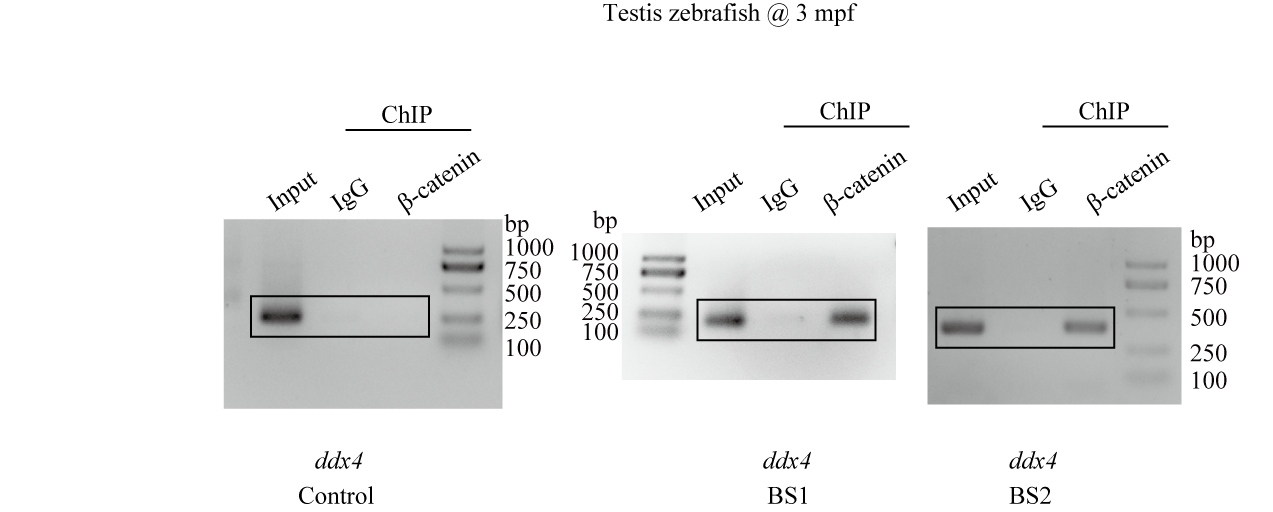

Supplement: Supplementary file 8 — Source data Fig. 4 [file 44319_2026_775_MOESM8_ESM.zip › Figure 4/Fig 4D/ddx4 3 mpf.tif]

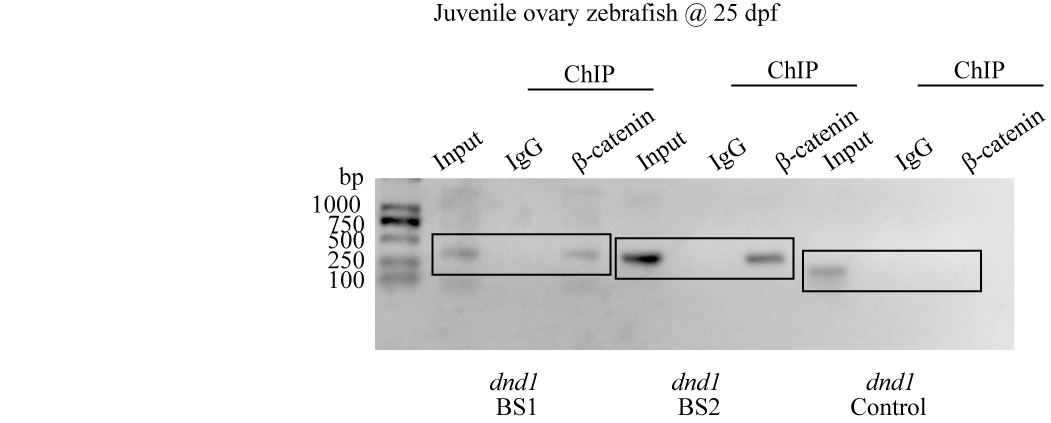

Supplement: Supplementary file 8 — Source data Fig. 4 [file 44319_2026_775_MOESM8_ESM.zip › Figure 4/Fig 4D/dnd1 25 dpf.tif]

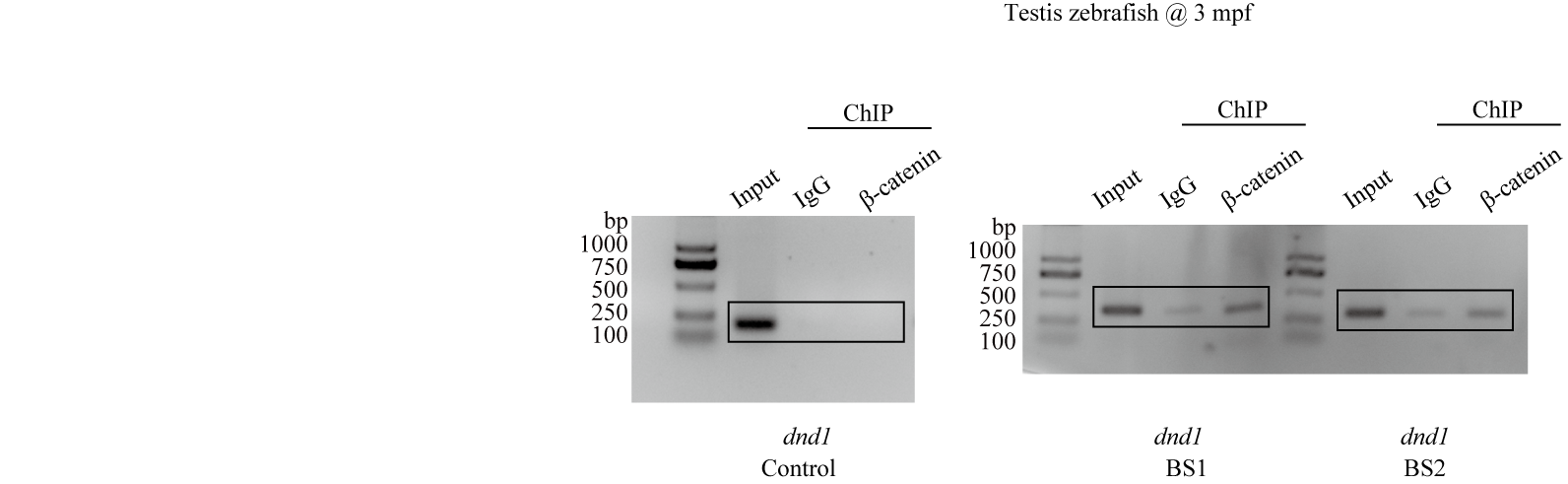

Supplement: Supplementary file 8 — Source data Fig. 4 [file 44319_2026_775_MOESM8_ESM.zip › Figure 4/Fig 4D/dnd1 3 mpf.tif]

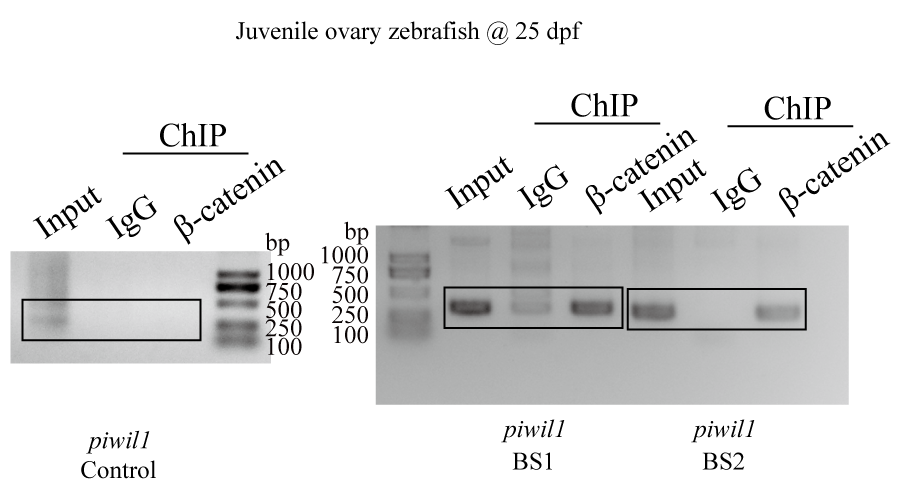

Supplement: Supplementary file 8 — Source data Fig. 4 [file 44319_2026_775_MOESM8_ESM.zip › Figure 4/Fig 4D/piwil1 25 dpf.tif]

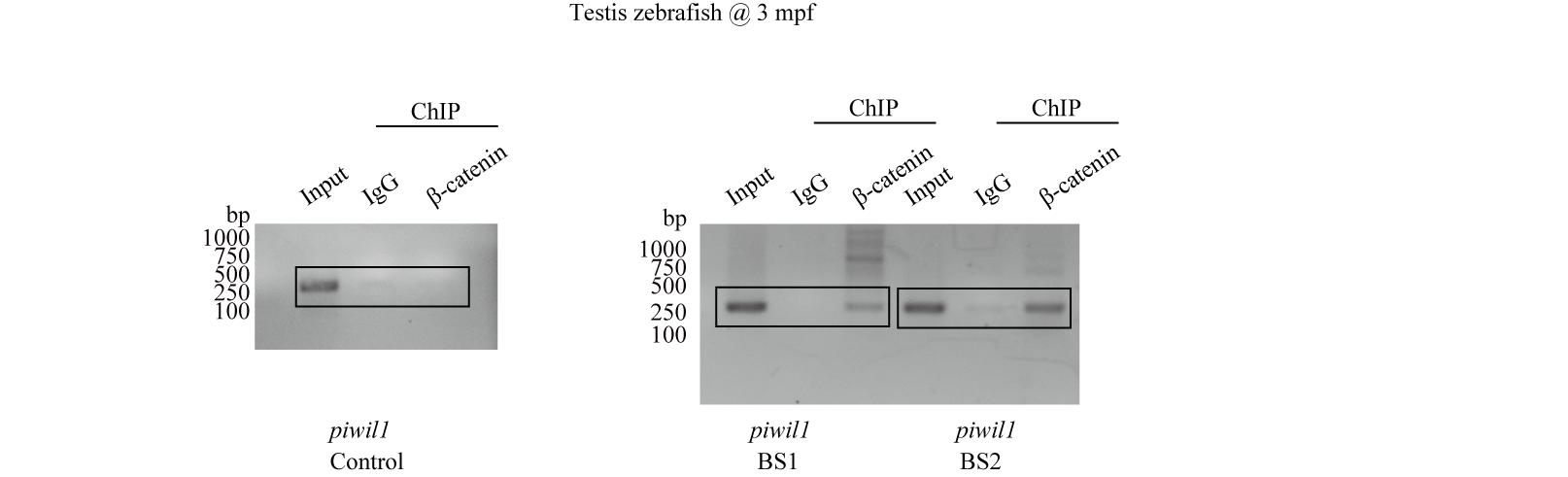

Supplement: Supplementary file 8 — Source data Fig. 4 [file 44319_2026_775_MOESM8_ESM.zip › Figure 4/Fig 4D/piwil1 3 mpf.tif]

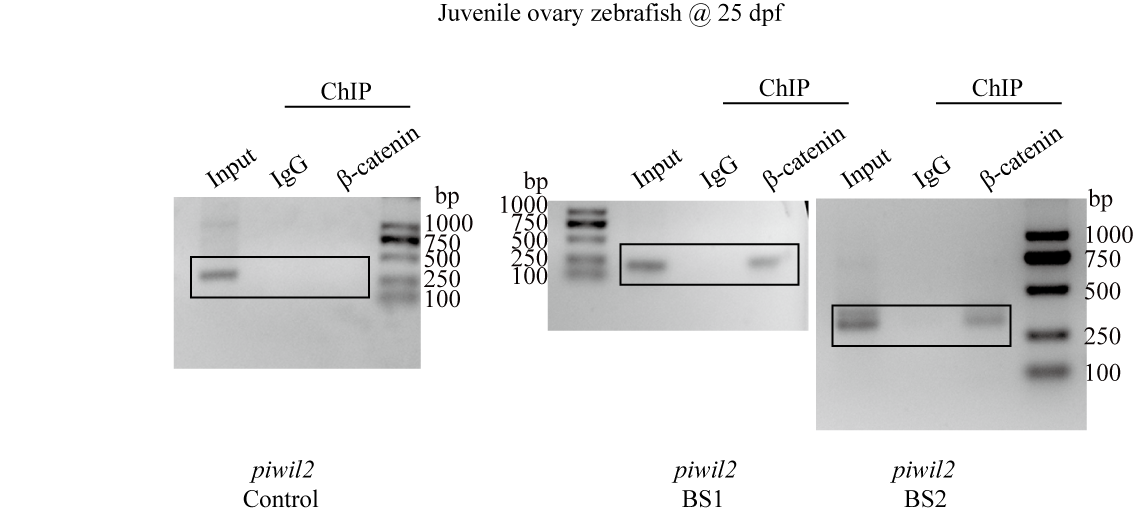

Supplement: Supplementary file 8 — Source data Fig. 4 [file 44319_2026_775_MOESM8_ESM.zip › Figure 4/Fig 4D/piwil2 25 dpf.tif]

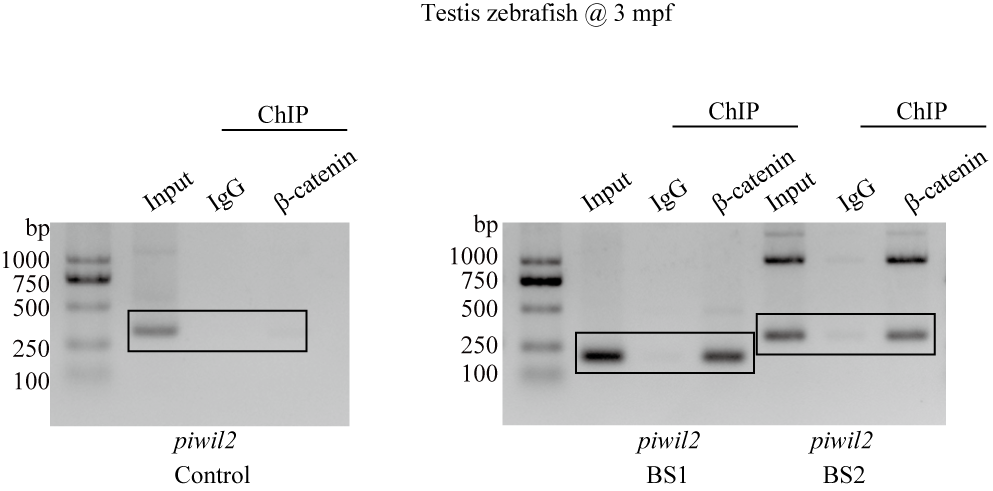

Supplement: Supplementary file 8 — Source data Fig. 4 [file 44319_2026_775_MOESM8_ESM.zip › Figure 4/Fig 4D/piwil2 3 mpf.tif]

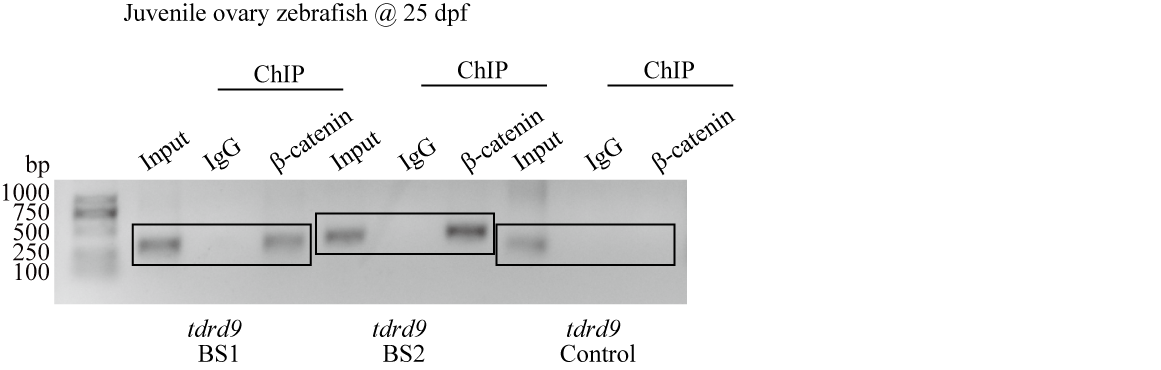

Supplement: Supplementary file 8 — Source data Fig. 4 [file 44319_2026_775_MOESM8_ESM.zip › Figure 4/Fig 4D/tdrd9 25 dpf.tif]

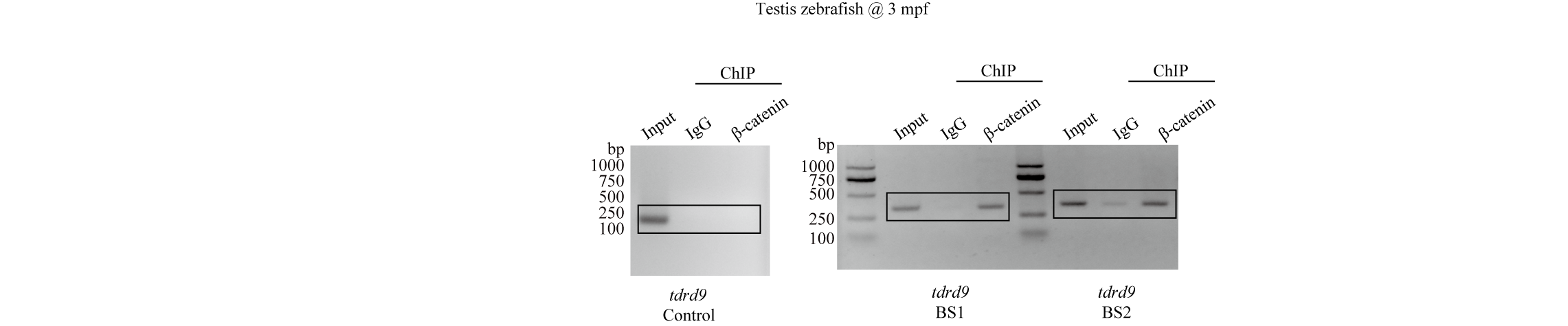

Supplement: Supplementary file 8 — Source data Fig. 4 [file 44319_2026_775_MOESM8_ESM.zip › Figure 4/Fig 4D/tdrd9 3 mpf.tif]

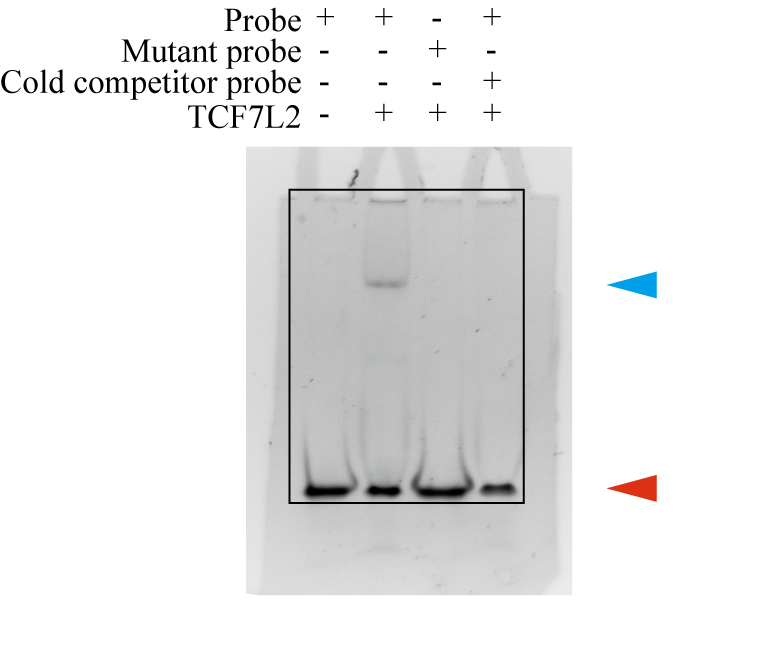

Supplement: Supplementary file 8 — Source data Fig. 4 [file 44319_2026_775_MOESM8_ESM.zip › Figure 4/Fig 4F/EMSA.tif]

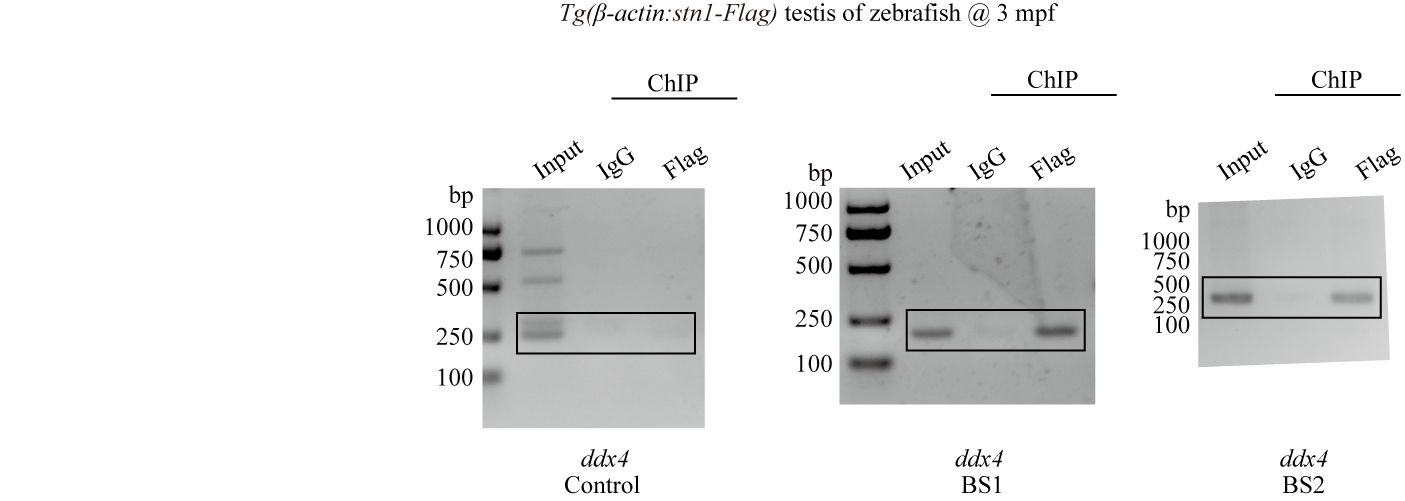

Supplement: Supplementary file 9 — Source data Fig. 5 [file 44319_2026_775_MOESM9_ESM.zip › Figure 5/Figure 5A/ddx4.tif]

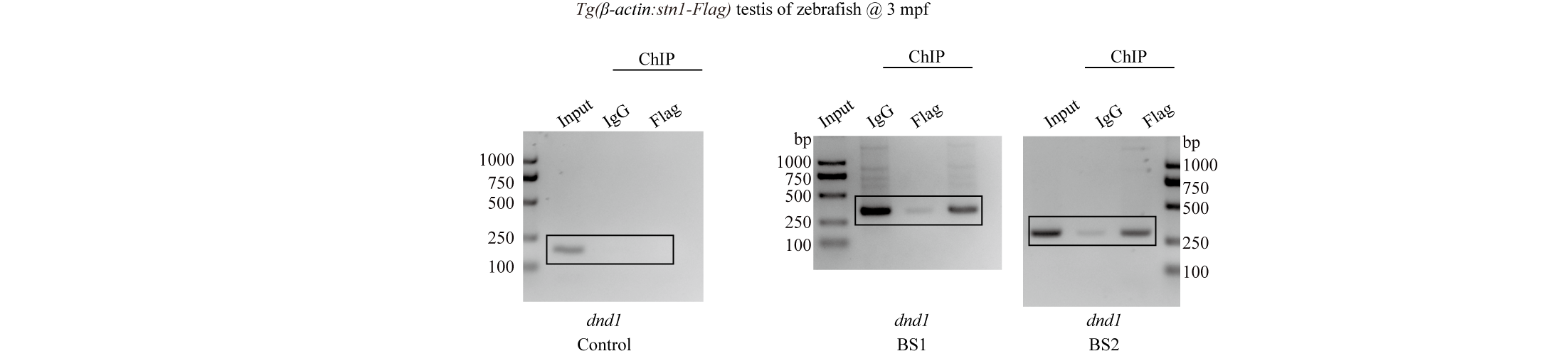

Supplement: Supplementary file 9 — Source data Fig. 5 [file 44319_2026_775_MOESM9_ESM.zip › Figure 5/Figure 5A/dnd1.tif]

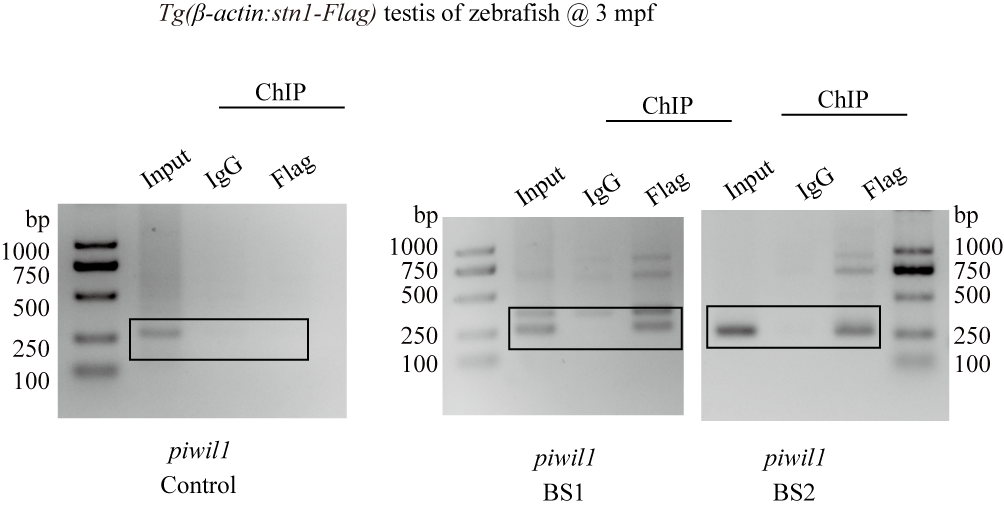

Supplement: Supplementary file 9 — Source data Fig. 5 [file 44319_2026_775_MOESM9_ESM.zip › Figure 5/Figure 5A/piwil1.tif]

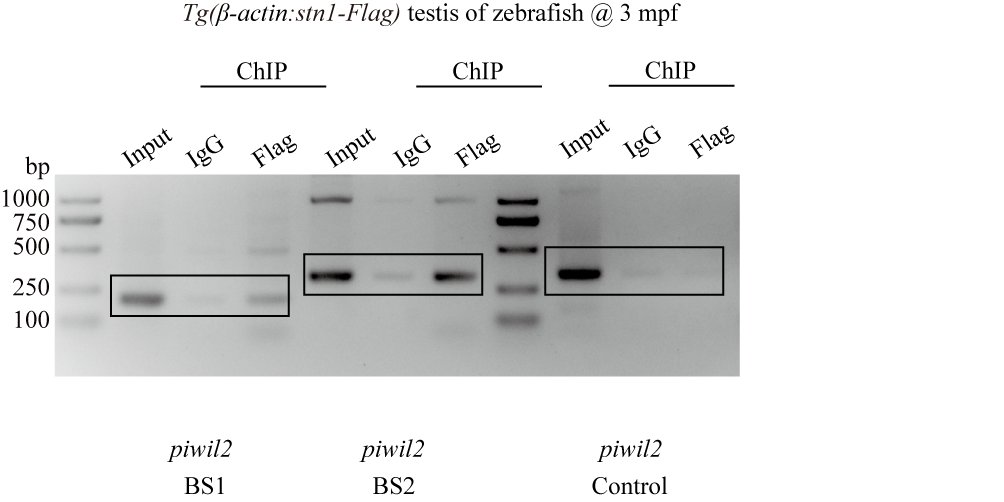

Supplement: Supplementary file 9 — Source data Fig. 5 [file 44319_2026_775_MOESM9_ESM.zip › Figure 5/Figure 5A/piwil2.tif]

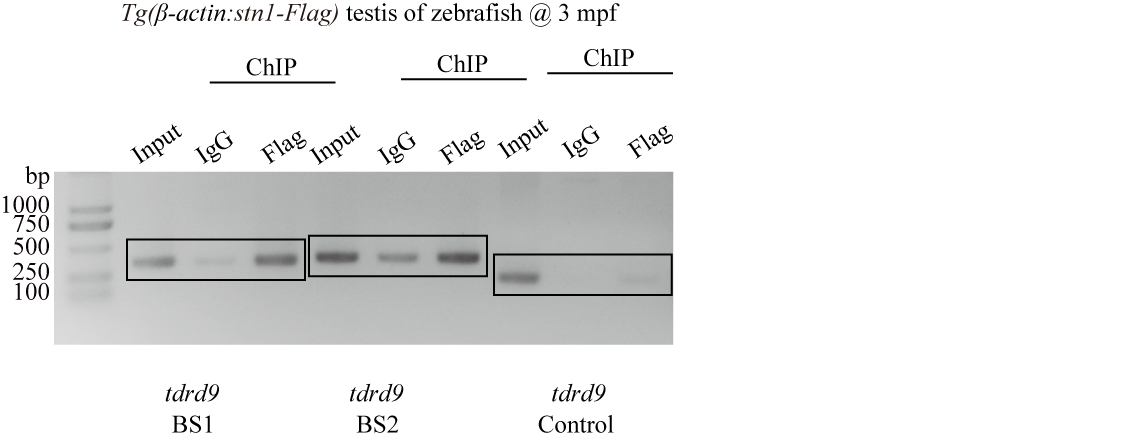

Supplement: Supplementary file 9 — Source data Fig. 5 [file 44319_2026_775_MOESM9_ESM.zip › Figure 5/Figure 5A/tdrd9.tif]

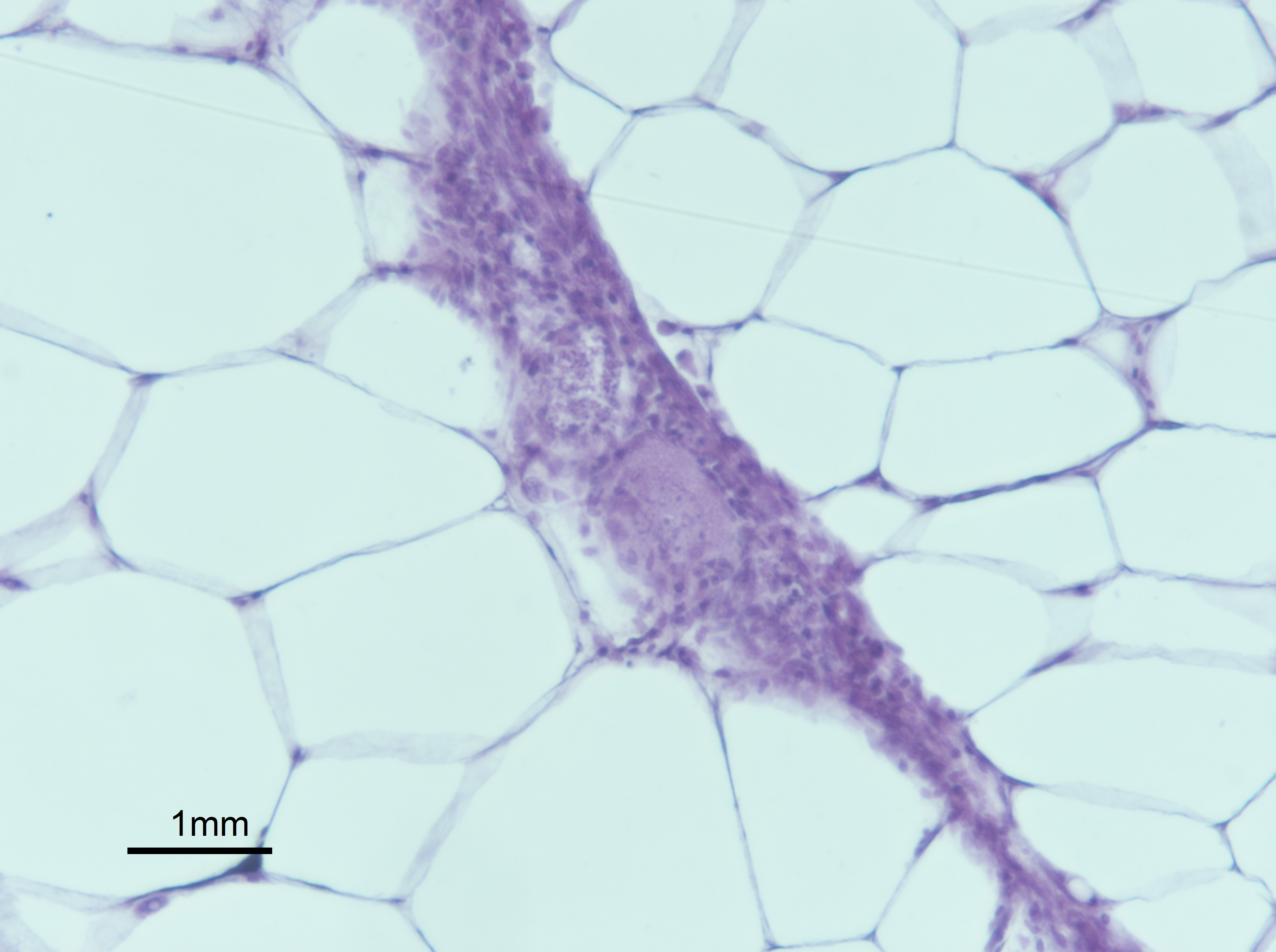

Supplement: Supplementary file 9 — Source data Fig. 5 [file 44319_2026_775_MOESM9_ESM.zip › Figure 5/Figure 5D/+7 line 45 dpf-hom.tif]

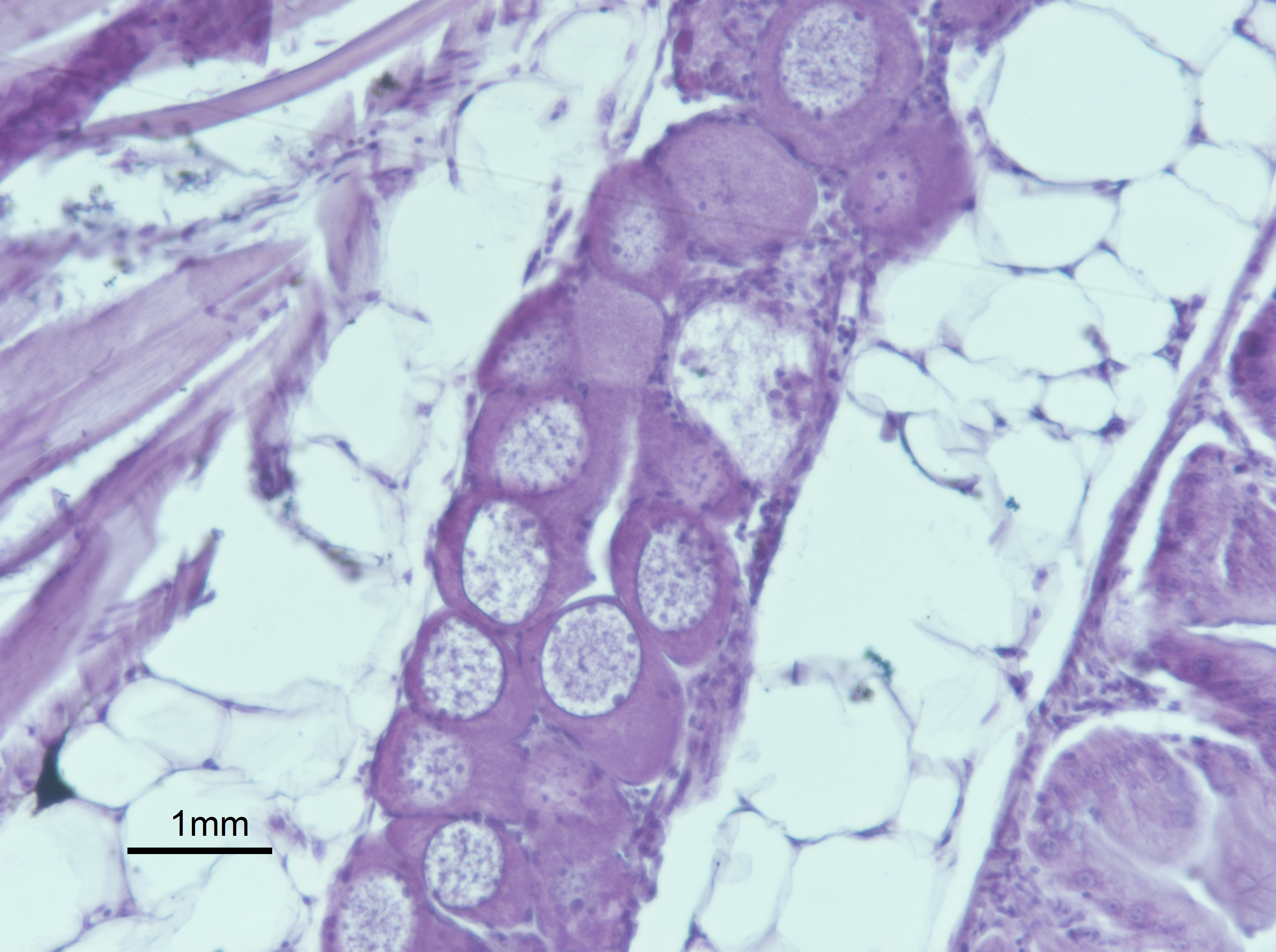

Supplement: Supplementary file 9 — Source data Fig. 5 [file 44319_2026_775_MOESM9_ESM.zip › Figure 5/Figure 5D/+7 line 45 dpf-wnt8 rescue.tif]

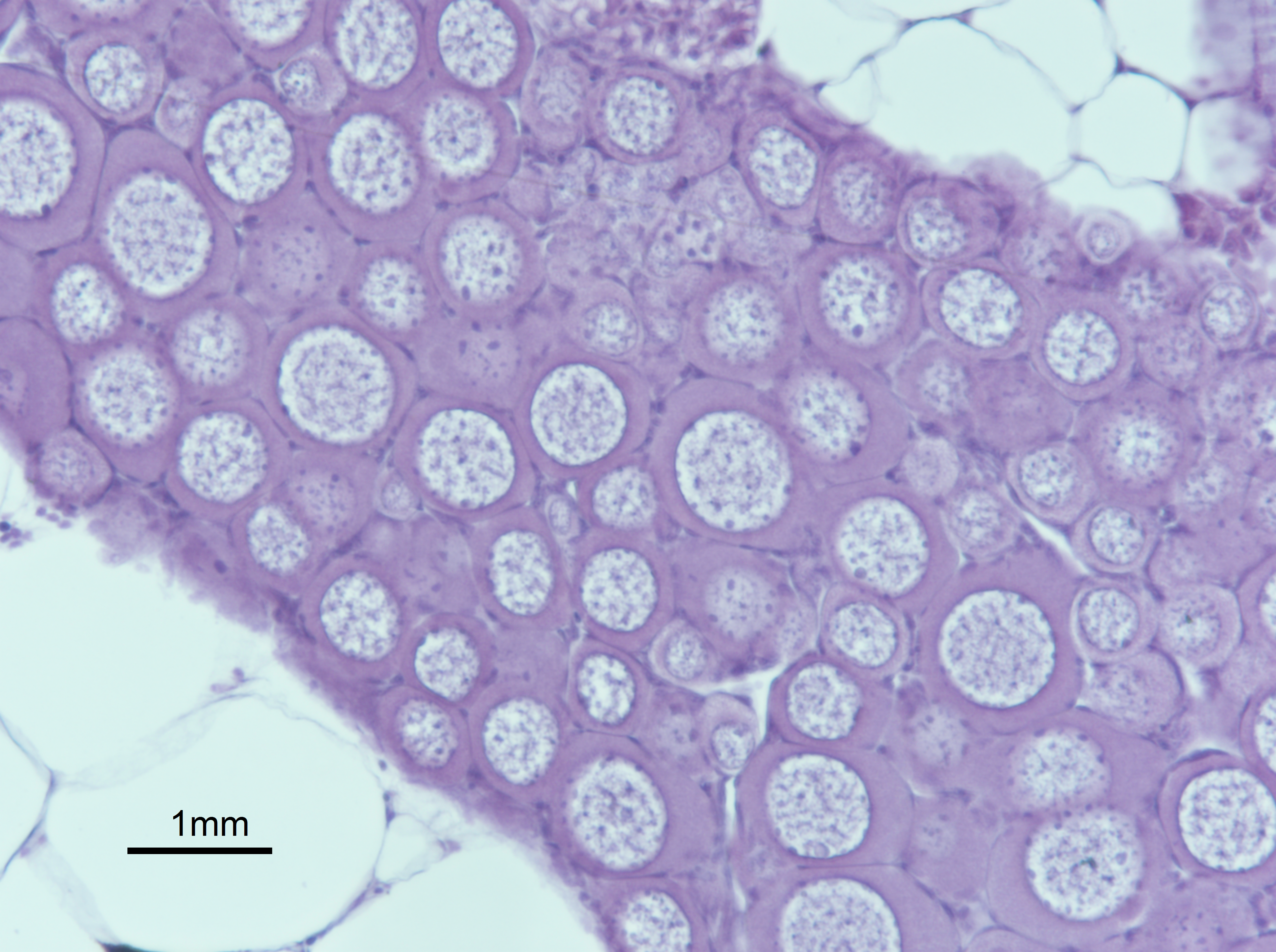

Supplement: Supplementary file 9 — Source data Fig. 5 [file 44319_2026_775_MOESM9_ESM.zip › Figure 5/Figure 5D/+7 line 45 dpf-WT ovary.tif]

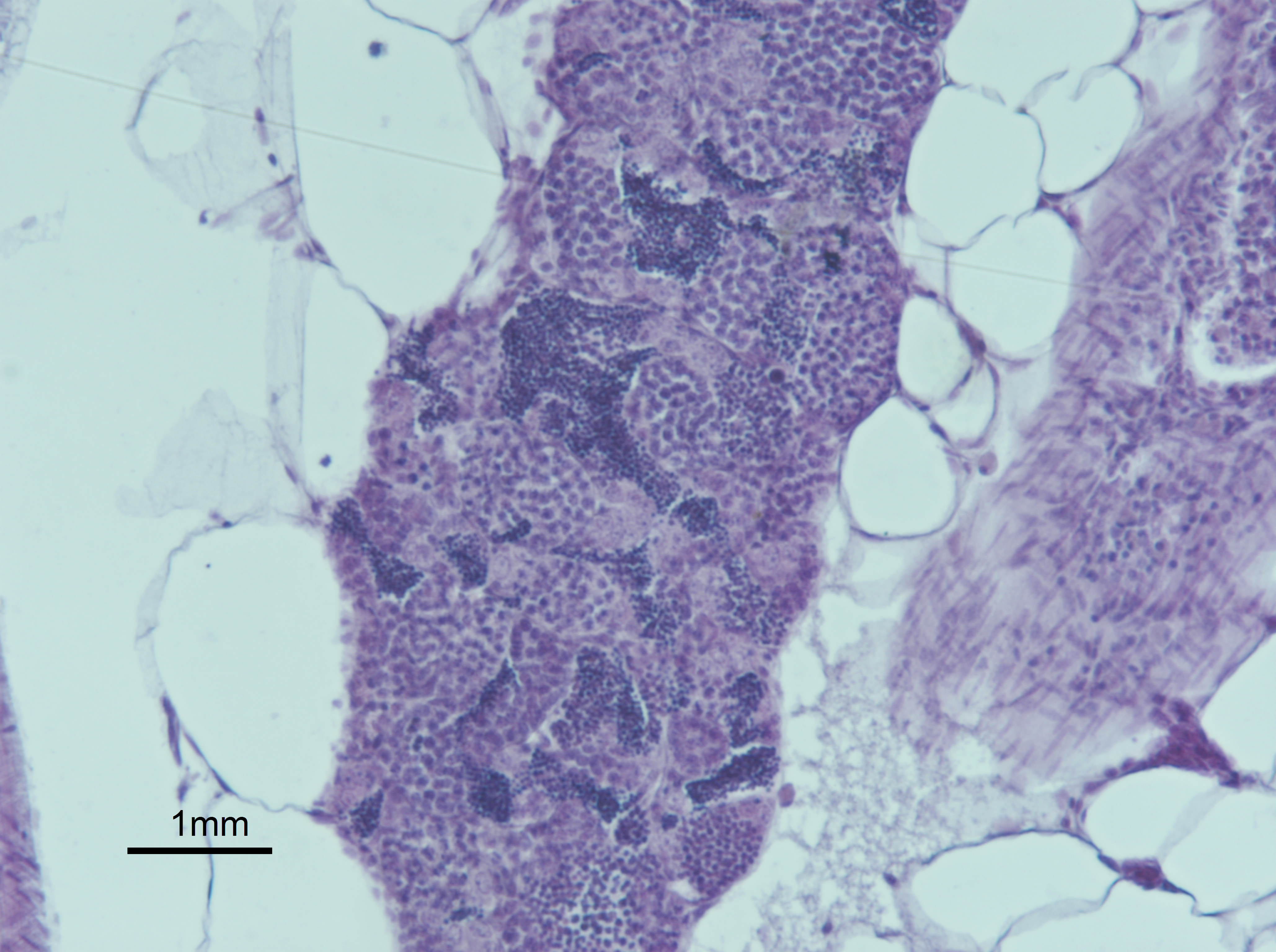

Supplement: Supplementary file 9 — Source data Fig. 5 [file 44319_2026_775_MOESM9_ESM.zip › Figure 5/Figure 5D/+7 line 45 dpf-WT testis.tif]

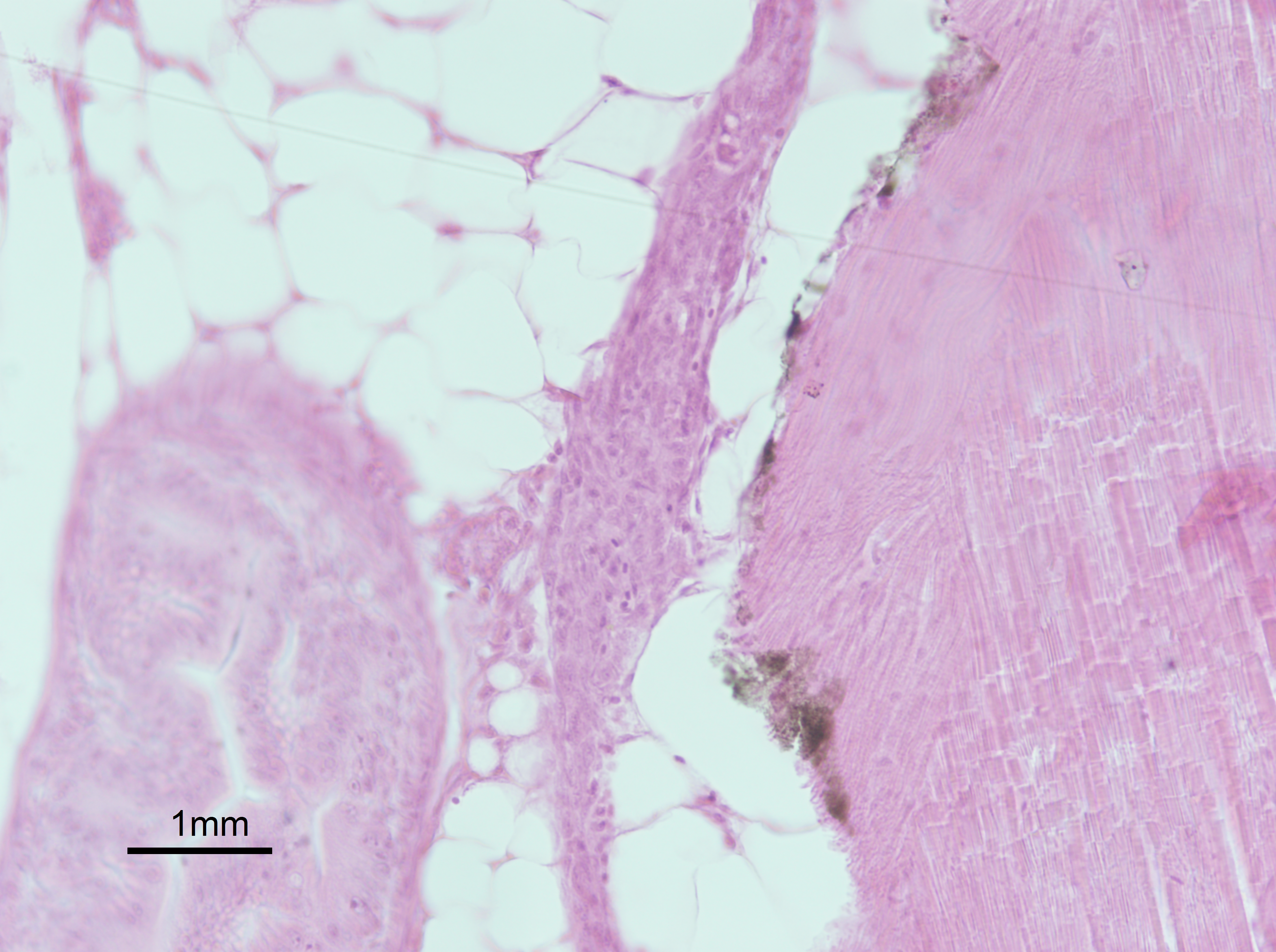

Supplement: Supplementary file 9 — Source data Fig. 5 [file 44319_2026_775_MOESM9_ESM.zip › Figure 5/Figure 5D/Δ7 line 45 dpf-hom.tif]

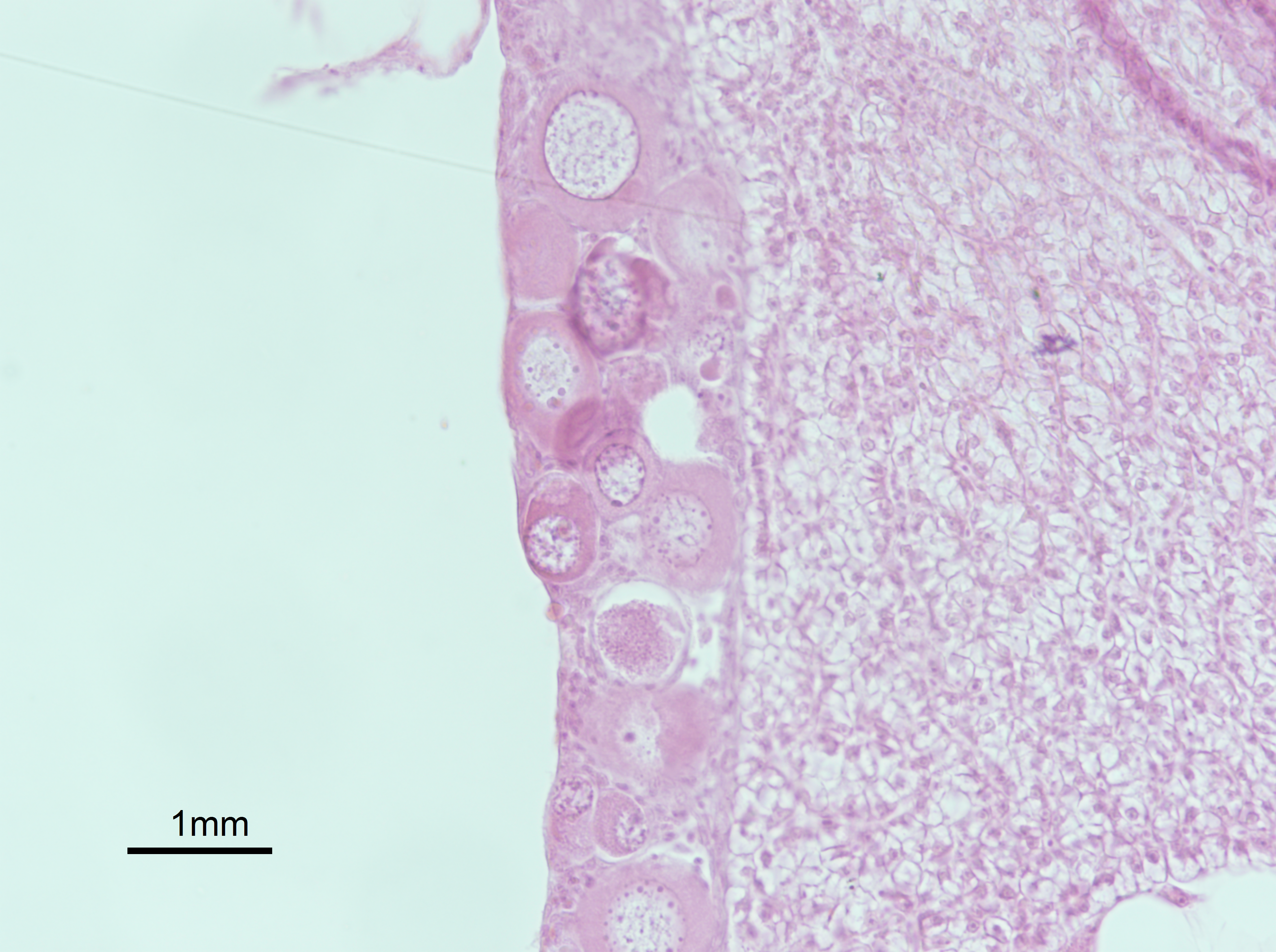

Supplement: Supplementary file 9 — Source data Fig. 5 [file 44319_2026_775_MOESM9_ESM.zip › Figure 5/Figure 5D/Δ7 line 45 dpf-wnt8 rescue.tif]

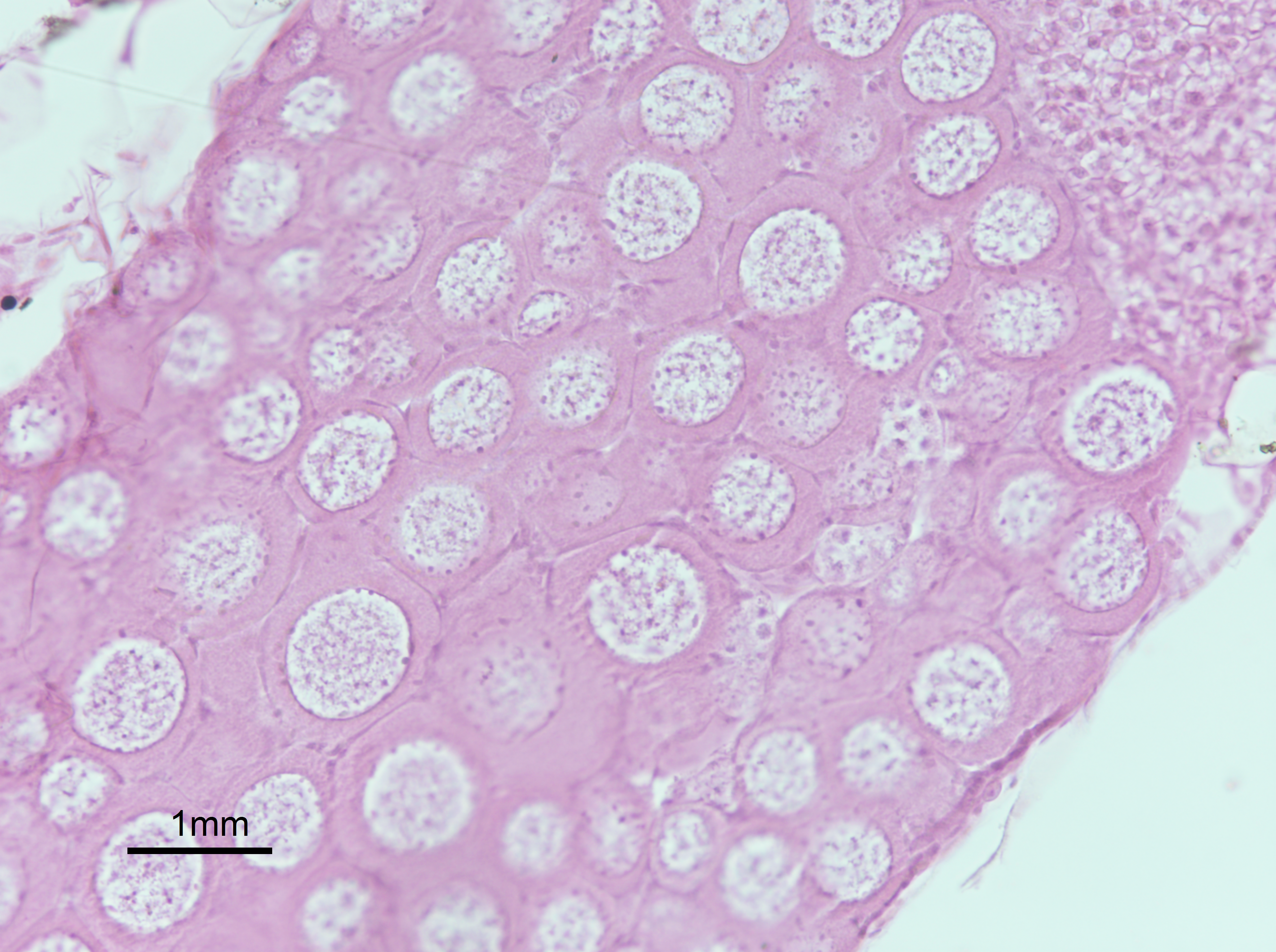

Supplement: Supplementary file 9 — Source data Fig. 5 [file 44319_2026_775_MOESM9_ESM.zip › Figure 5/Figure 5D/Δ7 line 45 dpf-WT ovary.tif]

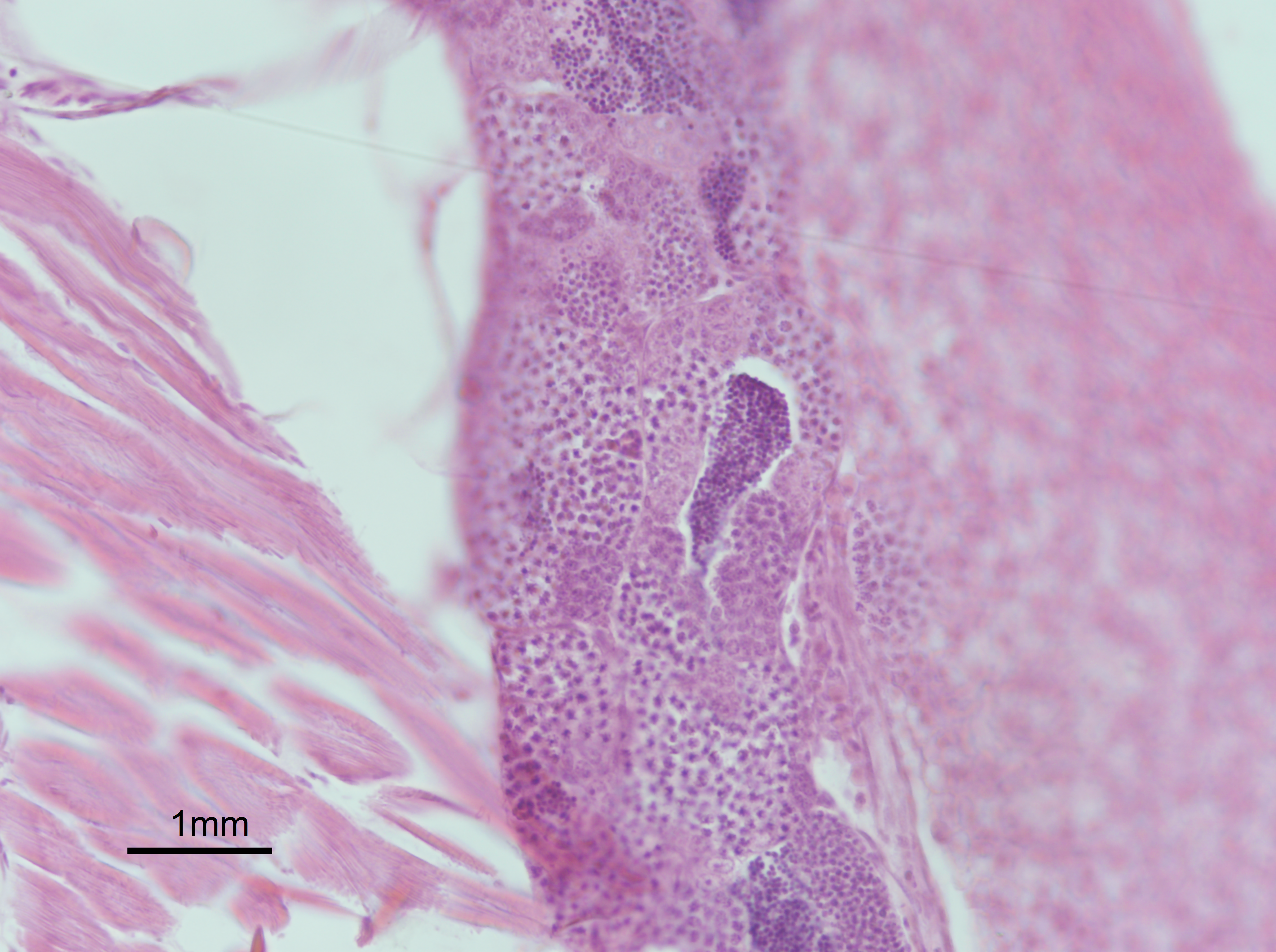

Supplement: Supplementary file 9 — Source data Fig. 5 [file 44319_2026_775_MOESM9_ESM.zip › Figure 5/Figure 5D/Δ7 line 45 dpf-WT testis.tif]

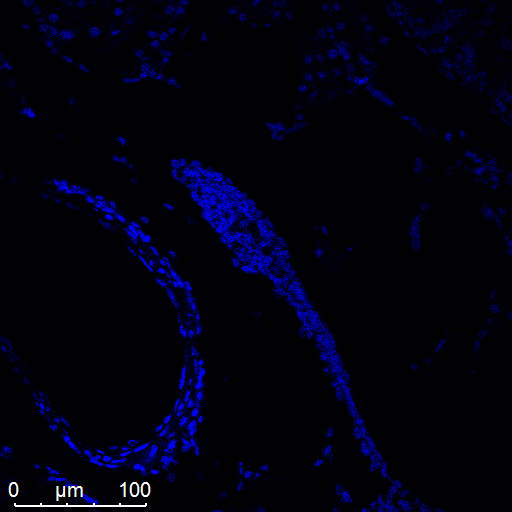

Supplement: Supplementary file 10 — EV Figures Source Data [file 44319_2026_775_MOESM10_ESM.zip › Figure EV3/DAPI hom.tif]

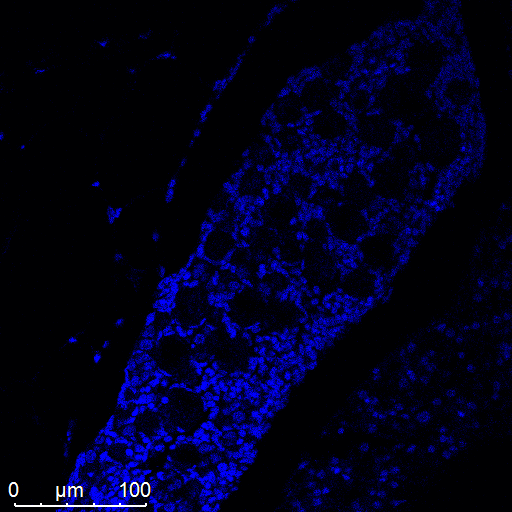

Supplement: Supplementary file 10 — EV Figures Source Data [file 44319_2026_775_MOESM10_ESM.zip › Figure EV3/DAPI WT.tif]

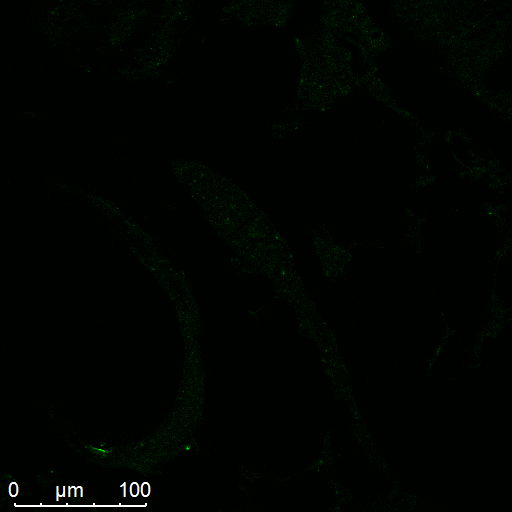

Supplement: Supplementary file 10 — EV Figures Source Data [file 44319_2026_775_MOESM10_ESM.zip › Figure EV3/gfp hom.tif]

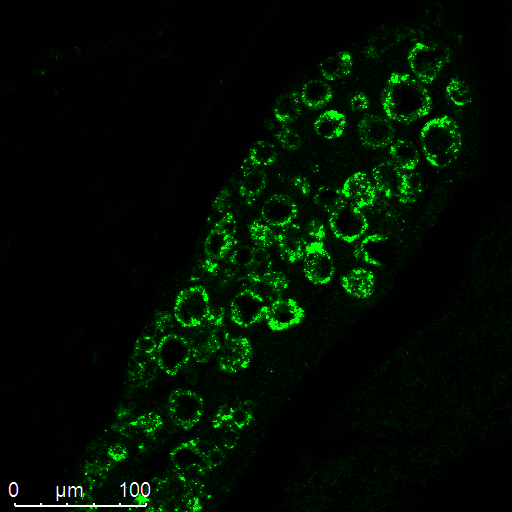

Supplement: Supplementary file 10 — EV Figures Source Data [file 44319_2026_775_MOESM10_ESM.zip › Figure EV3/gfp WT.tif]

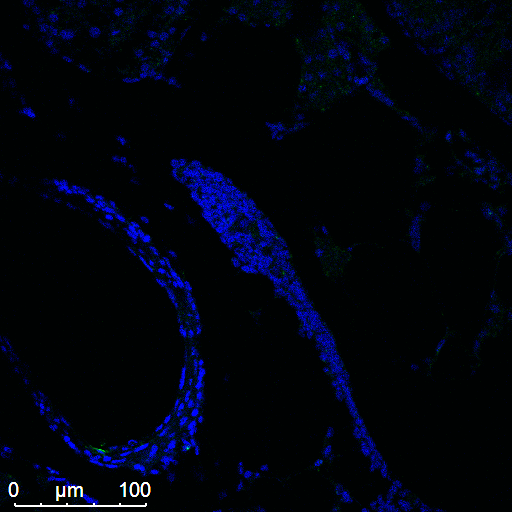

Supplement: Supplementary file 10 — EV Figures Source Data [file 44319_2026_775_MOESM10_ESM.zip › Figure EV3/Merge hom.tif]

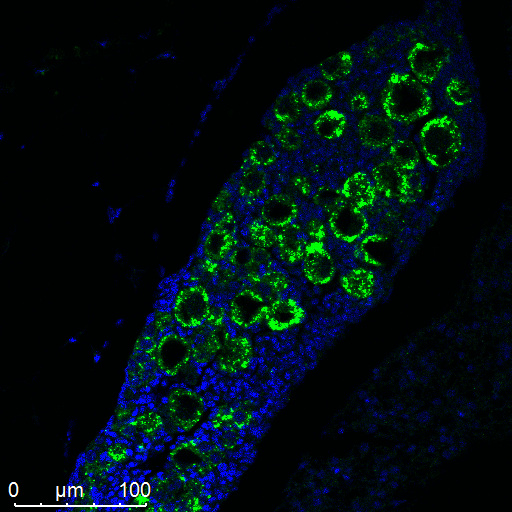

Supplement: Supplementary file 10 — EV Figures Source Data [file 44319_2026_775_MOESM10_ESM.zip › Figure EV3/Merge WT.tif]

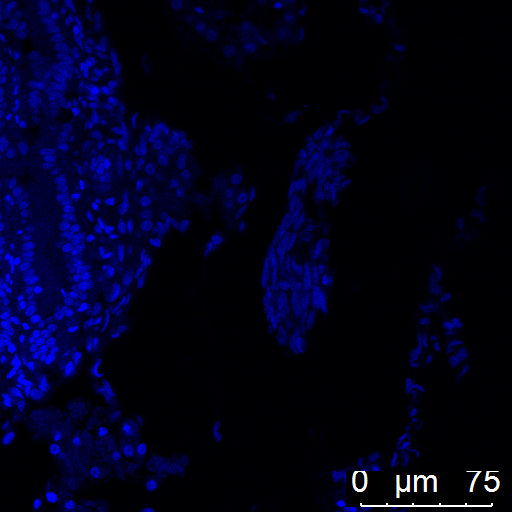

Supplement: Supplementary file 10 — EV Figures Source Data [file 44319_2026_775_MOESM10_ESM.zip › Figure EV4/Figure EV4A/ddx4/DAPI-hom.tif]

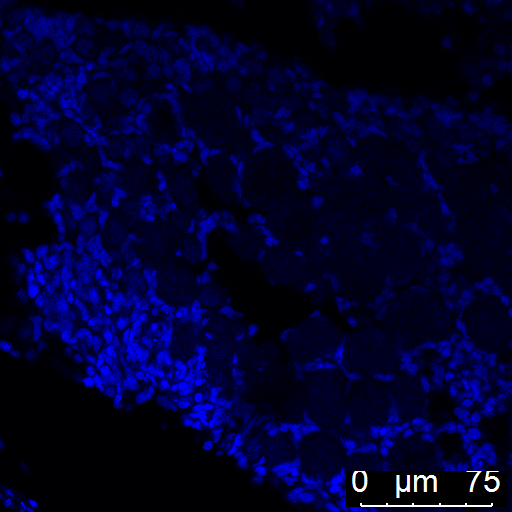

Supplement: Supplementary file 10 — EV Figures Source Data [file 44319_2026_775_MOESM10_ESM.zip › Figure EV4/Figure EV4A/ddx4/DAPI-wnt8 rescue.tif]

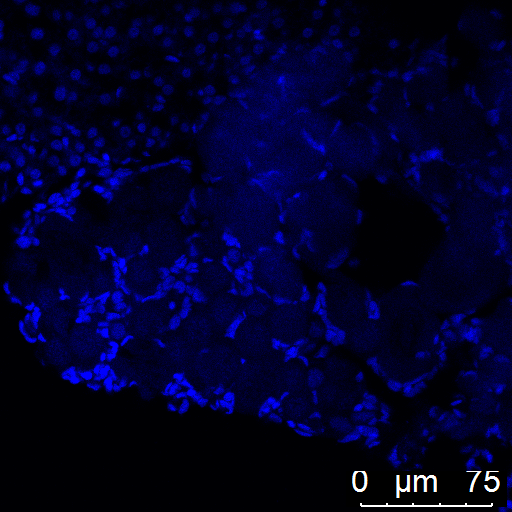

Supplement: Supplementary file 10 — EV Figures Source Data [file 44319_2026_775_MOESM10_ESM.zip › Figure EV4/Figure EV4A/ddx4/DAPI-WT.tif]

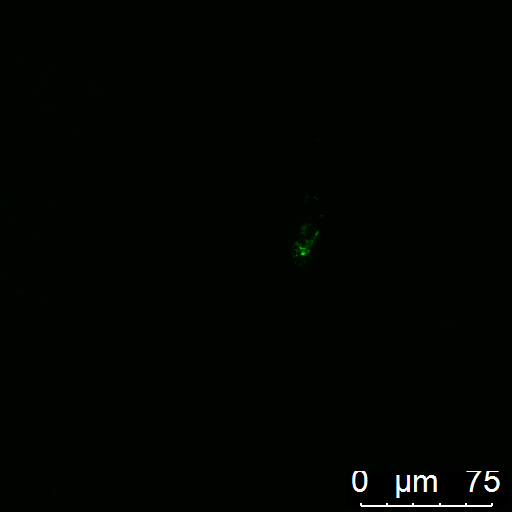

Supplement: Supplementary file 10 — EV Figures Source Data [file 44319_2026_775_MOESM10_ESM.zip › Figure EV4/Figure EV4A/ddx4/ddx4-hom.tif]

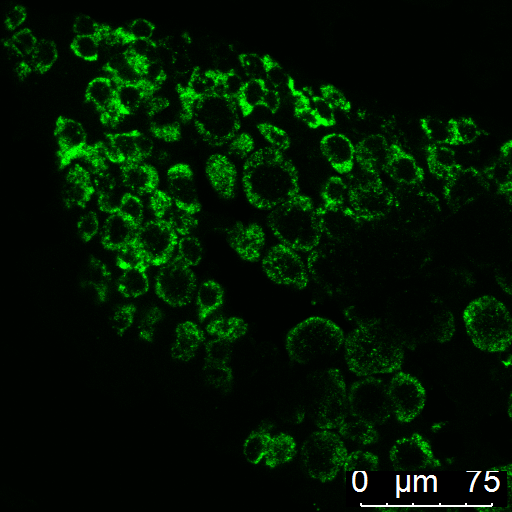

Supplement: Supplementary file 10 — EV Figures Source Data [file 44319_2026_775_MOESM10_ESM.zip › Figure EV4/Figure EV4A/ddx4/ddx4-wnt8 rescue.tif]

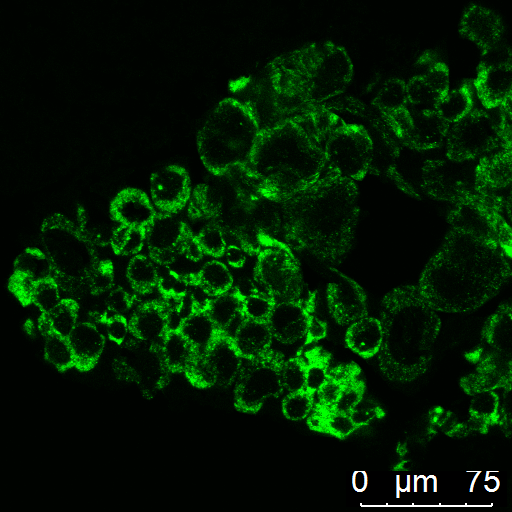

Supplement: Supplementary file 10 — EV Figures Source Data [file 44319_2026_775_MOESM10_ESM.zip › Figure EV4/Figure EV4A/ddx4/ddx4-WT.tif]

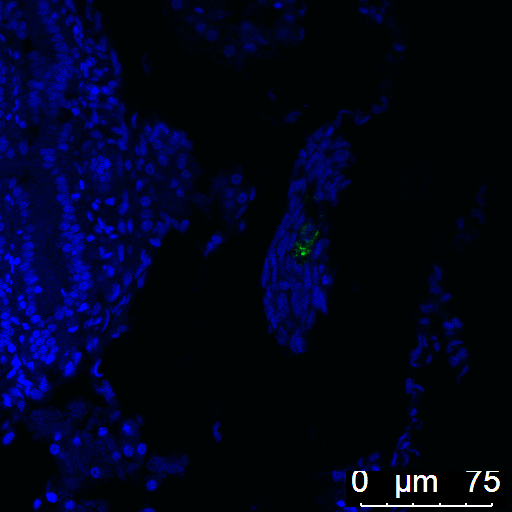

Supplement: Supplementary file 10 — EV Figures Source Data [file 44319_2026_775_MOESM10_ESM.zip › Figure EV4/Figure EV4A/ddx4/Merge-hom.tif]

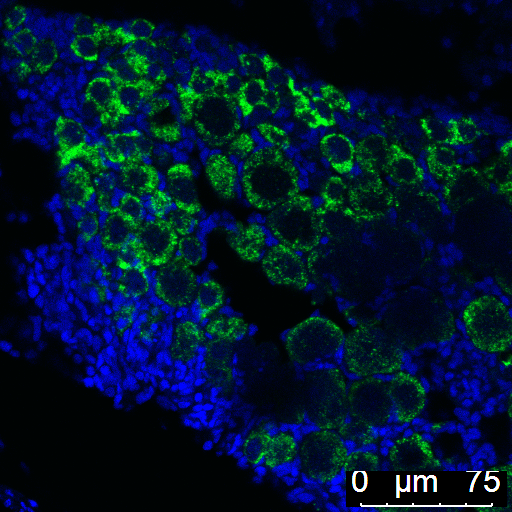

Supplement: Supplementary file 10 — EV Figures Source Data [file 44319_2026_775_MOESM10_ESM.zip › Figure EV4/Figure EV4A/ddx4/Merge-wnt8 rescue.tif]

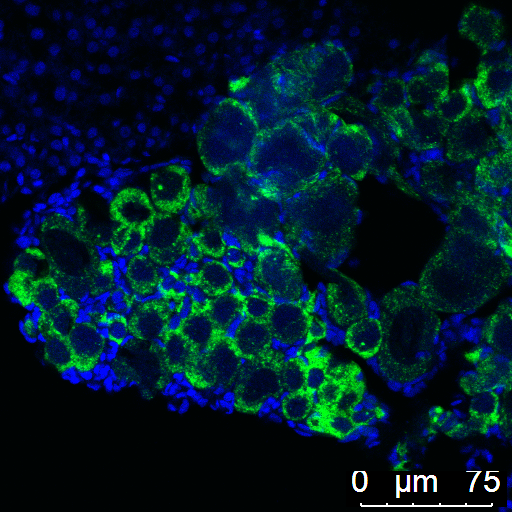

Supplement: Supplementary file 10 — EV Figures Source Data [file 44319_2026_775_MOESM10_ESM.zip › Figure EV4/Figure EV4A/ddx4/Merge-WT.tif]

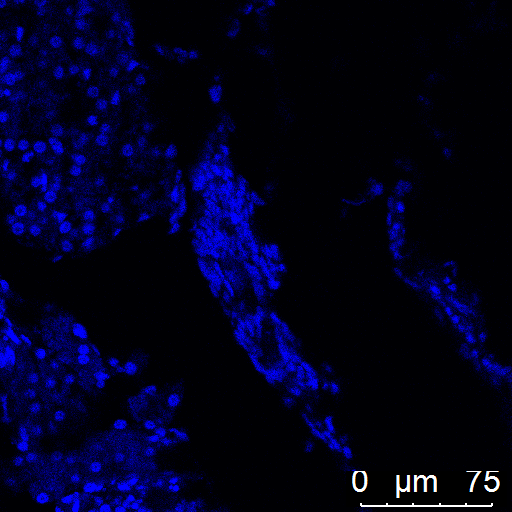

Supplement: Supplementary file 10 — EV Figures Source Data [file 44319_2026_775_MOESM10_ESM.zip › Figure EV4/Figure EV4A/dnd1/DAPI-hom.tif]

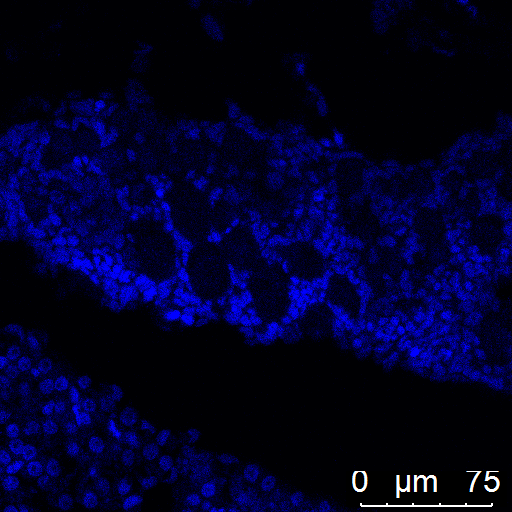

Supplement: Supplementary file 10 — EV Figures Source Data [file 44319_2026_775_MOESM10_ESM.zip › Figure EV4/Figure EV4A/dnd1/DAPI-wnt8 rescue.tif]

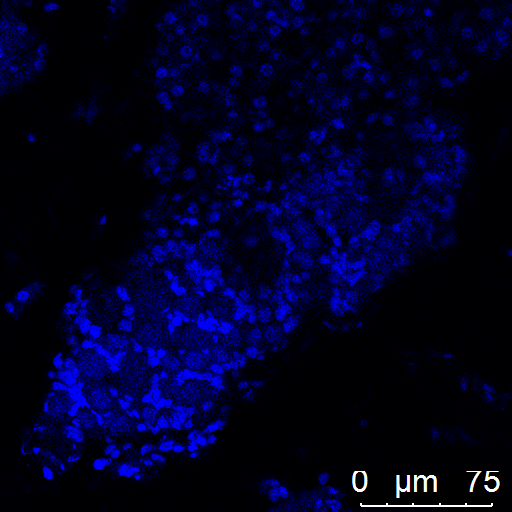

Supplement: Supplementary file 10 — EV Figures Source Data [file 44319_2026_775_MOESM10_ESM.zip › Figure EV4/Figure EV4A/dnd1/DAPI-WT.tif]

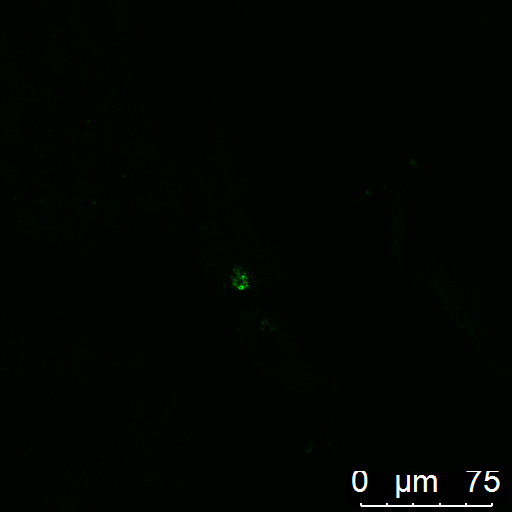

Supplement: Supplementary file 10 — EV Figures Source Data [file 44319_2026_775_MOESM10_ESM.zip › Figure EV4/Figure EV4A/dnd1/dnd1-hom.tif]

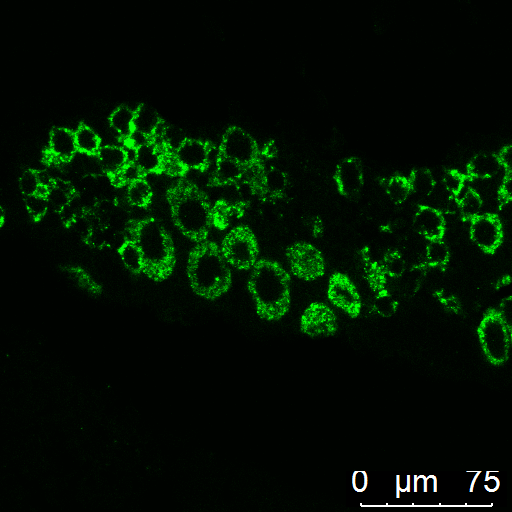

Supplement: Supplementary file 10 — EV Figures Source Data [file 44319_2026_775_MOESM10_ESM.zip › Figure EV4/Figure EV4A/dnd1/dnd1-wnt8 rescue.tif]

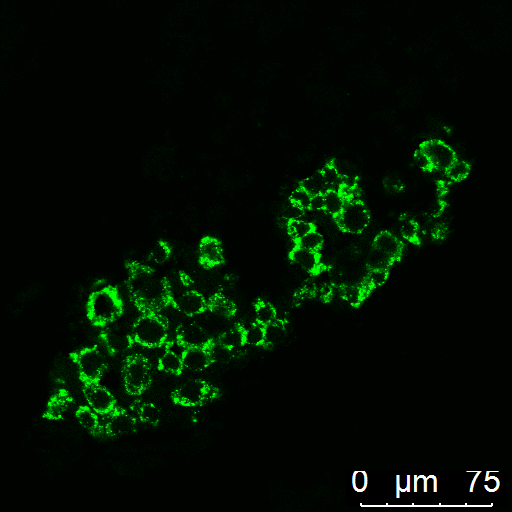

Supplement: Supplementary file 10 — EV Figures Source Data [file 44319_2026_775_MOESM10_ESM.zip › Figure EV4/Figure EV4A/dnd1/dnd1-WT.tif]

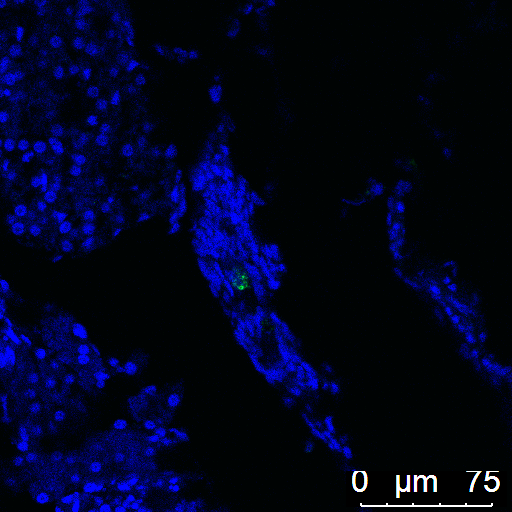

Supplement: Supplementary file 10 — EV Figures Source Data [file 44319_2026_775_MOESM10_ESM.zip › Figure EV4/Figure EV4A/dnd1/Merge-hom.tif]

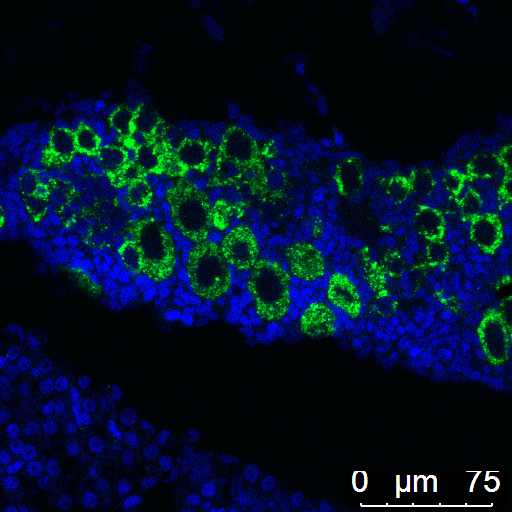

Supplement: Supplementary file 10 — EV Figures Source Data [file 44319_2026_775_MOESM10_ESM.zip › Figure EV4/Figure EV4A/dnd1/Merge-wnt8 rescue.tif]

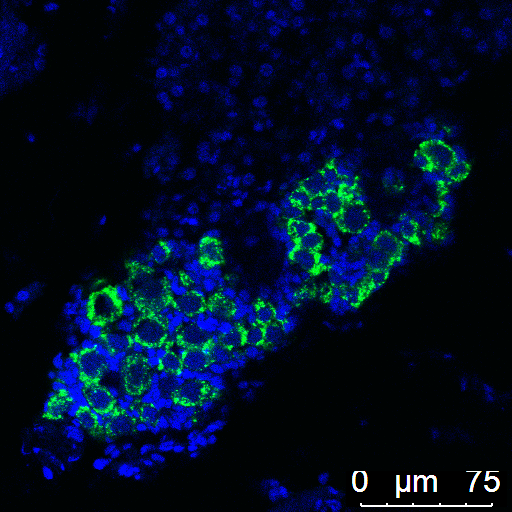

Supplement: Supplementary file 10 — EV Figures Source Data [file 44319_2026_775_MOESM10_ESM.zip › Figure EV4/Figure EV4A/dnd1/Merge-WT.tif]

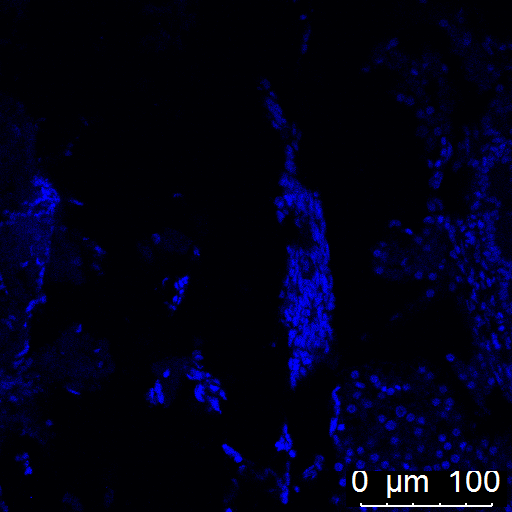

Supplement: Supplementary file 10 — EV Figures Source Data [file 44319_2026_775_MOESM10_ESM.zip › Figure EV4/Figure EV4A/piwil1/DAPI-hom.tif]

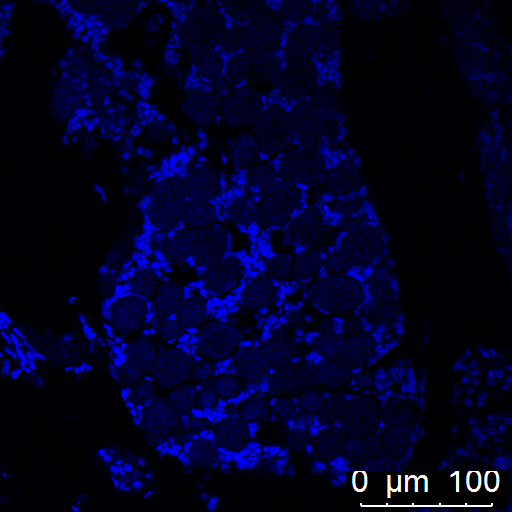

Supplement: Supplementary file 10 — EV Figures Source Data [file 44319_2026_775_MOESM10_ESM.zip › Figure EV4/Figure EV4A/piwil1/DAPI-wnt8 rescue.tif]

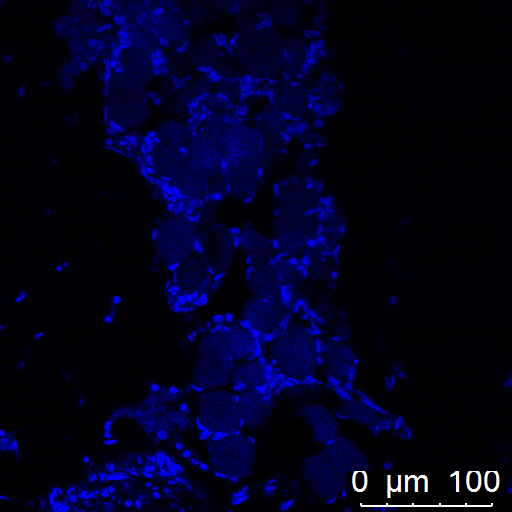

Supplement: Supplementary file 10 — EV Figures Source Data [file 44319_2026_775_MOESM10_ESM.zip › Figure EV4/Figure EV4A/piwil1/DAPI-WT.tif]

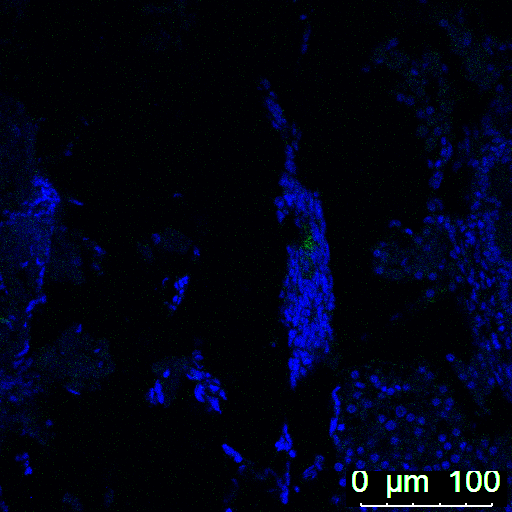

Supplement: Supplementary file 10 — EV Figures Source Data [file 44319_2026_775_MOESM10_ESM.zip › Figure EV4/Figure EV4A/piwil1/Merge-hom.tif]

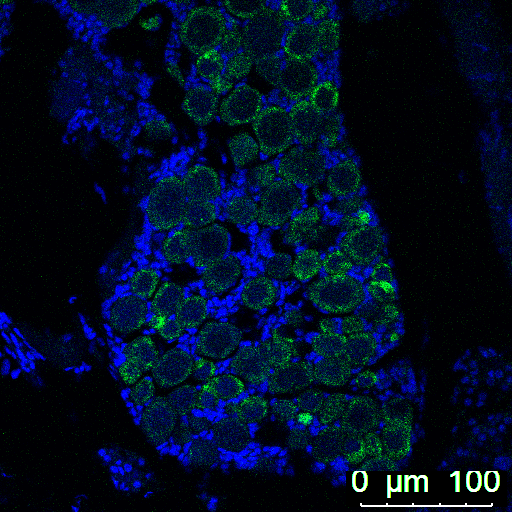

Supplement: Supplementary file 10 — EV Figures Source Data [file 44319_2026_775_MOESM10_ESM.zip › Figure EV4/Figure EV4A/piwil1/Merge-wnt8 rescue.tif]

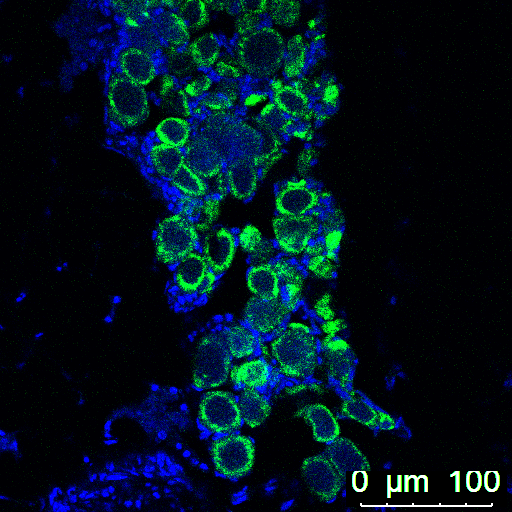

Supplement: Supplementary file 10 — EV Figures Source Data [file 44319_2026_775_MOESM10_ESM.zip › Figure EV4/Figure EV4A/piwil1/Merge-WT.tif]

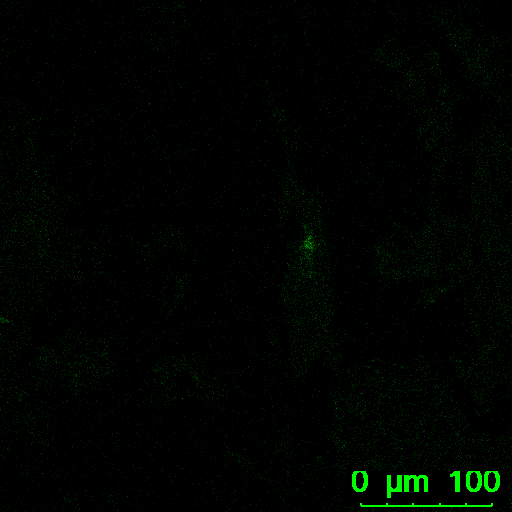

Supplement: Supplementary file 10 — EV Figures Source Data [file 44319_2026_775_MOESM10_ESM.zip › Figure EV4/Figure EV4A/piwil1/piwil1-hom.tif]

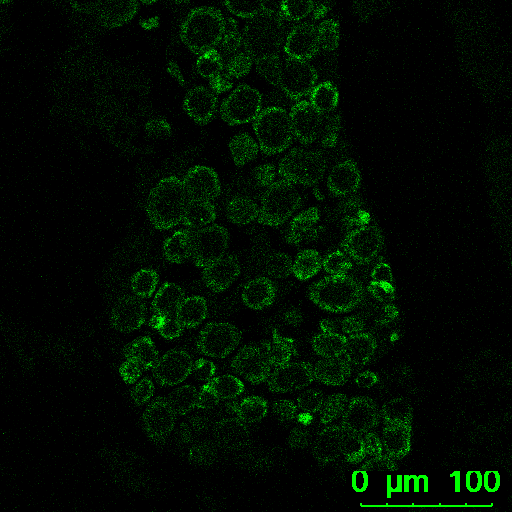

Supplement: Supplementary file 10 — EV Figures Source Data [file 44319_2026_775_MOESM10_ESM.zip › Figure EV4/Figure EV4A/piwil1/piwil1-wnt8 rescue.tif]

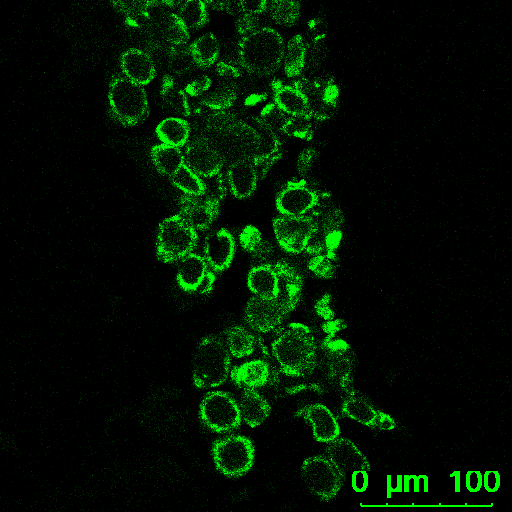

Supplement: Supplementary file 10 — EV Figures Source Data [file 44319_2026_775_MOESM10_ESM.zip › Figure EV4/Figure EV4A/piwil1/piwil1-WT.tif]

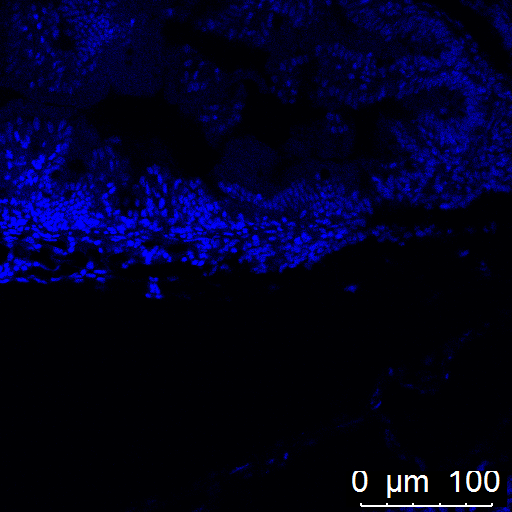

Supplement: Supplementary file 10 — EV Figures Source Data [file 44319_2026_775_MOESM10_ESM.zip › Figure EV4/Figure EV4A/piwil2/DAPI-hom.tif]

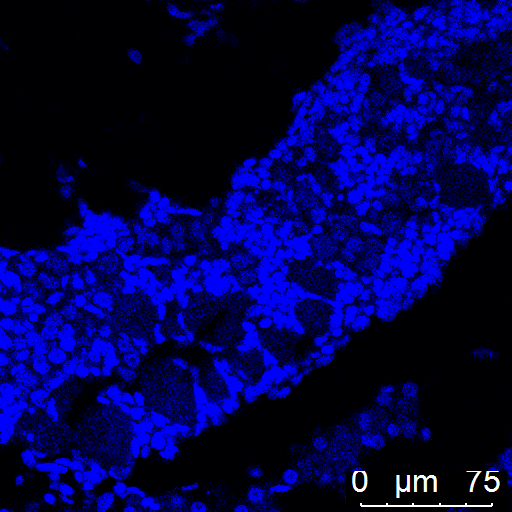

Supplement: Supplementary file 10 — EV Figures Source Data [file 44319_2026_775_MOESM10_ESM.zip › Figure EV4/Figure EV4A/piwil2/DAPI-wnt8 rescue.tif]

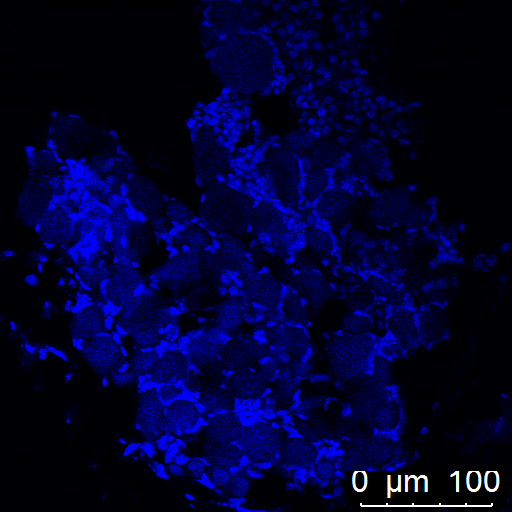

Supplement: Supplementary file 10 — EV Figures Source Data [file 44319_2026_775_MOESM10_ESM.zip › Figure EV4/Figure EV4A/piwil2/DAPI-WT.tif]

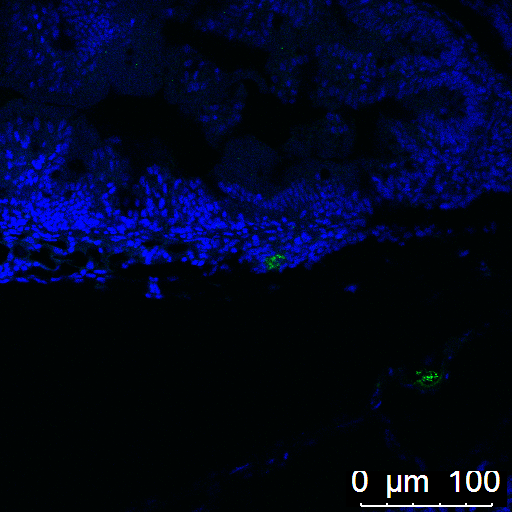

Supplement: Supplementary file 10 — EV Figures Source Data [file 44319_2026_775_MOESM10_ESM.zip › Figure EV4/Figure EV4A/piwil2/Merge-hom.tif]

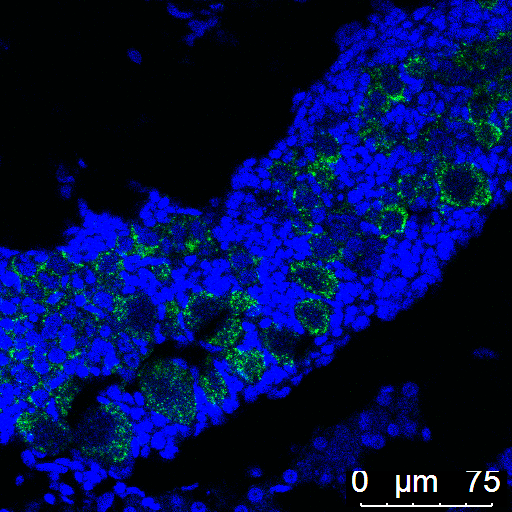

Supplement: Supplementary file 10 — EV Figures Source Data [file 44319_2026_775_MOESM10_ESM.zip › Figure EV4/Figure EV4A/piwil2/Merge-wnt8 rescue.tif]

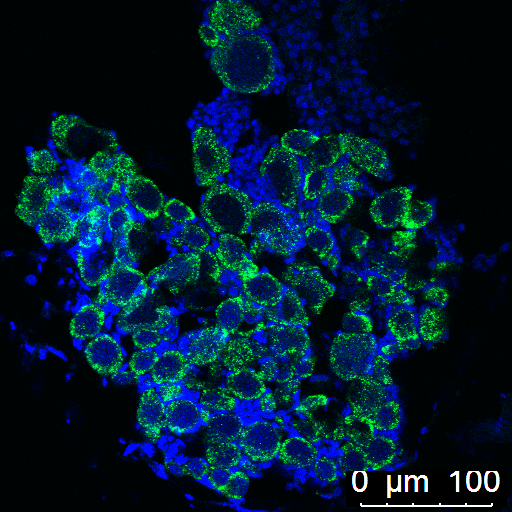

Supplement: Supplementary file 10 — EV Figures Source Data [file 44319_2026_775_MOESM10_ESM.zip › Figure EV4/Figure EV4A/piwil2/Merge-WT.tif]

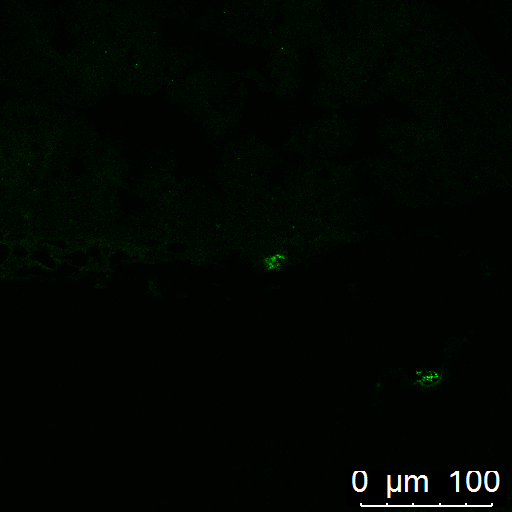

Supplement: Supplementary file 10 — EV Figures Source Data [file 44319_2026_775_MOESM10_ESM.zip › Figure EV4/Figure EV4A/piwil2/piwil2-homo.tif]

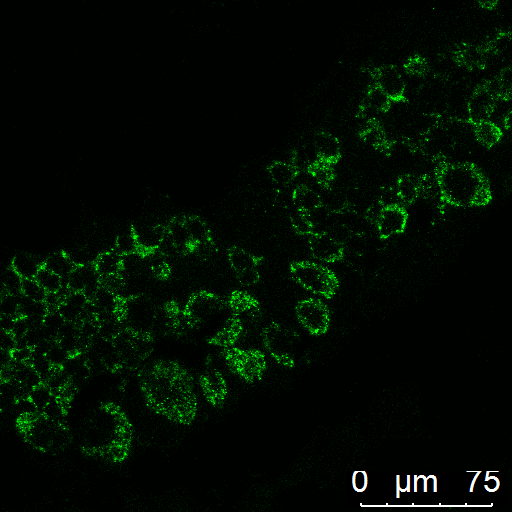

Supplement: Supplementary file 10 — EV Figures Source Data [file 44319_2026_775_MOESM10_ESM.zip › Figure EV4/Figure EV4A/piwil2/piwil2-wnt8 rescue.tif]

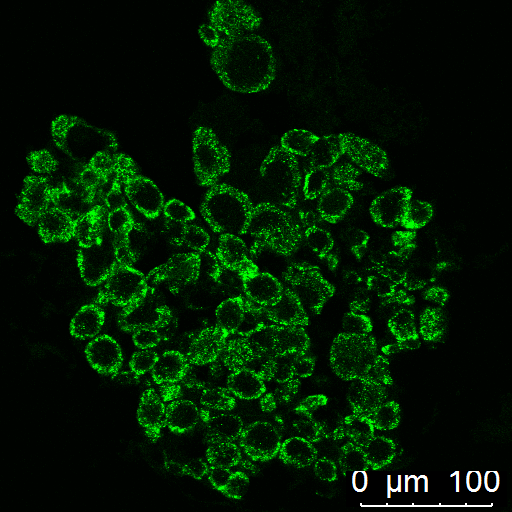

Supplement: Supplementary file 10 — EV Figures Source Data [file 44319_2026_775_MOESM10_ESM.zip › Figure EV4/Figure EV4A/piwil2/piwil2-WT.tif]

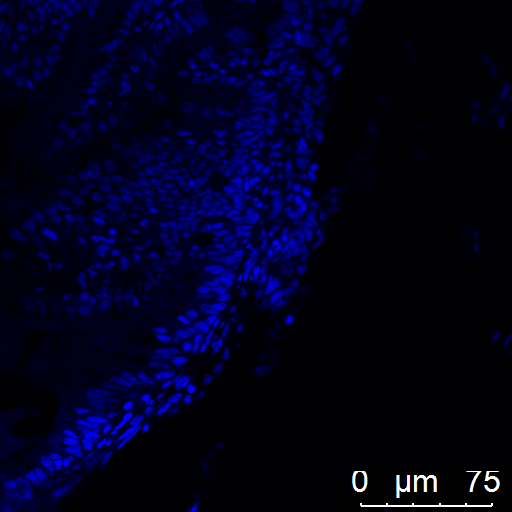

Supplement: Supplementary file 10 — EV Figures Source Data [file 44319_2026_775_MOESM10_ESM.zip › Figure EV4/Figure EV4A/tdrd1/DAPI-hom.tif]

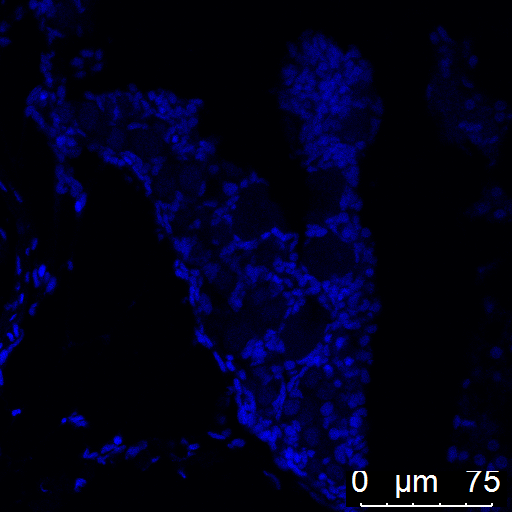

Supplement: Supplementary file 10 — EV Figures Source Data [file 44319_2026_775_MOESM10_ESM.zip › Figure EV4/Figure EV4A/tdrd1/DAPI-wnt8 rescue.tif]

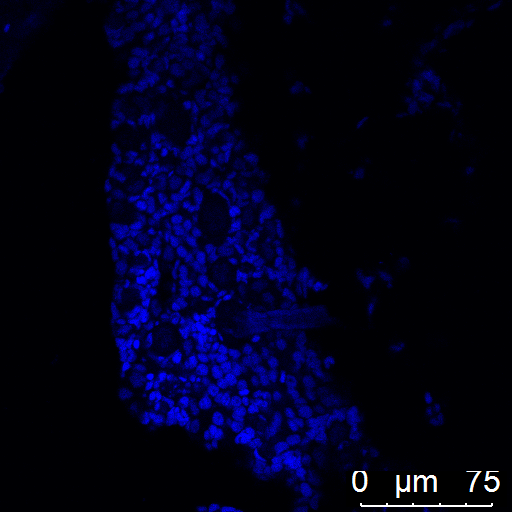

Supplement: Supplementary file 10 — EV Figures Source Data [file 44319_2026_775_MOESM10_ESM.zip › Figure EV4/Figure EV4A/tdrd1/DAPI-WT.tif]

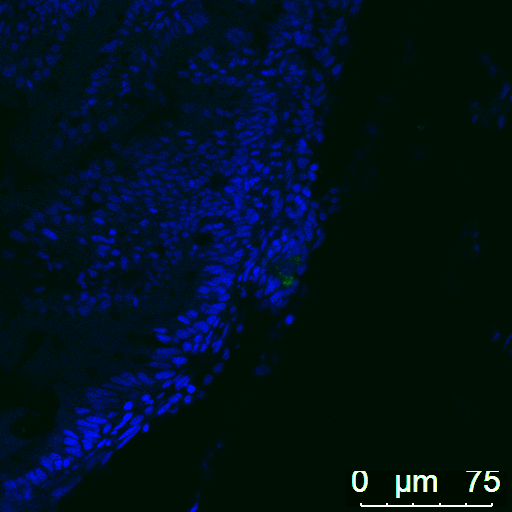

Supplement: Supplementary file 10 — EV Figures Source Data [file 44319_2026_775_MOESM10_ESM.zip › Figure EV4/Figure EV4A/tdrd1/Merge-hom.tif]

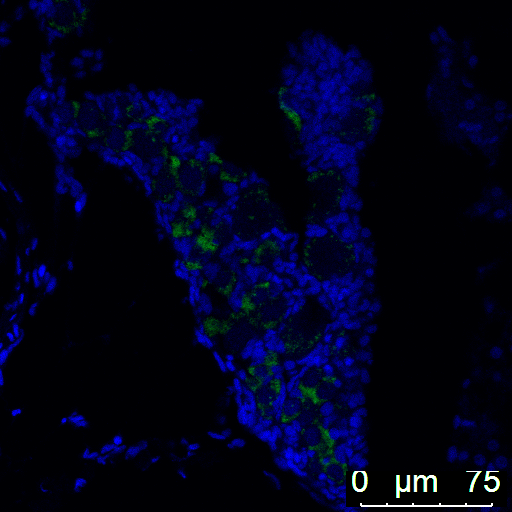

Supplement: Supplementary file 10 — EV Figures Source Data [file 44319_2026_775_MOESM10_ESM.zip › Figure EV4/Figure EV4A/tdrd1/Merge-wnt8 rescue.tif]

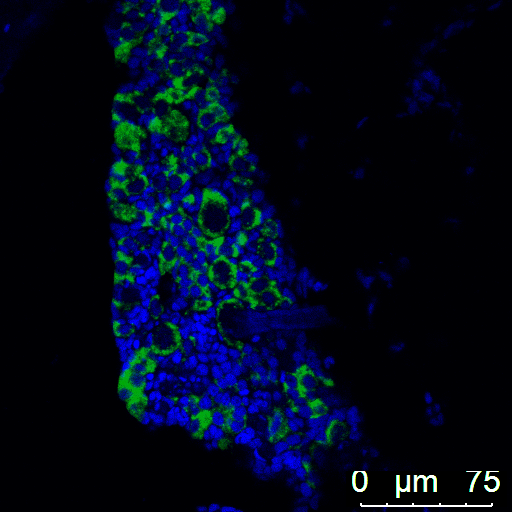

Supplement: Supplementary file 10 — EV Figures Source Data [file 44319_2026_775_MOESM10_ESM.zip › Figure EV4/Figure EV4A/tdrd1/Merge-WT.tif]

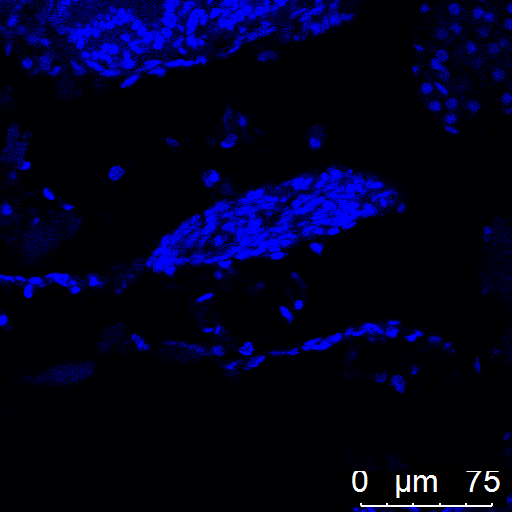

Supplement: Supplementary file 10 — EV Figures Source Data [file 44319_2026_775_MOESM10_ESM.zip › Figure EV4/Figure EV4A/tdrd7a/DAPI-hom.tif]

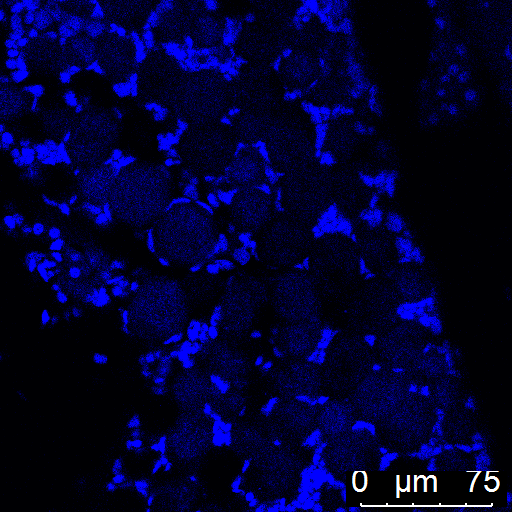

Supplement: Supplementary file 10 — EV Figures Source Data [file 44319_2026_775_MOESM10_ESM.zip › Figure EV4/Figure EV4A/tdrd7a/DAPI-wnt8 rescue.tif]

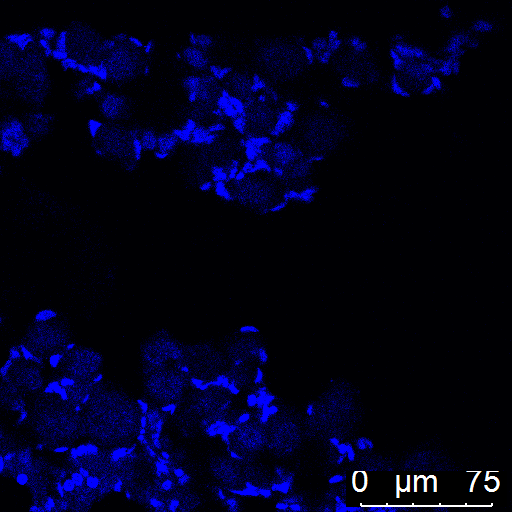

Supplement: Supplementary file 10 — EV Figures Source Data [file 44319_2026_775_MOESM10_ESM.zip › Figure EV4/Figure EV4A/tdrd7a/DAPI-WT.tif]

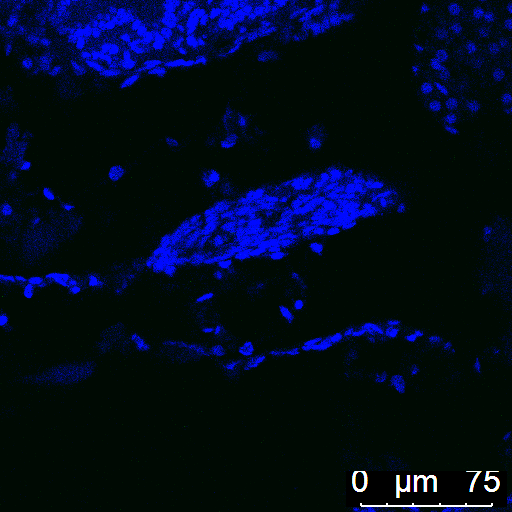

Supplement: Supplementary file 10 — EV Figures Source Data [file 44319_2026_775_MOESM10_ESM.zip › Figure EV4/Figure EV4A/tdrd7a/Merge-hom.tif]

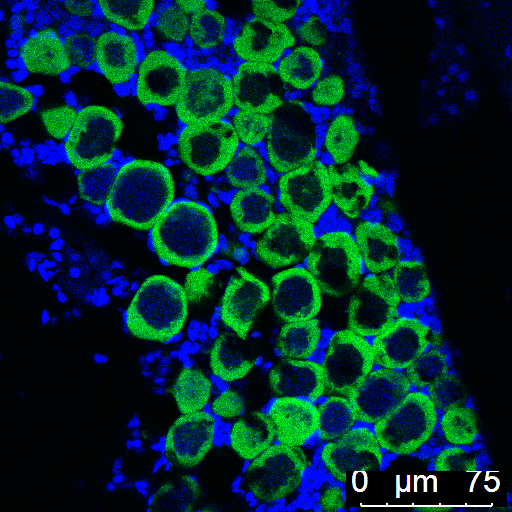

Supplement: Supplementary file 10 — EV Figures Source Data [file 44319_2026_775_MOESM10_ESM.zip › Figure EV4/Figure EV4A/tdrd7a/Merge-wnt8 rescue.tif]

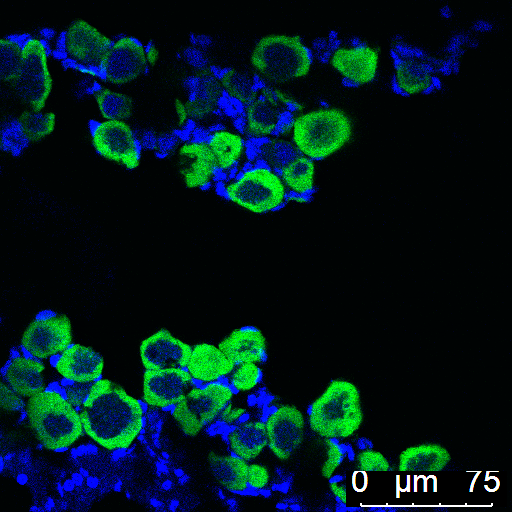

Supplement: Supplementary file 10 — EV Figures Source Data [file 44319_2026_775_MOESM10_ESM.zip › Figure EV4/Figure EV4A/tdrd7a/Merge-WT.tif]

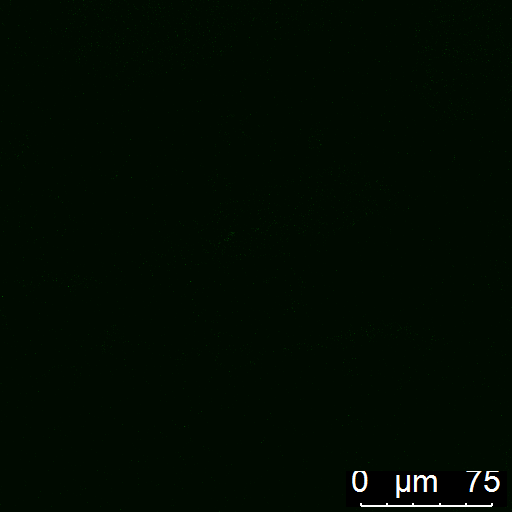

Supplement: Supplementary file 10 — EV Figures Source Data [file 44319_2026_775_MOESM10_ESM.zip › Figure EV4/Figure EV4A/tdrd7a/tdrd7a-hom.tif]

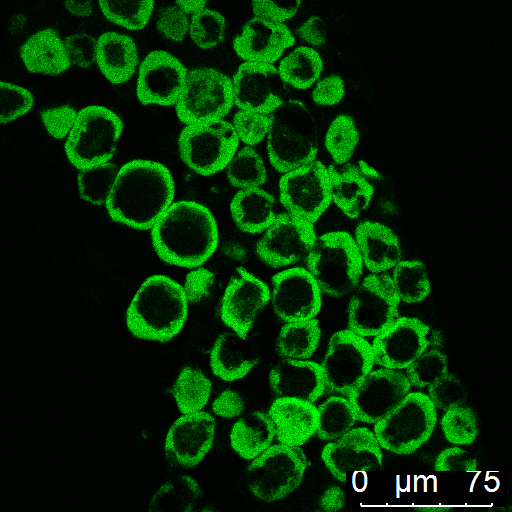

Supplement: Supplementary file 10 — EV Figures Source Data [file 44319_2026_775_MOESM10_ESM.zip › Figure EV4/Figure EV4A/tdrd7a/tdrd7a-wnt8 rescue.tif]
