# Supplementary figures and images for: Stn1 promotes zebrafish oocyte development via amplifying Wnt/β-catenin signaling (part 3 of 5)
Source: EMBO Rep. 2026 Apr 17;27(12):3252–76. doi: 10.1038/s44319-026-00775-8 (PMC13304171; doi:10.1038/s44319-026-00775-8)

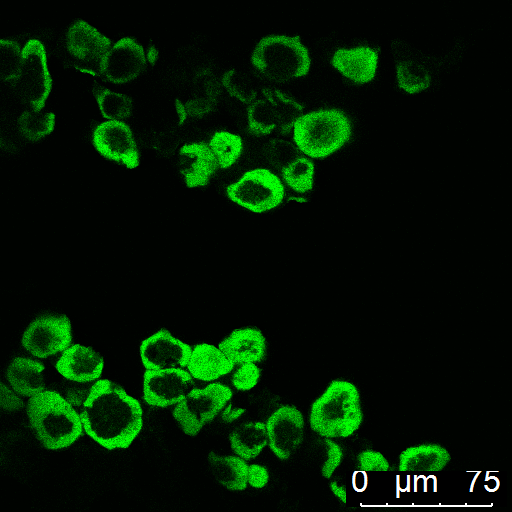

Supplement: Supplementary file 10 — EV Figures Source Data [file 44319_2026_775_MOESM10_ESM.zip › Figure EV4/Figure EV4A/tdrd7a/tdrd7a-WT.tif]

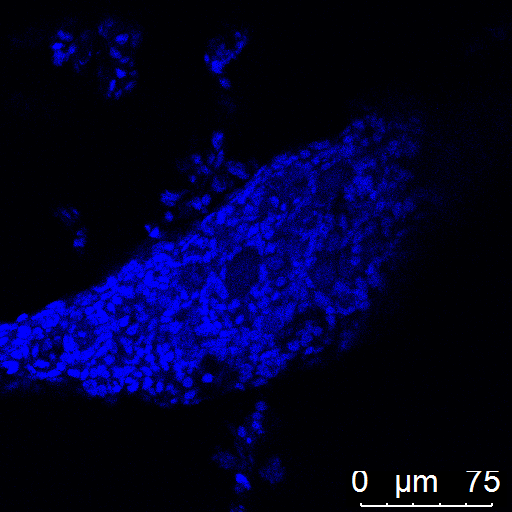

Supplement: Supplementary file 10 — EV Figures Source Data [file 44319_2026_775_MOESM10_ESM.zip › Figure EV4/Figure EV4A/tdrd9/DAPI-hom.tif]

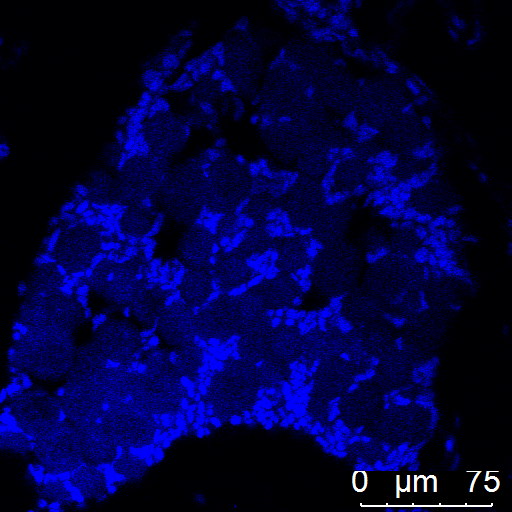

Supplement: Supplementary file 10 — EV Figures Source Data [file 44319_2026_775_MOESM10_ESM.zip › Figure EV4/Figure EV4A/tdrd9/DAPI-wnt8 rescue.tif]

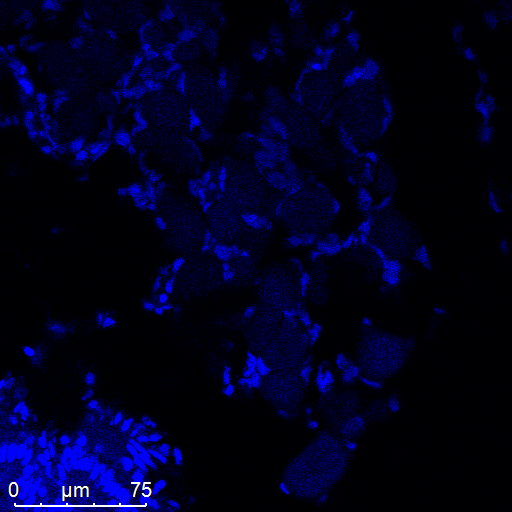

Supplement: Supplementary file 10 — EV Figures Source Data [file 44319_2026_775_MOESM10_ESM.zip › Figure EV4/Figure EV4A/tdrd9/DAPI-WT.tif]

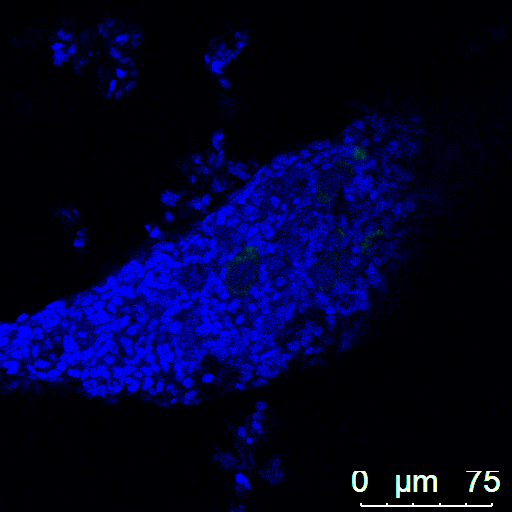

Supplement: Supplementary file 10 — EV Figures Source Data [file 44319_2026_775_MOESM10_ESM.zip › Figure EV4/Figure EV4A/tdrd9/Merge-hom.tif]

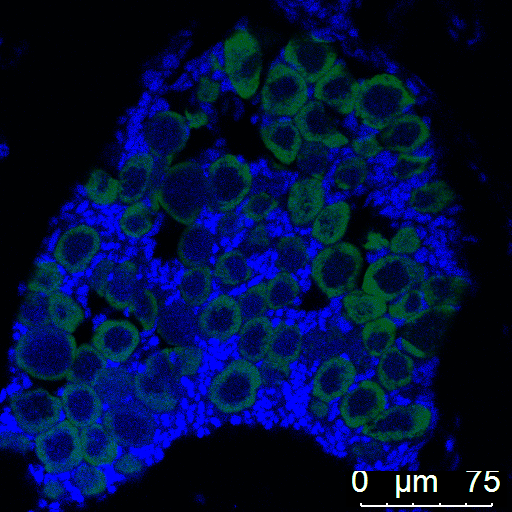

Supplement: Supplementary file 10 — EV Figures Source Data [file 44319_2026_775_MOESM10_ESM.zip › Figure EV4/Figure EV4A/tdrd9/Merge-wnt8 rescue.tif]

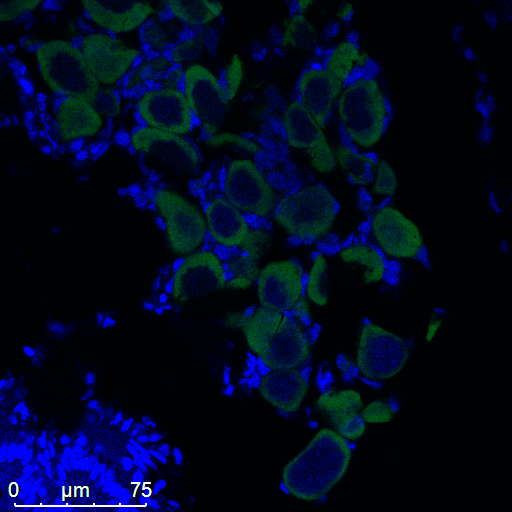

Supplement: Supplementary file 10 — EV Figures Source Data [file 44319_2026_775_MOESM10_ESM.zip › Figure EV4/Figure EV4A/tdrd9/Merge-WT.tif]

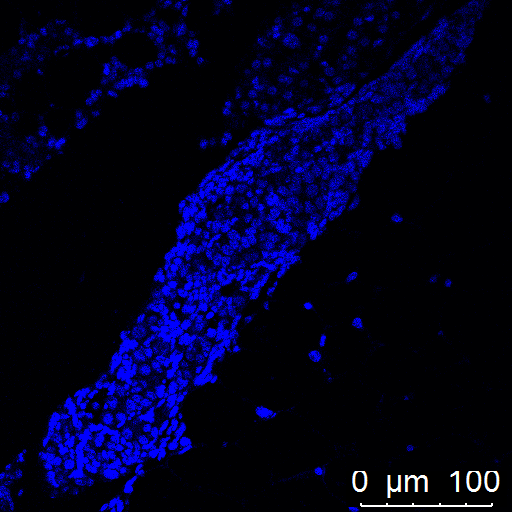

Supplement: Supplementary file 10 — EV Figures Source Data [file 44319_2026_775_MOESM10_ESM.zip › Figure EV1/Figure EV1A/19 dpf DAPI.tif]

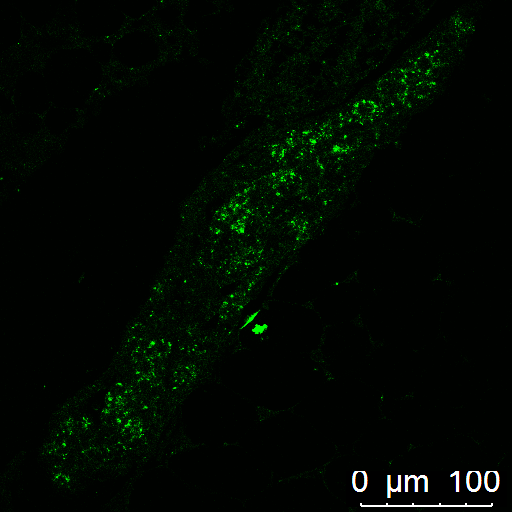

Supplement: Supplementary file 10 — EV Figures Source Data [file 44319_2026_775_MOESM10_ESM.zip › Figure EV1/Figure EV1A/19 dpf gfp.tif]

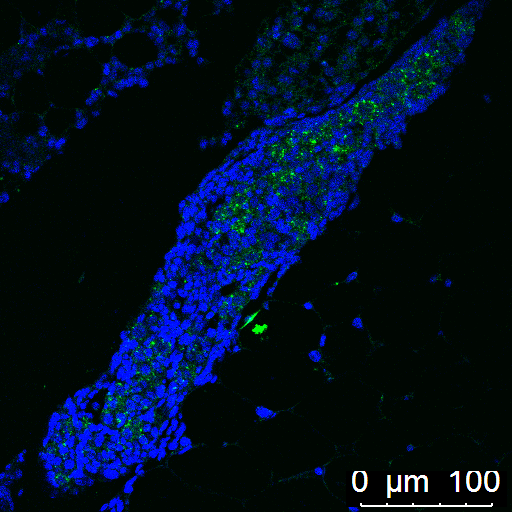

Supplement: Supplementary file 10 — EV Figures Source Data [file 44319_2026_775_MOESM10_ESM.zip › Figure EV1/Figure EV1A/19 dpf Merge.tif]

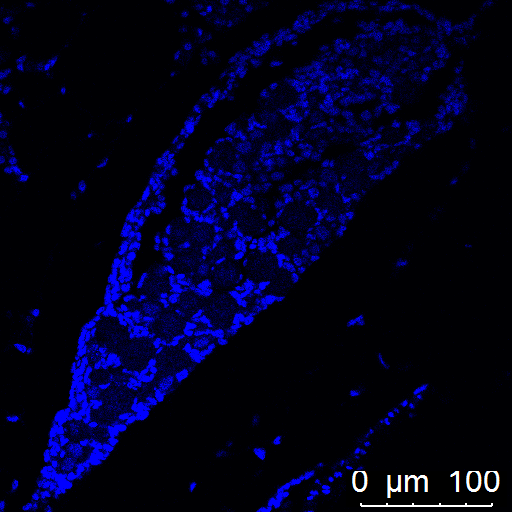

Supplement: Supplementary file 10 — EV Figures Source Data [file 44319_2026_775_MOESM10_ESM.zip › Figure EV1/Figure EV1A/25 dpf DAPI.tif]

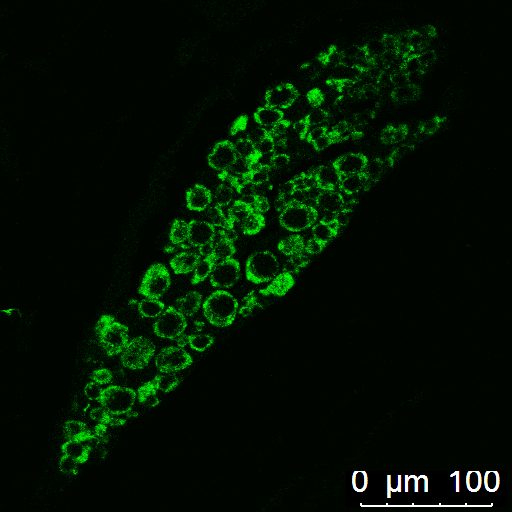

Supplement: Supplementary file 10 — EV Figures Source Data [file 44319_2026_775_MOESM10_ESM.zip › Figure EV1/Figure EV1A/25 dpf gfp.tif]

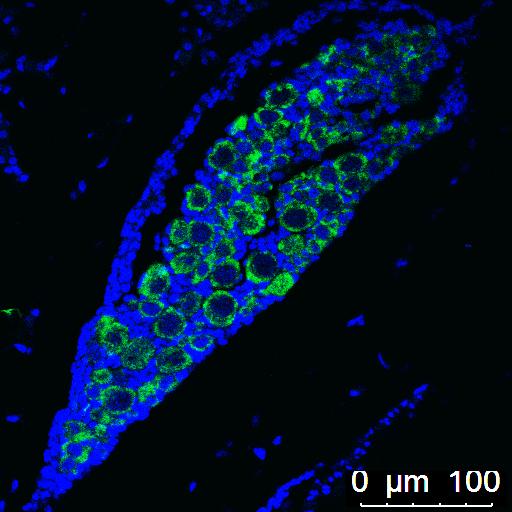

Supplement: Supplementary file 10 — EV Figures Source Data [file 44319_2026_775_MOESM10_ESM.zip › Figure EV1/Figure EV1A/25 dpf Merge.tif]

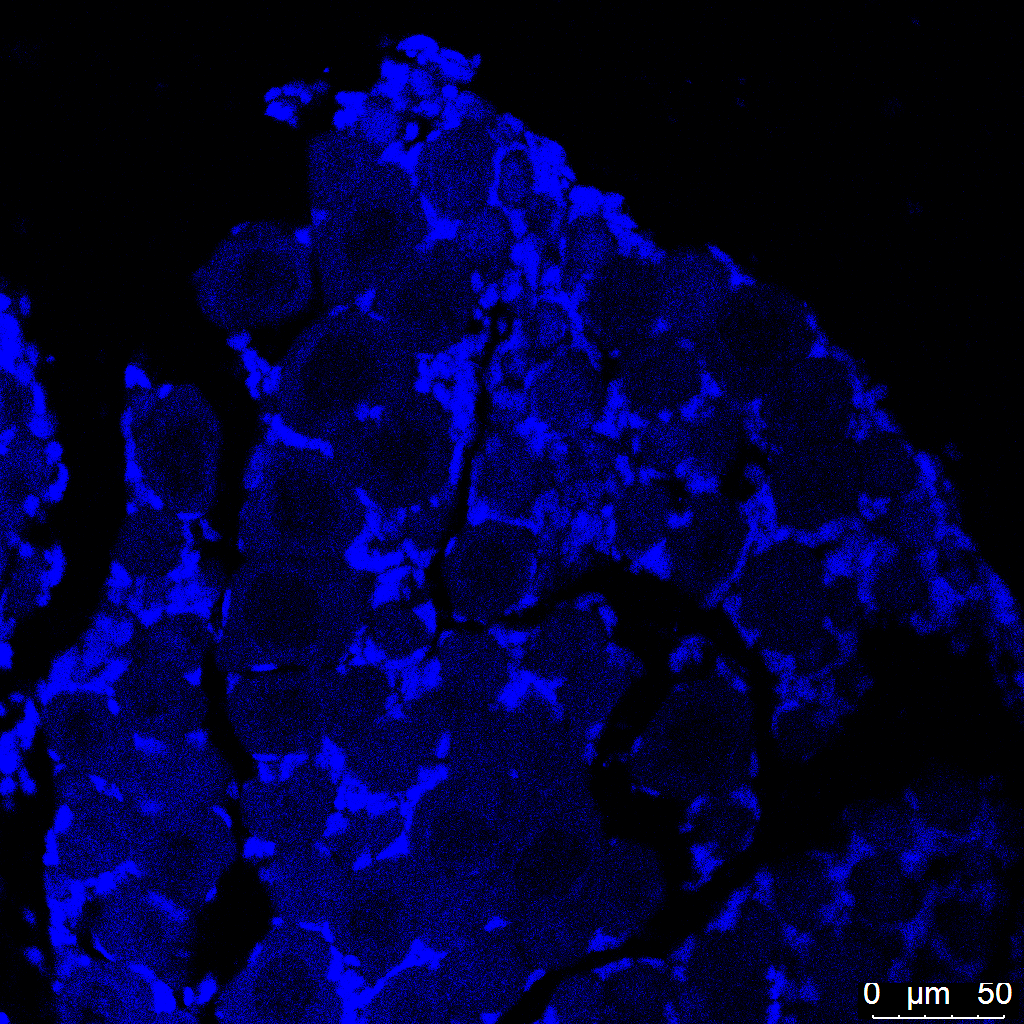

Supplement: Supplementary file 10 — EV Figures Source Data [file 44319_2026_775_MOESM10_ESM.zip › Figure EV1/Figure EV1A/33 dpf DAPI.tif]

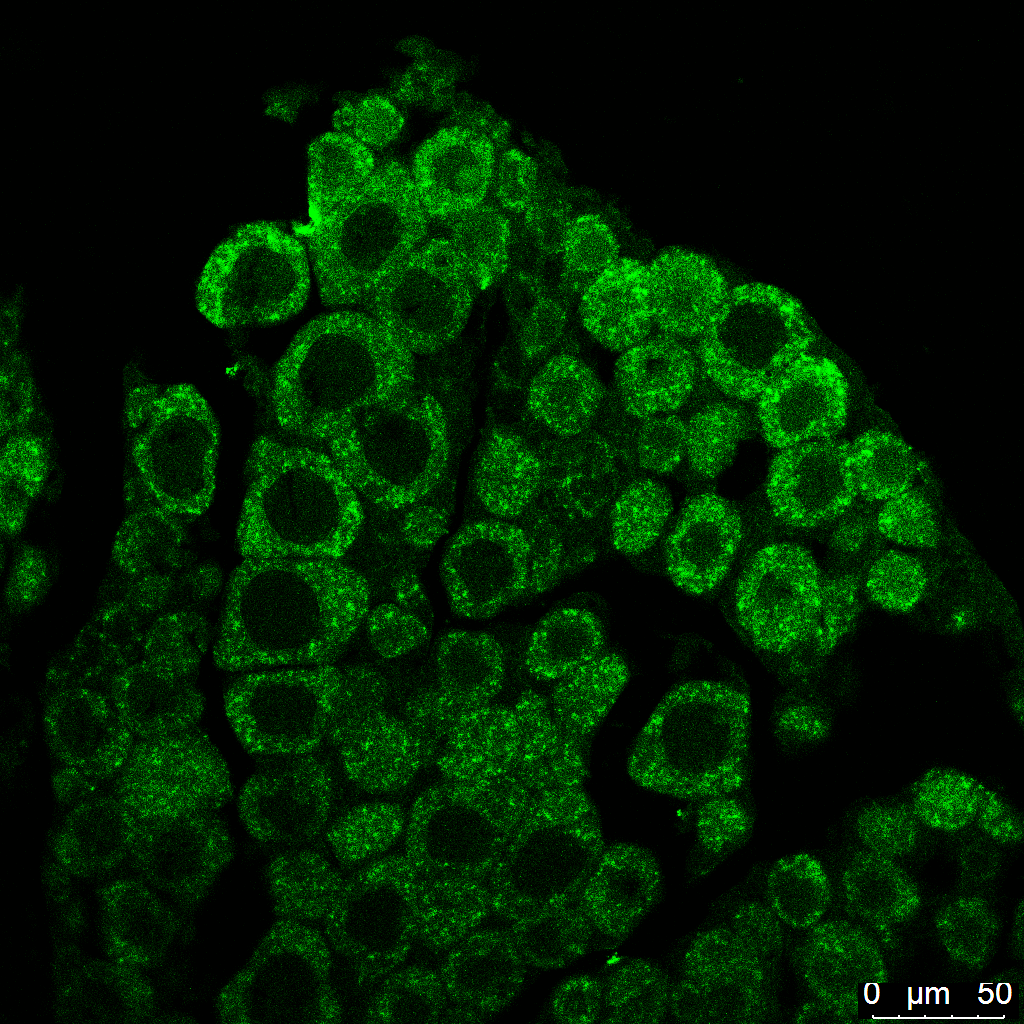

Supplement: Supplementary file 10 — EV Figures Source Data [file 44319_2026_775_MOESM10_ESM.zip › Figure EV1/Figure EV1A/33 dpf gfp.tif]

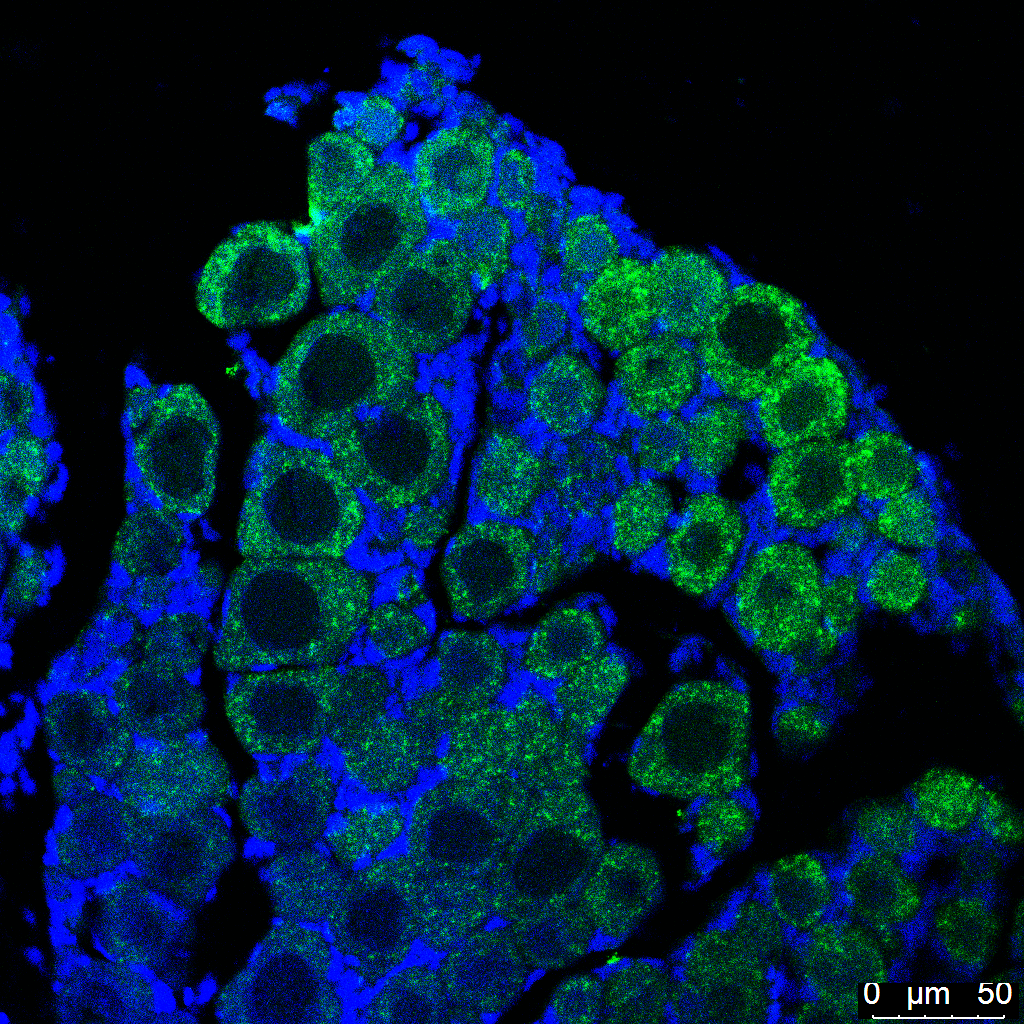

Supplement: Supplementary file 10 — EV Figures Source Data [file 44319_2026_775_MOESM10_ESM.zip › Figure EV1/Figure EV1A/33 dpf Merge.tif]

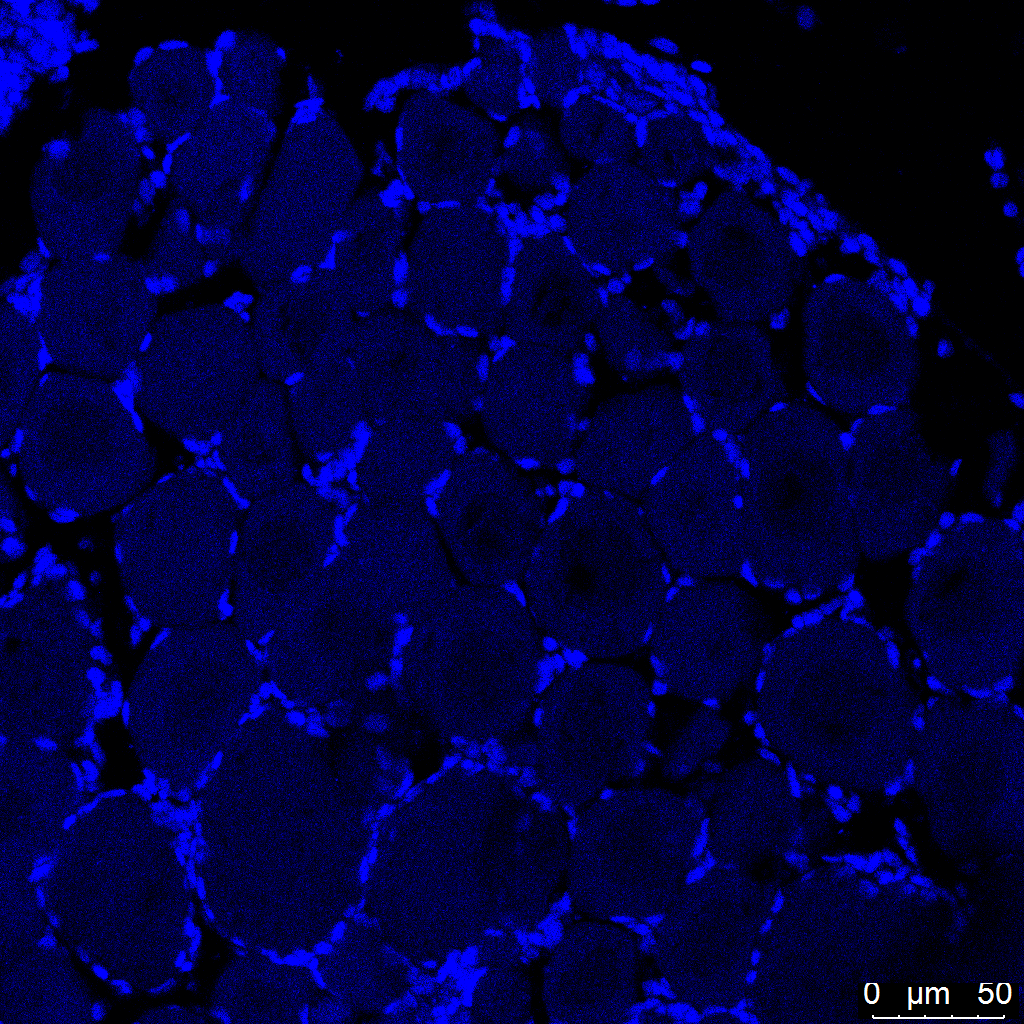

Supplement: Supplementary file 10 — EV Figures Source Data [file 44319_2026_775_MOESM10_ESM.zip › Figure EV1/Figure EV1A/45 dpf DAPI.tif]

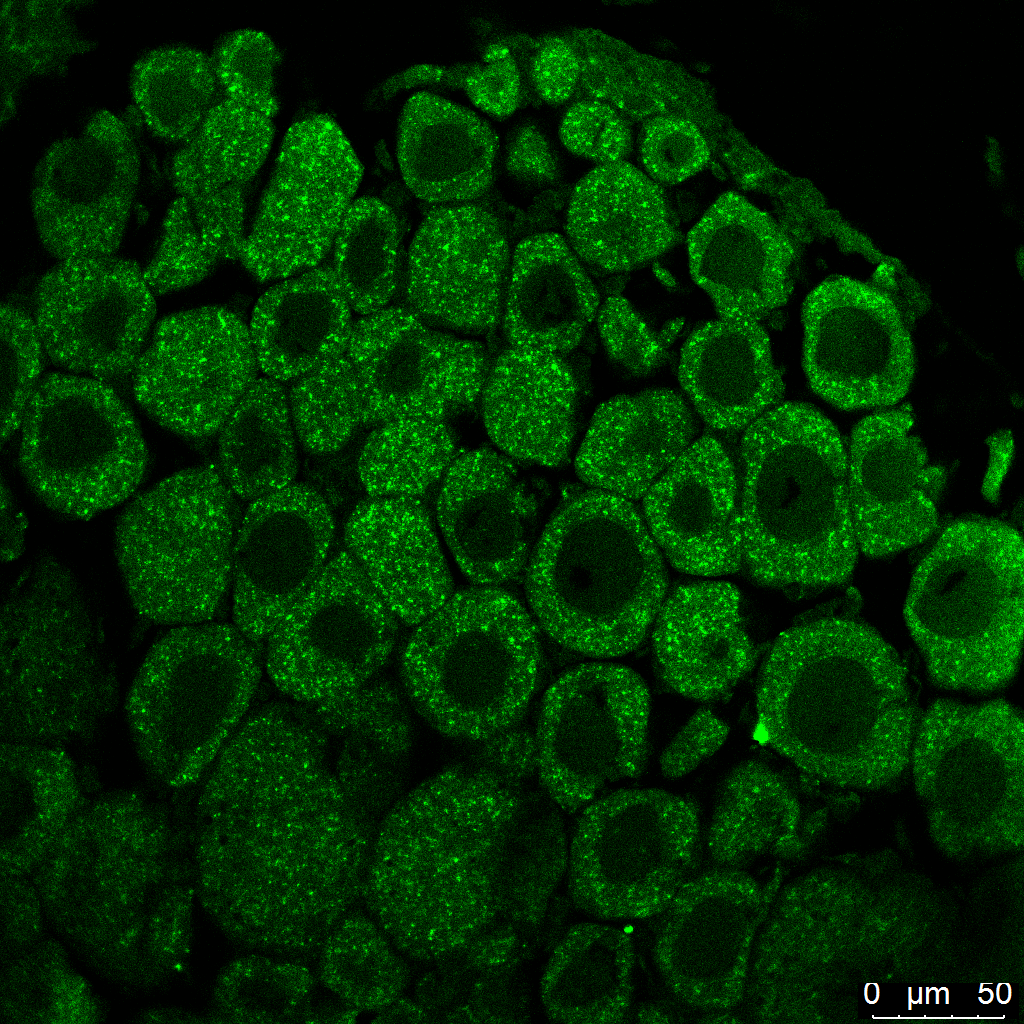

Supplement: Supplementary file 10 — EV Figures Source Data [file 44319_2026_775_MOESM10_ESM.zip › Figure EV1/Figure EV1A/45 dpf gfp.tif]

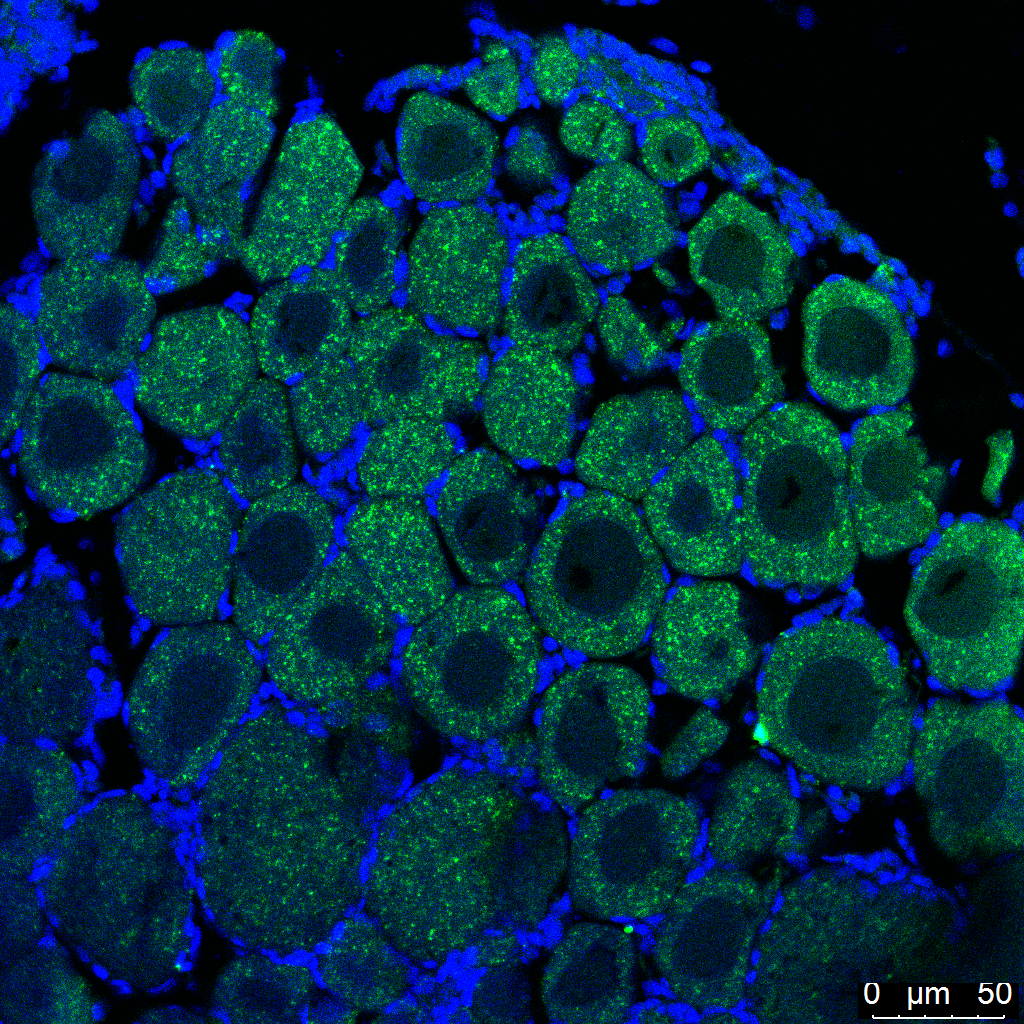

Supplement: Supplementary file 10 — EV Figures Source Data [file 44319_2026_775_MOESM10_ESM.zip › Figure EV1/Figure EV1A/45 dpf Merge.tif]

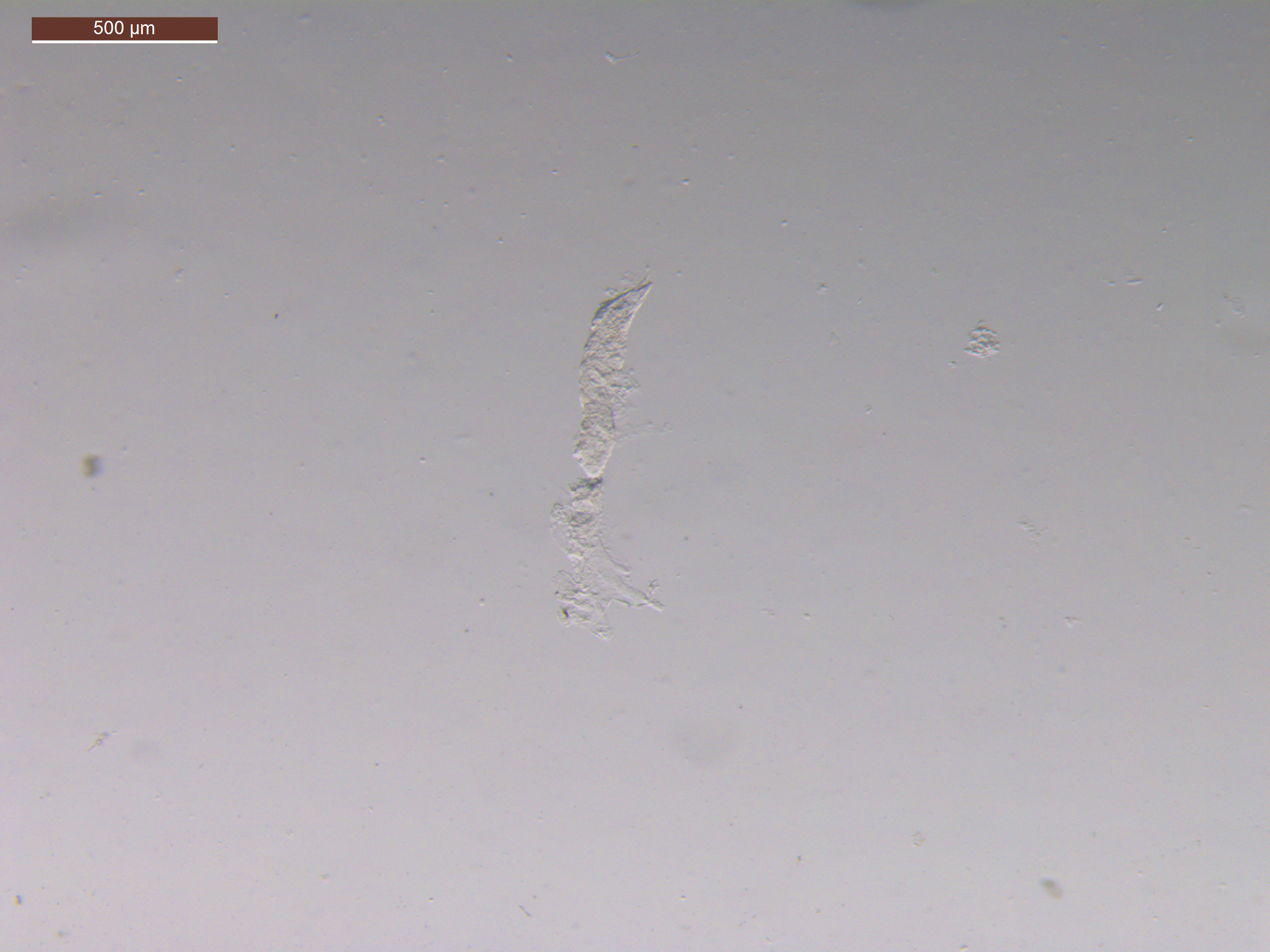

Supplement: Supplementary file 10 — EV Figures Source Data [file 44319_2026_775_MOESM10_ESM.zip › Figure EV2/Figure EV2A/+7 line 19 dpf-hom.tif]

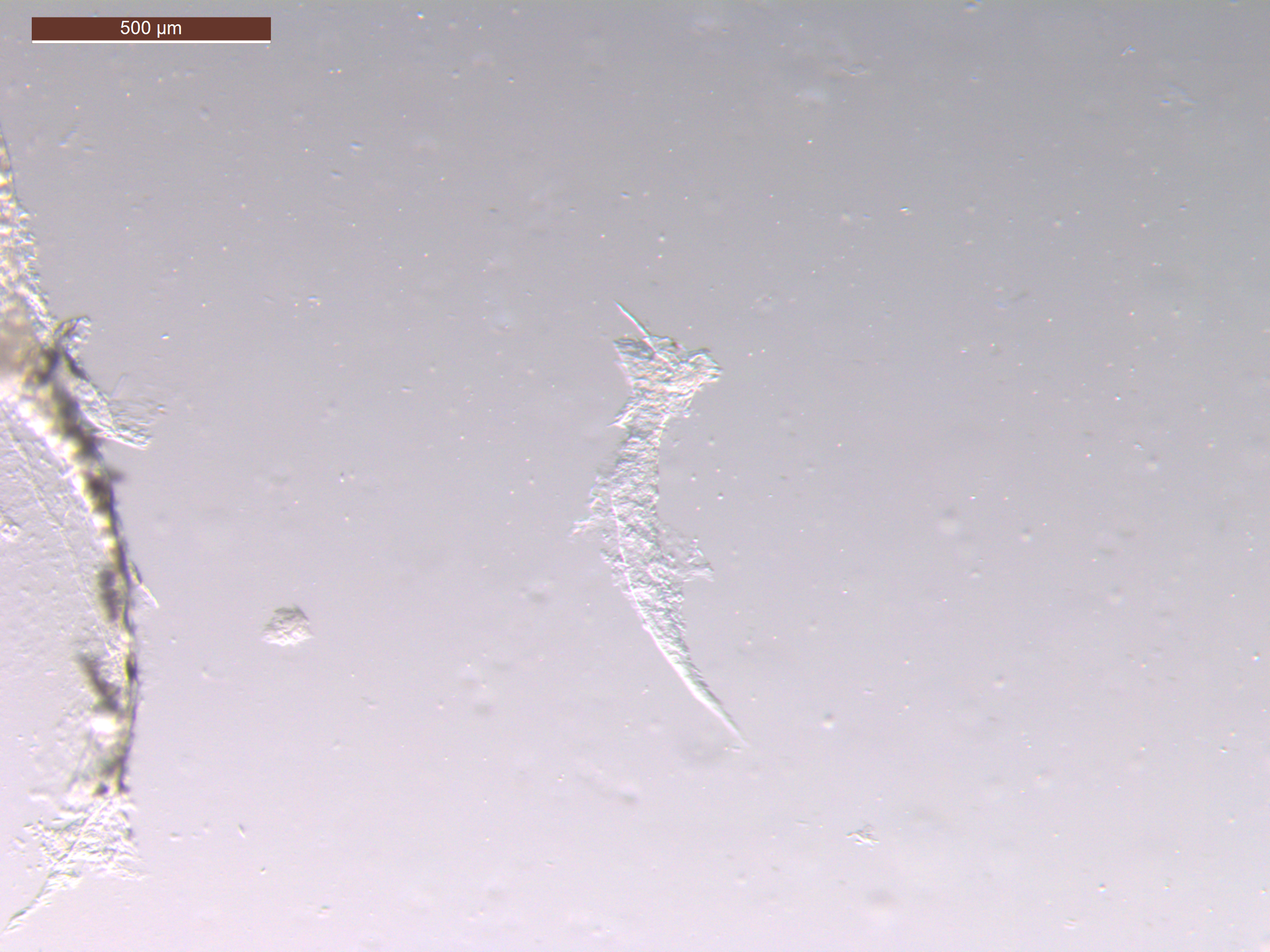

Supplement: Supplementary file 10 — EV Figures Source Data [file 44319_2026_775_MOESM10_ESM.zip › Figure EV2/Figure EV2A/+7 line 19 dpf-WT.tif]

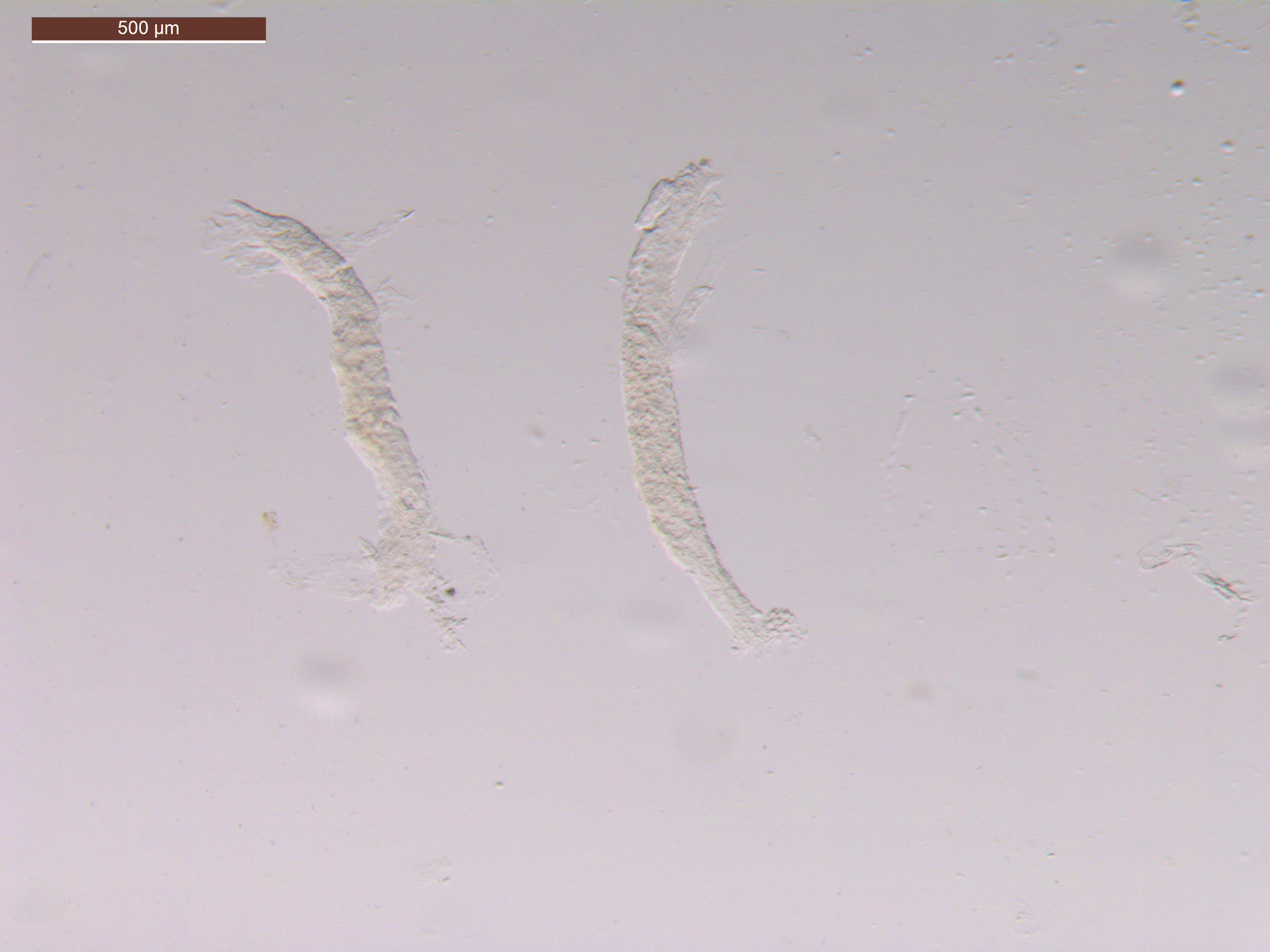

Supplement: Supplementary file 10 — EV Figures Source Data [file 44319_2026_775_MOESM10_ESM.zip › Figure EV2/Figure EV2A/+7 line 25 dpf-hom.tif]

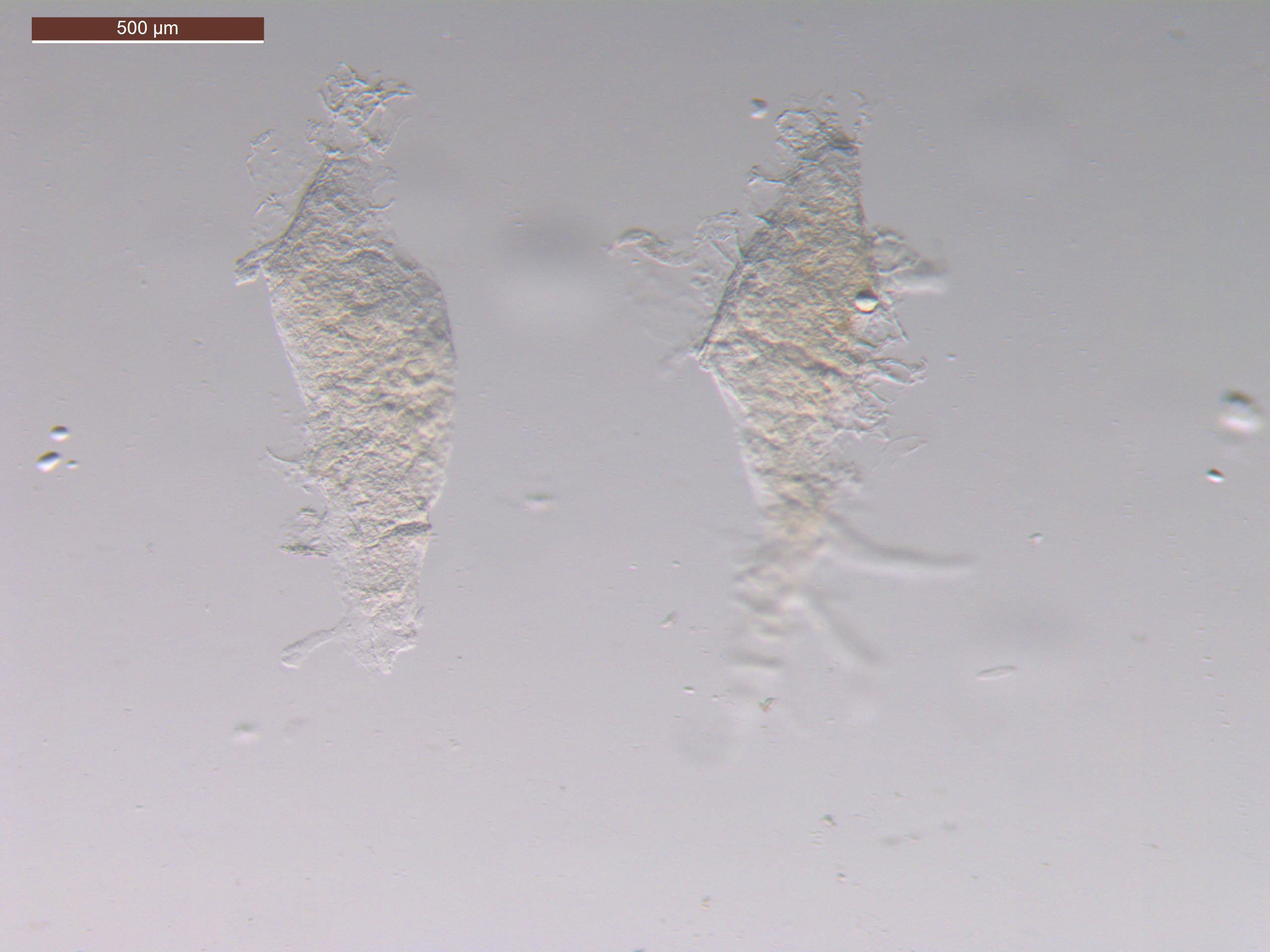

Supplement: Supplementary file 10 — EV Figures Source Data [file 44319_2026_775_MOESM10_ESM.zip › Figure EV2/Figure EV2A/+7 line 25 dpf-WT ovary.tif]

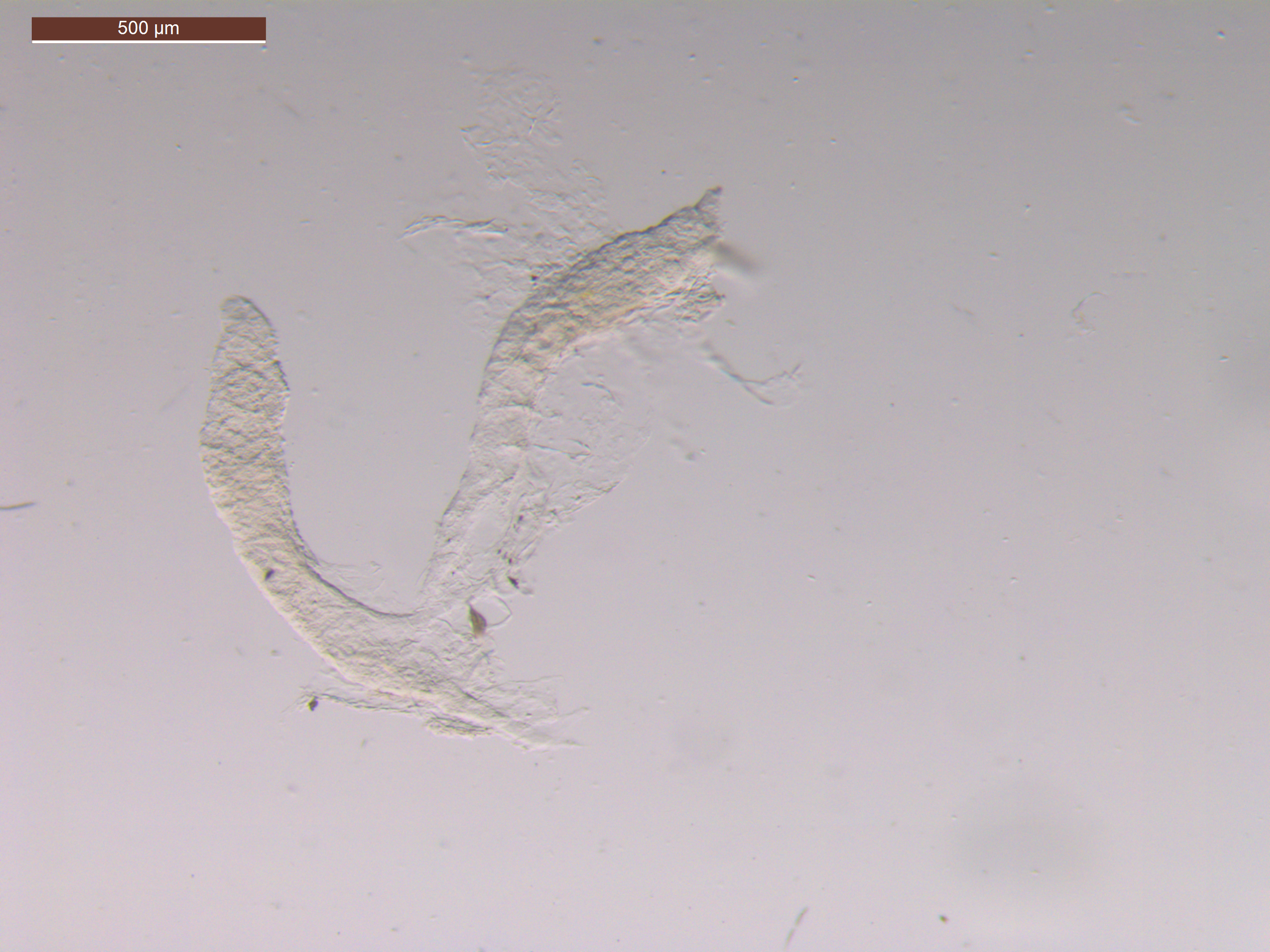

Supplement: Supplementary file 10 — EV Figures Source Data [file 44319_2026_775_MOESM10_ESM.zip › Figure EV2/Figure EV2A/+7 line 25 dpf-WT testis.tif]

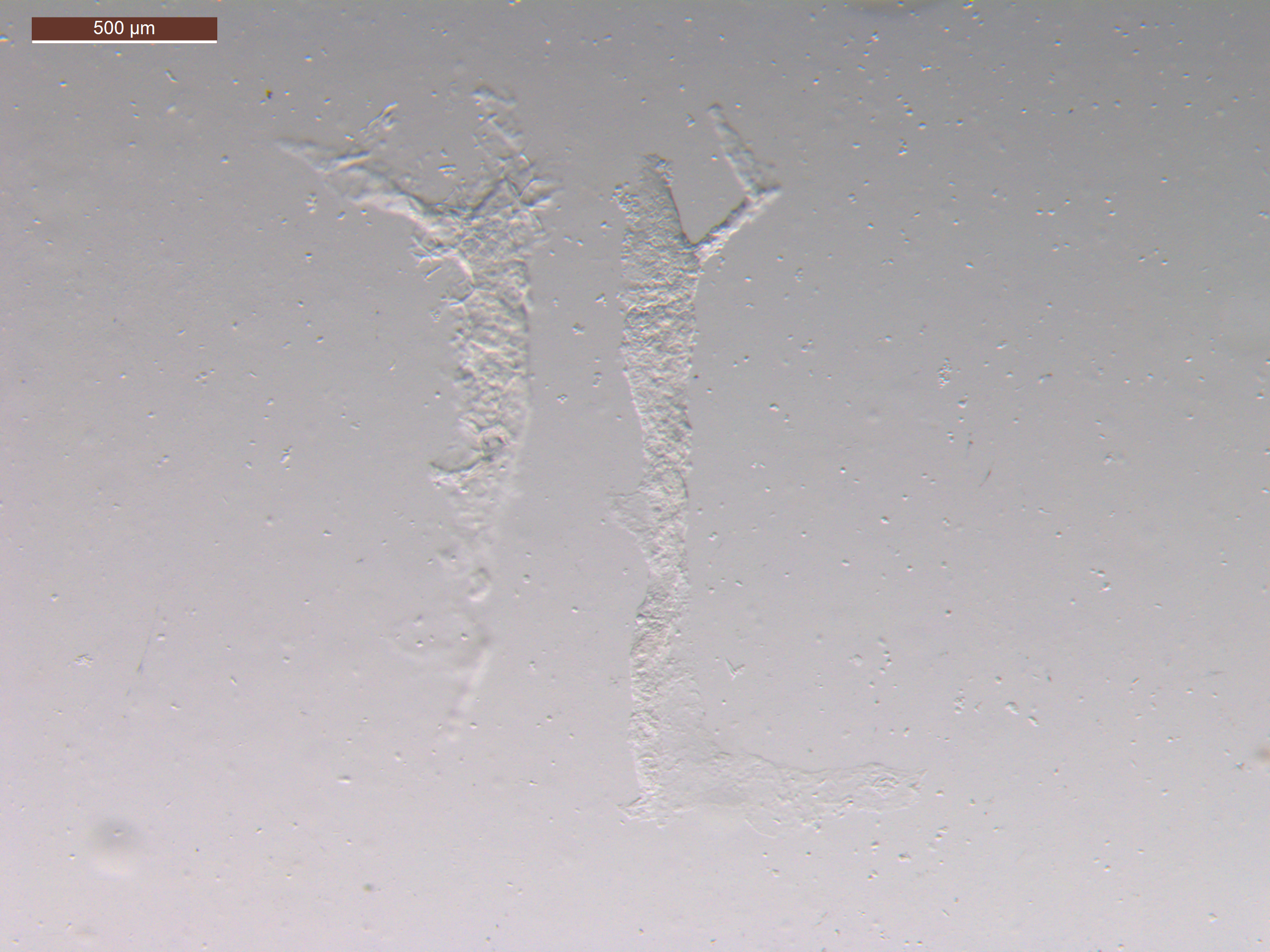

Supplement: Supplementary file 10 — EV Figures Source Data [file 44319_2026_775_MOESM10_ESM.zip › Figure EV2/Figure EV2A/+7 line 33 dpf-hom.tif]

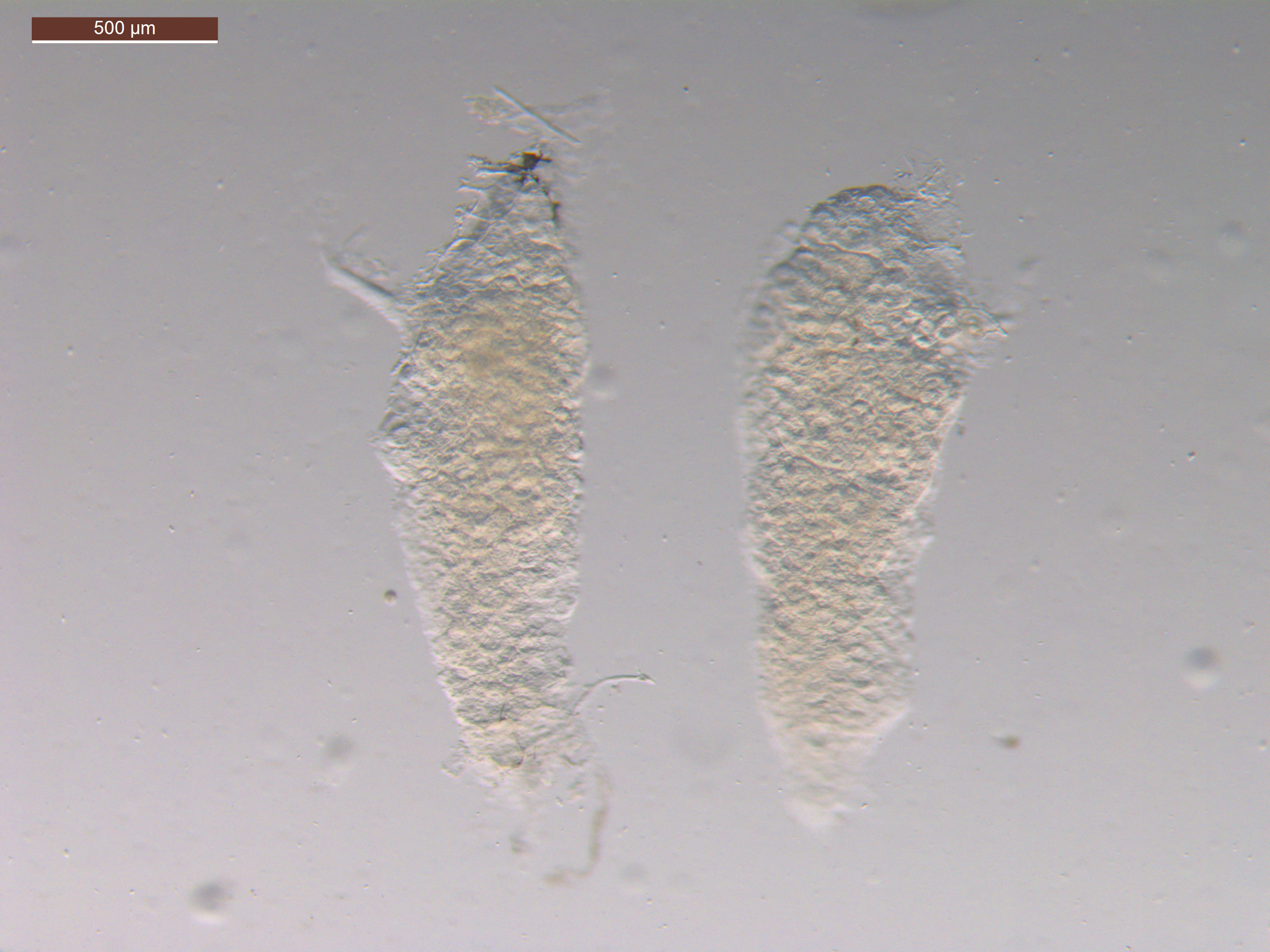

Supplement: Supplementary file 10 — EV Figures Source Data [file 44319_2026_775_MOESM10_ESM.zip › Figure EV2/Figure EV2A/+7 line 33 dpf-WT ovary.tif]

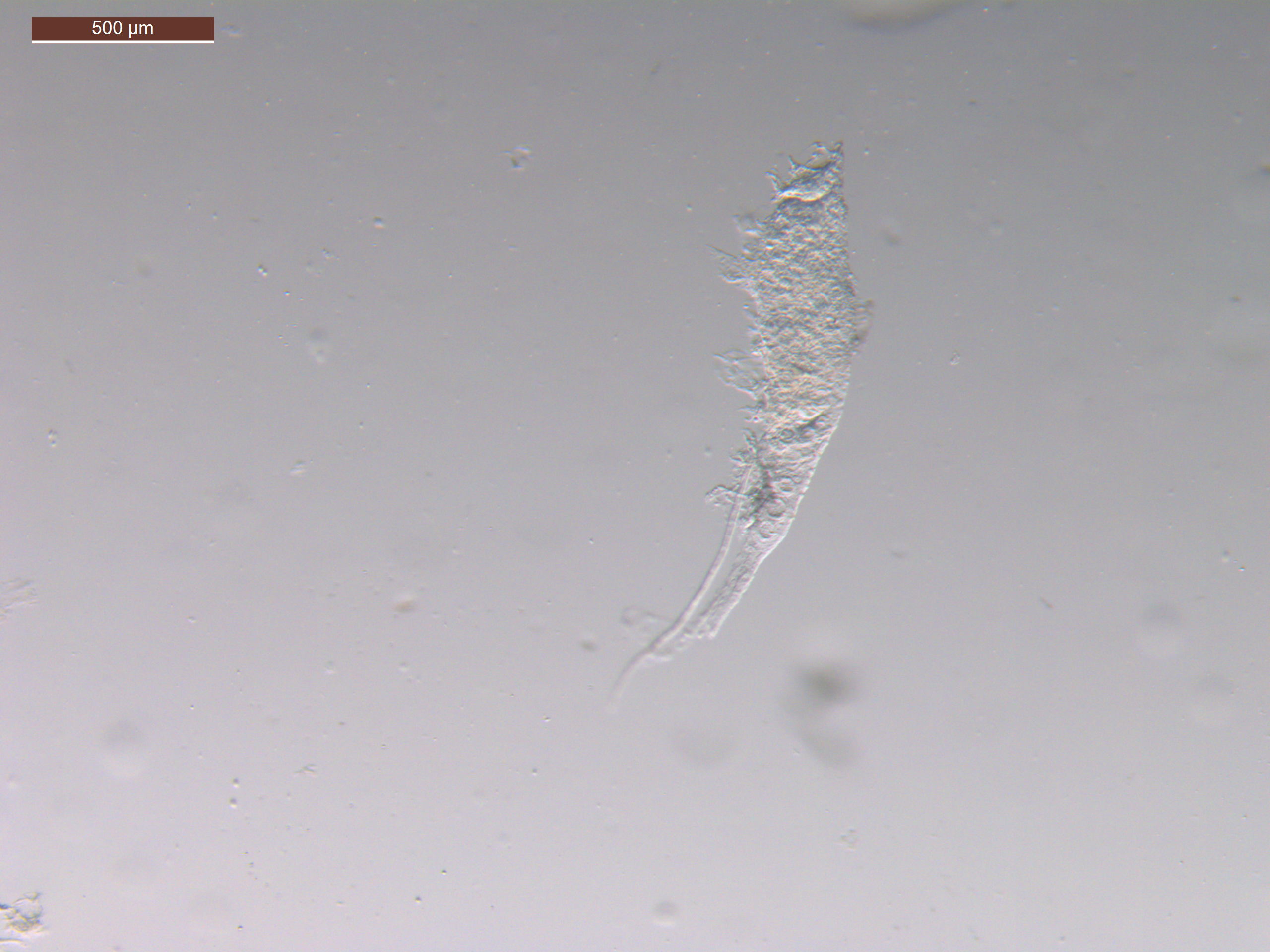

Supplement: Supplementary file 10 — EV Figures Source Data [file 44319_2026_775_MOESM10_ESM.zip › Figure EV2/Figure EV2A/+7 line 33 dpf-WT testis.tif]

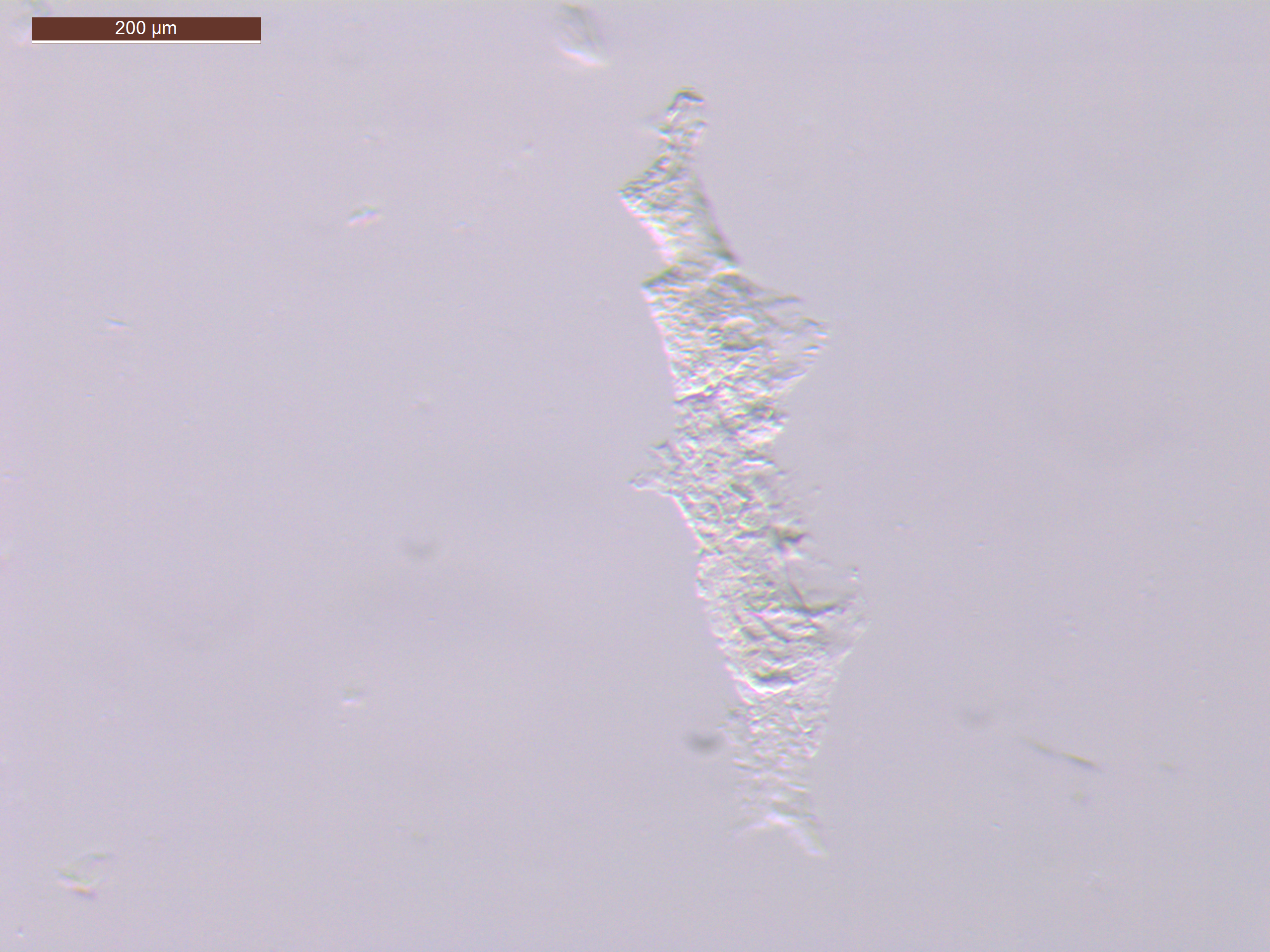

Supplement: Supplementary file 10 — EV Figures Source Data [file 44319_2026_775_MOESM10_ESM.zip › Figure EV2/Figure EV2A/Δ7 line 19 dpf-hom.tif]

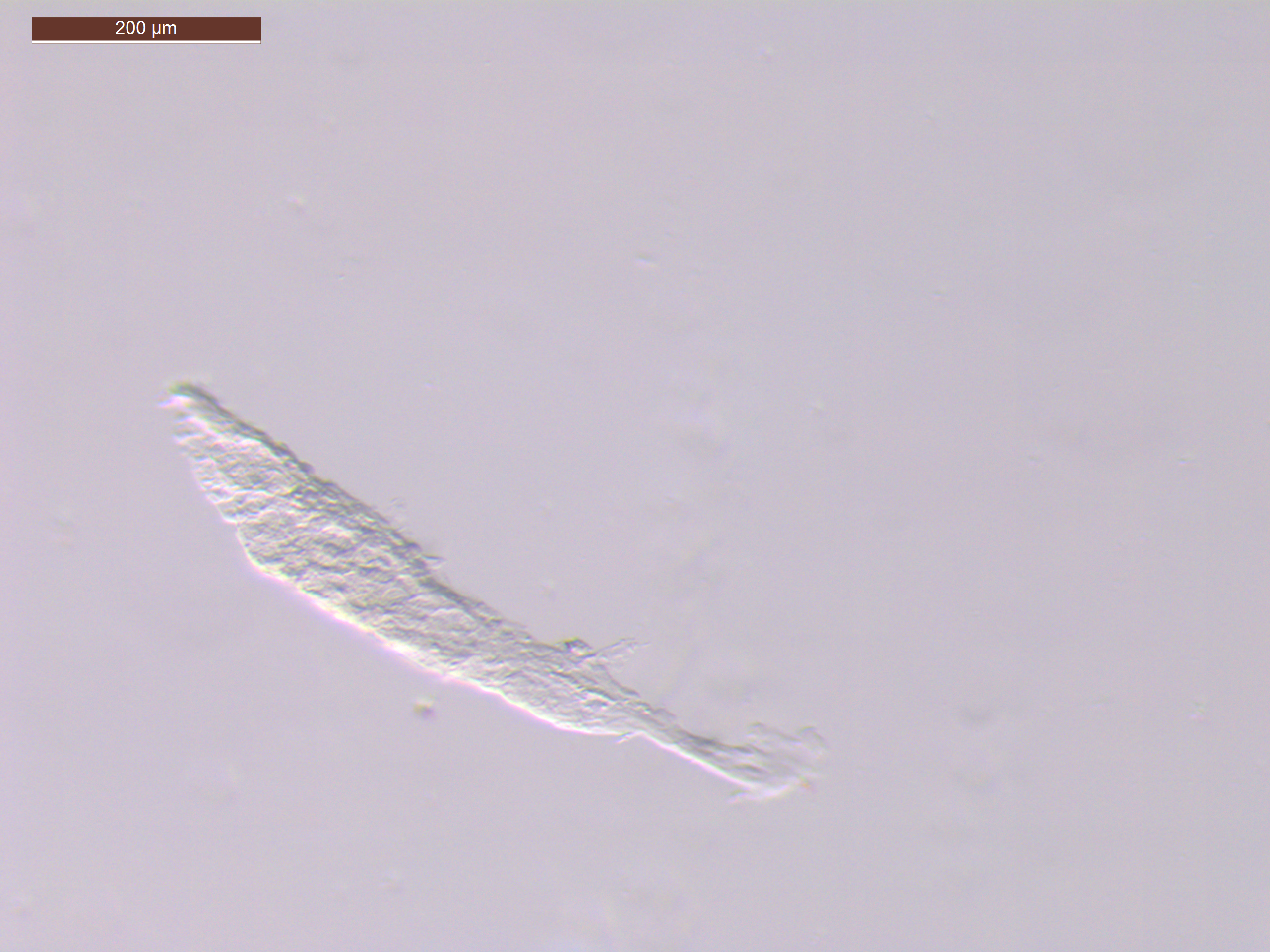

Supplement: Supplementary file 10 — EV Figures Source Data [file 44319_2026_775_MOESM10_ESM.zip › Figure EV2/Figure EV2A/Δ7 line 19 dpf-WT.tif]

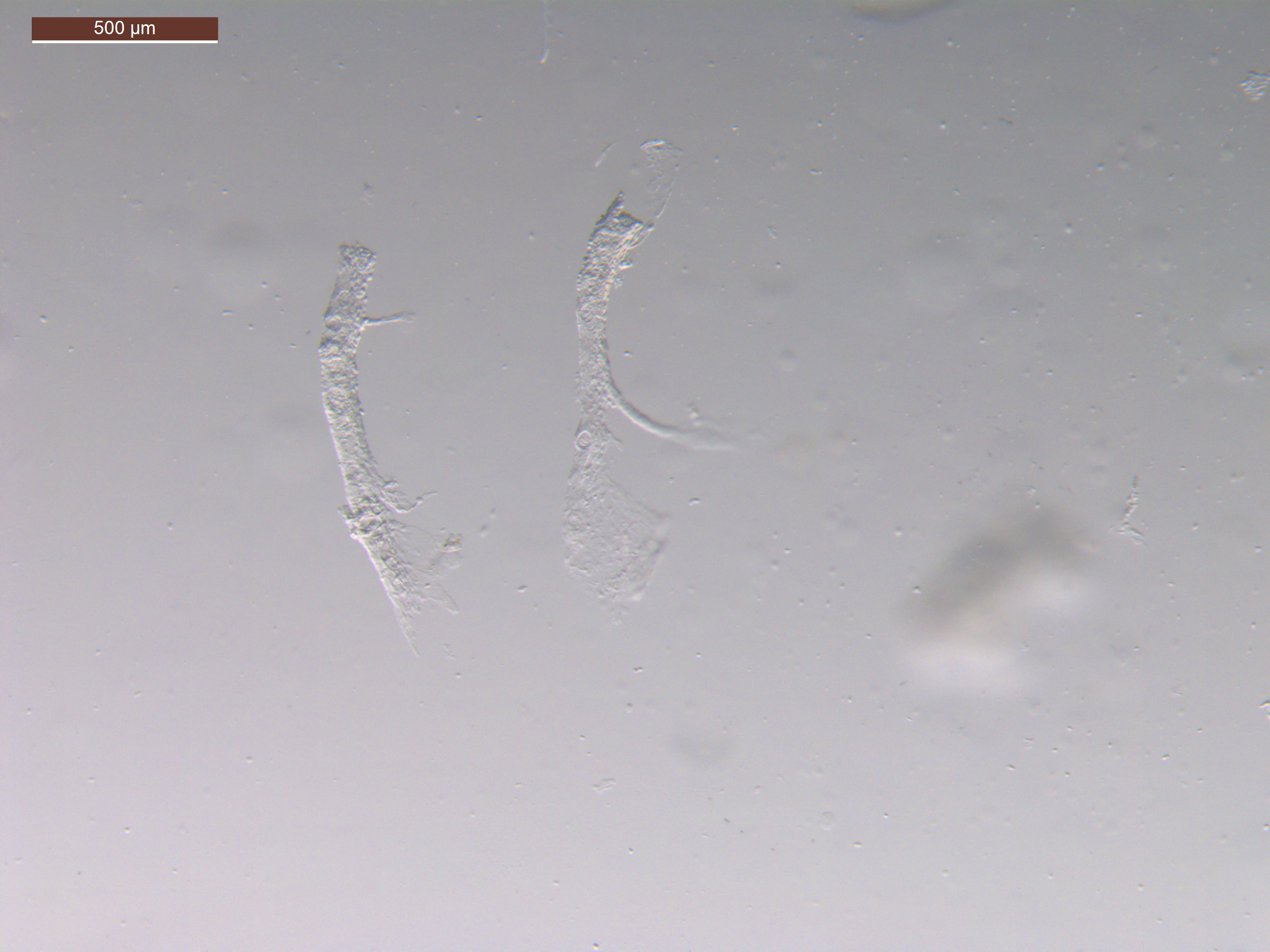

Supplement: Supplementary file 10 — EV Figures Source Data [file 44319_2026_775_MOESM10_ESM.zip › Figure EV2/Figure EV2A/Δ7 line 25 dpf-hom.tif]

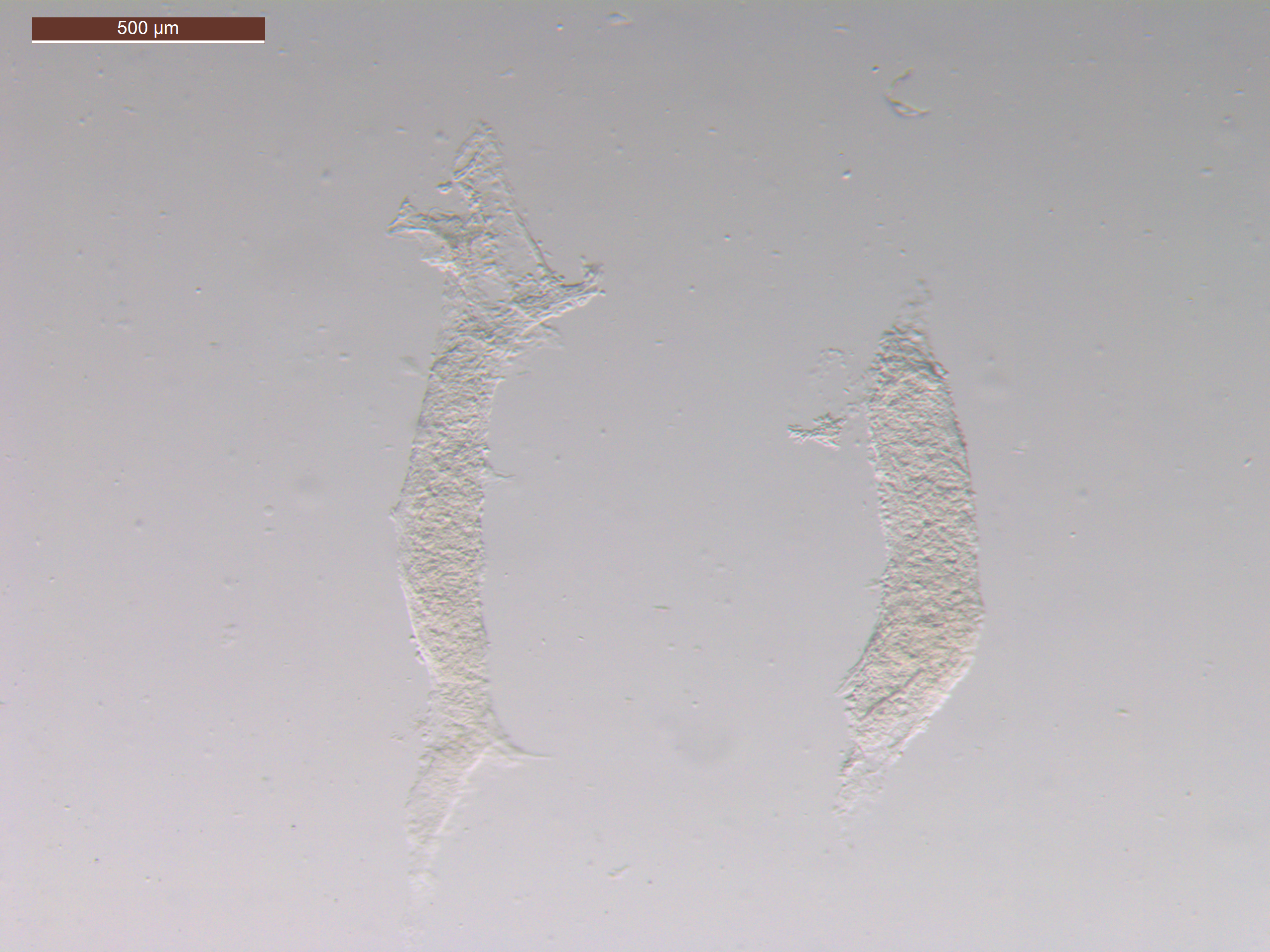

Supplement: Supplementary file 10 — EV Figures Source Data [file 44319_2026_775_MOESM10_ESM.zip › Figure EV2/Figure EV2A/Δ7 line 25 dpf-WT ovary.tif]

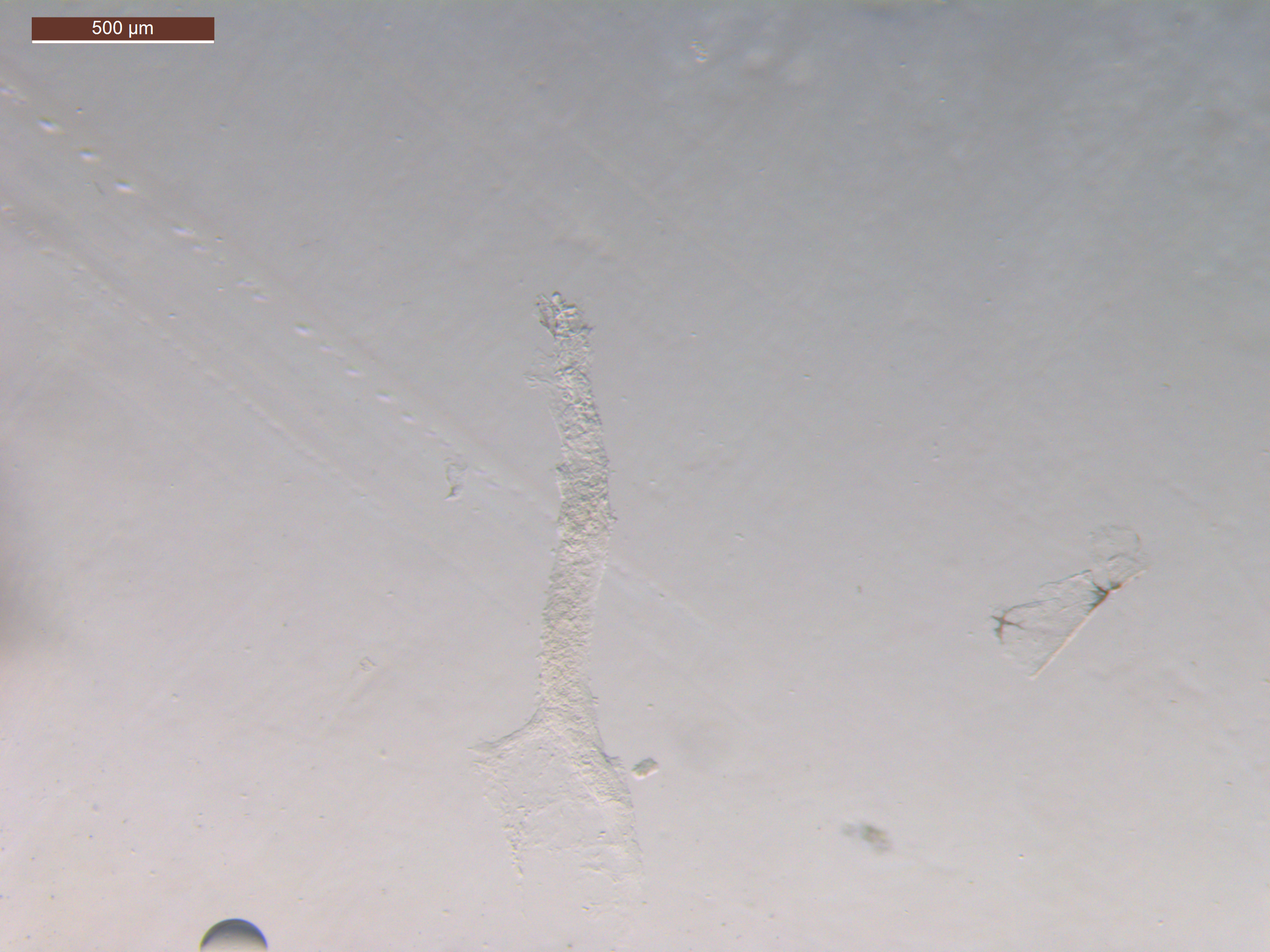

Supplement: Supplementary file 10 — EV Figures Source Data [file 44319_2026_775_MOESM10_ESM.zip › Figure EV2/Figure EV2A/Δ7 line 25 dpf-WT testis.tif]

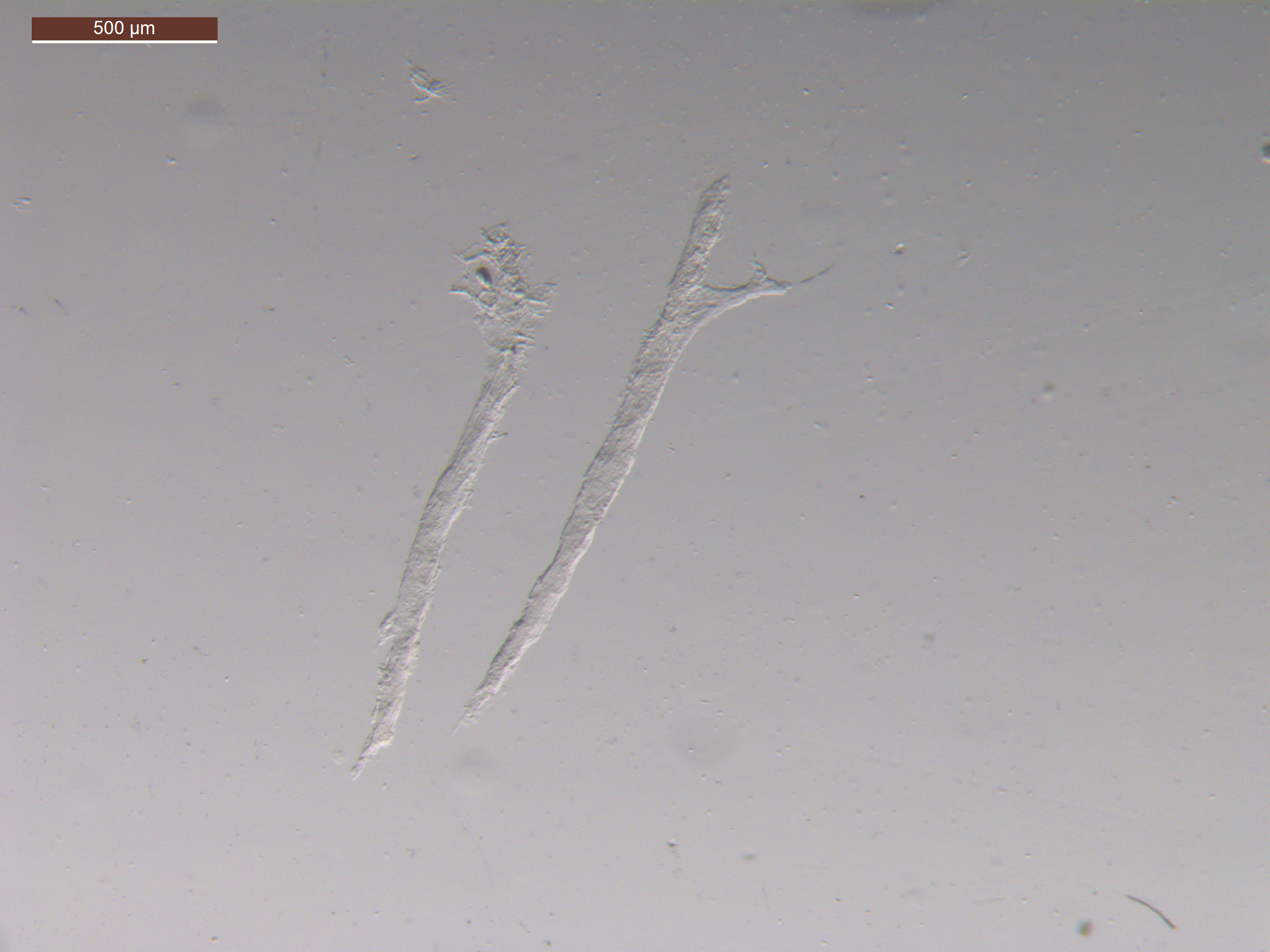

Supplement: Supplementary file 10 — EV Figures Source Data [file 44319_2026_775_MOESM10_ESM.zip › Figure EV2/Figure EV2A/Δ7 line 33 dpf-hom.tif]

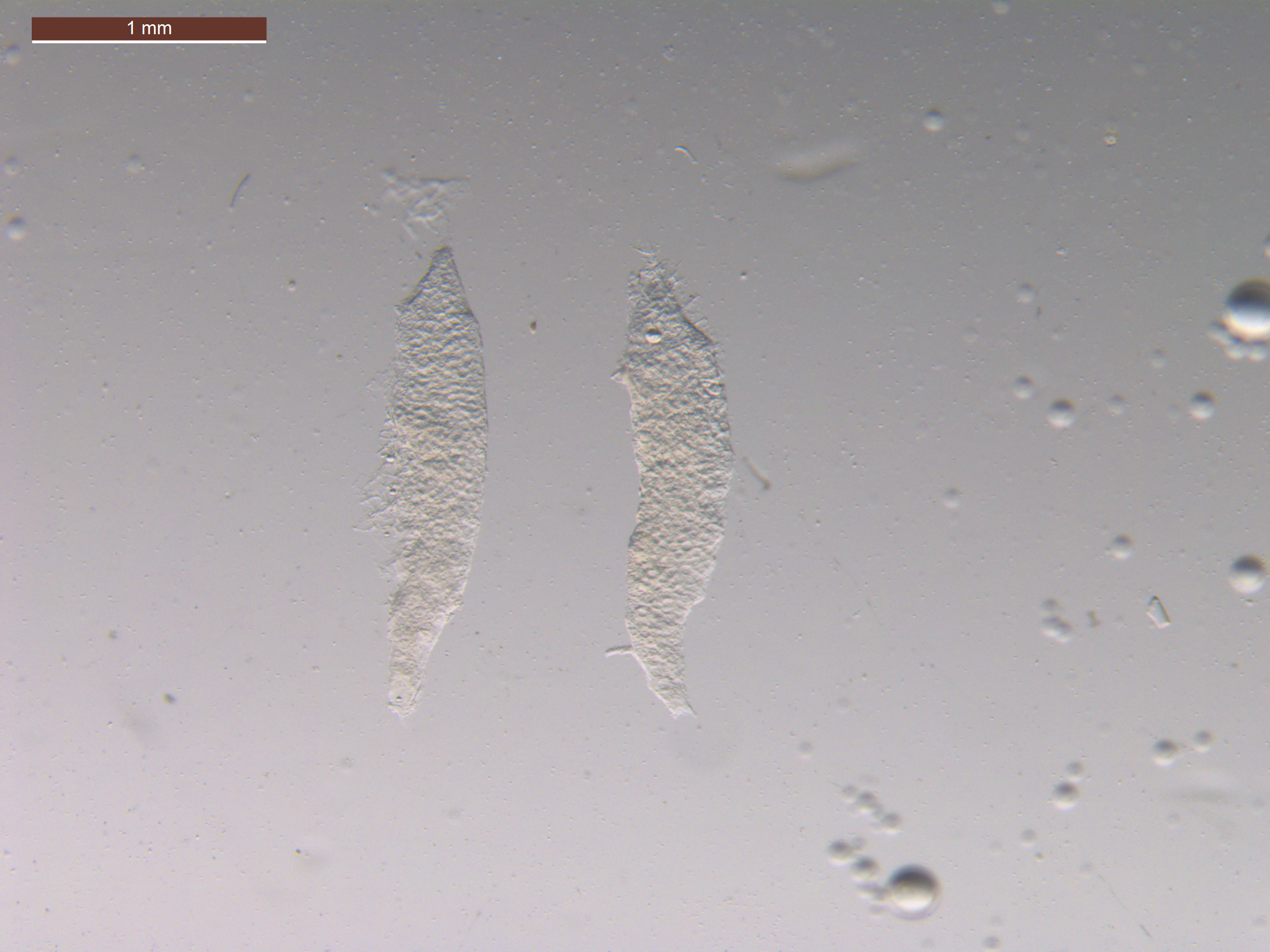

Supplement: Supplementary file 10 — EV Figures Source Data [file 44319_2026_775_MOESM10_ESM.zip › Figure EV2/Figure EV2A/Δ7 line 33 dpf-WT ovary.tif]

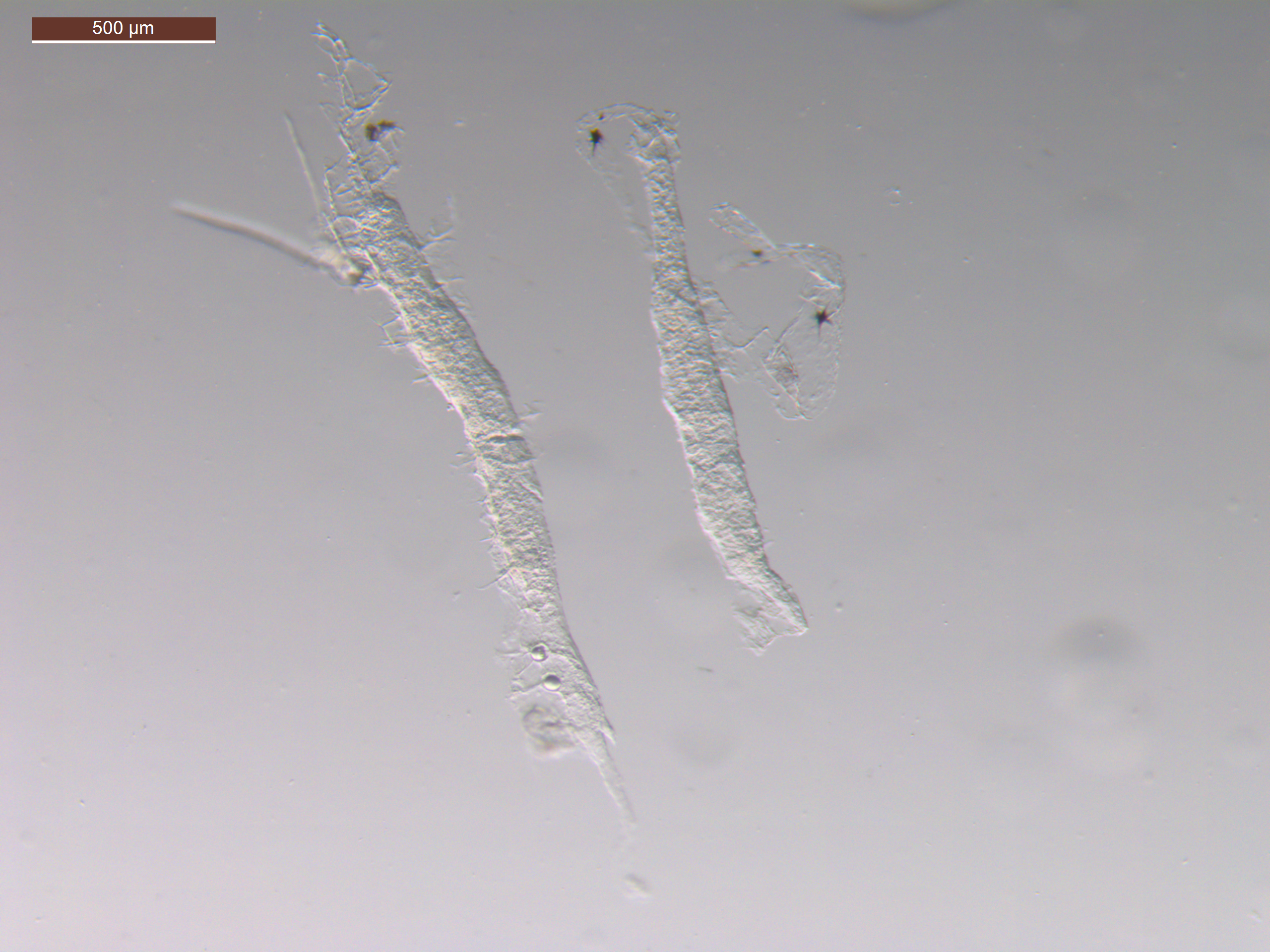

Supplement: Supplementary file 10 — EV Figures Source Data [file 44319_2026_775_MOESM10_ESM.zip › Figure EV2/Figure EV2A/Δ7 line 33 dpf-WT testis.tif]

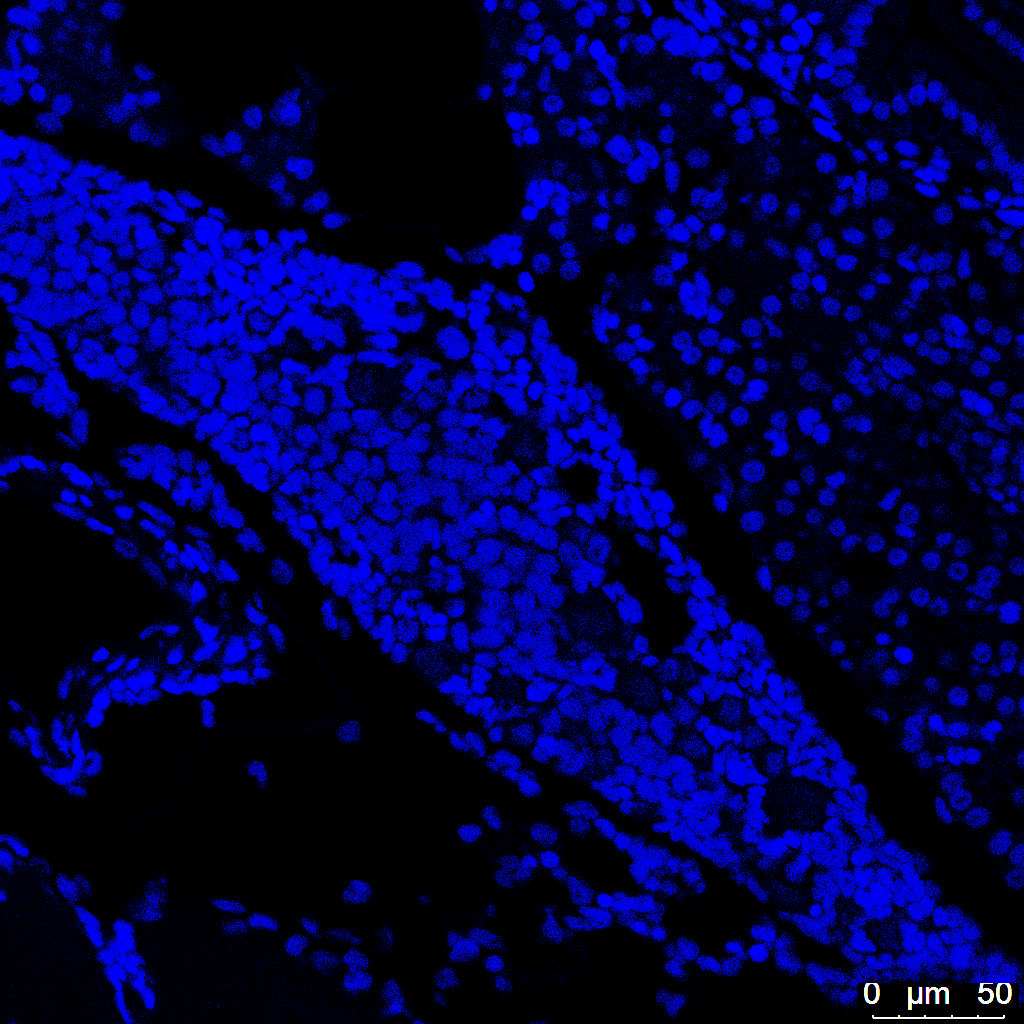

Supplement: Supplementary file 11 — Figure Source Data for Appendix Figures [file 44319_2026_775_MOESM11_ESM.zip › Source Data for Appendix Figure S1 3-7/Appendix Figure S1/Appendix Figure S1E/DAPI 19dpf.tif]

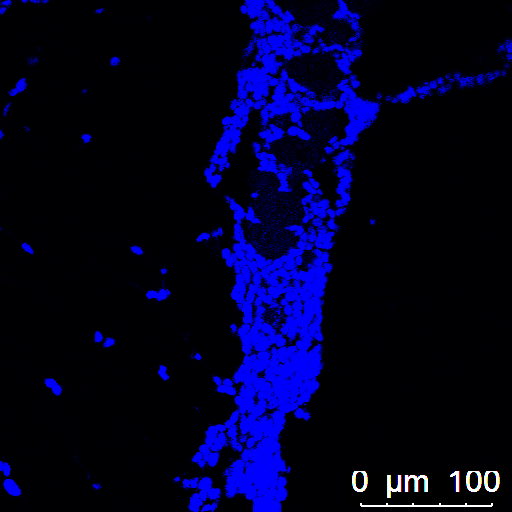

Supplement: Supplementary file 11 — Figure Source Data for Appendix Figures [file 44319_2026_775_MOESM11_ESM.zip › Source Data for Appendix Figure S1 3-7/Appendix Figure S1/Appendix Figure S1E/DAPI 25dpf.tif]

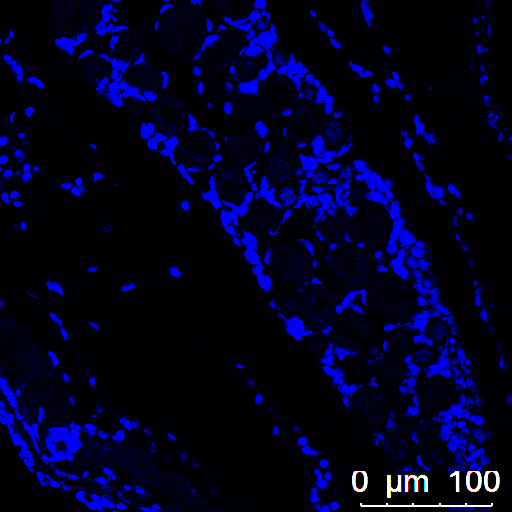

Supplement: Supplementary file 11 — Figure Source Data for Appendix Figures [file 44319_2026_775_MOESM11_ESM.zip › Source Data for Appendix Figure S1 3-7/Appendix Figure S1/Appendix Figure S1E/DAPI 33dpf .tif]

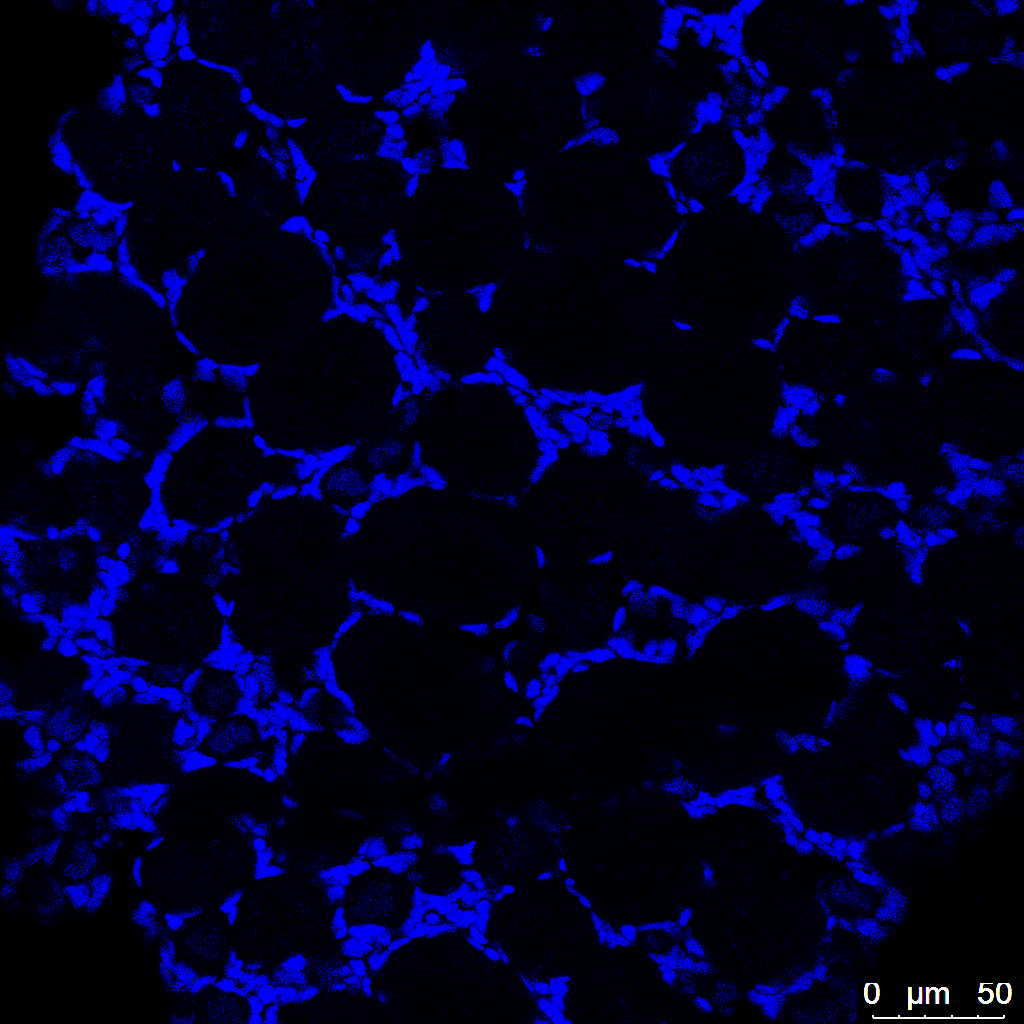

Supplement: Supplementary file 11 — Figure Source Data for Appendix Figures [file 44319_2026_775_MOESM11_ESM.zip › Source Data for Appendix Figure S1 3-7/Appendix Figure S1/Appendix Figure S1E/DAPI 45dpf.tif]

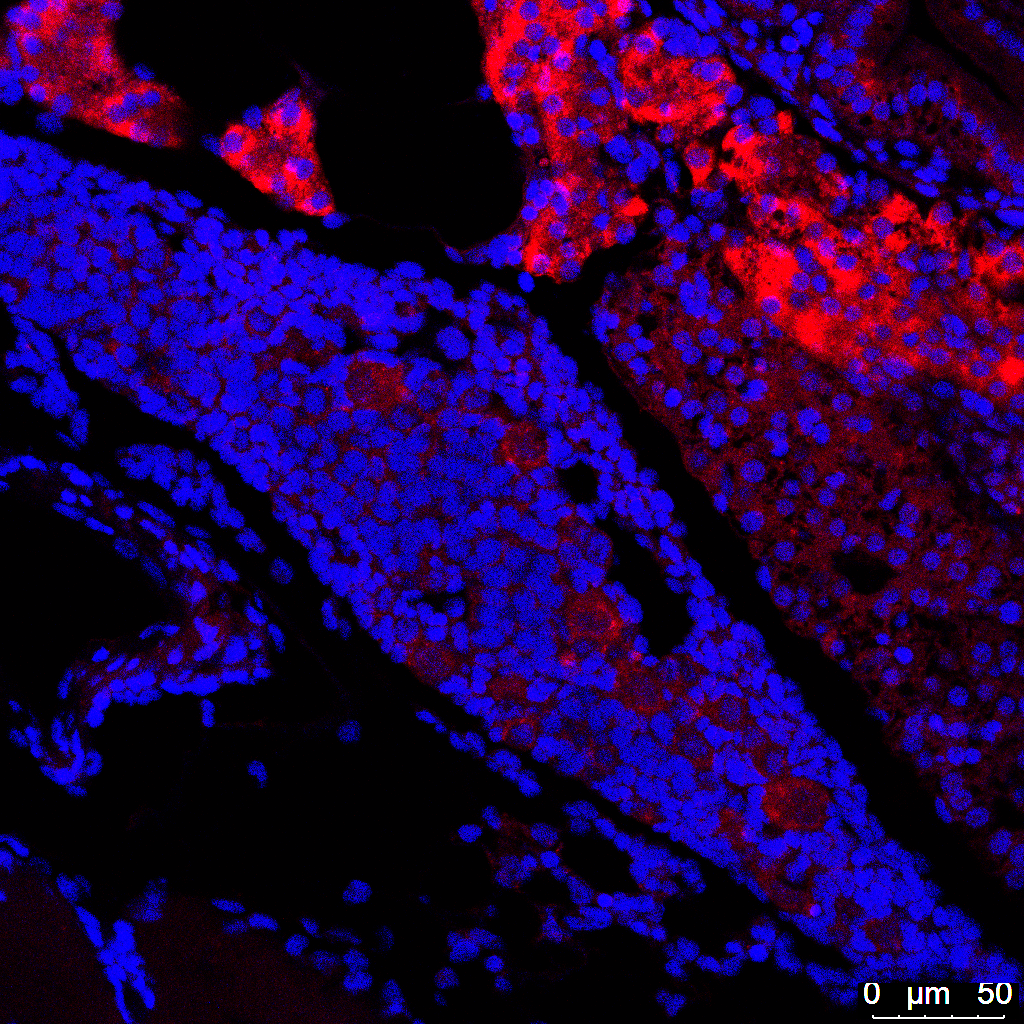

Supplement: Supplementary file 11 — Figure Source Data for Appendix Figures [file 44319_2026_775_MOESM11_ESM.zip › Source Data for Appendix Figure S1 3-7/Appendix Figure S1/Appendix Figure S1E/Merge 19dpf.tif]

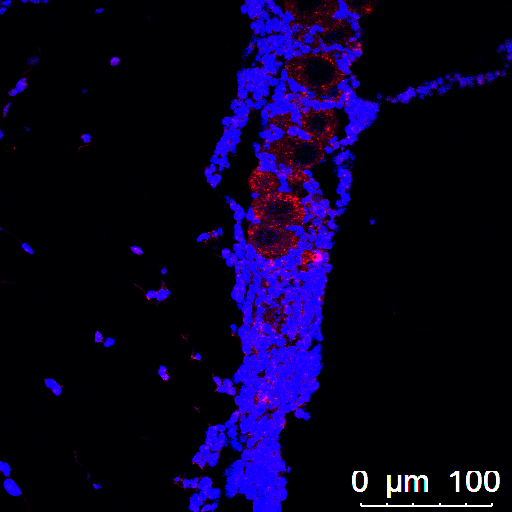

Supplement: Supplementary file 11 — Figure Source Data for Appendix Figures [file 44319_2026_775_MOESM11_ESM.zip › Source Data for Appendix Figure S1 3-7/Appendix Figure S1/Appendix Figure S1E/Merge 25dpf.tif]

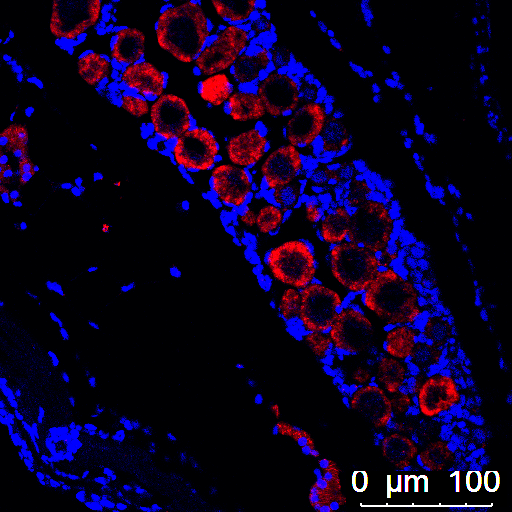

Supplement: Supplementary file 11 — Figure Source Data for Appendix Figures [file 44319_2026_775_MOESM11_ESM.zip › Source Data for Appendix Figure S1 3-7/Appendix Figure S1/Appendix Figure S1E/Merge 33dpf .tif]

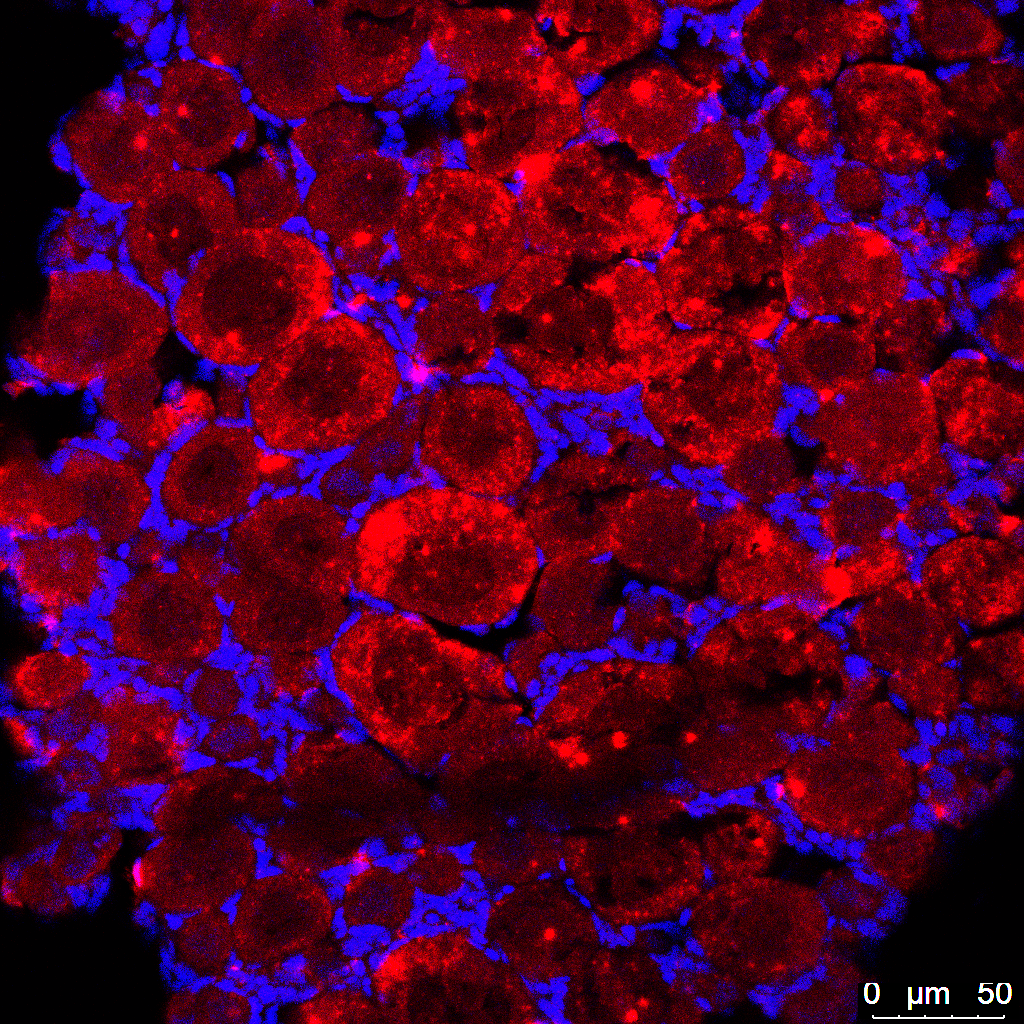

Supplement: Supplementary file 11 — Figure Source Data for Appendix Figures [file 44319_2026_775_MOESM11_ESM.zip › Source Data for Appendix Figure S1 3-7/Appendix Figure S1/Appendix Figure S1E/Merge 45dpf.tif]

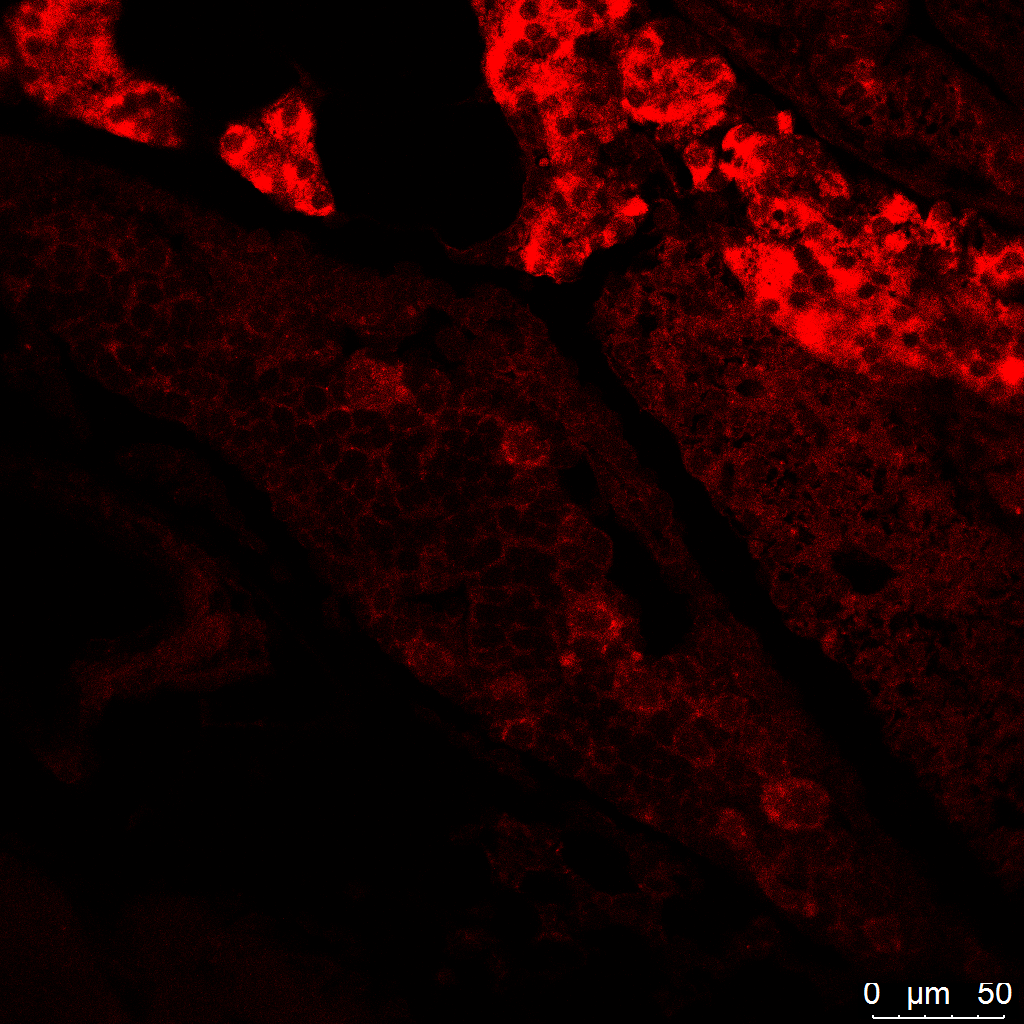

Supplement: Supplementary file 11 — Figure Source Data for Appendix Figures [file 44319_2026_775_MOESM11_ESM.zip › Source Data for Appendix Figure S1 3-7/Appendix Figure S1/Appendix Figure S1E/stn1 19dpf.tif]

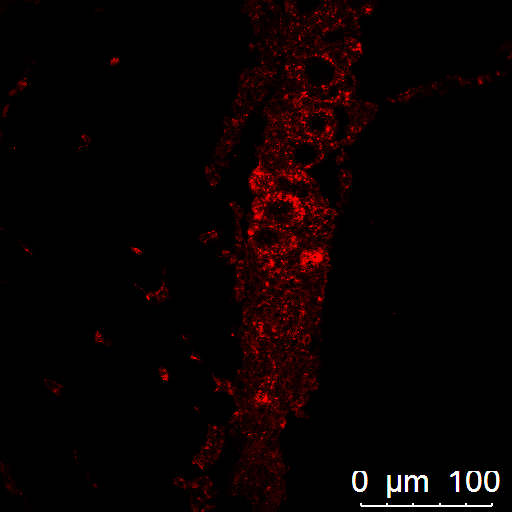

Supplement: Supplementary file 11 — Figure Source Data for Appendix Figures [file 44319_2026_775_MOESM11_ESM.zip › Source Data for Appendix Figure S1 3-7/Appendix Figure S1/Appendix Figure S1E/stn1 25dpf.tif]

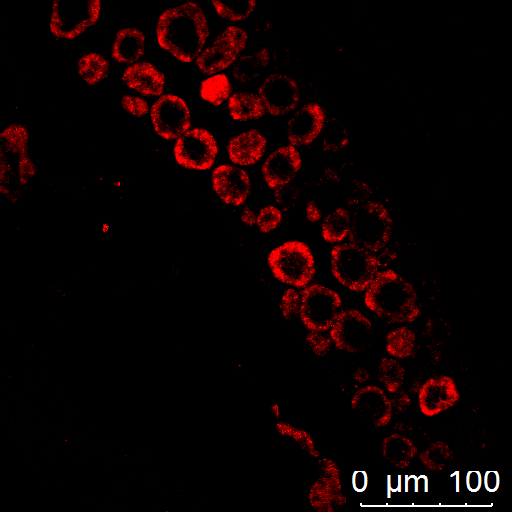

Supplement: Supplementary file 11 — Figure Source Data for Appendix Figures [file 44319_2026_775_MOESM11_ESM.zip › Source Data for Appendix Figure S1 3-7/Appendix Figure S1/Appendix Figure S1E/stn1 33dpf.tif]

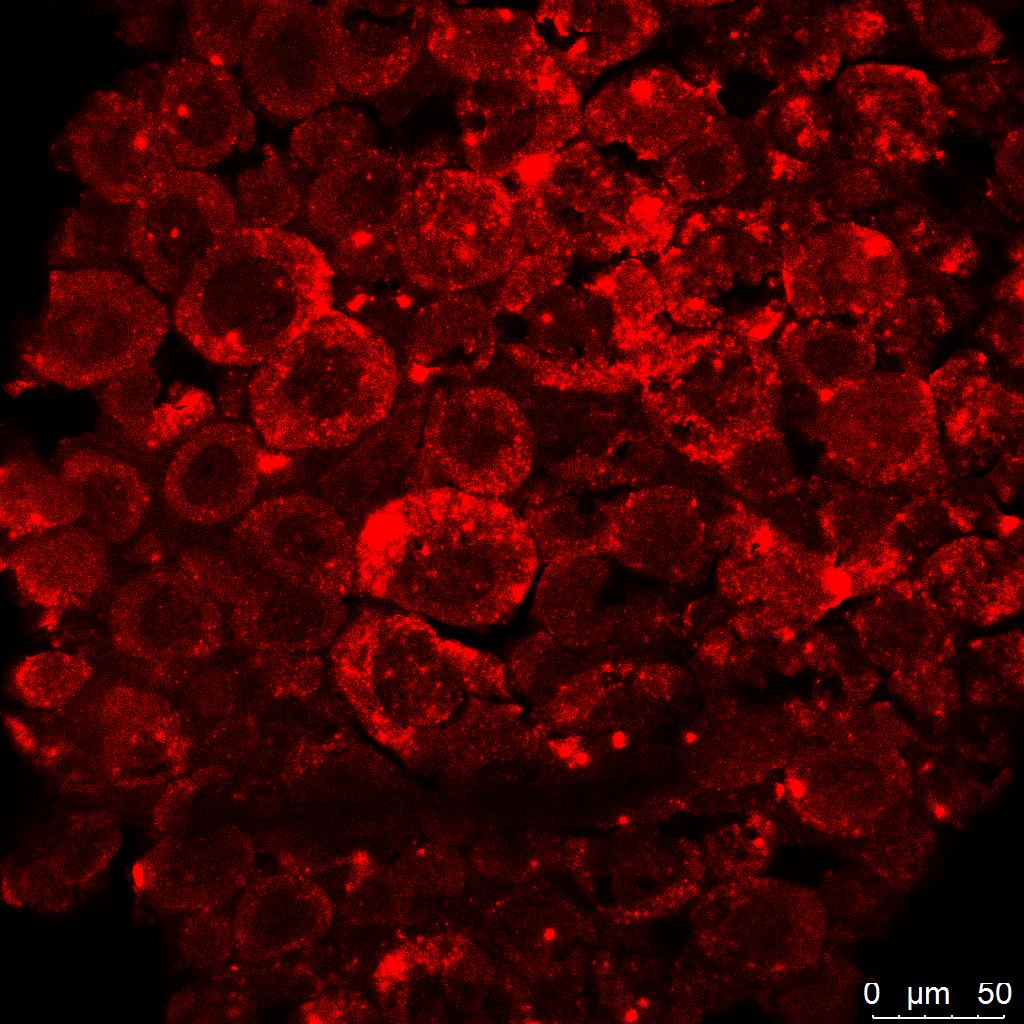

Supplement: Supplementary file 11 — Figure Source Data for Appendix Figures [file 44319_2026_775_MOESM11_ESM.zip › Source Data for Appendix Figure S1 3-7/Appendix Figure S1/Appendix Figure S1E/stn1 45dpf.tif]

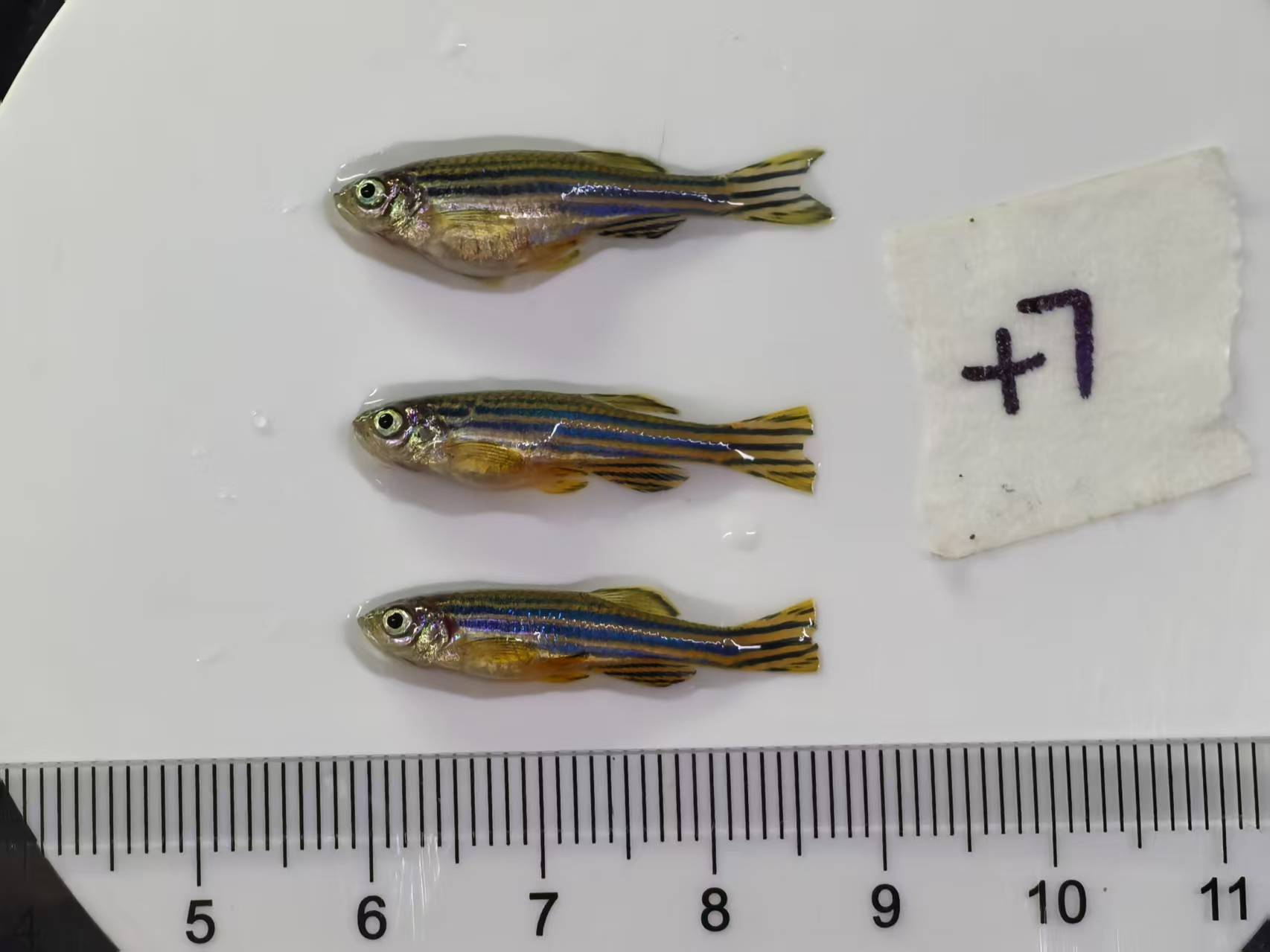

Supplement: Supplementary file 11 — Figure Source Data for Appendix Figures [file 44319_2026_775_MOESM11_ESM.zip › Source Data for Appendix Figure S1 3-7/Appendix Figure S3/Appendix Figure S3A/+7 line 3 mpf.jpg]

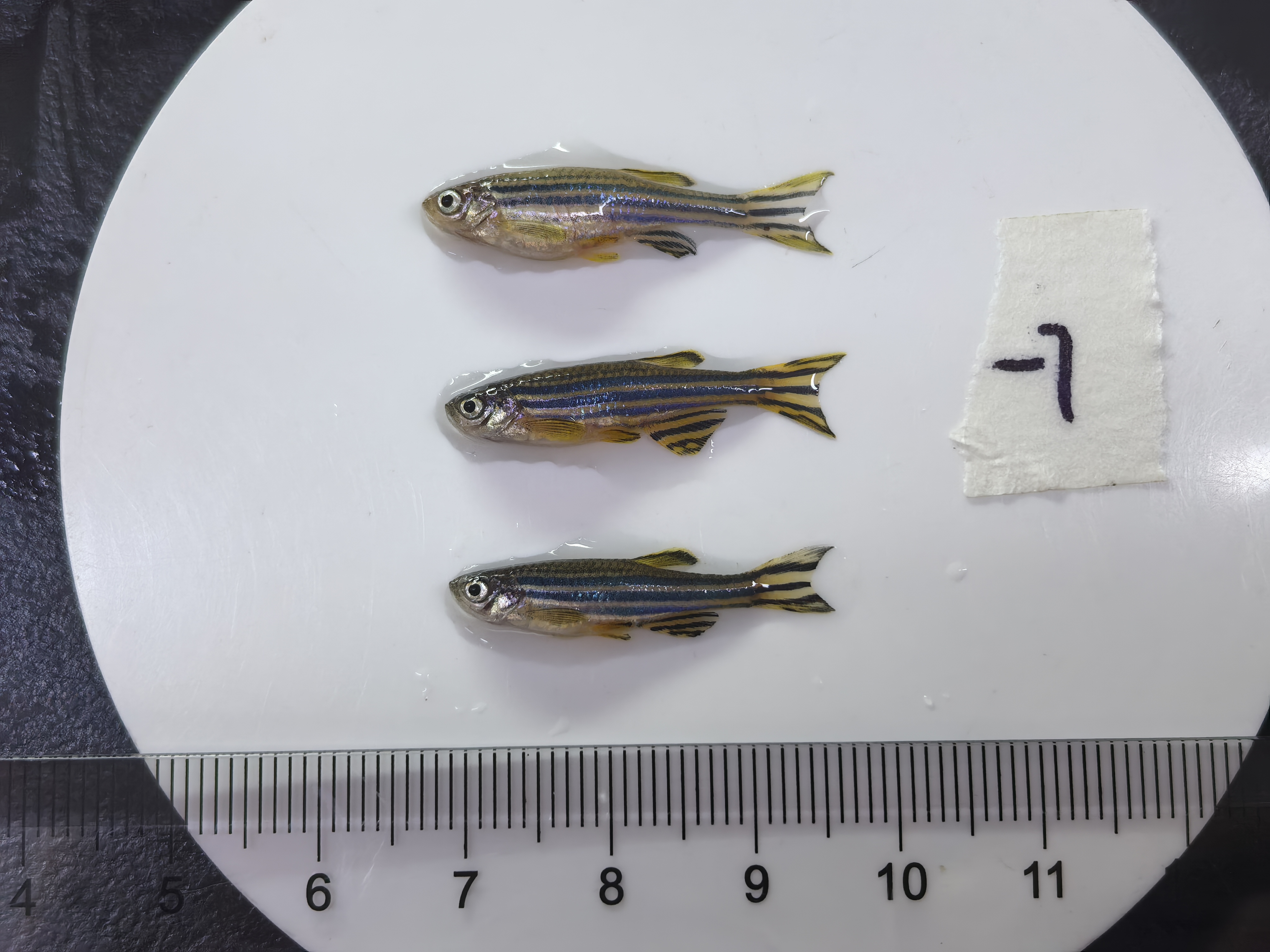

Supplement: Supplementary file 11 — Figure Source Data for Appendix Figures [file 44319_2026_775_MOESM11_ESM.zip › Source Data for Appendix Figure S1 3-7/Appendix Figure S3/Appendix Figure S3A/Δ7 line 3 mpf.jpg]

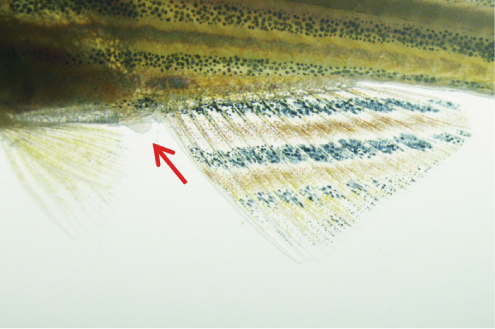

Supplement: Supplementary file 11 — Figure Source Data for Appendix Figures [file 44319_2026_775_MOESM11_ESM.zip › Source Data for Appendix Figure S1 3-7/Appendix Figure S3/Appendix Figure S3B/+7 line hom.tif]

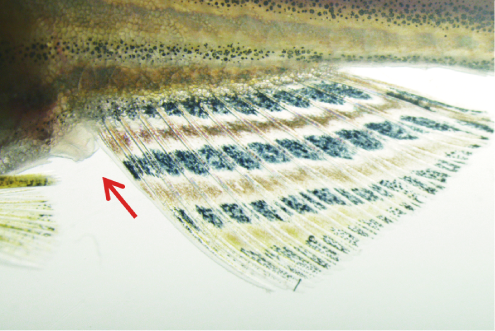

Supplement: Supplementary file 11 — Figure Source Data for Appendix Figures [file 44319_2026_775_MOESM11_ESM.zip › Source Data for Appendix Figure S1 3-7/Appendix Figure S3/Appendix Figure S3B/WT female.tif]

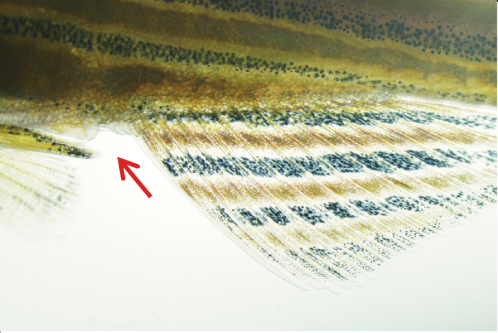

Supplement: Supplementary file 11 — Figure Source Data for Appendix Figures [file 44319_2026_775_MOESM11_ESM.zip › Source Data for Appendix Figure S1 3-7/Appendix Figure S3/Appendix Figure S3B/WT male.tif]

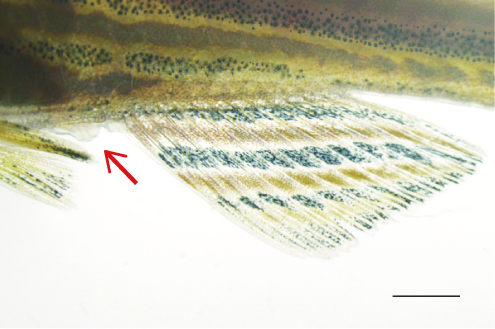

Supplement: Supplementary file 11 — Figure Source Data for Appendix Figures [file 44319_2026_775_MOESM11_ESM.zip › Source Data for Appendix Figure S1 3-7/Appendix Figure S3/Appendix Figure S3B/Δ7 line hom.tif]

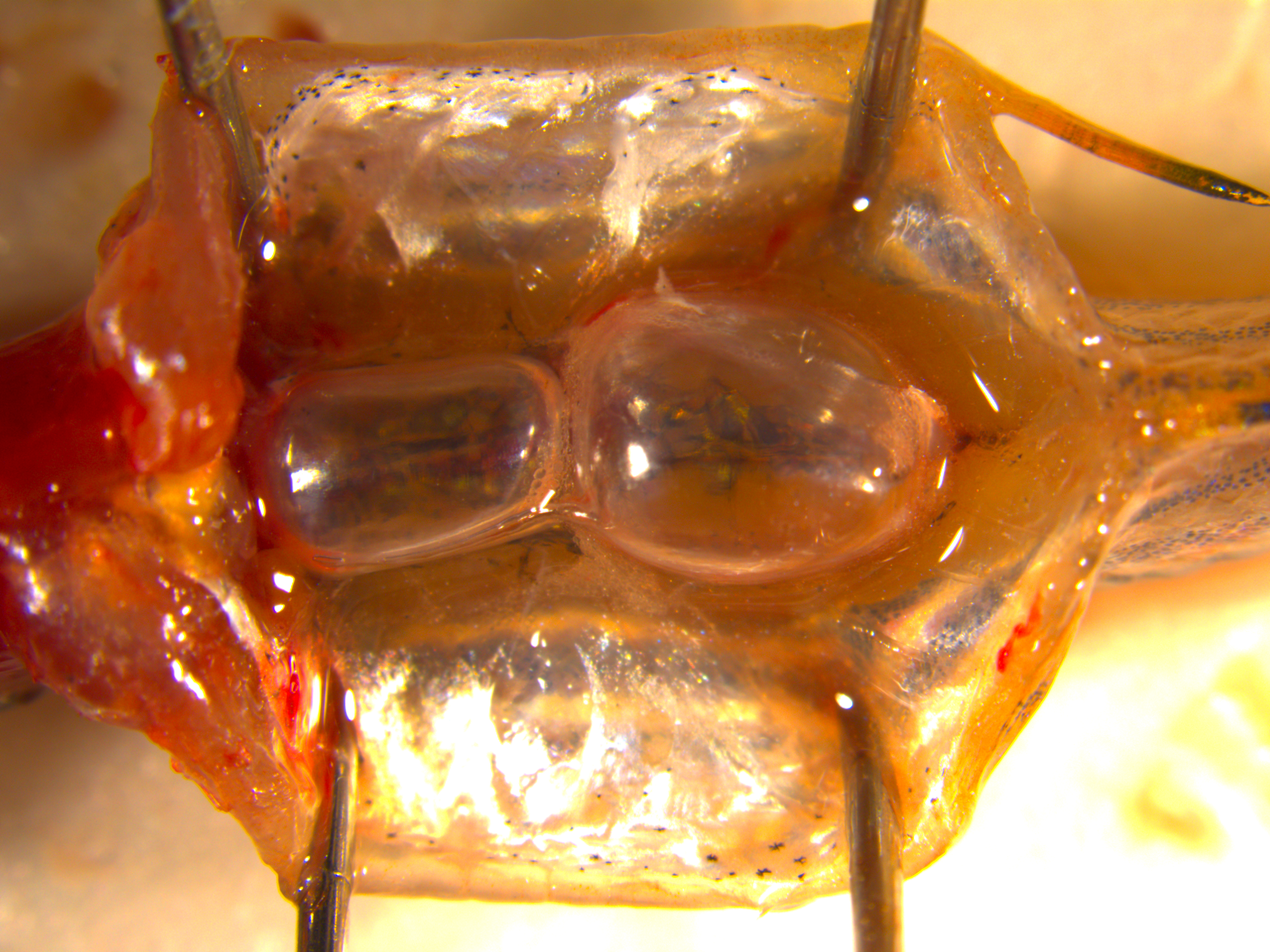

Supplement: Supplementary file 11 — Figure Source Data for Appendix Figures [file 44319_2026_775_MOESM11_ESM.zip › Source Data for Appendix Figure S1 3-7/Appendix Figure S3/Appendix Figure S3E/+7 line hom.tif]

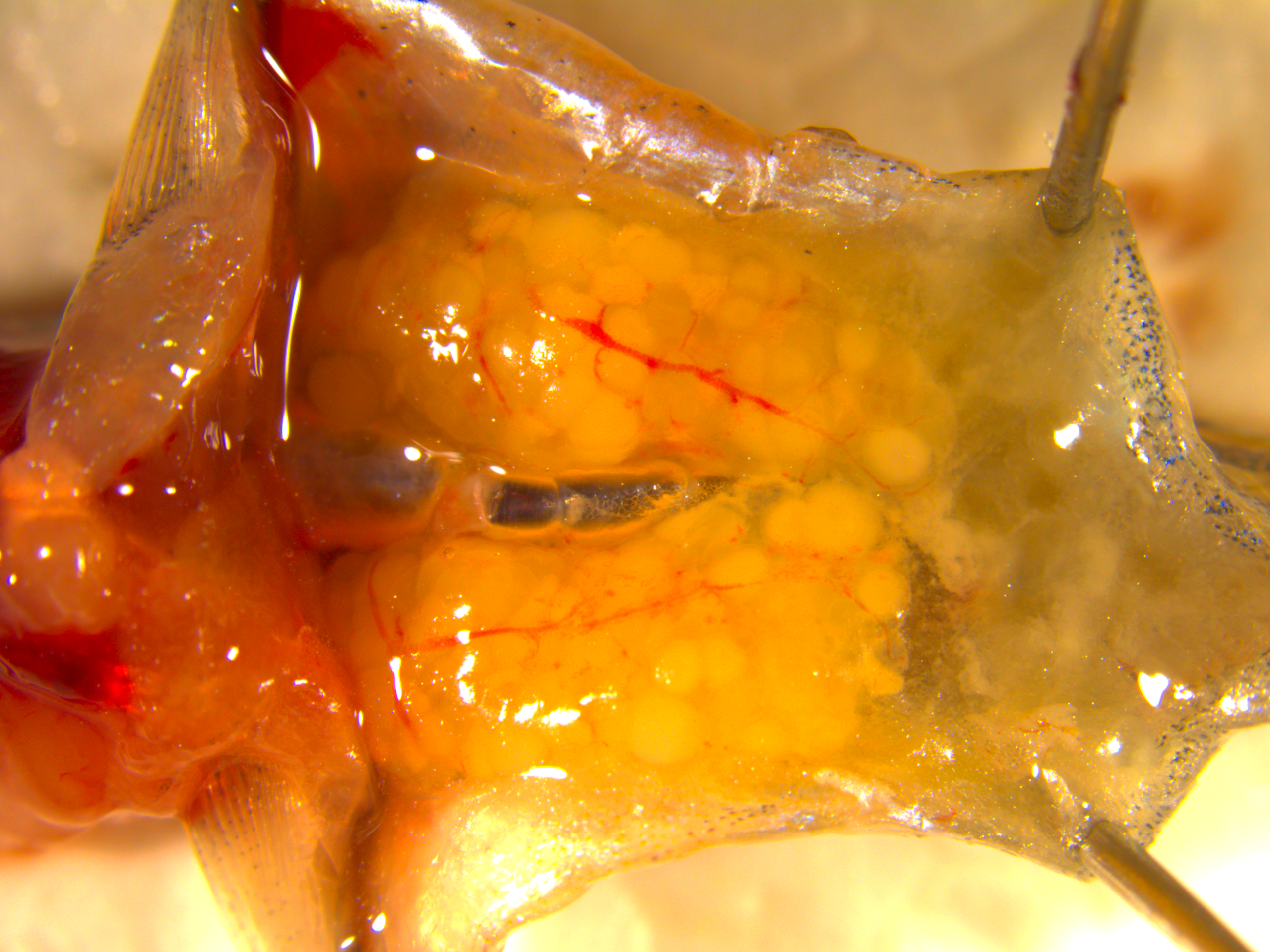

Supplement: Supplementary file 11 — Figure Source Data for Appendix Figures [file 44319_2026_775_MOESM11_ESM.zip › Source Data for Appendix Figure S1 3-7/Appendix Figure S3/Appendix Figure S3E/+7 line WT female.tif]

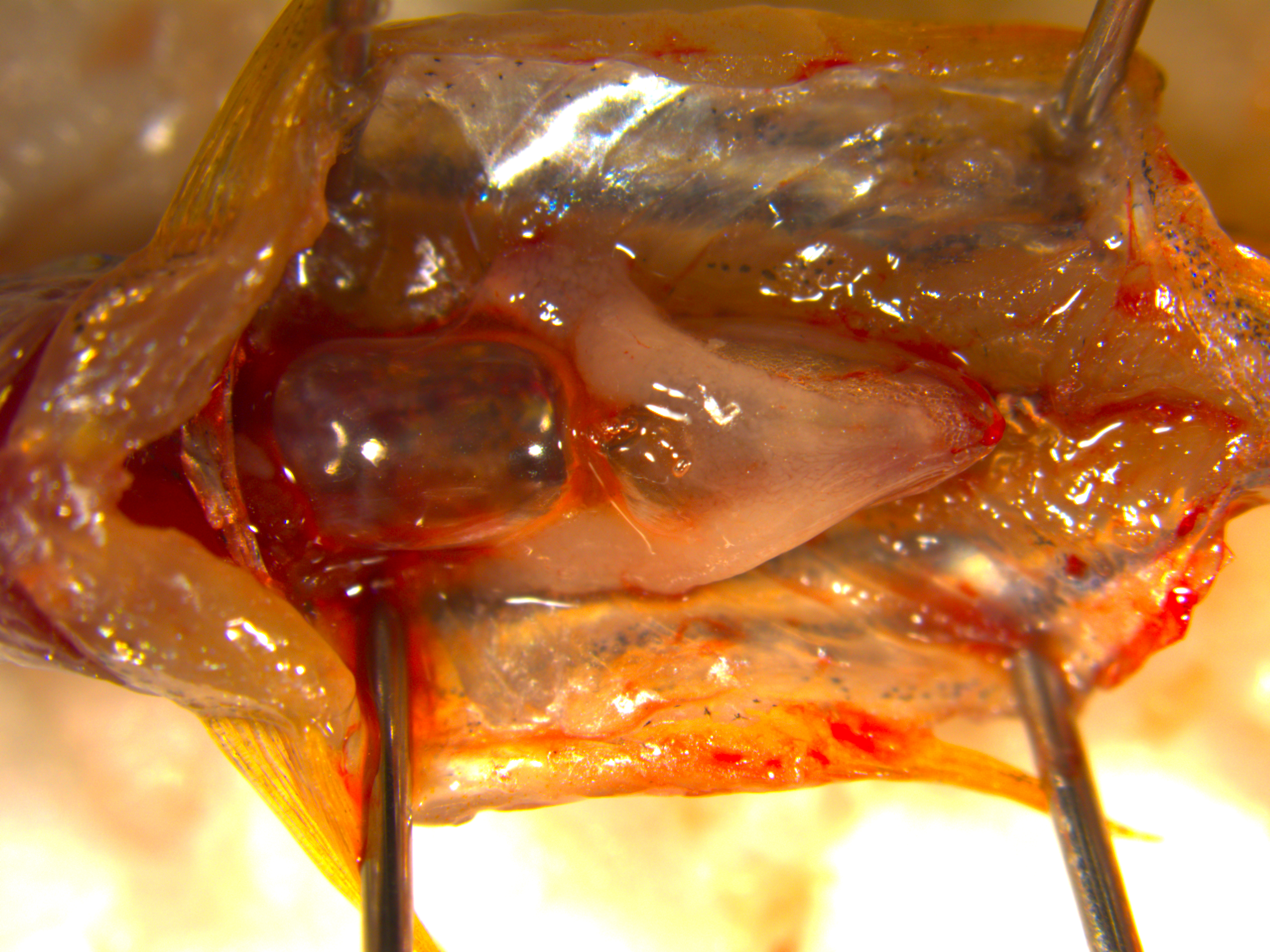

Supplement: Supplementary file 11 — Figure Source Data for Appendix Figures [file 44319_2026_775_MOESM11_ESM.zip › Source Data for Appendix Figure S1 3-7/Appendix Figure S3/Appendix Figure S3E/+7 line WT male.tif]

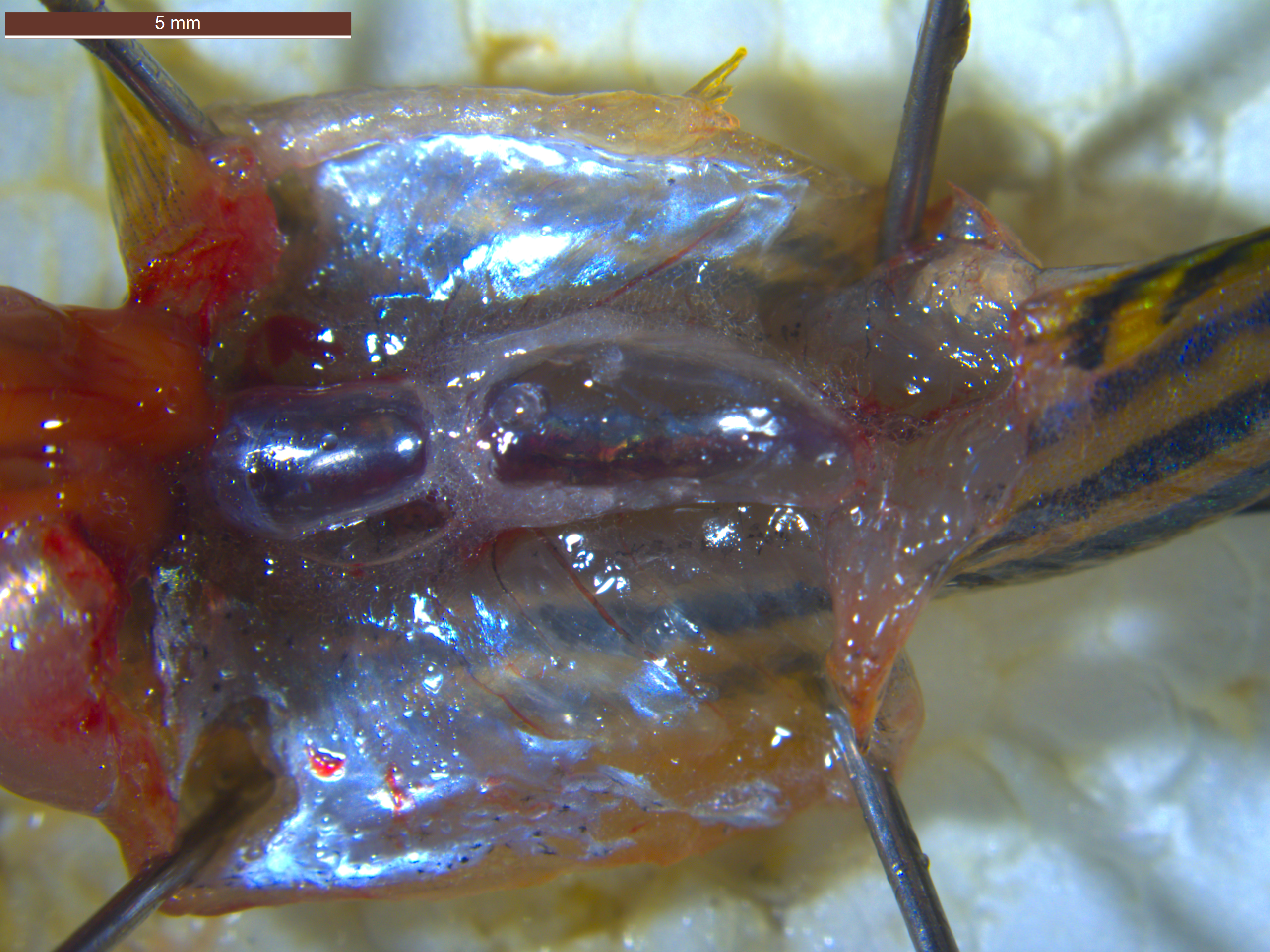

Supplement: Supplementary file 11 — Figure Source Data for Appendix Figures [file 44319_2026_775_MOESM11_ESM.zip › Source Data for Appendix Figure S1 3-7/Appendix Figure S3/Appendix Figure S3E/Δ7line hom.tif]

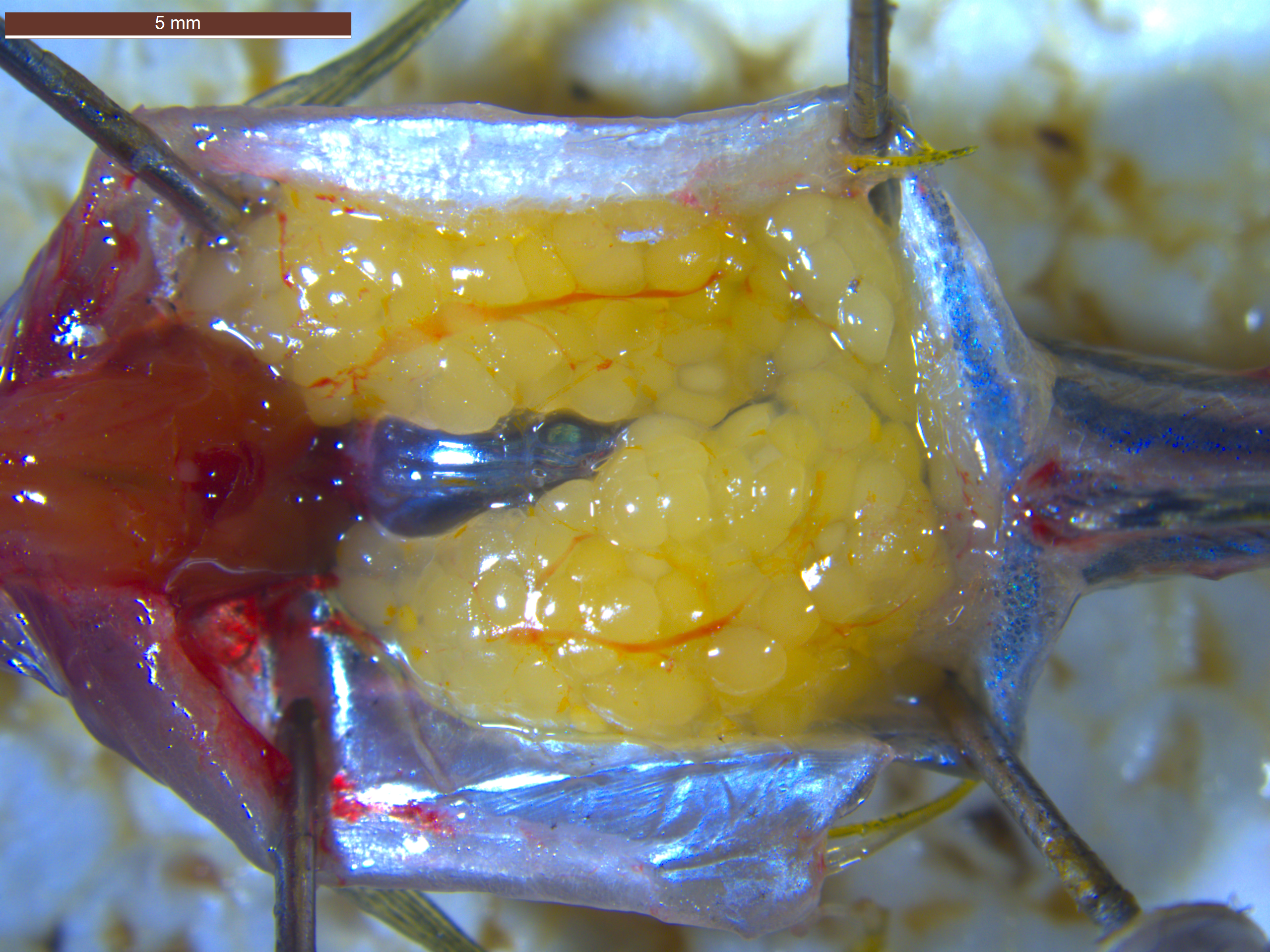

Supplement: Supplementary file 11 — Figure Source Data for Appendix Figures [file 44319_2026_775_MOESM11_ESM.zip › Source Data for Appendix Figure S1 3-7/Appendix Figure S3/Appendix Figure S3E/Δ7line WT female.tif]

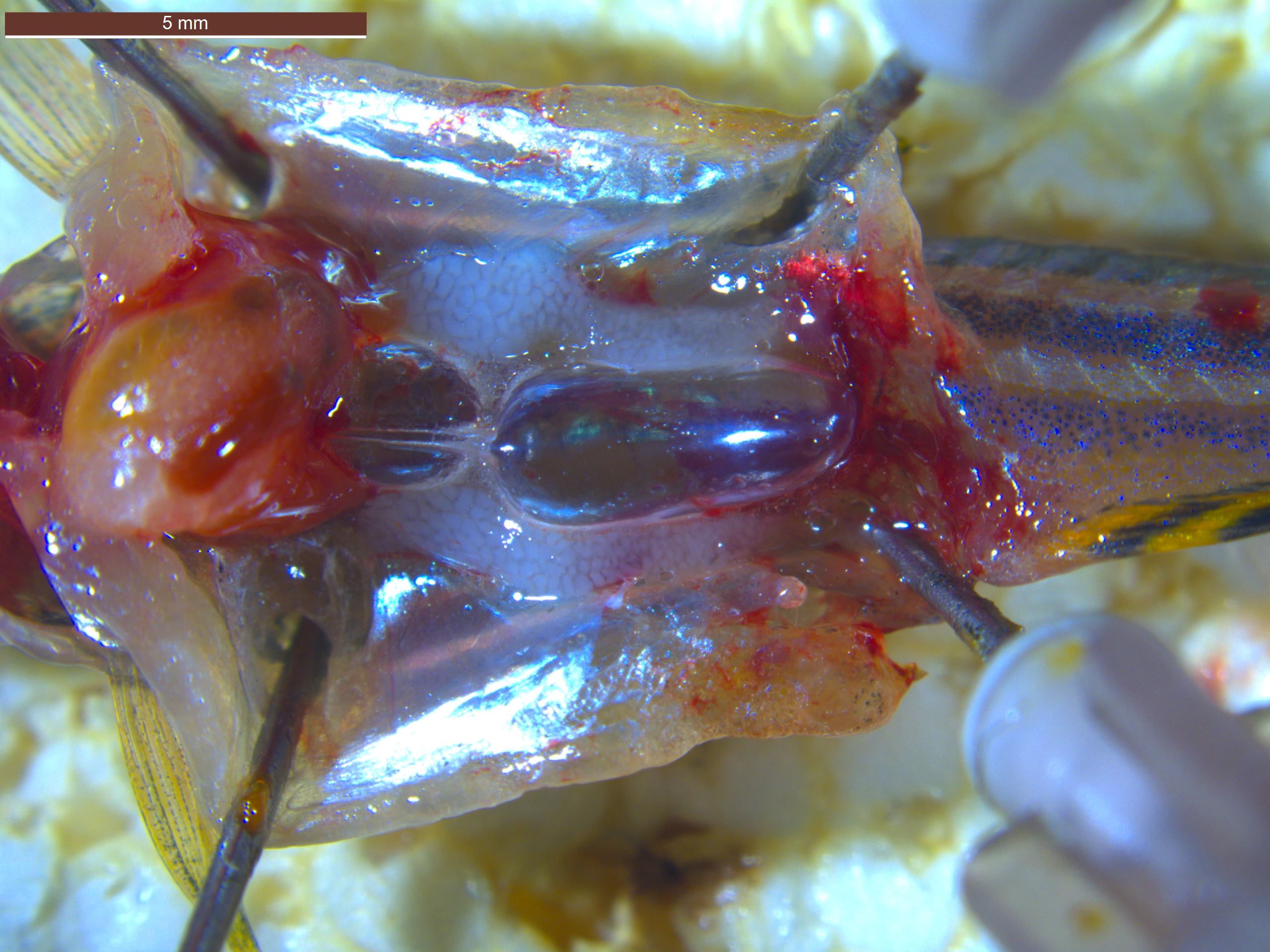

Supplement: Supplementary file 11 — Figure Source Data for Appendix Figures [file 44319_2026_775_MOESM11_ESM.zip › Source Data for Appendix Figure S1 3-7/Appendix Figure S3/Appendix Figure S3E/Δ7line WT male.tif]

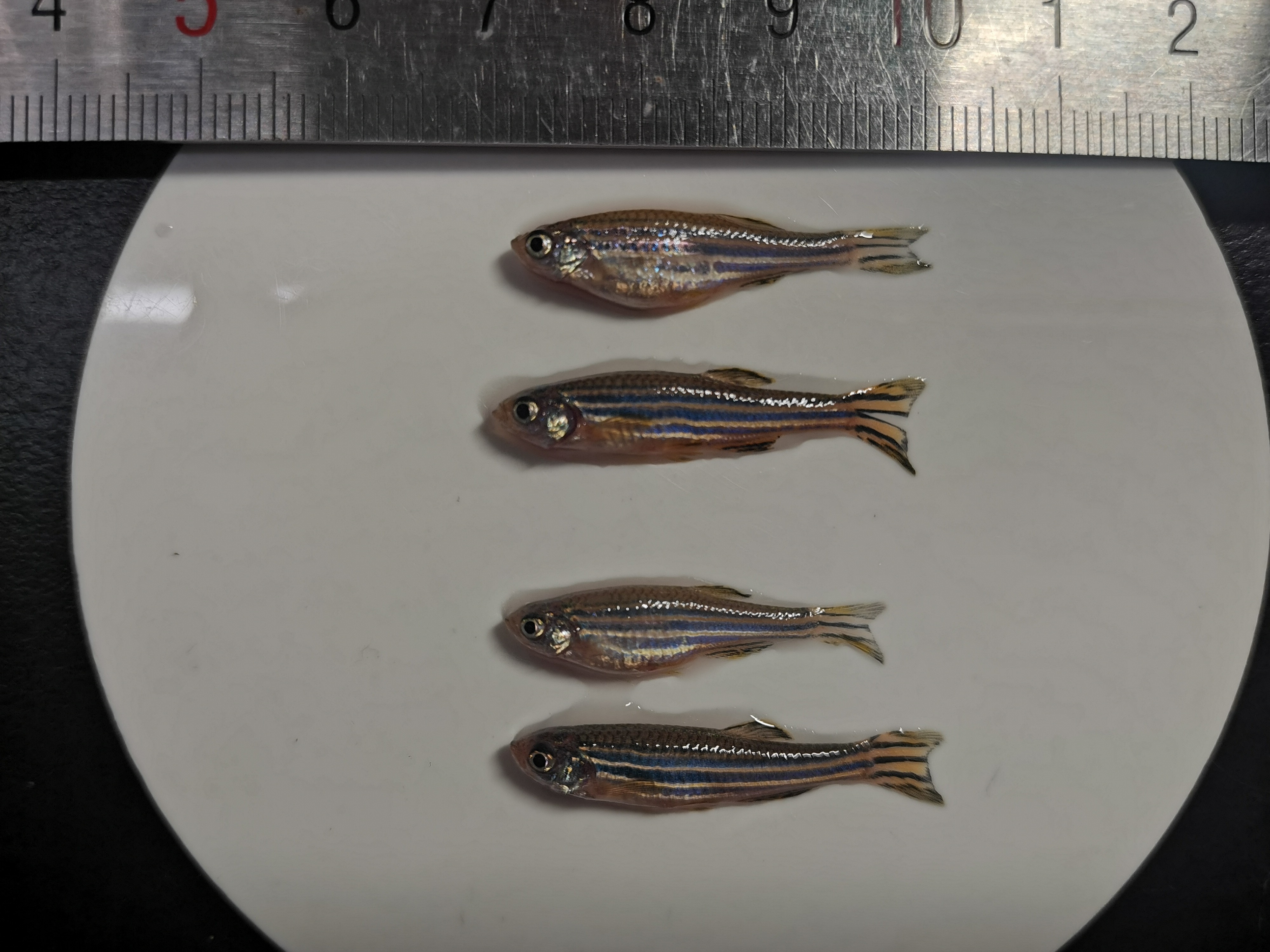

Supplement: Supplementary file 11 — Figure Source Data for Appendix Figures [file 44319_2026_775_MOESM11_ESM.zip › Source Data for Appendix Figure S1 3-7/Appendix Figure S3/Appendix Figure S3G/Figure S3G bright field .jpg]

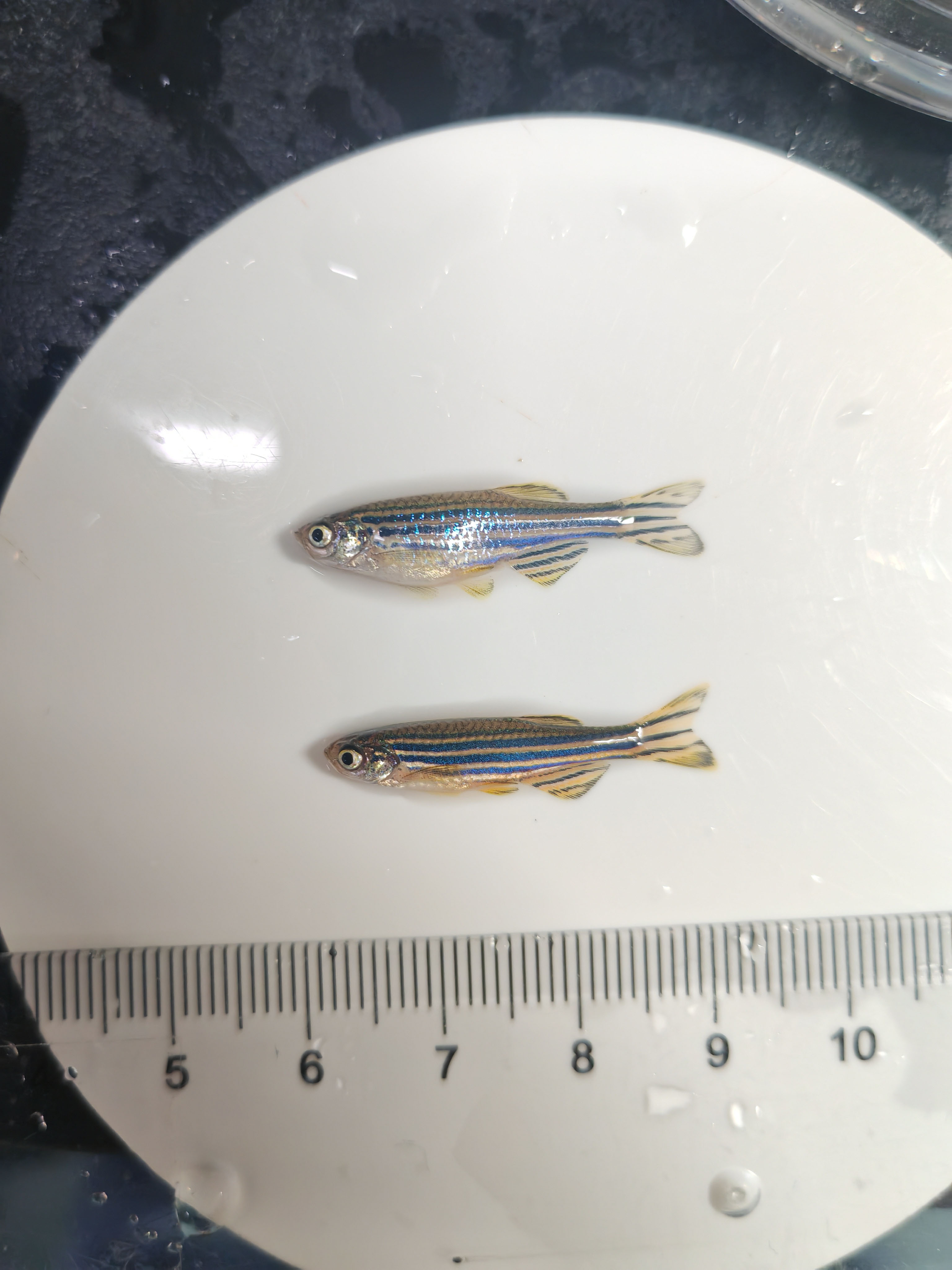

Supplement: Supplementary file 11 — Figure Source Data for Appendix Figures [file 44319_2026_775_MOESM11_ESM.zip › Source Data for Appendix Figure S1 3-7/Appendix Figure S3/Appendix Figure S3J/het piwil1stn1-Flag .jpg]

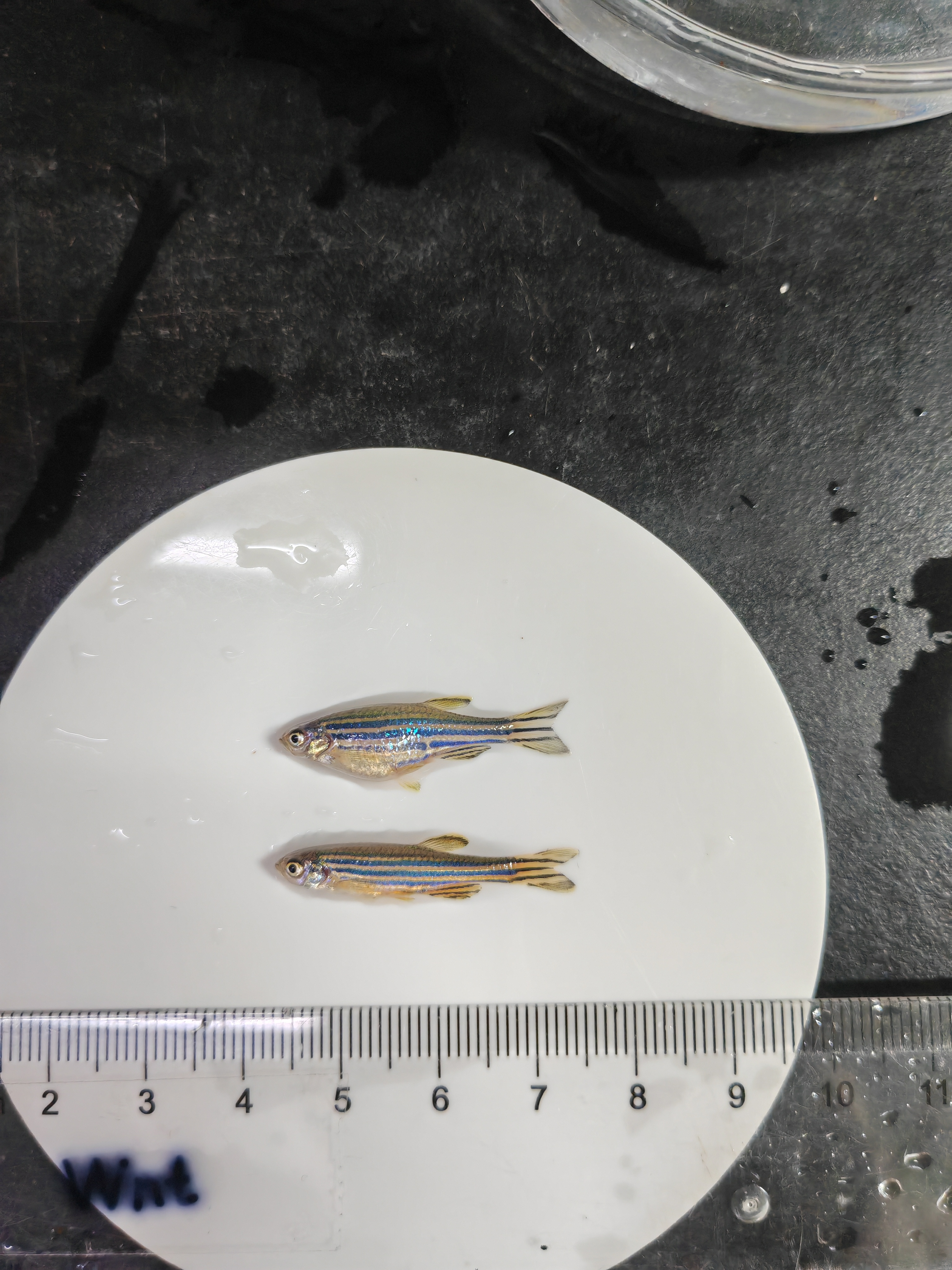

Supplement: Supplementary file 11 — Figure Source Data for Appendix Figures [file 44319_2026_775_MOESM11_ESM.zip › Source Data for Appendix Figure S1 3-7/Appendix Figure S3/Appendix Figure S3J/hom piwil1stn1-Flag .jpg]

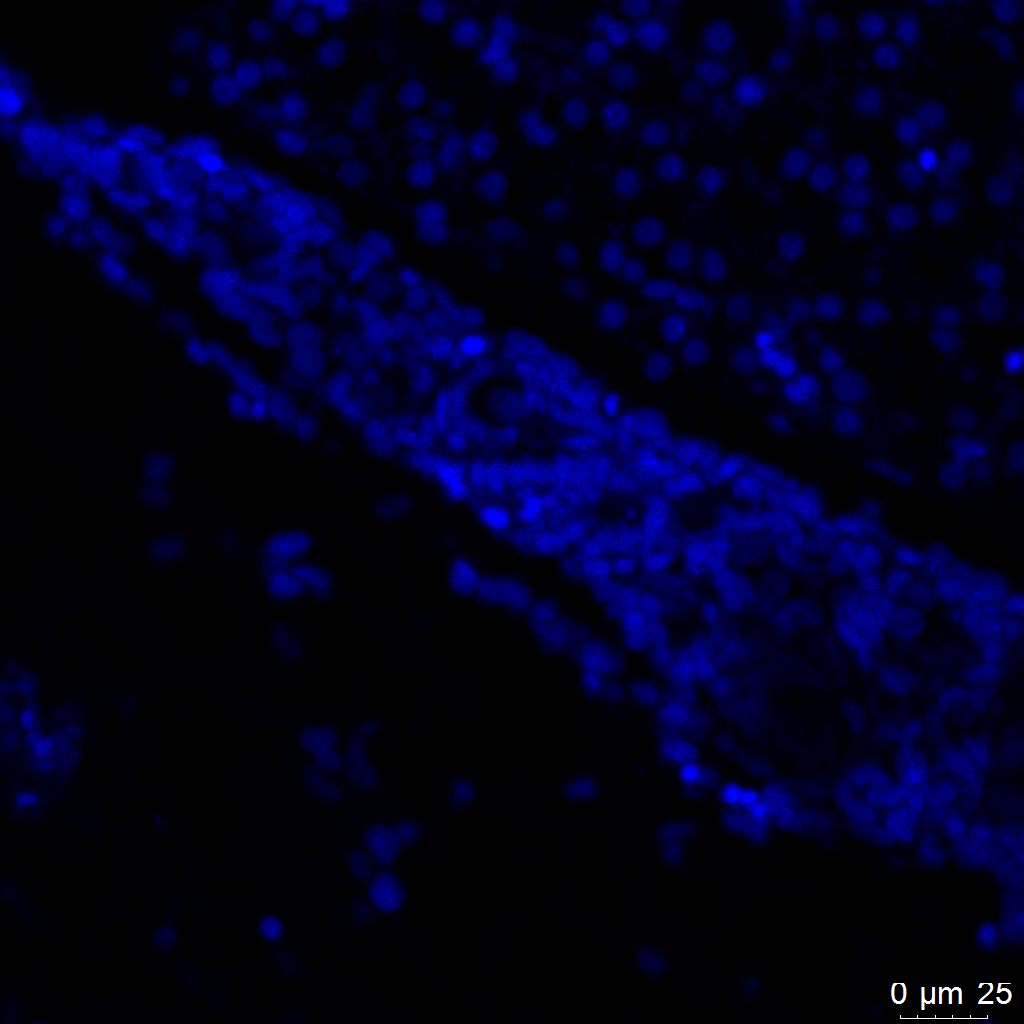

Supplement: Supplementary file 11 — Figure Source Data for Appendix Figures [file 44319_2026_775_MOESM11_ESM.zip › Source Data for Appendix Figure S1 3-7/Appendix Figure S4/Appendix Figure S4F/ddx4/DAPI hom.tif]

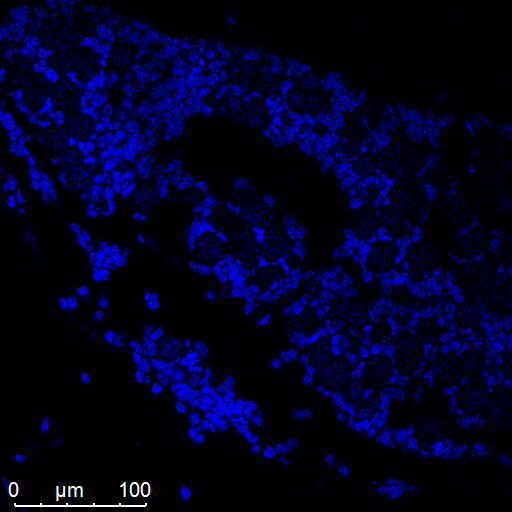

Supplement: Supplementary file 11 — Figure Source Data for Appendix Figures [file 44319_2026_775_MOESM11_ESM.zip › Source Data for Appendix Figure S1 3-7/Appendix Figure S4/Appendix Figure S4F/ddx4/DAPI WT.tif]

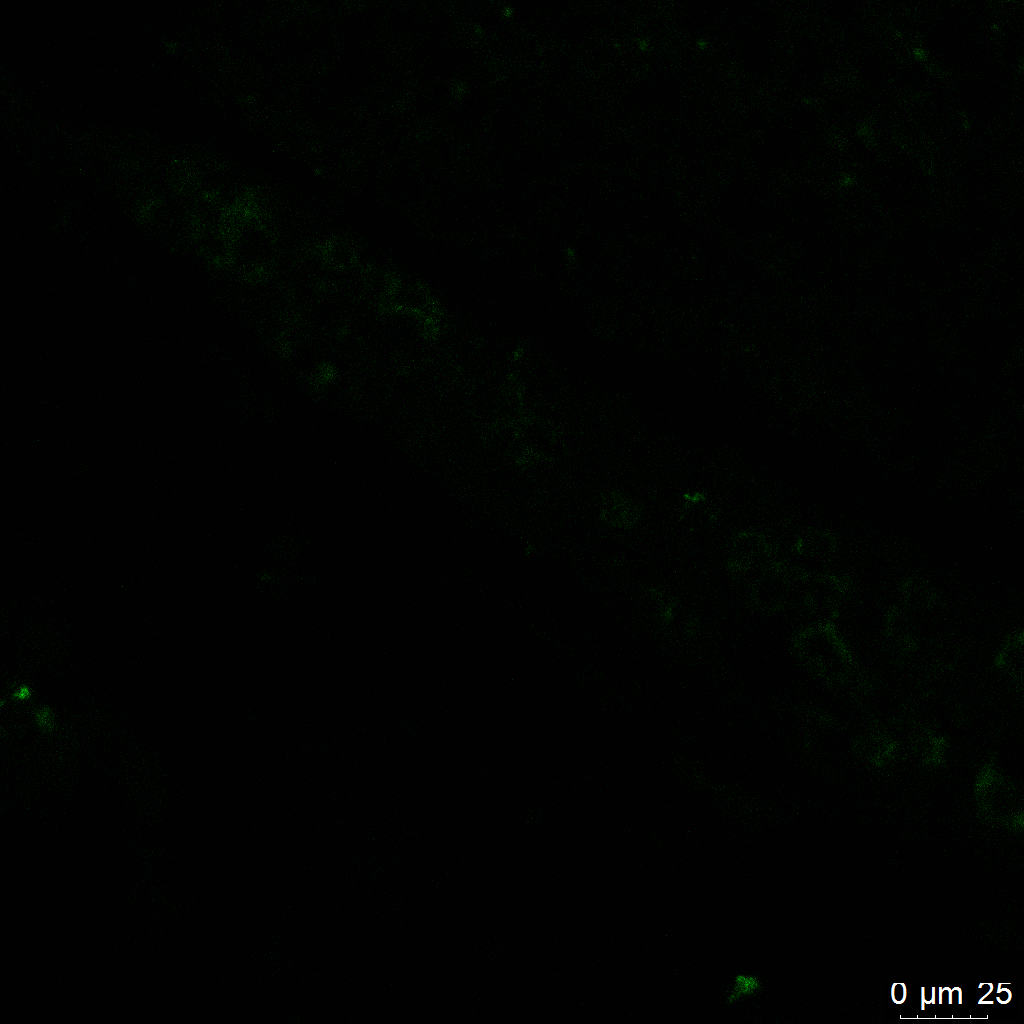

Supplement: Supplementary file 11 — Figure Source Data for Appendix Figures [file 44319_2026_775_MOESM11_ESM.zip › Source Data for Appendix Figure S1 3-7/Appendix Figure S4/Appendix Figure S4F/ddx4/ddx4 hom.tif]

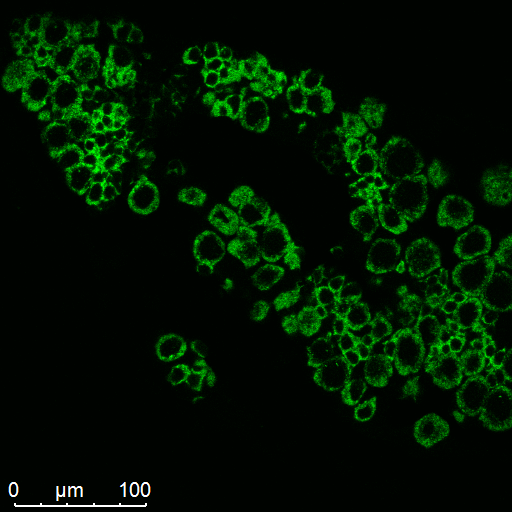

Supplement: Supplementary file 11 — Figure Source Data for Appendix Figures [file 44319_2026_775_MOESM11_ESM.zip › Source Data for Appendix Figure S1 3-7/Appendix Figure S4/Appendix Figure S4F/ddx4/ddx4 WT.tif]

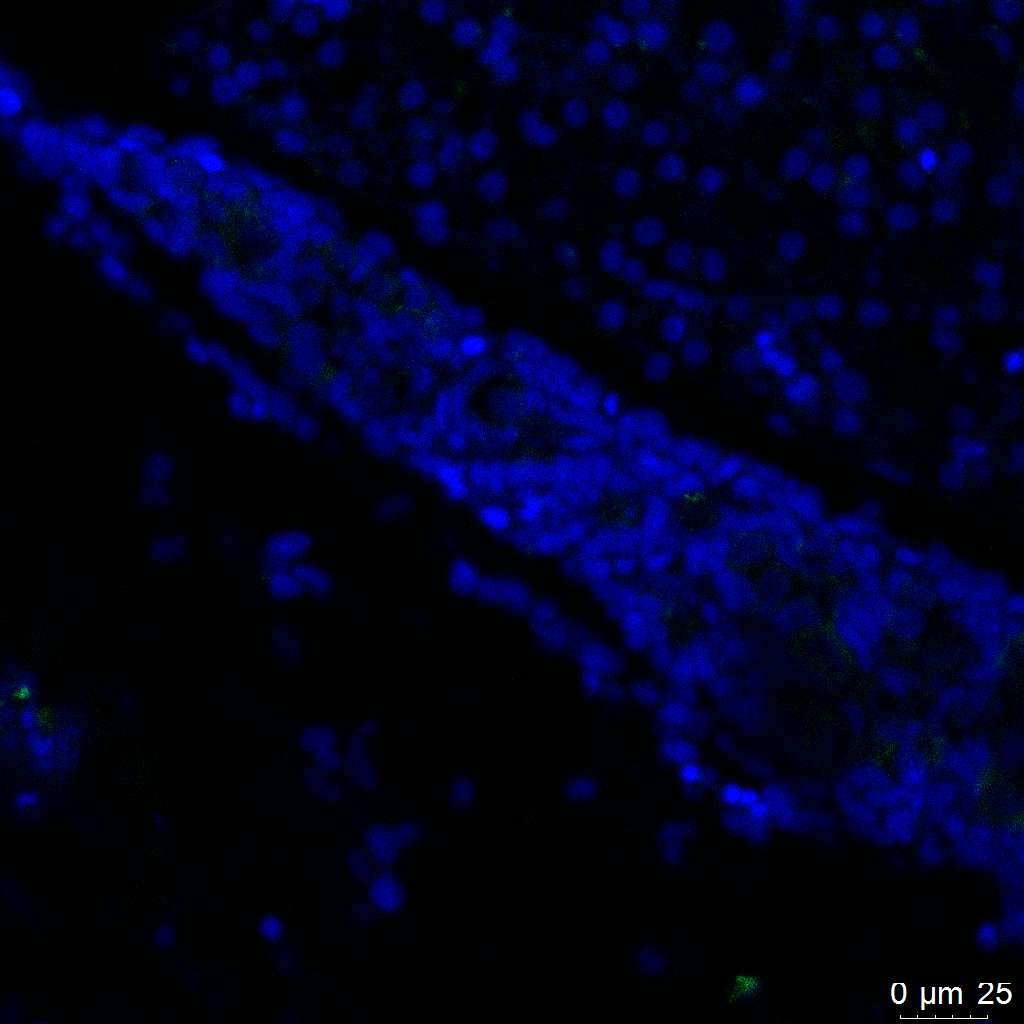

Supplement: Supplementary file 11 — Figure Source Data for Appendix Figures [file 44319_2026_775_MOESM11_ESM.zip › Source Data for Appendix Figure S1 3-7/Appendix Figure S4/Appendix Figure S4F/ddx4/Merge hom.tif]

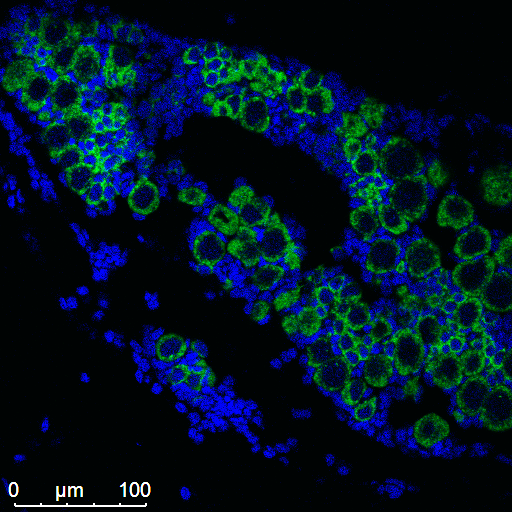

Supplement: Supplementary file 11 — Figure Source Data for Appendix Figures [file 44319_2026_775_MOESM11_ESM.zip › Source Data for Appendix Figure S1 3-7/Appendix Figure S4/Appendix Figure S4F/ddx4/Merge WT.tif]

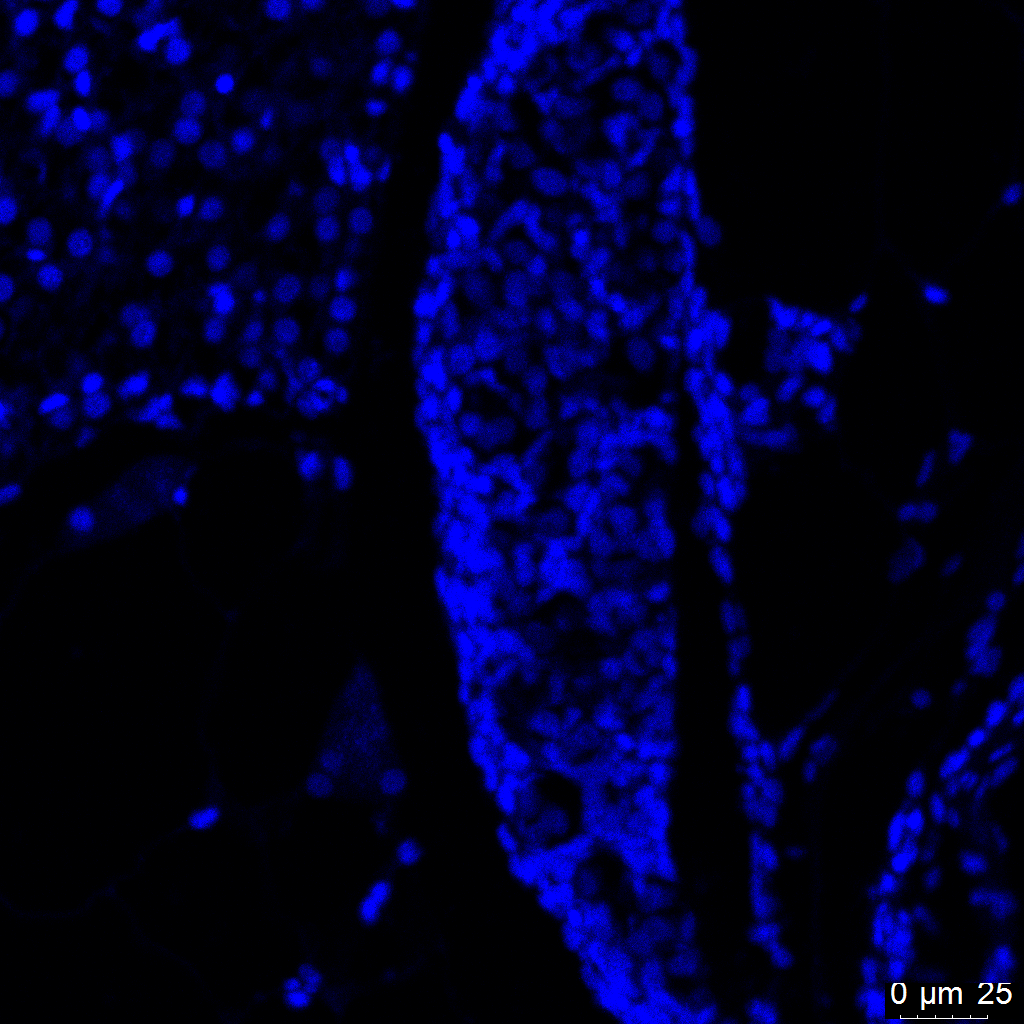

Supplement: Supplementary file 11 — Figure Source Data for Appendix Figures [file 44319_2026_775_MOESM11_ESM.zip › Source Data for Appendix Figure S1 3-7/Appendix Figure S4/Appendix Figure S4F/dnd1/DAPI hom.tif]

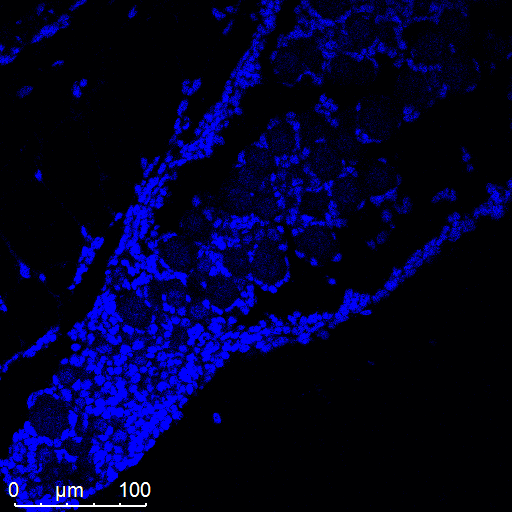

Supplement: Supplementary file 11 — Figure Source Data for Appendix Figures [file 44319_2026_775_MOESM11_ESM.zip › Source Data for Appendix Figure S1 3-7/Appendix Figure S4/Appendix Figure S4F/dnd1/DAPI WT.tif]

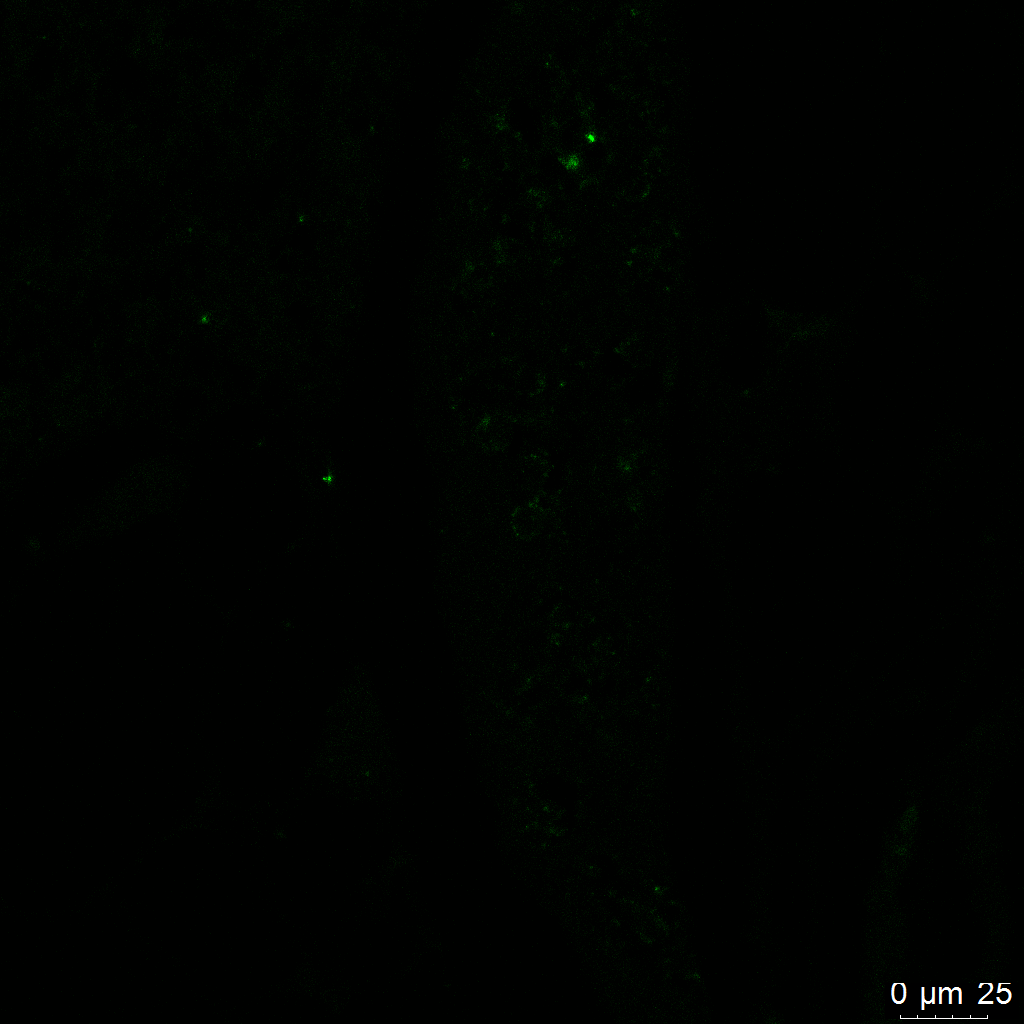

Supplement: Supplementary file 11 — Figure Source Data for Appendix Figures [file 44319_2026_775_MOESM11_ESM.zip › Source Data for Appendix Figure S1 3-7/Appendix Figure S4/Appendix Figure S4F/dnd1/dnd1 hom.tif]

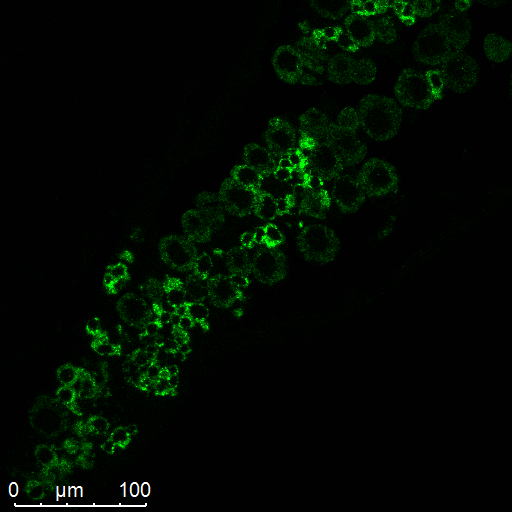

Supplement: Supplementary file 11 — Figure Source Data for Appendix Figures [file 44319_2026_775_MOESM11_ESM.zip › Source Data for Appendix Figure S1 3-7/Appendix Figure S4/Appendix Figure S4F/dnd1/dnd1 WT.tif]

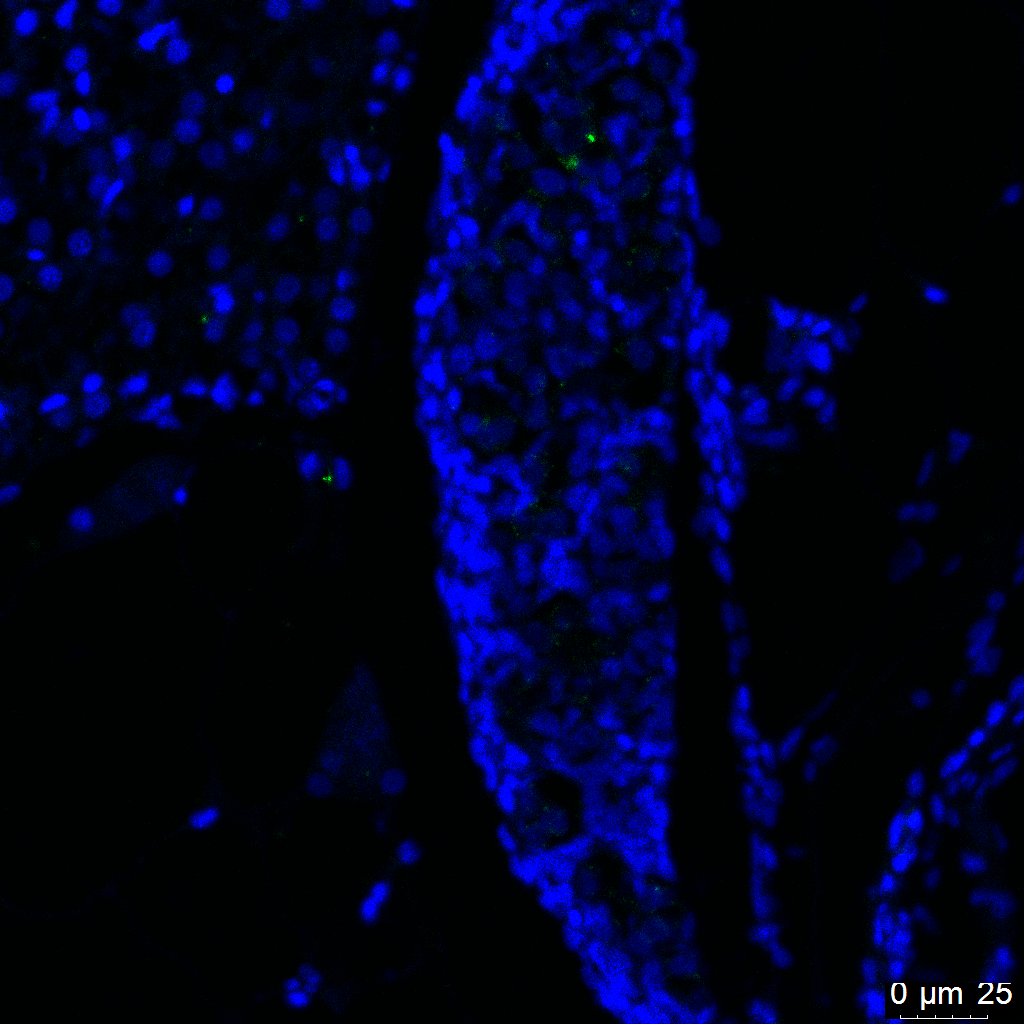

Supplement: Supplementary file 11 — Figure Source Data for Appendix Figures [file 44319_2026_775_MOESM11_ESM.zip › Source Data for Appendix Figure S1 3-7/Appendix Figure S4/Appendix Figure S4F/dnd1/Merge hom.tif]

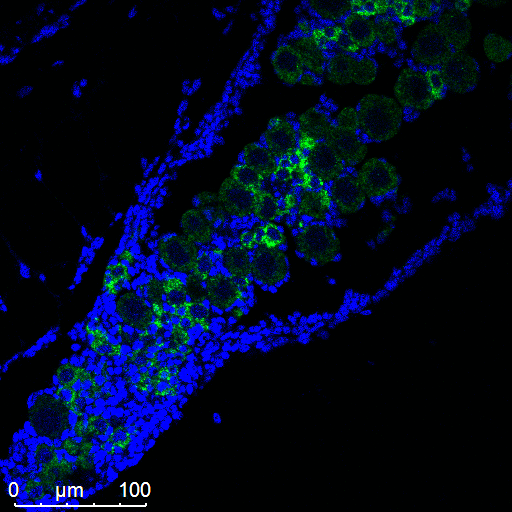

Supplement: Supplementary file 11 — Figure Source Data for Appendix Figures [file 44319_2026_775_MOESM11_ESM.zip › Source Data for Appendix Figure S1 3-7/Appendix Figure S4/Appendix Figure S4F/dnd1/Merge WT.tif]

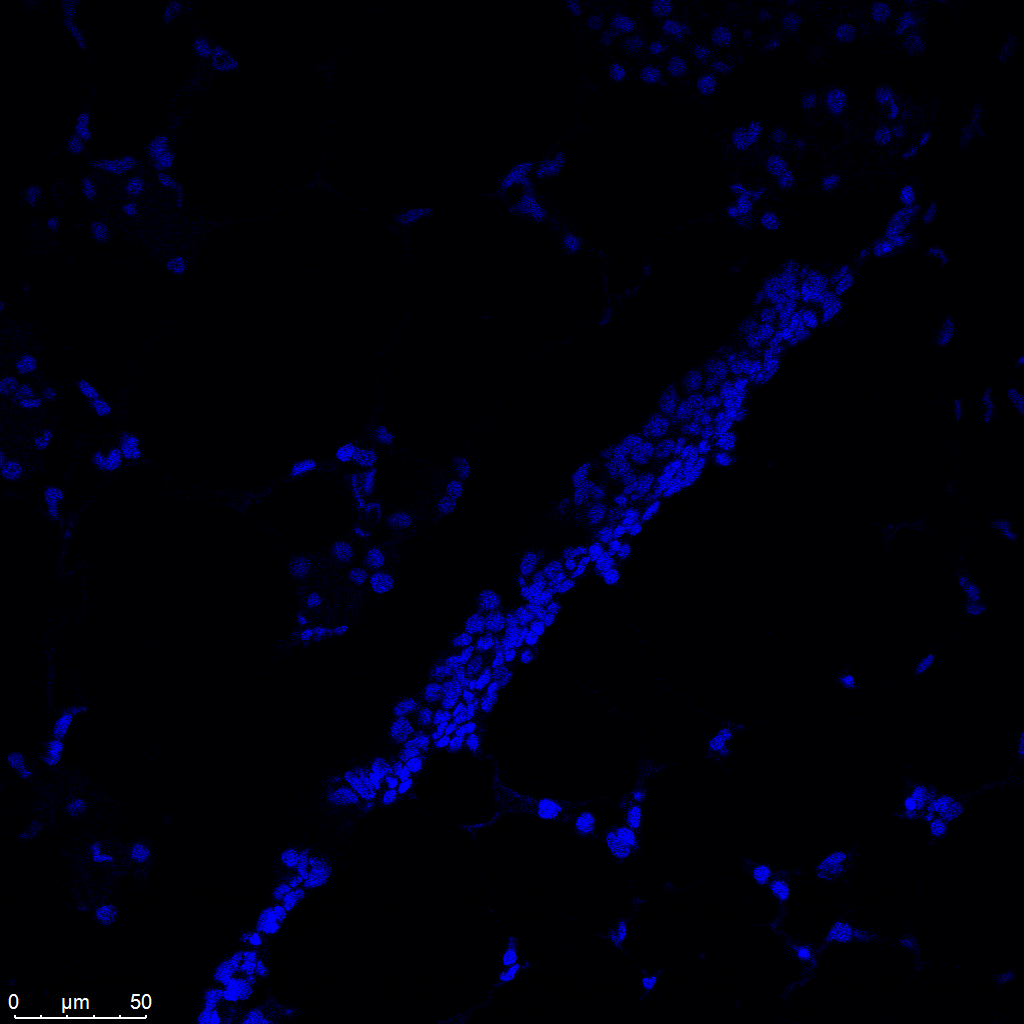

Supplement: Supplementary file 11 — Figure Source Data for Appendix Figures [file 44319_2026_775_MOESM11_ESM.zip › Source Data for Appendix Figure S1 3-7/Appendix Figure S4/Appendix Figure S4F/piwil1/DAPI hom.tif]

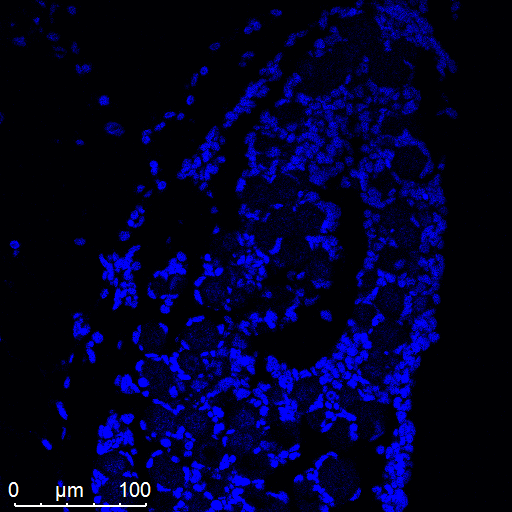

Supplement: Supplementary file 11 — Figure Source Data for Appendix Figures [file 44319_2026_775_MOESM11_ESM.zip › Source Data for Appendix Figure S1 3-7/Appendix Figure S4/Appendix Figure S4F/piwil1/DAPI WT.tif]

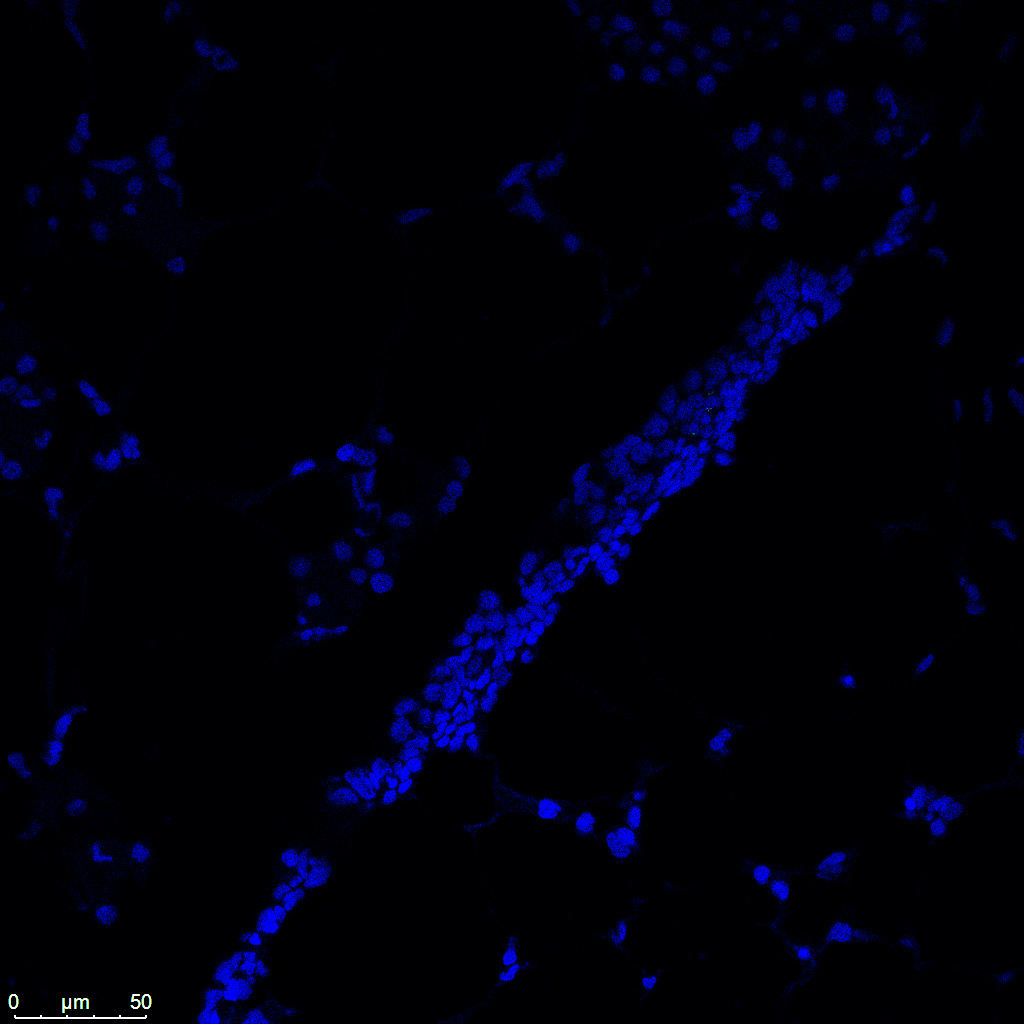

Supplement: Supplementary file 11 — Figure Source Data for Appendix Figures [file 44319_2026_775_MOESM11_ESM.zip › Source Data for Appendix Figure S1 3-7/Appendix Figure S4/Appendix Figure S4F/piwil1/Merge hom.tif]

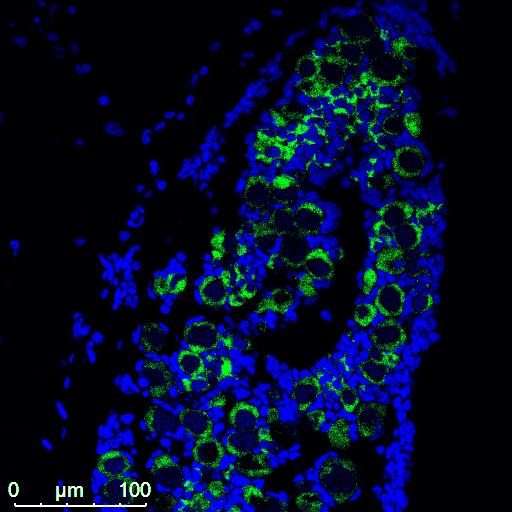

Supplement: Supplementary file 11 — Figure Source Data for Appendix Figures [file 44319_2026_775_MOESM11_ESM.zip › Source Data for Appendix Figure S1 3-7/Appendix Figure S4/Appendix Figure S4F/piwil1/Merge WT.tif]

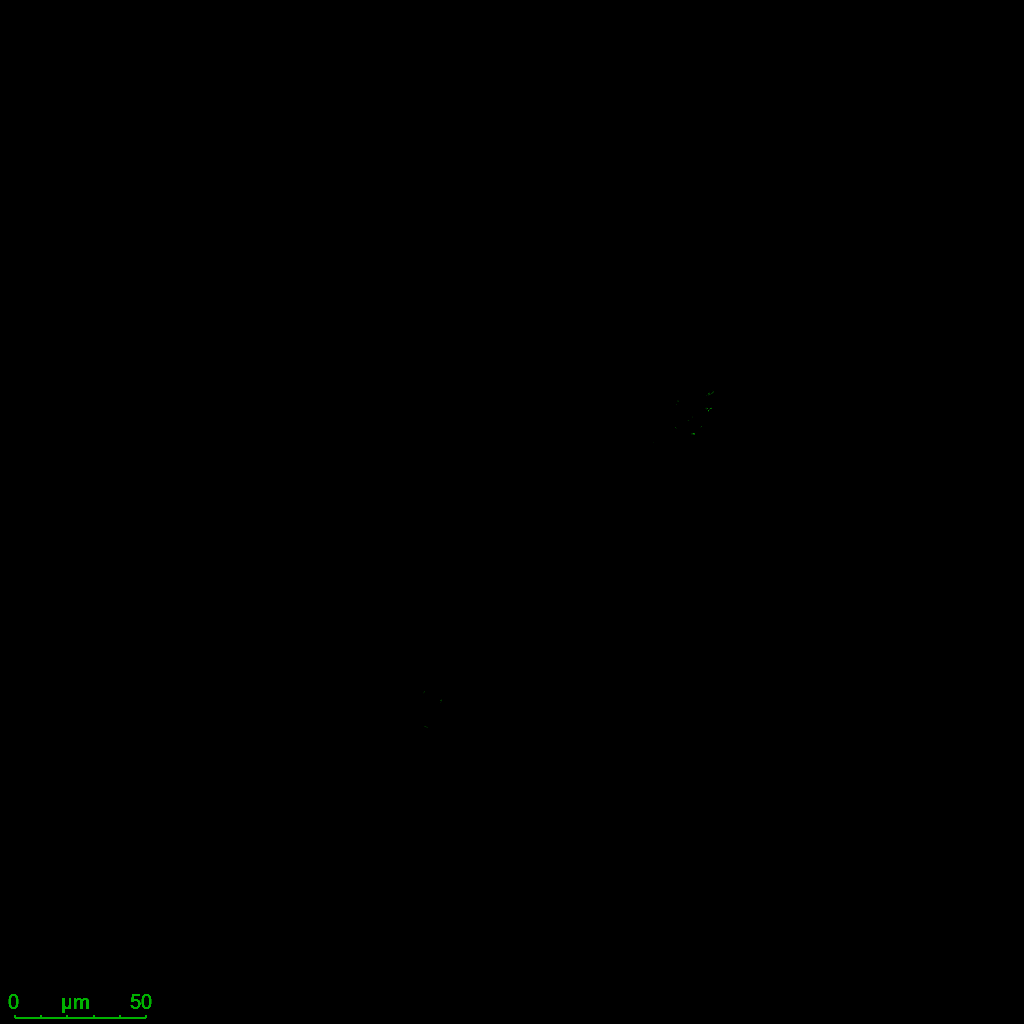

Supplement: Supplementary file 11 — Figure Source Data for Appendix Figures [file 44319_2026_775_MOESM11_ESM.zip › Source Data for Appendix Figure S1 3-7/Appendix Figure S4/Appendix Figure S4F/piwil1/piwil1 hom.tif]

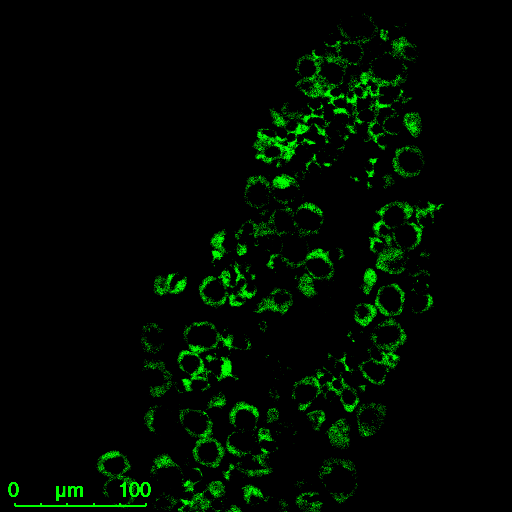

Supplement: Supplementary file 11 — Figure Source Data for Appendix Figures [file 44319_2026_775_MOESM11_ESM.zip › Source Data for Appendix Figure S1 3-7/Appendix Figure S4/Appendix Figure S4F/piwil1/piwil1 WT.tif]

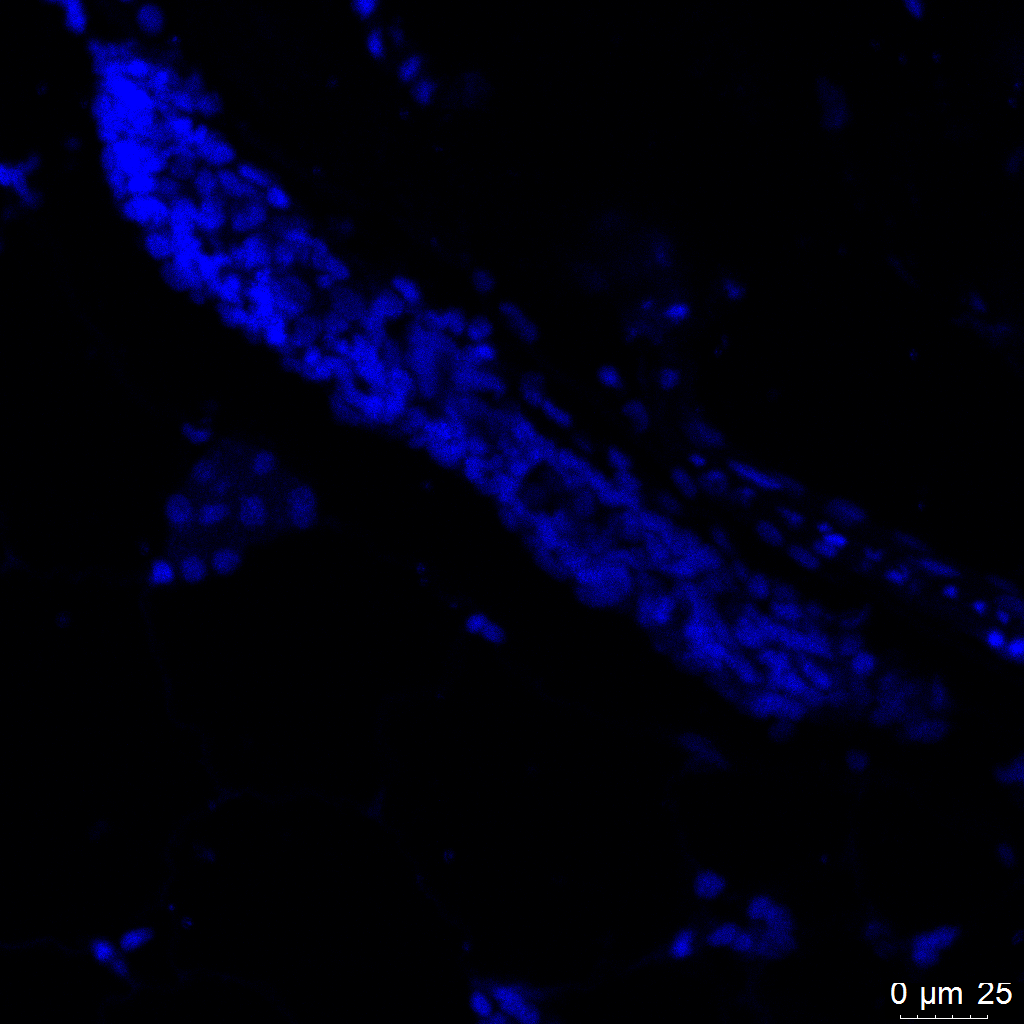

Supplement: Supplementary file 11 — Figure Source Data for Appendix Figures [file 44319_2026_775_MOESM11_ESM.zip › Source Data for Appendix Figure S1 3-7/Appendix Figure S4/Appendix Figure S4F/piwil2/DAPI hom.tif]

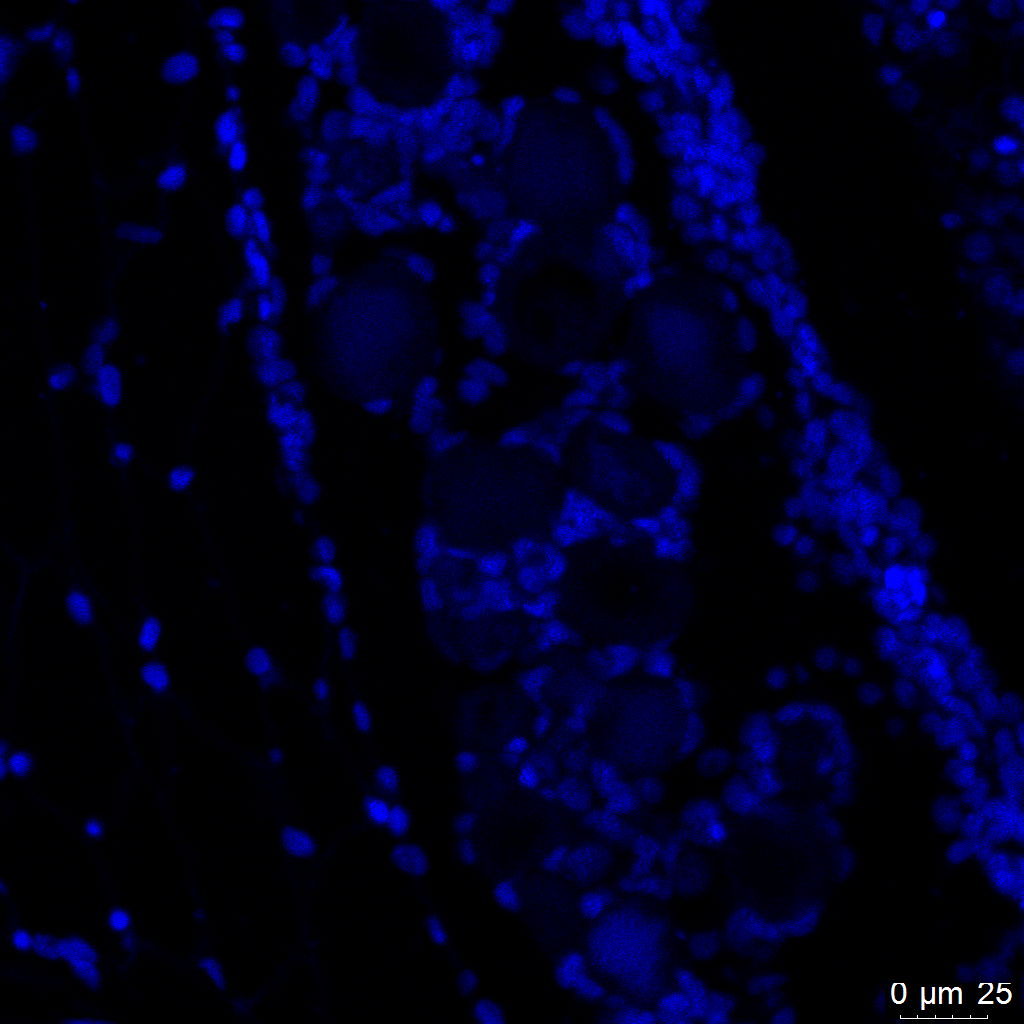

Supplement: Supplementary file 11 — Figure Source Data for Appendix Figures [file 44319_2026_775_MOESM11_ESM.zip › Source Data for Appendix Figure S1 3-7/Appendix Figure S4/Appendix Figure S4F/piwil2/DAPI WT.tif]

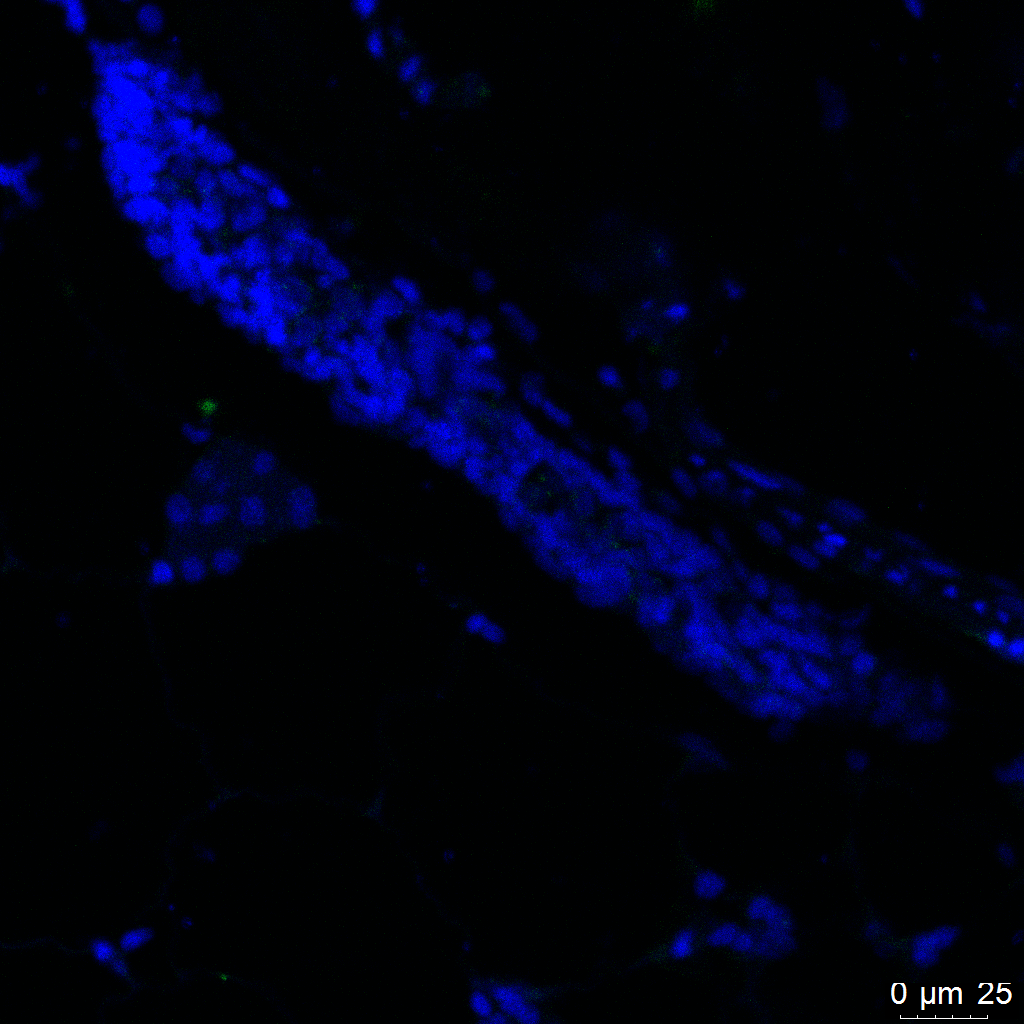

Supplement: Supplementary file 11 — Figure Source Data for Appendix Figures [file 44319_2026_775_MOESM11_ESM.zip › Source Data for Appendix Figure S1 3-7/Appendix Figure S4/Appendix Figure S4F/piwil2/Merge hom.tif]

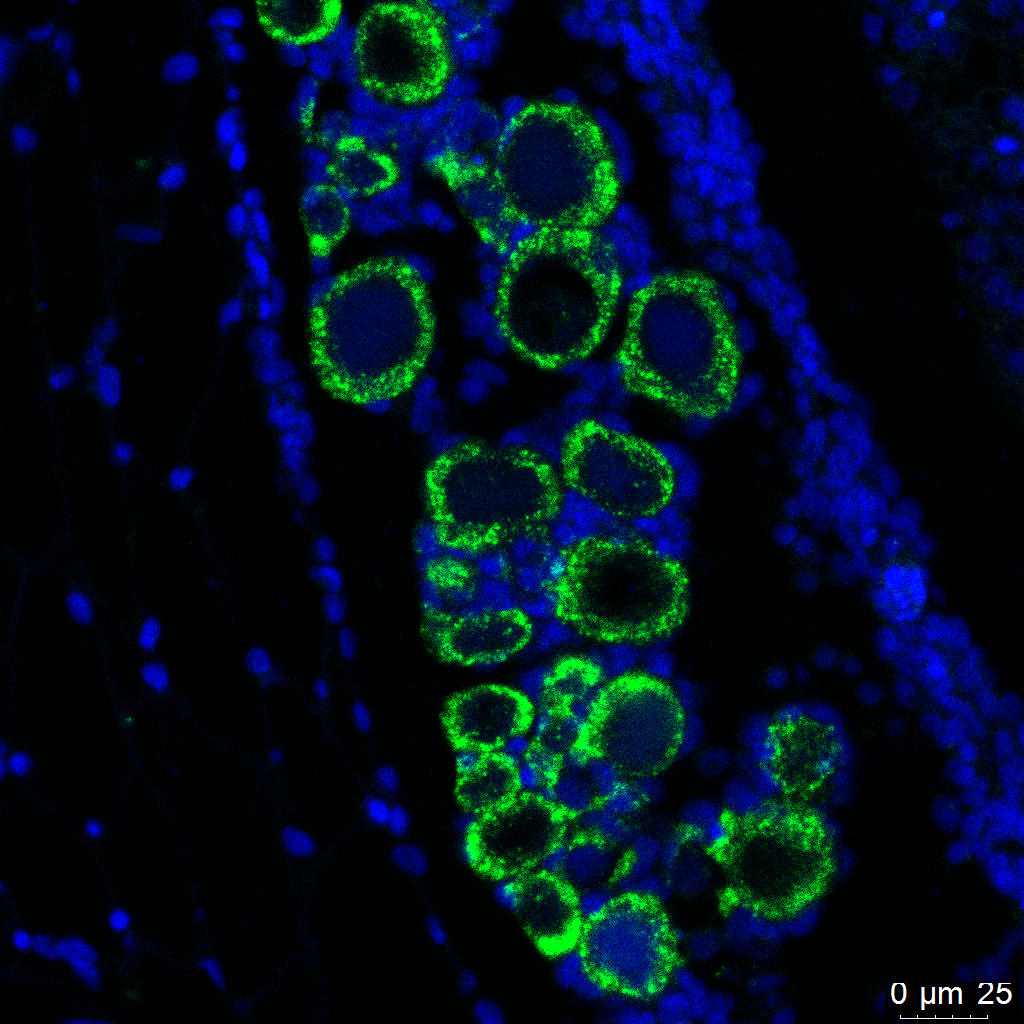

Supplement: Supplementary file 11 — Figure Source Data for Appendix Figures [file 44319_2026_775_MOESM11_ESM.zip › Source Data for Appendix Figure S1 3-7/Appendix Figure S4/Appendix Figure S4F/piwil2/Merge WT.tif]

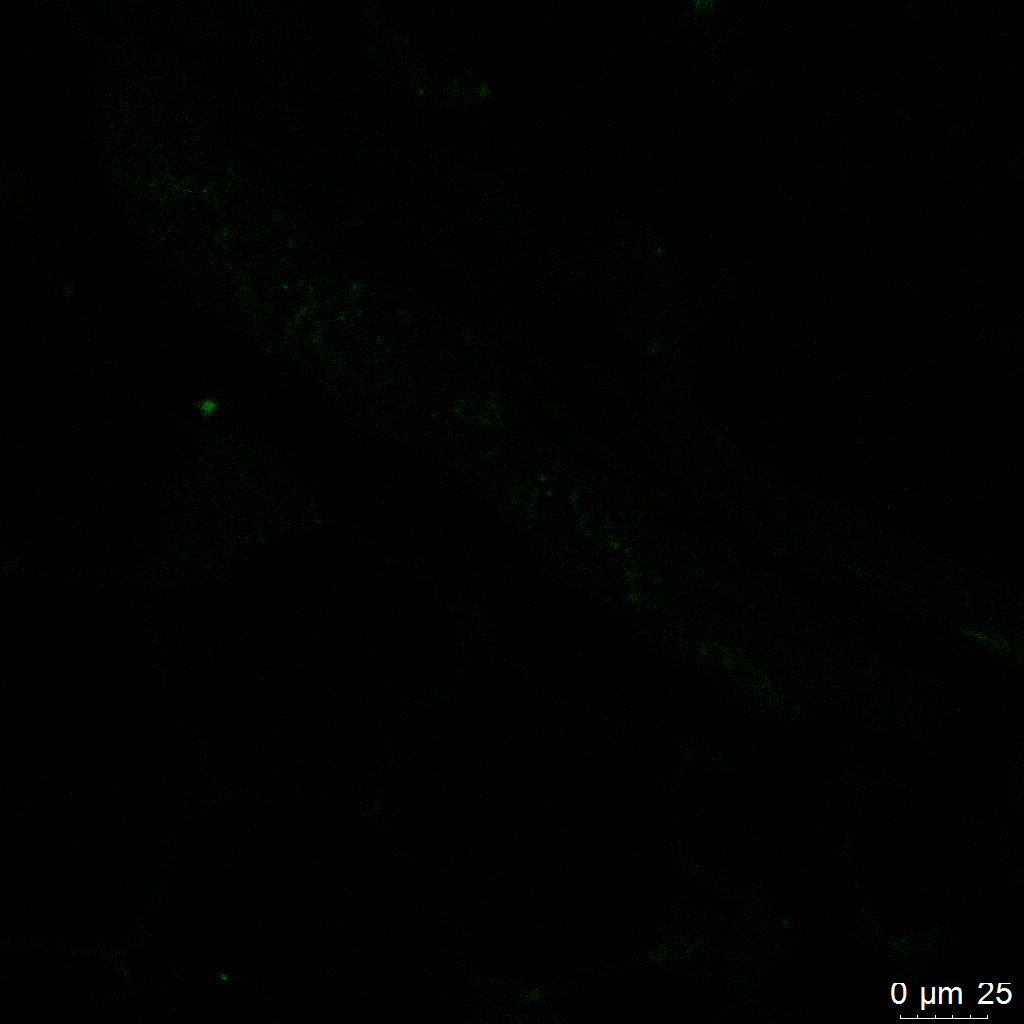

Supplement: Supplementary file 11 — Figure Source Data for Appendix Figures [file 44319_2026_775_MOESM11_ESM.zip › Source Data for Appendix Figure S1 3-7/Appendix Figure S4/Appendix Figure S4F/piwil2/piwil2 hom.tif]

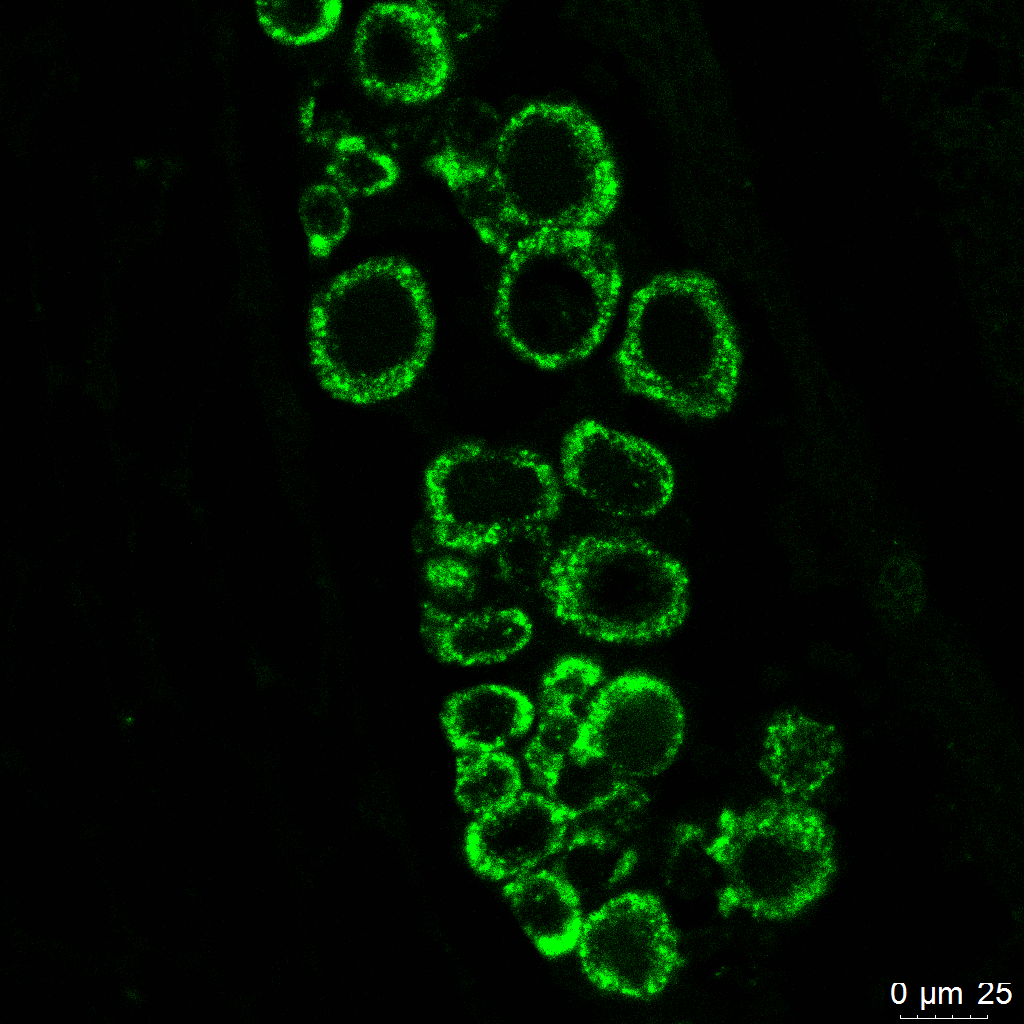

Supplement: Supplementary file 11 — Figure Source Data for Appendix Figures [file 44319_2026_775_MOESM11_ESM.zip › Source Data for Appendix Figure S1 3-7/Appendix Figure S4/Appendix Figure S4F/piwil2/piwil2 WT.tif]

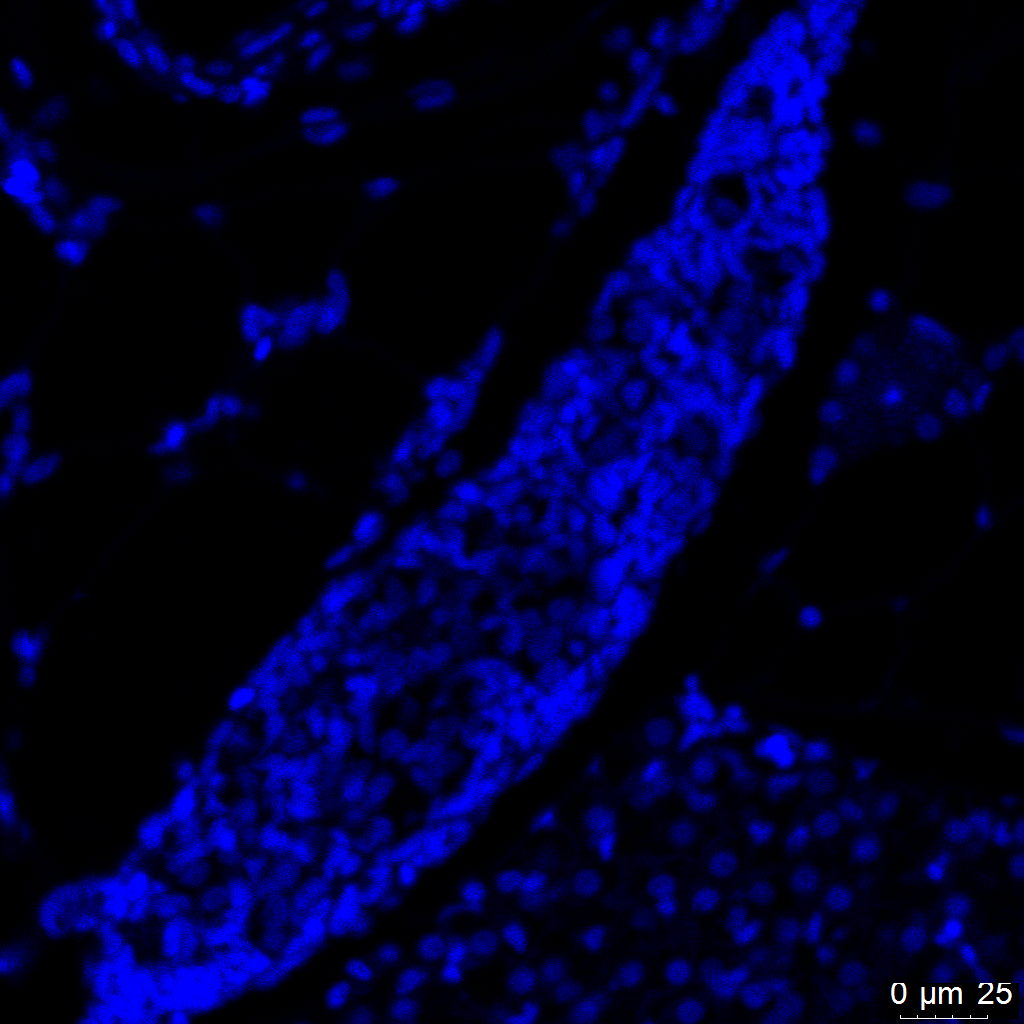

Supplement: Supplementary file 11 — Figure Source Data for Appendix Figures [file 44319_2026_775_MOESM11_ESM.zip › Source Data for Appendix Figure S1 3-7/Appendix Figure S4/Appendix Figure S4F/tdrd1/DAPI hom.tif]

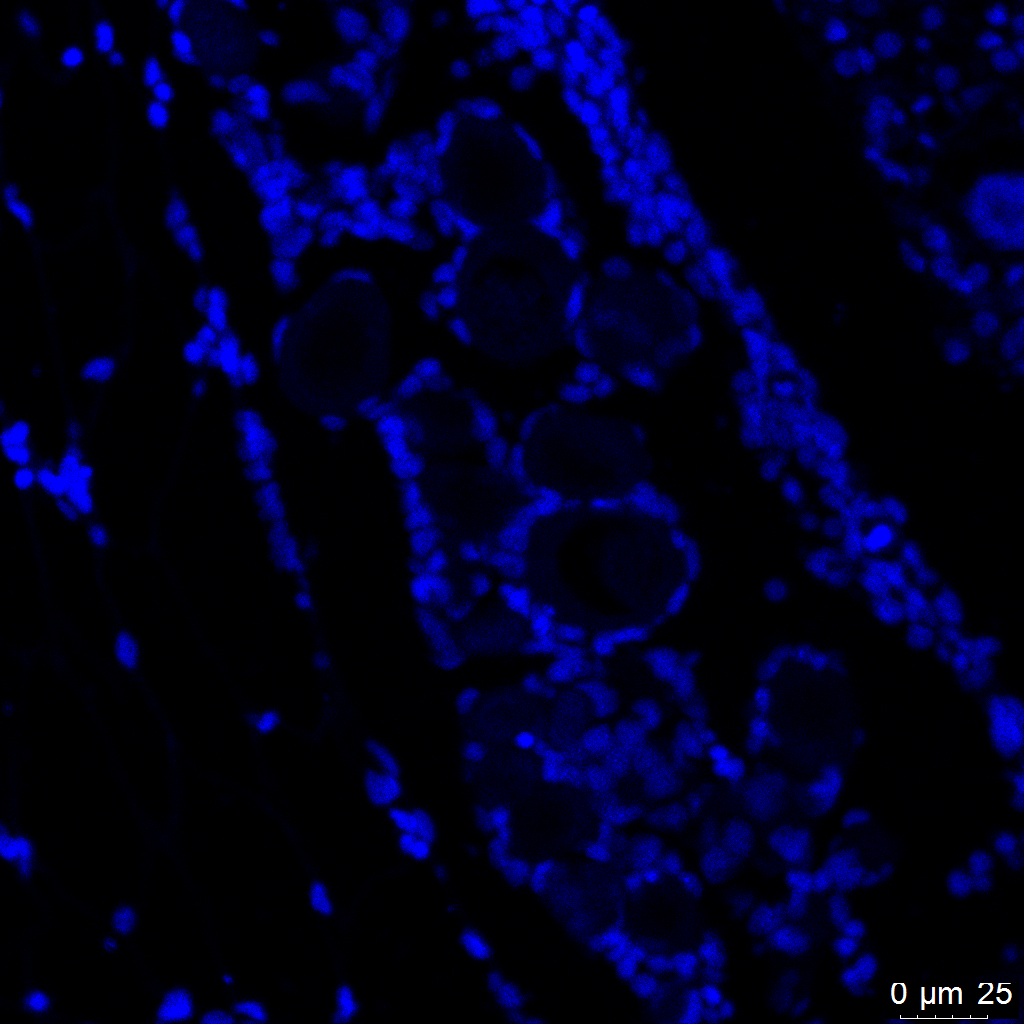

Supplement: Supplementary file 11 — Figure Source Data for Appendix Figures [file 44319_2026_775_MOESM11_ESM.zip › Source Data for Appendix Figure S1 3-7/Appendix Figure S4/Appendix Figure S4F/tdrd1/DAPI WT.tif]

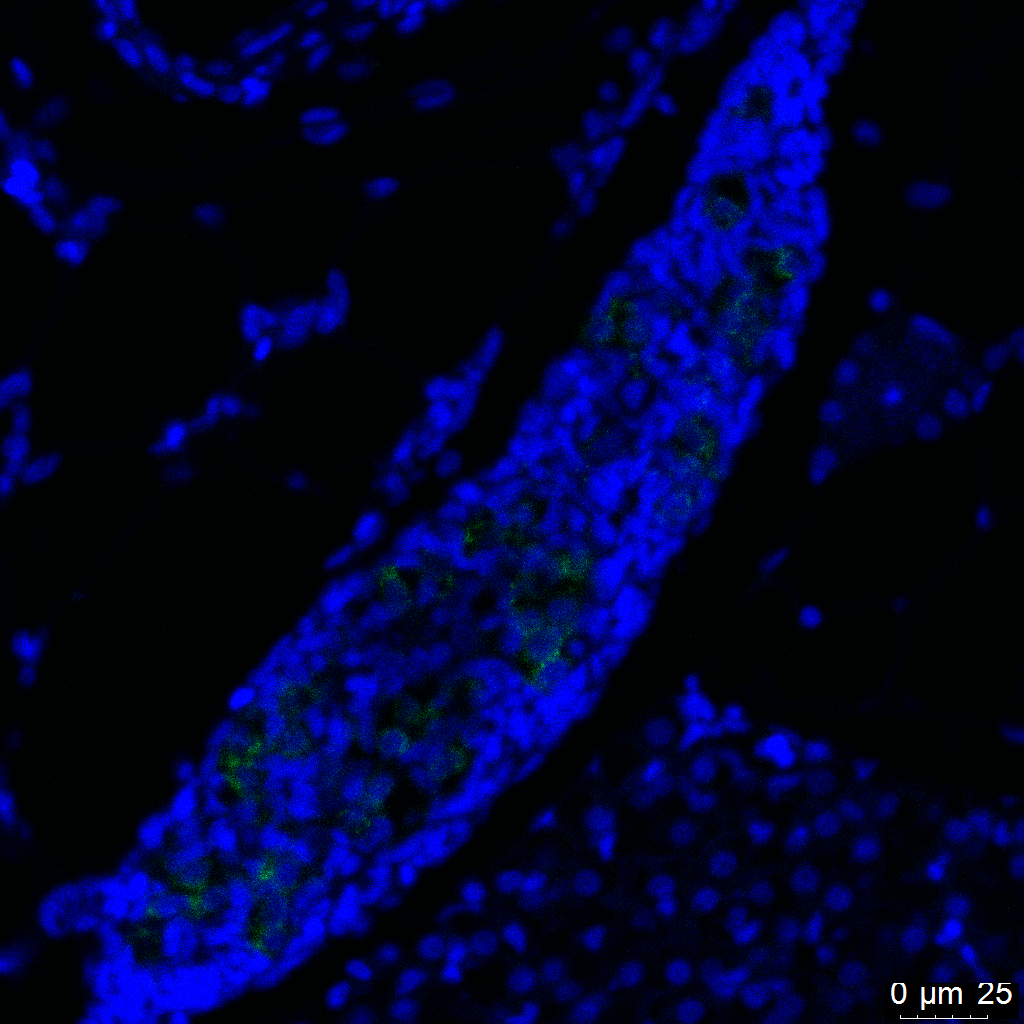

Supplement: Supplementary file 11 — Figure Source Data for Appendix Figures [file 44319_2026_775_MOESM11_ESM.zip › Source Data for Appendix Figure S1 3-7/Appendix Figure S4/Appendix Figure S4F/tdrd1/Merge hom.tif]

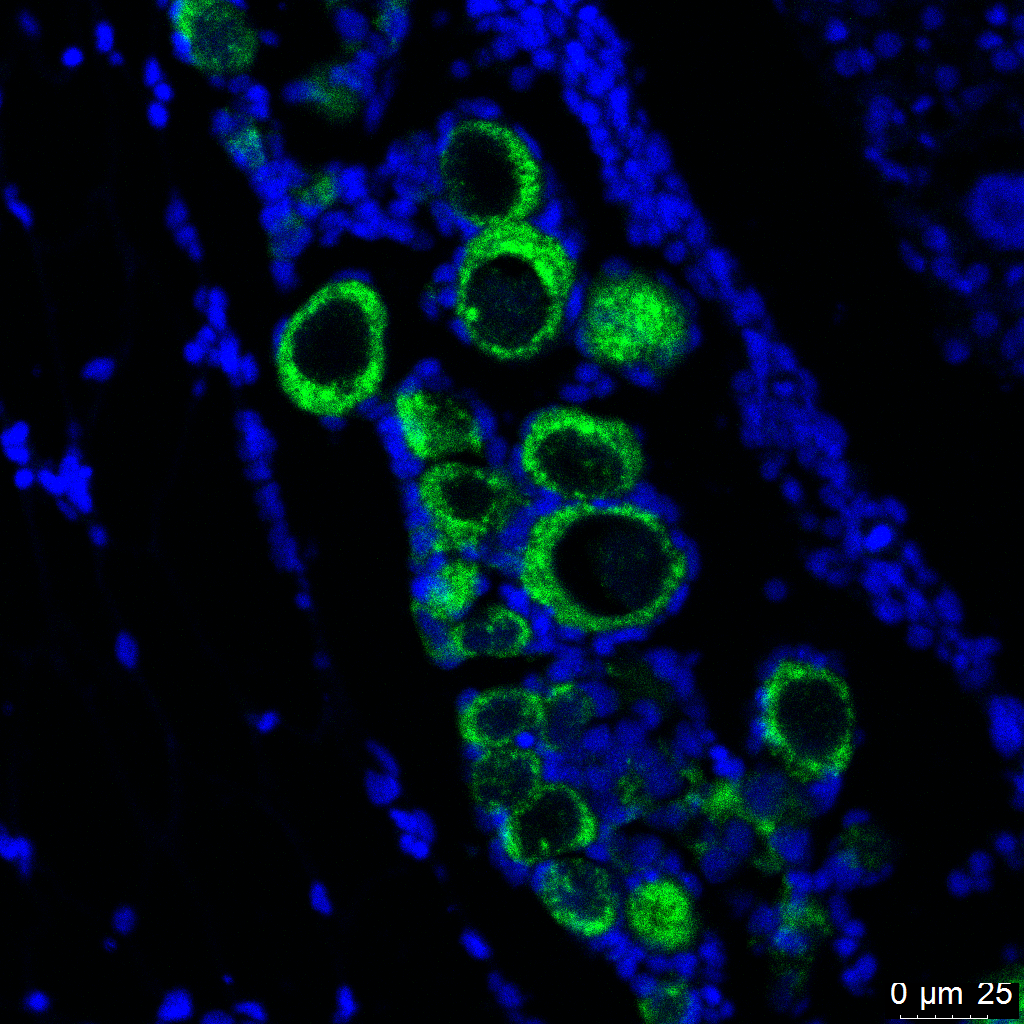

Supplement: Supplementary file 11 — Figure Source Data for Appendix Figures [file 44319_2026_775_MOESM11_ESM.zip › Source Data for Appendix Figure S1 3-7/Appendix Figure S4/Appendix Figure S4F/tdrd1/Merge WT.tif]

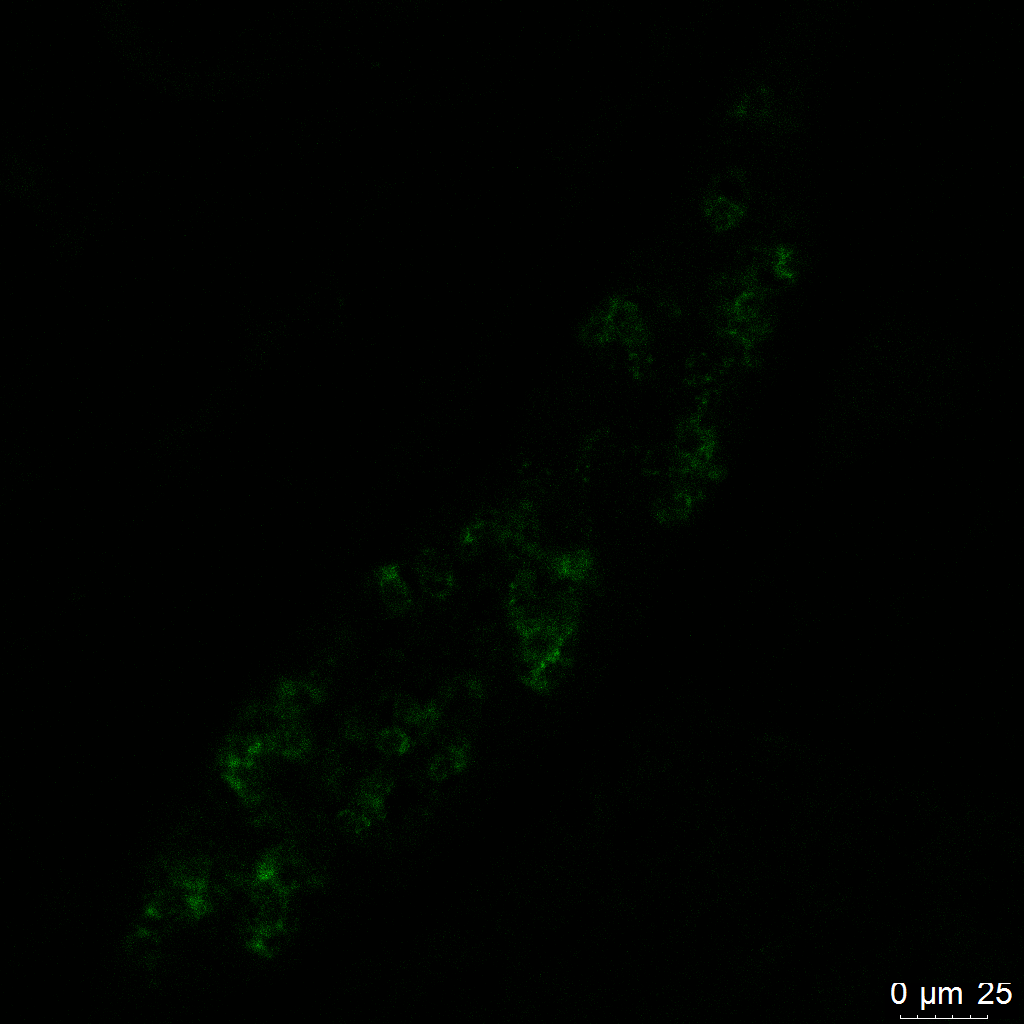

Supplement: Supplementary file 11 — Figure Source Data for Appendix Figures [file 44319_2026_775_MOESM11_ESM.zip › Source Data for Appendix Figure S1 3-7/Appendix Figure S4/Appendix Figure S4F/tdrd1/tdrd1 hom.tif]

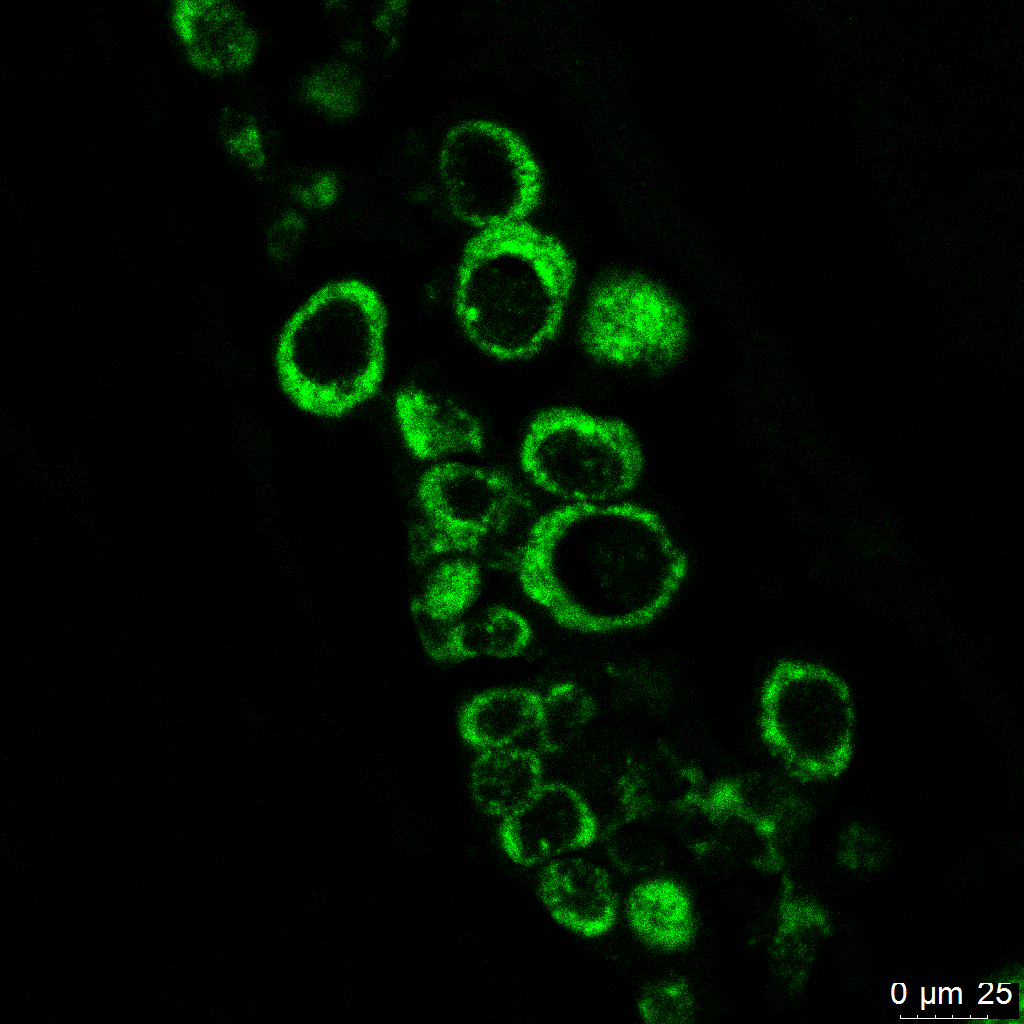

Supplement: Supplementary file 11 — Figure Source Data for Appendix Figures [file 44319_2026_775_MOESM11_ESM.zip › Source Data for Appendix Figure S1 3-7/Appendix Figure S4/Appendix Figure S4F/tdrd1/tdrd1 WT.tif]

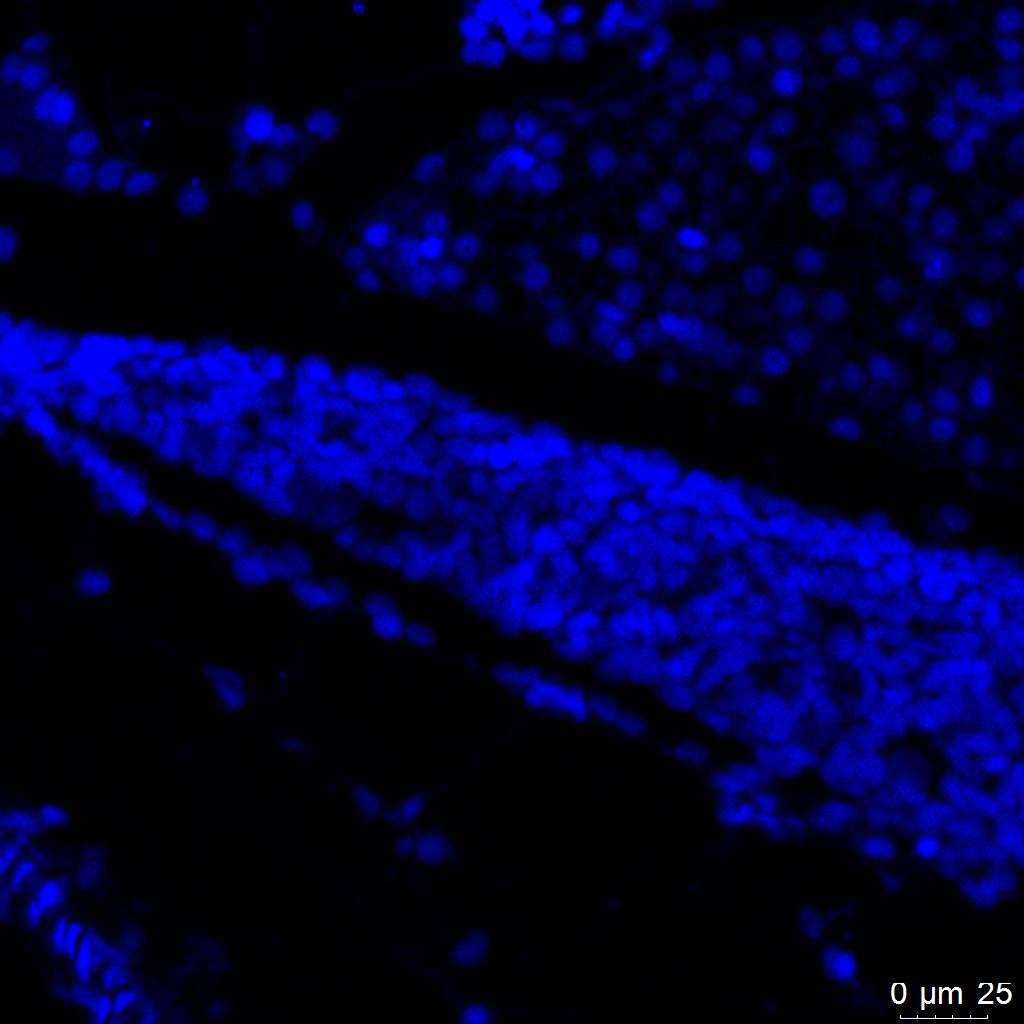

Supplement: Supplementary file 11 — Figure Source Data for Appendix Figures [file 44319_2026_775_MOESM11_ESM.zip › Source Data for Appendix Figure S1 3-7/Appendix Figure S4/Appendix Figure S4F/tdrd7a/DAPI hom.tif]

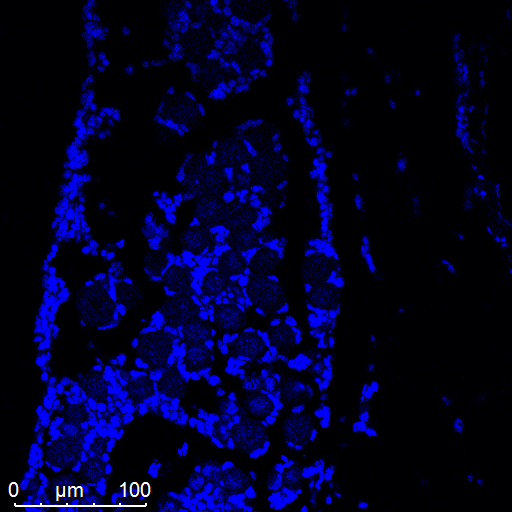

Supplement: Supplementary file 11 — Figure Source Data for Appendix Figures [file 44319_2026_775_MOESM11_ESM.zip › Source Data for Appendix Figure S1 3-7/Appendix Figure S4/Appendix Figure S4F/tdrd7a/DAPI WT.tif]

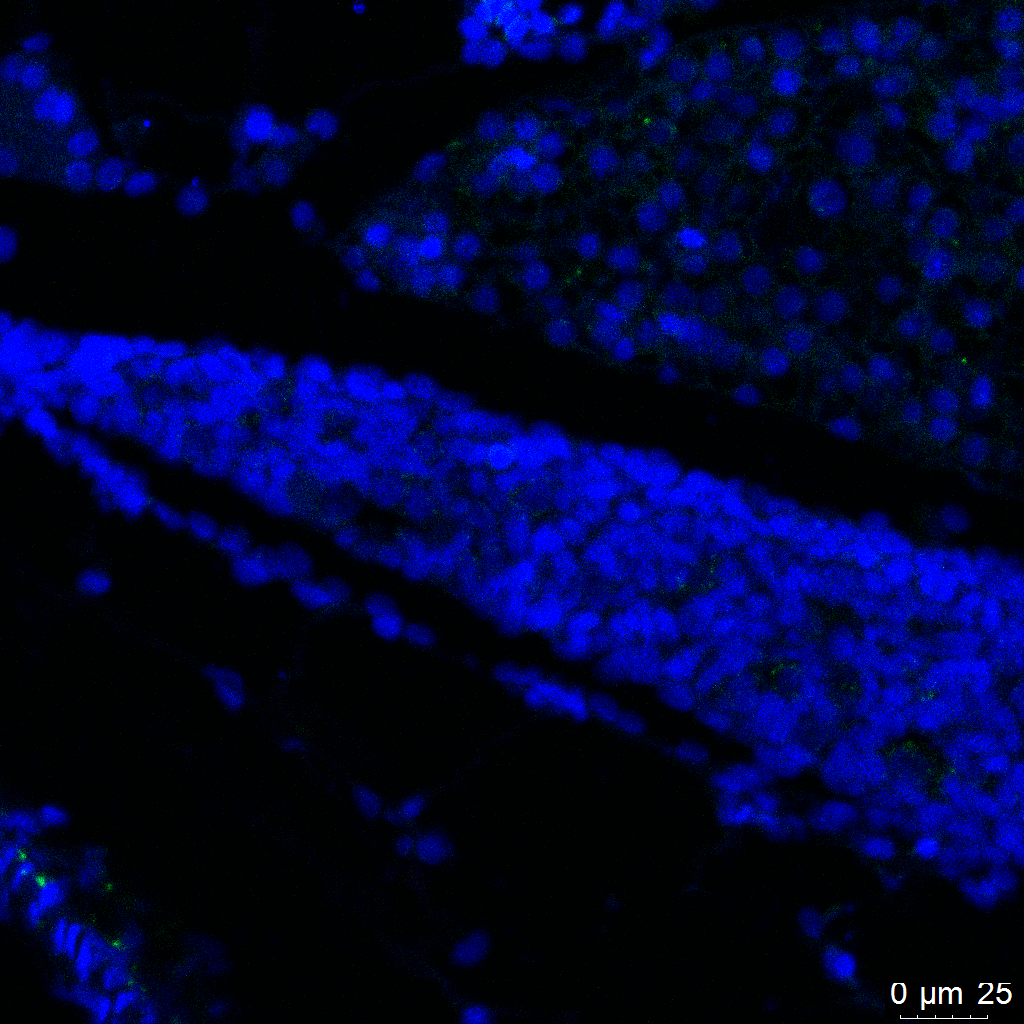

Supplement: Supplementary file 11 — Figure Source Data for Appendix Figures [file 44319_2026_775_MOESM11_ESM.zip › Source Data for Appendix Figure S1 3-7/Appendix Figure S4/Appendix Figure S4F/tdrd7a/Merge hom.tif]

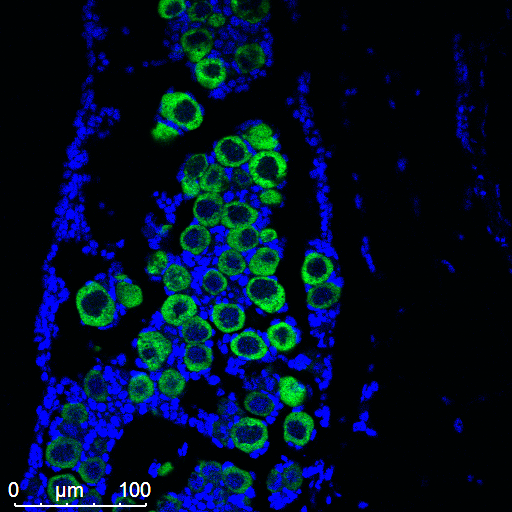

Supplement: Supplementary file 11 — Figure Source Data for Appendix Figures [file 44319_2026_775_MOESM11_ESM.zip › Source Data for Appendix Figure S1 3-7/Appendix Figure S4/Appendix Figure S4F/tdrd7a/Merge WT.tif]

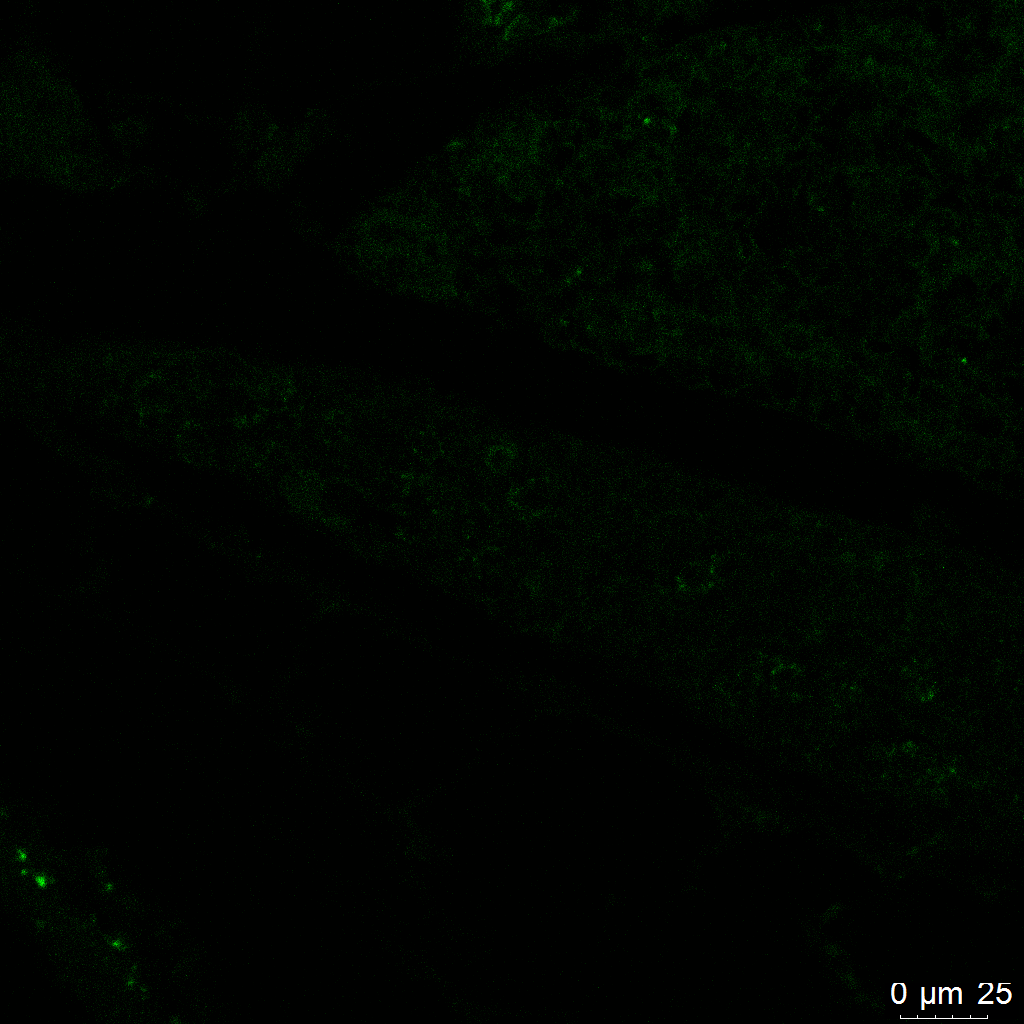

Supplement: Supplementary file 11 — Figure Source Data for Appendix Figures [file 44319_2026_775_MOESM11_ESM.zip › Source Data for Appendix Figure S1 3-7/Appendix Figure S4/Appendix Figure S4F/tdrd7a/tdrd7a hom.tif]

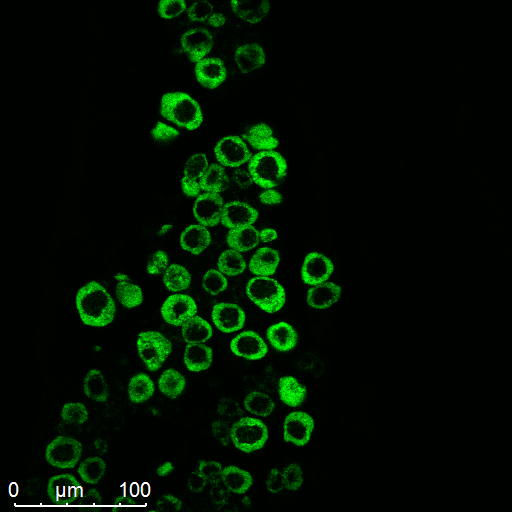

Supplement: Supplementary file 11 — Figure Source Data for Appendix Figures [file 44319_2026_775_MOESM11_ESM.zip › Source Data for Appendix Figure S1 3-7/Appendix Figure S4/Appendix Figure S4F/tdrd7a/tdrd7a WT.tif]

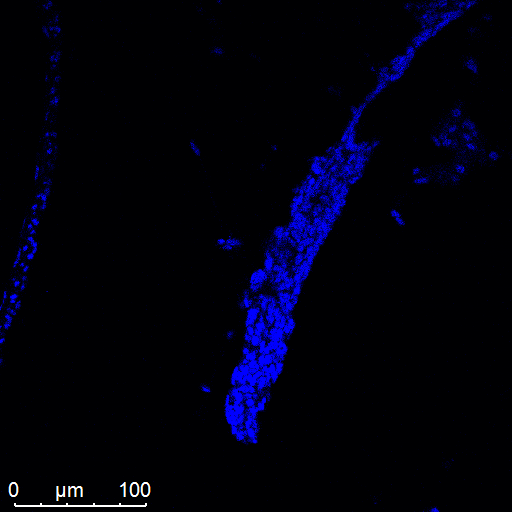

Supplement: Supplementary file 11 — Figure Source Data for Appendix Figures [file 44319_2026_775_MOESM11_ESM.zip › Source Data for Appendix Figure S1 3-7/Appendix Figure S4/Appendix Figure S4F/tdrd9/DAPI hom.tif]

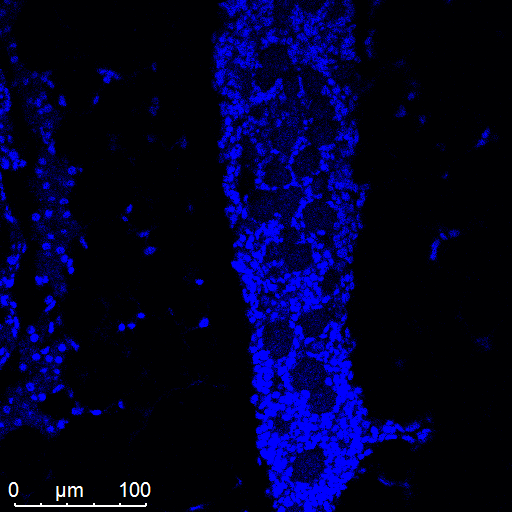

Supplement: Supplementary file 11 — Figure Source Data for Appendix Figures [file 44319_2026_775_MOESM11_ESM.zip › Source Data for Appendix Figure S1 3-7/Appendix Figure S4/Appendix Figure S4F/tdrd9/DAPI WT.tif]
