# Supplementary figures and images for: Stn1 promotes zebrafish oocyte development via amplifying Wnt/β-catenin signaling (part 5 of 5)
Source: EMBO Rep. 2026 Apr 17;27(12):3252–76. doi: 10.1038/s44319-026-00775-8 (PMC13304171; doi:10.1038/s44319-026-00775-8)

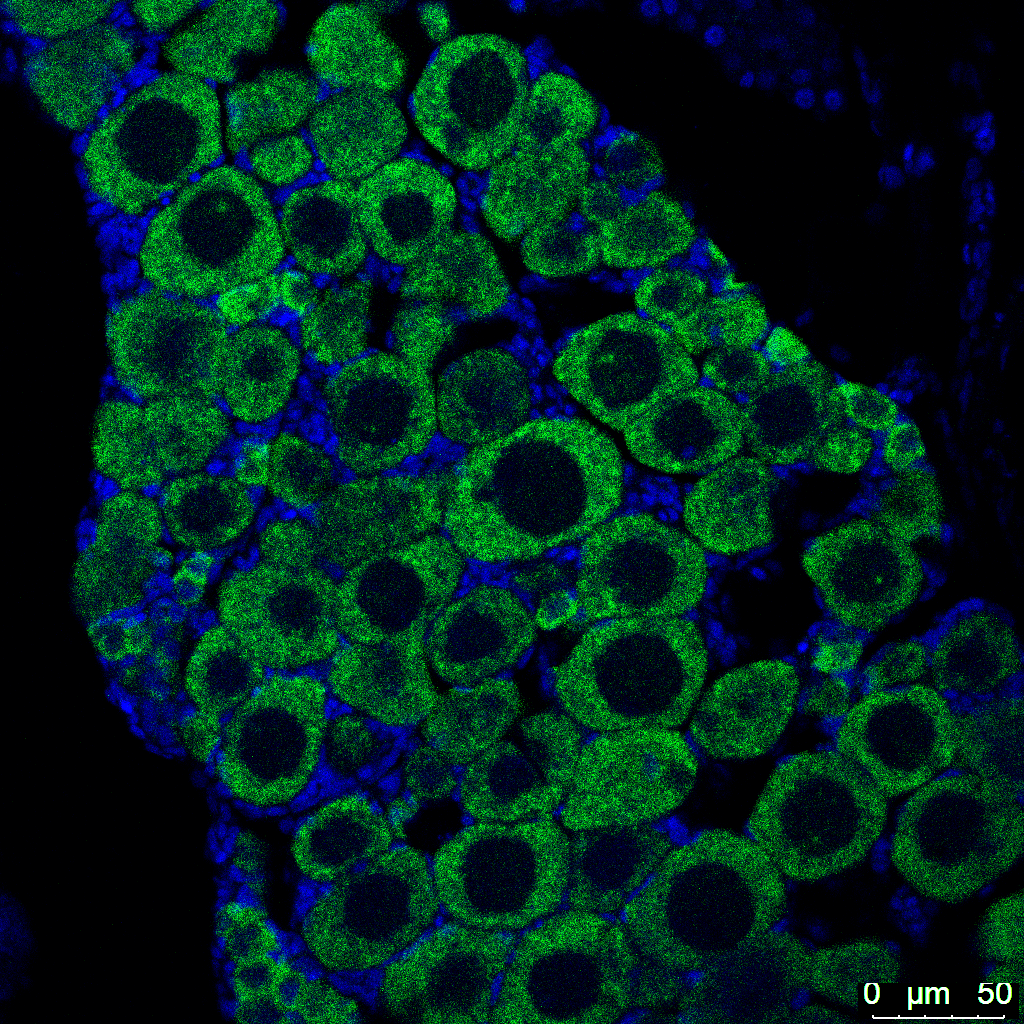

Supplement: Supplementary file 11 — Figure Source Data for Appendix Figures [file 44319_2026_775_MOESM11_ESM.zip › Source Data for Appendix Figure S1 3-7/Appendix Figure S7/Appendix Figure S7C/tdrd1/Merge WT.tif]

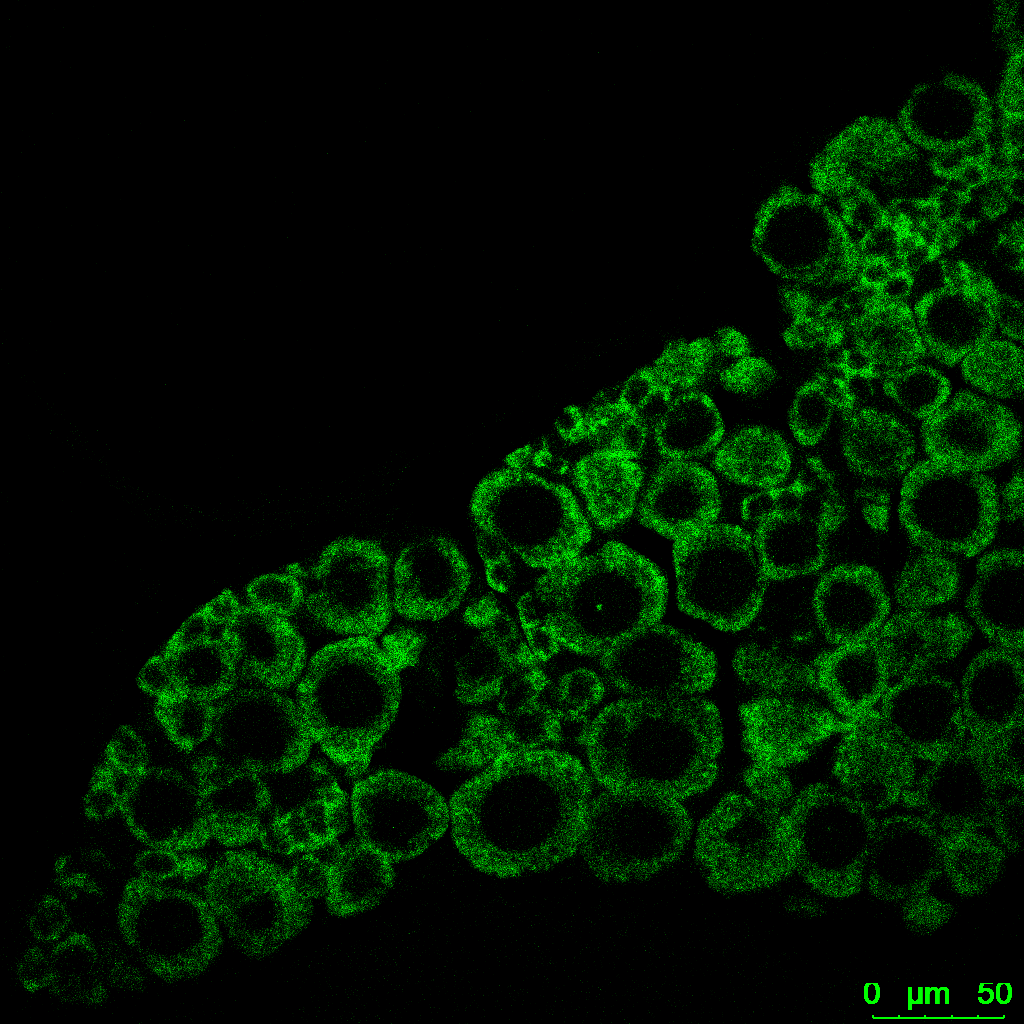

Supplement: Supplementary file 11 — Figure Source Data for Appendix Figures [file 44319_2026_775_MOESM11_ESM.zip › Source Data for Appendix Figure S1 3-7/Appendix Figure S7/Appendix Figure S7C/tdrd1/tdrd1 wnt8.tif]

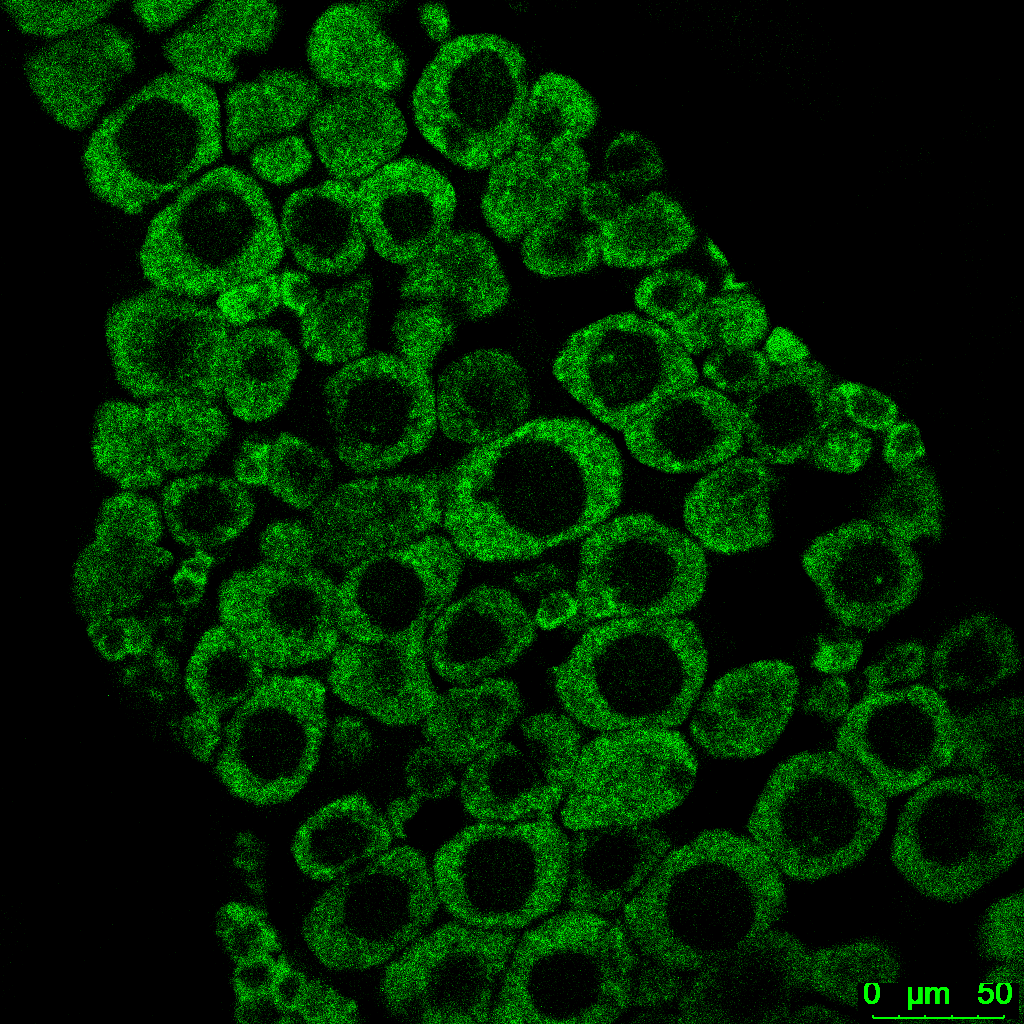

Supplement: Supplementary file 11 — Figure Source Data for Appendix Figures [file 44319_2026_775_MOESM11_ESM.zip › Source Data for Appendix Figure S1 3-7/Appendix Figure S7/Appendix Figure S7C/tdrd1/tdrd1 WT.tif]

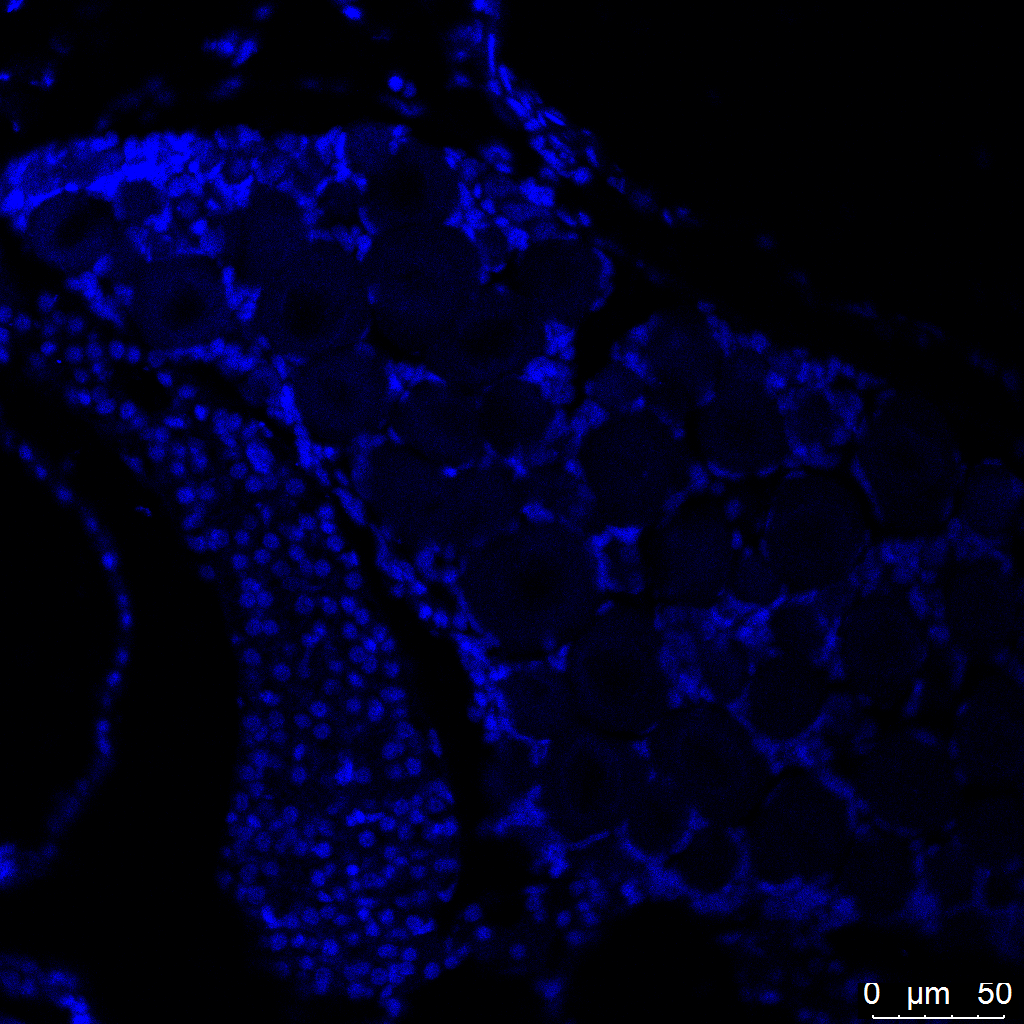

Supplement: Supplementary file 11 — Figure Source Data for Appendix Figures [file 44319_2026_775_MOESM11_ESM.zip › Source Data for Appendix Figure S1 3-7/Appendix Figure S7/Appendix Figure S7C/tdrd7a/DAPI wnt8.tif]

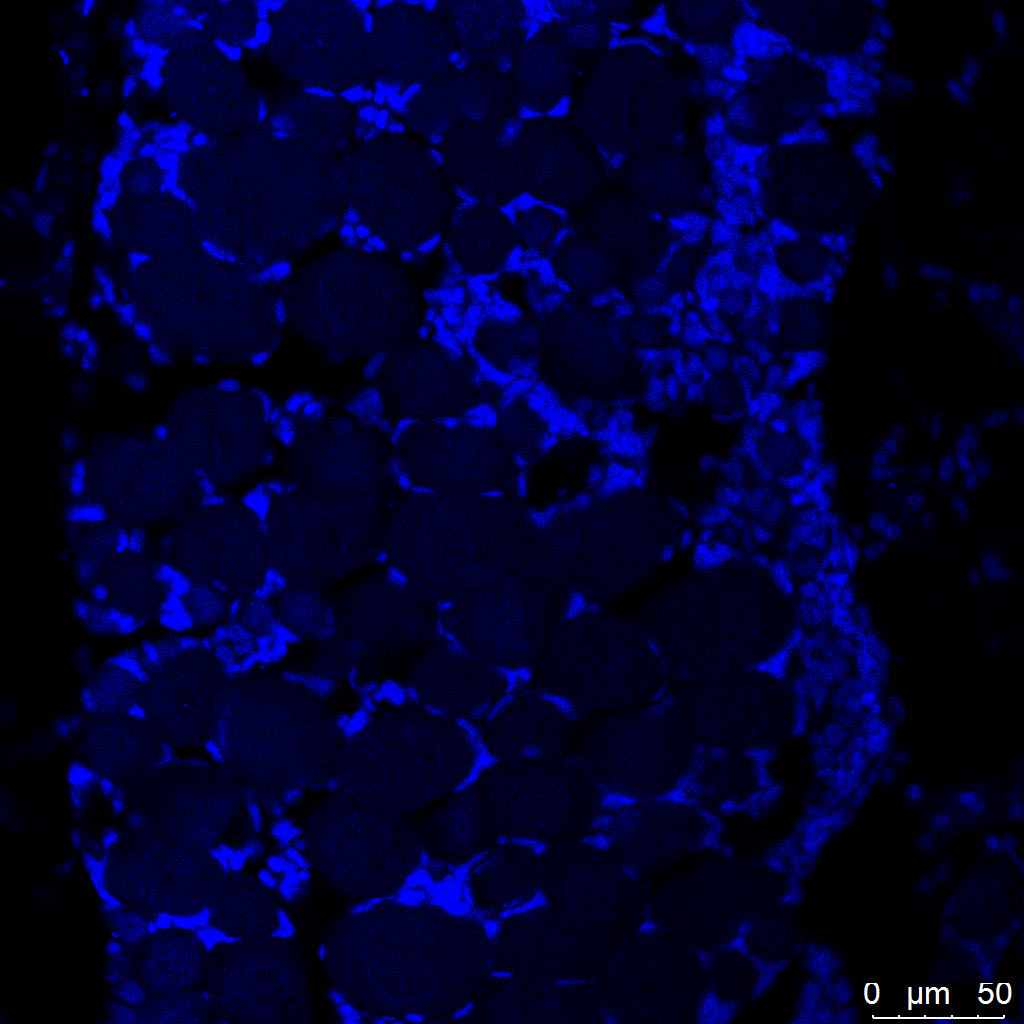

Supplement: Supplementary file 11 — Figure Source Data for Appendix Figures [file 44319_2026_775_MOESM11_ESM.zip › Source Data for Appendix Figure S1 3-7/Appendix Figure S7/Appendix Figure S7C/tdrd7a/DAPI WT.tif]

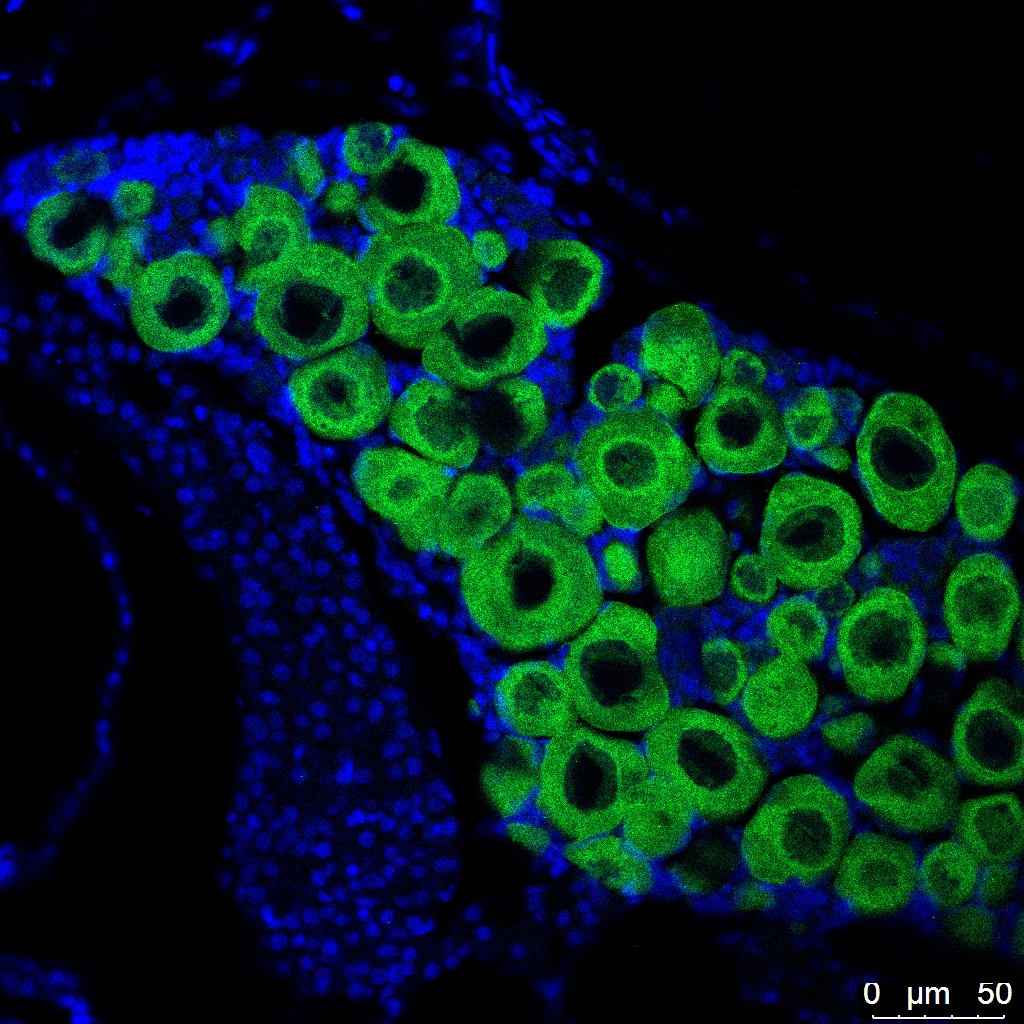

Supplement: Supplementary file 11 — Figure Source Data for Appendix Figures [file 44319_2026_775_MOESM11_ESM.zip › Source Data for Appendix Figure S1 3-7/Appendix Figure S7/Appendix Figure S7C/tdrd7a/Merge wnt8.tif]

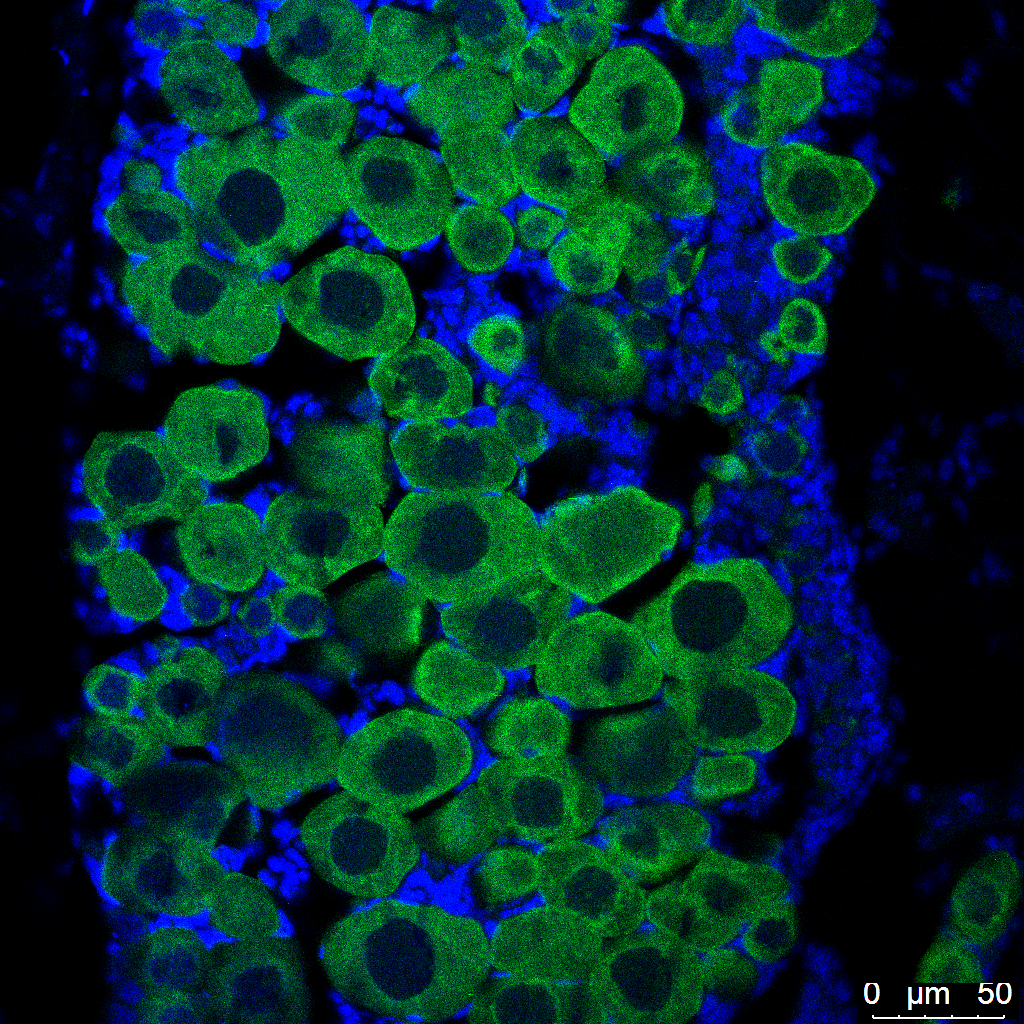

Supplement: Supplementary file 11 — Figure Source Data for Appendix Figures [file 44319_2026_775_MOESM11_ESM.zip › Source Data for Appendix Figure S1 3-7/Appendix Figure S7/Appendix Figure S7C/tdrd7a/Merge WT.tif]

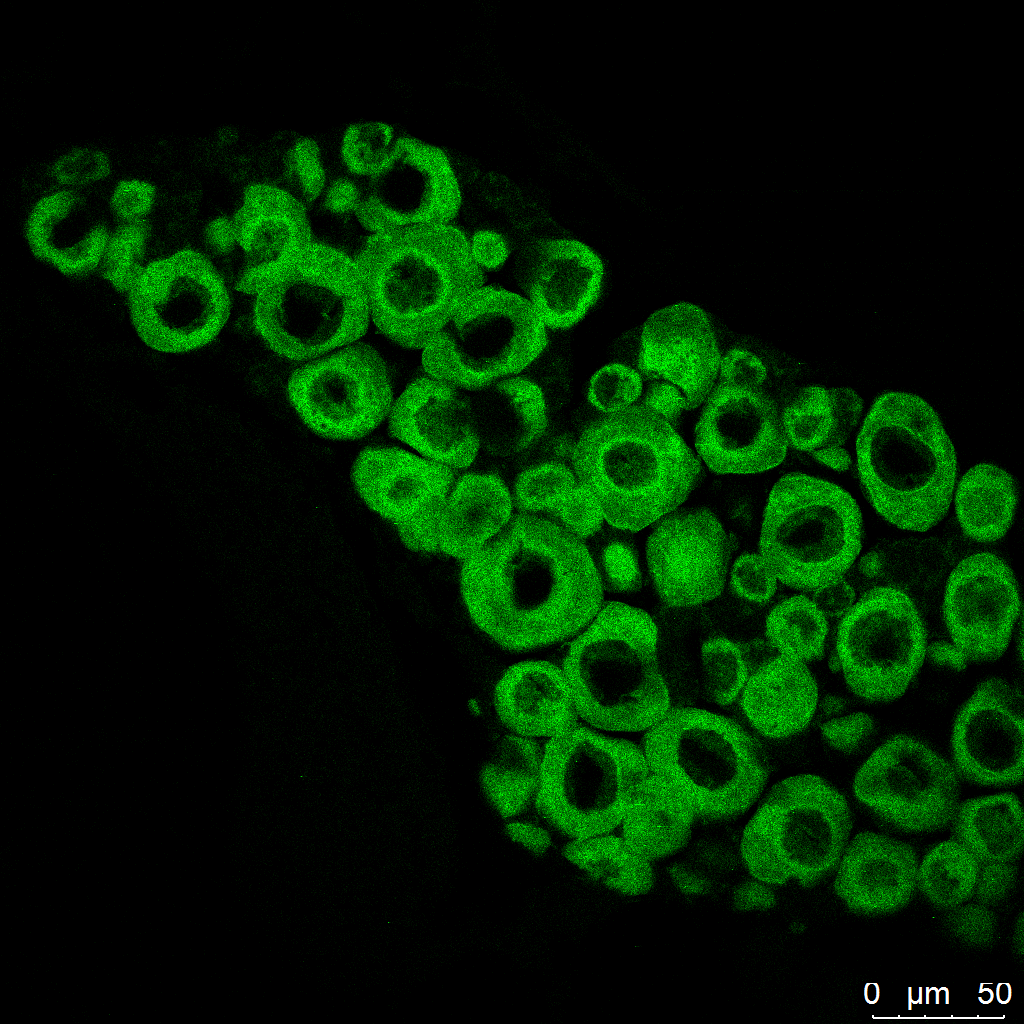

Supplement: Supplementary file 11 — Figure Source Data for Appendix Figures [file 44319_2026_775_MOESM11_ESM.zip › Source Data for Appendix Figure S1 3-7/Appendix Figure S7/Appendix Figure S7C/tdrd7a/tdrd7a wnt8.tif]

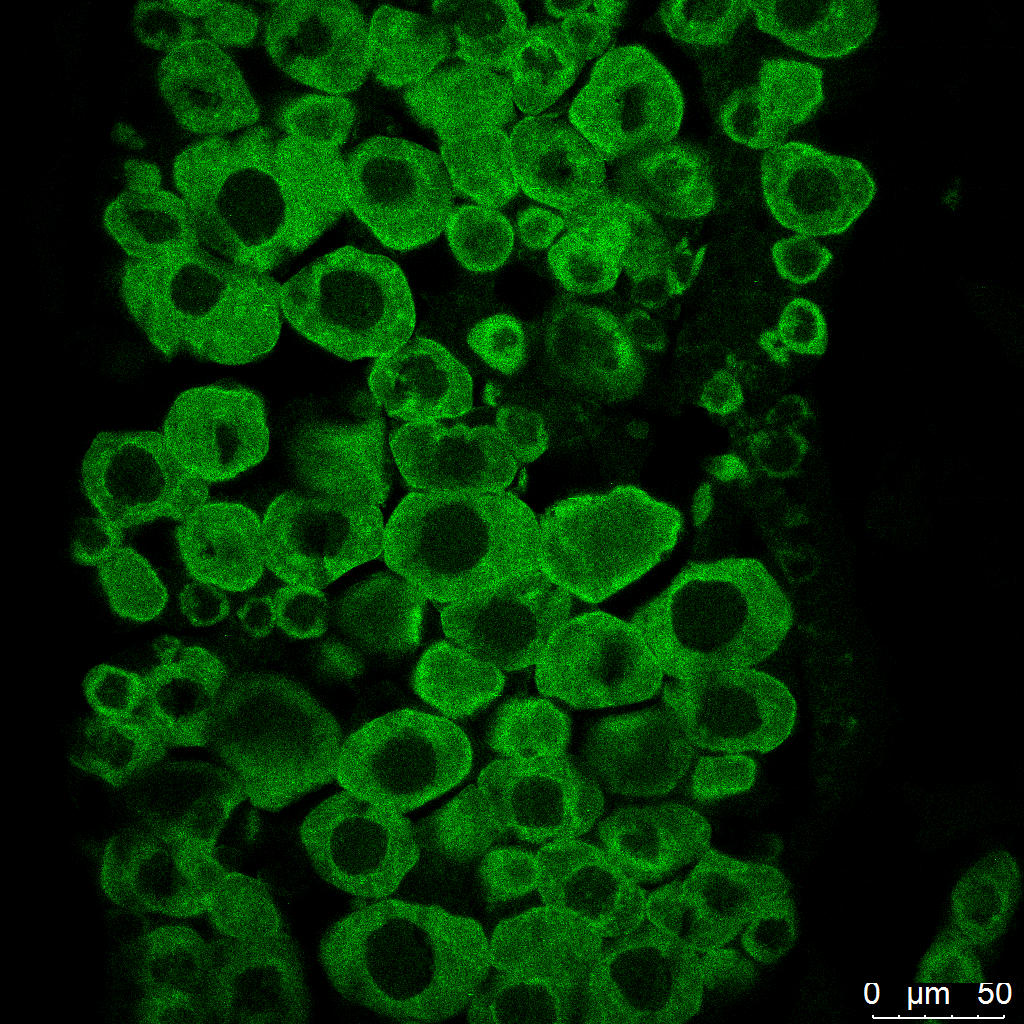

Supplement: Supplementary file 11 — Figure Source Data for Appendix Figures [file 44319_2026_775_MOESM11_ESM.zip › Source Data for Appendix Figure S1 3-7/Appendix Figure S7/Appendix Figure S7C/tdrd7a/tdrd7a WT.tif]

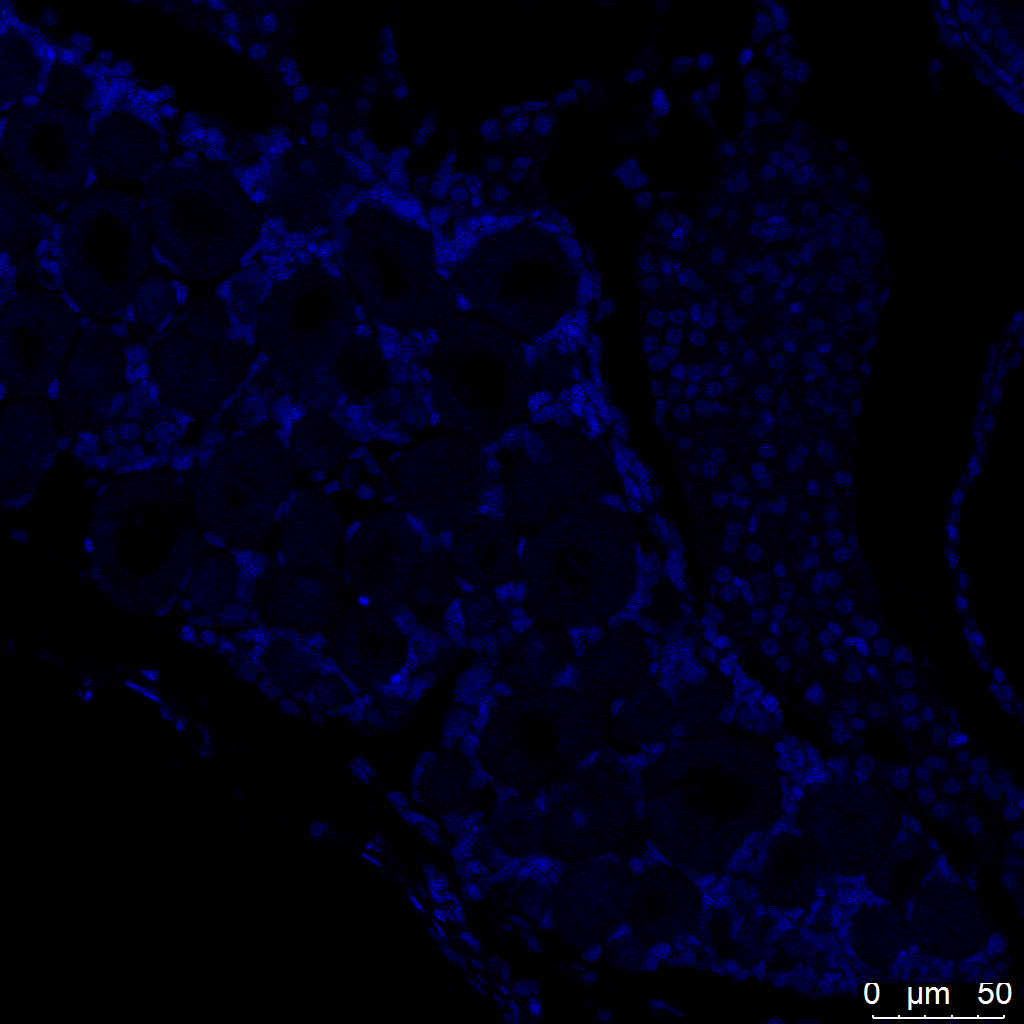

Supplement: Supplementary file 11 — Figure Source Data for Appendix Figures [file 44319_2026_775_MOESM11_ESM.zip › Source Data for Appendix Figure S1 3-7/Appendix Figure S7/Appendix Figure S7C/tdrd9/DAPI wnt8.tif]

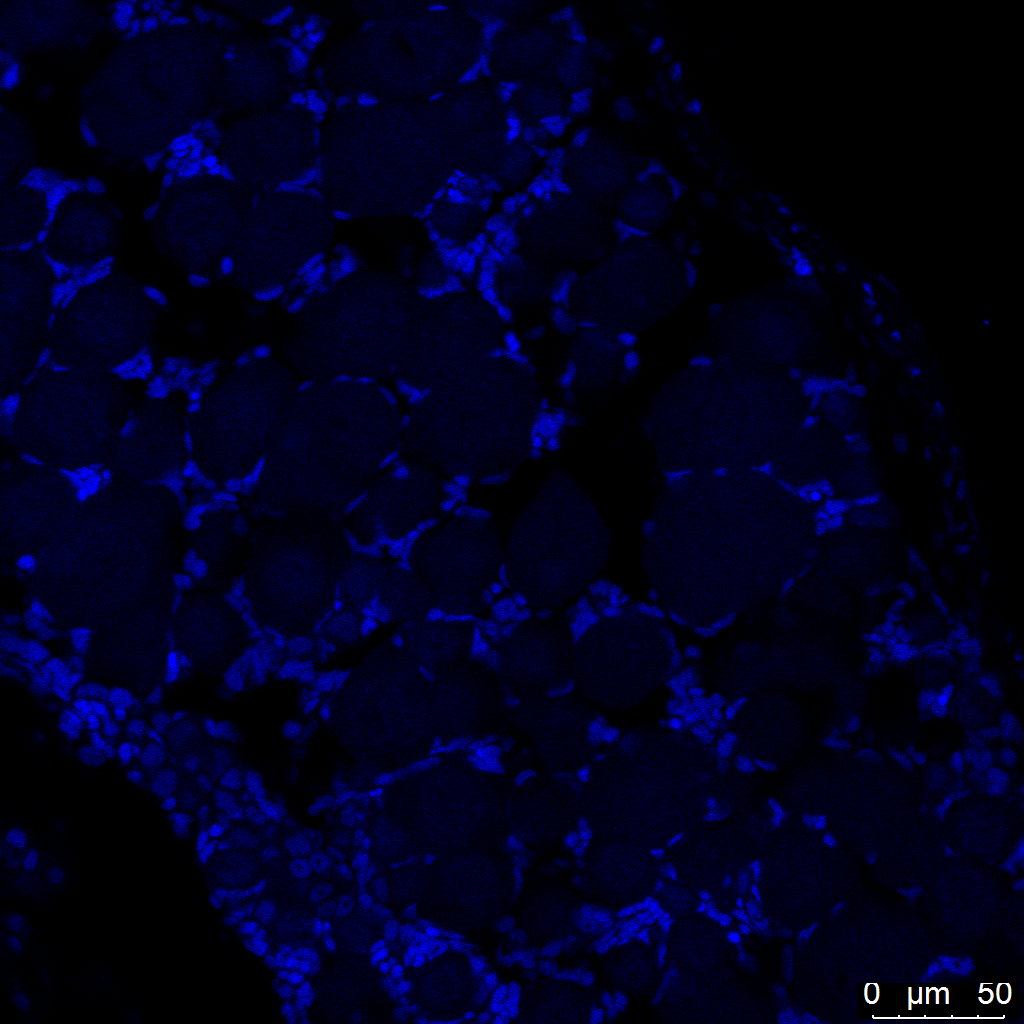

Supplement: Supplementary file 11 — Figure Source Data for Appendix Figures [file 44319_2026_775_MOESM11_ESM.zip › Source Data for Appendix Figure S1 3-7/Appendix Figure S7/Appendix Figure S7C/tdrd9/DAPI WT.tif]

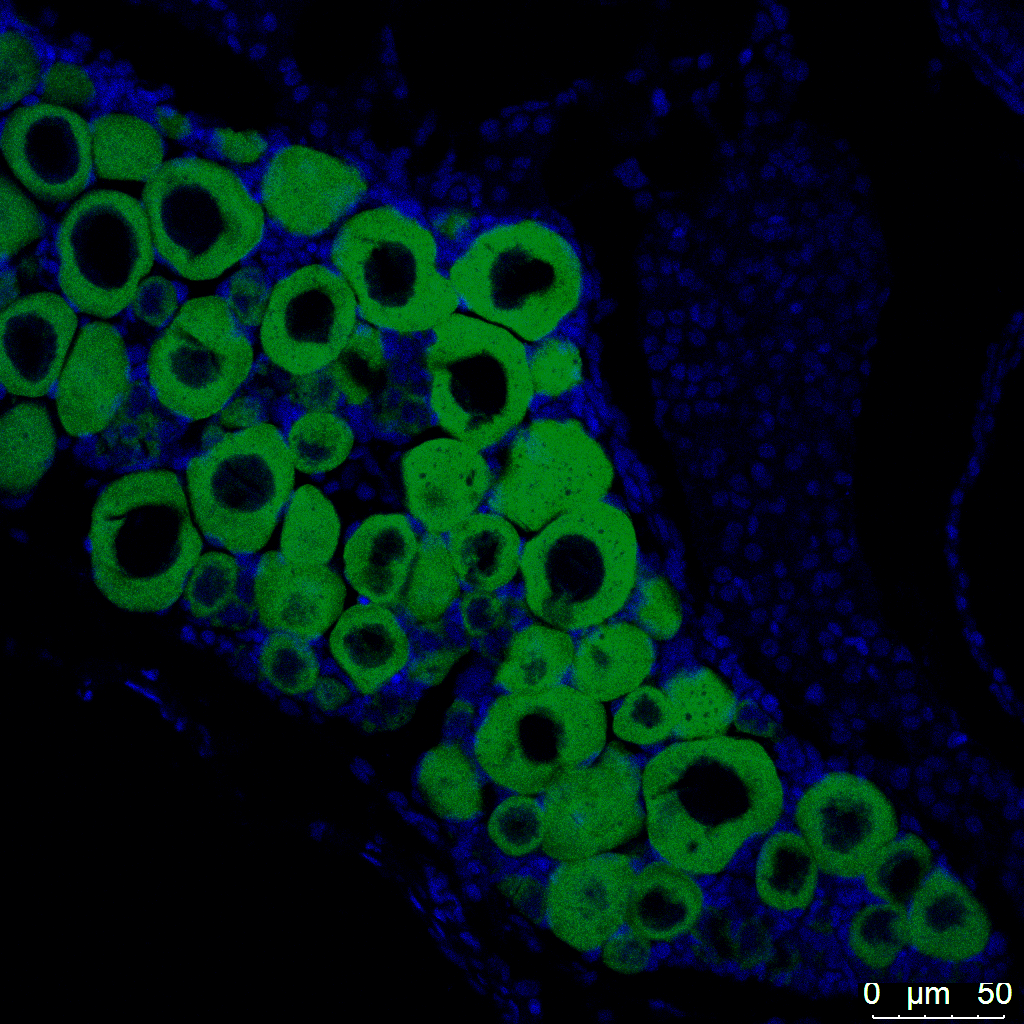

Supplement: Supplementary file 11 — Figure Source Data for Appendix Figures [file 44319_2026_775_MOESM11_ESM.zip › Source Data for Appendix Figure S1 3-7/Appendix Figure S7/Appendix Figure S7C/tdrd9/Merge wnt8.tif]

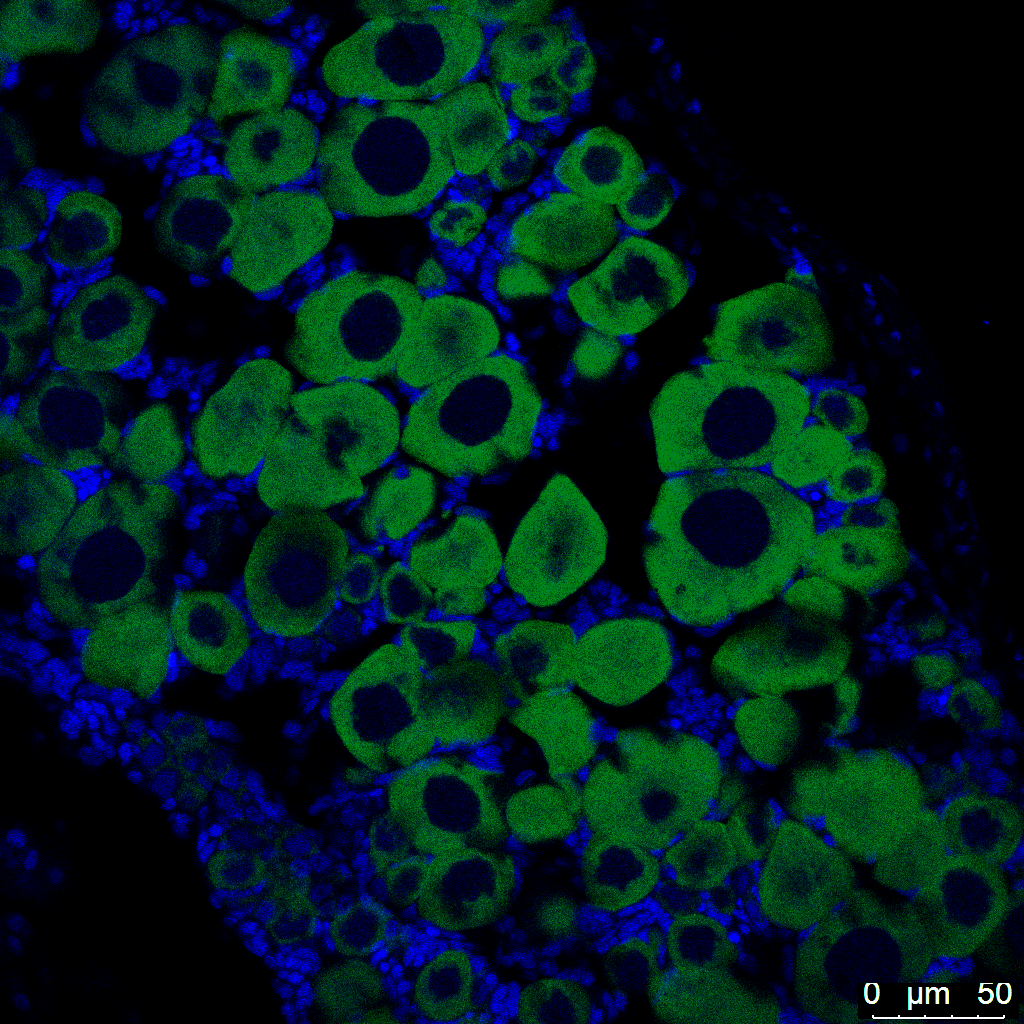

Supplement: Supplementary file 11 — Figure Source Data for Appendix Figures [file 44319_2026_775_MOESM11_ESM.zip › Source Data for Appendix Figure S1 3-7/Appendix Figure S7/Appendix Figure S7C/tdrd9/Merge WT.tif]

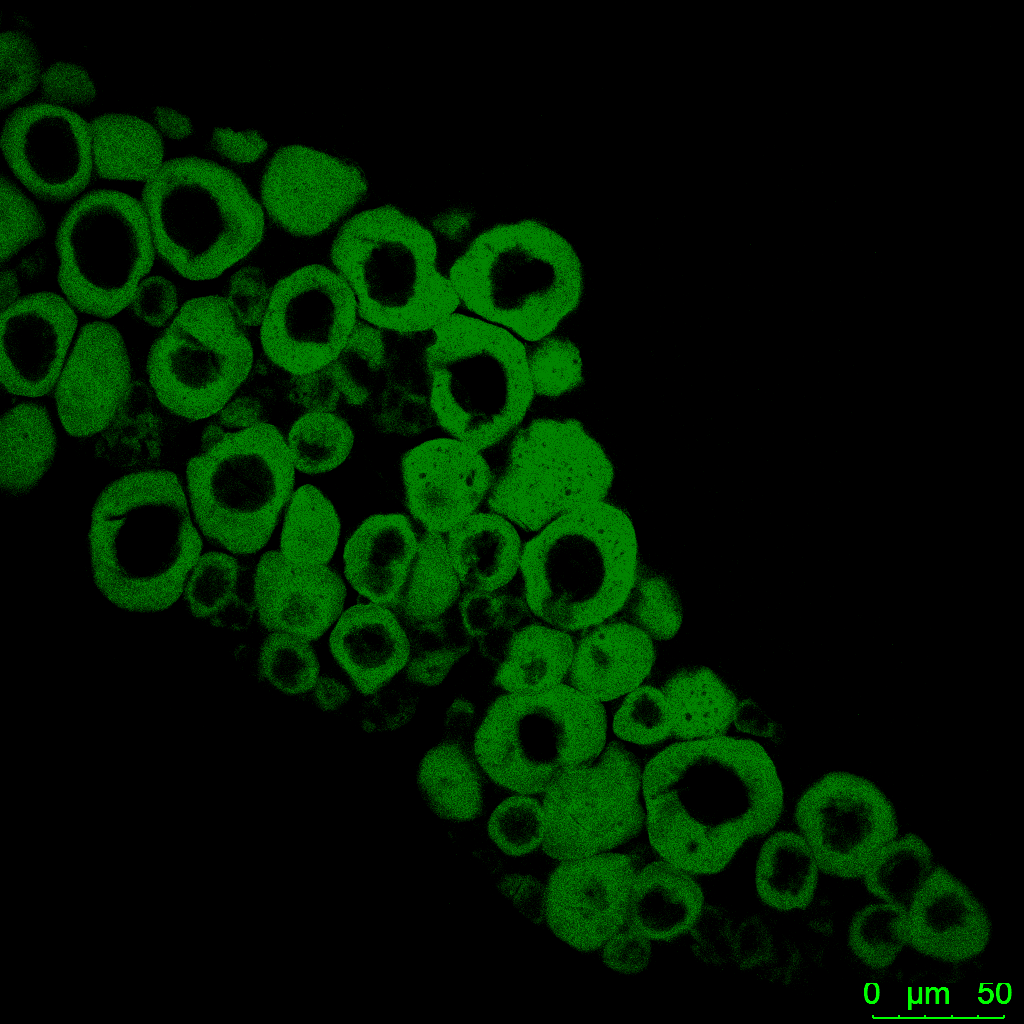

Supplement: Supplementary file 11 — Figure Source Data for Appendix Figures [file 44319_2026_775_MOESM11_ESM.zip › Source Data for Appendix Figure S1 3-7/Appendix Figure S7/Appendix Figure S7C/tdrd9/tdrd9 wnt8.tif]

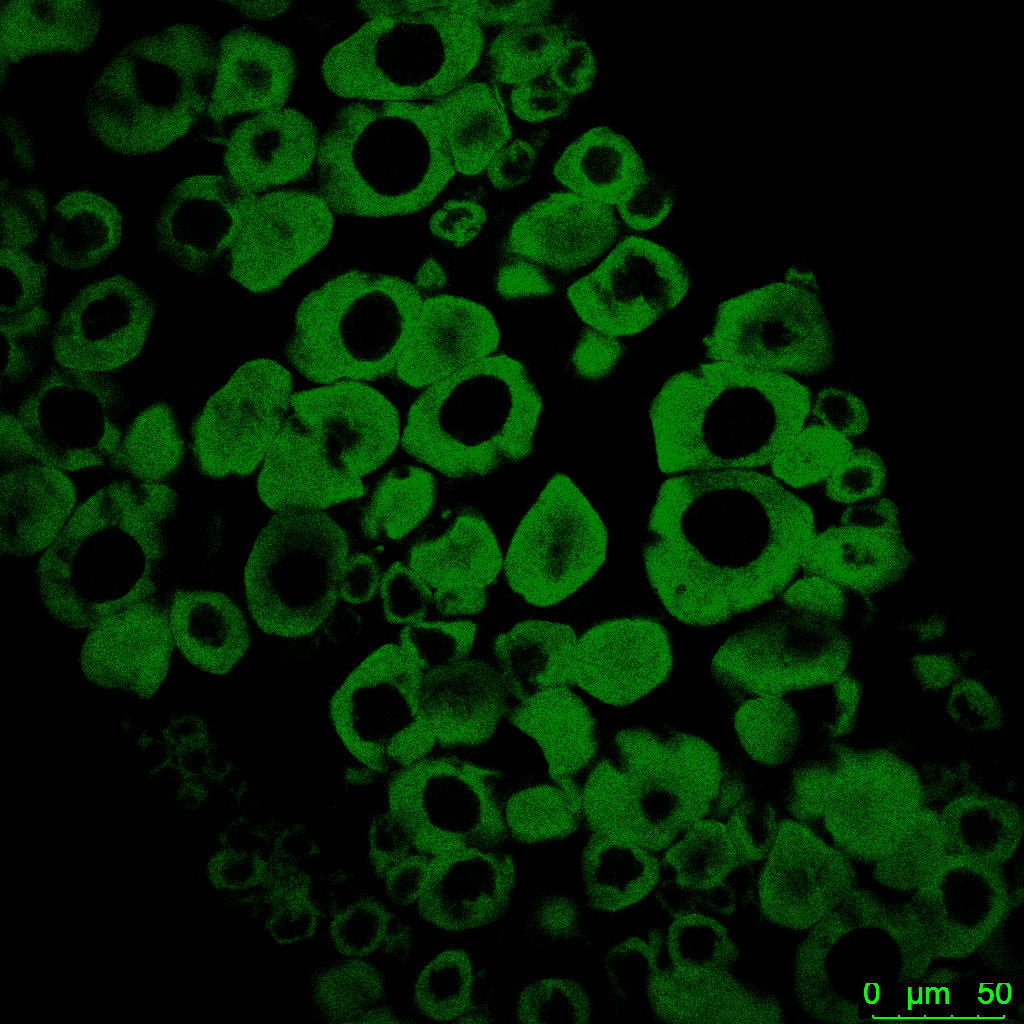

Supplement: Supplementary file 11 — Figure Source Data for Appendix Figures [file 44319_2026_775_MOESM11_ESM.zip › Source Data for Appendix Figure S1 3-7/Appendix Figure S7/Appendix Figure S7C/tdrd9/tdrd9 WT.tif]
