# Supplementary figures and images for: Stn1 promotes zebrafish oocyte development via amplifying Wnt/β-catenin signaling (part 4 of 5)
Source: EMBO Rep. 2026 Apr 17;27(12):3252–76. doi: 10.1038/s44319-026-00775-8 (PMC13304171; doi:10.1038/s44319-026-00775-8)

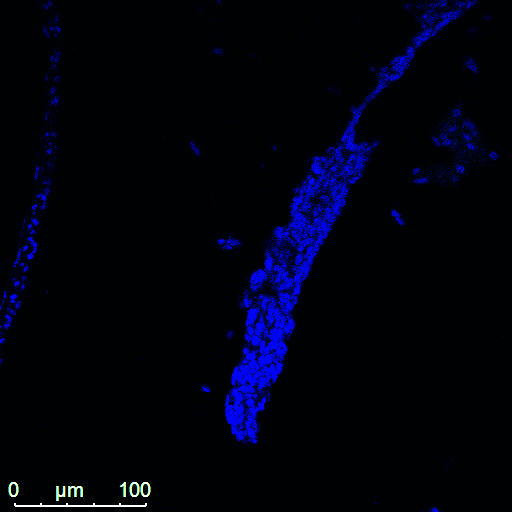

Supplement: Supplementary file 11 — Figure Source Data for Appendix Figures [file 44319_2026_775_MOESM11_ESM.zip › Source Data for Appendix Figure S1 3-7/Appendix Figure S4/Appendix Figure S4F/tdrd9/Merge hom.tif]

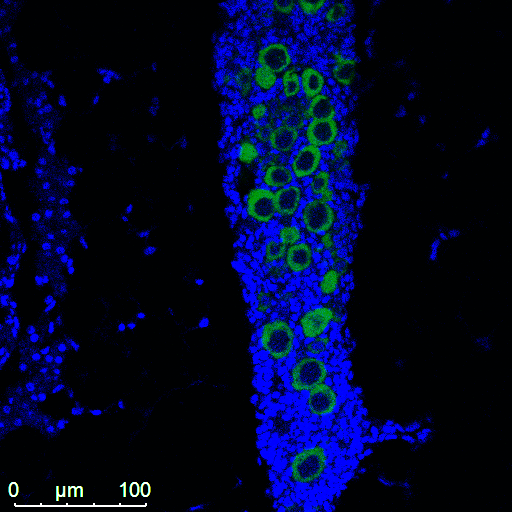

Supplement: Supplementary file 11 — Figure Source Data for Appendix Figures [file 44319_2026_775_MOESM11_ESM.zip › Source Data for Appendix Figure S1 3-7/Appendix Figure S4/Appendix Figure S4F/tdrd9/Merge WT.tif]

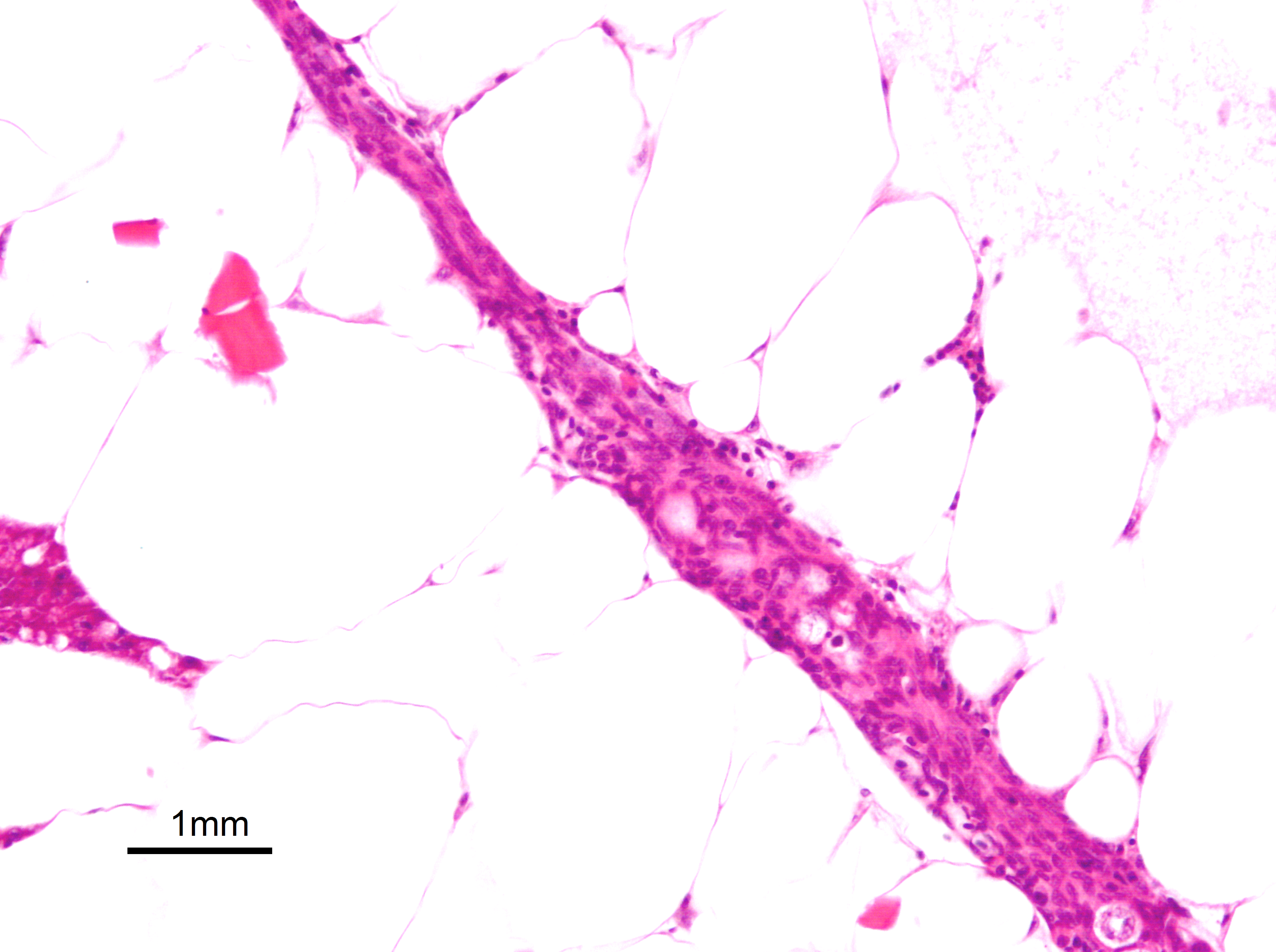

Supplement: Supplementary file 11 — Figure Source Data for Appendix Figures [file 44319_2026_775_MOESM11_ESM.zip › Source Data for Appendix Figure S1 3-7/Appendix Figure S5/Appendix Figure S5B/BIBR1532 hom.tif]

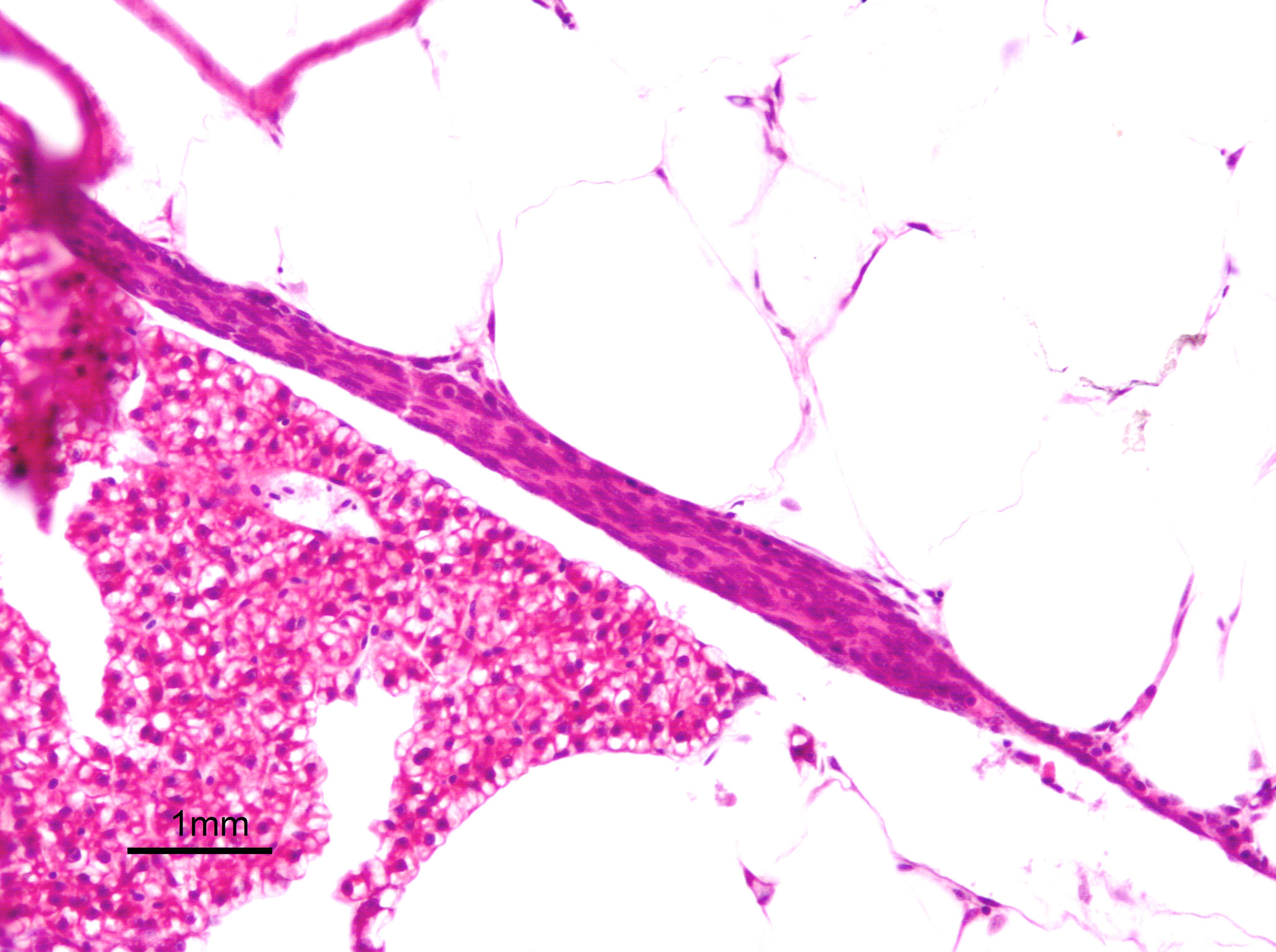

Supplement: Supplementary file 11 — Figure Source Data for Appendix Figures [file 44319_2026_775_MOESM11_ESM.zip › Source Data for Appendix Figure S1 3-7/Appendix Figure S5/Appendix Figure S5B/DMSO hom.tif]

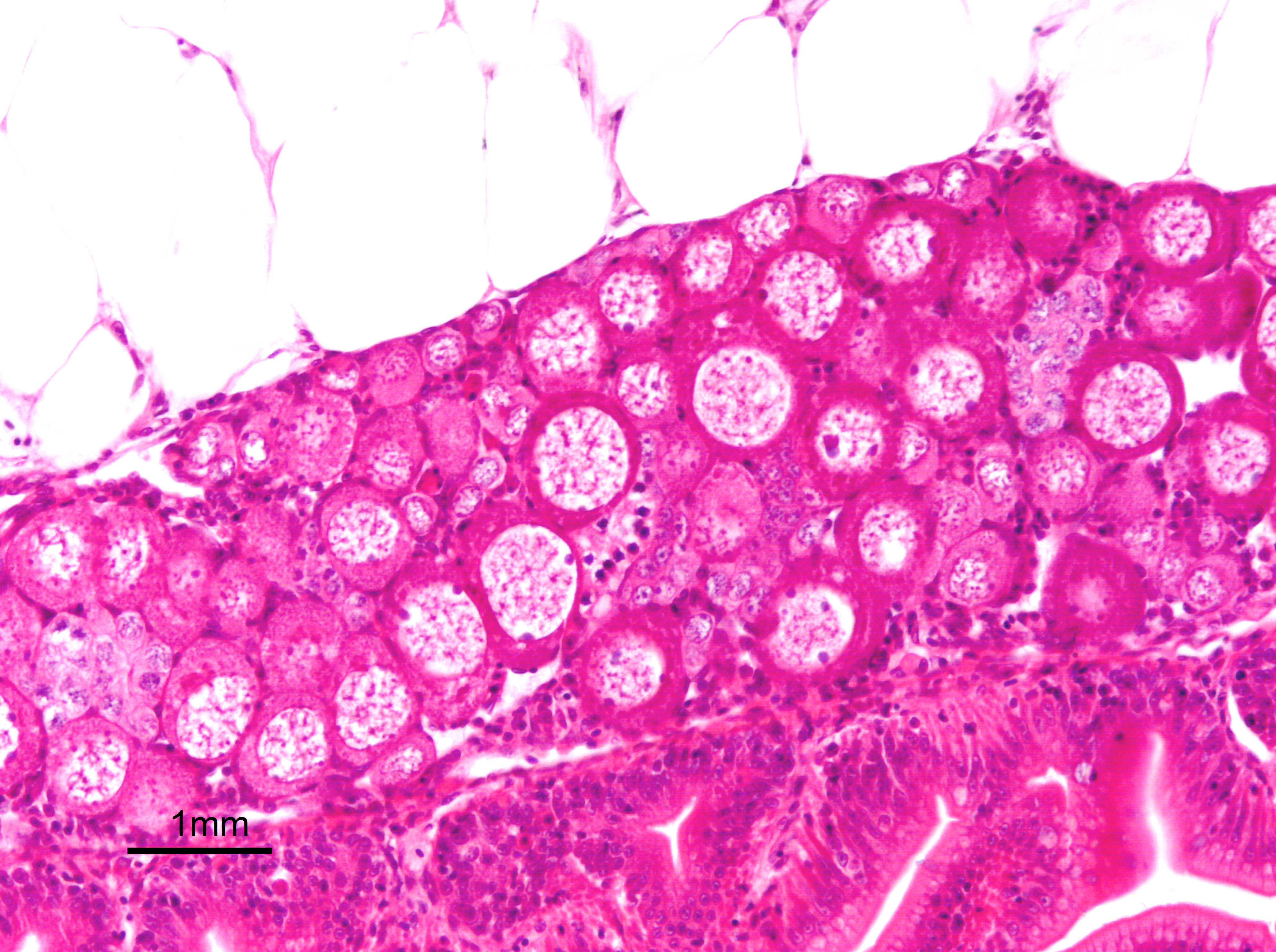

Supplement: Supplementary file 11 — Figure Source Data for Appendix Figures [file 44319_2026_775_MOESM11_ESM.zip › Source Data for Appendix Figure S1 3-7/Appendix Figure S5/Appendix Figure S5B/DMSO WT ovary.tif]

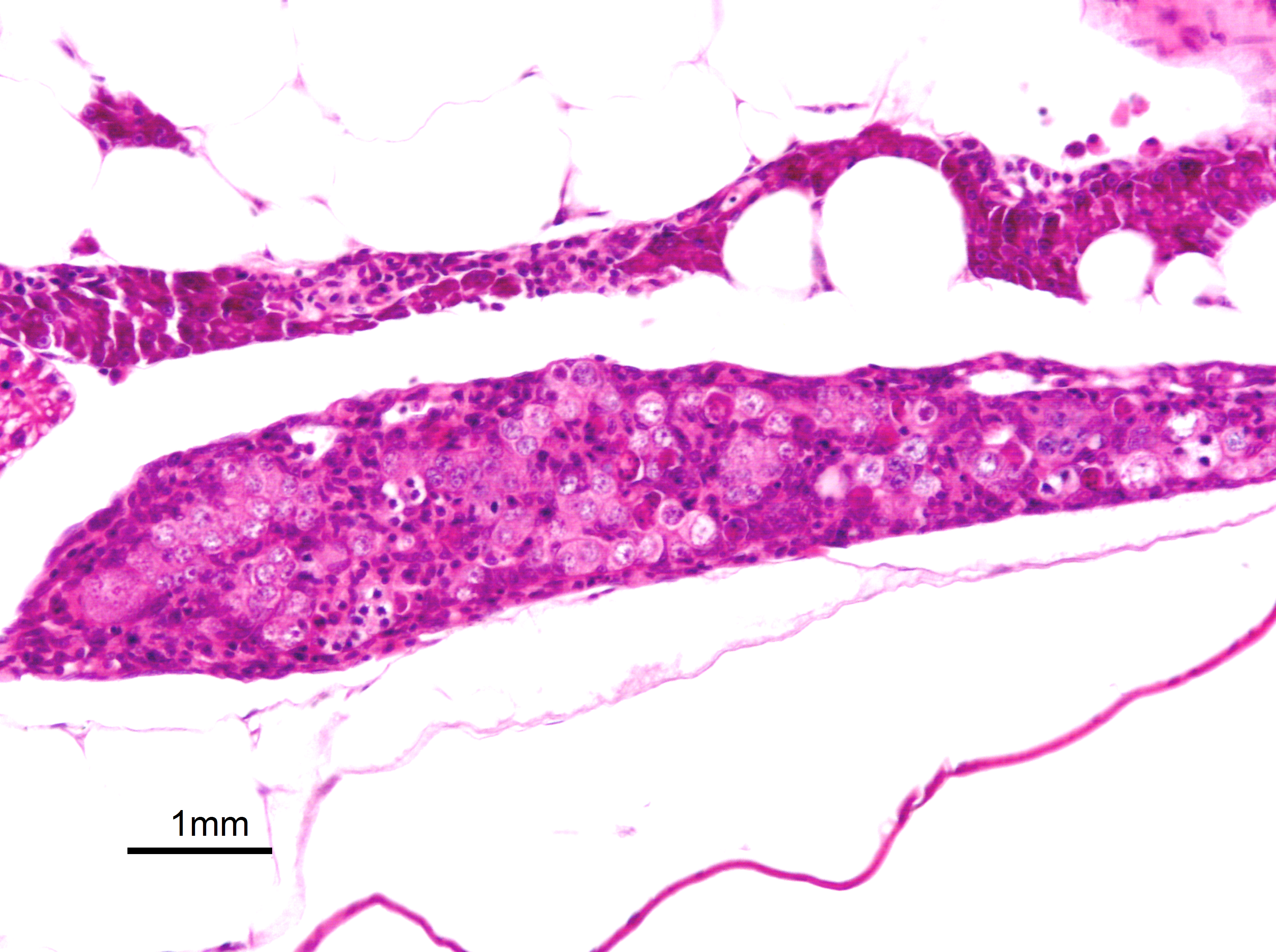

Supplement: Supplementary file 11 — Figure Source Data for Appendix Figures [file 44319_2026_775_MOESM11_ESM.zip › Source Data for Appendix Figure S1 3-7/Appendix Figure S5/Appendix Figure S5B/DMSO WT testis.tif]

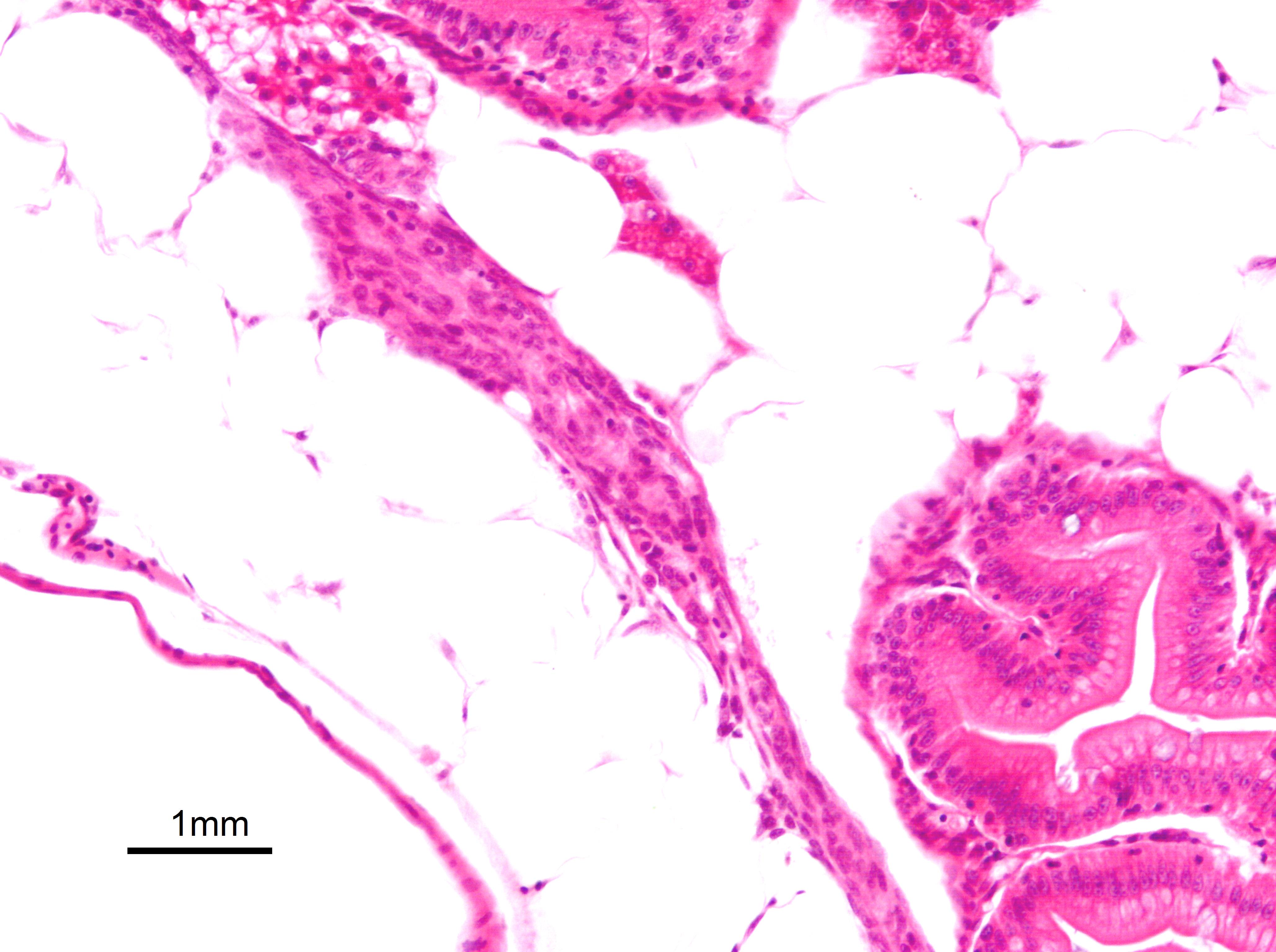

Supplement: Supplementary file 11 — Figure Source Data for Appendix Figures [file 44319_2026_775_MOESM11_ESM.zip › Source Data for Appendix Figure S1 3-7/Appendix Figure S5/Appendix Figure S5B/RHPS4 hom.tif]

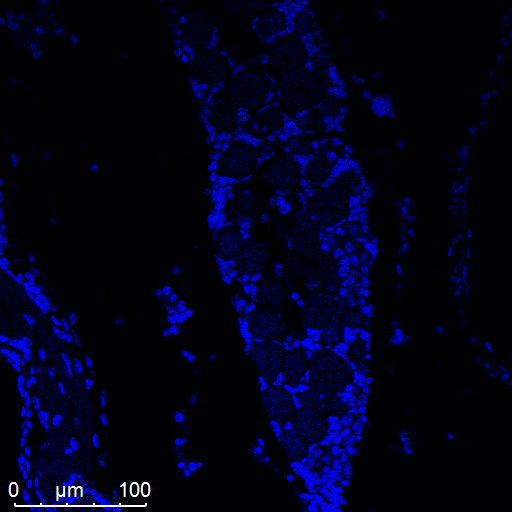

Supplement: Supplementary file 11 — Figure Source Data for Appendix Figures [file 44319_2026_775_MOESM11_ESM.zip › Source Data for Appendix Figure S1 3-7/Appendix Figure S6/Appendix Figure S6D/ddx4/DAPI DMSO.tif]

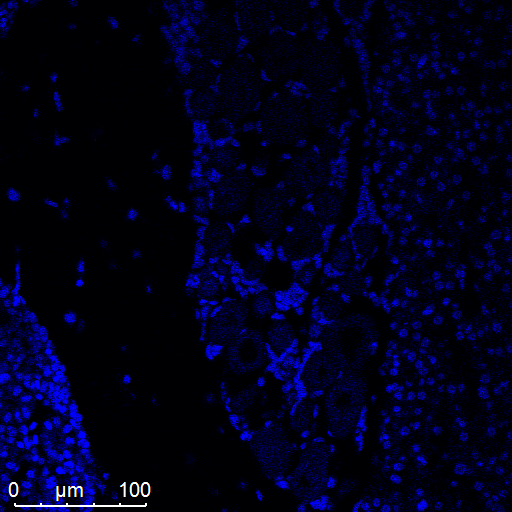

Supplement: Supplementary file 11 — Figure Source Data for Appendix Figures [file 44319_2026_775_MOESM11_ESM.zip › Source Data for Appendix Figure S1 3-7/Appendix Figure S6/Appendix Figure S6D/ddx4/DAPI PNU74654.tif]

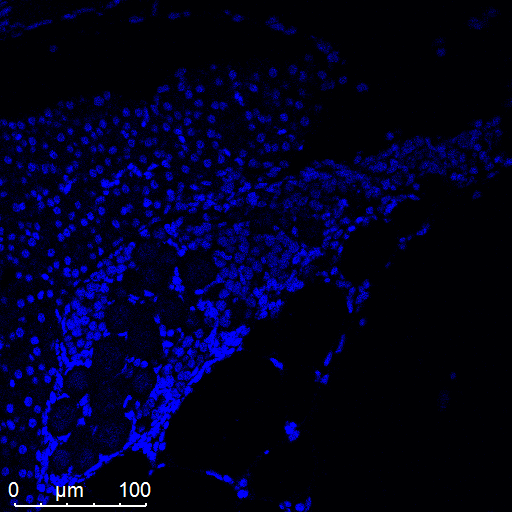

Supplement: Supplementary file 11 — Figure Source Data for Appendix Figures [file 44319_2026_775_MOESM11_ESM.zip › Source Data for Appendix Figure S1 3-7/Appendix Figure S6/Appendix Figure S6D/ddx4/DAPI XAV939.tif]

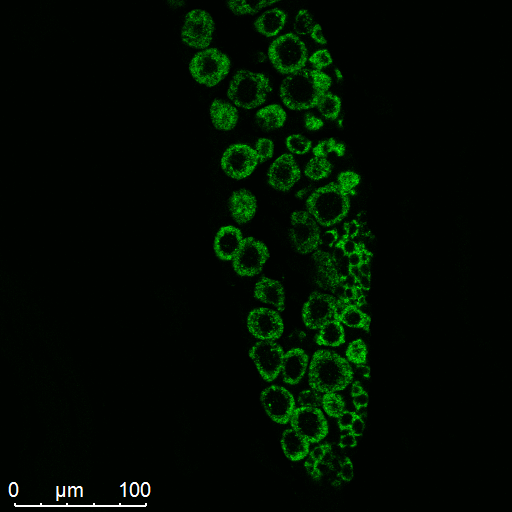

Supplement: Supplementary file 11 — Figure Source Data for Appendix Figures [file 44319_2026_775_MOESM11_ESM.zip › Source Data for Appendix Figure S1 3-7/Appendix Figure S6/Appendix Figure S6D/ddx4/ddx4 DMSO.tif]

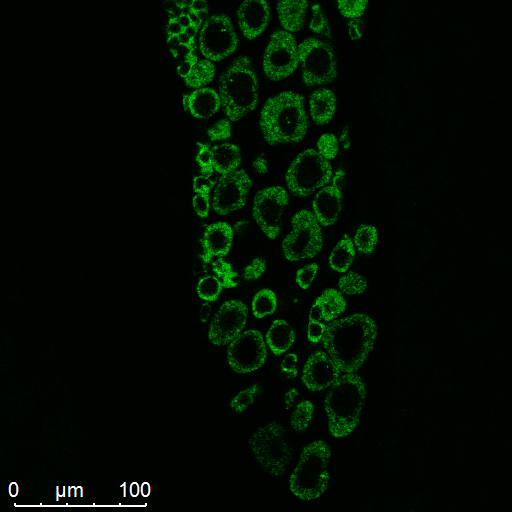

Supplement: Supplementary file 11 — Figure Source Data for Appendix Figures [file 44319_2026_775_MOESM11_ESM.zip › Source Data for Appendix Figure S1 3-7/Appendix Figure S6/Appendix Figure S6D/ddx4/ddx4 PNU74654.tif]

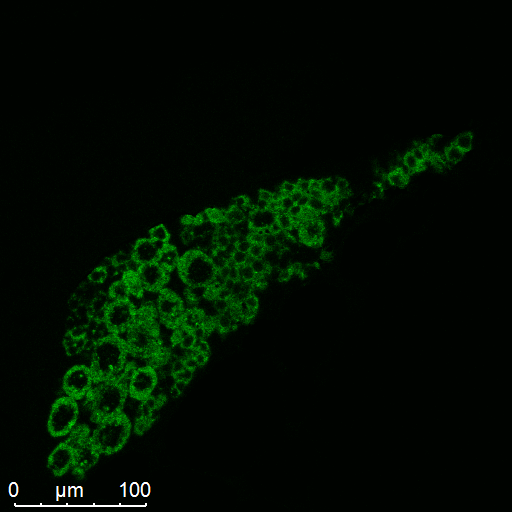

Supplement: Supplementary file 11 — Figure Source Data for Appendix Figures [file 44319_2026_775_MOESM11_ESM.zip › Source Data for Appendix Figure S1 3-7/Appendix Figure S6/Appendix Figure S6D/ddx4/ddx4 XAV939.tif]

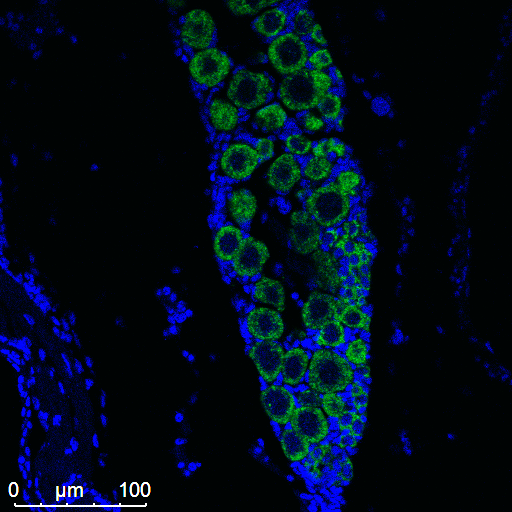

Supplement: Supplementary file 11 — Figure Source Data for Appendix Figures [file 44319_2026_775_MOESM11_ESM.zip › Source Data for Appendix Figure S1 3-7/Appendix Figure S6/Appendix Figure S6D/ddx4/Merge DMSO.tif]

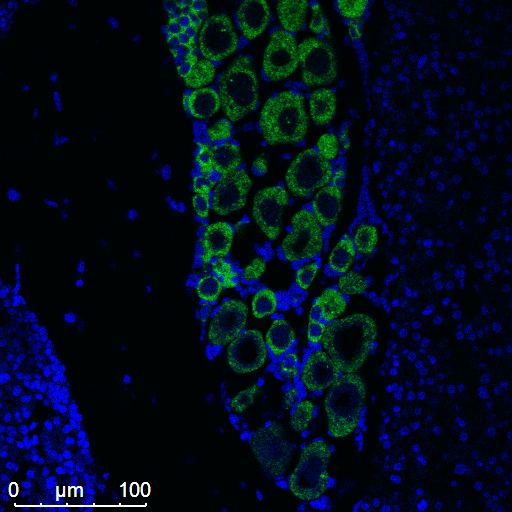

Supplement: Supplementary file 11 — Figure Source Data for Appendix Figures [file 44319_2026_775_MOESM11_ESM.zip › Source Data for Appendix Figure S1 3-7/Appendix Figure S6/Appendix Figure S6D/ddx4/Merge PNU74654.tif]

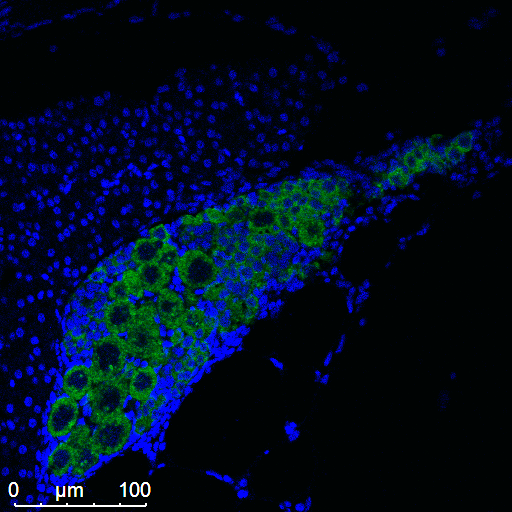

Supplement: Supplementary file 11 — Figure Source Data for Appendix Figures [file 44319_2026_775_MOESM11_ESM.zip › Source Data for Appendix Figure S1 3-7/Appendix Figure S6/Appendix Figure S6D/ddx4/Merge XAV939 .tif]

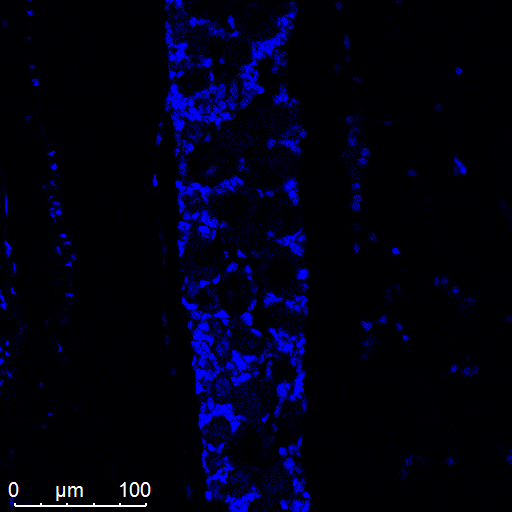

Supplement: Supplementary file 11 — Figure Source Data for Appendix Figures [file 44319_2026_775_MOESM11_ESM.zip › Source Data for Appendix Figure S1 3-7/Appendix Figure S6/Appendix Figure S6D/dnd1/DAPI DMSO.tif]

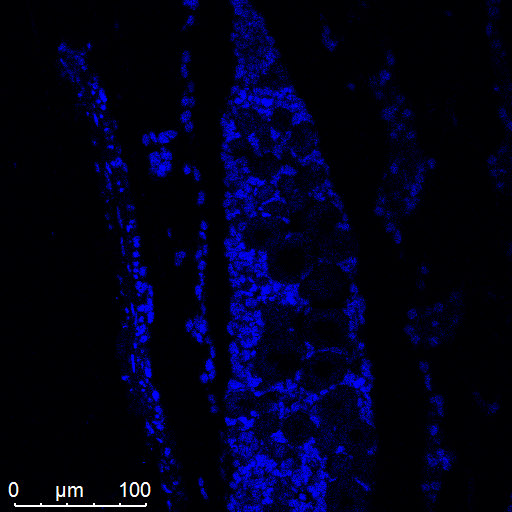

Supplement: Supplementary file 11 — Figure Source Data for Appendix Figures [file 44319_2026_775_MOESM11_ESM.zip › Source Data for Appendix Figure S1 3-7/Appendix Figure S6/Appendix Figure S6D/dnd1/DAPI PNU74654.tif]

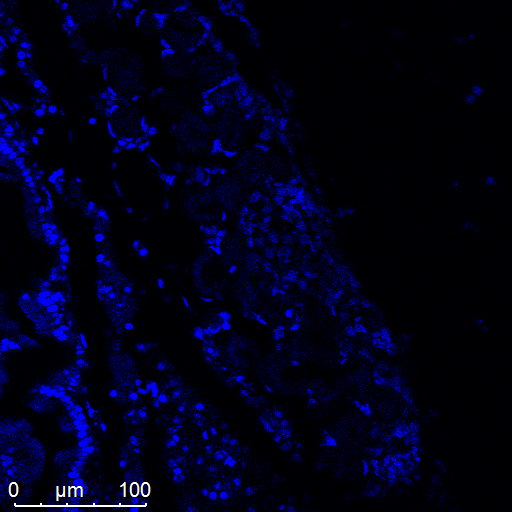

Supplement: Supplementary file 11 — Figure Source Data for Appendix Figures [file 44319_2026_775_MOESM11_ESM.zip › Source Data for Appendix Figure S1 3-7/Appendix Figure S6/Appendix Figure S6D/dnd1/DAPI XAV939.tif]

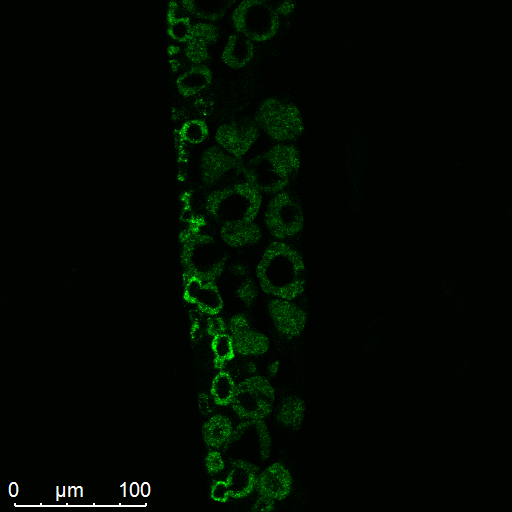

Supplement: Supplementary file 11 — Figure Source Data for Appendix Figures [file 44319_2026_775_MOESM11_ESM.zip › Source Data for Appendix Figure S1 3-7/Appendix Figure S6/Appendix Figure S6D/dnd1/dnd1 DMSO.tif]

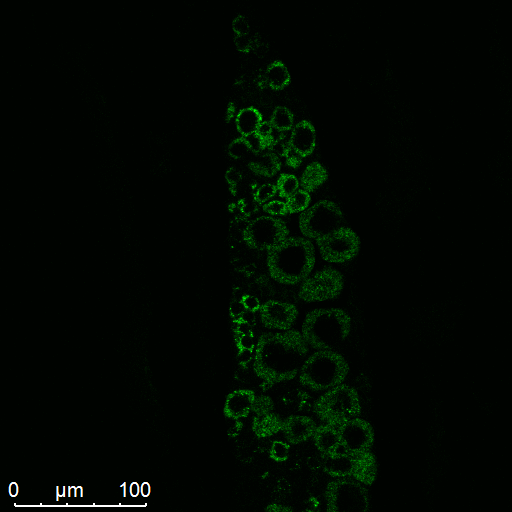

Supplement: Supplementary file 11 — Figure Source Data for Appendix Figures [file 44319_2026_775_MOESM11_ESM.zip › Source Data for Appendix Figure S1 3-7/Appendix Figure S6/Appendix Figure S6D/dnd1/dnd1 PNU74654.tif]

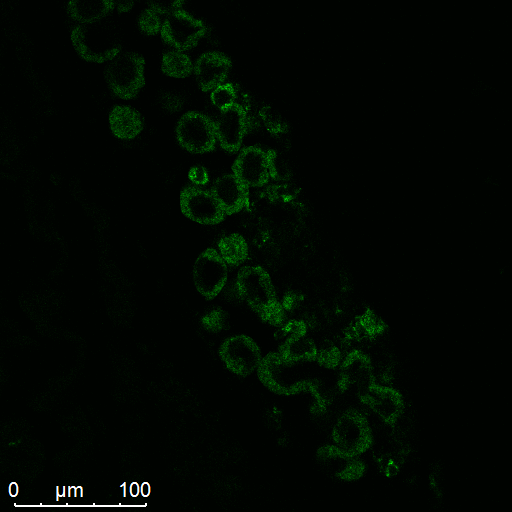

Supplement: Supplementary file 11 — Figure Source Data for Appendix Figures [file 44319_2026_775_MOESM11_ESM.zip › Source Data for Appendix Figure S1 3-7/Appendix Figure S6/Appendix Figure S6D/dnd1/dnd1 XAV939.tif]

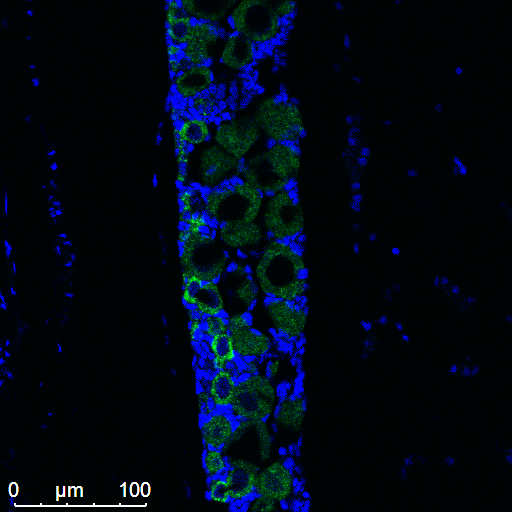

Supplement: Supplementary file 11 — Figure Source Data for Appendix Figures [file 44319_2026_775_MOESM11_ESM.zip › Source Data for Appendix Figure S1 3-7/Appendix Figure S6/Appendix Figure S6D/dnd1/Merge DMSO.tif]

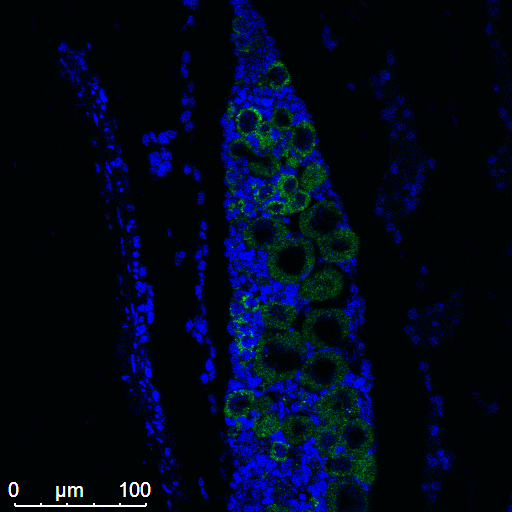

Supplement: Supplementary file 11 — Figure Source Data for Appendix Figures [file 44319_2026_775_MOESM11_ESM.zip › Source Data for Appendix Figure S1 3-7/Appendix Figure S6/Appendix Figure S6D/dnd1/Merge PNU74654.tif]

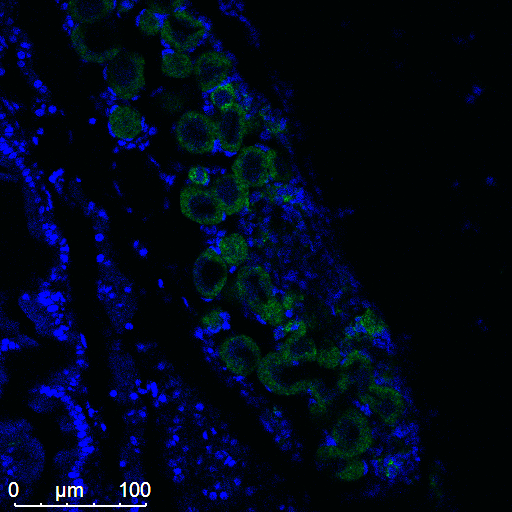

Supplement: Supplementary file 11 — Figure Source Data for Appendix Figures [file 44319_2026_775_MOESM11_ESM.zip › Source Data for Appendix Figure S1 3-7/Appendix Figure S6/Appendix Figure S6D/dnd1/Merge XAV939.tif]

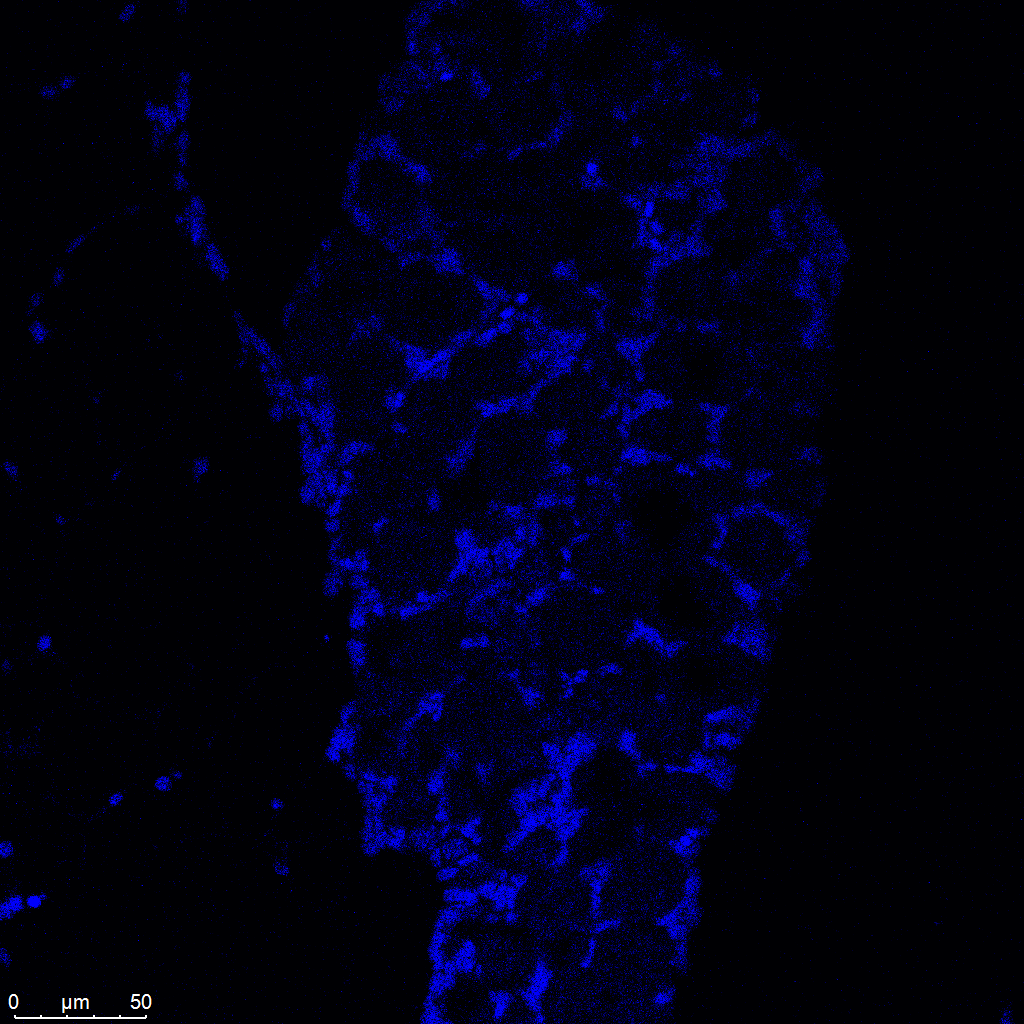

Supplement: Supplementary file 11 — Figure Source Data for Appendix Figures [file 44319_2026_775_MOESM11_ESM.zip › Source Data for Appendix Figure S1 3-7/Appendix Figure S6/Appendix Figure S6D/gfp/DAPI DMSO.tif]

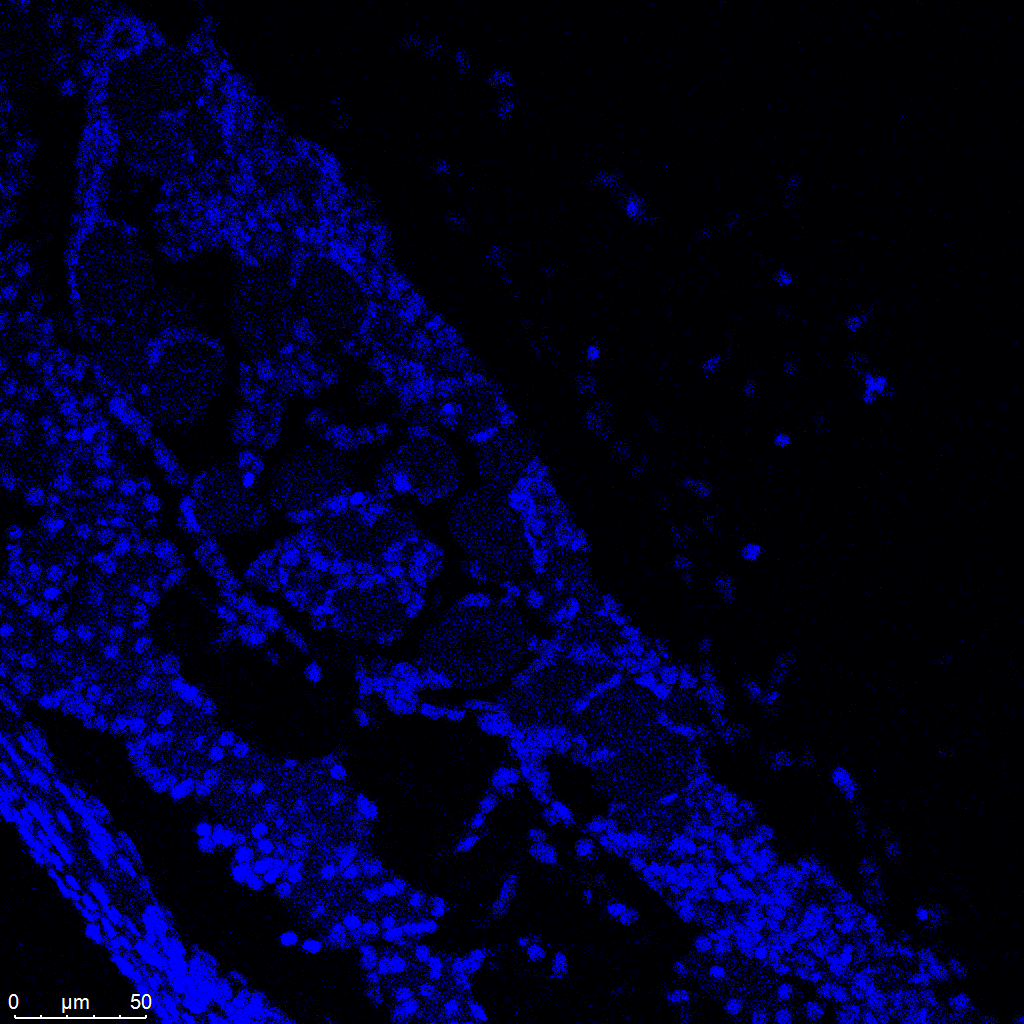

Supplement: Supplementary file 11 — Figure Source Data for Appendix Figures [file 44319_2026_775_MOESM11_ESM.zip › Source Data for Appendix Figure S1 3-7/Appendix Figure S6/Appendix Figure S6D/gfp/DAPI PNU74654.tif]

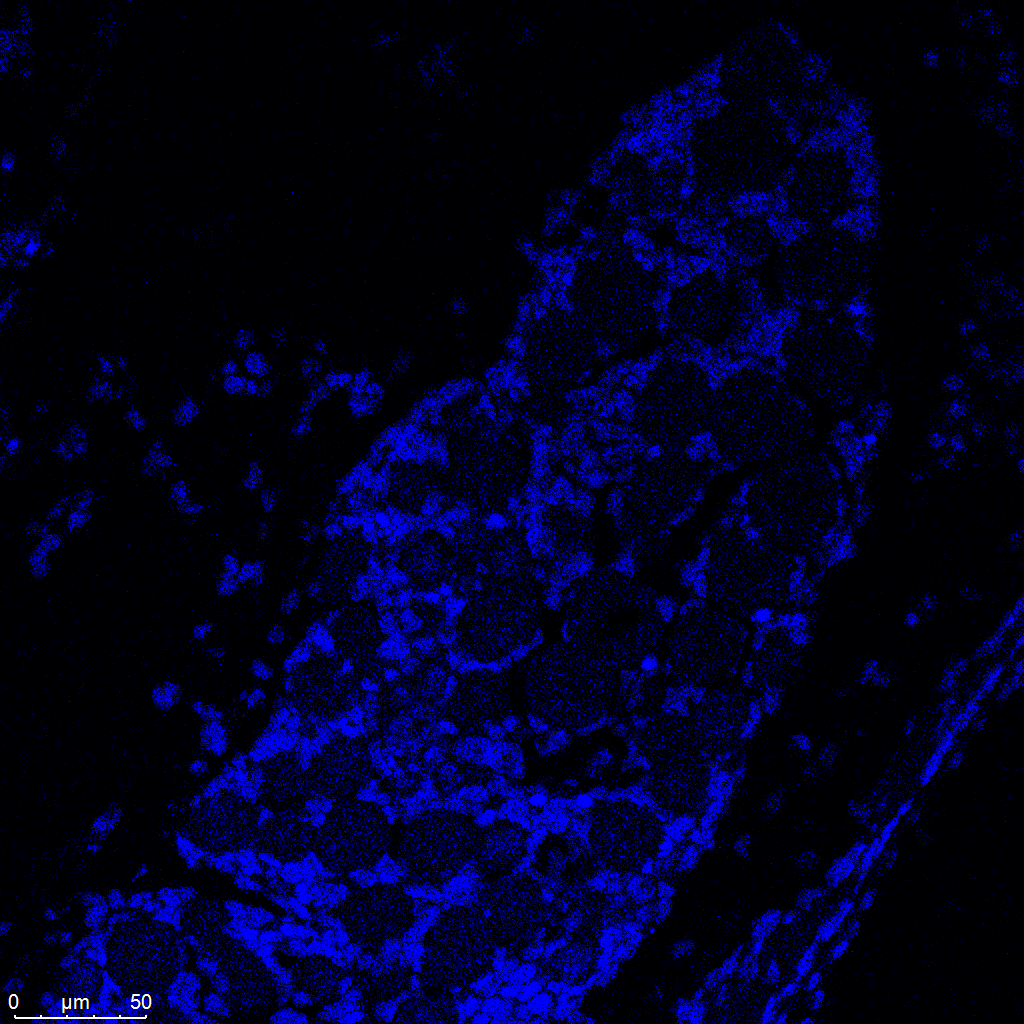

Supplement: Supplementary file 11 — Figure Source Data for Appendix Figures [file 44319_2026_775_MOESM11_ESM.zip › Source Data for Appendix Figure S1 3-7/Appendix Figure S6/Appendix Figure S6D/gfp/DAPI XAV939.tif]

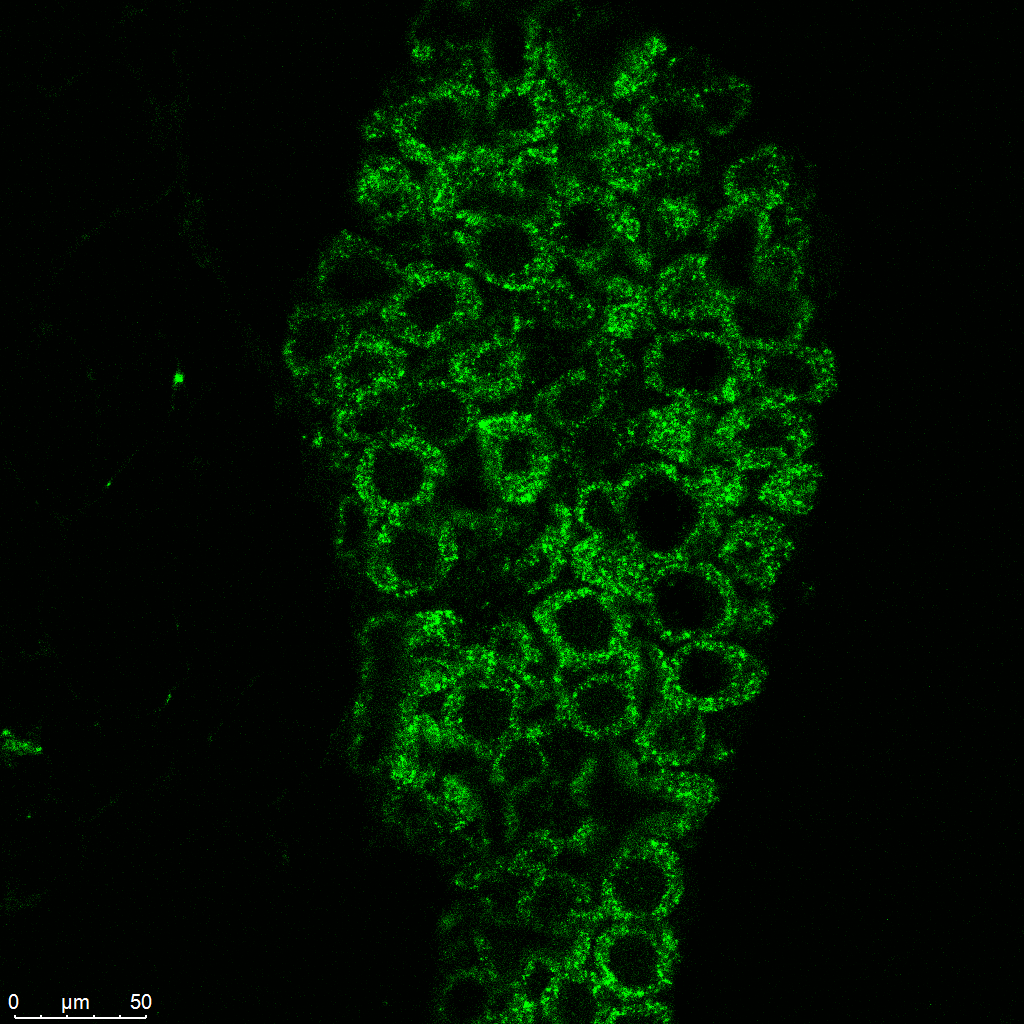

Supplement: Supplementary file 11 — Figure Source Data for Appendix Figures [file 44319_2026_775_MOESM11_ESM.zip › Source Data for Appendix Figure S1 3-7/Appendix Figure S6/Appendix Figure S6D/gfp/gfp DMSO.tif]

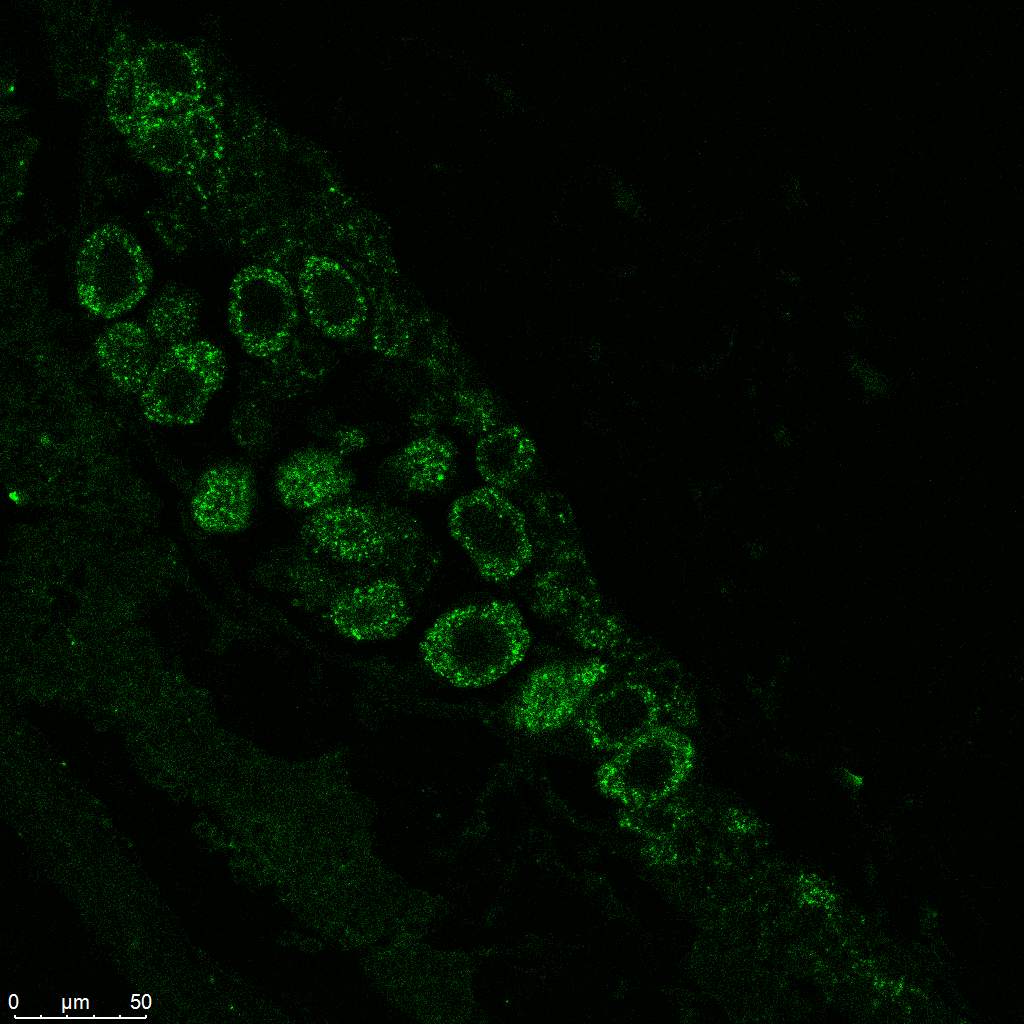

Supplement: Supplementary file 11 — Figure Source Data for Appendix Figures [file 44319_2026_775_MOESM11_ESM.zip › Source Data for Appendix Figure S1 3-7/Appendix Figure S6/Appendix Figure S6D/gfp/gfp PNU74654.tif]

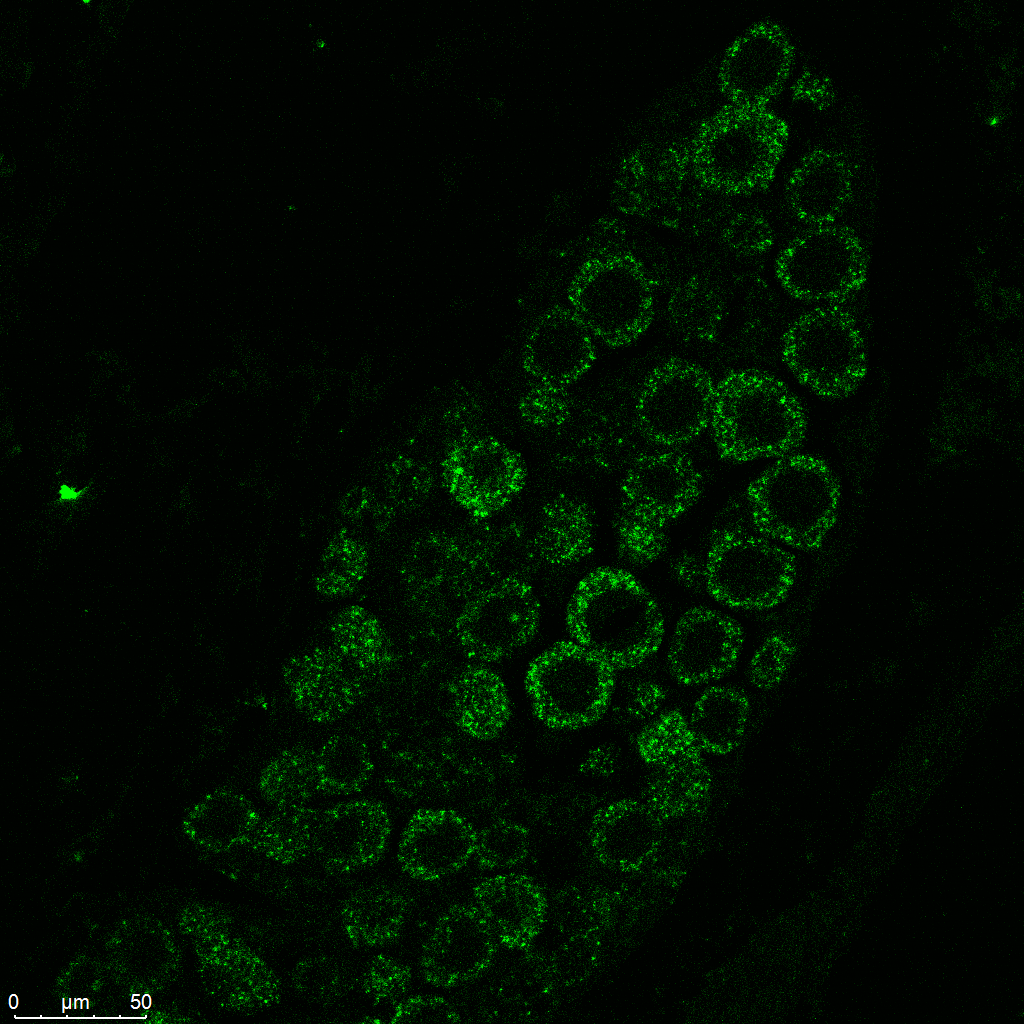

Supplement: Supplementary file 11 — Figure Source Data for Appendix Figures [file 44319_2026_775_MOESM11_ESM.zip › Source Data for Appendix Figure S1 3-7/Appendix Figure S6/Appendix Figure S6D/gfp/gfp XAV939.tif]

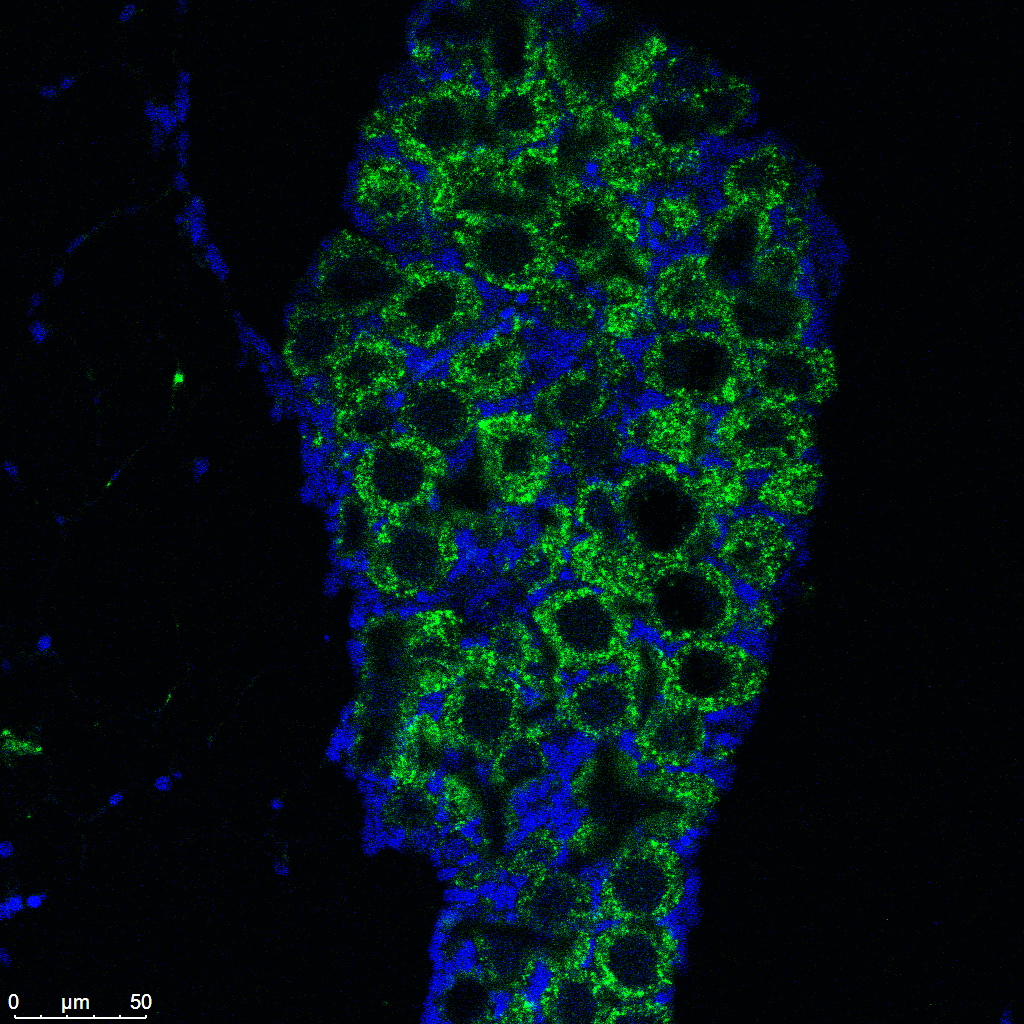

Supplement: Supplementary file 11 — Figure Source Data for Appendix Figures [file 44319_2026_775_MOESM11_ESM.zip › Source Data for Appendix Figure S1 3-7/Appendix Figure S6/Appendix Figure S6D/gfp/Merge DMSO.tif]

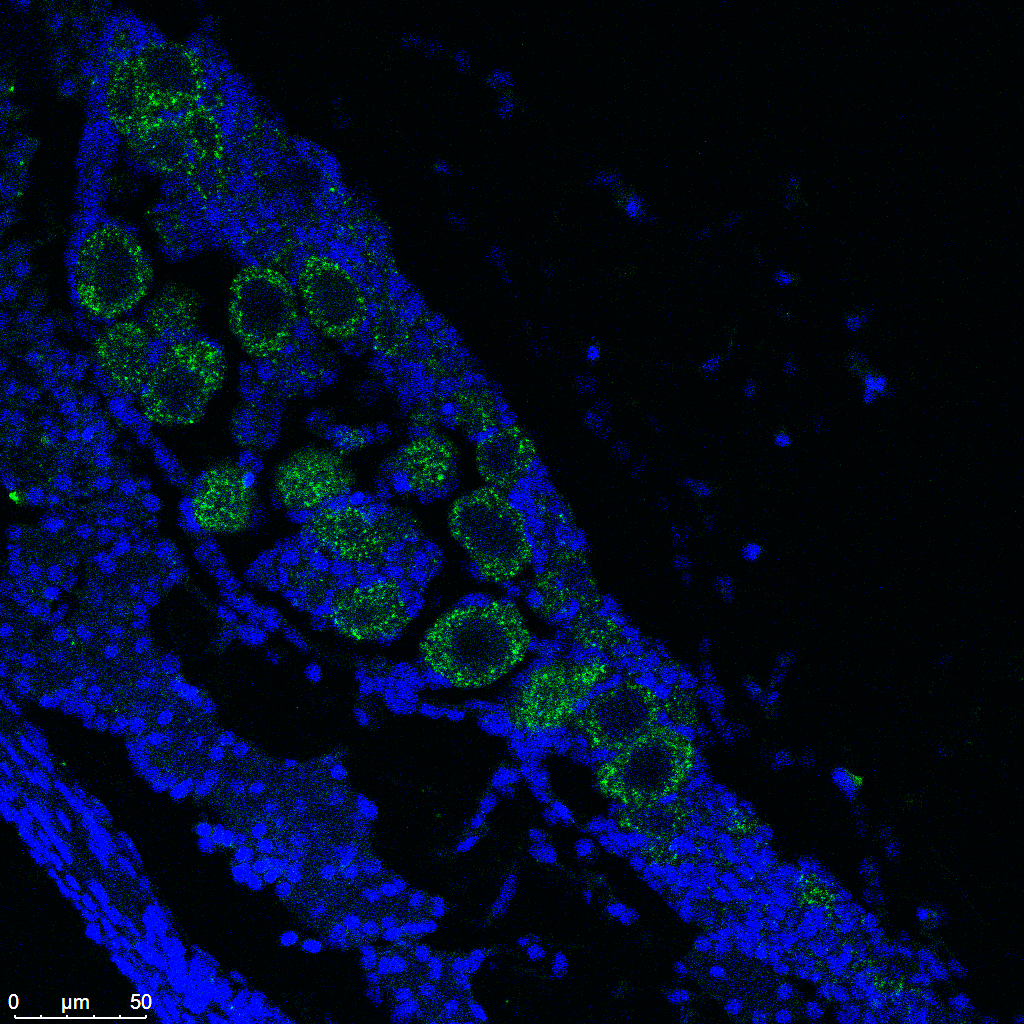

Supplement: Supplementary file 11 — Figure Source Data for Appendix Figures [file 44319_2026_775_MOESM11_ESM.zip › Source Data for Appendix Figure S1 3-7/Appendix Figure S6/Appendix Figure S6D/gfp/Merge PNU74654.tif]

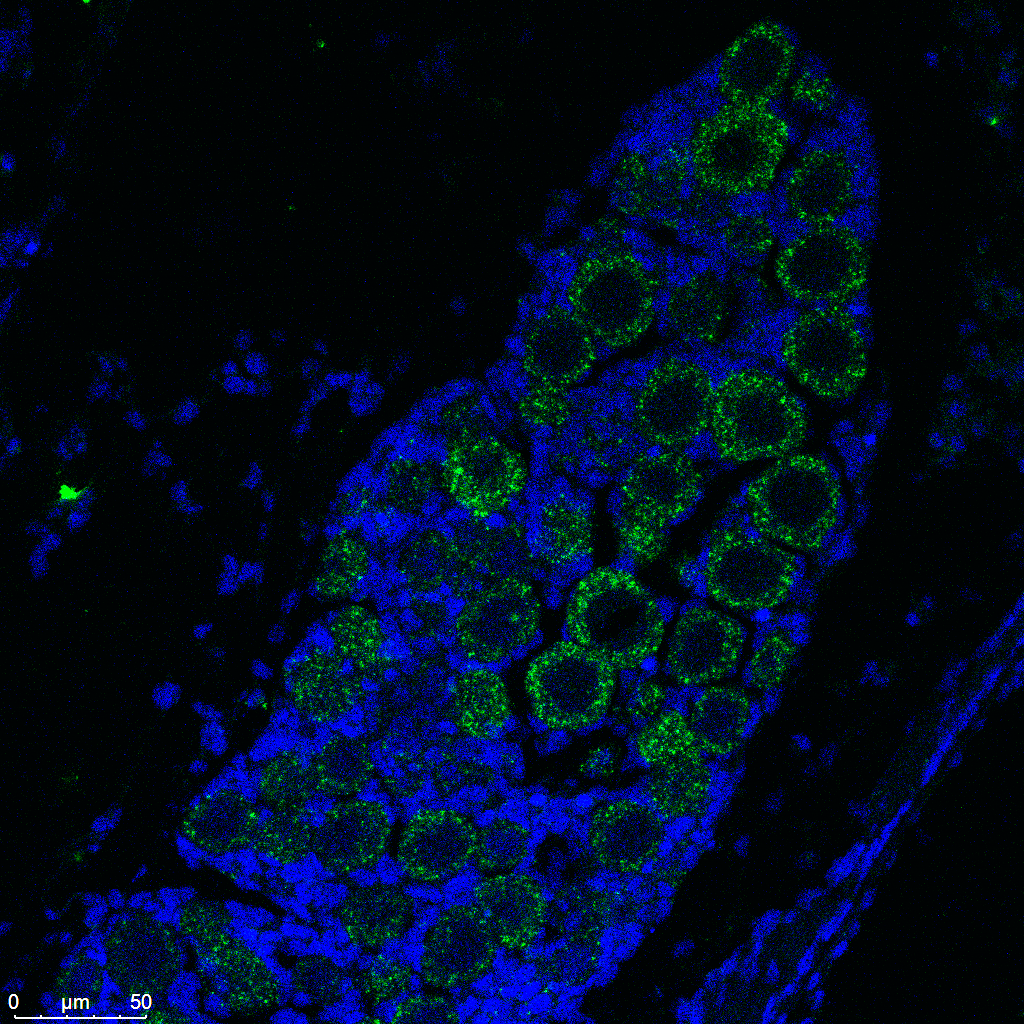

Supplement: Supplementary file 11 — Figure Source Data for Appendix Figures [file 44319_2026_775_MOESM11_ESM.zip › Source Data for Appendix Figure S1 3-7/Appendix Figure S6/Appendix Figure S6D/gfp/Merge XAV939.tif]

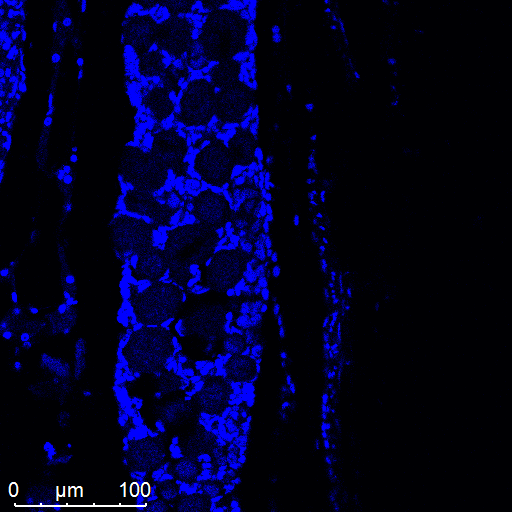

Supplement: Supplementary file 11 — Figure Source Data for Appendix Figures [file 44319_2026_775_MOESM11_ESM.zip › Source Data for Appendix Figure S1 3-7/Appendix Figure S6/Appendix Figure S6D/piwil1/DAPI PNU74654.tif]

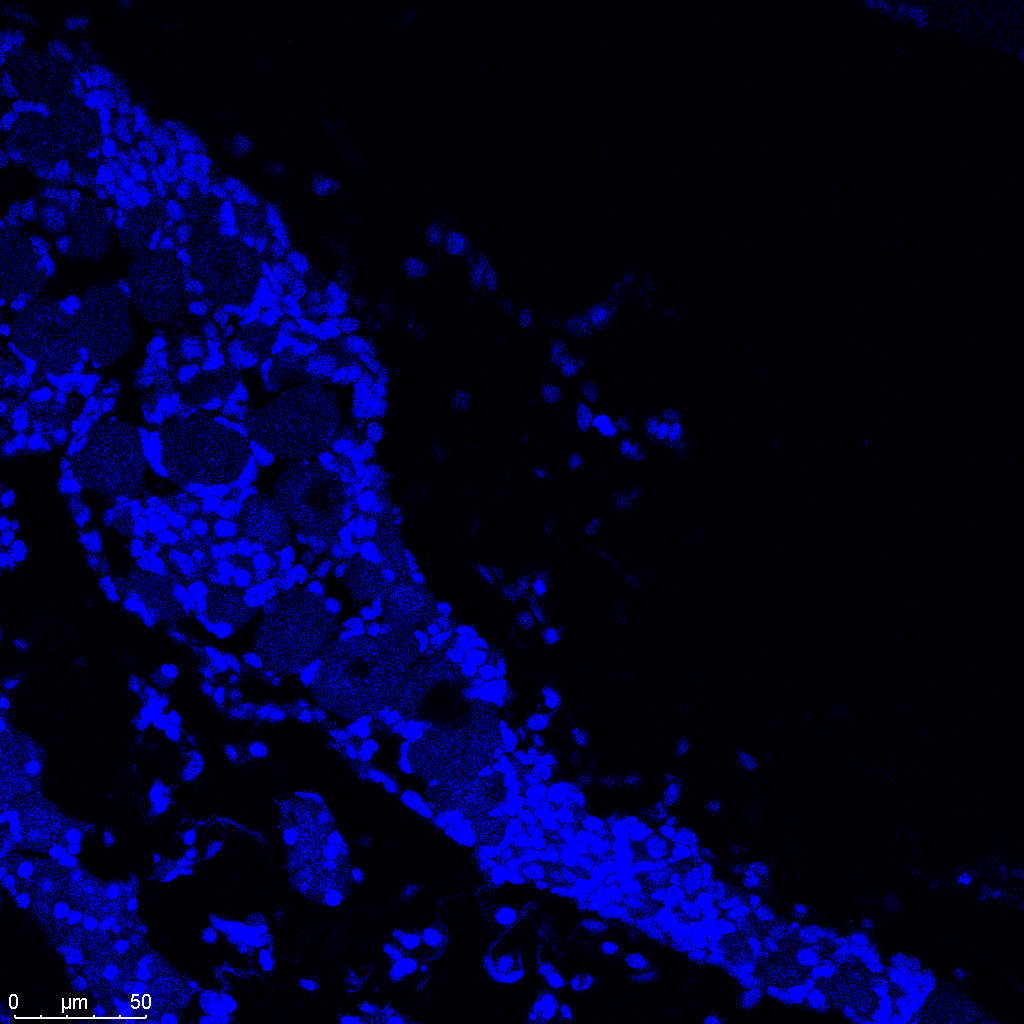

Supplement: Supplementary file 11 — Figure Source Data for Appendix Figures [file 44319_2026_775_MOESM11_ESM.zip › Source Data for Appendix Figure S1 3-7/Appendix Figure S6/Appendix Figure S6D/piwil1/DAPI XAV939.tif]

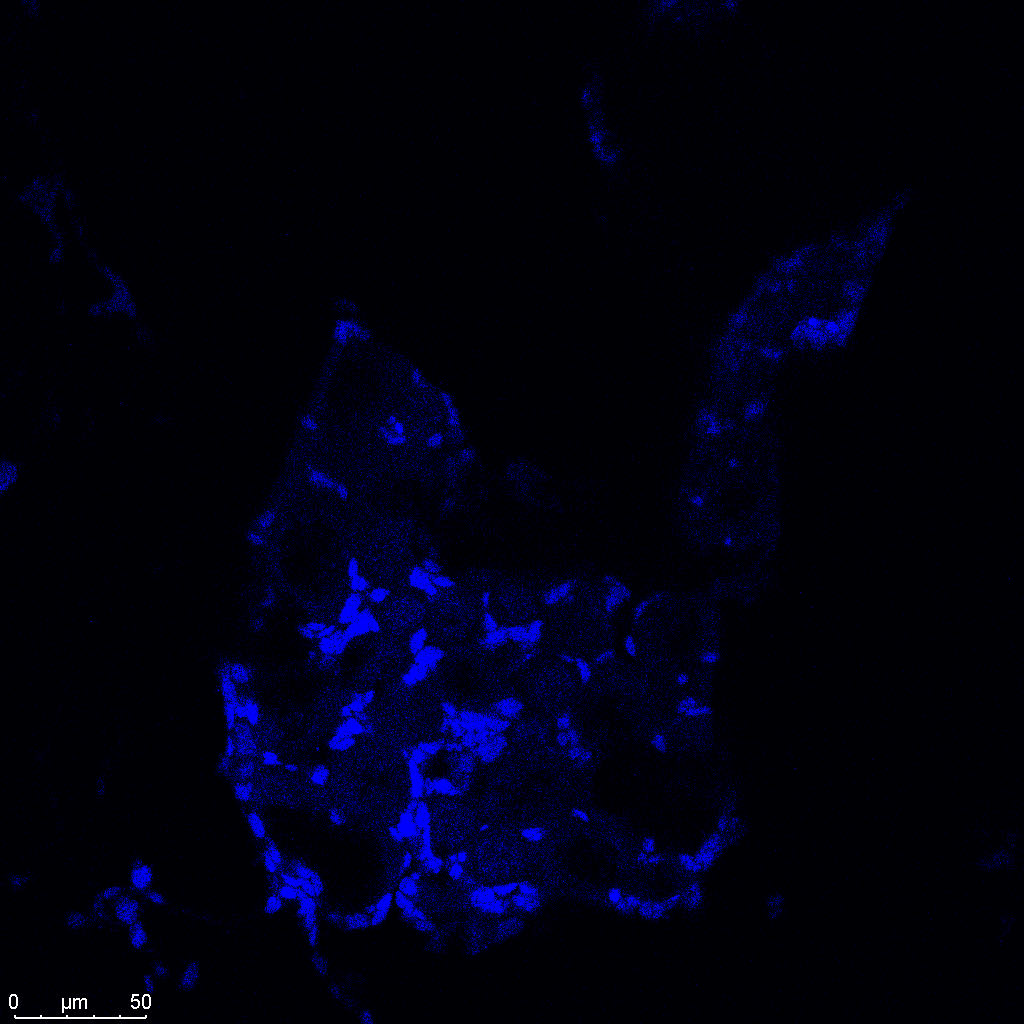

Supplement: Supplementary file 11 — Figure Source Data for Appendix Figures [file 44319_2026_775_MOESM11_ESM.zip › Source Data for Appendix Figure S1 3-7/Appendix Figure S6/Appendix Figure S6D/piwil1/DPAI DMSO.tif]

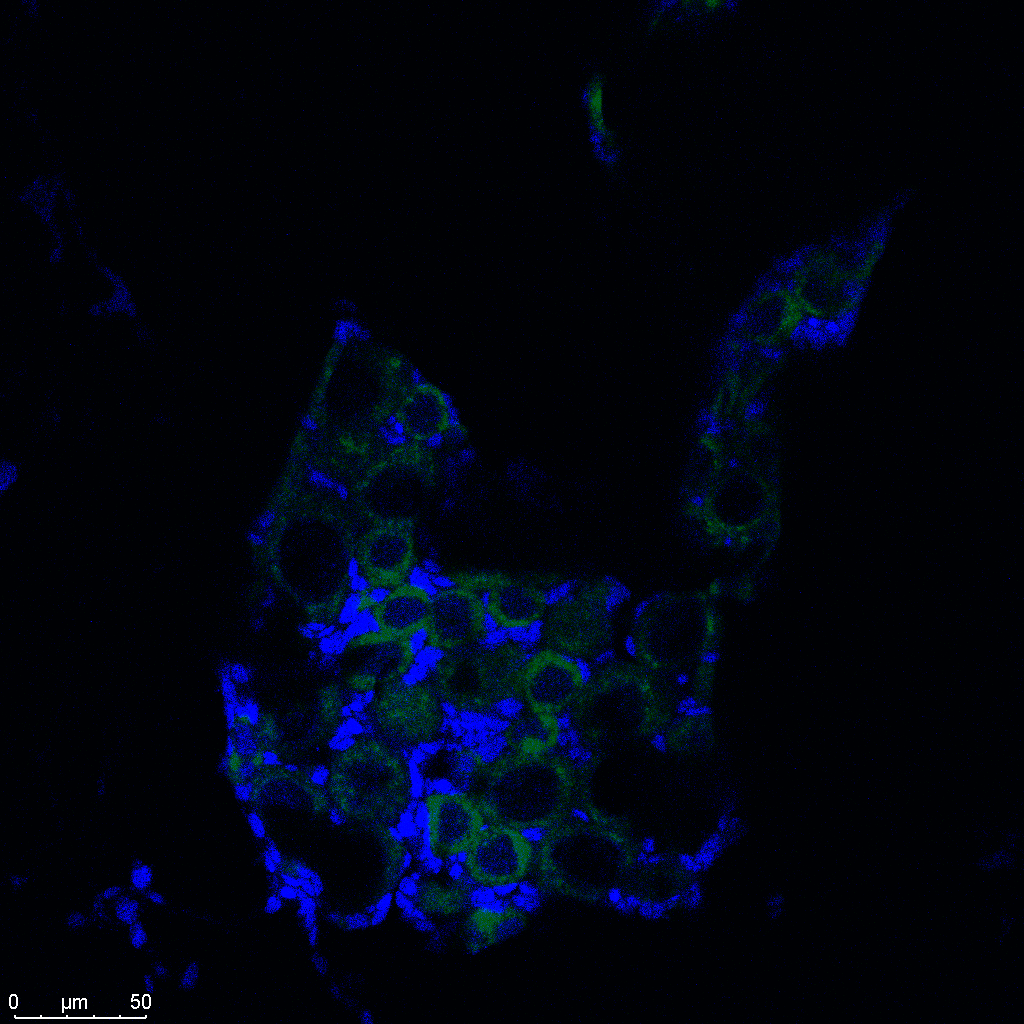

Supplement: Supplementary file 11 — Figure Source Data for Appendix Figures [file 44319_2026_775_MOESM11_ESM.zip › Source Data for Appendix Figure S1 3-7/Appendix Figure S6/Appendix Figure S6D/piwil1/Merge DMSO.tif]

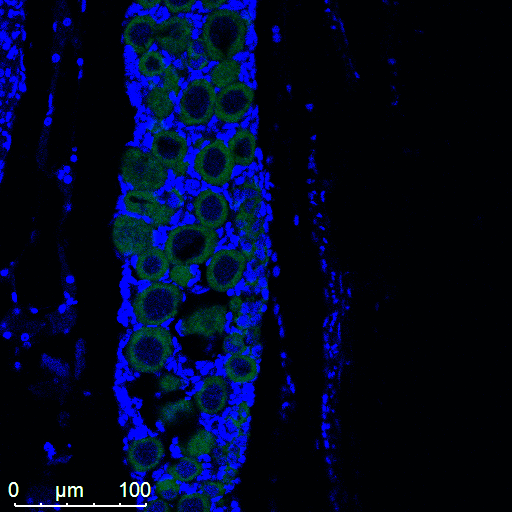

Supplement: Supplementary file 11 — Figure Source Data for Appendix Figures [file 44319_2026_775_MOESM11_ESM.zip › Source Data for Appendix Figure S1 3-7/Appendix Figure S6/Appendix Figure S6D/piwil1/Merge PNU74654.tif]

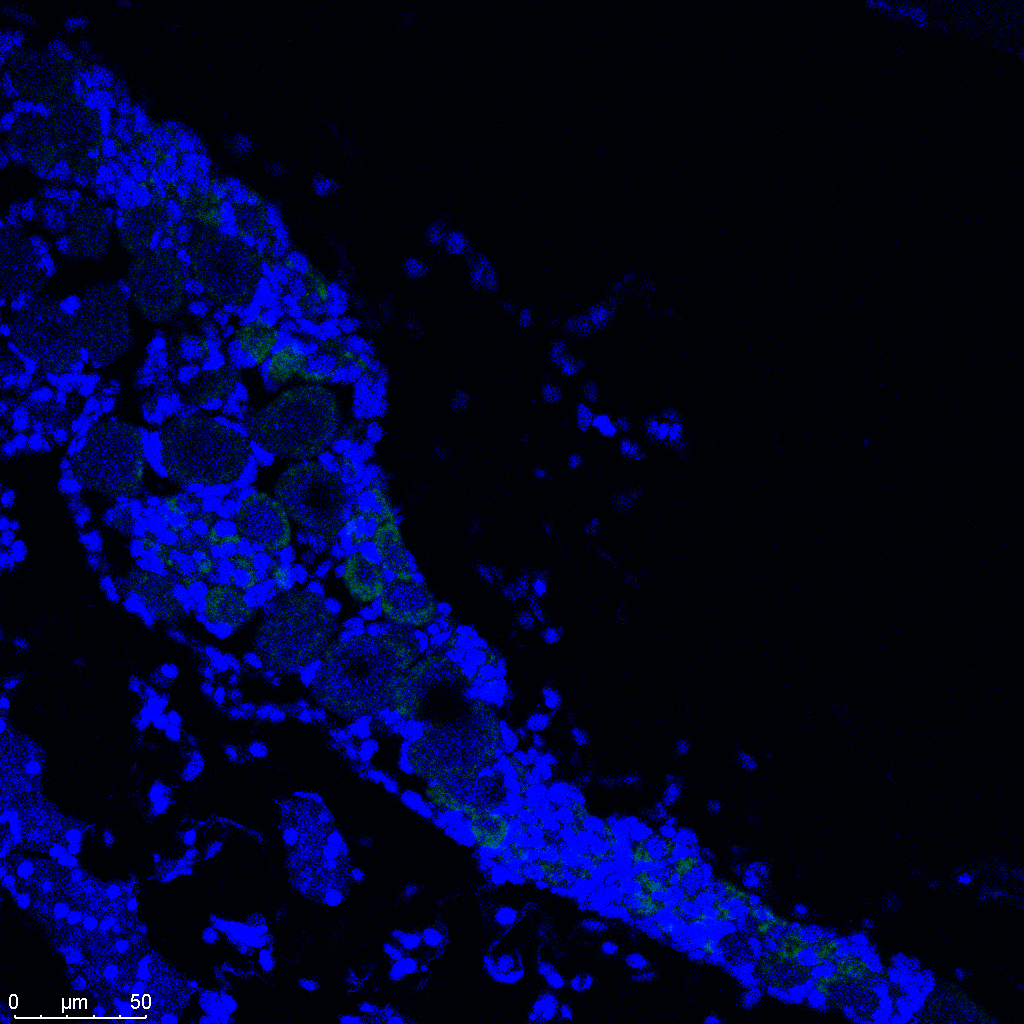

Supplement: Supplementary file 11 — Figure Source Data for Appendix Figures [file 44319_2026_775_MOESM11_ESM.zip › Source Data for Appendix Figure S1 3-7/Appendix Figure S6/Appendix Figure S6D/piwil1/Merge XAV939.tif]

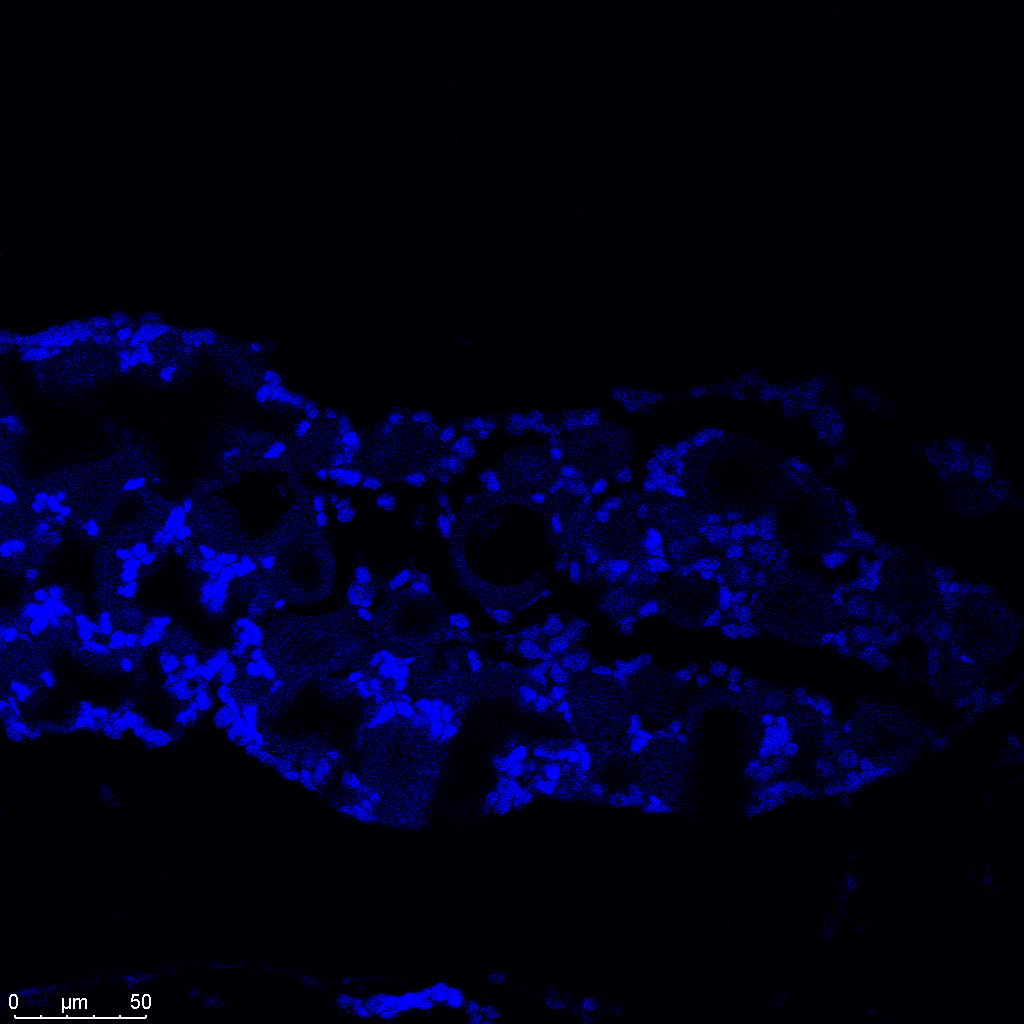

Supplement: Supplementary file 11 — Figure Source Data for Appendix Figures [file 44319_2026_775_MOESM11_ESM.zip › Source Data for Appendix Figure S1 3-7/Appendix Figure S6/Appendix Figure S6D/piwil2/DAPI DMSO.tif]

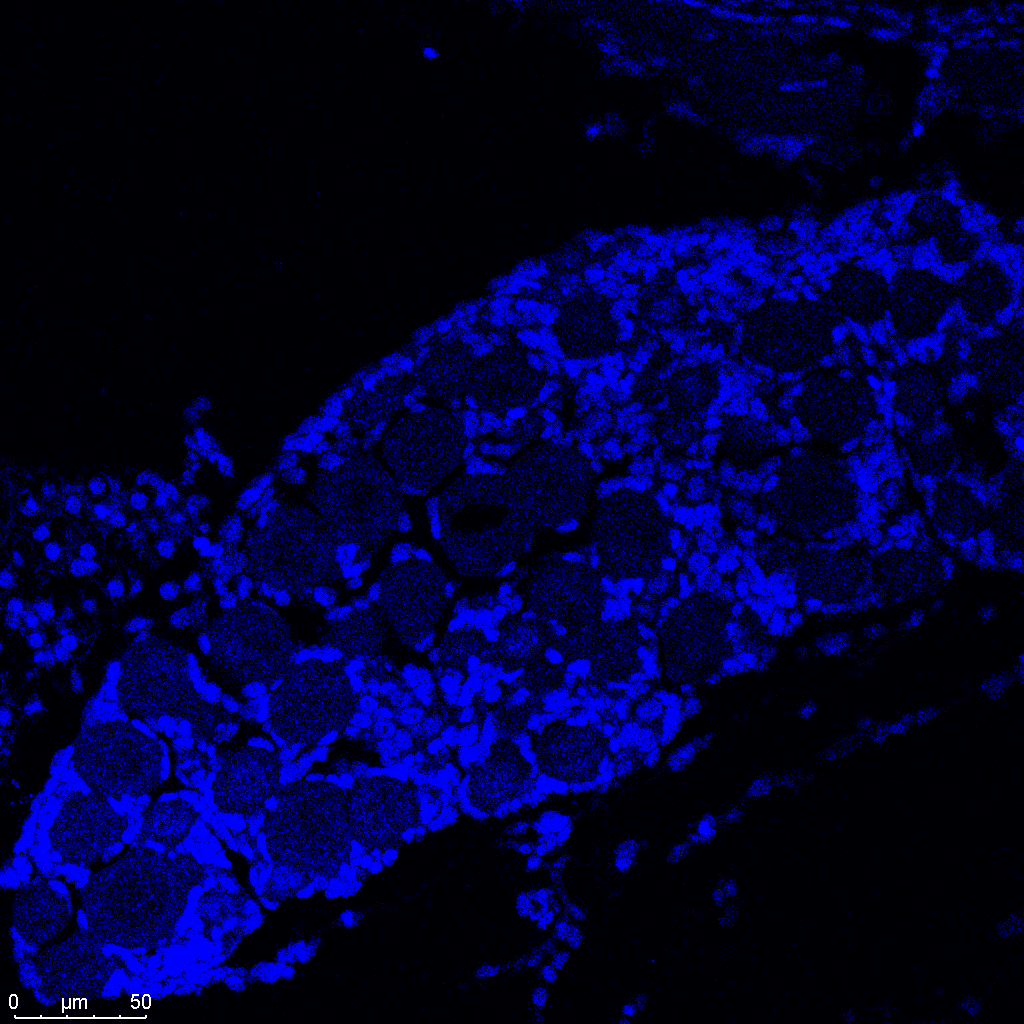

Supplement: Supplementary file 11 — Figure Source Data for Appendix Figures [file 44319_2026_775_MOESM11_ESM.zip › Source Data for Appendix Figure S1 3-7/Appendix Figure S6/Appendix Figure S6D/piwil2/DAPI PNU74654.tif]

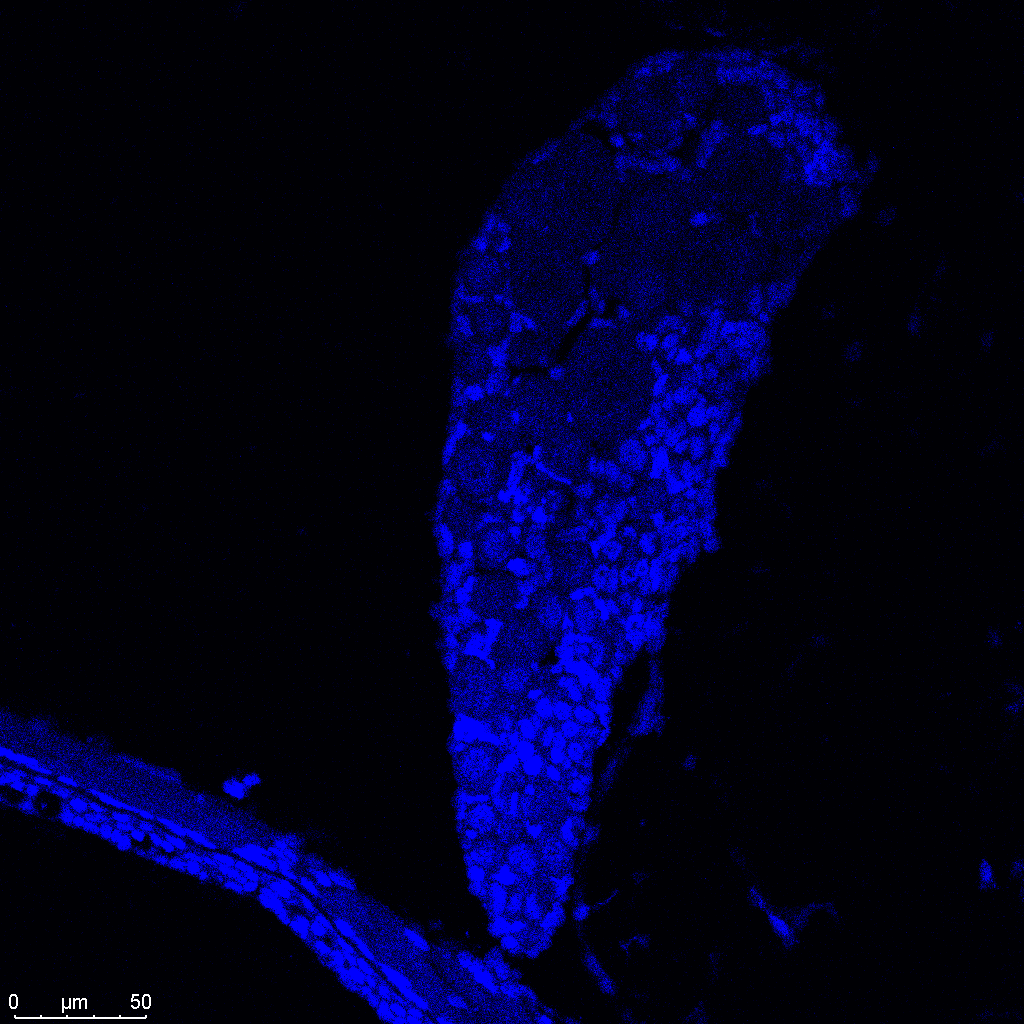

Supplement: Supplementary file 11 — Figure Source Data for Appendix Figures [file 44319_2026_775_MOESM11_ESM.zip › Source Data for Appendix Figure S1 3-7/Appendix Figure S6/Appendix Figure S6D/piwil2/DAPI XAV939.tif]

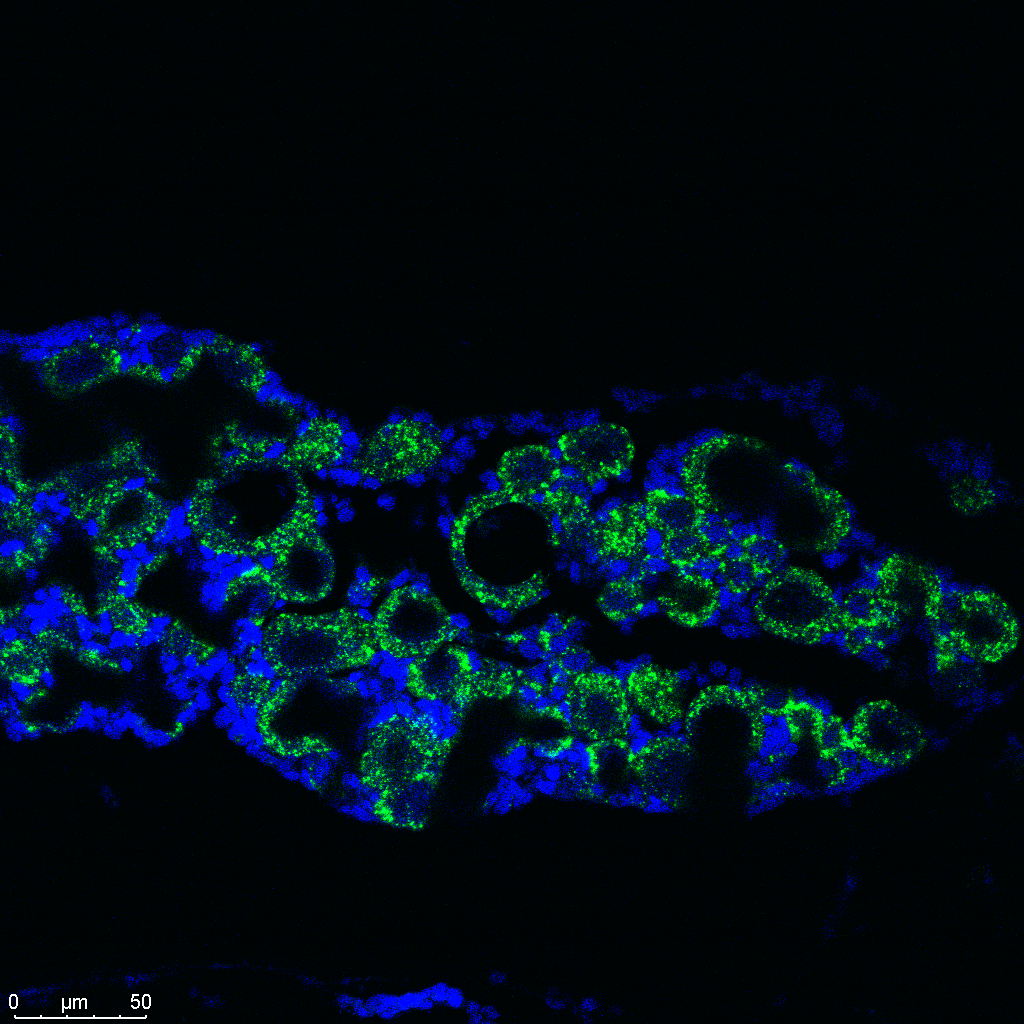

Supplement: Supplementary file 11 — Figure Source Data for Appendix Figures [file 44319_2026_775_MOESM11_ESM.zip › Source Data for Appendix Figure S1 3-7/Appendix Figure S6/Appendix Figure S6D/piwil2/Merge DMSO.tif]

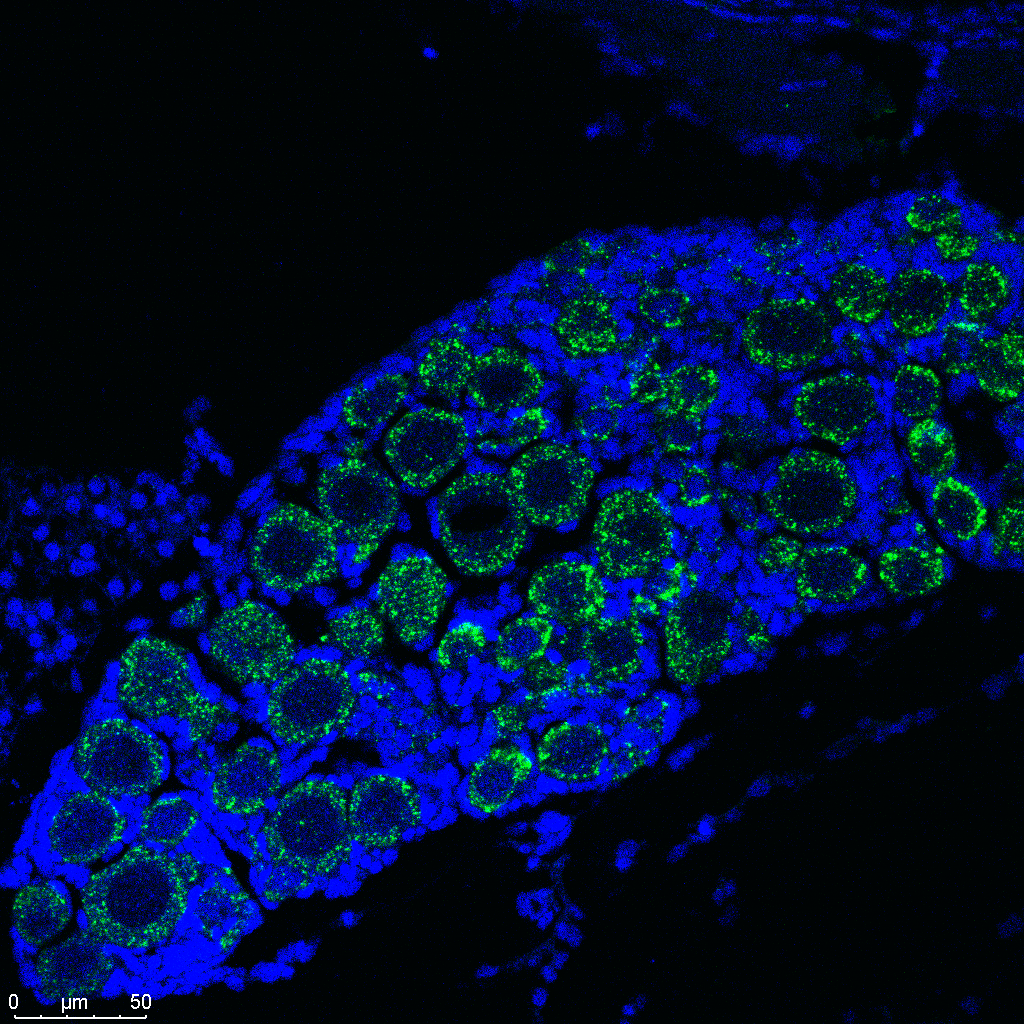

Supplement: Supplementary file 11 — Figure Source Data for Appendix Figures [file 44319_2026_775_MOESM11_ESM.zip › Source Data for Appendix Figure S1 3-7/Appendix Figure S6/Appendix Figure S6D/piwil2/Merge PNU74654.tif]

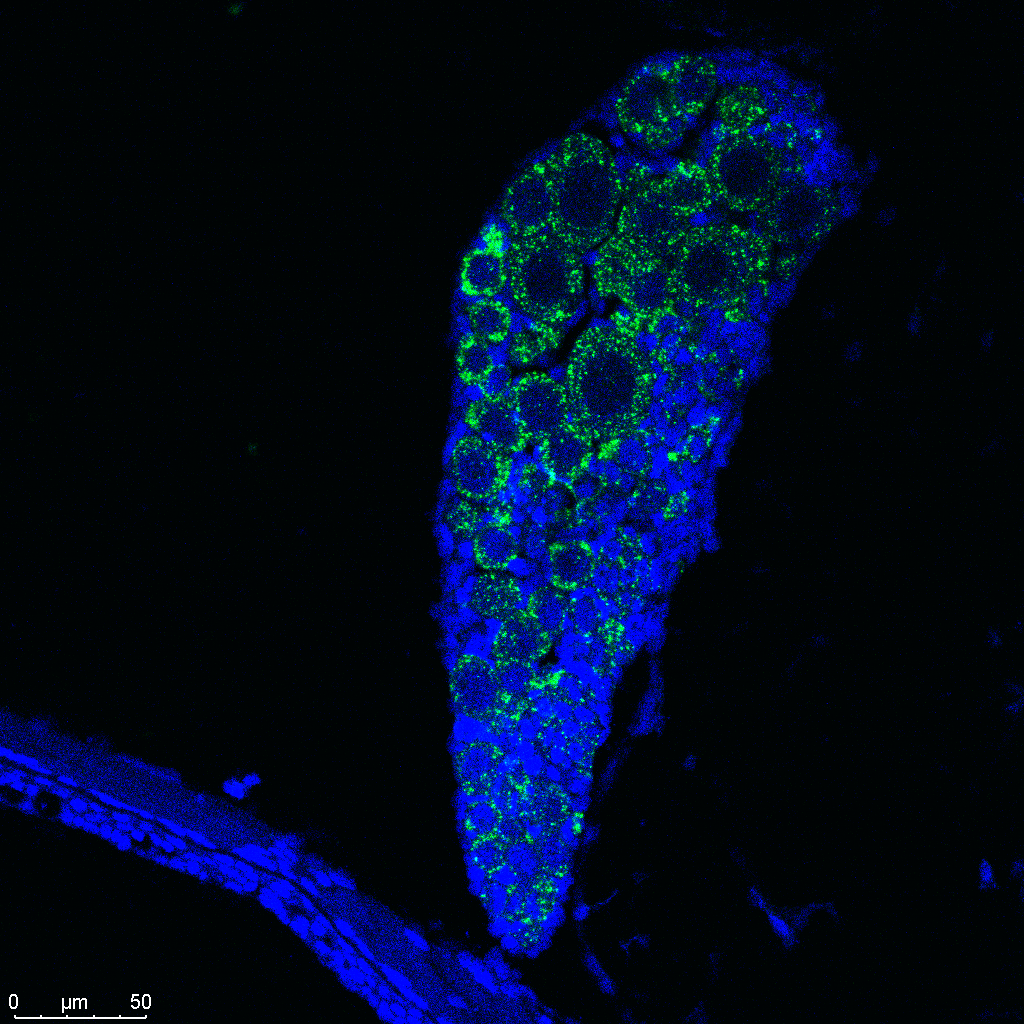

Supplement: Supplementary file 11 — Figure Source Data for Appendix Figures [file 44319_2026_775_MOESM11_ESM.zip › Source Data for Appendix Figure S1 3-7/Appendix Figure S6/Appendix Figure S6D/piwil2/Merge XAV939.tif]

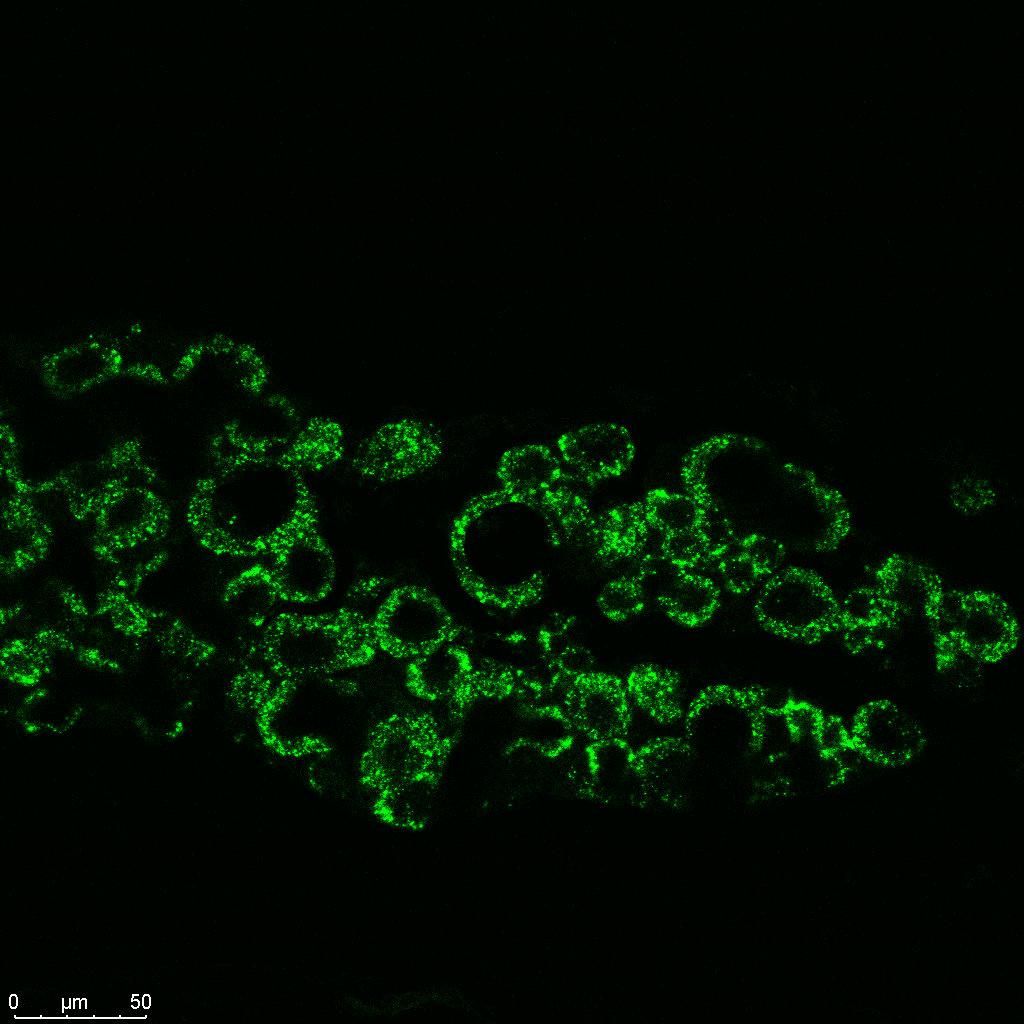

Supplement: Supplementary file 11 — Figure Source Data for Appendix Figures [file 44319_2026_775_MOESM11_ESM.zip › Source Data for Appendix Figure S1 3-7/Appendix Figure S6/Appendix Figure S6D/piwil2/piwil2 DMSO.tif]

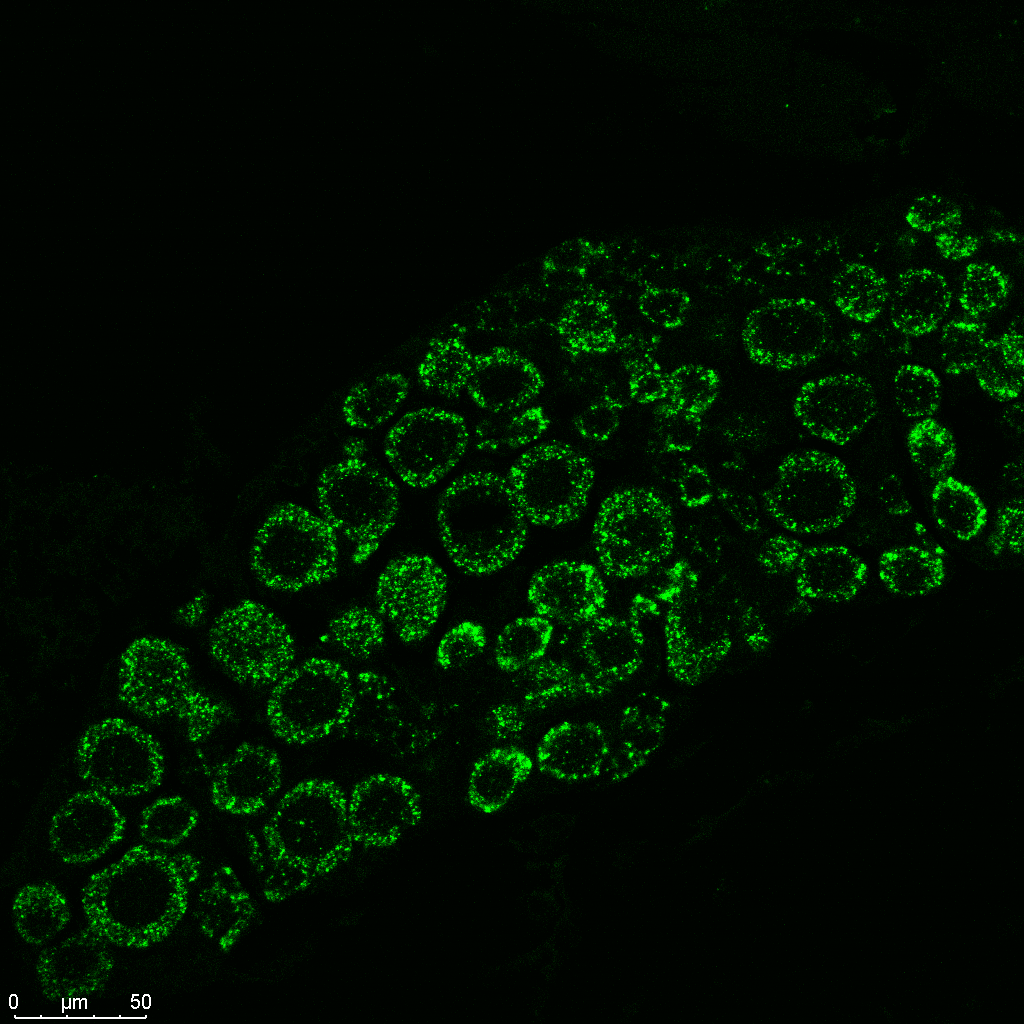

Supplement: Supplementary file 11 — Figure Source Data for Appendix Figures [file 44319_2026_775_MOESM11_ESM.zip › Source Data for Appendix Figure S1 3-7/Appendix Figure S6/Appendix Figure S6D/piwil2/piwil2 PNU74654.tif]

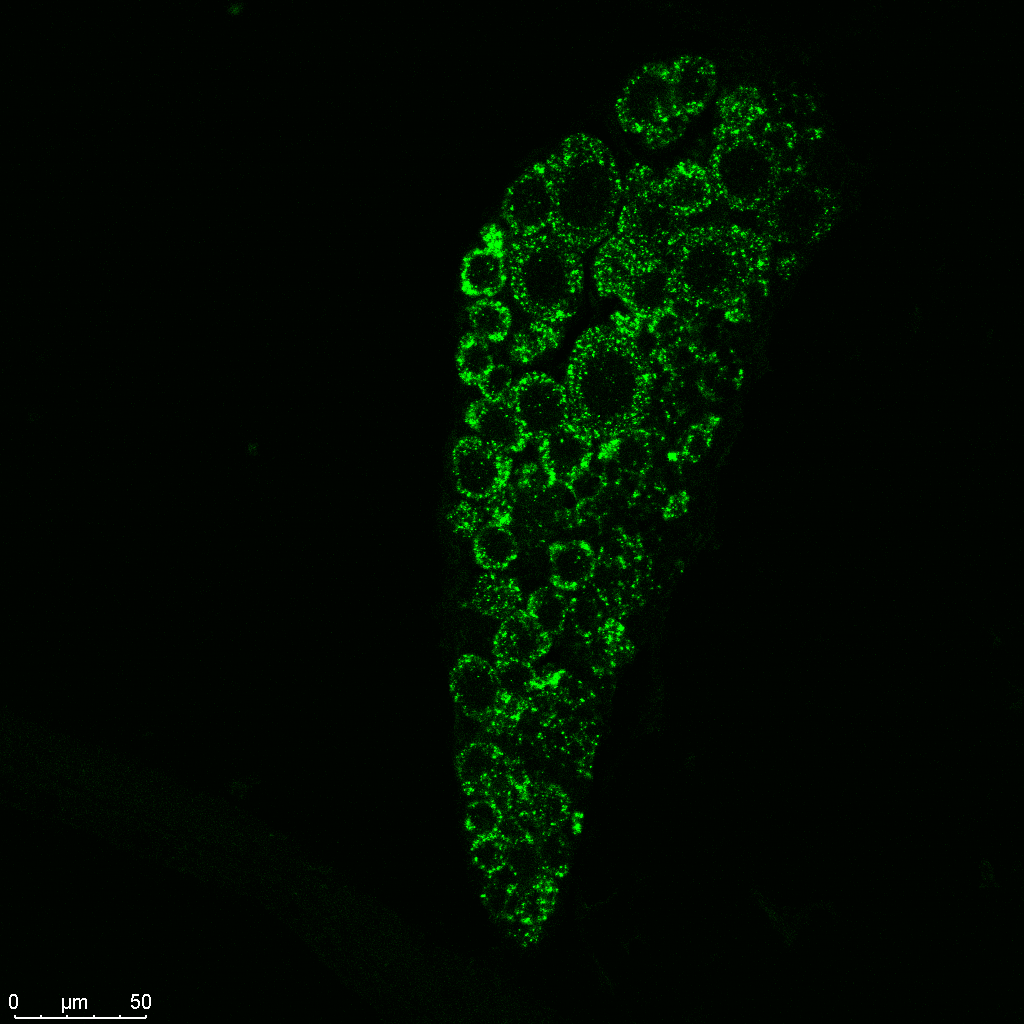

Supplement: Supplementary file 11 — Figure Source Data for Appendix Figures [file 44319_2026_775_MOESM11_ESM.zip › Source Data for Appendix Figure S1 3-7/Appendix Figure S6/Appendix Figure S6D/piwil2/piwil2 XAV939.tif]

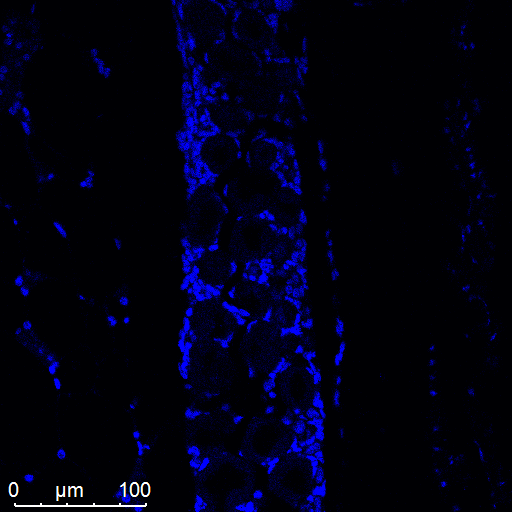

Supplement: Supplementary file 11 — Figure Source Data for Appendix Figures [file 44319_2026_775_MOESM11_ESM.zip › Source Data for Appendix Figure S1 3-7/Appendix Figure S6/Appendix Figure S6D/tdrd1/DAPI DMSO.tif]

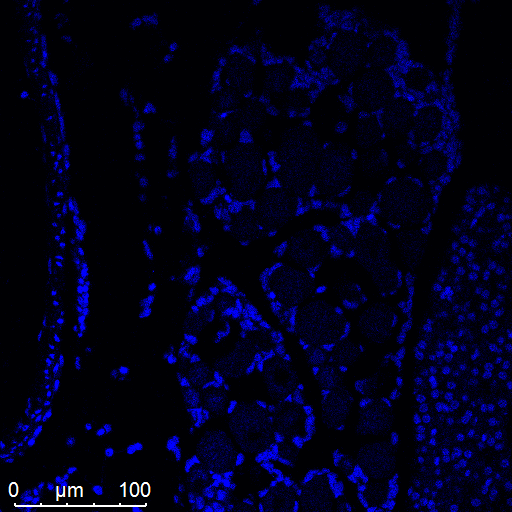

Supplement: Supplementary file 11 — Figure Source Data for Appendix Figures [file 44319_2026_775_MOESM11_ESM.zip › Source Data for Appendix Figure S1 3-7/Appendix Figure S6/Appendix Figure S6D/tdrd1/DAPI PNU74654.tif]

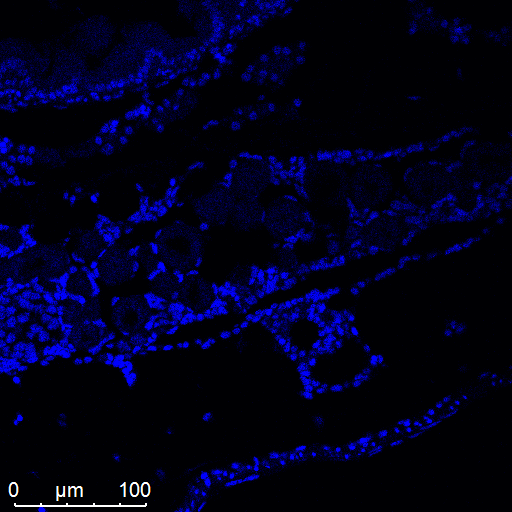

Supplement: Supplementary file 11 — Figure Source Data for Appendix Figures [file 44319_2026_775_MOESM11_ESM.zip › Source Data for Appendix Figure S1 3-7/Appendix Figure S6/Appendix Figure S6D/tdrd1/DAPI XAV939.tif]

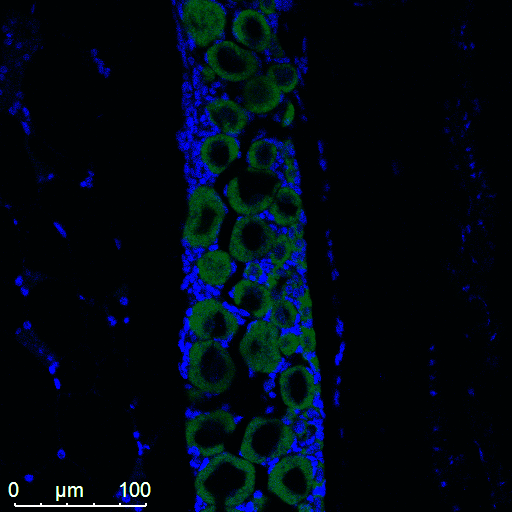

Supplement: Supplementary file 11 — Figure Source Data for Appendix Figures [file 44319_2026_775_MOESM11_ESM.zip › Source Data for Appendix Figure S1 3-7/Appendix Figure S6/Appendix Figure S6D/tdrd1/Merge DMSO.tif]

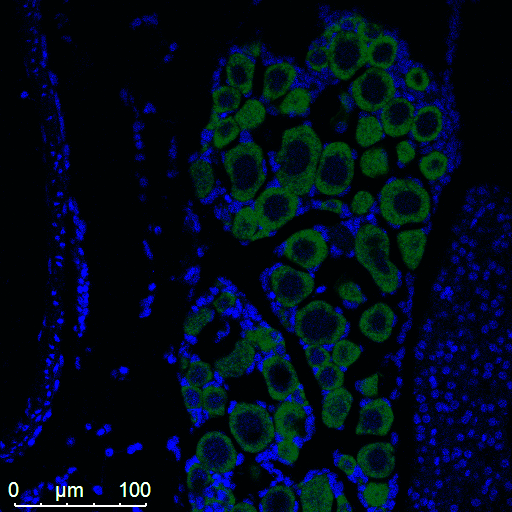

Supplement: Supplementary file 11 — Figure Source Data for Appendix Figures [file 44319_2026_775_MOESM11_ESM.zip › Source Data for Appendix Figure S1 3-7/Appendix Figure S6/Appendix Figure S6D/tdrd1/Merge PNU74654.tif]

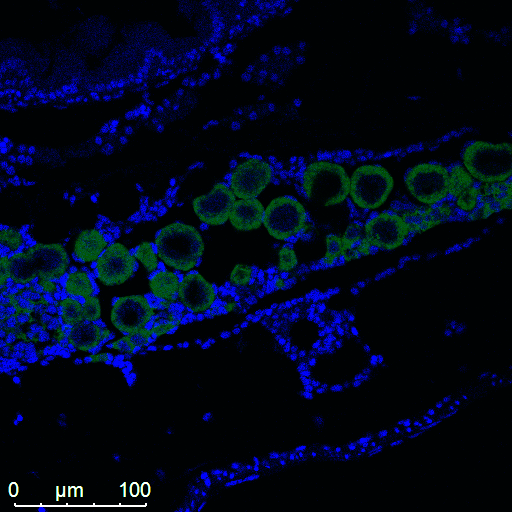

Supplement: Supplementary file 11 — Figure Source Data for Appendix Figures [file 44319_2026_775_MOESM11_ESM.zip › Source Data for Appendix Figure S1 3-7/Appendix Figure S6/Appendix Figure S6D/tdrd1/Merge XAV939.tif]

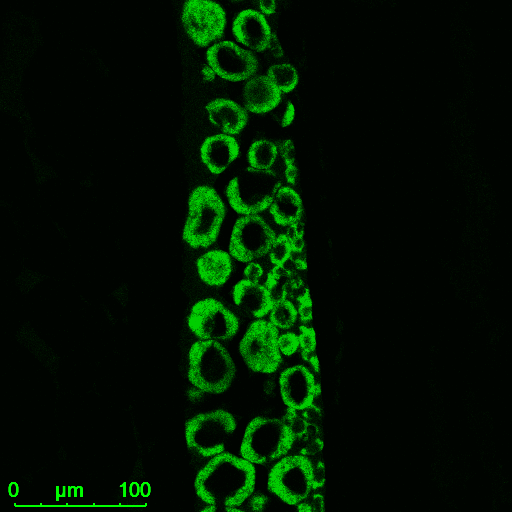

Supplement: Supplementary file 11 — Figure Source Data for Appendix Figures [file 44319_2026_775_MOESM11_ESM.zip › Source Data for Appendix Figure S1 3-7/Appendix Figure S6/Appendix Figure S6D/tdrd1/tdrd1 DMSO.tif]

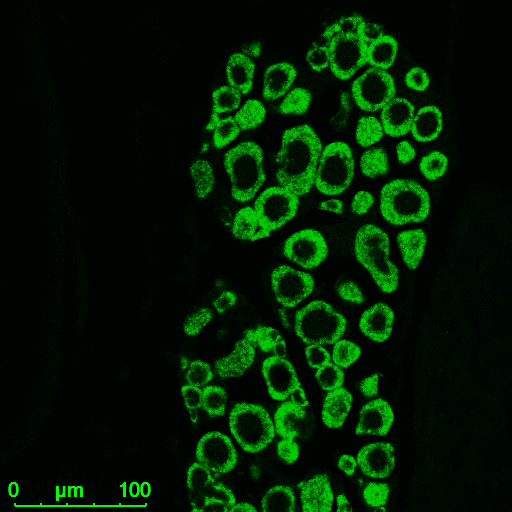

Supplement: Supplementary file 11 — Figure Source Data for Appendix Figures [file 44319_2026_775_MOESM11_ESM.zip › Source Data for Appendix Figure S1 3-7/Appendix Figure S6/Appendix Figure S6D/tdrd1/tdrd1 PNU74654.tif]

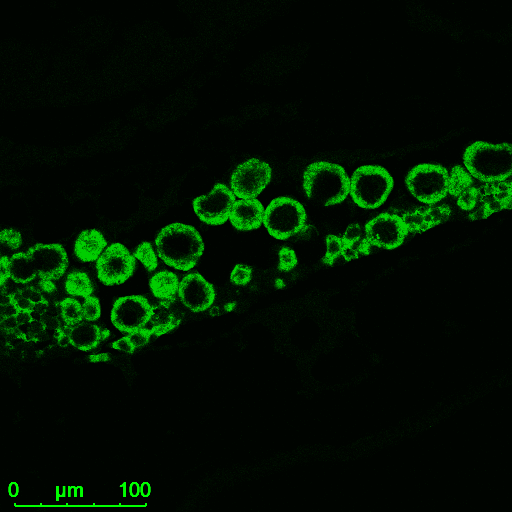

Supplement: Supplementary file 11 — Figure Source Data for Appendix Figures [file 44319_2026_775_MOESM11_ESM.zip › Source Data for Appendix Figure S1 3-7/Appendix Figure S6/Appendix Figure S6D/tdrd1/tdrd1 XAV939.tif]

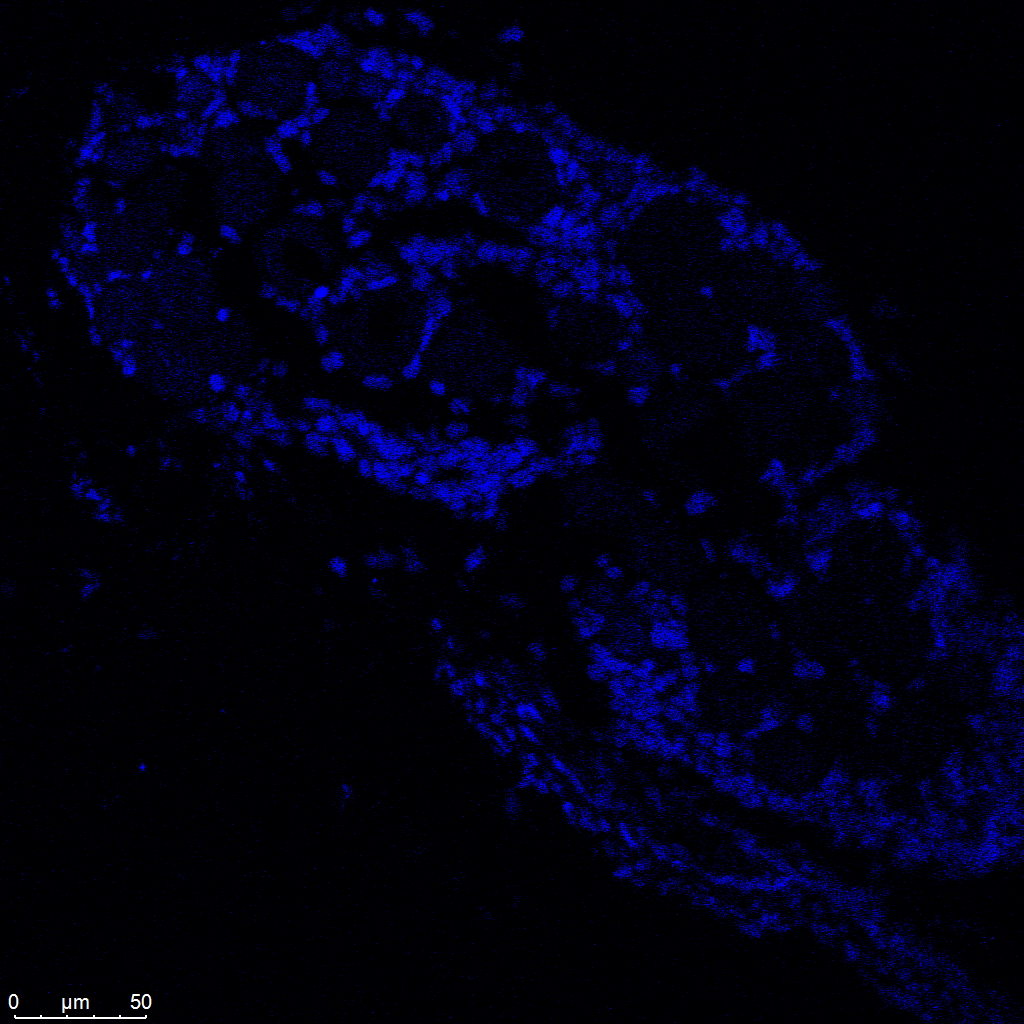

Supplement: Supplementary file 11 — Figure Source Data for Appendix Figures [file 44319_2026_775_MOESM11_ESM.zip › Source Data for Appendix Figure S1 3-7/Appendix Figure S6/Appendix Figure S6D/tdrd7a/DAPI DMSO.tif]

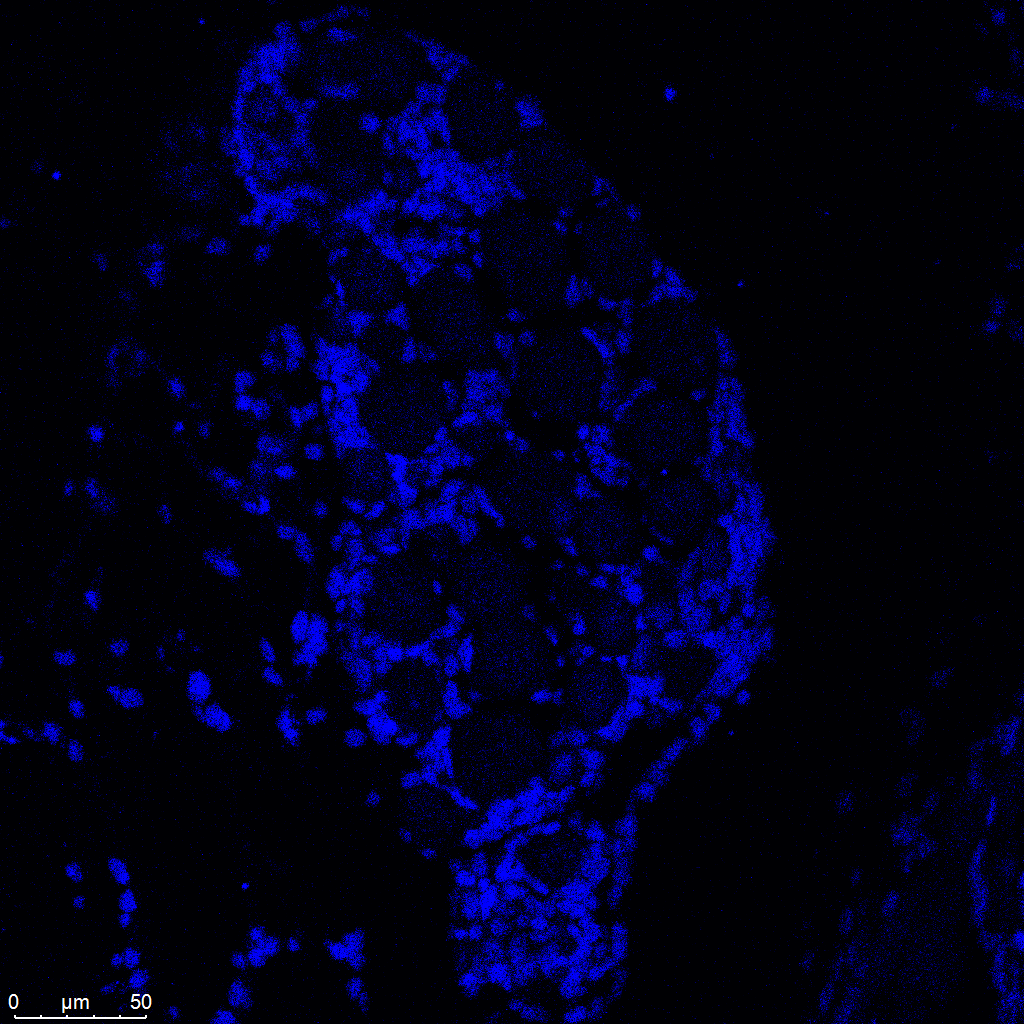

Supplement: Supplementary file 11 — Figure Source Data for Appendix Figures [file 44319_2026_775_MOESM11_ESM.zip › Source Data for Appendix Figure S1 3-7/Appendix Figure S6/Appendix Figure S6D/tdrd7a/DAPI PNU74654.tif]

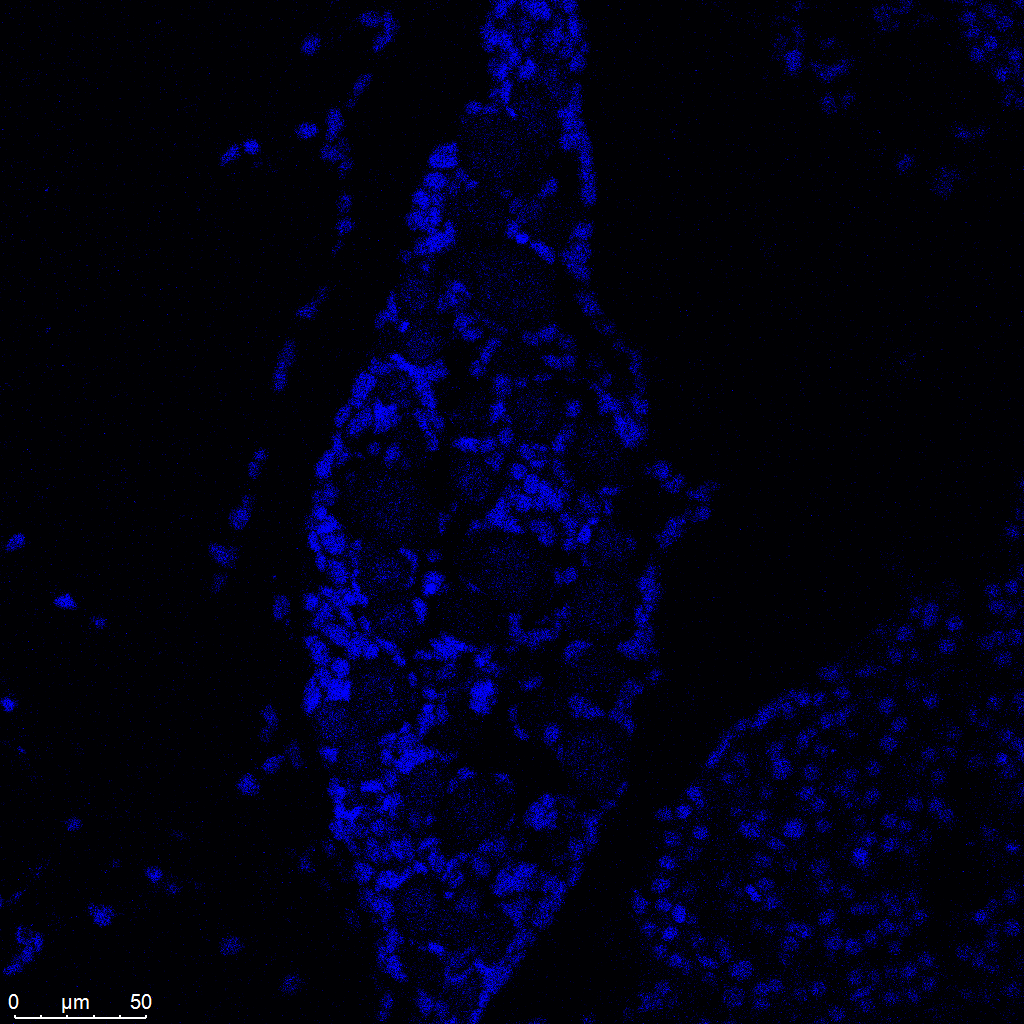

Supplement: Supplementary file 11 — Figure Source Data for Appendix Figures [file 44319_2026_775_MOESM11_ESM.zip › Source Data for Appendix Figure S1 3-7/Appendix Figure S6/Appendix Figure S6D/tdrd7a/DAPI XAV939.tif]

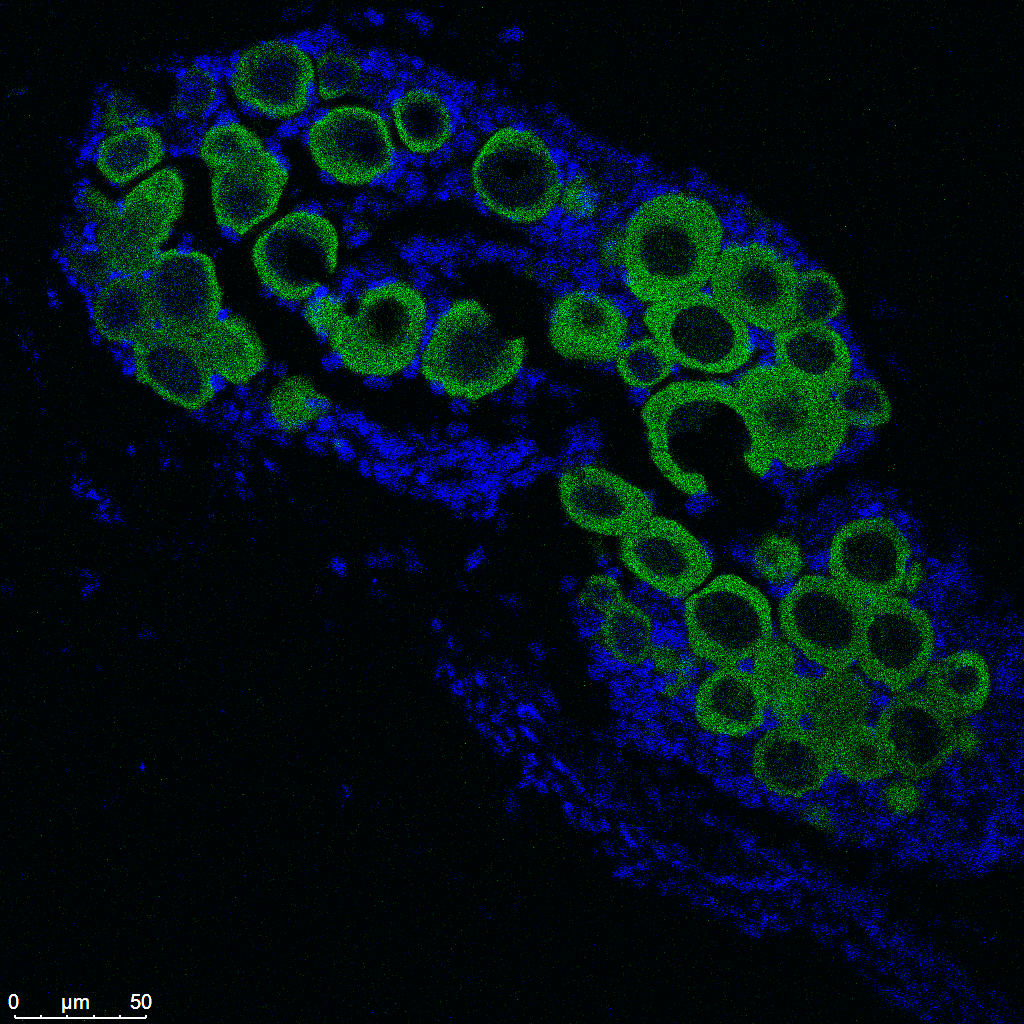

Supplement: Supplementary file 11 — Figure Source Data for Appendix Figures [file 44319_2026_775_MOESM11_ESM.zip › Source Data for Appendix Figure S1 3-7/Appendix Figure S6/Appendix Figure S6D/tdrd7a/Merge DMSO.tif]

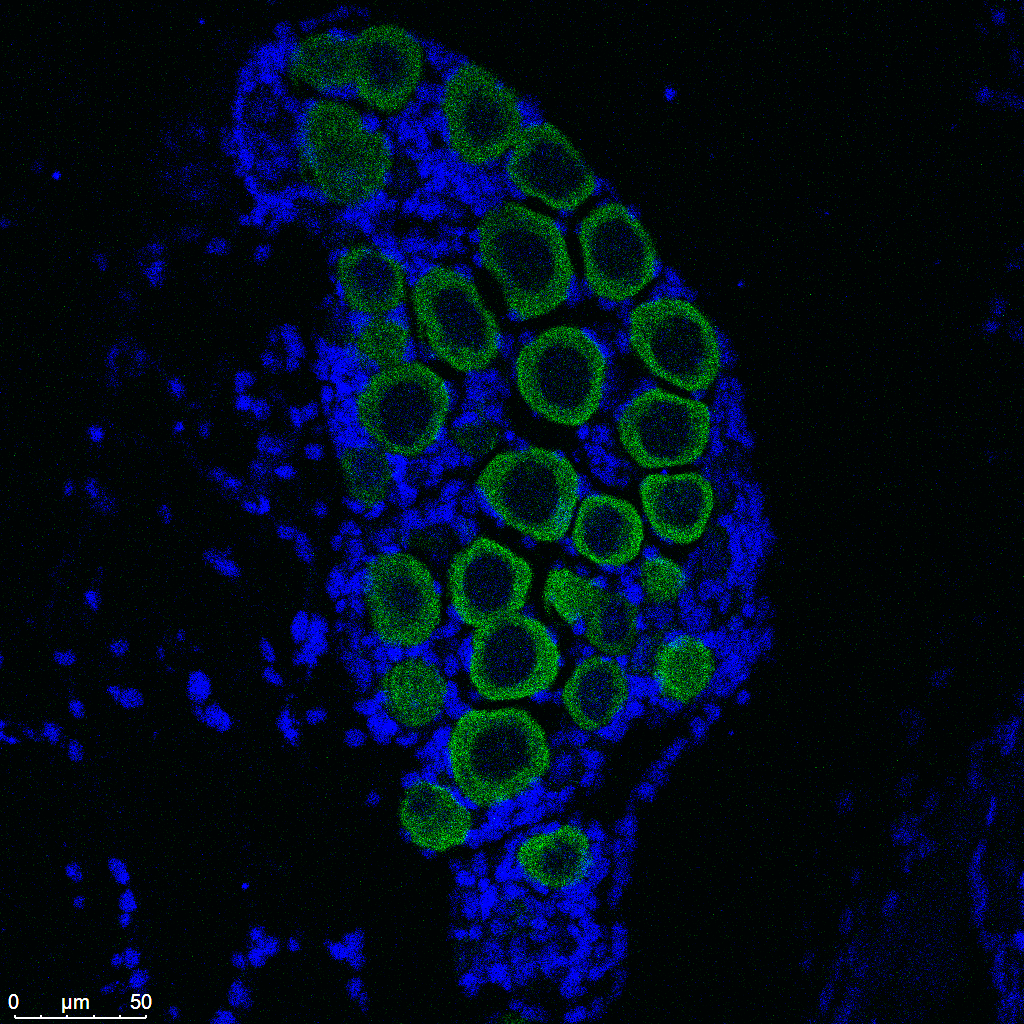

Supplement: Supplementary file 11 — Figure Source Data for Appendix Figures [file 44319_2026_775_MOESM11_ESM.zip › Source Data for Appendix Figure S1 3-7/Appendix Figure S6/Appendix Figure S6D/tdrd7a/Merge PNU74654.tif]

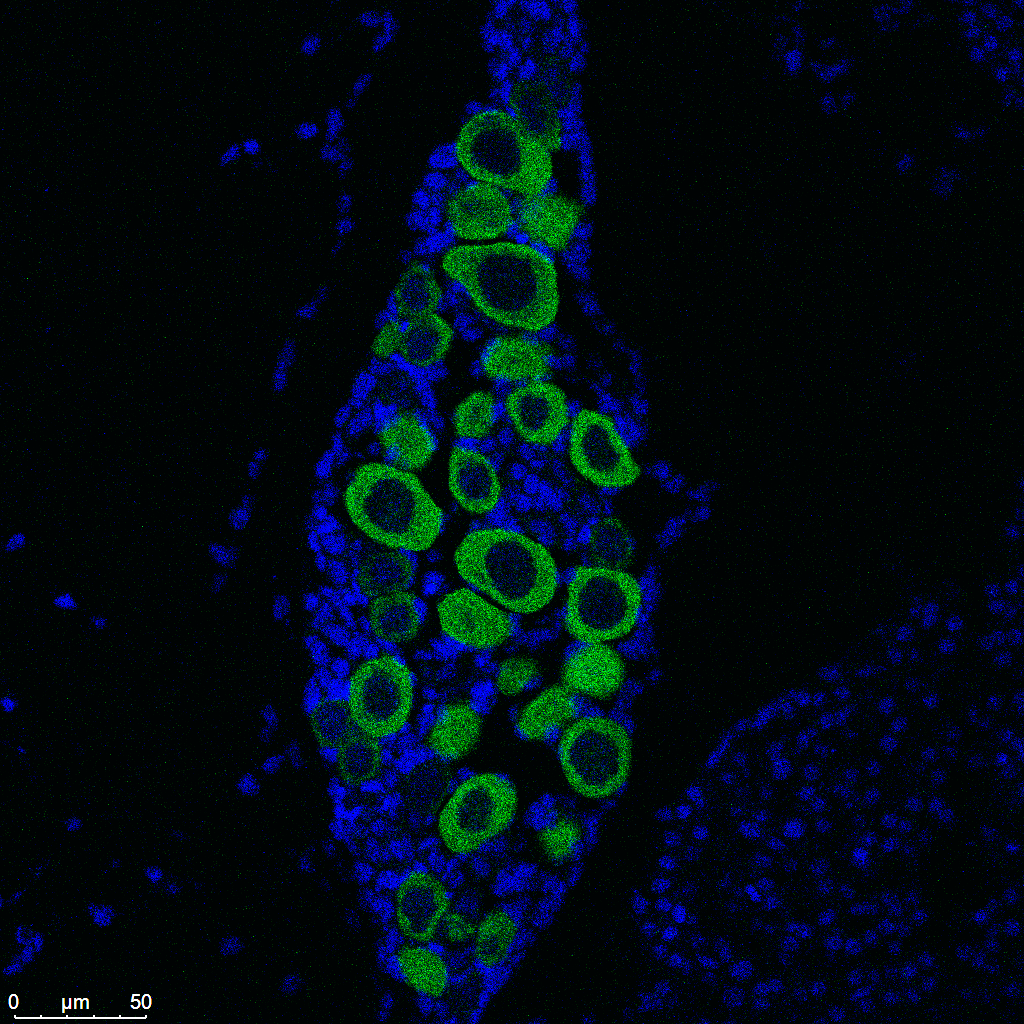

Supplement: Supplementary file 11 — Figure Source Data for Appendix Figures [file 44319_2026_775_MOESM11_ESM.zip › Source Data for Appendix Figure S1 3-7/Appendix Figure S6/Appendix Figure S6D/tdrd7a/Merge XAV939.tif]

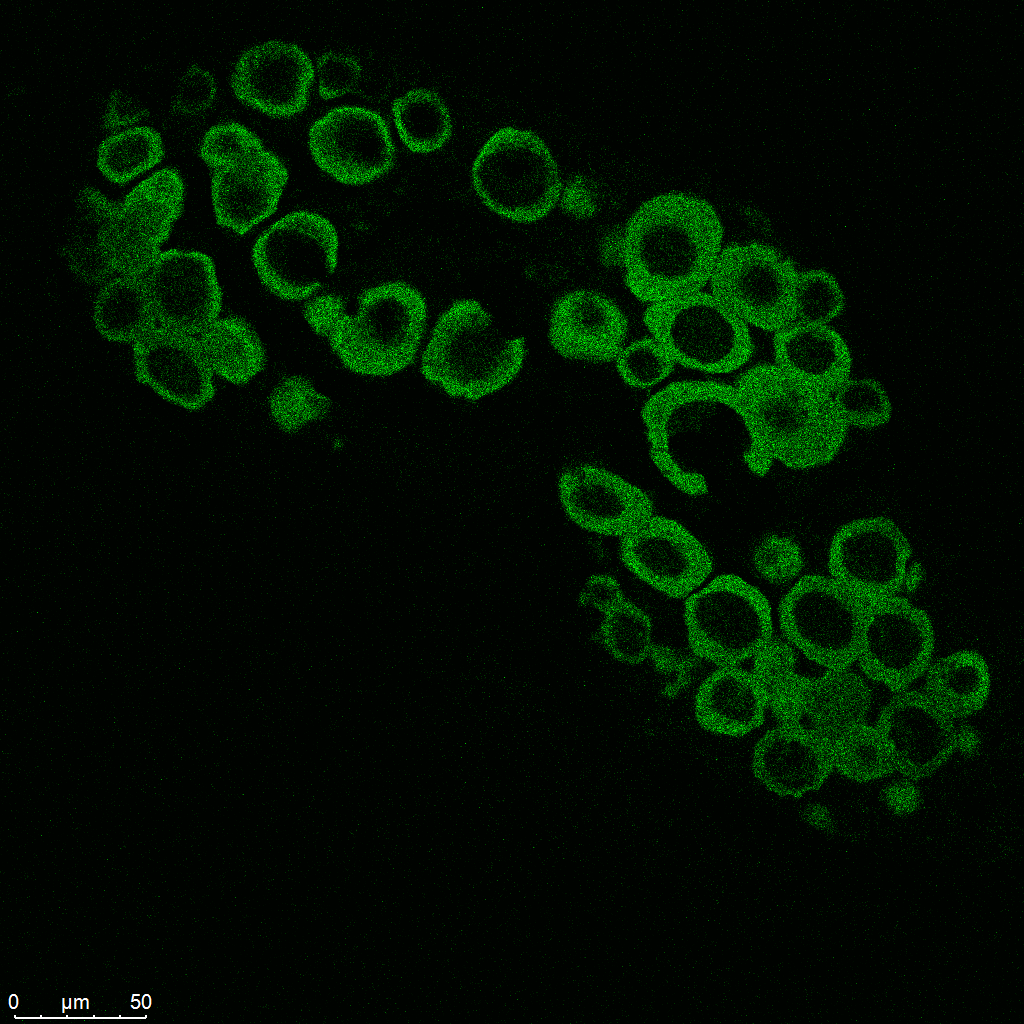

Supplement: Supplementary file 11 — Figure Source Data for Appendix Figures [file 44319_2026_775_MOESM11_ESM.zip › Source Data for Appendix Figure S1 3-7/Appendix Figure S6/Appendix Figure S6D/tdrd7a/tdrd7a DMSO.tif]

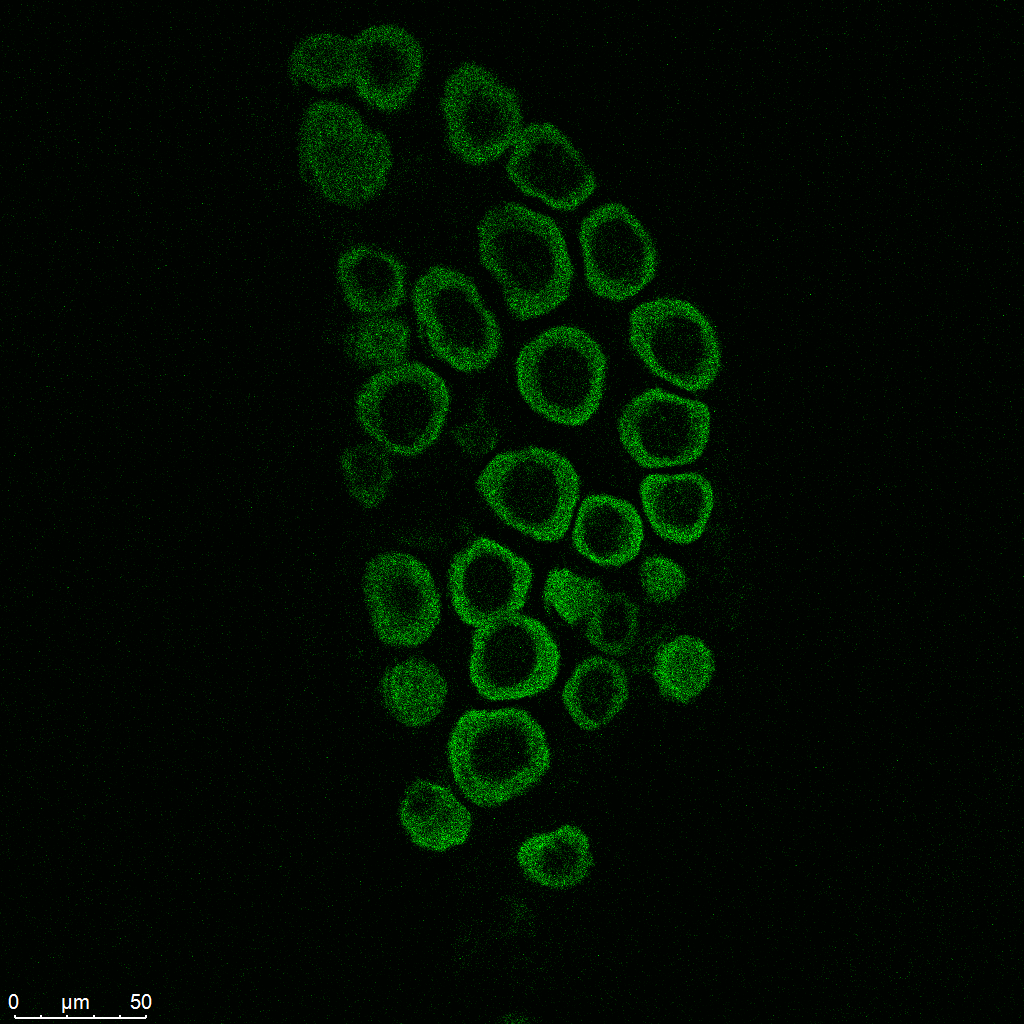

Supplement: Supplementary file 11 — Figure Source Data for Appendix Figures [file 44319_2026_775_MOESM11_ESM.zip › Source Data for Appendix Figure S1 3-7/Appendix Figure S6/Appendix Figure S6D/tdrd7a/tdrd7a PNU74654.tif]

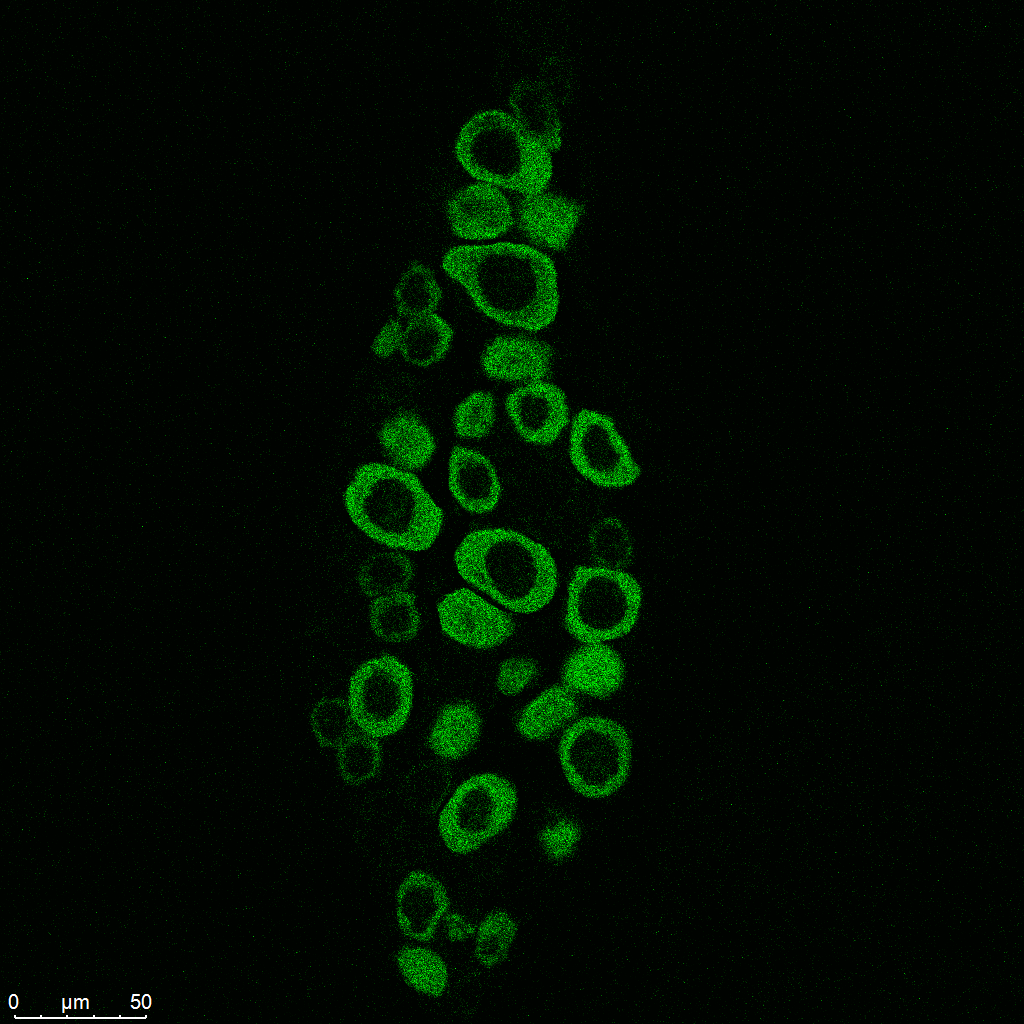

Supplement: Supplementary file 11 — Figure Source Data for Appendix Figures [file 44319_2026_775_MOESM11_ESM.zip › Source Data for Appendix Figure S1 3-7/Appendix Figure S6/Appendix Figure S6D/tdrd7a/tdrd7a XAV939.tif]

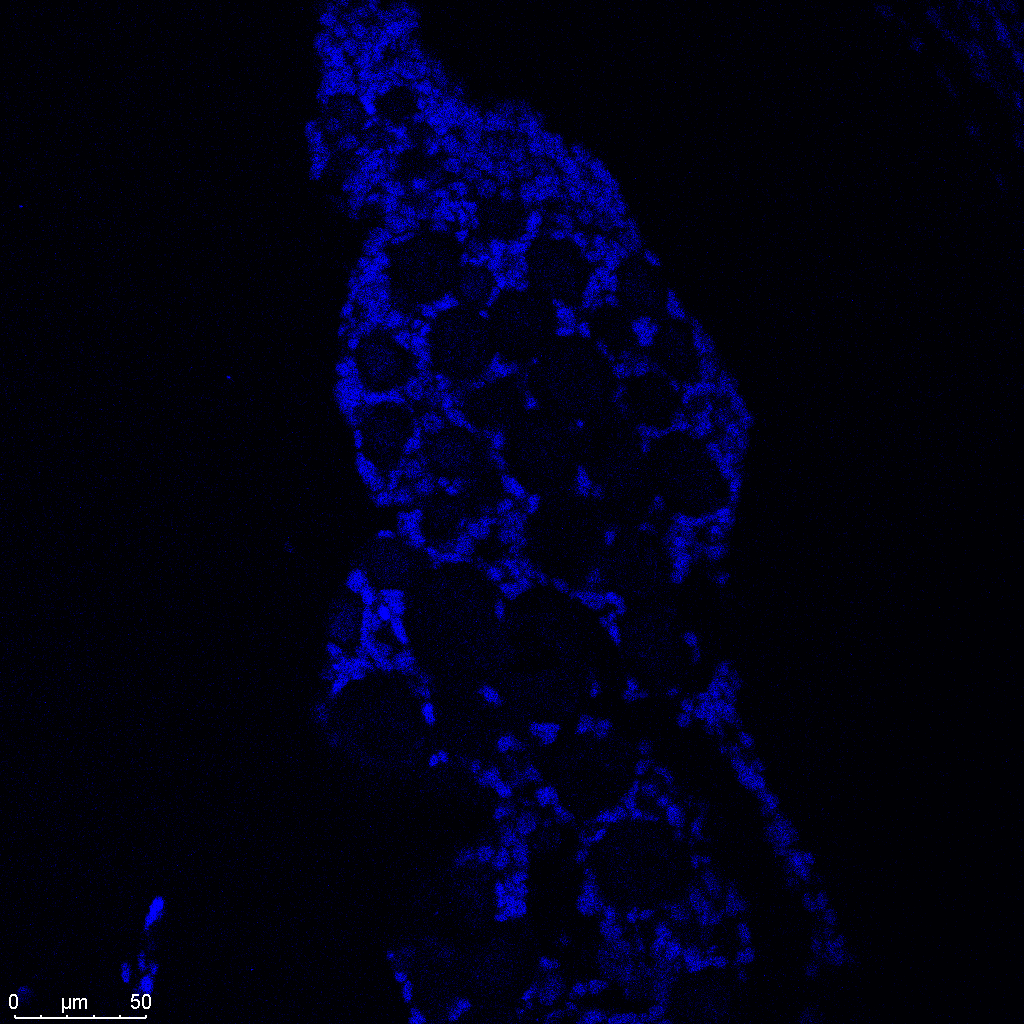

Supplement: Supplementary file 11 — Figure Source Data for Appendix Figures [file 44319_2026_775_MOESM11_ESM.zip › Source Data for Appendix Figure S1 3-7/Appendix Figure S6/Appendix Figure S6D/tdrd9/DAPI DMSO.tif]

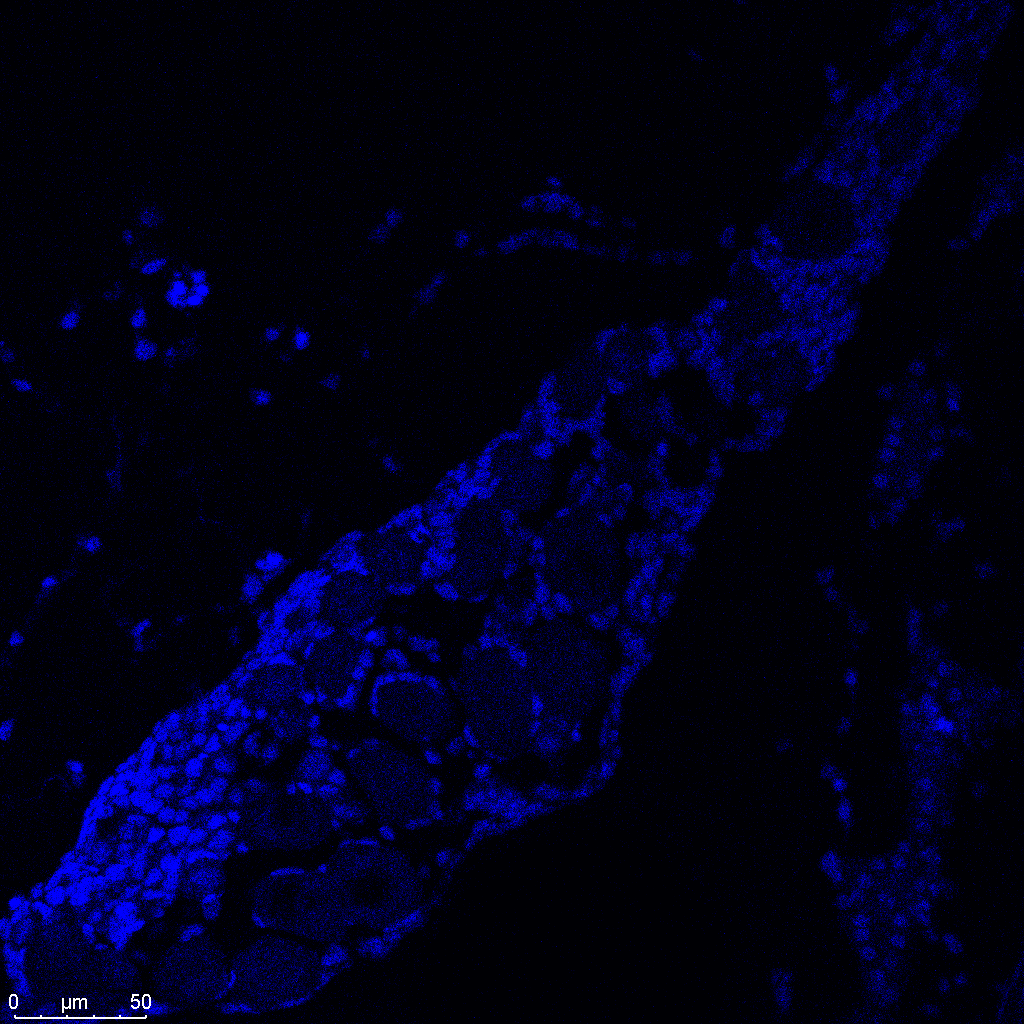

Supplement: Supplementary file 11 — Figure Source Data for Appendix Figures [file 44319_2026_775_MOESM11_ESM.zip › Source Data for Appendix Figure S1 3-7/Appendix Figure S6/Appendix Figure S6D/tdrd9/DAPI PNU74654.tif]

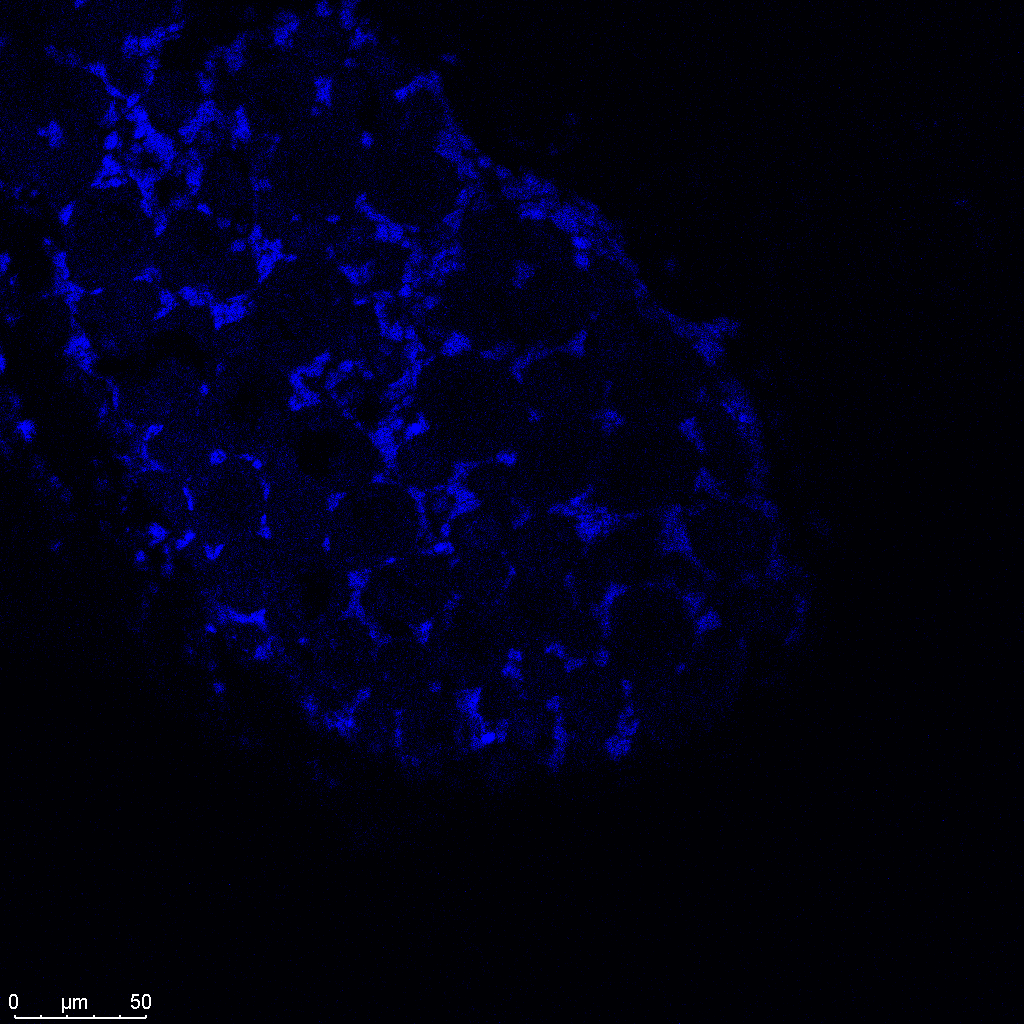

Supplement: Supplementary file 11 — Figure Source Data for Appendix Figures [file 44319_2026_775_MOESM11_ESM.zip › Source Data for Appendix Figure S1 3-7/Appendix Figure S6/Appendix Figure S6D/tdrd9/DAPI XAV939.tif]

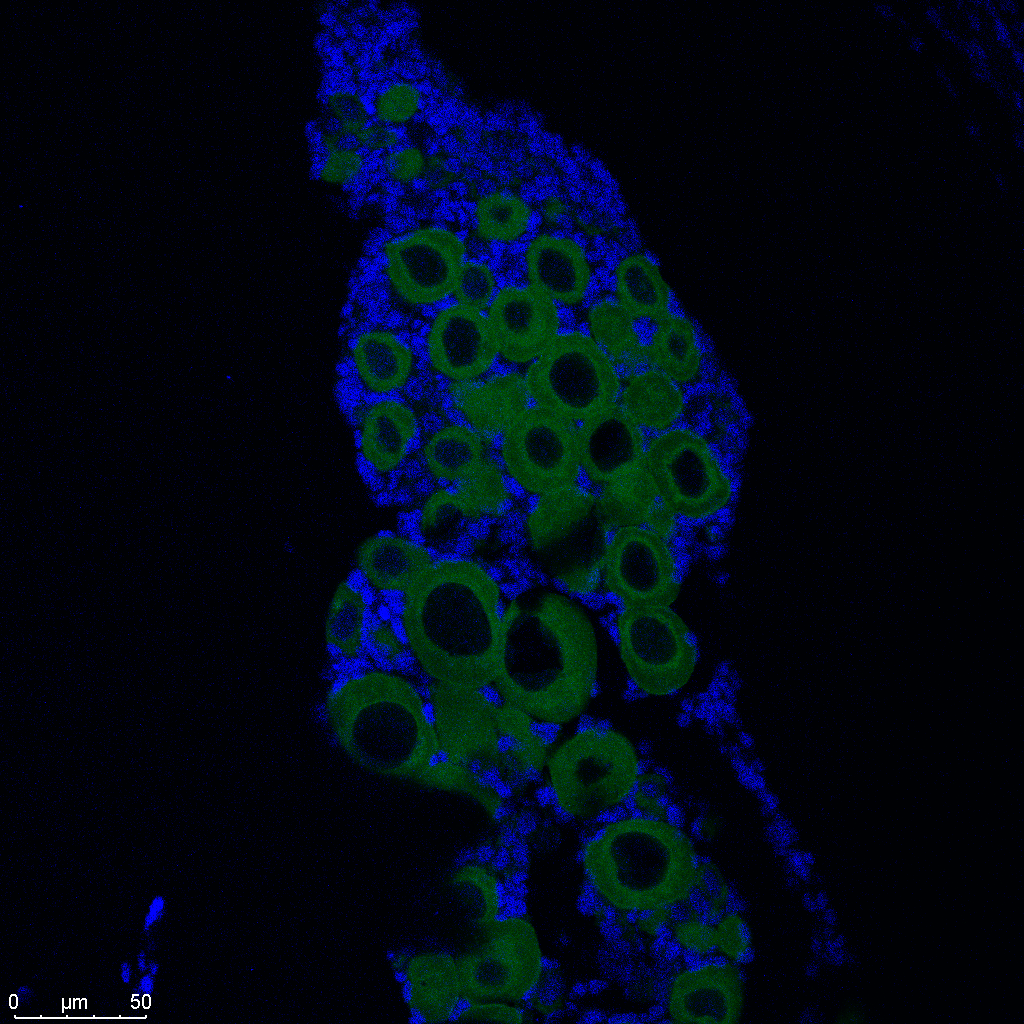

Supplement: Supplementary file 11 — Figure Source Data for Appendix Figures [file 44319_2026_775_MOESM11_ESM.zip › Source Data for Appendix Figure S1 3-7/Appendix Figure S6/Appendix Figure S6D/tdrd9/Merge DMSO.tif]

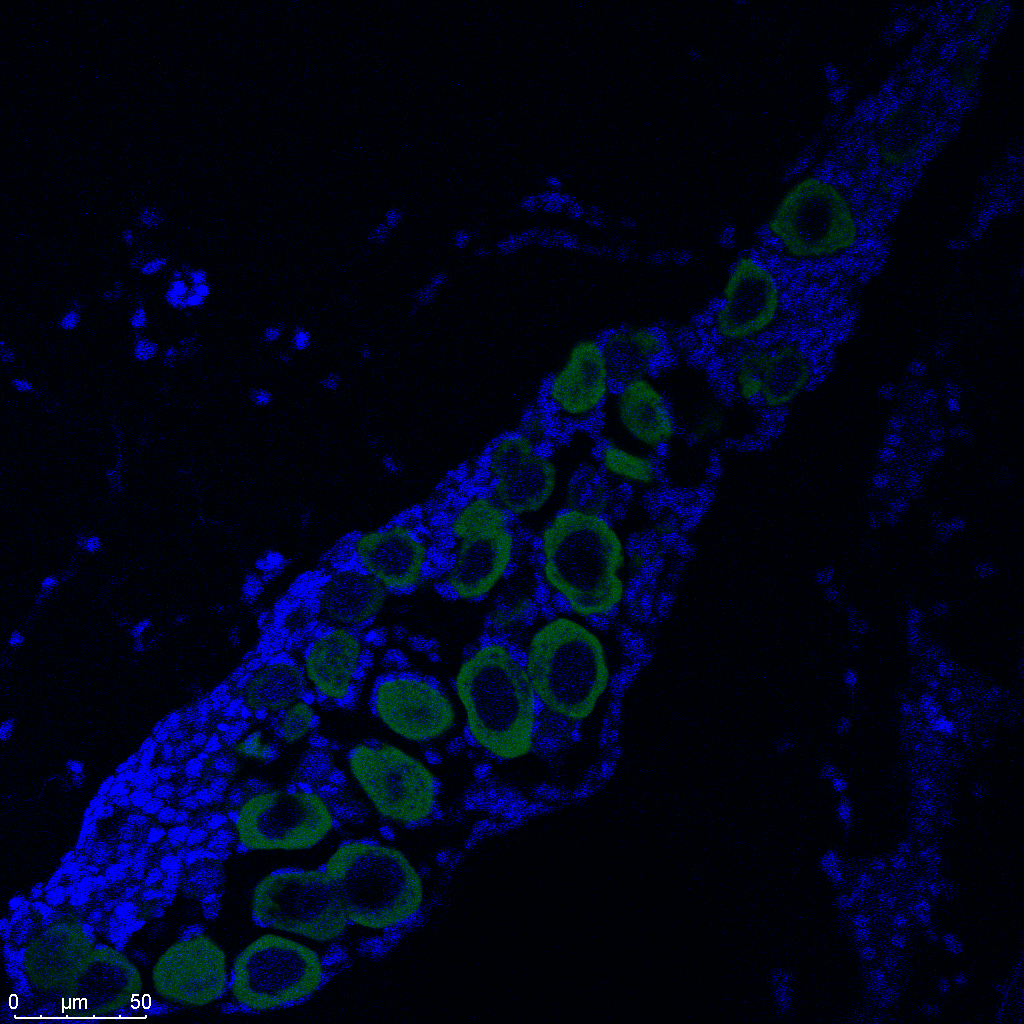

Supplement: Supplementary file 11 — Figure Source Data for Appendix Figures [file 44319_2026_775_MOESM11_ESM.zip › Source Data for Appendix Figure S1 3-7/Appendix Figure S6/Appendix Figure S6D/tdrd9/Merge PNU74654.tif]

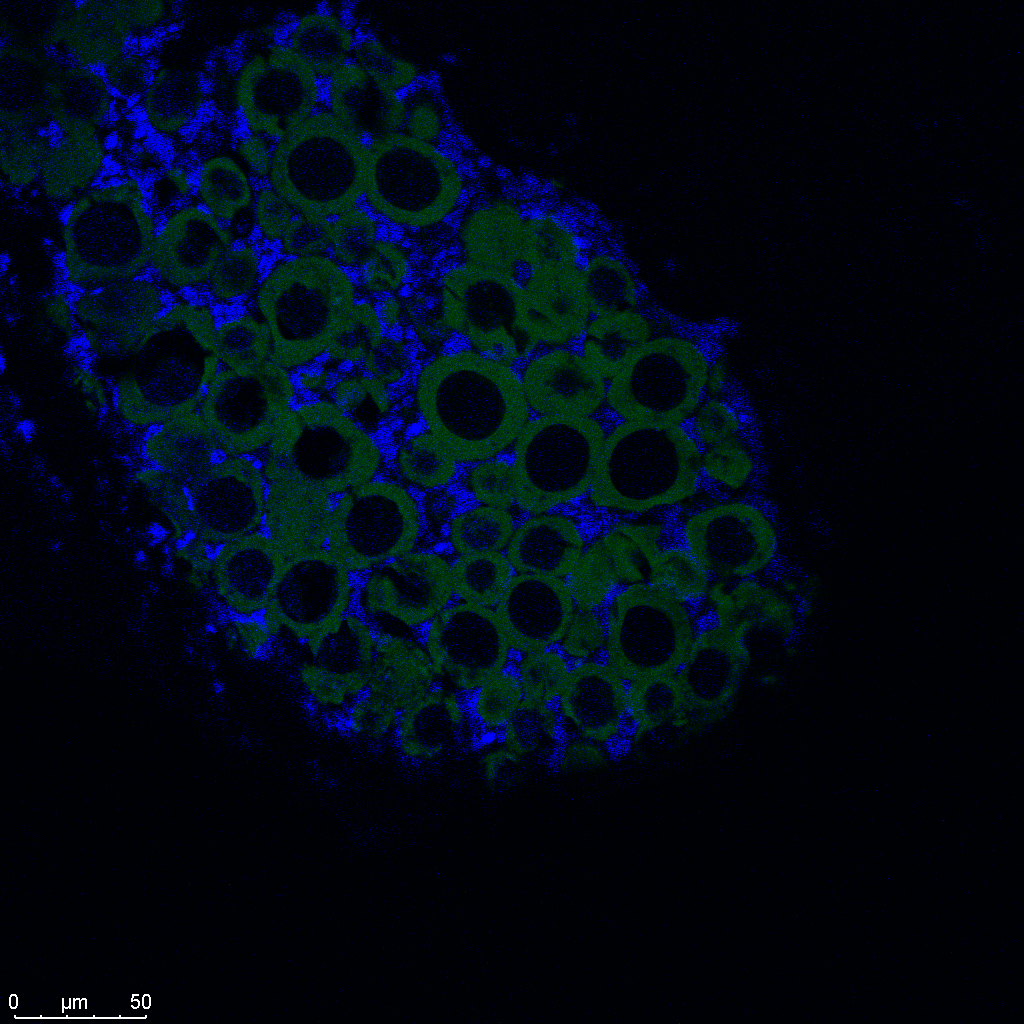

Supplement: Supplementary file 11 — Figure Source Data for Appendix Figures [file 44319_2026_775_MOESM11_ESM.zip › Source Data for Appendix Figure S1 3-7/Appendix Figure S6/Appendix Figure S6D/tdrd9/Merge XAV939.tif]

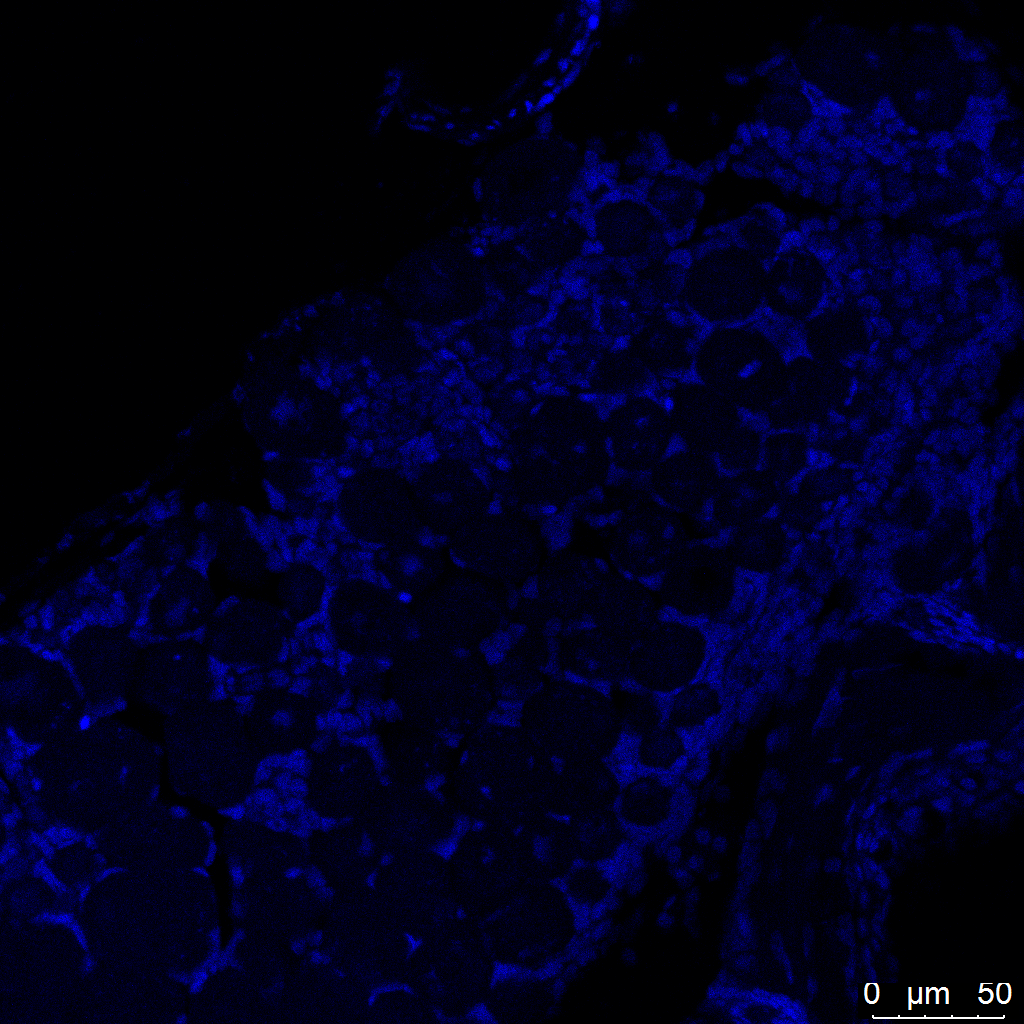

Supplement: Supplementary file 11 — Figure Source Data for Appendix Figures [file 44319_2026_775_MOESM11_ESM.zip › Source Data for Appendix Figure S1 3-7/Appendix Figure S7/Appendix Figure S7C/ddx4/DAPI wnt8.tif]

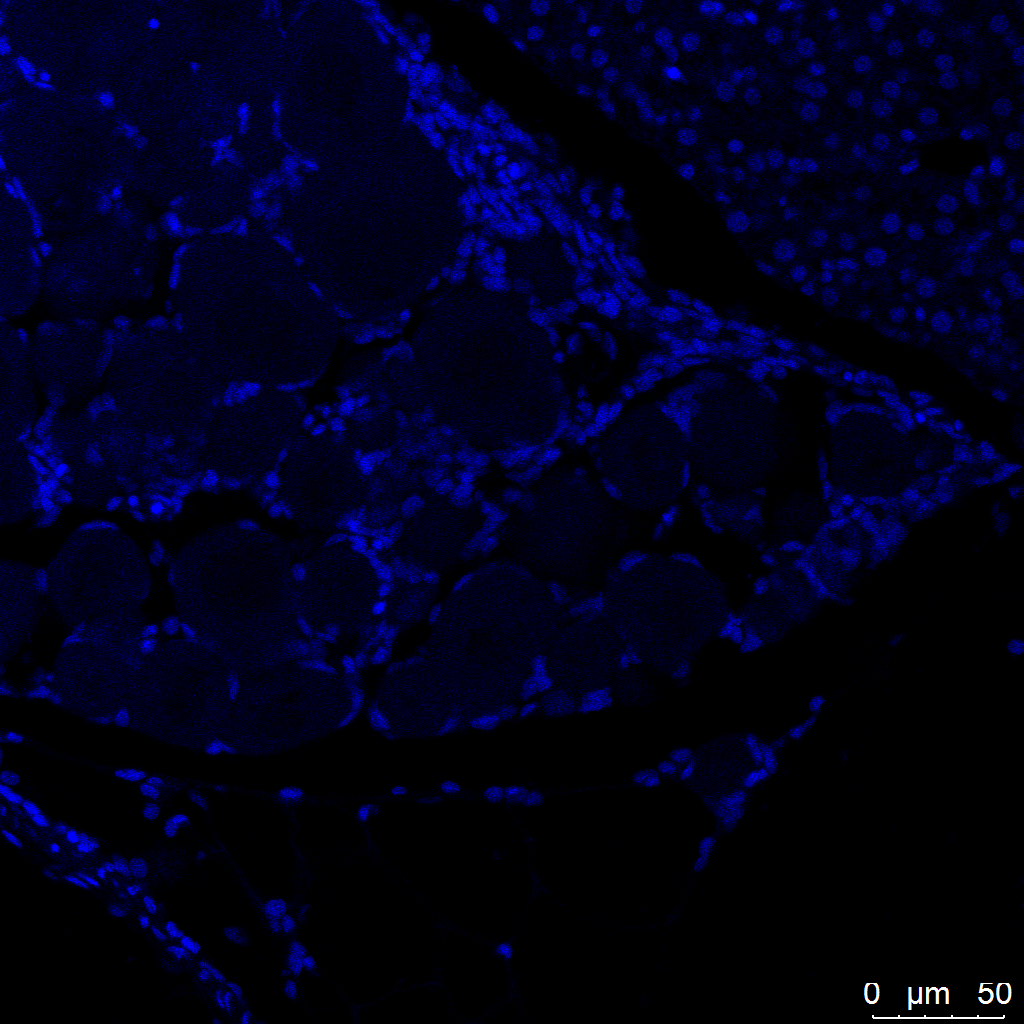

Supplement: Supplementary file 11 — Figure Source Data for Appendix Figures [file 44319_2026_775_MOESM11_ESM.zip › Source Data for Appendix Figure S1 3-7/Appendix Figure S7/Appendix Figure S7C/ddx4/DAPI WT.tif]

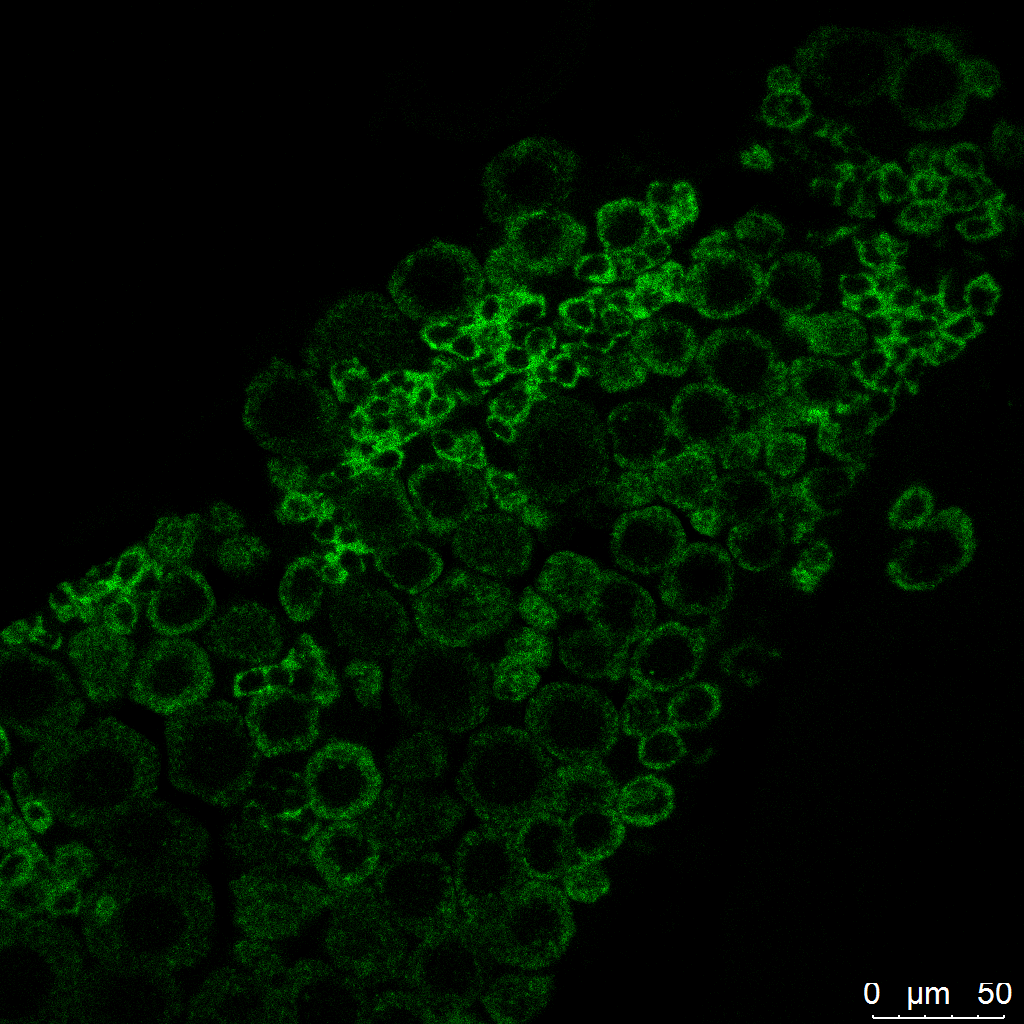

Supplement: Supplementary file 11 — Figure Source Data for Appendix Figures [file 44319_2026_775_MOESM11_ESM.zip › Source Data for Appendix Figure S1 3-7/Appendix Figure S7/Appendix Figure S7C/ddx4/ddx4 wnt8.tif]

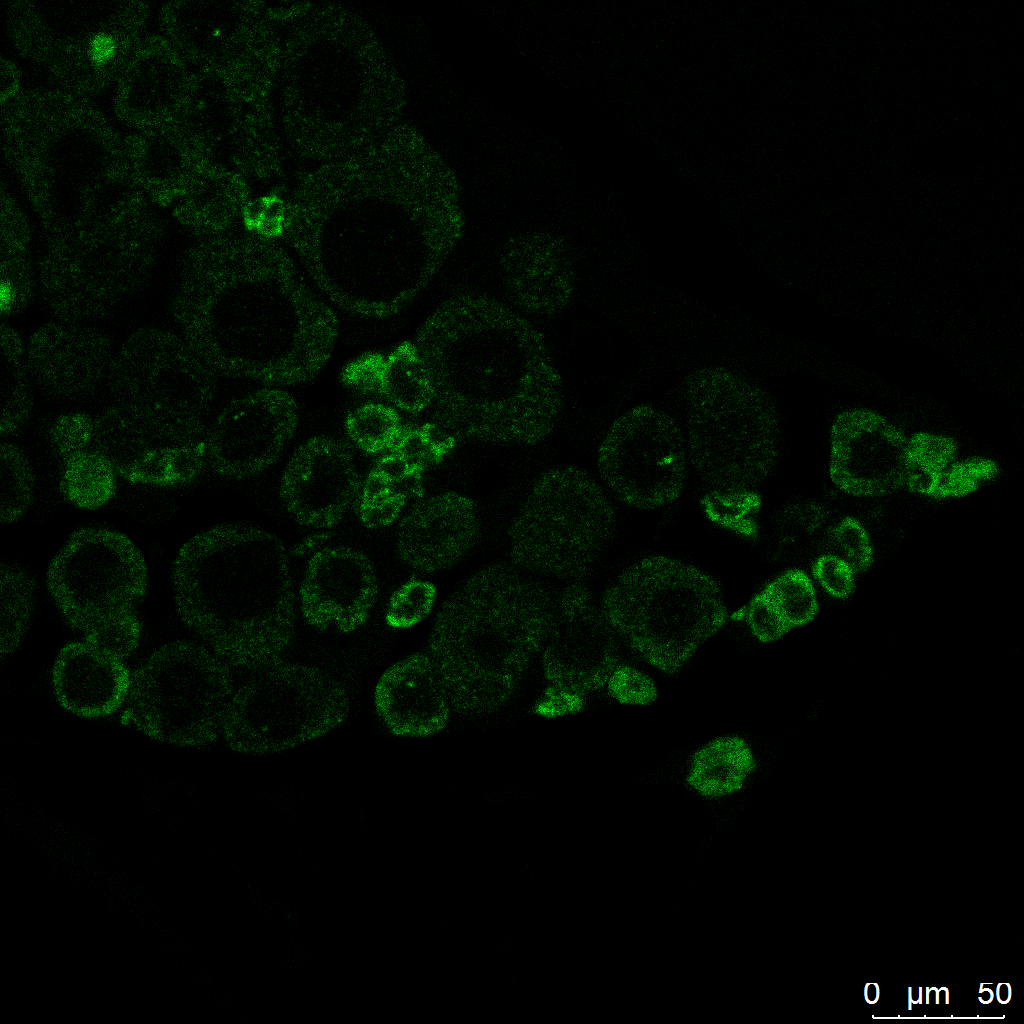

Supplement: Supplementary file 11 — Figure Source Data for Appendix Figures [file 44319_2026_775_MOESM11_ESM.zip › Source Data for Appendix Figure S1 3-7/Appendix Figure S7/Appendix Figure S7C/ddx4/ddx4 WT.tif]

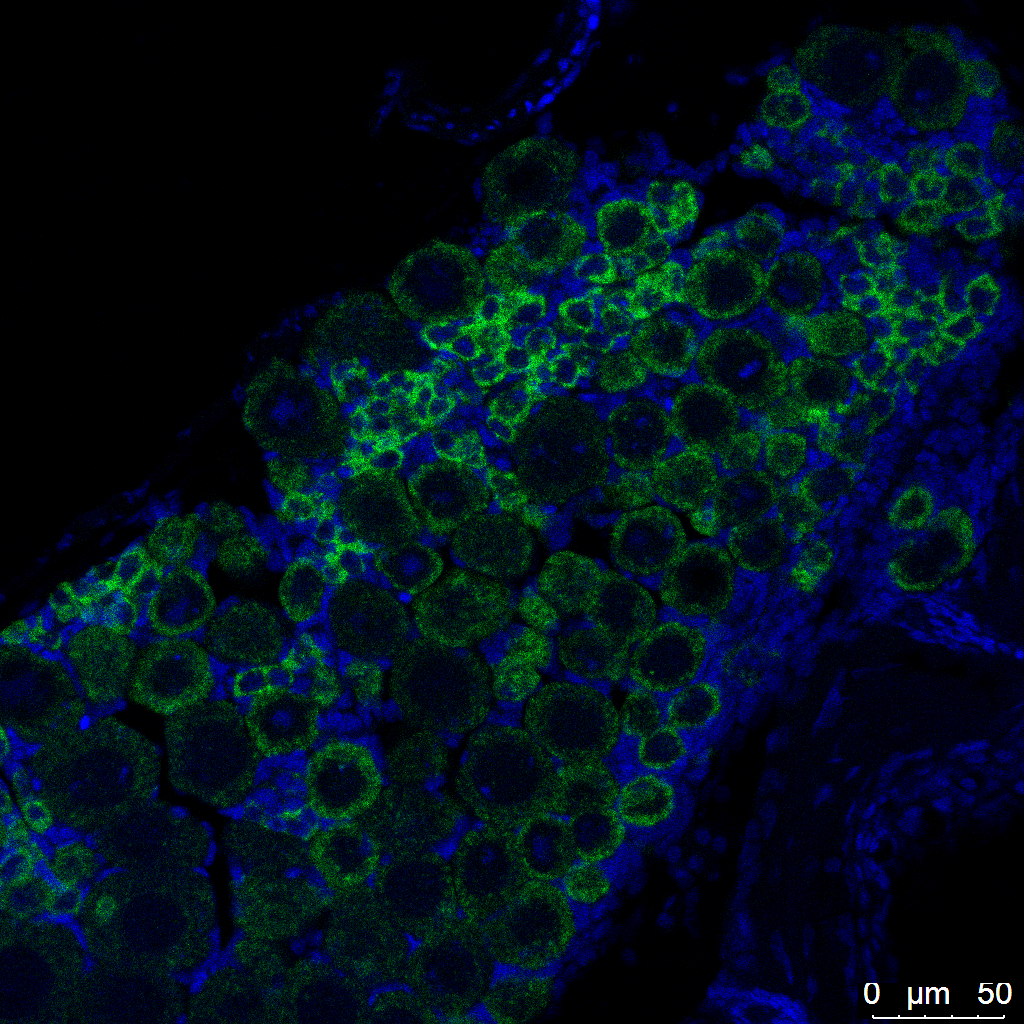

Supplement: Supplementary file 11 — Figure Source Data for Appendix Figures [file 44319_2026_775_MOESM11_ESM.zip › Source Data for Appendix Figure S1 3-7/Appendix Figure S7/Appendix Figure S7C/ddx4/Merge wnt8.tif]

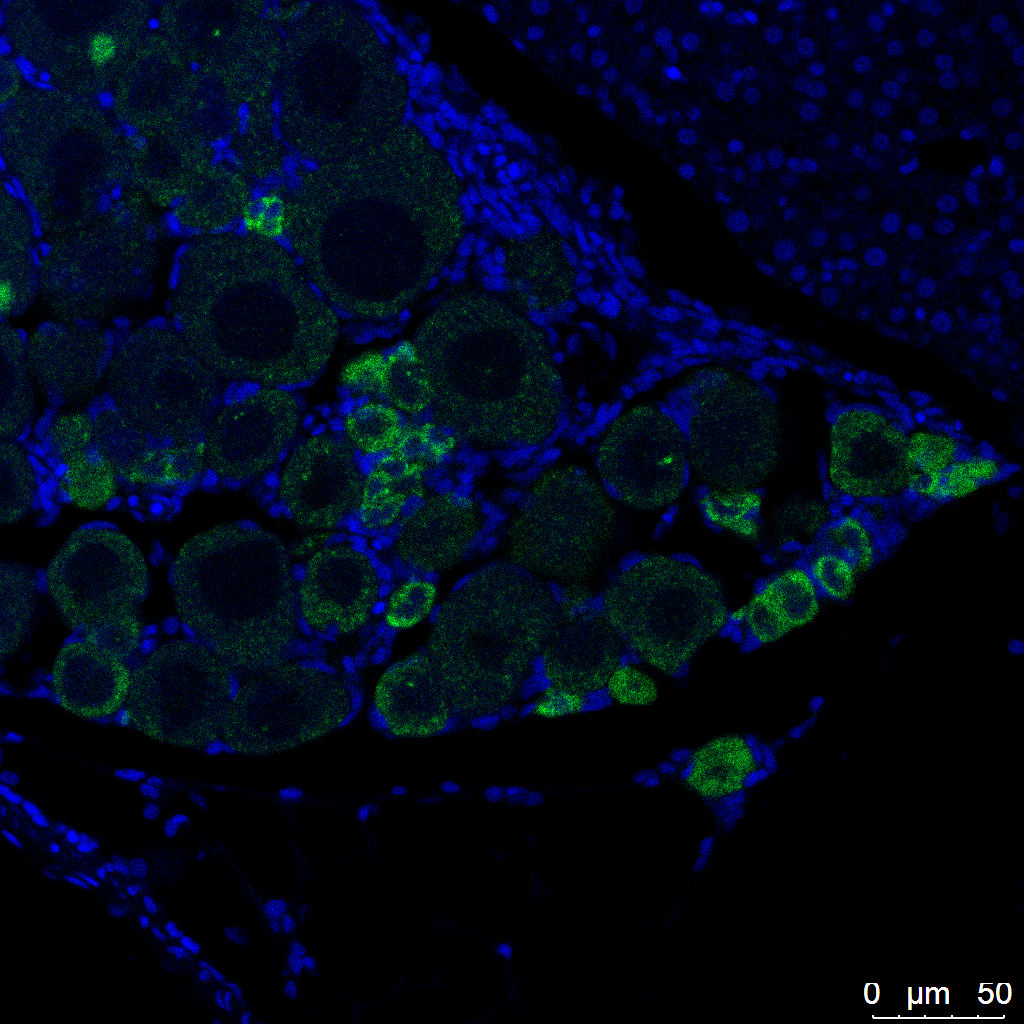

Supplement: Supplementary file 11 — Figure Source Data for Appendix Figures [file 44319_2026_775_MOESM11_ESM.zip › Source Data for Appendix Figure S1 3-7/Appendix Figure S7/Appendix Figure S7C/ddx4/Merge WT.tif]

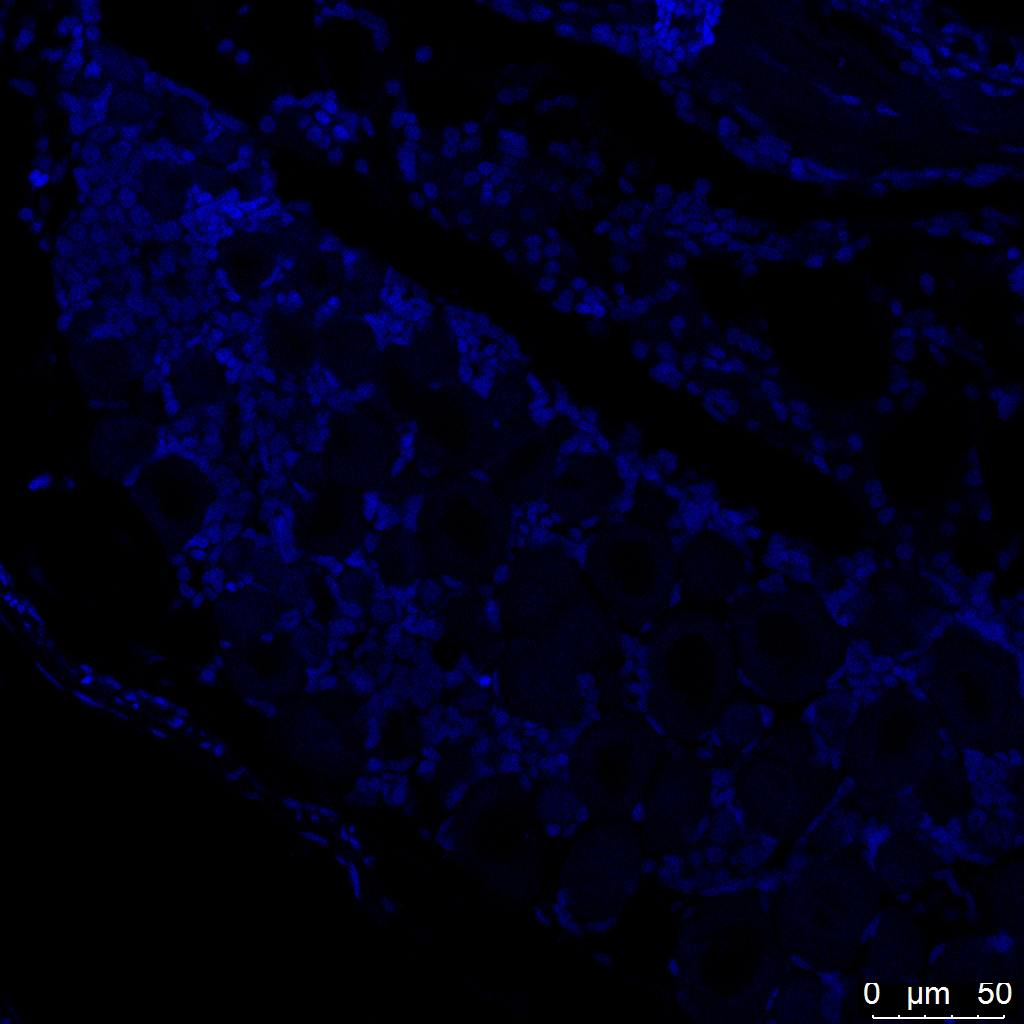

Supplement: Supplementary file 11 — Figure Source Data for Appendix Figures [file 44319_2026_775_MOESM11_ESM.zip › Source Data for Appendix Figure S1 3-7/Appendix Figure S7/Appendix Figure S7C/dnd1/DAPI wnt8.tif]

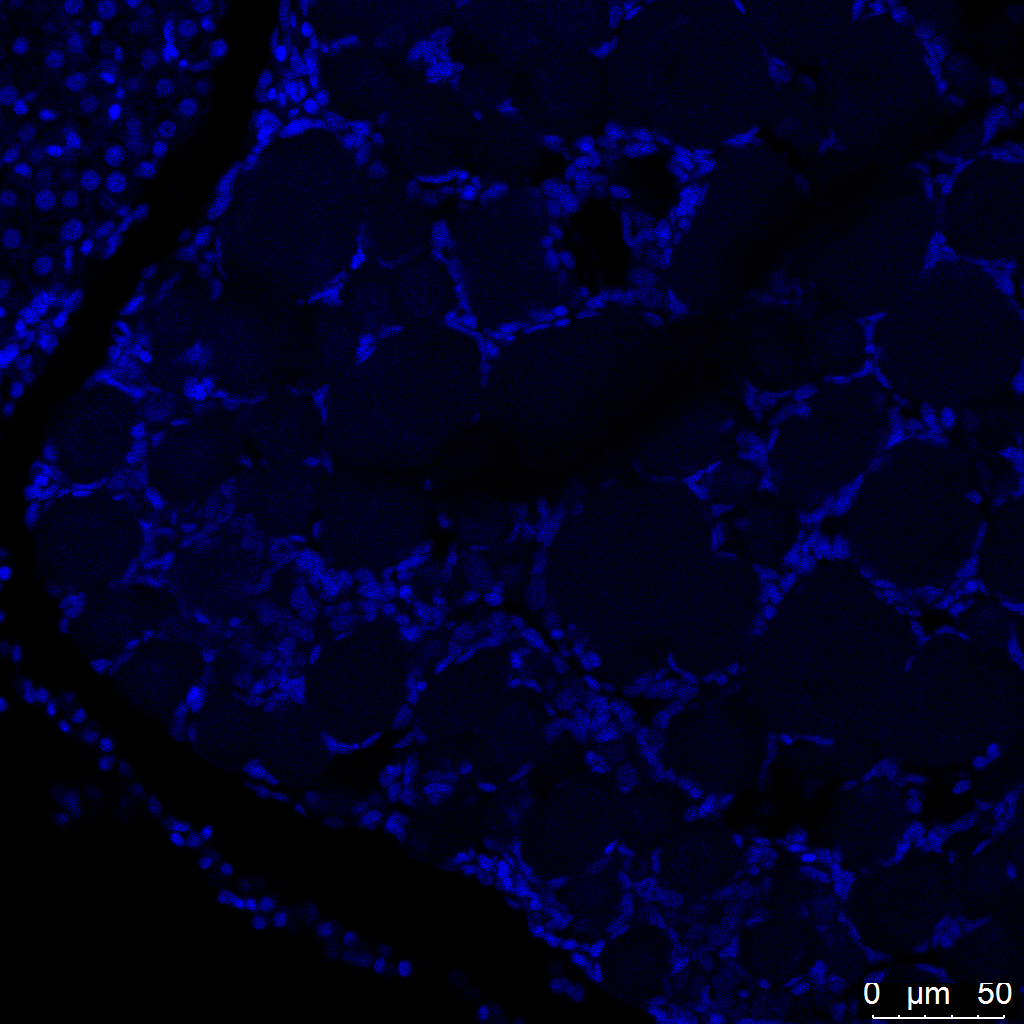

Supplement: Supplementary file 11 — Figure Source Data for Appendix Figures [file 44319_2026_775_MOESM11_ESM.zip › Source Data for Appendix Figure S1 3-7/Appendix Figure S7/Appendix Figure S7C/dnd1/DAPI WT.tif]

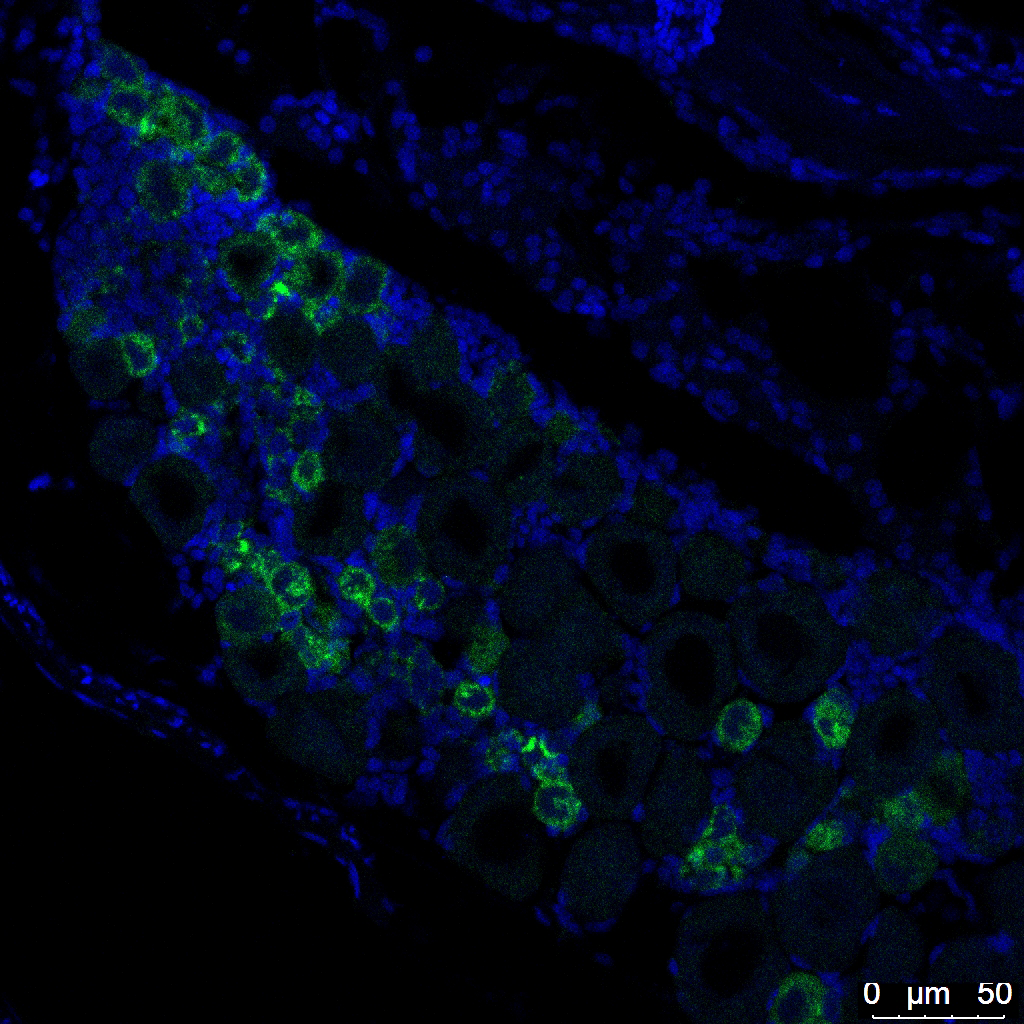

Supplement: Supplementary file 11 — Figure Source Data for Appendix Figures [file 44319_2026_775_MOESM11_ESM.zip › Source Data for Appendix Figure S1 3-7/Appendix Figure S7/Appendix Figure S7C/dnd1/Merge wnt8.tif]

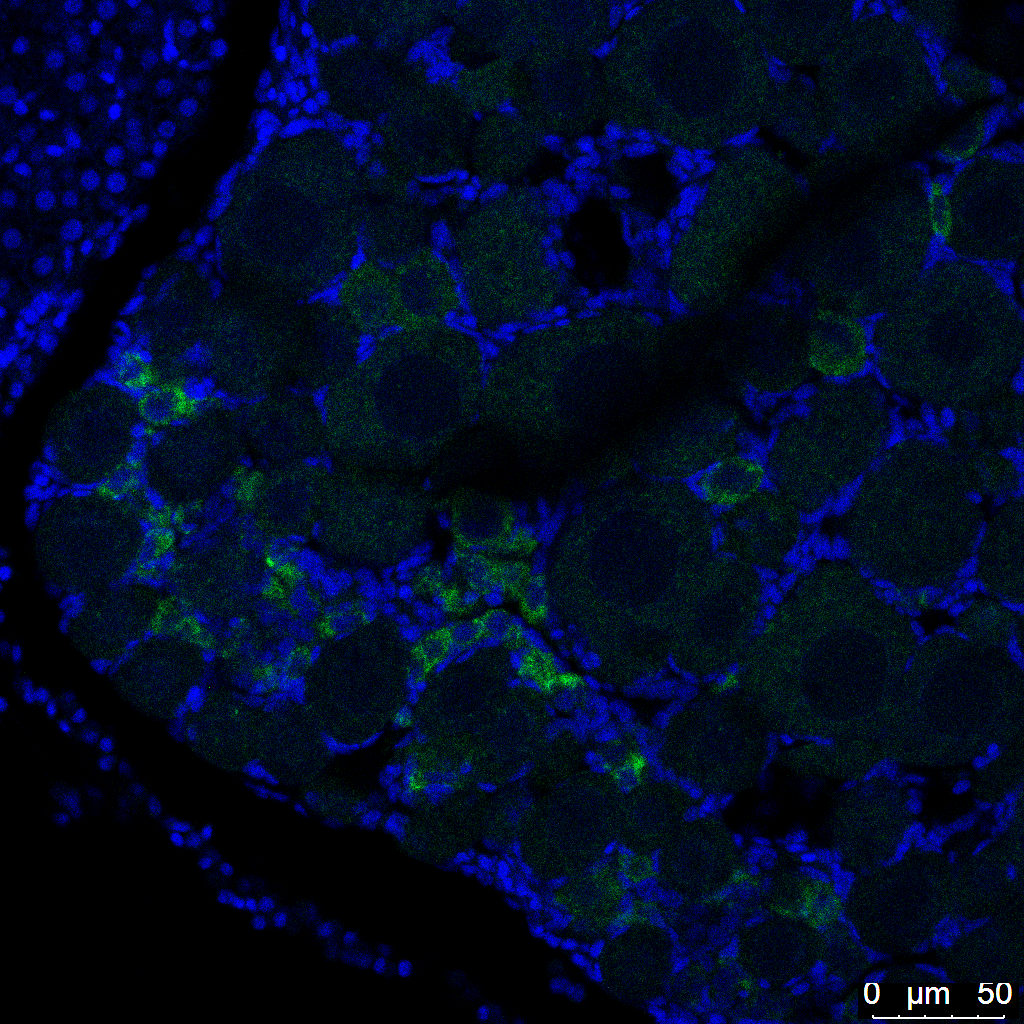

Supplement: Supplementary file 11 — Figure Source Data for Appendix Figures [file 44319_2026_775_MOESM11_ESM.zip › Source Data for Appendix Figure S1 3-7/Appendix Figure S7/Appendix Figure S7C/dnd1/Merge WT.tif]

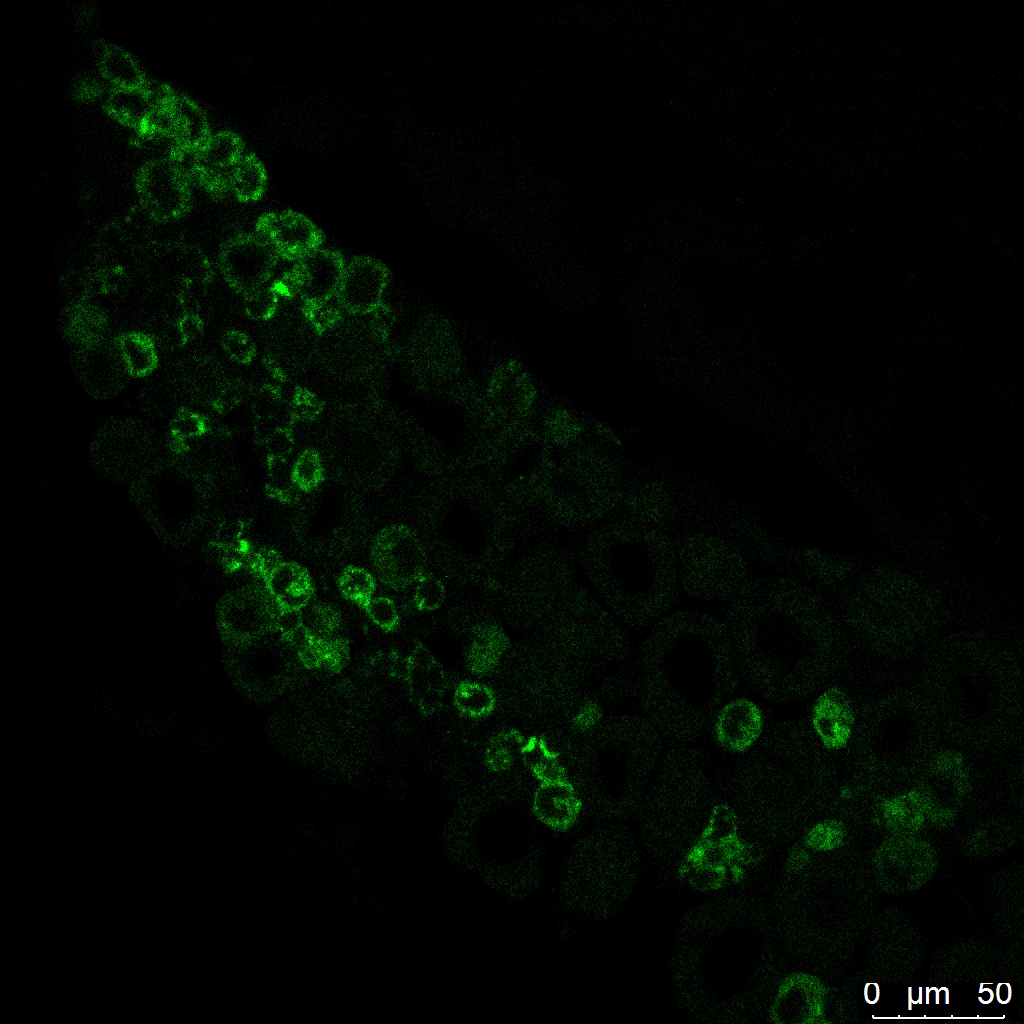

Supplement: Supplementary file 11 — Figure Source Data for Appendix Figures [file 44319_2026_775_MOESM11_ESM.zip › Source Data for Appendix Figure S1 3-7/Appendix Figure S7/Appendix Figure S7C/dnd1/tdrd9 wnt8.tif]

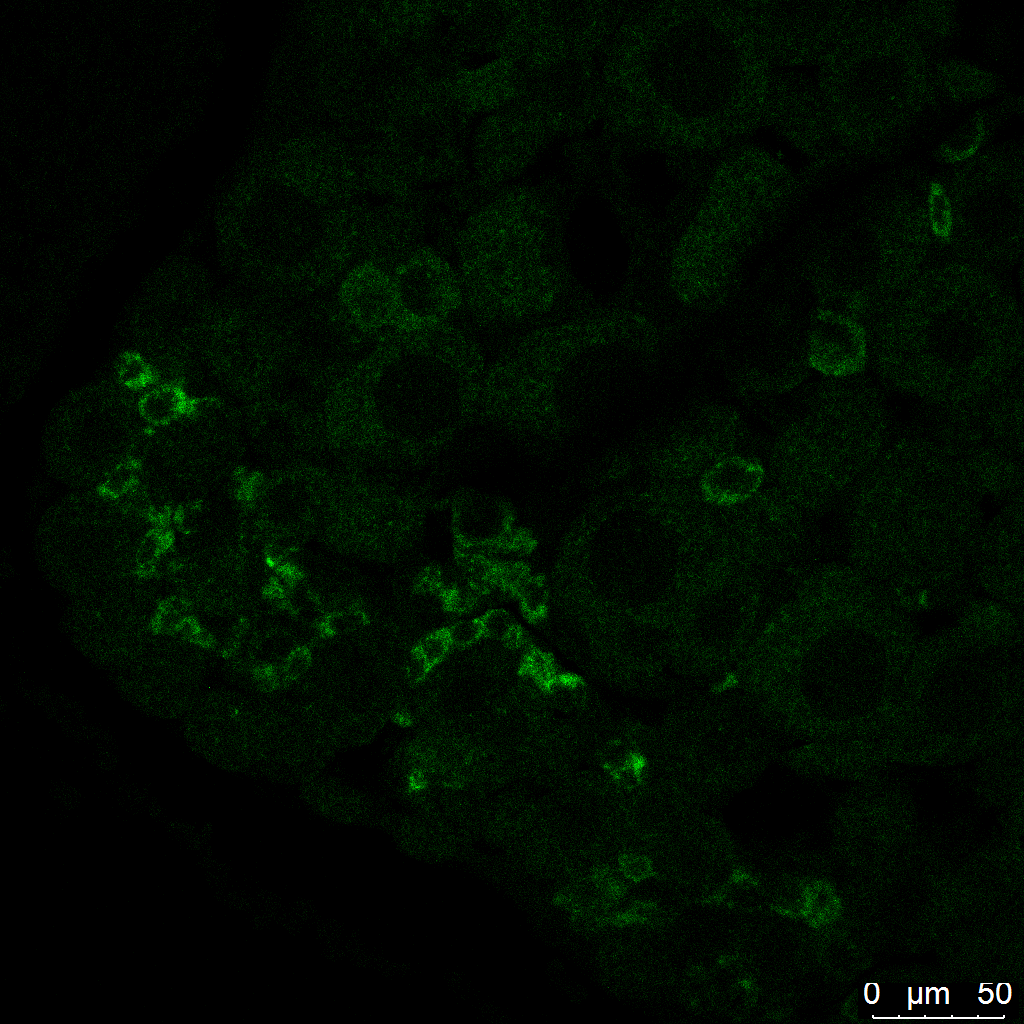

Supplement: Supplementary file 11 — Figure Source Data for Appendix Figures [file 44319_2026_775_MOESM11_ESM.zip › Source Data for Appendix Figure S1 3-7/Appendix Figure S7/Appendix Figure S7C/dnd1/tdrd9 WT.tif]

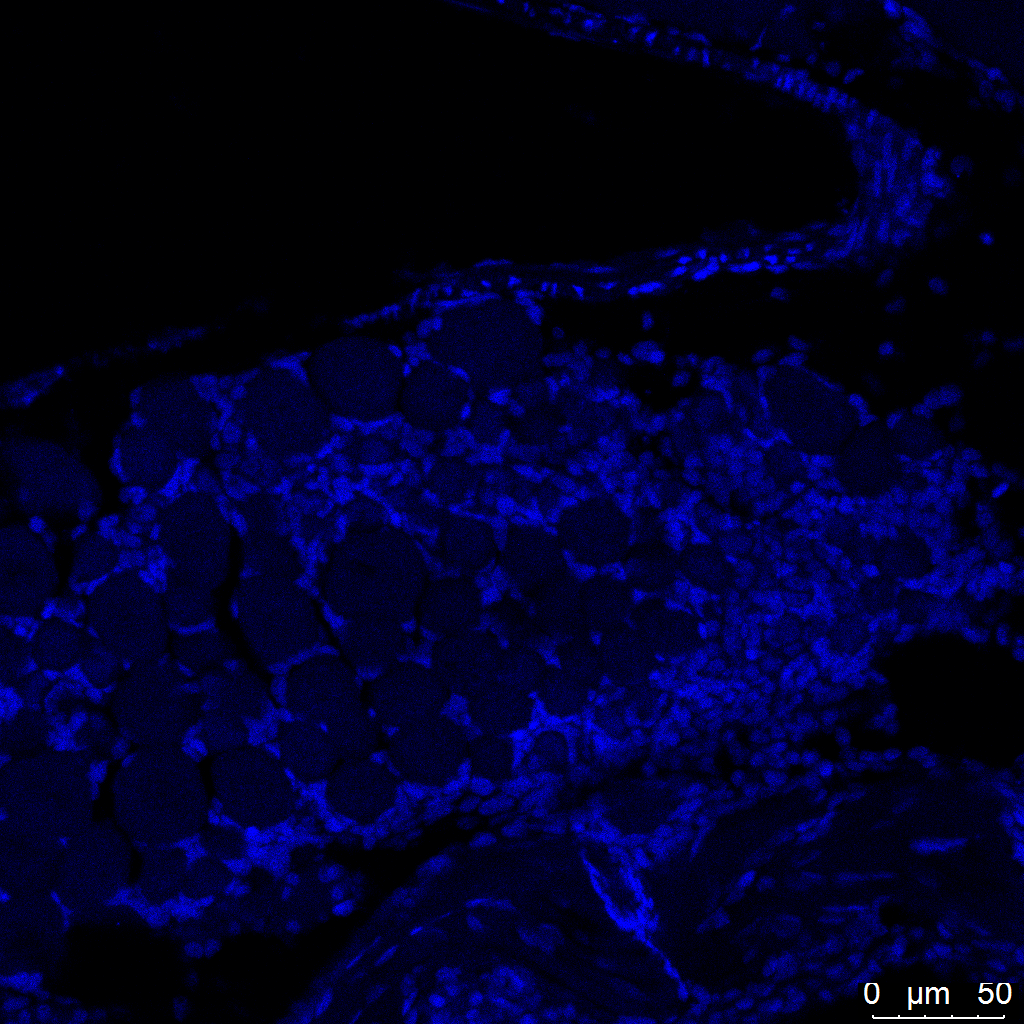

Supplement: Supplementary file 11 — Figure Source Data for Appendix Figures [file 44319_2026_775_MOESM11_ESM.zip › Source Data for Appendix Figure S1 3-7/Appendix Figure S7/Appendix Figure S7C/piwil1/DAPI wnt8.tif]

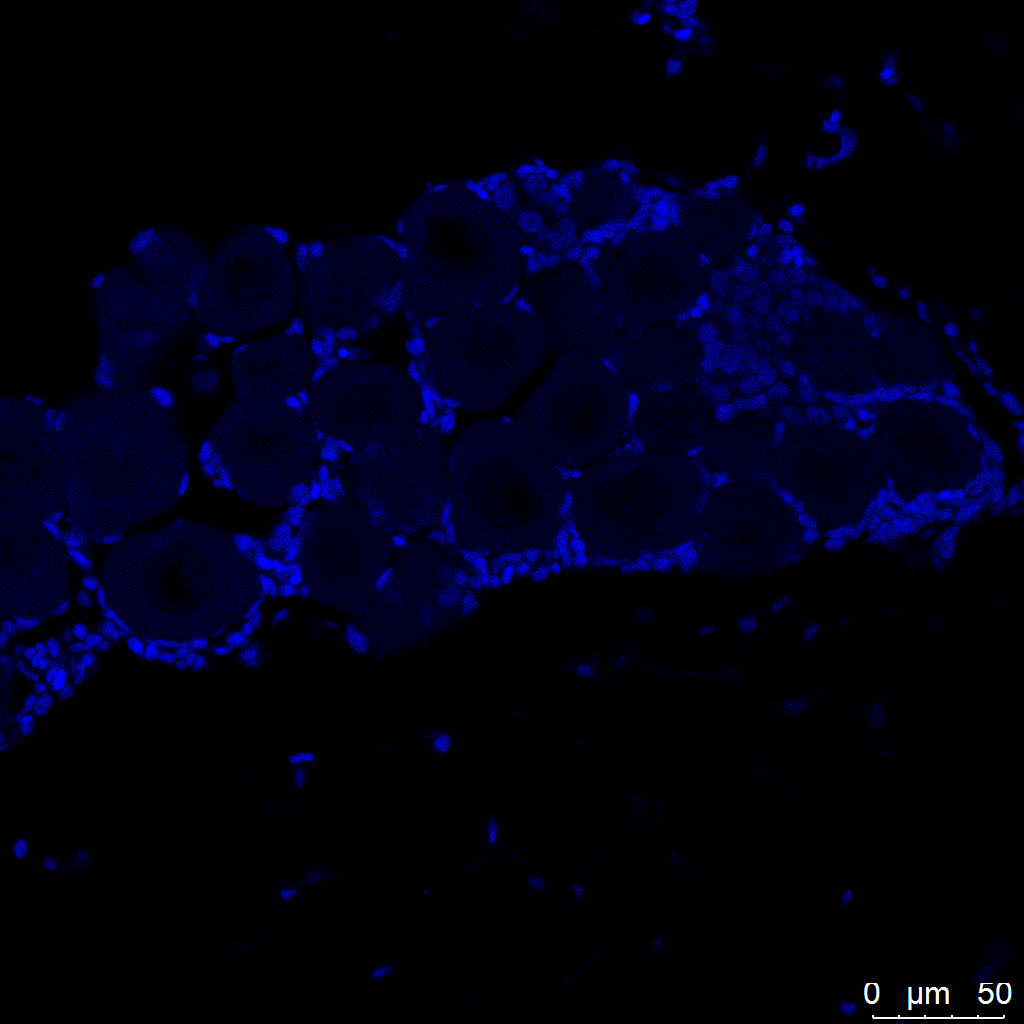

Supplement: Supplementary file 11 — Figure Source Data for Appendix Figures [file 44319_2026_775_MOESM11_ESM.zip › Source Data for Appendix Figure S1 3-7/Appendix Figure S7/Appendix Figure S7C/piwil1/DAPI WT.tif]

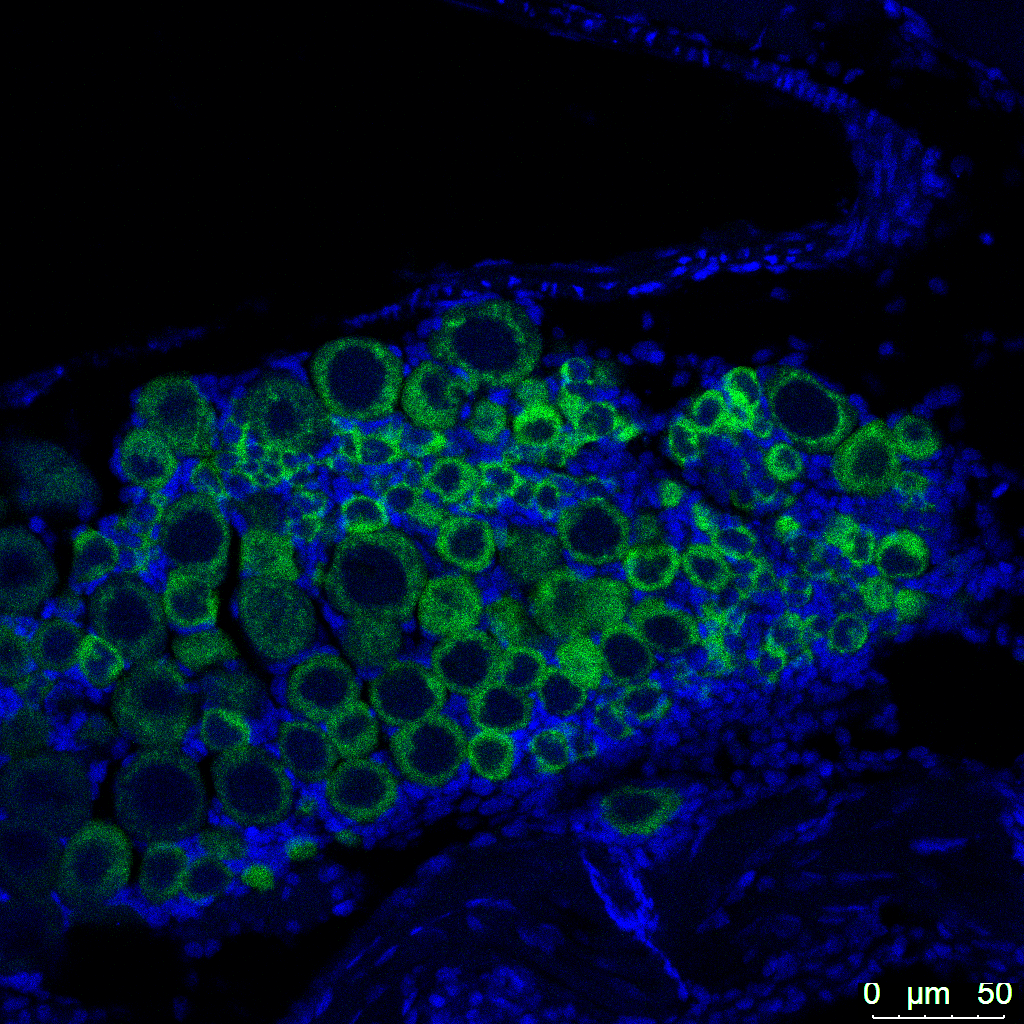

Supplement: Supplementary file 11 — Figure Source Data for Appendix Figures [file 44319_2026_775_MOESM11_ESM.zip › Source Data for Appendix Figure S1 3-7/Appendix Figure S7/Appendix Figure S7C/piwil1/Merge wnt8.tif]

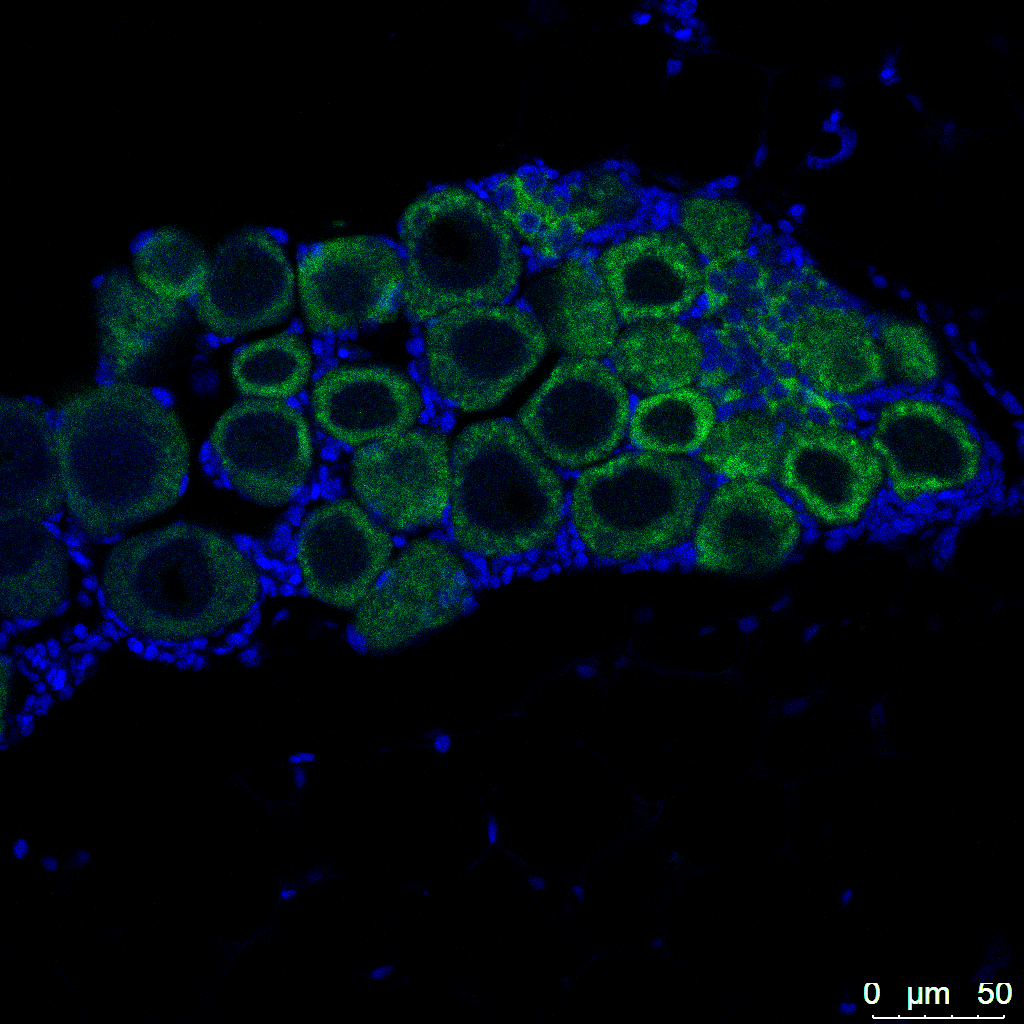

Supplement: Supplementary file 11 — Figure Source Data for Appendix Figures [file 44319_2026_775_MOESM11_ESM.zip › Source Data for Appendix Figure S1 3-7/Appendix Figure S7/Appendix Figure S7C/piwil1/Merge WT.tif]

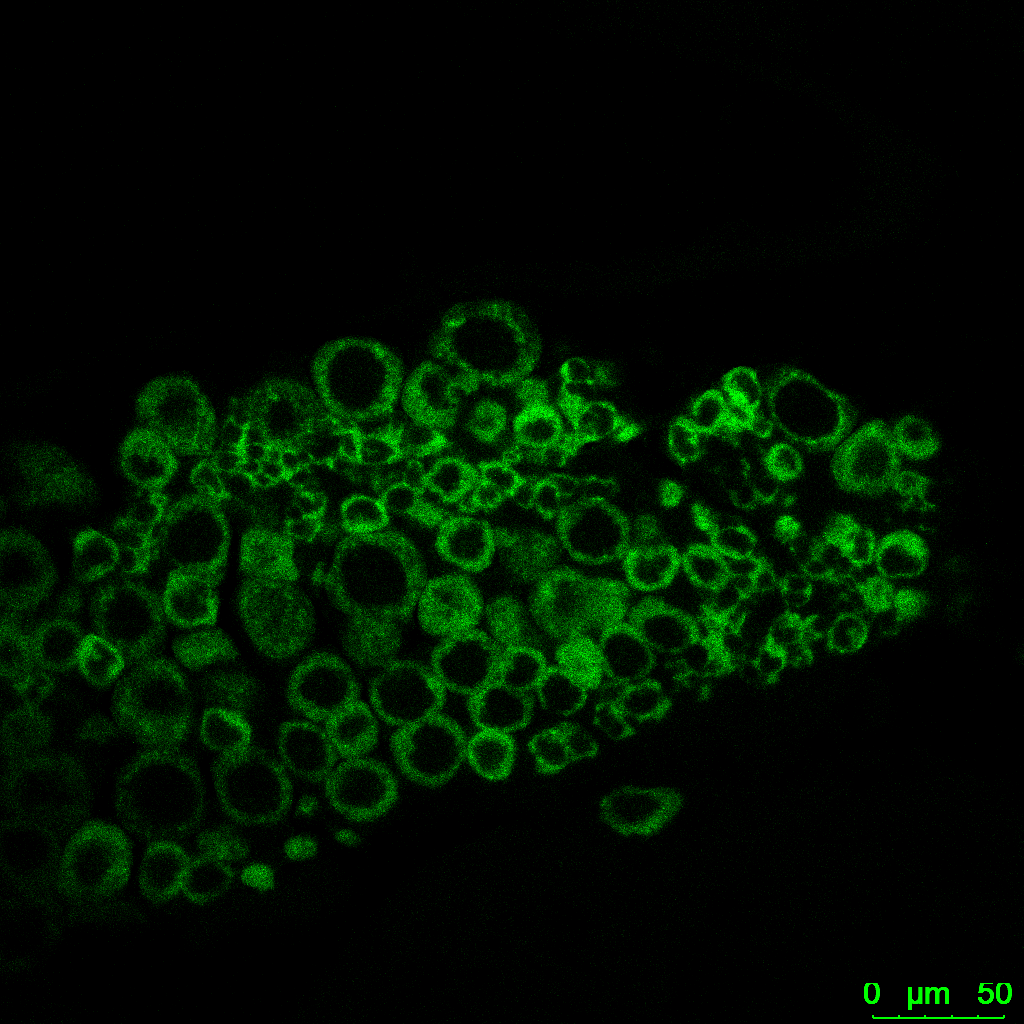

Supplement: Supplementary file 11 — Figure Source Data for Appendix Figures [file 44319_2026_775_MOESM11_ESM.zip › Source Data for Appendix Figure S1 3-7/Appendix Figure S7/Appendix Figure S7C/piwil1/piwil1 wnt8.tif]

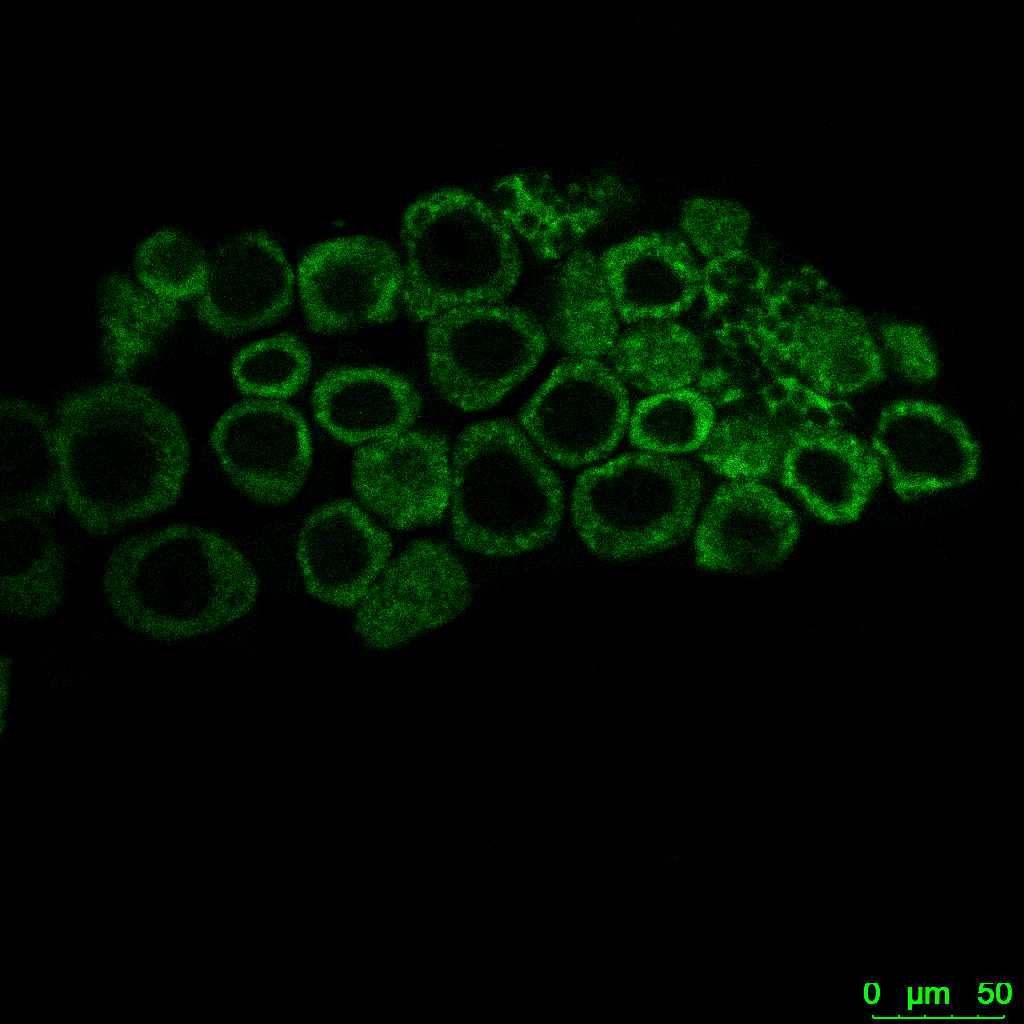

Supplement: Supplementary file 11 — Figure Source Data for Appendix Figures [file 44319_2026_775_MOESM11_ESM.zip › Source Data for Appendix Figure S1 3-7/Appendix Figure S7/Appendix Figure S7C/piwil1/piwil1 WT.tif]

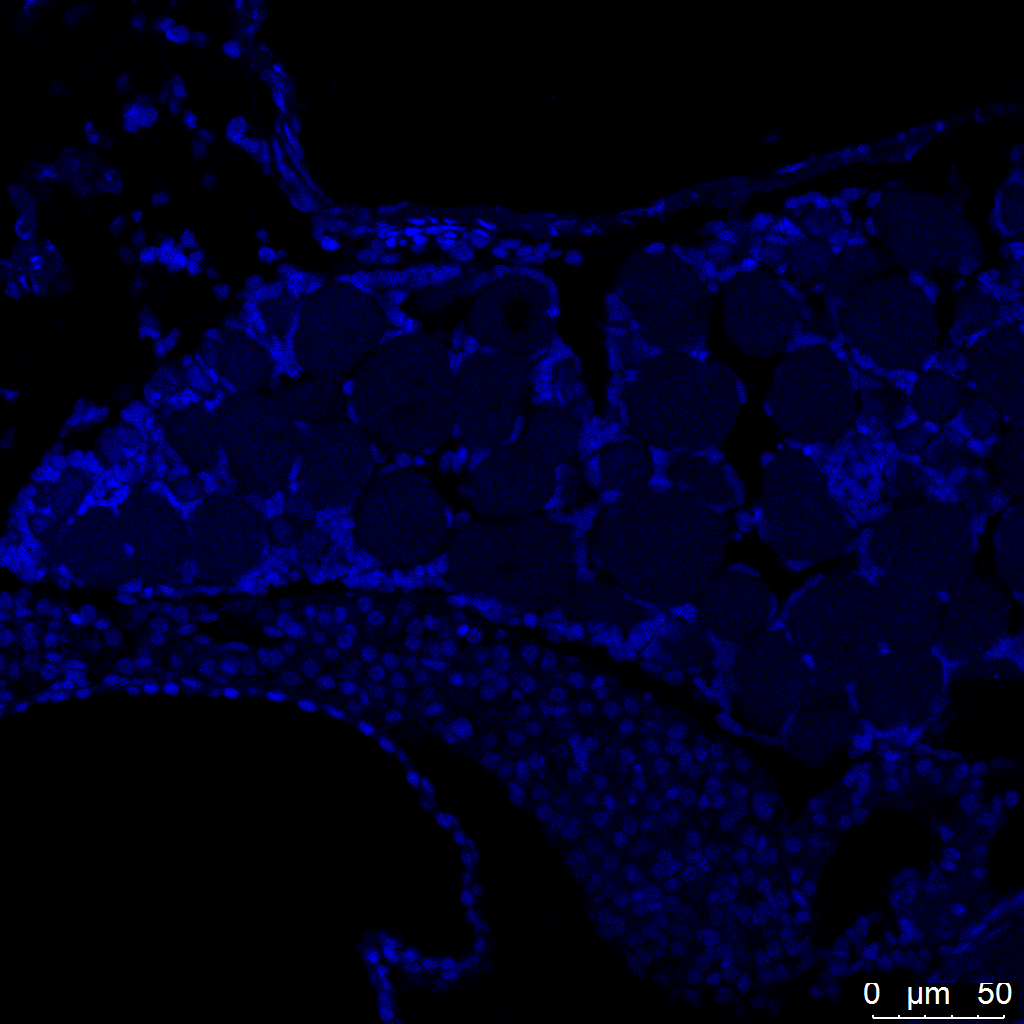

Supplement: Supplementary file 11 — Figure Source Data for Appendix Figures [file 44319_2026_775_MOESM11_ESM.zip › Source Data for Appendix Figure S1 3-7/Appendix Figure S7/Appendix Figure S7C/piwil2/DAPI wnt8.tif]

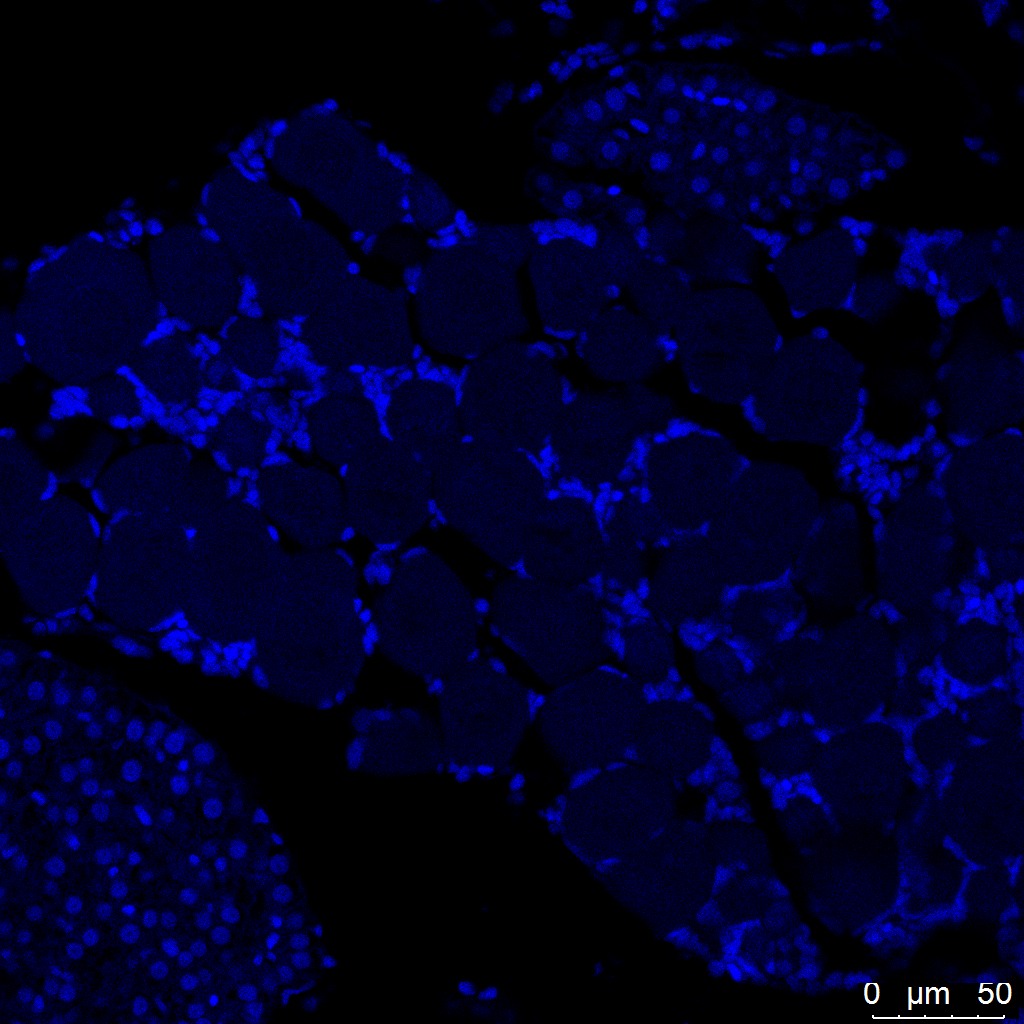

Supplement: Supplementary file 11 — Figure Source Data for Appendix Figures [file 44319_2026_775_MOESM11_ESM.zip › Source Data for Appendix Figure S1 3-7/Appendix Figure S7/Appendix Figure S7C/piwil2/DAPI WT.tif]

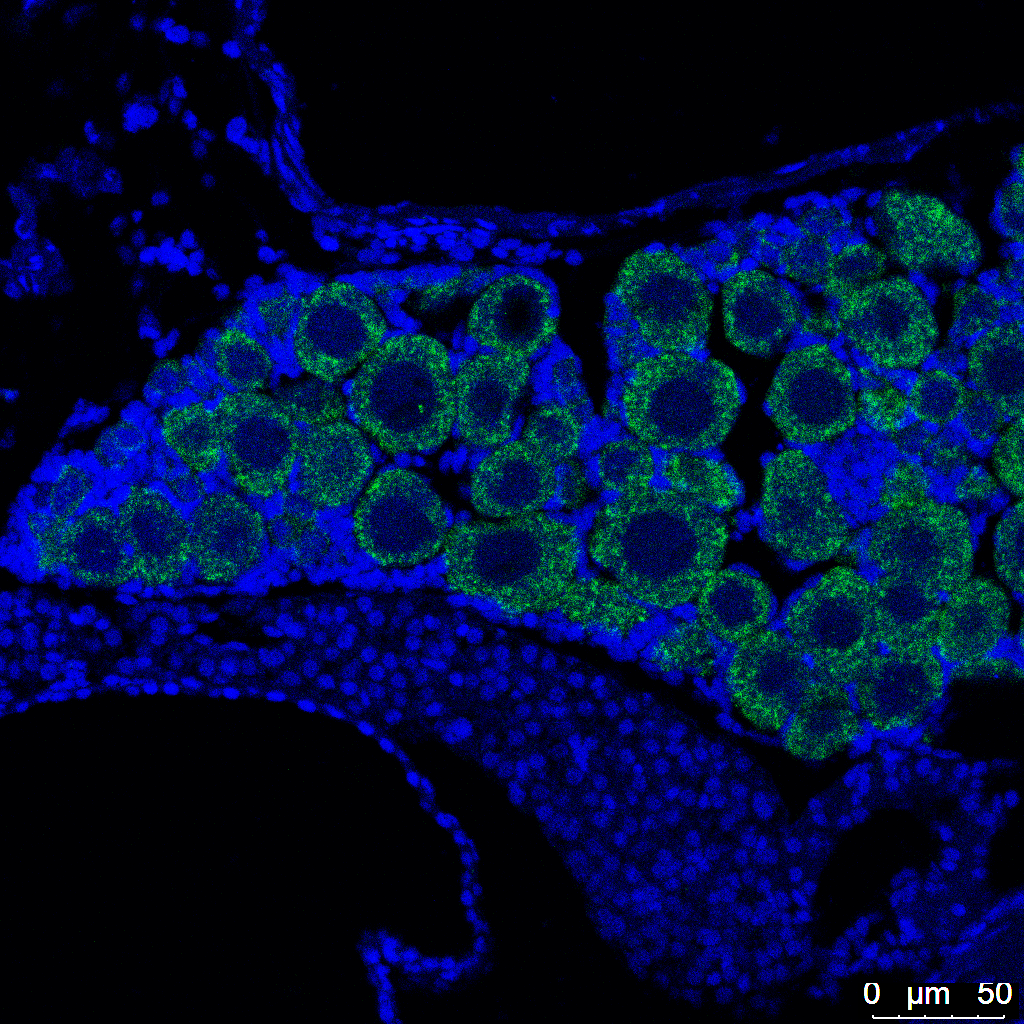

Supplement: Supplementary file 11 — Figure Source Data for Appendix Figures [file 44319_2026_775_MOESM11_ESM.zip › Source Data for Appendix Figure S1 3-7/Appendix Figure S7/Appendix Figure S7C/piwil2/Merge wnt8.tif]

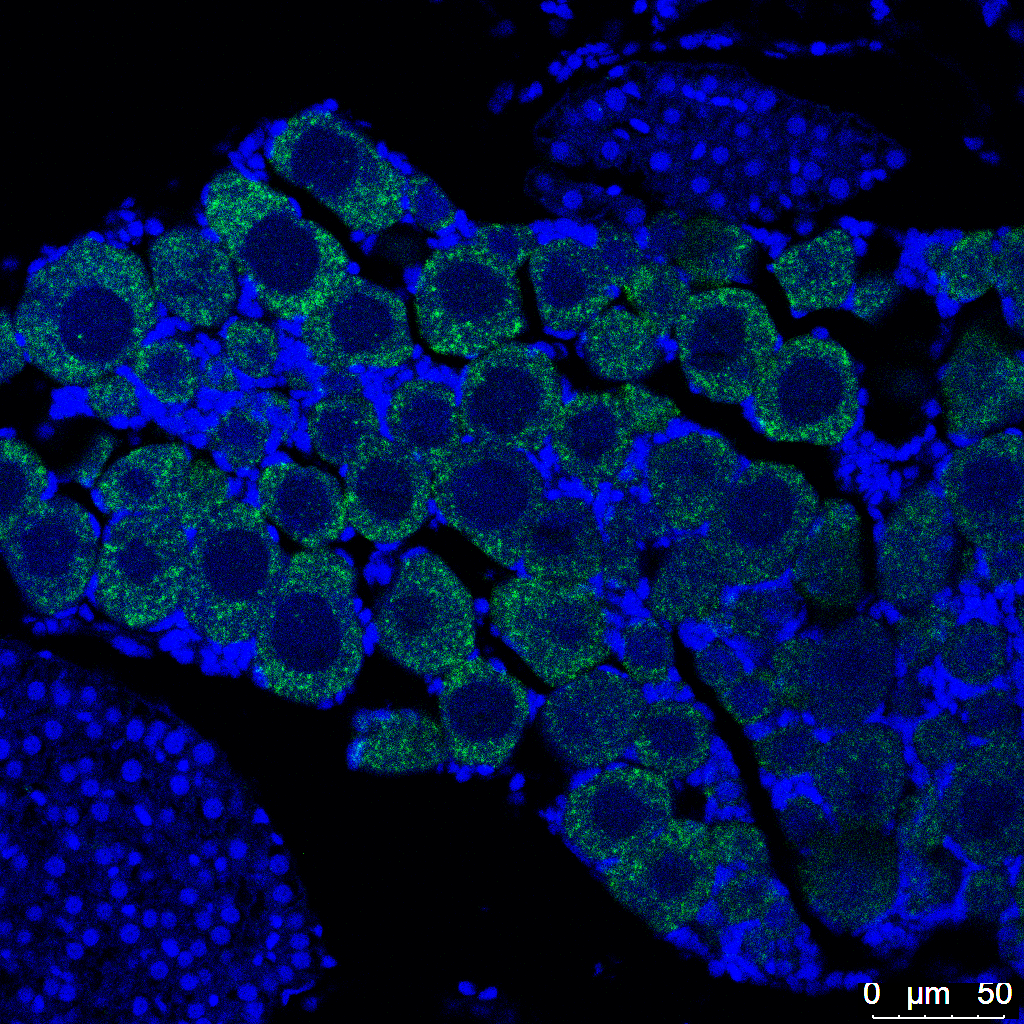

Supplement: Supplementary file 11 — Figure Source Data for Appendix Figures [file 44319_2026_775_MOESM11_ESM.zip › Source Data for Appendix Figure S1 3-7/Appendix Figure S7/Appendix Figure S7C/piwil2/Merge WT.tif]

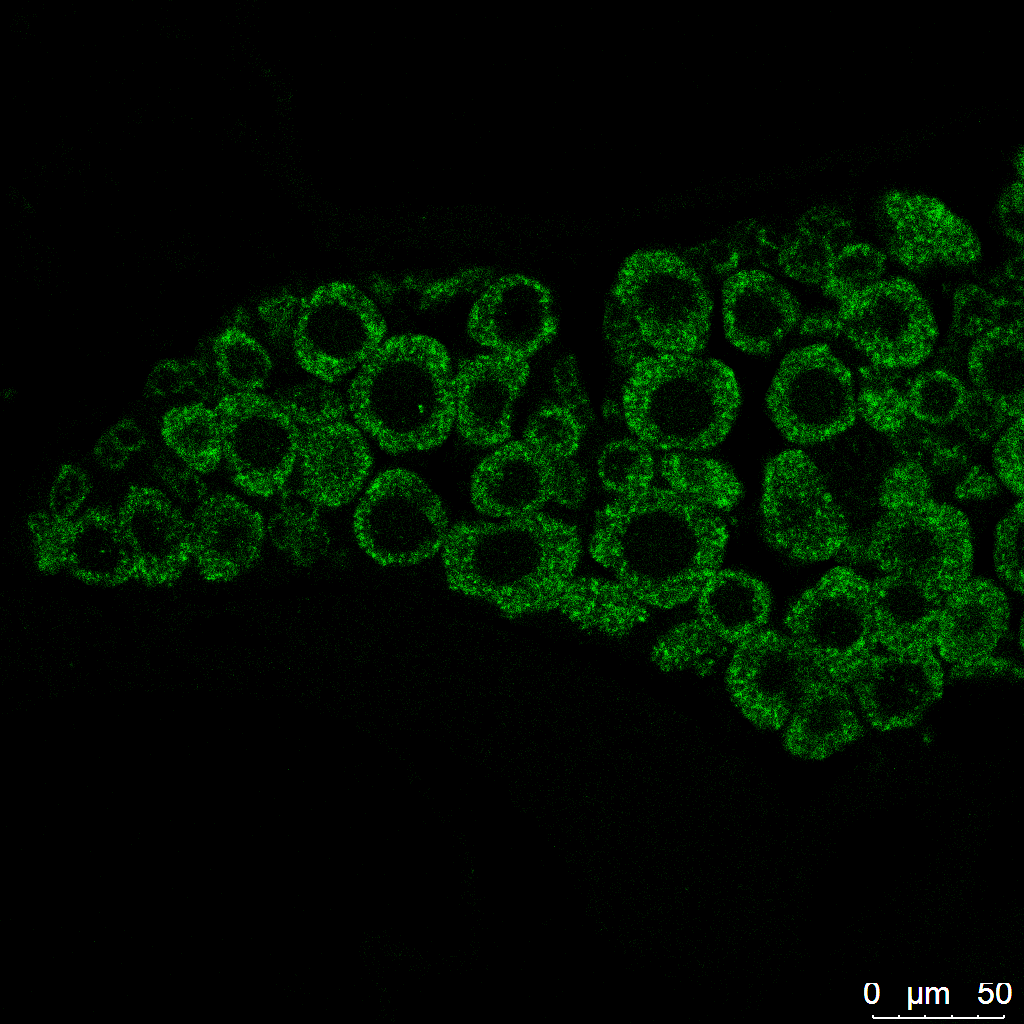

Supplement: Supplementary file 11 — Figure Source Data for Appendix Figures [file 44319_2026_775_MOESM11_ESM.zip › Source Data for Appendix Figure S1 3-7/Appendix Figure S7/Appendix Figure S7C/piwil2/piwil2 wnt8.tif]

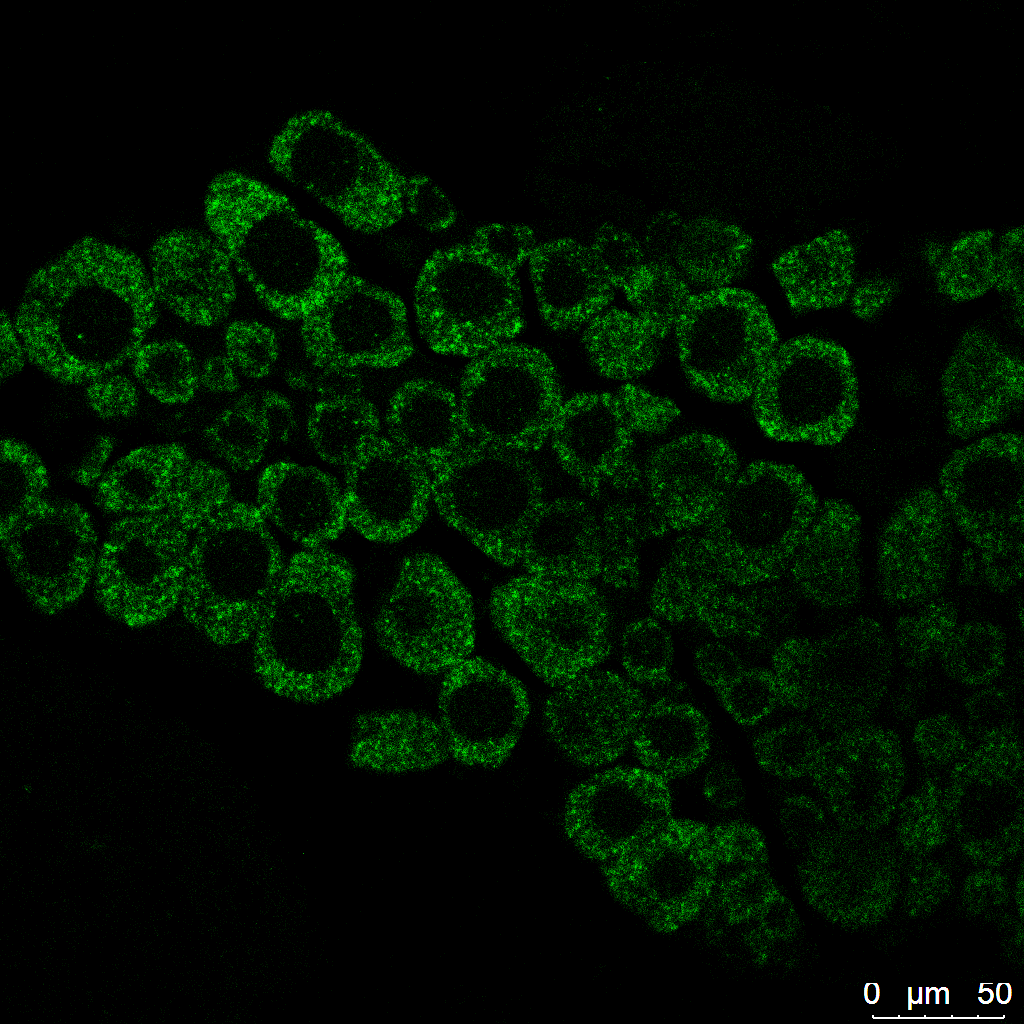

Supplement: Supplementary file 11 — Figure Source Data for Appendix Figures [file 44319_2026_775_MOESM11_ESM.zip › Source Data for Appendix Figure S1 3-7/Appendix Figure S7/Appendix Figure S7C/piwil2/piwil2 WT.tif]

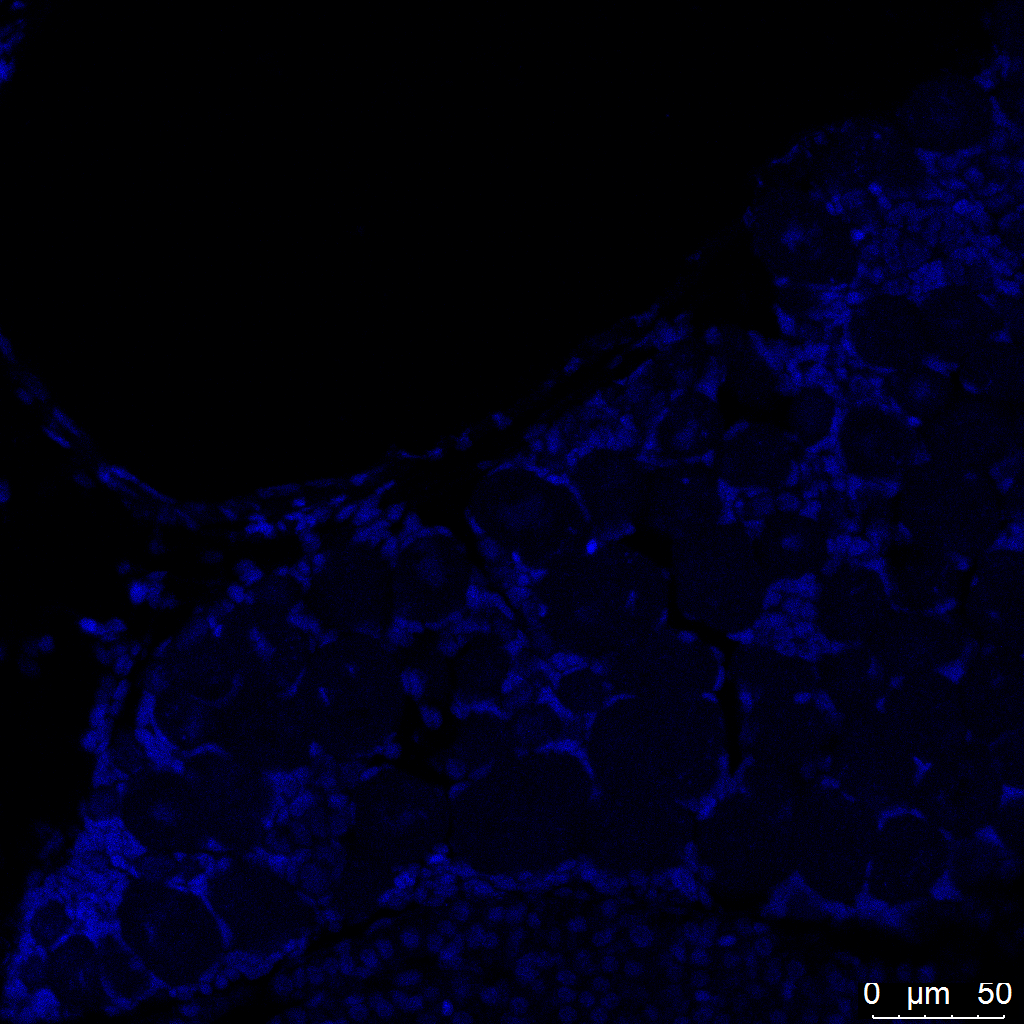

Supplement: Supplementary file 11 — Figure Source Data for Appendix Figures [file 44319_2026_775_MOESM11_ESM.zip › Source Data for Appendix Figure S1 3-7/Appendix Figure S7/Appendix Figure S7C/tdrd1/DAPI wnt8.tif]

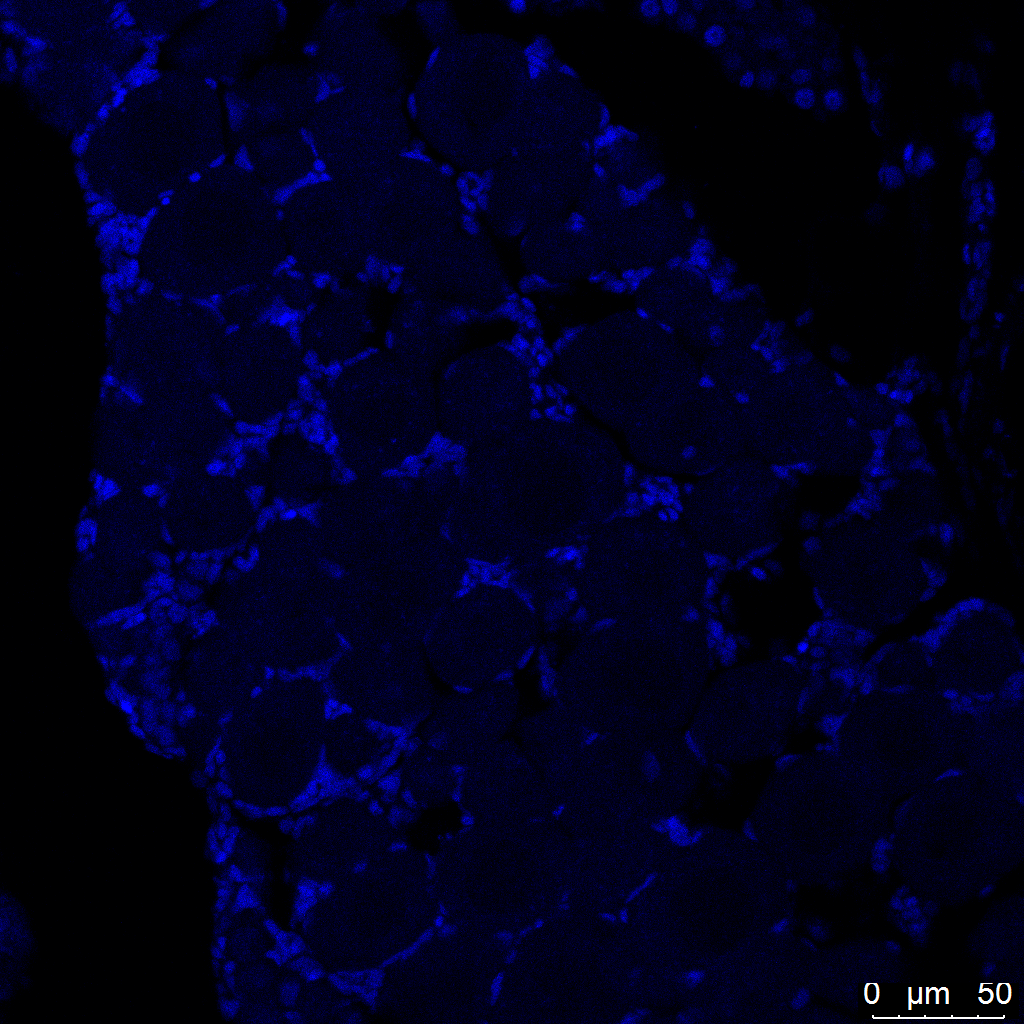

Supplement: Supplementary file 11 — Figure Source Data for Appendix Figures [file 44319_2026_775_MOESM11_ESM.zip › Source Data for Appendix Figure S1 3-7/Appendix Figure S7/Appendix Figure S7C/tdrd1/DAPI WT.tif]

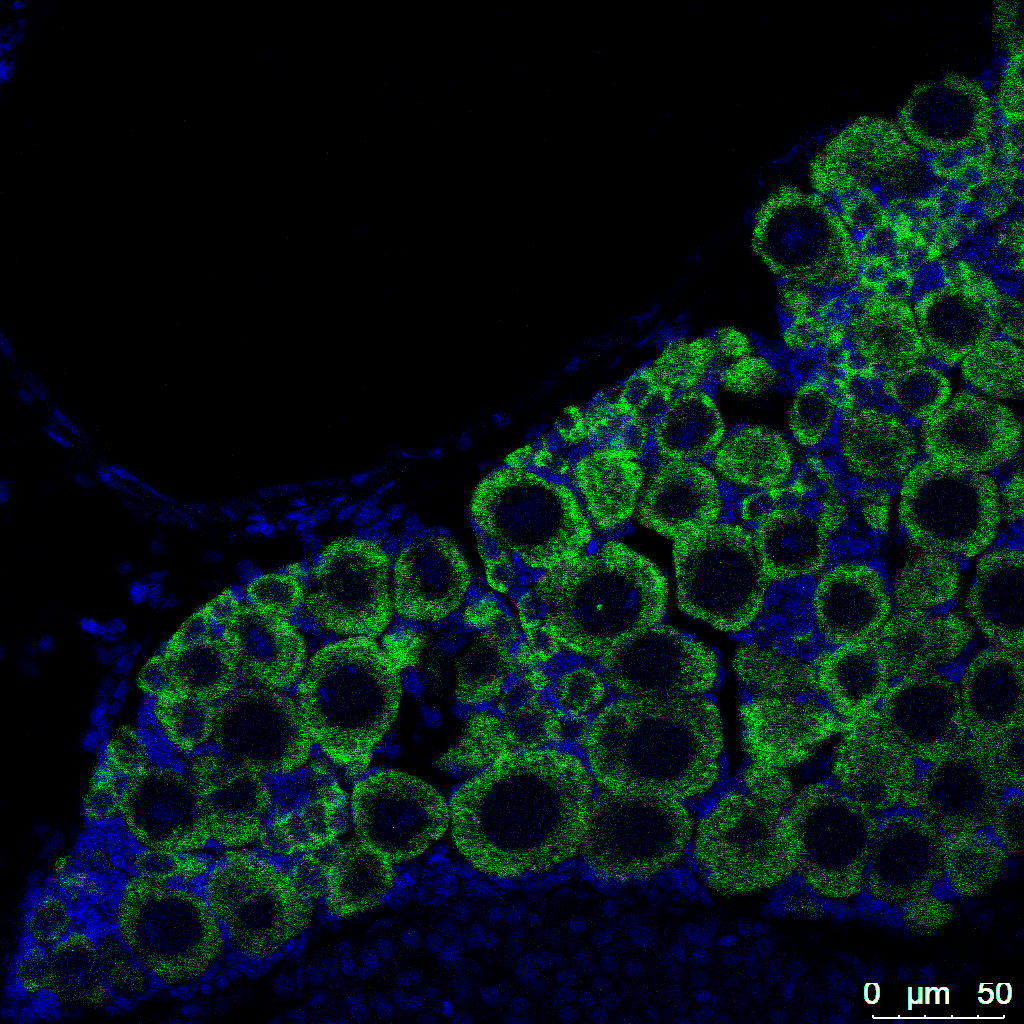

Supplement: Supplementary file 11 — Figure Source Data for Appendix Figures [file 44319_2026_775_MOESM11_ESM.zip › Source Data for Appendix Figure S1 3-7/Appendix Figure S7/Appendix Figure S7C/tdrd1/Merge wnt8.tif]
