# Supplementary material for: Stn1 promotes zebrafish oocyte development via amplifying Wnt/β-catenin signaling
Source: EMBO Rep. 2026 Apr 17;27(12):3252–76. doi: 10.1038/s44319-026-00775-8 (PMC13304171; doi:10.1038/s44319-026-00775-8)
Supplement: Supplementary file 12 — Expanded View Figures [file 44319_2026_775_MOESM12_ESM.pdf]

Expanded View Figures

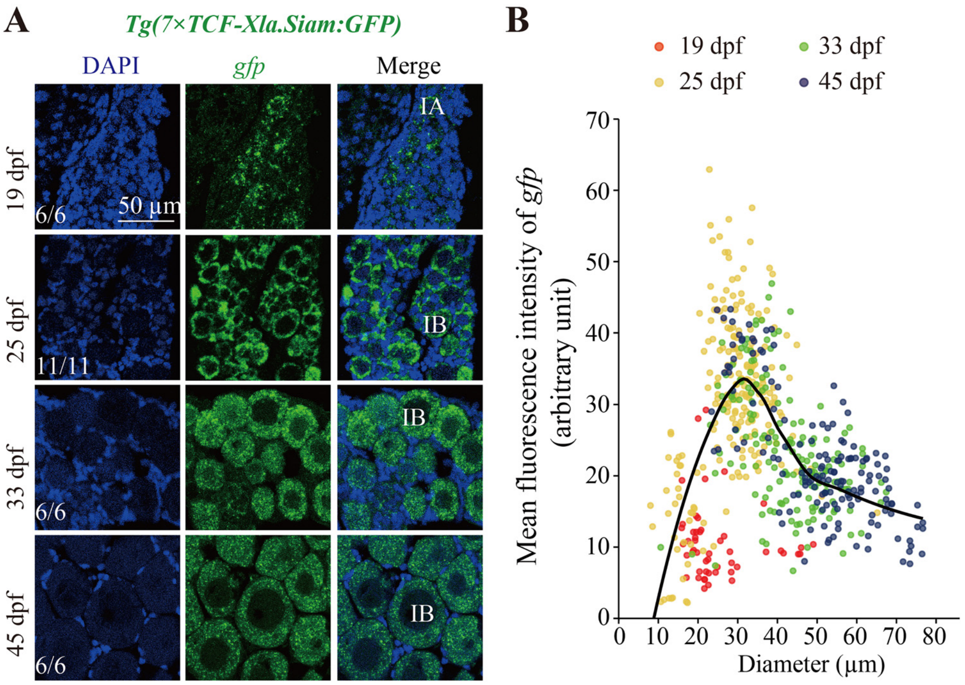

Figure EV1. The *gfp* mRNA levels in the gonads of juvenile zebrafish with a *Tg(7×TCF-Xla.Siam:GFP)* transgenic background during growth processes.

(**A**) Representative confocal images of gonads of female fish with a *Tg(7×TCF-Xla.Siam:GFP)* transgenic background at the indicated time points following staining for *gfp* mRNA. IA stage IA, IB stage IB. Scale bar: 50 µm. The proportion of sections with the indicated phenotypes is shown in the bottom left corner of each panel. Each section was obtained from an individual zebrafish. (**B**) Quantitative results from images shown in (**A**). Dot plot showing the mean fluorescence of *gfp* mRNA of germ cells. Each data point represents an individual oocyte. The number of zebrafish counted at different time points was 6 at 19 dpf, 11 at 25 dpf, 6 at 33 dpf, and 6 at 45 dpf. Source data are available online for this figure.

 © The Author(s)

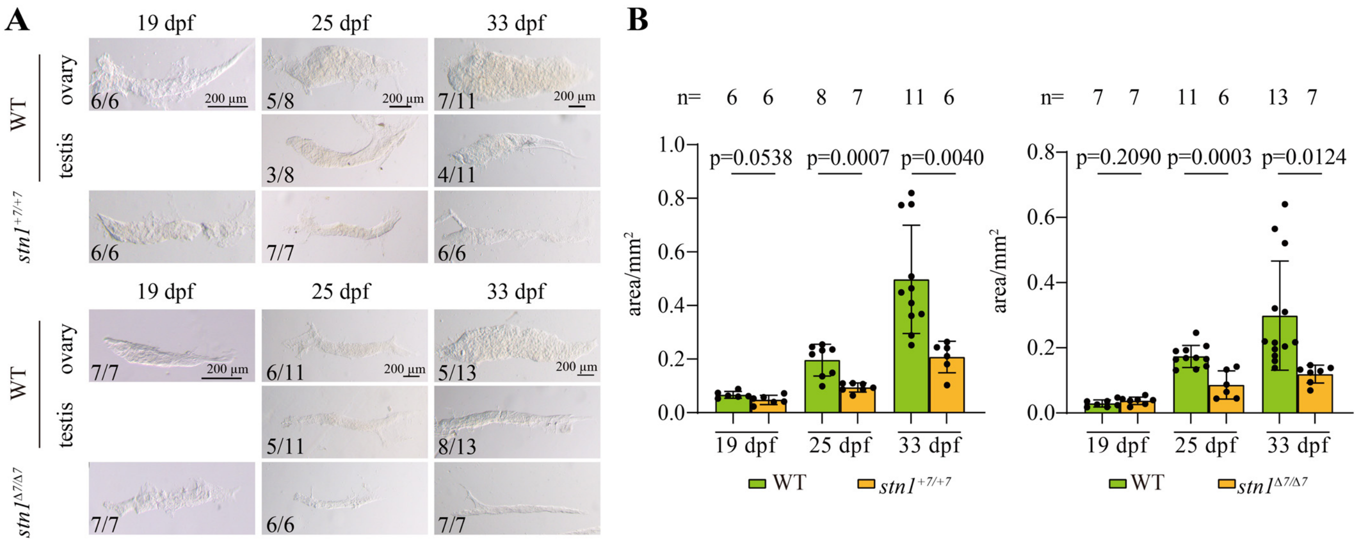

Figure EV2. Loss of Stn1 impairs gonad development.

(**A**) Representative gonads from sibling and *stn1* mutant zebrafish at the indicated time points. The proportion of gonads with the indicated phenotypes is shown in the bottom left corner of each panel. Scale bar: 200 µm. (**B**) Quantitative results from images shown in (**A**). Each data point represents an individual gonad, and the total numbers (*n*) are given at the top of columns. Values are represented as means ± SD; Unpaired *t* test, two-tailed. Source data are available online for this figure.

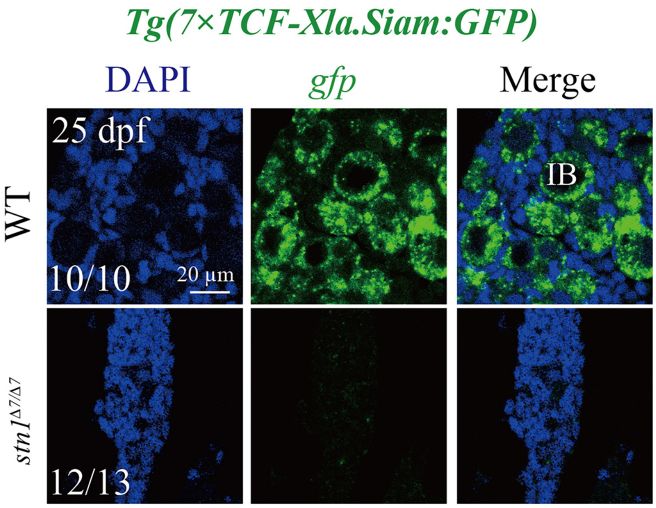

Figure EV3. Loss of Stn1 reduces *gfp* mRNA levels in germ cells of juvenile zebrafish with a *Tg(7×TCF-Xla.Siam:GFP)* transgenic background.

Representative confocal images of gonads from siblings and *stn1* mutants at 25 dpf. Gonads of juvenile fish at 25 dpf with a *Tg(7×TCF-Xla.Siam:GFP)* transgenic background were stained with *gfp* mRNA. IB stage IB. Scale bar: 20 µm. The frequency of juvenile zebrafish with the indicated phenotypes is shown in the bottom left corner of each panel. Source data are available online for this figure.

 © The Author(s)

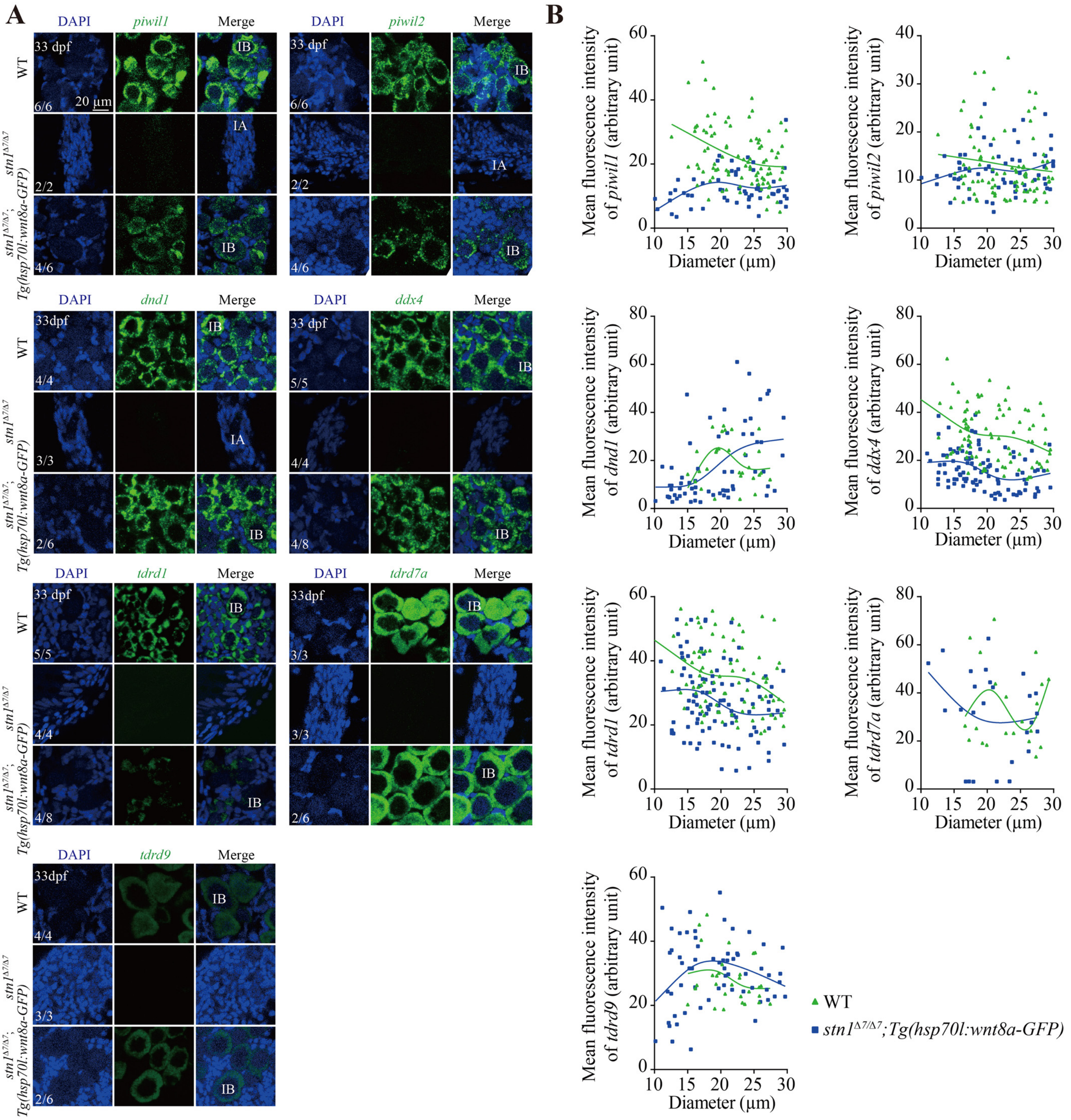

Figure EV4. Temporally inducible expression of Wnt8a partially restores the expression of germ cell-specific Wnt target genes in *stn1* mutants.

(**A**) Representative confocal images of gonads from wild-type siblings and *stn1* mutants with or without *Tg(hsp70l:wnt8a-GFP)* genetic background at 33 dpf. Gonads from juvenile fish of the indicated genotypes at 33 dpf were stained for the mRNA of each specified gene. IA stage IA, IB stage IB. Scale bar: 20 µm. The frequency of the indicated phenotypes is shown in the bottom left corner of each panel. (**B**) Quantitative results from images shown in (**A**). Each data point represents an individual oocyte. The counts of wild-type siblings, and *stn1*$^{\Delta7/\Delta7}$; *Tg(hsp70l:wnt8a-GFP)* zebrafish were as follows: 4, and 6 for *dnd1* and *tdrd9*; 5, and 8 for *ddx4* and *tdrd1*; 6, and 6 for *piwil1* and *piwil2*; and 3, and 6 for *tdrd7a*, respectively. Source data are available online for this figure.
