# Supplementary material for: Stn1 promotes zebrafish oocyte development via amplifying Wnt/β-catenin signaling
Source: EMBO Rep. 2026 Apr 17;27(12):3252–76. doi: 10.1038/s44319-026-00775-8 (PMC13304171; doi:10.1038/s44319-026-00775-8)
Supplement: Supplementary file 2 — Peer Review File [file 44319_2026_775_MOESM2_ESM.pdf]

EMBO reports

Stn1 promotes zebrafish oocyte development via amplifying Wnt/β-catenin signaling

Xin Zhang, Yanpeng Zhai, Caixia Wang, Yuxue He, Yuxin He, Bo Wang, Chensong Huang, Aibo Sheng, Yan Bai, Xiaozhi Rong, and Jianfeng Zhou

Corresponding author(s): Jianfeng Zhou (jfzhou@ouc.edu.cn) , Xiaozhi Rong (rongxiaozhi@ouc.edu.cn)

Review Timeline:

Submission Date:	6th Apr 25
Editorial Decision:	12th May 25
Revision Received:	28th Nov 25
Editorial Decision:	12th Mar 26
Revision Received:	18th Mar 26
Accepted:	23rd Mar 26

Editor: Martina Rembold

Transaction Report:

(Note: With the exception of the correction of typographical or spelling errors that could be a source of ambiguity, letters and reports are not edited. Depending on transfer agreements, referee reports obtained elsewhere may or may not be included in this compilation. Referee reports are anonymous unless the Referee chooses to sign their reports.)

1st Editorial Decision **12th May 2025**

Dear Dr. Zhou

Thank you for the submission of your research manuscript to our journal. We have now received the full set of referee reports that is copied below.

As you will see, the referees acknowledge that the findings are potentially interesting, but they also raise a number of concerns and have suggestions how to further strengthen the data. All concerns raised appear to be pertinent and should be addressed. In particular, Stn1's reported role in telomere biology and its potential contribution to germ cell maintenance must be addressed and investigated.

Given these constructive comments, we would like to invite you to revise your manuscript with the understanding that the referee concerns (as detailed above and in their reports) must be fully addressed and their suggestions taken on board. Please address all referee concerns in a complete point-by-point response. Acceptance of the manuscript will depend on a positive outcome of a second round of review. It is EMBO Reports policy to allow a single round of revision only and acceptance or rejection of the manuscript will therefore depend on the completeness of your responses included in the next, final version of the manuscript.

We realize that it is difficult to revise to a specific deadline. In the interest of protecting the conceptual advance provided by the work, we recommend a revision within 3 months (August 12th). Please discuss the revision progress ahead of this time with the editor if you require more time to complete the revisions.

I am also happy to discuss the revision further via e-mail or a video call, if you wish.

You can either publish the study as a short report or as a full article. For short reports, the revised manuscript should not exceed 27,000 characters (including spaces but excluding materials & methods and references) and 5 main plus 5 expanded view figures. The results and discussion sections must further be combined, which will help to shorten the manuscript text by eliminating some redundancy that is inevitable when discussing the same experiments twice. For a normal article there are no length limitations, but it should have more than 5 main figures and the results and discussion sections must be separate. In both cases, the entire materials and methods must be included in the main manuscript file.

================

IMPORTANT NOTE:

We perform an initial quality control of all revised manuscripts before re-review. Your manuscript will FAIL this control and the handling will be delayed IN CASE the following APPLIES:

1) A data availability section providing access to data deposited in public databases is missing. If you have not deposited any data, please add a sentence to the data availability section that explains that.

2) Your manuscript contains statistics and error bars based on n=2. Please use scatter blots in these cases. No statistics should be calculated if n=2.

When submitting your revised manuscript, please carefully review the instructions that follow below. Failure to include requested items will delay the evaluation of your revision.

================

When submitting your revised manuscript, we will require:

1) a .docx formatted version of the manuscript text (including legends for main figures, EV figures and tables). Please make sure that the changes are highlighted to be clearly visible.

2) individual production quality figure files as .eps, .tif, .jpg (one file per figure).
Please download our Figure Preparation Guidelines (figure preparation pdf) from our Author Guidelines pages https://www.embopress.org/page/journal/14693178/authorguide for more info on how to prepare your figures.

3) a .docx formatted letter INCLUDING the reviewers' reports and your detailed point-by-point responses to their comments. As part of the EMBO Press transparent editorial process, the point-by-point response is part of the Review Process File (RPF), which will be published alongside your paper.

4) a complete author checklist, which you can download from our author guidelines (<https://www.embopress.org/page/journal/14693178/authorguide>). Please insert information in the checklist that is also reflected in the manuscript. The completed author checklist will also be part of the RPF.

5) Please note that all corresponding authors are required to supply an ORCID ID for their name upon submission of a revised manuscript (<https://orcid.org/>). Please find instructions on how to link your ORCID ID to your account in our manuscript tracking system in our Author guidelines (<https://www.embopress.org/page/journal/14693178/authorguide#authorshipguidelines>)

6) We replaced Supplementary Information with Expanded View (EV) Figures and Tables that are collapsible/expandable online. A maximum of 5 EV Figures can be typeset. EV Figures should be cited as 'Figure EV1, Figure EV2" etc... in the text and their respective legends should be included in the main text after the legends of regular figures.

- For the figures that you do NOT wish to display as Expanded View figures, they should be bundled together with their legends in a single PDF file called *Appendix*, which should start with a short Table of Content. Appendix figures should be referred to in the main text as: "Appendix Figure S1, Appendix Figure S2" etc. See detailed instructions regarding expanded view here: <https://www.embopress.org/page/journal/14693178/authorguide#expandedview>

- Additional Tables/Datasets should be labeled and referred to as Table EV1, Dataset EV1, etc. Legends have to be provided in a separate tab in case of .xls files. Alternatively, the legend can be supplied as a separate text file (README) and zipped together with the Table/Dataset file.

7) Data availability section: please remove the current text on data sharing and instead place the description and access to the RNA-seq data at the Sequence Read Archive there, which you currently have in the methods under "RNA-seq...".
See also <https://www.embopress.org/page/journal/14693178/authorguide#dataavailability>

8) At EMBO Press we ask authors to provide source data for the main manuscript figures. You will receive a separate email with instructions for providing source data with your revised manuscript, including how to upload and organize the files.

Additional information on source data and instruction on how to label the files are available <https://www.embopress.org/page/journal/14693178/authorguide#sourcedata>

9) The journal requires a statement specifying whether or not authors have competing interests (defined as all potential or actual interests that could be perceived to influence the presentation or interpretation of an article). In case of competing interests, this must be specified in your disclosure statement. Further information: https://www.embopress.org/competing-interests

10) Figure legends and data quantification:
The following points must be specified in each figure legend:

- the name of the statistical test used to generate error bars and P values,
- the EXACT p-values,
- the number (n) of independent experiments (please specify technical or biological replicates) underlying each data point,
- the nature of the bars and error bars (s.d., s.e.m.)

- If the data are obtained from n {less than or equal to} 5, show the individual data points in addition to the SD or SEM.
- If the data are obtained from n {less than or equal to} 2, use scatter blots showing the individual data points.

Discussion of statistical methodology can be reported in the materials and methods section, but figure legends should contain a basic description of n, P and the test applied.

See also the guidelines for figure legend preparation:
https://www.embopress.org/page/journal/14693178/authorguide#figureformat

- Please also include scale bars in all microscopy images and define their size in the legend, not the image.

11) Our journal encourages inclusion of *data citations in the reference list* to directly cite datasets that were re-used and obtained from public databases. Data citations in the article text are distinct from normal bibliographical citations and should directly link to the database records from which the data can be accessed. In the main text, data citations are formatted as follows: "Data ref: Smith et al, 2001" or "Data ref: NCBI Sequence Read Archive PRJNA342805, 2017". In the Reference list, data citations must be labeled with "[DATASET]". A data reference must provide the database name, accession number/identifiers and a resolvable link to the landing page from which the data can be accessed at the end of the reference. Further instructions are available at <https://www.embopress.org/page/journal/14693178/authorguide#referencesformat>.

12) All Materials and Methods need to be described in the main text using our 'Structured Methods' format. According to this

format, the Methods section includes a Reagents and Tools Table (listing key reagents, experimental models, software and relevant equipment and including their sources and relevant identifiers) followed by a Methods and Protocols section describing the methods, ideally using a step-by-step protocol format. The aim is to facilitate adoption of the methodologies across labs. Please download and fill our Reagents and Tools Table template (.docx), which you can find in our author guidelines: https://www.embopress.org/page/journal/14693178/authorguide#structuredmethods.
When submitting your revised manuscript, please do not include the Reagents and Tools Table in the Methods section of the manuscript but upload it as a separate file choosing the file type "Reagent Table".
An example of a Method paper with Structured Methods can be found here: https://www.embopress.org/doi/10.15252/msb.20178071.

13) As part of the EMBO publication's Transparent Editorial Process, EMBO Reports publishes online a Review Process File to accompany accepted manuscripts. This File will be published in conjunction with your paper and will include the referee reports, your point-by-point response and all pertinent correspondence relating to the manuscript.

You are able to opt out of this by letting the editorial office know (emboreports@embo.org). If you do opt out, the Review Process File link will point to the following statement: "No Review Process File is available with this article, as the authors have chosen not to make the review process public in this case."

We would also welcome the submission of cover suggestions, or motifs to be used by our Graphics Illustrator in designing a cover.

I look forward to seeing a revised form of your manuscript when it is ready and please let me know if you have questions or comments regarding the revision.

Yours sincerely,

Martina Rembold, PhD
Senior Editor
EMBO reports

=================

Referee #1:

In this interesting manuscript, Zhang et al investigate the role of stn1 in the regulation Wnt/ß-catenin signaling in zebrafish ovary development. Like in mammals, Wnt signaling was known to play an important role during zebrafish female sex determination, as most wnt4 and wnt9b mutants develop as males. Both wnt4 and wnt9b are expressed in somatic follicle cells that surround the developing germ cells, but it was not known what cells respond to these signals. Using a transgenic Wnt reporter line, they show that the primary cell type in the ovary that appears to be Wnt responsive is germ cells, and in particular premeiotic germ cells and early-stage oocytes. Because wnt4/wnt9b are expressed in follicle cells that surround both Wnt responsive and Wnt non-responsive oocytes, they hypothesized that the Wnt responsive oocytes contain a factor that is permissive for activation of downstream responses to Wnt signal reception. They then asked if factors known to interact with ß-catenin were also enriched in early-stage germ cells, and identified Stn1, which encodes a subunit of the CST complex with known roles in telomere maintenance. Stn1 had been identified as a potential ß-catenin-associated protein by Ehyai et al, 2018, thought the evidence for this was weak. In addition, a zebrafish mutant for stn1 had been made as part of the Zebrafish All Genes KO Consortium for Chromosome 1 (ZAKOC) Chromosome 1 consortium (Sun et al., 2019). Using published scRNA-seq data for the zebrafish ovary they found that stn1 is primarily expressed in germ cells. Consistent with a function in germ cells development, stn1 mutants have normal germ cell numbers early, but have lost all germ cells by ~45 dpf. Consistent with a role in Wnt signaling, the germ cells present in late larval stn1 mutants have slightly reduced expression of the Wnt/ ß-catenin reporter transgene. To gain mechanistic insights into the role of stn1 in germ cell development, they compared the transcriptomes of wild-type and mutants at 19 and 25 dpf, and not surprisingly, found that mutants had reduced expression of genes that are normally expressed in germ cells. Using co-IP experiments they found evidence that Stn1 interacts with both ß-catenin and one of its downstream transcription factors, Tcf7L2. Evidence that Stn1 functions to enhance the output of ß-catenin signaling came from showing that it can enhance the transcriptional output of Tcf7L2 in a transcriptional reporter assay in HEK293 cells. They next identified putative Tcf/Lef binding sites in the gene regulatory regions of germ cell-expressed genes that were downregulated in the stn1 mutants and tested these in transcriptional enhancer assays using Tcf7L1 and also provide corroborating evidence using ChIP-qPCR that ß-catenin associates with these sequences in both ovaries and testes. Finally, they showed that overexpression of Wnt8a using a heat inducible transgene system could partially rescue germ cell development, and in particular oocytes, in stn1 mutants, consistent with stn1 mutants having a compromised response to endogenous Wnt signals.

Identifying novel regulators of Wnt/ß-catenin signaling would constitute a significant advance in the field, and the data presented in this manuscript makes a compelling argument that Stn functions in Wnt signal amplification. However, there are a few open questions and alternative explanations that need to be addressed before strong conclusions can be drawn. The first major concern is that Stn has known roles in telomere biology as a component of the CST complex that is required for telomere replication, capping, and protection. The authors argue that Stn has a germ cell-specific role, as loss of germ cells is the only major phenotype reported in the snt1 mutants. However, this reviewer could imagine that germ cells may be more sensitive to loss of the telomere maintenance functions than other cell types and thus telomere defects are the root cause of germ cell loss, not reduced Wnt signaling. The second major concern is that the authors argue that Stn1 function germ cell autonomous, but all evidence is correlative. In supplemental Figure 1E they show that stn expression is not restricted to the gonad, but is also expressed in other tissues. While they were able to partial rescue the germ cell defect by transgene overexpression of stn, this transgene expressed stn in all cells, not just germ cells. A more convincing experiment would be to attempt rescue by expressing stn in only germ cells using a germ cell-specific promoter (e.g. piwl1).

Minor criticisms/suggestions (in approximate order in text):

1. "In zebrafish, Wnt/β-catenin signaling plays important roles.." for completeness, Kossack et al., 2019 (PMID:30446521) and Liu et al., 2022 (PMID: 35588359) should also be referenced as these papers more directly demonstrate roles for Wnt signaling (wnt4 and wnt9b) in female development then the two papers that are currently referenced. (Also first add these to first sentence of Results). Also, the accepted nomenclature for wnt4a is now simply "wnt4(see updated nomenclature on ZFIN).

2. The experiments using 7X-tcf-Xla-siam:GFP to monitor wnt signaling has the significant caveat that GFP is very stable and may not represent active expression. It would be more accurate to look at gfp RNA by in situ hybridization.

3. Fig 1C: Using GFP intensity is not an accurate measure of expression. If the same amount of GFP is present in a small oocyte vs. a large oocyte, the signal will look stronger in the small because the volume is smaller and therefore [GFP] is higher.

4. FigS1a: it is not clear what this data represents. Is the Y-axis oocyte diameter? Is this data from RNA in situ? If so, a representative in situ should be shown.

5. "Since the intrinsic Wnt/β-catenin signaling activity sharply increased at this stage..." The phrase "..intrinsic Wnt/β-catenin signaling..." is misleading as it implies that the Wnt signal is intrinsic to the germ cells, yet the lead up to this was describing the role of wnt4, which is expressed in somatic cells (follicle cells). Perhaps the authors mean "germ cell-intrinsic response."

6. For completeness, Wnt4 is not the only Wnt expressed in the ovary. In addition to wnt4, wnt9b is also expressed in follicle cells and wnt8a in oocytes (Liu et al., 2022).

7. Figure 2C. In normal animals, the gonads of males and females at 25-30 dpf are significantly different in size, and therefore have different brightness of GFP (females larger than males; Krovel and Olsen, 2004; Dranow et al., 2016). The wildtype image shown in fig 2C looks to be female, but it would be expected that some gonads looked male-like. Were any WT male-looking gonads present? For completeness, sex ratios should be provided and an male example shown.

8. Fig 2D. Quantifying gonad size from images of gonads that have not been dissected is not an accurate method. Gonads should first be dissected (fixation of fish in PFA overnight aids in dissecting gonads; shrinkage may occur, but it will be proportional to all fish so relative size will still be accurate).

9. Pg6 : "The downregulated genes in the mutants at 19 hpf were not sorted..." should be "...19dpf...."

10. Fig 4b: Bulk RNA seq does not allow you to tell if the reduction of gene expression is due to reduced gene expression in germ cells, or due to mutants having many fewer germ cells relative to wildtype controls.

11. The RNA-seq results do not add anything novel to the story and should be provide as supplemental data. The authors only highlight germ cells expressed genes. Where any non-germ cell expressed genes also affected? Where any gene upregulated?

12. Fig. 4C Why were 4 hpf embryos chosen for IP analysis instead of using germ cells? Is stn a maternal transcript? Is there a role for stn at 4hpf? What is the relevance of its function at 4 hpf to its function in germ cells? Is Tcf7L2 expressed in germ cells? If so, at what stages?

13. What was the reasoning for only assaying for interactions between Stn1 and Tcf7L2 in Fig. 4C? Where Tcf7 and Tcf7L2 also assayed in pulldowns from fish lysates?

14. Why was Tcf7L1 used instead of Tcf7L2 in figure 4H? Does Stn also interact with Tcf7L1 in pull down assays? Are Tcf7L1a/b expressed in germ cells?

15. BIO assay: Fig4I, J It is not clear why the levels of protein not quantified in this assay? Instead, it is presented as only a qualitative result. Were these assays transient transfections of Stn expression constructs? Were these normalized for transfection efficiency?

16. "Taken together, these data indicate that Stn1 depletion leads to reduced oocyte-intrinsic activity of Wnt/β-catenin signaling, resulting in decreased expression of germ cell-specific genes and aberrant progression of oocytes." It should be noted that loss of stn1 also affects spermatogenesis as all mutant animals are sterile males.

Referee #2:

Summary:
In this paper by Zhou et al, the authors aim to better understand the role of Wnt/β-catenin and how they interact with Stn1 during zebrafish oogenesis. They start by measuring Wnt/β -catenin activity using a transgenic fish model and found that Wnt/ β-catenin signaling is most prominently expressed in oocytes sized 10 to 50um and is dynamic in expression. The authors follow up on this finding using RNA-seq data to identify the Wnt/ β-catenin amplifier Stn1. When Stn1 was ablated, the Stn1 null fish had no gonads and male phenotypes and were infertile. The loss of germ cells was not caused by Caspase3-mediated apoptosis and Stn1 was shown to downregulate germ cells specific genes. Upon further analysis, Stn1 is directly related to Wnt/ β-catenin activity through modulation of the β-catenin/Tcf complex. Lastly, these authors show that Wnt8a is partially sufficient at modulating oocyte loss caused by stn1 loss. These data show the importance of Wnt/ β-catenin in zebrafish oogenesis and support the importance of Stn1 in oogenesis.

Major:
1. Figure 1B,C: 1B) please use indicate oocytes of specific diameters to establish correlation of diameter with GFP brightness. 1C, please clarify that each dot represents a single oocyte. Please include how many animals and ovaries are represented at each time point?
2. Results, paragraph 2, supplementary Figure 1 text. Please rectify the discussion related to the timing of somatic cell Wnt4a, which was reported from a literature source (Kossack et al 2019) to be at 20-25 hourspf, to the data outlined in Supplementary Figure 1, which was focused at 20dayspf (S1A), 40dpf (S1B-D), and adult animals (S1E). It would be helpful to show the images used to calculate data in figure S1A.
3. Supplementary Figure S3: Please define the outcome of breeding images shown in the figure legend of Figure S3C. Figure S3G control appears that it may have the potential to overexpress stn1; was any phenotype noticed in the gonads of these mice?
4. Throughout the manuscript: Please define stn1+/? genotype; it appears that it represents a sibling control indicating possibly a heterozygote genotype (+/-).
5. Figure 2: the phenotype of the stn mutant zebrafish is very exciting. The results would be strengthened if there was more description to explain the discrepancy in results in mutant animals, many 50% or less representative sections. In addition, germ cell loss did not appear to be caused by a caspase 3-mediated apoptosis pathway, but no quantification is provided. It would also be helpful to investigate other death pathways or proliferation defects.
6. Figure 4: while it is a strength that both fish and in vitro data are used to generate these data, please clarify the rationale for the use of HEK293 cells, it is not clear what a "Wnt-off" cell line means.
7. Figures 5B, 6F, S4, please include a negative control to ensure the effect tested for is specific

Minor:
Throughout: please recheck all hpf and dpf notations, some to appear to be mislabeled (for example last line in the section describing Figure 3).

Referee #3:

The mechanisms of early germline differentiation are not fully understood in vertebrates. Previous studies in mammals and zebrafish have identified canonical Wnts, including Wnt8 and Wnt4 as a regulator of early germline development and germline stem cell renewal, respectively. In this interesting paper from Zhang and colleagues, the authors investigate the role of the Stn1 gene, a component of the CST complex that plays a role in telomere synthesis, replication, and response to replication stress. The authors report multiple loss of function mutant alleles disrupting zebrafish stn1. They provide compelling evidence for an essential Stn1 function development of gonads of both sexes, as all mutants are sterile males. They provide evidence that initial

germ cell numbers are intact; however, all germ cells are lost in juveniles. As anticipated based on loss of the germline, they report reduced expression of germ cell markers. The authors explored the upstream regulatory sequences in several germ cell genes and identified binding sites in silico and through CHIP and luciferase assays for TCF/Beta-Catenin and Stn1 in cultured HEK cells and/or from gonadal tissues. They hypothesized and concluded that Wnt signaling is amplified by Stn1 through a cooperative binding mechanism. Using a transgenic approach, they demonstrate that germ cell loss in stn1 mutant gonads can be suppressed by overexpressing Wnt8a. Based on expression of Wnt4 in the somatic gonad of zebrafish and its roles in mammals, they propose Wnt4 may be the relevant ligand. Overall, the study is well done, and the findings are novel; however, there are several points that should be clarified and some of the conclusions are not fully supported by the data provided. In addition, there are several grammatical errors throughout the manuscript. These are noted below in instances where the meaning or interpretation of the sentences are unclear.

Major:
1) Stn1 is not introduced in the introduction so the telomere discussion in the discussion sections comes out of context.
2) Although Wnt8a can suppress the mutant phenotype when overexpressed, Wnt4 but not Wnt8a expression in the ovary/testis is discussed. The rescue of germ cell loss by heat shock Wnt8a is convincing; however, what happens to the gonad at later stages is not discussed. Do the gonads develop further, are the rescued males/females fertile or do they arrest in oocyte development? If rescue is incomplete, is it possible that canonical Wnt is only required for the early stage and that noncanonical Wnt might be required later as Wnt4 has been shown to act in both the canonical and noncanonical Wnt pathways?
3) Expression of stn1 transcripts is shown from published single cell data. This data suggests that stn1 is expressed in germline stem cells and then is off and then is expressed again in early oocytes. Although PCR data are shown for stn1 expression, it is important to validate the single cell data with HCR or a similar in situ hybridization analysis to determine which cells/stages express stn1. This might be relevant to the point above, if Wnt8 rescue is incomplete.
4) The model suggests that Stn1 amplifies Wnt8, and the cartoon as depicted suggests that there is Wnt- independent Stn1 but also shows Wnt8 upstream of Stn1 abundance. This seems inconsistent with the rescue data, which seem to place Wnt downstream or in a parallel pathway to Stn1. It would help to clarify this in the model. Also, given that Stn1 expression also appears to be dynamic it would help to compare its expression to Wnt expression and to some of the targets indicated, and also to the Wnt receptors since they would likely be an intermediate between an activating Wnt and downstream signaling.
5) Related to stn1 expression, it is unclear how the cells/stages labeled by the Wnt reporter relate to the dynamic expression pattern of stn1.
6) "Conversely, in the stn1 mutants, the oocytes gradually diminished, ultimately leaving only fibrous tissue in the gonads." What was the evidence that the tissue is fibrous tissue as opposed to somatic gonad tissue?
7) In several places, the germ cell genes are described as downregulated. However, it is likely that the reduced expression is due to the reduced number and less advanced stages of germ cells in the mutant gonads. To distinguish between downregulation and less expression because there are fewer germ cells one would need to look at single cells, either with quantitative HCR (normalized to a "housekeeping" gene or single cell analysis.
8) "Wildtype larval zebrafish were treated from 19 to 25 dpf with XAV939 or PNU74654, which are Wnt/β-catenin signaling specific inhibitors that activate Axin or inhibit the β-catenin/TCF complex, respectively (Huang et al, 2009; Trosset et al, 2006). Subsequently, the transcript levels of ddx4, dnd1, piwil1, piwil2, tdrd9, tdrd1, and tdrd7a were evaluated. evaluated. All transcripts were decreased following XAV939 or PNU74654 treatment, as indicated by qRT-PCR analysis (Fig. S4A, B)." Did these treatments lead to loss of germ cells or phenocopy stn1 mutants? Were there any other phenotypes observed in treated fish? It is important to distinguish whether reduced expression was due to regulation, loss of germ cells, or other developmental delay phenotypes, and to determine the extent to which the phenotypes resemble stn1 mutants. Examination of transcripts by PCR is not sufficient to distinguish between these possibilities.
9) "Stn1 depletion weakened the association between β-catenin and the promoter regions of germ cell-specific genes at 25 dpf (Fig. 6B)." In a later sentence "strengthens" is used. The problem with these statements is that the stages of activation appear not to be achieved in the mutant and there are fewer germ cells, thus it is difficult to claim weaker association is directly due to loss of stn1 rather than due to loss of the germ cells or stage differences between mutant and control gonads. This should be qualified.
10) "which acts as a novel binding partner of Tcf/Lef and cooperates with the β-catenin/Tcf complex to amplify Wnt/β-catenin signaling in oocytes." The mutant data provided are not sufficient to support the statement that there is cooperative activity in oocytes.
11) In Figure 4A, it appears that the mutant germ cells do not reach the stage when robust activation of the reporter is activated. Failure to reach the stage makes it challenging to establish a regulatory relationship. Initial expression/reporter expression in early GCs of Ddx4 seems independent of stn1. Plus, as mentioned above, expression data indicates Stn1 is on in GSCs, then off then on in oocytes? How does reporter activation align with Stn1 expression? Stn1 expression should be validated.
12) Figure 4G, in the IP, all of the proteins also appear to come down with just GST.
13) For the luciferase assays, empty vector may not be the right control given how low the activity is for the germ cell genes compared to TOPFlash.
14) Fig. 6F, it seems challenging to make these comparisons since there are different numbers and stages of germ cells and there is high variation from gonad to gonad, even in the controls. Histological evaluation is likely to provide more meaningful comparisons as the stages can be directly compared.

Minor:
1) In Fig. 4H, there appears to be an extra plus sign in the line for Stn1-Flag.

2) In the abstract the following sentence needs context. It reads like this is already known as written. "The dynamically intrinsic activity. "However, the dynamically intrinsic activity of Wnt/β-catenin signaling in the progression of oocytes is poorly understood."." Maybe "the dynamics of Wnt signaling in oocyte progression has not been reported".
3) Stn1 should be spelled out and introduced the first time it is used.
4) In the abstract, "is enrichingly expressed in germ cells" should instead be "expression is enriched in..."
5) Also in the abstract, "the" is needed before transcription factor.
6) Abstract: "stn1 leads to loss of massive oocytes prior to or during their development to the IB stage," "should instead be leads to massive loss of oocytes."
7) Abstract: By "male-like phenotype" I assume the authors mean "phenotypically male" or "sterile male".
8) Abstract: The following sentence is unclear as written. "Temporally induced activation of the Wnt/β-catenin signaling partially reverses Stn1 depletion induced loss of germ cells and eventually promotes development of oocytes to the stage IB."
9) Abstract: I believe this is a typo and that "facilitating" should be "facilitating".
10) Introduction: The following sentence is unclear as written. "In ovaries, a key germ cell-intrinsic activity of signaling pathway, Wnt/β-catenin signaling that govern their development is evolutionarily conserved across the animal kingdom(Le Rolle et al, 2021; Takase & Nusse, 2016)."
11) Introduction: "is crucial for its pluripotent maintenance." "its" is not needed.
12) Introduction: "meiosis entry" is typically written as "meiotic entry"
13) Introduction: "regulated in progression". Consider instead "regulated during the progression".
14) The conclusion "These results imply that Stn1 may regulate Wnt/β-catenin signaling pathway and play key roles in germ cell development." Isn't really supported by the data presented immediately before it is made.
15) The following is a complex sentence. Consider splitting it into two sentences. Hence, an anti-active-caspase-3 antibody was employed to immunostain stn1 mutant embryos with a Tg(piwil1:EGFP-UTRnanos3) genetic background at 25 dpf. In agreement with phenomenon in vasa mutants (Hartung et al, 2014), the abundance of active-caspase-3-positive oocytes in mutants was not higher than in siblings despite oocyte sin stn1 mutants are apparent loss (Fig. 2F)."
16) In the section titled "Loss of Stn1 reduces the expression abandance of germ cell-specific genes" hpf is used several times to refer to the stage, but I think these are typos and should be dpf. - also, there is a typo in this title "abundance".
17) The following sentence is unclear as written. "(Fig. 3E). Taken together, these results suggest that loss of Stn1 leads to the germ cell numbers are greatly reduced in mutant gonads at 25 while not 19 hpf.
18) The first "the" is not needed in this sentence "The Stn1 bound with the Tcf7l2 fragment spanning residues 63-327 (Fig. 4F and G).
19) The basis for describing HEK cells as a "wnt off" line should be discussed briefly.
20) There is a typo in the following sentence. "Thus, Stn1 likely functions by binding with Tcf/Lef at target promoters and promoting the association of β-catenin/Tcf complexe with the target gene promoters.
21) "And its genetic deletion in zebrafish leads to loss of oocytes and results in a male-like phenotype and infertility." It appears to lead to loss of the stem cells too.
22) "its actin in oogenesis." Should "actin" be "action".
23) "In zebrafish, Ddx4 is required for germ cell differentiation and maintenance, while its disruption results in sterile adult male-like fish (Hartung et al., 2014). Similarly, Dnd1 is implicated in germ cell fate maintenance; its depletion in zebrafish leads to complete loss of germ cells (Chu et al, 2023; Weidinger et al, 2003)." The Ddx4 and Dnd1 phenotypes both lead to loss of the germline but are very different phenotypes from one another. In Dnd1 loss, the primordial germ cells are lost within the first few days of development and in ddx4 mutants they are lost much later in gonad development. Thus, they should not be described as "similar"
24) For the genotypes +/? is used throughout the figures. Instead, label these, which I assume to be either heterozygotes or homozygous WT, as "wild type", and assuming this is the case, in the figure state that no differences were observed between heterozygotes and homozygous WT, thus both genotypes are referred to as wild type.

Point-to-point response:

We thank the reviewers for their comments and suggestions. In response to these comments and suggestions, the following changes have been made. Also, a formatted pdf of the response was attached as supplementary information.

Referee #1:

In this interesting manuscript, Zhang et al investigate the role of stn1 in the regulation Wnt/ß-catenin signaling in zebrafish ovary development. Like in mammals, Wnt signaling was known to play an important role during zebrafish female sex determination, as most wnt4 and wnt9b mutants develop as males. Both wnt4 and wnt9b are expressed in somatic follicle cells that surround the developing germ cells, but it was not known what cells respond to these signals. Using a transgenic Wnt reporter line, they show that the primary cell type in the ovary that appears to be Wnt responsive is germ cells, and in particular premeiotic germ cells and early-stage oocytes. Because wnt4/wnt9b are expressed in follicle cells that surround both Wnt responsive and Wnt non-responsive oocytes, they hypothesized that the Wnt responsive oocytes contain a factor that is permissive for activation of downstream responses to Wnt signal reception. They then asked if factors known to interact with ß-catenin were also enriched in early-stage germ cells, and identified Stn1, which encodes a subunit of the CST complex with known roles in telomere maintenance. Stn1 had been identified as a potential ß-catenin-associated protein by Ehyai et al, 2018, thought the evidence for this was weak. In addition, a zebrafish mutant for stn1 had been made as part of the Zebrafish All Genes KO Consortium for Chromosome 1 (ZAKOC) Chromosome 1 consortium (Sun et al., 2019). Using published scRNA-seq data for the zebrafish ovary they found that stn1 is primarily expressed in germ cells. Consistent with a function in germ cells development, stn1 mutants have normal germ cell numbers early, but have lost all germ cells by ~45 dpf. Consistent with a role in Wnt signaling, the germ cells present in late larval stn1 mutants have slightly reduced expression of the Wnt/ ß-catenin reporter transgene. To gain mechanistic insights into the role of stn1 in germ cell development, they compared the transcriptomes of wild-type and mutants at 19 and 25 dpf, and not surprisingly, found that mutants had reduced expression of genes that are normally expressed in germ cells. Using co-IP experiments they found evidence that Stn1 interacts with both ß-catenin and one of its downstream transcription factors, Tcf7L2. Evidence that Stn1 functions to enhance the output of ß-catenin signaling came from showing that it can enhance the transcriptional output of Tcf7L2 in a transcriptional reporter assay in HEK293 cells. They next identified putative Tcf/Lef binding sites in the gene regulatory regions of germ cell-expressed genes that were downregulated in the stn1 mutants and tested these in transcriptional enhancer assays using Tcf7L1 and also provide corroborating evidence using ChIP-qPCR that ß-catenin associates with these sequences in both ovaries and testes. Finally, they showed that overexpression of Wnt8a using a heat inducible transgene system could partially rescue germ cell development, and in particular oocytes, in stn1 mutants, consistent with stn1 mutants having a compromised response to endogenous Wnt signals.

Response: We highly appreciate the positive feedback from this referee.

Identifying novel regulators of Wnt/ß-catenin signaling would constitute a significant advance in the field, and the data presented in this manuscript makes a compelling argument that Stn functions in Wnt signal amplification. However, there are a few open questions and alternative explanations that need to be addressed before strong conclusions can be drawn. The first major concern is that Stn has known roles in telomere biology as a component of the CST complex that is required for telomere replication, capping, and protection. The authors argue that Stn has a germ cell-specific role, as loss of germ cells is the only major phenotype reported in the snt1 mutants. However, this reviewer could imagine that germ cells may be more sensitive to loss of the telomere maintenance functions than other cell types and thus telomere defects are the root cause of germ cell loss, not reduced Wnt signaling. The second major concern is that the authors argue that Stn1 function germ cell autonomous, but all evidence is correlative. In supplemental Figure 1E they show that stn expression is not restricted to the gonad, but is also expressed in other tissues. While they were able to partial rescue the germ cell defect by transgene overexpression of stn, this transgene expressed stn in all cells, not just germ cells. A more convincing experiment would be to attempt rescue by expressing stn in only germ cells using a germ cell-specific promoter (e.g. piwl1).

Response: We sincerely appreciate your valuable comments and constructive suggestions. In response to the two major concerns raised, we performed additional experiments. The new results have been incorporated into the revised manuscript as Appendix Figure S5 and Appendix Figure S3I-K. Furthermore, we have added the corresponding results and discussion for these two sections to enhance the clarity and readability of the manuscript.

1) The role of telomere defects in germ cell loss after Stn1 depletion

Stn1 is a component of CST complex. CST depletion results in excessive telomerase activity and unleashed telomere length control (Chen et al, 2012, Nature 23; 488:540-4. doi: 10.1038/nature11269; Zaug et al., 2021, Nucleic Acids Res 49:11653-11665. DOI: 10.1093/nar/gkab942). As the reviewer pointed out, the role of telomere defects in germ cell loss after Stn1 depletion should be investigated. Adult telomerase-depleted zebrafish mutants (*tert*$^{-/-}$) exhibit both female and male phenotypes, and both sexes are capable of producing fertilized eggs and functional sperm (Anchelin et al., 2013 Dis Model Mech 6: 1101-1112 doi: 10.1242/dmm.011635; Henriques et al., 2013 PLoS Genet 9: e1003214 doi: 10.1371/journal.pgen.1003214). If excessive telomerase activity is a major contributor to germ cell loss after Stn1 depletion, inhibition of telomerase activity would be expected to restore germ cell development. To investigate this hypothesis, we treated *stn1* mutant zebrafish with specific telomerase inhibitors from 19 to 33 dpf to assess whether gonadal defects could be ameliorated. Small-molecule compounds BIBR1532 and RHPS4 are structurally distinct telomerase inhibitors. Both selectively inhibit telomerase activity and promote telomere shortening (Damm et al., 2001 EMBO J doi: 10.1093/emboj/20.24.6958; Chen et al, 2012, Nature 23; 488:540-4. doi: 10.1038/nature11269; Cookson et al., 2005, Mol. Pharmacol doi:10.1124/mol.105.013300; Gowan et al., Mol. Pharmacol doi:

10.1124/mol.60.5.981). Addition of BIBR1532 or RHPS4 did not ameliorate germ cell loss caused by Stn1 depletion. Therefore, telomerase dysfunction is unlikely to be a major contributor to germ cell loss after Stn1 depletion. This result has been included as Appendix Figure S5 in the revised manuscript and discussed in page 7-8, lines 294-309, and page 13, lines 539-556.

2) Stn1 functions in a germ cell-autonomous manner

We agree with your opinion that the current evidence regarding Stn1 function in a germ cell-autonomous manner remains correlative, and that this issue warrants further investigation. Following your suggestion, we generated a germ cell-specific transgenic line, *Tg(piwil1:stn1-Flag)*, expressing Stn1-Flag, for use in rescue analysis. Adult *stn1*$^{-/-}$*; Tg(piwil1:stn1-Flag)* zebrafish exhibit both female and male, and both are capable of producing fertilized eggs when crossed with wild-type males and females, respectively. Thus, we concluded that Stn1 functions in a germ cell-autonomous manner. This result has been included as Appendix Figure S3I-K in the revised manuscript and discussed in page 5-6, lines 216-224.

Minor criticisms/suggestions (in approximate order in text):

1. "In zebrafish, Wnt/β-catenin signaling plays important roles." for completeness, Kossack et al., 2019 (PMID:30446521) and Liu et al., 2022 (PMID: 35588359) should also be referenced as these papers more directly demonstrate roles for Wnt signaling (wnt4 and wnt9b) in female development then the two papers that are currently referenced. (Also first add these to first sentence of Results). Also, the accepted nomenclature for wnt4a is now simply "wnt4(see updated nomenclature on ZFIN).

Thank you for pointing out these issues. In accordance with your suggestion, the two papers have been cited in the revised manuscript as you indicated in both *Introduction* section and *Result* section. Additionally, *wnt4a* has been corrected to *wnt4* throughout the manuscript.

2. The experiments using 7X-tcf-Xla-siam:GFP to monitor wnt signaling has the significant caveat that GFP is very stable and may not represent active expression. It would be more accurate to look at gfp RNA by in situ hybridization.

Thanks for this comment and helpful suggestion. We agree with your opinion that the GFP in this transgenic line is highly stable and may not accurately reflect active gene expression. Following your suggestion, we performed fluorescence *in situ* hybridization to examine *gfp* mRNA expression levels in germ cells of different sizes, and quantified the fluorescence intensity of *gfp* mRNA. The results showed a similar pattern to total GFP fluorescence, with the size range shifting from 10- 45 μm to 10-35 μm when comparing GFP protein to *gfp* mRNA. The slight discrepancy in the oocyte size range between GFP mRNA and protein expression is likely attributable to the greater stability of the GFP protein. These new data have been included as Figure EV1 in the revised manuscript.

3. Fig 1C: Using GFP intensity is not an accurate measure of expression. If the same amount of GFP is present in a small oocyte vs. a large oocyte, the signal will look stronger in the small because the volume is smaller and therefore [GFP] is higher.

Thanks for this comment. We totally agree with your opinion that using GFP intensity as a measure of expression is not entirely accurate. To address the question, we re-quantified the total fluorescence intensity per cell in oocytes of specific diameters and established a correlation between oocyte diameter and total GFP fluorescence intensity. The new data has been included as Figure 1B, C in the revised manuscript. Both the new data and the fluorescence intensity of *gfp* mRNA support that Wnt/β-catenin signaling activation undergoes rapid up-and-down fluctuations in developing oocytes during the early primary growth stage.

4. FigS1a: it is not clear what this data represents. Is the Y-axis oocyte diameter? Is this data from RNA in situ? If so, a representative in situ should be shown.

Thank you for pointing out this issue. We regret that the data were not presented clearly. In addition, the citation was unintentionally omitted in the Figure legend section, as all authors were using the “Track Changes” feature in Microsoft Word during manuscript revision. The Y-axis represents the diameter of oocytes surrounded by *wnt4*$^{+}$ somatic cells. These raw data were extracted from confocal images of gonads at different developmental stages, which were stained for *wnt4* mRNA and are presented in Figure 2 of Kossack et al., 2019 (DOI: 10.1534/genetics.118.301620). We measured the oocyte diameter surrounded by *wnt4*$^{+}$ somatic cells and qualified the data. To present the data more clearly, Appendix Figure S1A has been revised, and the citation to Figure 2 of Kossack et al., 2019 has been added to the revised figure legend.

5. "Since the intrinsic Wnt/β-catenin signaling activity sharply increased at this stage..." The phrase "..intrinsic Wnt/β-catenin signaling..." is misleading as it implies that the Wnt signal is intrinsic to the germ cells, yet the lead up to this was describing the role of wnt4, which is expressed in somatic cells (follicle cells). Perhaps the authors mean "germ cell-intrinsic response."

Thank you for pointing out this issue. This sentence has been revised to state: “Since the germ cell-intrinsic response of Wnt/β-catenin signaling sharply increases at this stage”.

6. For completeness, Wnt4 is not the only Wnt expressed in the ovary. In addition to wnt4, wnt9b is also expressed in follicle cells and wnt8a in oocytes (Liu et al., 2022).

Thank you for pointing out this issue. The citation to Liu et al., 2022 has been added, and the information regarding Wnt9b and Wnt8a has been included in the revised *Results* and *Discussion* sections. Thank you again for your helpful suggestion.

7. Figure 2C. In normal animals, the gonads of males and females at 25-30 dpf are significantly different in size, and therefore have different brightness of GFP (females larger than males; Krovel and Olsen, 2004; Dranow et al., 2016). The wildtype image shown in fig 2C looks to be female, but it would be expected that some gonads looked male-like. Were any WT male-looking gonads present? For completeness, sex ratios should be provided and a male example shown.

Thank you for your helpful suggestion. Some wide-type gonads exhibited “male-like” morphology. Following your suggestion, an image of a representative male image and the corresponding sex ratios have been added to the revised Figure 2C to ensure completeness.

8. Fig 2D. Quantifying gonad size from images of gonads that have not been dissected is not an accurate method. Gonads should first be dissected (fixation of fish in PFA overnight aids in dissecting gonads; shrinkage may occur, but it will be proportional to all fish so relative size will still be accurate).

Thanks for this comment and helpful suggestion. Following your suggestion, we performed additional experiments to dissect PFA-fixed gonads and compare gonadal size between sibling and mutant fish at 19, 25, and 33 dpf. These new data have been incorporated into the revised manuscript as Figure EV2. These results are consistent with the observations from gonadal images. Hence, they further indicate that genetic loss of Stn1 leads to severely underdeveloped gonads starting at approximately 25 dpf.

9. Pg6 : "The downregulated genes in the mutants at 19 hpf were not sorted..." should be "...19dpf...."

Changed. Thank you.

10. Fig 4b: Bulk RNA seq does not allow you to tell if the reduction of gene expression is due to reduced gene expression in germ cells, or due to mutants having many fewer germ cells relative to wildtype controls.

Thank you for your comment. We totally agree with this opinion that it is difficult to determine whether the reduction in gene expression is attributable to decreased expression levels in germ cells or to mutants possessing significantly fewer germ cells. We have revised the sentence to read: “In contrast, 376 reduced transcripts were specifically identified in the mutants at 25 dpf (Appendix Fig. S4B, Table S1).”

11. The RNA-seq results do not add anything novel to the story and should be provide as supplemental data. The authors only highlight germ cells expressed genes. Where any non-germ cell expressed genes also affected? Where any gene upregulated?

Thank you for your comments and helpful suggestions. The bulk RNA-seq data derived from the trunk region (including the gonads) make it difficult to identify non-germ cell-specific

genes in the gonads that are affected, apart from the previously reported germ cell-specific genes. The increased transcripts have not been annotated to any "biological process" category, and no specific germ cell-expressed genes have been reported. Therefore, the reduced transcripts that are not germ cell-specific, along with the increased transcripts, were excluded from further analysis. Following this suggestion, the affected non-germ cell-specific expressed genes have been included in the revised Supplementary Table to ensure completeness of the dataset presentation. The RNA-seq results have been relocated to the supplementary data as Appendix Figure S4 to improve clarity of presentation.

12. Fig. 4C Why were 4 hpf embryos chosen for IP analysis instead of using germ cells? Is stn a maternal transcript? Is there a role for stn at 4hpf? What is the relevance of its function at 4 hpf to its function in germ cells? Is Tcf7L2 expressed in germ cells? If so, at what stages?

Thanks for this comment. We regret that our initial rationale for selecting 4 hpf embryos for IP analysis, rather than using germ cells, was not adequately explained. As mentioned in the manuscript, STN1 was identified as a potential β-catenin-associated protein from cultured cells through IP-MS analysis. However, the interaction between β-catenin and STN1 has not yet been validated by Co-IP or pull-down assays. Hence, we first investigated whether STN1 forms a complex with β-catenin and/or TCF. However, suitable antibodies against zebrafish Stn1 were not available. A *stn1*$^{-/-}$*;Tg (β-actin:stn1-Flag)* genetic background was utilized in combination with an anti-Flag antibody for the Co-IP assay. The *β-actin* promoter is ubiquitously active throughout development and into adulthood. To minimize the sacrifice of adult female fish while ensuring sufficient cell yield for Co-IP analysis, we used embryos from a *stn1*$^{-/-}$*;Tg (β-actin:stn1-Flag)* line to examine the interaction between Flag-Stn1 and endogenous β-catenin and/or Tcf. A recent study by He et al. (2020, PLoS Biol 18: e3000561) demonstrated that β-catenin interacts with Tcf7 or Tcf7l2 to induce expression of maternal β-catenin target genes at 4 hpf. Given that Flag-Stn1 is ubiquitously expressed in the *stn1*$^{-/-}$*;Tg(β-actin:stn1-Flag)* line, embryos at 4 hpf were selected for Co-IP analysis to investigate whether Flag-Stn1 forms a complex with endogenous β-catenin and/or Tcf7l2. To ensure a clearer description, we revised the corresponding section in the *Results* (page 8, lines 319-335).

The *stn1* mRNA is indeed maternally deposited, as shown in the Figure 1-12, which was generated from previously published RNA-seq data (White et al., 2017, Elife https://doi.org/10.7554/elife.30860 PMID: 29144233; https://www.ebi.ac.uk/gxa/experiments/E-ERAD-475/). Since the *stn1* zygotic mutant fish exhibits male-like phenotype associated with infertility, we could not obtain maternal mutant embryos at 4 hpf to eliminate maternally derived mRNA or protein for phenotypic analysis. Therefore, investigating the function of maternally deposited Stn1 and comparing its functional significance at 4 hpf with that in germ cells remains challenging. In future studies, we plan to employ translation-blocking morpholinos to perform knockdown (KD) analysis for elucidating the additional role of maternal Stn1 in embryos at 4 hpf.

He et al. (2020) has examined the expression of *tcf7l2* during oogenesis. *tcf7l2* exhibited strong expression in developing oocytes (Figure S9, PLoS Biol 18: e3000561). Additionally, the scRNA-seq dataset generated by Liu et al. (2022) revealed that *tcf7l2* mRNA is expressed

in subclusters from germline stem cells to early oocytes (Figure 1-13). Given that *stn1* and *tcf7l2* are expressed during the same developmental stage, their interaction is likely to occur.

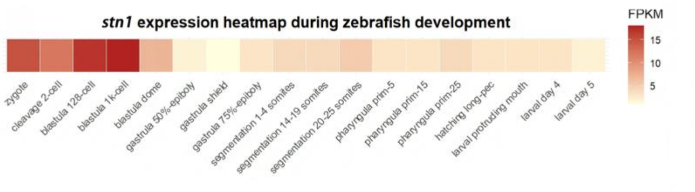

Figure 1-12 The expression of *stn1* during embryogenesis.

13. What was the reasoning for only assaying for interactions between Stn1 and Tcf7L2 in Fig. 4C? Where Tcf7 and Tcf7L2 also assayed in pulldowns from fish lysates?

Thank you for your questions. He et al. (2020, PLoS Biol 18: e3000561) examined the expression of *tcf7*, *tcf7l2*, *tcf7l1a*, and *tcf7l1b* during early developmental stages. Their results showed that *tcf7* and *tcf7l2* exhibited strong maternal expression in pre-ZGA embryos, whereas *tcf7l1a* and *tcf7l1b* showed very weak expression at these stages. Due to an antibody against zebrafish Tcf7l2 was already validated by knockout analysis, we therefore detected the interaction between Stn1 and Tcf7l2 in the Co-IP assay. However, due to the absence of antibodies specific to zebrafish Tcf7 and the failure to obtain suitable commercial antibodies against zebrafish Tcf7l1, we were unable to detect these two Tcf family members in the fish lysates. To address these questions raised by the reviewer, we alternatively performed additional GST pull-down assays using HEK293T cells expressing all tagged members of the Tcf/Lef family. The GST pull-down assays showed that Stn1 binds to all tagged members of the Tcf/Lef family. These results have been included as Figure 3D in the revised manuscript. Additionally, given that *stn1* and each member of the *tcf/lef* family are co-expressed in germ cells based on the scRNA-Seq dataset (Figure 1-13), it is likely that Stn1 interacts with members of the Tcf/Lef family in these cells.

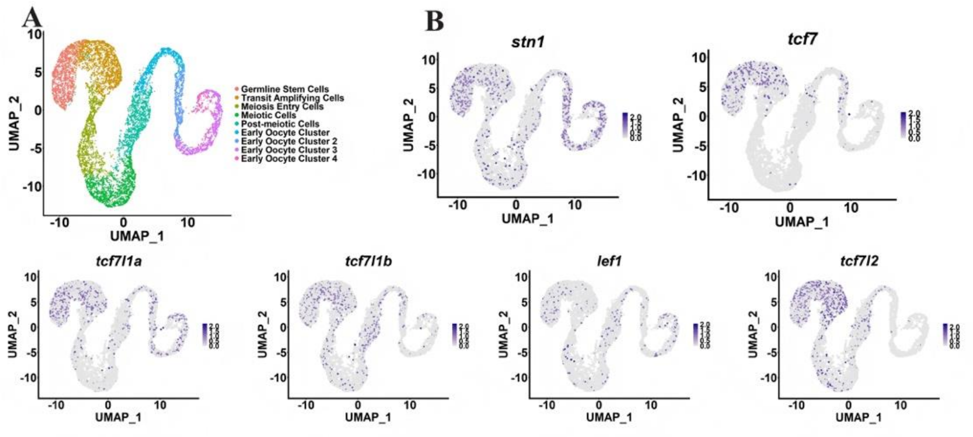

Figure 1-13 Gene expression UMAP plots of the *tcf/lef* gene family. (A) UMAP plot of germ cell subclusters, with cells colored according to computationally inferred subtypes. (B) UMAP plots showing gene expression patterns for the indicated genes.

14. Why was Tcf7L1 used instead of Tcf7L2 in figure 4H? Does Stn also interact with Tcf7L1 in pull down assays? Are Tcf7L1a/b expressed in germ cells?

In the current version of the manuscript, only Tcf7l2 is included in Figure 4D to maintain consistency with Figure 4C. To investigate whether Stn1 interacts with Tcf/Lef family members, we performed GST pull-down experiments using all members of the Tcf/Lef family. The results showed that Stn1 binds to all Tcf/Lef family members. From these questions raised by the reviewer, we realized the complete dataset should be incorporated into the Figure to make the manuscript easily to read. Hence, the complete dataset has been incorporated into the revised manuscript as Figure 3D. Given that Stn1 interacts with all Tcf/Lef family members and that the constitutively active VP16-Tcf7l1ΔN construct was exactly in our hands, we used this construct together with the Flag-Stn1 construct to perform co-transfection experiments. Indeed, both *tcf7l1a* and *tcf7l1b* mRNAs were detected in germ cells from single-cell RNA-Seq data of ovaries at 40 dpf (Figure 1-13) (Liu et al., 2022, eLife 11: e76014) as well as by *in situ* hybridization on cryosections of adult ovaries (Fig. S9 in He et al., 2020, PLoS Biology 18: e3000561).

15. BIO assay: Fig4I, J It is not clear why the levels of protein not quantified in this assay? Instead, it is presented as only a qualitative result. Were these assays transient transfections of Stn expression constructs? Were these normalized for transfection efficiency?

Thank you for your comments and helpful suggestions. All experiments were performed in at least three independent replicates, and a representative result is presented in Figure 4I, J. These assays were performed using transient transfections of Flag-Stn1 expression constructs co-transfected with GFP expression constructs. The expression of GFP was used as a control to monitor transfection efficiency. Following this suggestion, we quantified the relative Stn1 protein levels normalized to GFP, and the new data have been included in the revised Figure 4I.

16. "Taken together, these data indicate that Stn1 depletion leads to reduced oocyte-intrinsic activity of Wnt/β-catenin signaling, resulting in decreased expression of germ cell-specific genes and aberrant progression of oocytes." It should be noted that loss of stn1 also affects spermatogenesis as all mutant animals are sterile males.

Thank you for your valuable comments. We have revised this sentence to state: "Taken together, these data indicate that Stn1 depletion leads to reduced germ cell-intrinsic Wnt/β-catenin signaling activity, resulting in decreased expression of germ cell-specific genes and aberrant progression of germ cell development".

Referee #2:

Summary:

In this paper by Zhou et al, the authors aim to better understand the role of Wnt/β-catenin and how they interact with Stn1 during zebrafish oogenesis. They start by measuring Wnt/β -catenin activity using a transgenic fish model and found that Wnt/ β-catenin signaling is most prominently expressed in oocytes sized 10 to 50um and is dynamic in expression. The authors follow up on this finding using RNA-seq data to identify the Wnt/ β-catenin amplifier Stn1. When Stn1 was ablated, the Stn1 null fish had no gonads and male phenotypes and were infertile. The loss of germ cells was not caused by Caspase3-mediated apoptosis and Stn1 was shown to downregulate germ cells specific genes. Upon further analysis, Stn1 is directly related to Wnt/ β-catenin activity through modulation of the β-catenin/Tcf complex. Lastly, these authors show that Wnt8a is partially sufficient at modulating oocyte loss caused by stn1 loss. These data show the importance of Wnt/ β-catenin in zebrafish oogenesis and support the importance of Stn1 in oogenesis.

Major:

1. Figure 1B, C: 1B) please use indicate oocytes of specific diameters to establish correlation of diameter with GFP brightness. 1C, please clarify that each dot represents a single oocyte. Please include how many animals and ovaries are represented at each time point?

Thank you for your valuable suggestions. Following your suggestion, we re-analyzed the data and calculated the correlation between diameters of oocyte and total GFP fluorescence intensity per oocyte. The updated data have been incorporated into revised Figure 1C. Additionally, the statement "each data point represents an individual oocyte" has been added to the figure legend for panel 1C. The number of zebrafish analyzed at each time point is indicated in the figure legend.

1. Results, paragraph 2, supplementary Figure 1 text. Please rectify the discussion related to the timing of somatic cell Wnt4a, which was reported from a literature source (Kossack et al 2019) to be at 20-25 hourspf, to the data outlined in Supplementary Figure 1, which was focused at 20dayspf (S1A), 40dpf (S1B-D), and adult animals (S1E). It would be helpful to show the images used to calculate data in figure S1A.

Thank you for your valuable suggestion. Following this suggestion, we revised the *Result* section pertaining to Supplementary Figure 1 in paragraph 2 as follows: “We noted that *wnt4*, a Wnt ligand, is specifically expressed in the somatic cells surrounding oocytes measuring 20 to 50 μm in zebrafish gonads at 20 to 25 dpf (Appendix Fig. S1A) (Kossack et al., 2019),” to highlight the oocytes surrounded by *wnt4*$^{+}$ somatic cells within this size range. The data presented in Appendix Fig. S1A were re-analyzed from confocal images shown in Figure 2E–G of Kossack et al., 2019. Since these images have already been published in the journal Genetics, we have included a citation to "Figure 2E–G, Kossack et al., 2019" in the legend for Appendix Fig. S1A to ensure clarity and proper attribution.

2. Supplementary Figure S3: Please define the outcome of breeding images shown in the figure legend of Figure S3C. Figure S3G control appears that it may have the potential to overexpress stn1; was any phenotype noticed in the gonads of these mice?

Thank you for this valuable suggestion. We determined the fertilization rate based on whether embryos reached the shield stage by 6 hpf. The “Representative images of embryos at 6 hpf from different mating combination are shown” has been included in the figure legend for S3C to ensure clarity in the description. For the control in Appendix Figure S3G with Stn1 overexpression, no morphological phenotypic differences in the gonads were observed between Stn1-overexpressing WT and control WT.

3. Throughout the manuscript: Please define stn1+/? genotype; it appears that it represents a sibling control indicating possibly a heterozygote genotype (+/-).

Thank you for pointing out this issue. We regret that we did not present this clearly. In the revised manuscript, the *stn1*$^{+/?}$ genotype was designated as the WT sibling to ensure clarity in the description.

4. Figure 2: the phenotype of the stn mutant zebrafish is very exciting. The results would be strengthened if there was more description to explain the discrepancy in results in mutant animals, many 50% or less representative sections. In addition, germ cell loss did not appear to be caused by a caspase 3-mediated apoptosis pathway, but no quantification is provided. It would also be helpful to investigate other death pathways or proliferation defects.

Thanks for your comments and helpful suggestions. Indeed, the proportion of representative sections corresponds to that observed in WT siblings, rather than in mutants. Representative sections with 50% or less were in the sibling group to show the sex ratios.

Following this suggestion, we quantified the cleaved caspase-3 immunostaining data presented in Figure 2F, and the quantitative analysis has been included as Figure 2G in the revised manuscript. The results show that the number of active caspase-3-positive oocytes in the mutants was not significantly increased compared to sibling controls. Therefore, germ cell loss in *stn1* mutants does not primarily occur via caspase-mediated apoptosis.

Germ cell loss may involve non-caspase-mediated cell death pathways or defects in proliferation. It appears unlikely that proliferative defects occur in *stn1* mutants, as germ cells are gradually lost over time from the H.E. sections. Our new results from germ cell-specific rescue experiment by *Tg(piwil1:stn1-Flag)* showed that Stn1 functions in a germ cell-autonomous manner. Stn1 depletion leads to a reduction in the expression of various germ cell-specific genes, including *ddx4*, *piwil1*, and *piwil2*. Mutants of *ddx4* and *piwil2* exhibit germ cell loss through non-caspase-mediated mechanisms (Hartung et al., 2014, Molecular Reproduction and Development, 81: 946–961, doi: 10.1002/mrd.22414 ; Houwing et al., 2008, The EMBO Journal DOI: 10.1038/emboj.2008.204). In contrast, *piwil1* mutants exhibit germ cell loss through caspase-mediated mechanisms (Houwing et al., 2007, Cell, doi: 10.1016/j.cell.2007.03.026). However, the mechanisms underlying germ cell loss through

non-caspase-mediated pathways remain poorly understood. Given the complexity of the process, a comprehensive understanding of the molecular mechanisms driving germ cell loss in *stn1* mutants within a short timeframe is challenging. As the reviewer pointed out, this represents an important issue that warrants further investigation. Additional studies are therefore required to fully elucidate these mechanisms. This issue has been discussed in the revised manuscript page 13, line 530-538.

5. Figure 4: while it is a strength that both fish and in vitro data are used to generate these data, please clarify the rationale for the use of HEK293 cells, it is not clear what a "Wnt-off" cell line means.

Thank you for your suggestion. We regret that we did not clarify the rationale for using HEK293T cells or provide sufficient information regarding the "Wnt-off" cell line HEK293T. HEK293T cells exhibit a functional Wnt signaling system with basal Wnt/β-catenin activity and are commonly regarded as a Wnt-off cell line. This cell line is highly amenable to induction of Wnt/β-catenin signaling activity through modulation of Wnt activators at multiple levels. Therefore, HEK293T cells were transfected with a low dose of VP16-Tcf7l1ΔN) along with different dose of Stn1 to examine whether Stn1 facilitated VP16-Tcf7l1ΔN-induced Wnt activity in a dose-dependent manner. This paragraph has been revised in the new version according to your suggestion.

7. Figures 5B, 6F, S4, please include a negative control to ensure the effect tested for is specific

Thank you for your helpful suggestion. For Figure 5B, we repeated the experiments using the FopFlash plasmid (a mutant version of TopFlash containing mutated TCF/LEF binding sites upstream of a luciferase reporter, serving as a control for Wnt reporter activity) as a negative control, and the promoter of the Wnt target gene *lef1*-driven luciferase expression plasmid as a positive control. The new result has been included as revised Figure 4B to ensure that the effect being tested is specific. For Figures 6F and S4, the original results have been removed and replaced with fluorescence *in situ* hybridization data from ovarian cryosections as revised Figure EV4 and Appendix Figure S6, 7. These updated data help avoid potential misinterpretation due to the reduced number of germ cells in mutants compared to WT siblings.

Minor:

Throughout: please recheck all hpf and dpf notations, some to appear to be mislabeled (for example last line in the section describing Figure 3).

We carefully checked the entire manuscript and changed all mislabeled hpf to dpf. Thank you.

Referee #3:

The mechanisms of early germline differentiation are not fully understood in vertebrates.

Previous studies in mammals and zebrafish have identified canonical Wnts, including Wnt8 and Wnt4 as a regulator of early germline development and germline stem cell renewal, respectively. In this interesting paper from Zhang and colleagues, the authors investigate the role of the Stn1 gene, a component of the CST complex that plays a role in telomere synthesis, replication, and response to replication stress. The authors report multiple loss of function mutant alleles disrupting zebrafish stn1. They provide compelling evidence for an essential Stn1 function development of gonads of both sexes, as all mutants are sterile males. They provide evidence that initial germ cell numbers are intact; however, all germ cells are lost in juveniles. As anticipated based on loss of the germline, they report reduced expression of germ cell markers. The authors explored the upstream regulatory sequences in several germ cell genes and identified binding sites in silico and through CHIP and luciferase assays for TCF/Beta-Catenin and Stn1 in cultured HEK cells and/or from gonadal tissues. They hypothesized and concluded that Wnt signaling is amplified by Stn1 through a cooperative binding mechanism. Using a transgenic approach, they demonstrate that germ cell loss in stn1 mutant gonads can be suppressed by overexpressing Wnt8a. Based on expression of Wnt4 in the somatic gonad of zebrafish and its roles in mammals, they propose Wnt4 may be the relevant ligand. Overall, the study is well done, and the findings are novel; however, there are several points that should be clarified and some of the conclusions are not fully supported by the data provided. In addition, there are several grammatical errors throughout the manuscript. These are noted below in instances where the meaning or interpretation of the sentences are unclear.

Major:

1) Stn1 is not introduced in the introduction so the telomere discussion in the discussion sections comes out of context.

Thank you for your helpful suggestion. Following this suggestion, a paragraph introducing Stn1 has been added to the *Introduction* section to improve the logical coherence of the manuscript.

2) Although Wnt8a can suppress the mutant phenotype when overexpressed, Wnt4 but not Wnt8a expression in the ovary/testis is discussed. The rescue of germ cell loss by heat shock Wnt8a is convincing; however, what happens to the gonad at later stages is not discussed. Do the gonads develop further, are the rescued males/females fertile or do they arrest in oocyte development? If rescue is incomplete, is it possible that canonical Wnt is only required for the early stage and that noncanonical Wnt might be required later as Wnt4 has been shown to act in both the canonical and noncanonical Wnt pathways?

Thank you for your comments. In addition to *wnt4, wnt9b* was expressed in follicle cells, and *wnt8a* was expressed in oocytes at this stage. This information has been added and discussed in the revised manuscript in accordance with the suggestion of Reviewer 1. The *stn1* mutants with *Tg(hsp70l:wnt8a-GFP)* transgenic background were heated shock daily from 19 to 45 dpf, and oocyte development was restored by heat shock-induced Wnt8a expression to stage IB with diameters ranging from 20 to 60 μm; however, further development did not proceed.

These results suggest that activation of Wnt/β-catenin signaling maintains oocyte development in Stn1-deficient individuals up to 20-60 μm (Figure 5D, E). This result is consistent with the observation that Wnt activity sharply increases in developing oocytes ranging in size from 10 to 45 μm and then rapidly declines (Figure 1B, C; Figure EV1). We also performed additional experiments. The *stn1* mutants with *Tg(hsp70l:wnt8a-GFP)* transgenic background were subjected to daily heat shock from 19 to 90 dpf; however, neither adult females nor males were obtained, and oocyte development failed to progress. When heat-shocked adult *stn1*$^{-/-}$*;Tg(hsp70l:wnt8a-GFP)* male-like fish crossed with wild-type females, these fish induced the females to lay unfertilized eggs. Therefore, the restoration is incomplete. There are several possible explanations for this observation. First, as mentioned by the reviewer, Wnt4 and Wnt9b have been shown to function in both canonical and noncanonical Wnt signaling pathways. Canonical Wnt signaling may be required only at early stages, whereas noncanonical Wnt signaling might be necessary at later stages. Second, Stn1 is a component of the CST complex, which plays an essential role in the suppression of telomerase activity and telomere length control. The function of the CST complex may be important during later stages of development. Further studies are required to elucidate these underlying mechanisms. This issue has been discussed in the revised manuscript, page 13, line 539-556. Thank you again for your valuable suggestion.

3) Expression of stn1 transcripts is shown from published single cell data. This data suggests that stn1 is expressed in germline stem cells and then is off and then is expressed again in early oocytes. Although PCR data are shown for stn1 expression, it is important to validate the single cell data with HCR or a similar in situ hybridization analysis to determine which cells/stages express stn1. This might be relevant to the point above, if Wnt8 rescue is incomplete.

Thank you for your valuable suggestion. The *stn1*$^{-/-}$*; Tg(hsp70l:wnt8a-GFP)* were heated shock daily from 19 to 45 dpf, and oocyte size was restored to the range of 20–60 μm without further developmental progression (Figure 5D, E). Additionally, we generated a transgenic line with the germ cell-specific *piwil1* promoter driving Stn1-Flag expression, designated *Tg(piwil1:stn1-Flag)*, and performed rescue analysis. *Tg(piwil1:stn1-Flag)* was able to rescue the germ cell deficiency in *stn1* mutants, restoring to both adult female and male associated with fertility (Appendix Figure S3I-K). Given distinct expression patterns between *piwil1* and *stn1* during germ cell development, as shown in Figure 3-4a, and considering that Stn1 protein stability is regulated by Wnt/β-catenin signaling activity at the post-translational level according to our biochemical analysis, it is likely that post-translational regulation of the germ cell-specifically expressed Stn1 plays a more critical role in its function during germ cell development than its mRNA level. Additionally, as the reviewer suggested in Question 2, alternative explanations may exist for the incomplete rescue observed with Wnt8a. Following this suggestion, we also performed fluorescence *in situ* hybridization on ovarian cryosections to stain *stn1* mRNA for validating the single-cell data. The new data has been included as Appendix Figure S1E in the revised manuscript. We observed that *stn1* mRNA expression is enriched at different developmental stages, including oogonia and oocytes at stages IA and IB, which are characterized by Wnt activation-driven GFP protein or mRNA expression as shown

in the revised Figure 1B and EV1. These results validated the single-cell data and further confirms the high expression of *stn1* across various developmental stages of female germ cells.

4) The model suggests that Stn1 amplifies Wnt8, and the cartoon as depicted suggests that there is Wnt- independent Stn1 but also shows Wnt8 upstream of Stn1 abundance. This seems inconsistent with the rescue data, which seem to place Wnt downstream or in a parallel pathway to Stn1. It would help to clarify this in the model. Also, given that Stn1 expression also appears to be dynamic it would help to compare its expression to Wnt expression and to some of the targets indicated, and also to the Wnt receptors since they would likely be an intermediate between an activating Wnt and downstream signaling.

Thank you for your comments and valuable suggestions. We regret that we did not present the last paragraph of the *Results* section clearly and provide a schematic illustration sufficient to summarize our findings. Our results showed that Wnt/β-catenin signaling activity sharply rises in the oocytes with size from 10 to 45 μm and rapidly declines later. Additionally, Wnt activity promotes the stability of Stn1, which in turn binds to Tcf and facilitates the association of the β-catenin/Tcf transcriptional complex with the promoter regions of germ cell-specific Wnt target genes, thereby amplifying Wnt activity. Depletion of Stn1 fails to amplify Wnt signaling in zebrafish germ cells, resulting in reduced Wnt activity and impaired oocyte development, with size of most oocytes failing to reach 20 μm. Therefore, forced activation of Wnt signaling in *stn1*$^{-/-}$ mutant fish with the *Tg(hsp70l:wnt8a-GFP)* transgenic background, achieved through daily heat shock from 19 to 45 dpf, was used to determine whether it could promote oocyte development in these mutants. Indeed, forced activation of Wnt signaling in *stn1*$^{-/-}$ mutant fish promotes progression of oocyte development to a size within the 20–60 μm range, which corresponds to the stage characterized by Wnt activation. These results suggest that Stn1-mediated amplification of Wnt signaling is essential for oocyte development to attain a size within the 20–60 μm range. Indeed, the transgenic line was used to induce ectopic expression of Wnt8a in order to enhance Wnt signaling in *stn1*$^{-/-}$ mutant fish. In our opinion, this approach should not be considered a phenotypic rescue but rather an experimental manipulation involving forced gene expression. Therefore, the last paragraph of the *Result* section has been revised for clarity. The final paragraph of the *Discussion* section has been revised to enhance the readability of the accompanying schematic. Additionally, the cartoon has been modified to enhance its clarity and facilitate understanding (Figure 5F).

Following this suggestion, we used UMAP analysis of the developmental trajectory of female germ cells from a single-cell RNA sequencing dataset (in which germ cells with a diameter less than 40 μm) to compare *stn1* expression with the Wnt germ cell-specific targets that we identified (Liu et al., 2022). As shown in Figures 3–4a, *stn1* expression is abundant in subpopulations of germline stem cells and early oocytes, and relatively lower in subpopulations located at intermediate stages. The expression of the Wnt germ cell-specific targets that we identified spans across the developmental stages of germ cells, albeit with varying abundance. The overlapping expression subpopulations between these Wnt targets and *stn1* suggest that the expression of the germ cell-specific targets are likely regulated by

Stn1. The *stn1* mRNA levels may not accurately reflect its protein abundance in germ cells, as our biochemical analysis showed that Stn1 stability is regulated by Wnt/β-catenin signaling activity at the post-translational level. Additionally, as mentioned in the response to Question 3, the rescue results observed in the *piwil1* promoter-driven Stn1-Flag transgenic background *Tg(piwil1:stn1-Flag)* suggest that post-translational regulation of the germ cell-specifically expressed Stn1 likely plays a more critical role in its function during germ cell development. Furthermore, we analyzed the expression subpopulations of multiple Frizzled (Fzd) receptors. As shown in Figures 3–4b, the expression subpopulations of *fzd* overlap with that of *stn1*, although the number of germ cells expressing certain Fzd members is low. Thus, the overlapping expression subpopulations of *stn1*, *fzd*, and Wnt germ cell-specific targets do exist. Taken together, these results suggest that Stn1 likely influences Wnt activity and the expression of germ cell-specific genes.

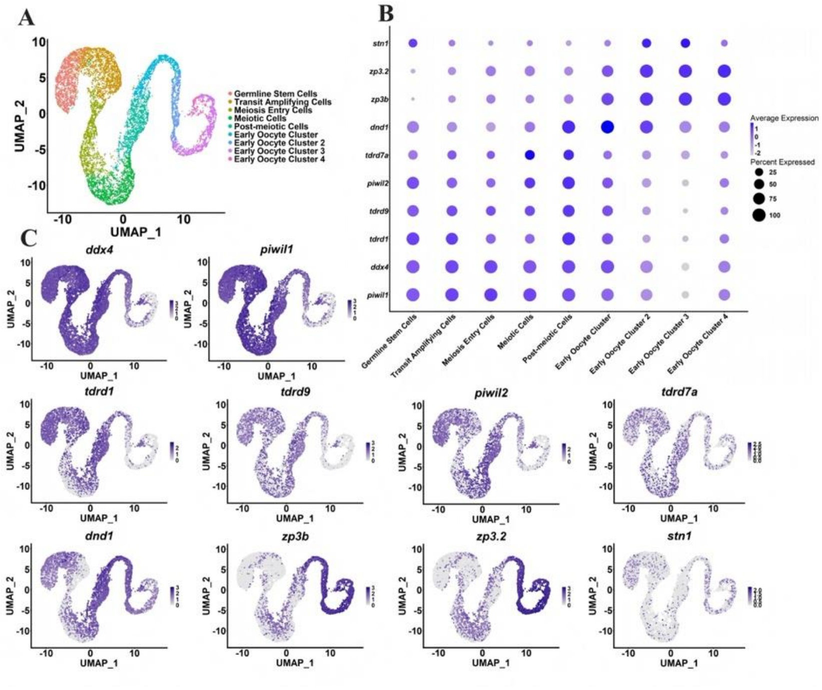

Figure 3-4a Gene expression UMAP plots of the Wnt germ cell-specific targets gene family. (A) UMAP plot of germ cell subclusters, with cells colored according to computationally inferred subtypes. (B) UMAP plots showing gene expression patterns for the indicated genes. (C) UMAP plots showing gene expression patterns for the indicated genes.

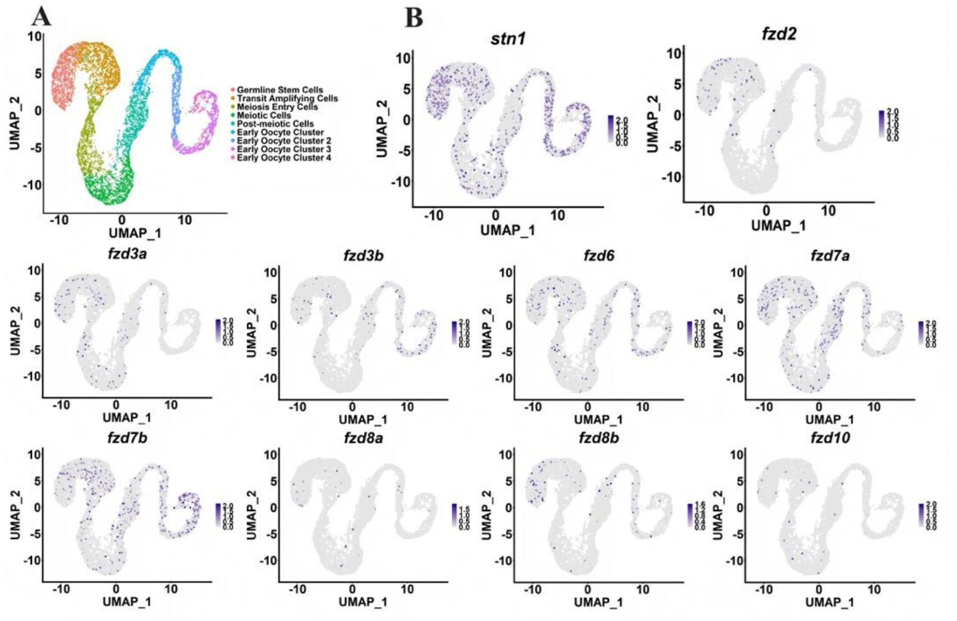

Figure 3-4b Gene expression UMAP plots of the *frizzled* gene family. (A) UMAP plot of germ cell subclusters, with cells colored according to computationally inferred subtypes. (B) UMAP plots showing gene expression patterns for the indicated Frizzed receptors.

5) Related to stn1 expression, it is unclear how the cells/stages labeled by the Wnt reporter relate to the dynamic expression pattern of stn1.

Thank you for your comments. To address this question, we performed additional fluorescence *in situ* hybridization experiments on ovarian cryosections to investigate the expression of *stn1* and Wnt-activated *gfp* in ovaries across different developmental stages. The new data has been included in the revised Appendix Figure S1E and Figure EV1, respectively. The expression pattern of *stn1* closely resembles that of *gfp* in germ cells of varying sizes. These results suggest that Stn1 likely functions in Wnt-activated germ cells. The mRNA level of *stn1* may not accurately reflect its protein abundance in germ cells, as our biochemical analysis showed that Stn1 stability is regulated by Wnt/β-catenin signaling activity at the post-translational level (Figure 3I).

6) "Conversely, in the stn1 mutants, the oocytes gradually diminished, ultimately leaving only fibrous tissue in the gonads." What was the evidence that the tissue is fibrous tissue as opposed to somatic gonad tissue?

Thank you for your comments. There is no evidence to support the identification of this tissue as fibrous tissue. Therefore, this sentence has been revised as follows: “Conversely, in the *stn1* mutants, the oocytes gradually diminished from the gonads.”

7) In several places, the germ cell genes are described as downregulated. However, it is

likely that the reduced expression is due to the reduced number and less advanced stages of germ cells in the mutant gonads. To distinguish between downregulation and less expression because there are fewer germ cells one would need to look at single cells, either with quantitative HCR (normalized to a "housekeeping" gene or single cell analysis.

Thank you for your comments and valuable suggestions. We totally agree with the opinion of the reviewer that describing germ cell genes as downregulated is inappropriate, given the reduced number of germ cells in the mutants. Following this suggestion, we performed additional fluorescence *in situ* hybridization on ovarian cryosections to examine the mRNA levels of each gene for comparison between siblings and mutants. The qRT-PCR results have been removed, and new data have been included as Appendix Figure S4F in the revised manuscript. Indeed, the expression of germ cell-specific genes is relatively lower in the residual germ cells of the mutants. Thank you again for your valuable suggestion.

8) "Wildtype larval zebrafish were treated from 19 to 25 dpf with XAV939 or PNU74654, which are Wnt/β-catenin signaling specific inhibitors that activate Axin or inhibit the β-catenin/TCF complex, respectively (Huang et al, 2009; Trosset et al, 2006). Subsequently, the transcript levels of ddx4, dnd1, piwil1, piwil2, tdrd9, tdrd1, and tdrd7a were evaluated. evaluated. All transcripts were decreased following XAV939 or PNU74654 treatment, as indicated by qRT-PCR analysis (Fig. S4A, B)." Did these treatments lead to loss of germ cells or phenocopy stn1 mutants? Were there any other phenotypes observed in treated fish? It is important to distinguish whether reduced expression was due to regulation, loss of germ cells, or other developmental delay phenotypes, and to determine the extent to which the phenotypes resemble stn1 mutants. Examination of transcripts by PCR is not sufficient to distinguish between these possibilities.

Thank you for your comments. We totally agree with the reviewer's opinion that transcript examination by qRT-PCR is insufficient, and we should exclude the possibility you mentioned above. To address these questions, we performed additional experiments. After treatment with XAV939 or PNU74654 from 19 to 33 dpf, juvenile zebrafish were collected and subjected to body length measurement. Their ovaries were dissected for cryosection preparation to assess germ cell loss and to perform fluorescence *in situ* hybridization, which was used to examine the mRNA levels of each gene and quantify expression levels in oocytes of different sizes for comparison between the inhibitor-treated and DMSO control groups. Treatment with XAV939 or PNU74654 did not reduce the body length of zebrafish. In addition, morphological difference was not observed between the inhibitor-treated and DMSO control groups. Furthermore, slight or minimal germ cell loss was observed following exposure to either inhibitor. This observation may be attributed to the suboptimal concentration of the inhibitors administered, which was maintained at a lower level to minimize potential toxicity in juvenile zebrafish. Given that the *gfp* mRNA level in the *Tg(7×TCF-Xla.Siam:GFP)* transgenic line sharply increases in oocytes with diameters ranging from 10 to 35 μm and then rapidly declines, we performed FISH to assess the

transcript levels of Wnt-induced *gfp* and the germ cell-specific target genes in oocytes measuring 10 to 30 µm in diameter. The mRNA levels of the examined genes were significantly reduced. Thus, the reduction in mRNA levels of the examined genes was not attributable to developmental delay, a finding that resembles the phenotype observed in *stn1* mutants. The new data have been included as Appendix Figure S6 in the revised manuscript. This issue has been discussed in the revised manuscript, page 9-10, lines 385-415. Thank you again for your valuable suggestion.

9) "Stn1 depletion weakened the association between β-catenin and the promoter regions of germ cell-specific genes at 25 dpf (Fig. 6B)." In a later sentence "strengthens" is used. The problem with these statements is that the stages of activation appear not to be achieved in the mutant and there are fewer germ cells, thus it is difficult to claim weaker association is directly due to loss of stn1 rather than due to loss of the germ cells or stage differences between mutant and control gonads. This should be qualified.

Thank you for your comments. We totally agree with the reviewer's opinion that "it is difficult to claim a weaker association is directly due to loss of Stn1 rather than due to loss of the germ cells or stage differences between mutant and control gonads". To address this question, we performed new ChIP experiments. The ovaries of sibling zebrafish were dissected, and germ cells were dissociated from the gonad using a modified protocol based on Blokhina et al., 2019 (PLOS Genetics 15:e1007730). Given that the oocyte diameter of most germ cells in the mutants was less than 20 µm, the dissociated cells were filtered through a sieve to isolate germ cells with a diameter less than 20 µm. To ensure the reliability of the results, equal numbers of cells from siblings and mutants were then subjected to ChIP-qPCR analysis. These results indicate that Stn1 depletion resulted in a reduced association between β-catenin and the promoter regions of germ cell-specific genes at 25 dpf. The new data were included as revised Figure 5B in the revised manuscript.

10) "which acts as a novel binding partner of Tcf/Lef and cooperates with the β-catenin/Tcf complex to amplify Wnt/β-catenin signaling in oocytes." The mutant data provided are not sufficient to support the statement that there is cooperative activity in oocytes.

Thank you for pointing out this issue. We have revised the sentence as follows: "which functions as a novel binding partner of Tcf/Lef and facilitates the activity of β-catenin/Tcf complex, thereby amplifying Wnt/β-catenin signaling in oocytes" in the revised manuscript.

11) In Figure 4A, it appears that the mutant germ cells do not reach the stage when robust activation of the reporter is activated. Failure to reach the stage makes it challenging to establish a regulatory relationship. Initial expression/reporter expression in early GCs of Ddx4 seems independent of stn1. Plus, as mentioned above, expression data indicates Stn1 is on in GSCs, then off then on in oocytes? How does reporter activation align with Stn1 expression? Stn1 expression should be validated.

Thank you for your comments and helpful suggestions. We totally agree with the reviewer's

opinion that establishing a regulatory relationship between Stn1 and Wnt activity is challenging. As the reviewer noted, mutant germ cells do not reach the developmental stage at which robust activation of the reporter occurs. Our results showed that Stn1 associates with and facilitates the activity of the β-catenin/Tcf complex, thereby amplifying Wnt/β-catenin signaling. Thus, Stn1 does not function as a master regulator of these germ cell-specific genes, including *ddx4*, but rather as an amplifier to enhance Wnt/β-catenin signaling levels. Hence, the observation that initial expression or reporter expression in early germ cells of Ddx4 is independent of stn1 is understandable. To address the question raised by the reviewer, we performed additional fluorescence *in situ* hybridization experiments on ovarian cryosections to examine the expression of *stn1* across different developmental stages. The new data has been included as Appendix Figure S1E. The expression of *stn1* in germ cells of varying sizes suggests that Stn1 may play a functional role in germ cells exhibiting Wnt activation. The mRNA level of *stn1* does not accurately reflect its protein abundance in germ cells, as our biochemical analysis showed that Stn1 stability is regulated by Wnt/β-catenin signaling activity at the post-translational level.

12) Figure 4G, in the IP, all of the proteins also appear to come down with just GST.

Thank you for your comments. We acknowledge that the washing steps for the glutathione sepharose beads following incubation with cell lysate containing potentially interacting proteins were insufficient, resulting in incomplete removal of unbound proteins. Therefore, we repeated the GST pull-down assays with increased washing steps to ensure complete removal of unbound proteins. The new result has been included as Figure 3G in the manuscript. This result demonstrated that Stn1 binds to the Tcf7l2 fragment spanning residues 63–327.

13) For the luciferase assays, empty vector may not be the right control given how low the activity is for the germ cell genes compared to TOPFlash.

Thank you for your comments. We totally agree with the reviewer's opinion. To address this question, we performed new experiments using the FopFlash plasmid (a mutant version of TopFlash containing mutated TCF/LEF binding sites upstream of a luciferase reporter, serving as a control for Wnt reporter activity) as a negative control, and the promoter of the Wnt target gene *lef1* driven luciferase expression plasmid as a positive control. The relative Wnt reporter activity driven by the indicated gene promoters is presented as fold change relative to the FopFlash control. The new result has been included as the revised figure 4B. The expression of these reporters was induced when co-transfected with VP16-Tcf7l1ΔN into HEK293T cells. Hence, the promoter regions of these germ cell-specific genes contain Wnt/β-catenin-responsive elements.

14) Fig. 6F, it seems challenging to make these comparisons since there are different numbers and stages of germ cells and there is high variation from gonad to gonad, even in the controls. Histological evaluation is likely to provide more meaningful comparisons as the stages can be directly compared.

Thank you for your comments. We totally agree with the reviewer's opinion. To address this question, we performed additional experiments. Following heat shock treatment from 19 to 33 dpf, juvenile zebrafish were collected and subjected to body length measurement and genotyping analysis. Their ovaries were dissected for cryosection preparation to perform fluorescence *in situ* hybridization, which was used to examine the mRNA levels of the target genes and quantify expression levels in oocytes of different sizes for comparison between mutant groups with and without heat shock. The qRT-PCR results have been moved and the new data have been included as Figure EV4 in the revised manuscript. These results showed that temporal induction of Wnt/β-catenin signaling activity alleviates the reduced mRNA levels of germ cell-specific genes caused by Stn1 loss.

Minor:
1) In Fig. 4H, there appears to be an extra plus sign in the line for Stn1-Flag.

The symbol "++" was used to indicate a higher dose compared to "+".

2) In the abstract the following sentence needs context. It reads like this is already known as written. "The dynamically intrinsic activity. "However, the dynamically intrinsic activity of Wnt/β-catenin signaling in the progression of oocytes is poorly understood." Maybe "the dynamics of Wnt signaling in oocyte progression has not been reported".

Changed.

3) Stn1 should be spelled out and introduced the first time it is used.

Added.

4) In the abstract, "is enrichingly expressed in germ cells" should instead be "expression is enriched in..."

Changed.

5) Also in the abstract, "the" is needed before transcription factor.

Added.

6) Abstract: "stn1 leads to loss of massive oocytes prior to or during their development to the IB stage," "should instead be leads to massive loss of oocytes."

Changed.

7) Abstract: By "male-like phenotype" I assume the authors mean "phenotypically male" or "sterile male".

Thank you for pointing out this issue. This sentence has been revised to “male-like phenotype associated with infertility”.

8) Abstract: The following sentence is unclear as written. "Temporally induced activation of the Wnt/β-catenin signaling partially reverses Stn1 depletion induced loss of germ cells and eventually promotes development of oocytes to the stage IB."

Thank you for pointing out this issue. This sentence has been changed to “The loss of germ cells caused by Stn1 depletion can be partially restored by temporal activation of the Wnt/β-catenin signaling pathway, which facilitates oocyte development to stage IB”.

9) Abstract: I believe this is a typo and that "facilitating" should be "facilitating".

Changed.

10) Introduction: The following sentence is unclear as written. "In ovaries, a key germ cell-intrinsic activity of signaling pathway, Wnt/β-catenin signaling that govern their development is evolutionarily conserved across the animal kingdom (Le Rolle et al, 2021; Takase & Nusse, 2016)."

Thank you for pointing out this issue. This sentence has been changed to “In ovaries, the germ cell-intrinsic activity of the Wnt/β-catenin signaling pathway, which regulates their development, is evolutionarily conserved across the animal kingdom (Le Rolle *et al*, 2021; Takase & Nusse, 2016)”.

11) Introduction: "is crucial for its pluripotent maintenance." "its" is not needed.

Deleted.

12) Introduction: "meiosis entry" is typically written as "meiotic entry"

Changed.

13) Introduction: "regulated in progression". Consider instead "regulated during the progression".

Changed.

14) The conclusion "These results imply that Stn1 may regulate Wnt/β-catenin signaling pathway and play key roles in germ cell development." Isn't really supported by the data presented immediately before it is made.

Thank you for pointing out this issue. This sentence has been changed to “Collectively, these

results suggest that Stn1 may play a role in germ cell development".

15) The following is a complex sentence. Consider splitting it into two sentences. Hence, an anti-active-caspase-3 antibody was employed to immunostain stn1 mutant embryos with a Tg(piwil1:EGFP-UTRnanos3) genetic background at 25 dpf. In agreement with phenomenon in vasa mutants (Hartung et al, 2014), the abundance of active-caspase-3-positive oocytes in mutants was not higher than in siblings despite oocyte sin stn1 mutants are apparent loss (Fig. 2F)."

Thank you for pointing out this issue. This sentence has been changed in the revised manuscript.

16) In the section titled "Loss of Stn1 reduces the expression abandance of germ cell-specific genes" hpf is used several times to refer to the stage, but I think these are typos and should be dpf. - also, there is a typo in this title "abundance".

Changed.

17) The following sentence is unclear as written. "(Fig. 3E). Taken together, these results suggest that loss of Stn1 leads to the germ cell numbers are greatly reduced in mutant gonads at 25 while not 19 hpf.

Thank you for pointing out this issue. This sentence has been changed to "Taken together, these results suggest that loss of Stn1 leads to a significant reduction in germ cell numbers in mutant gonads starting from 25 dpf".

18) The first "the" is not needed in this sentence "The Stn1 bound with the Tcf7l2 fragment spanning residues 63-327 (Fig. 4F and G).

Deleted.

19) The basis for describing HEK cells as a "wnt off" line should be discussed briefly.

Thank you for pointing out this issue. The following sentence has been added: "Human HEK293T cells exhibit a functional Wnt signaling system with basal Wnt/β-catenin activity and are commonly regarded as a Wnt-off cell line. This cell line is highly amenable to induction of Wnt/β-catenin signaling activity through modulation of Wnt activators at multiple levels.".

20) There is a typo in the following sentence. "Thus, Stn1 likely functions by binding with Tcf/Lef at target promoters and promoting the association of β-catenin/Tcf complexe with the target gene promoters.

Changed.

21) "And its genetic deletion in zebrafish leads to loss of oocytes and results in a male-like phenotype and infertility." It appears to lead to loss of the stem cells too.

Thank you for pointing out this issue. The sentence has been revised as “And its genetic deletion in zebrafish leads to germ cell loss and results in a male-like phenotype associated with infertility.”

22) "its actin in oogenesis." Should "actin" be "action".

Changed. Thank you.

23) "In zebrafish, Ddx4 is required for germ cell differentiation and maintenance, while its disruption results in sterile adult male-like fish (Hartung et al., 2014). Similarly, Dnd1 is implicated in germ cell fate maintenance; its depletion in zebrafish leads to complete loss of germ cells (Chu et al, 2023; Weidinger et al, 2003)." The Ddx4 and Dnd1 phenotypes both lead to loss of the germline but are very different phenotypes from one another. In Dnd1 loss, the primordial germ cells are lost within the first few days of development and in ddx4 mutants they are lost much later in gonad development. Thus, they should not be described as "similar"

Thank you for pointing out this issue. The term "similar" has been removed to improve clarity and precision in the description.

24) For the genotypes +/? is used throughout the figures. Instead, label these, which I assume to be either heterozygotes or homozygous WT, as "wild type", and assuming this is the case, in the figure state that no differences were observed between heterozygotes and homozygous WT, thus both genotypes are referred to as wild type.

We appreciate the reviewer’s helpful comment. Your assumption is correct, and in response, we have replaced “+/?” with “wild type” in all figures and text. Because wild type and heterozygous individuals exhibited no significant differences, both are described as “wild type.” Thank you.

1st Revision - Editorial Decision 12th Mar 2026

Dear Dr. Zhou

Thank you for the submission of your revised manuscript to EMBO reports. Please accept my sincere apologies regarding the delay in handling your manuscript. Unfortunately, referee #3, despite having raised a number of concerns, was not responsive and available to review your revised manuscript, but I acknowledge that you have performed extensive additional experimentation to address all concerns. Referee #1 and #2 have evaluated your revised manuscript and both are very positive about your study and recommend publication.

Before I can accept the manuscript, I need you to address some minor points below:

- Your manuscript will be published in our Reports section. In order to comply with this format, I kindly ask you to combine the Results and Discussion section.

- Regarding the Author Contributions, we now use CRediT to specify the contributions of each author in the journal submission system. CRediT replaces the author contribution section, which therefore needs to be removed from the manuscript text. You can use the free text box in our system if you wish to provide more detailed descriptions. See also guide to authors https://www.embopress.org/page/journal/14693178/authorguide#authorshipguidelines.

- References: please use et al after 10 author names.

- Author checklist: please select responses from the pull-down menu, in column D.

- In the Author checklist lines 81 and 82 you refer to statements about blinding or randomization in the methods section, which I could not locate. Please make sure these are present.

- Please add the fund ZR2025MS429 in the online manuscript handling system. The information in the system and in the text must be congruent. In the manuscript text all funding information needs to go under Acknowledgments; a separate section (heading) should not be used.

- Please add a callout for Fig. EV1

- Appendix: please remove the legend for Appendix Table S1 as the table is not part of this file but a separate dataset.
- The Appendix Table S1 file should be renamed to Dataset EV1. The Excel file needs to be upldd without the zip folder; the legend should be provided in another tab of the file; the correct nomenclature should be Dataset EV1 in all places (source file name, legend in the Excel file, title in the system, callouts in the manuscript.)
- Appendix Figure legends: please specify whether 'n' refers to technical or biological/independent replicates.
- Appendix Figure S3H, K: please define the statistical test used.

- Materials and methods should be Methods

- Please add information related to n in the legends of figures 3H, I.
- Please make sure that it is clear in all legends what 'n' refers to, in the case of cell culture please specify whether it is technical or independent replicates and please make sure to apply statistical analysis only to independent experiments.

- Data availability section: this section should only refer to the deposited data. Please remove the statement on data sharing on request.

- The link to the Single Cell Portal in line 615 seems broken. I received an error message when clicking on it. Could you please check?

- You reanalysed the scRNA-seq dataset from Liue et al, 2022. You can add a data citation that refers to the dataset in addition to citing the paper it was published in.
In the text this would look like (Liu et al., 2022, data ref: Liu et al., 2022).
The data ref in the reference list should refer to the dataset using its URL instead of journal name etc. and have the tag [DATASET] at the end of the reference.

- Why are the numbers for WT male and WT female the exact same in Appendix Fig. S3K and the associated source data .xls file (fertilization rate and number of embryos)?

- For Figure 5B, source data file, it appears that the number showin in Column A, line 8 is the same as that in line 9. The same applies to column B, column E and column F lines 8 and 9. It appears that the numbers have been duplicated (line 8 = line 9). Please check and confirm whether this is intended or a mistake.

- As a standard procedure, we modify abstract and title to make them more accessible to our general readership. Please find my suggestions copied below my signature and please try to shorten the abstract further to 175 words.

We look forward to seeing a final version of your manuscript as soon as possible.

With kind regards,

Martina Rembold, PhD
Senior Editor
EMBO reports

=================

Referee #1:

The authors addressed all of my previous critiques and suggestions. The new data provide much stronger support for the conclusions they draw.

Referee #2:

The authors should be commended for the thoughtful approach to addressing reviewer concerns. They performed additional experiments that added significant value to their manuscript in rigor and clarity.

==============

Stn1 promotes zebrafish oocyte development via amplifying Wnt/β-catenin signaling

Wnt/β-catenin signaling regulates oocyte development in vertebrates. However, the dynamics of Wnt signaling in oocyte progression has not been reported. Here, we analyse oocytes across different developmental stages in zebrafish and find that the activity of Wnt/β-catenin signaling sharply increases when oocytes reach a size of 10 to 45 μm (stage IA to middle stage IB) and subsequently declines rapidly. In addition, we show that expression of the CST complex subunit Stn1 is enriched in germ cells. Stn1interacts with the transcription factor Tcf/Lef, facilitates its association with promoters of various germ cell-specific genes, thereby enhancing Wnt/β-catenin signaling activity in oocytes. Genetic deletion of stn1 leads to massive loss of oocytes prior to or during their development to stage IB, resulting in a male-like phenotype associated with infertility. The loss of germ cells caused by Stn1 depletion can be partially restored by temporal activation of the Wnt/β-catenin signaling pathway, which facilitates oocyte development to stage IB. Our findings highlight the importance of Wnt/β-catenin signaling in promoting the expression of germ cell-specific genes and provide novel insights into the physiological functions of Stn1 in maintaining oocyte development in zebrafish.

The authors have addressed all minor editorial requests.

Dr. Jianfeng Zhou
Ocean University of China
School of Medicine and Pharmacy
5 Yushan Road
Qingdao 266003
China

Dear Dr. Zhou,

I am very pleased to accept your manuscript for publication in the next available issue of EMBO reports. Thank you for your contribution to our journal.

Your manuscript will be processed for publication by EMBO Press. It will be copy edited and you will receive page proofs prior to publication. Please note that you will be contacted by Springer Nature Author Services to complete licensing and payment information.

You may qualify for financial assistance for your publication charges - either via a Springer Nature fully open access agreement or an EMBO initiative. Check your eligibility: https://link.springer.com/journal/44319/how-to-publish-with-us

Should you be planning a Press Release on your article, please get in contact with embo_production@springernature.com as early as possible in order to coordinate publication and release dates.

If you have any questions, please do not hesitate to contact the Editorial Office. Thank you for your contribution to EMBO Reports.

Yours sincerely,

Martina Rembold, PhD
Senior Editor
EMBO reports

>>> Please note that it is EMBO Reports policy for the transcript of the editorial process (containing referee reports and your response letter) to be published as an online supplement to each paper. If you do NOT want this, you will need to inform the Editorial Office via email immediately. More information is available here: https://link.springer.com/partners/embo-press/editorial-policies#Peer%20review

EMBO Press Author Checklist

Corresponding Author Name: Jianfeng Zhou	
Journal Submitted to: EMBO Reports	
Manuscript Number: EMBOR-2025-61689V1	

USEFUL LINKS FOR COMPLETING THIS FORM
The EMBO Journal - Author Guidelines
EMBO Reports - Author Guidelines
Molecular Systems Biology - Author Guidelines
EMBO Molecular Medicine - Author Guidelines

Reporting Checklist for Life Science Articles (updated January
This checklist is adapted from Materials Design Analysis Reporting (MDAR) Checklist for Authors. MDAR establishes a minimum set of requirements in transparent reporting in the life sciences (see Statement of Task: 10.31222/osf.io/9sm4x). Please follow the journal's guidelines in preparing your
Please note that a copy of this checklist will be published alongside your article.

Abridged guidelines for figures
1. Data
The data shown in figures should satisfy the following conditions:
- the data were obtained and processed according to the field's best practice and are presented to reflect the results of the experiments in an accurate and unbiased manner.
- ideally, figure panels should include only measurements that are directly comparable to each other and obtained with the same assay.
- plots include clearly labeled error bars for independent experiments and sample sizes. Unless justified, error bars should not be shown for technical
- if n<5, the individual data points from each experiment should be plotted. Any statistical test employed should be justified.
- Source Data should be included to report the data underlying figures according to the guidelines set out in the authorship guidelines on Data

2. Captions
Each figure caption should contain the following information, for each panel where they are relevant:
- a specification of the experimental system investigated (eg cell line, species name).
- the assay(s) and method(s) used to carry out the reported observations and measurements.
- an explicit mention of the biological and chemical entity(ies) that are being measured.
- an explicit mention of the biological and chemical entity(ies) that are altered/varied/perturbed in a controlled manner.
- the exact sample size (n) for each experimental group/condition, given as a number, not a range;
- a description of the sample collection allowing the reader to understand whether the samples represent technical or biological replicates (including how many animals, litters, cultures, etc.).
- a statement of how many times the experiment shown was independently replicated in the laboratory.
- definitions of statistical methods and measures:
  - common tests, such as t-test (please specify whether paired vs. unpaired), simple χ2 tests, Wilcoxon and Mann-Whitney tests, can be unambiguously identified by name only, but more complex techniques should be described in the methods section;
  - are tests one-sided or two-sided?
  - are there adjustments for multiple comparisons?
  - exact statistical test results, e.g., P values = x but not P values < x;
  - definition of 'center values' as median or average;
  - definition of error bars as s.d. or s.e.m.

Please complete ALL of the questions below.
Select "Not Applicable" only when the requested information is not relevant for your study.

Materials

Newly Created Materials	Information included in the manuscript?	In which section is the information available? (Reagents and Tools Table, Materials and Methods, Figures, Data Availability Section)
New materials and reagents need to be available; do any restrictions apply?	Not Applicable	Not Applicable

Antibodies	Information included in the manuscript?	In which section is the information available? (Reagents and Tools Table, Materials and Methods, Figures, Data Availability Section)
For **antibodies** provide the following information:<br>- Commercial antibodies: RRID (if possible) or supplier name, catalogue number and or/clone number<br>- Non-commercial: RRID or citation	Yes	Reagents and Tools Table

DNA and RNA sequences	Information included in the manuscript?	In which section is the information available? (Reagents and Tools Table, Materials and Methods, Figures, Data Availability Section)
**Short novel DNA or RNA including primers, probes**: provide the sequences.	Yes	Reagents and Tools Table

Cell materials	Information included in the manuscript?	In which section is the information available? (Reagents and Tools Table, Materials and Methods, Figures, Data Availability Section)
**Cell lines:** Provide species information, strain. Provide accession number in repository **OR** supplier name, catalog number, clone number, and/**OR** RRID.	Yes	Reagents and Tools Table
**Primary cultures:** Provide species, strain, sex of origin, genetic modification status.	Not Applicable	Not Applicable
Report if the cell lines were recently **authenticated** (e.g., by STR profiling) and tested for mycoplasma contamination.	Yes	Materials and Methods

Experimental animals	Information included in the manuscript?	In which section is the information available? (Reagents and Tools Table, Materials and Methods, Figures, Data Availability Section)
**Laboratory animals or Model organisms:** Provide species, strain, sex, age, genetic modification status. Provide accession number in repository **OR** supplier name, catalog number, clone number, **OR** RRID.	Yes	Reagents and Tools Table
**Animal observed in or captured from the field:** Provide species, sex, and age where possible.	Not Applicable	Not Applicable
Please detail **housing and husbandry conditions**.	Yes	Materials and Methods

Plants and microbes	Information included in the manuscript?	In which section is the information available? (Reagents and Tools Table, Materials and Methods, Figures, Data Availability Section)
**Plants:** provide species and strain, ecotype and cultivar where relevant, unique accession number if available, and source (including location for collected wild specimens).	Not Applicable	Not Applicable
**Microbes:** provide species and strain, unique accession number if available, and source.	Yes	Materials and Methods

Human research participants	Information included in the manuscript?	In which section is the information available? (Reagents and Tools Table, Materials and Methods, Figures, Data Availability Section)
If collected and within the bounds of privacy constraints report on age, sex and gender or ethnicity for all study participants.	Not Applicable	Not Applicable

Core facilities	Information included in the manuscript?	In which section is the information available? (Reagents and Tools Table, Materials and Methods, Figures, Data Availability Section)
If your work benefited from core facilities, was their service mentioned in the acknowledgments section?	Not Applicable	Not Applicable

Design

Study protocol	Information included in the manuscript?	In which section is the information available? (Reagents and Tools Table, Materials and Methods, Figures, Data Availability Section)
If study protocol has been **pre-registered, provide DOI in the manuscript**. For clinical trials, provide the trial registration number **OR** cite DOI.	Not Applicable	Not Applicable
Report the **clinical trial registration number** (at ClinicalTrials.gov or equivalent), where applicable.	Not Applicable	Not Applicable

Laboratory protocol	Information included in the manuscript?	In which section is the information available? (Reagents and Tools Table, Materials and Methods, Figures, Data Availability Section)
Provide DOI OR other citation details if **external detailed step-by-step protocols** are available.	Yes	Materials and Methods

Experimental study design and statistics	Information included in the manuscript?	In which section is the information available? (Reagents and Tools Table, Materials and Methods, Figures, Data Availability Section)
Include a statement about **sample size** estimate even if no statistical methods were used.	Not Applicable	Not Applicable
Were any steps taken to minimize the effects of subjective bias when allocating animals/samples to treatment (e.g. **randomization procedure**)? If yes, have they been described?	Yes	Materials and Methods
Include a statement about **blinding** even if no blinding was done.	Yes	Materials and Methods
Describe **inclusion/exclusion criteria** if samples or animals were excluded from the analysis. Were the criteria pre-established?<br><br>If sample or data points were omitted from analysis, report if this was due to attrition or intentional exclusion and provide justification.	Yes	Materials and Methods
For every figure, are **statistical tests** justified as appropriate? Do the data meet the assumptions of the tests (e.g., normal distribution)? Describe any methods used to assess it. Is there an estimate of variation within each group of data? Is the variance similar between the groups that are being statistically compared?	Yes	Materials and Methods

Sample definition and in-laboratory replication	Information included in the manuscript?	In which section is the information available? (Reagents and Tools Table, Materials and Methods, Figures, Data Availability Section)
In the figure legends: state number of times the experiment was **replicated** in laboratory.	Yes	Figure Legends
In the figure legends: define whether data describe **technical or biological replicates**.	Yes	Figure Legends

Ethics

Ethics	Information included in the manuscript?	In which section is the information available? (Reagents and Tools Table, Materials and Methods, Figures, Data Availability Section)
Studies involving **human participants**: State details of **authority granting ethics approval** (IRB or equivalent committee(s), provide reference number for approval.	Not Applicable	Not Applicable
Studies involving **human participants**: Include a statement confirming that **informed consent** was obtained from all subjects and that the experiments conformed to the principles set out in the WMA Declaration of Helsinki and the Department of Health and Human Services Belmont Report.	Not Applicable	Not Applicable
Studies involving **human participants:** For publication of **patient photos**, include a statement confirming that consent to publish was obtained.	Not Applicable	Not Applicable
Studies involving experimental **animals**: State details of **authority granting ethics approval** (IRB or equivalent committee(s), provide reference number for approval. Include a statement of compliance with ethical regulations.	Yes	Materials and Methods
Studies involving **specimen and field samples:** State if relevant **permits** obtained, provide details of authority approving study; if none were required, explain why.	Not Applicable	Not Applicable

Dual Use Research of Concern (DURC)	Information included in the manuscript?	In which section is the information available? (Reagents and Tools Table, Materials and Methods, Figures, Data Availability Section)
Could your study fall under dual use research restrictions? Please check biosecurity documents and list of **select agents and toxins** (CDC): https://www.selectagents.gov/sat/list.htm	Not Applicable	Not Applicable
If you used a select agent, is the security level of the lab appropriate and reported in the manuscript?	Not Applicable	Not Applicable
If a study is subject to dual use research of concern regulations, is the name of the **authority granting approval and reference number** for the regulatory approval provided in the manuscript?	Not Applicable	Not Applicable

Reporting

The MDAR framework recommends adoption of discipline-specific guidelines, established and endorsed through community initiatives. Journals have their own policy about requiring specific guidelines and recommendations to complement MDAR.

Adherence to community standards	Information included in the manuscript?	In which section is the information available? (Reagents and Tools Table, Materials and Methods, Figures, Data Availability Section)
State if relevant guidelines or checklists (e.g., **ICMJE, MIBBI, ARRIVE, PRISMA**) have been followed or provided.	Not Applicable	Not Applicable
For **tumor marker prognostic studies,** we recommend that you follow the **REMARK** reporting guidelines (see link list at top right). See author guidelines, under 'Reporting Guidelines'. Please confirm you have followed these guidelines.	Not Applicable	Not Applicable
For **phase II and III randomized controlled trials**, please refer to the **CONSORT** flow diagram (see link list at top right) and submit the CONSORT checklist (see link list at top right) with your submission. See author guidelines, under 'Reporting Guidelines'. Please confirm you have submitted this list.	Not Applicable	Not Applicable

Data Availability

Data availability	Information included in the manuscript?	In which section is the information available? (Reagents and Tools Table, Materials and Methods, Figures, Data Availability Section)
Have **primary datasets** been deposited according to the journal's guidelines (see 'Data Deposition' section) and the respective accession numbers provided in the Data Availability Section?	Yes	Data Availability Section
Were **human clinical and genomic datasets** deposited in a public access-controlled repository in accordance to ethical obligations to the patients and to the applicable consent agreement?	Not Applicable	Not Applicable
Are **computational models** that are central and integral to a study available without restrictions in a machine-readable form? Were the relevant accession numbers or links provided?	Not Applicable	Not Applicable
If publicly available data were reused, provide the respective **data citations in the reference list**.	Yes	Results
